# Size is Important: Artificial Catalyst Mimics Behavior of Natural Enzymes

**DOI:** 10.1016/j.isci.2020.100960

**Published:** 2020-03-05

**Authors:** Jianzhong Chen, Ilya D. Gridnev

**Affiliations:** 1 Shanghai Key Laboratory for Molecular Engineering of Chiral Drugs, School of Chemistry and Chemical Engineering, Shanghai Jiao Tong University, 800 Dongchuan Road, Shanghai 200240, P. R. China; 2 Department of Chemistry, Graduate School of Science, Tohoku University, Aramaki 3-6. Aoba-ku, Sendai 8578, Japan

**Keywords:** Catalysis, Organic Reaction, Computational Chemistry

## Abstract

Heavily substituted (*R*)-DTBM-SegPHOS is active in the asymmetric Pd(II)-catalyzed hydrogenation or C−O bond cleavage of α-pivaloyloxy-1-(2-furyl)ethanone, whereas (*R*)-SegPHOS fails to catalyze either of these transformations. An extensive network of C−H ··· H−C interactions provided by the heavily substituted phenyl rings of (*R*)-DTBM-SegPHOS leads to increased stabilities of all intermediates and transition states in the corresponding catalytic cycles compared with the unsubstituted analogues. Moreover, formation of the encounter complex and its rearrangement into the reactive species proceeds in a fashion similar to that seen in natural enzymatic reactions. Computations demonstrate that this feature is the origin of enantioselection in asymmetric hydrogenation, since the stable precursor is formed only when the catalyst is approached by one prochiral plane of the substrate.

## Highlights

• Non-covalent interactions substrate-DTBM-SegPHOS Pd are essential for reactivity
• Stereoselectivity is induced during approach of a substrate to the reactive site
• This mechanism of enantioselection mimics enzymatic transformations
• Performance of a catalyst can be improved via increasing the size of its ligand

## Graphical Abstract

**Figure:**
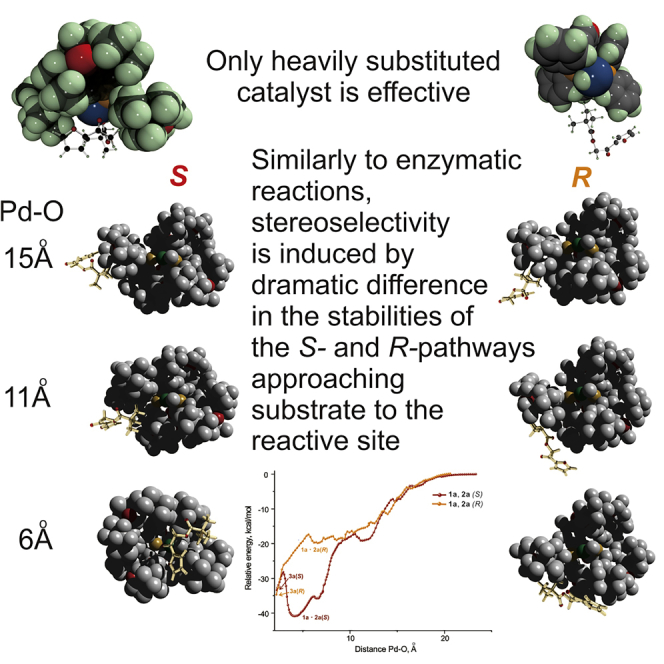

## Introduction

Catalytic reactions constitute the most effective and environmentally friendly transformations available in the contemporary chemical industry. Synthetic studies of various catalytic cycles helped to improve the performance and selectivity levels of practically important transformations. However, the standards seen in the biochemical reactions catalyzed by natural enzymes are not achieved yet.

Although it had been noticed already quite early that the bulkiness of a catalyst usually improves its performance, it had been thought for many years that this is due to the increasing limitation of space remaining for the coordination of the substrate. Recently, it has been recognized, however, that in fact the bulkiness of the ligands increases the number of non-covalent attractive disperse interactions, thus stabilizing intermediates and transition states.

Differing from covalent interactions, which can be easily detected and analyzed in molecular structures, London forces, one of a number of noncovalent interactions, are difficult to observe within the bonding network (Johnson et al., 2010; Wagner and Schreiner, 2015). Recently, it has been recognized that the London dispersion interactions, a type of van der Waals interaction, play an important role in improving reactivity and securing the generation of chirality in the asymmetric catalytic reactions; most examples have been reported in the fields of biochemistry and organocatalysis (Johnson et al., 2010, Wagner and Schreiner, 2015, Cheong et al., 2011, Wheeler et al., 2016, Gridnev and Dub, 2016, Strauss and Wegner, 2019, Neuvonen et al., 2017, Motherwell et al., 2018, Fabrizio and Corminboeuf, 2018, Rösel et al., 2018, Reddi et al., 2019). The significant effects of London dispersion interactions between substrate and ligand in homogeneous transition-metal catalyzed enantioselective transformations have been less widely described (Wang et al., 2011, Ding et al., 2012, Lu et al., 2017, Thomas et al., 2018, Gridnev, 2016, Chen et al., 2018, Li et al., 2019, Lyngvi et al., 2015, Wolters et al., 2015, Sperger et al., 2016, Meyer et al., 2017).

In the mainstream of the theory of molecular catalysis, the selectivity of catalytic reactions is thought to be mainly determined by the difference in stabilities of diastereomeric transition states of the rate-determining stages of the catalytic cycles. The importance of the relative stabilities of the precursors is largely neglected, mostly due to established traditions, although the equilibrium constant reflecting the relative abundance of the precursors is clearly seen in the equation for calculating selectivity (e.g., Equation 1).

In some of our own recent studies, we have found that the approach of the substrate to the catalyst (Liu et al., 2014) or intramolecular rearrangement of the initially formed encounter complex (Imamoto et al., 2012, Gridnev et al., 2014a, Gridnev et al., 2014b, Gridnev and Imamoto, 2015, Gridnev and Imamoto, 2016) was seemingly strongly affecting the high enantioselectivities experimentally observed in these catalytic reactions.

Herein we report for the first time a clear-cut case in which the relative stabilities of the competing transition states are irrelevant for the handedness and order of enantioselection. Instead, the initially formed weak encounter complexes are rearranging, keeping the substrate coordinated via intermolecular C−H ··· H−C interactions, and the existence of a favorable pathway between the encounter complex and the active site discriminates between productive and unfavorable encounter complexes. This behavior mimics the experimentally supported and well-documented mechanism of perfect enantioselectivity in enzymatic transformations (Crowley and Ubbink, 2003, Tang et al., 2006, Xu et al., 2008, Andralojć et al., 2017).

Our computational results reported in this paper demonstrate clearly that:

(1) Numerous additional *t-*Bu (and OMe) substituents seen in the (*R*)-DTBM-SegPHOS provide an extended framework of C−H ··· H−C interactions for coordination of a substrate. In the S-pathway of the asymmetric hydrogenation, an encounter complex is formed already at the long distances between the reactive centers. Furthermore, the substrate remains safely coordinated during its accommodation leading to the formation of highly reactive configuration. The same is not possible for the *R*-pathway that results in a high S-enantioselectivity of the reaction. This completely unrecognized mechanism of enantioselection provides important information for directed catalyst design.
(2) The decisive factor determining the enantioselectivity is the Boltzmann distribution between *S*- and *R-* precursors that reaches approximately 2,300:1, whereas the ratio of the rates of competing reactions is much smaller.
(3) The lack of an extended framework of C−H ··· H−C interactions in the unsubstituted SegPHOS catalyst makes its Pd-complex ineffective.
(4) The extended network of C−H ···H−C interactions is able to stabilize intermediates with significant charge separation, as is seen in the example with the catalytic C-O bond cleavage reaction. This conclusion opens the door for discovering so far unknown catalytic transformations.
(5) The overall picture of effective and stereoselective catalytic transformations outlined in this work closely resembles the highly specific enzymatic reactions taking place in Nature.

## Results and Discussion

### Scope of the Study

**Scheme 1:**
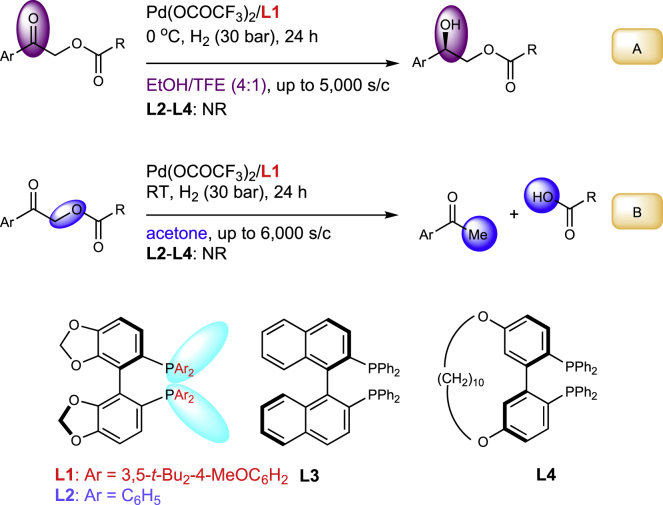
Enantioselective Hydrogenation and C-O Bond Cleavage of α-Acetoxy Ketones

Recently, palladium-catalyzed asymmetric hydrogenations of α-acyloxy-1-arylethanones were reported by our group (Chen et al., 2013) (Scheme 1A). Interestingly, only (*R*)-DTBM-SegPHOS (**L1**) proved to be an effective catalyst (up to 5,000 S/C), whereas structurally similar ligands **L2–L4** with non-substituted phenyl rings were ineffective. Remarkably, application of the same catalytic system to the same substrates in acetone instead of EtOH/TFE led to a different reaction yielding the corresponding ketones even at low catalytic loadings (Chen et al., 2016; Transparent Methods 6) (Scheme 1B). In this case, the application of heavily substituted ligand **L1** was also essential.

**Scheme 2:**
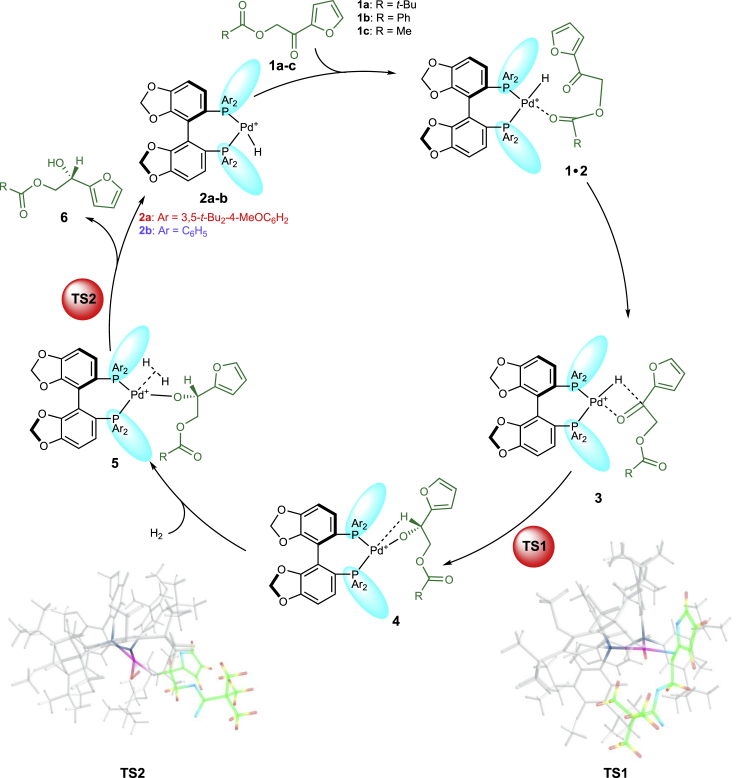
Computed Catalytic Cycles for the Enantioselective Hydrogenation of **1a** with **2a** and **2b**

We have tried to isolate the Pd-complexes and identify palladium hydrogen species of DTBM-SegPHOS by NMR, but our attempts were unsuccessful. Nevertheless, according to the recent mechanistic studies, hydrogenation of the precatalyst in a similar system results in generation of a Pd^+^−H complex **2** that was shown to be an active species (Scheme 2) (Duan et al., 2014). Hence, we investigated the enantioselective reactions of **2a** and **2b** with **1a**-**c** (*t*-Bu, Ph, Me, see Supplemental Information for their enantioselectivities and yields), since **1a** afforded the highest enantioselectivity detected experimentally, whereas **1b** and **1c** provide representative examples of various substituted substrates.

### Catalytic Cycle of Pd-Catalyzed Asymmetric Hydrogenation

**Figure 1:**
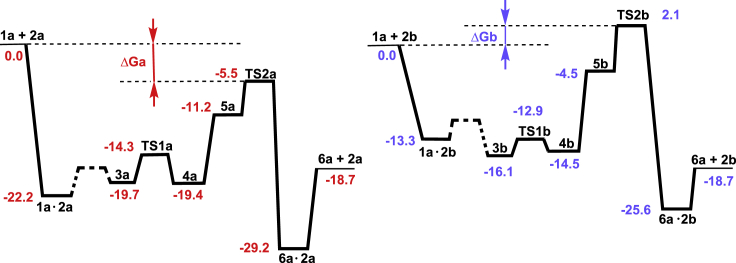
The Energy Profiles of the Catalytic Cycles for the Hydrogenation of **1a** with **2a** and **2b**

**Figure 2:**
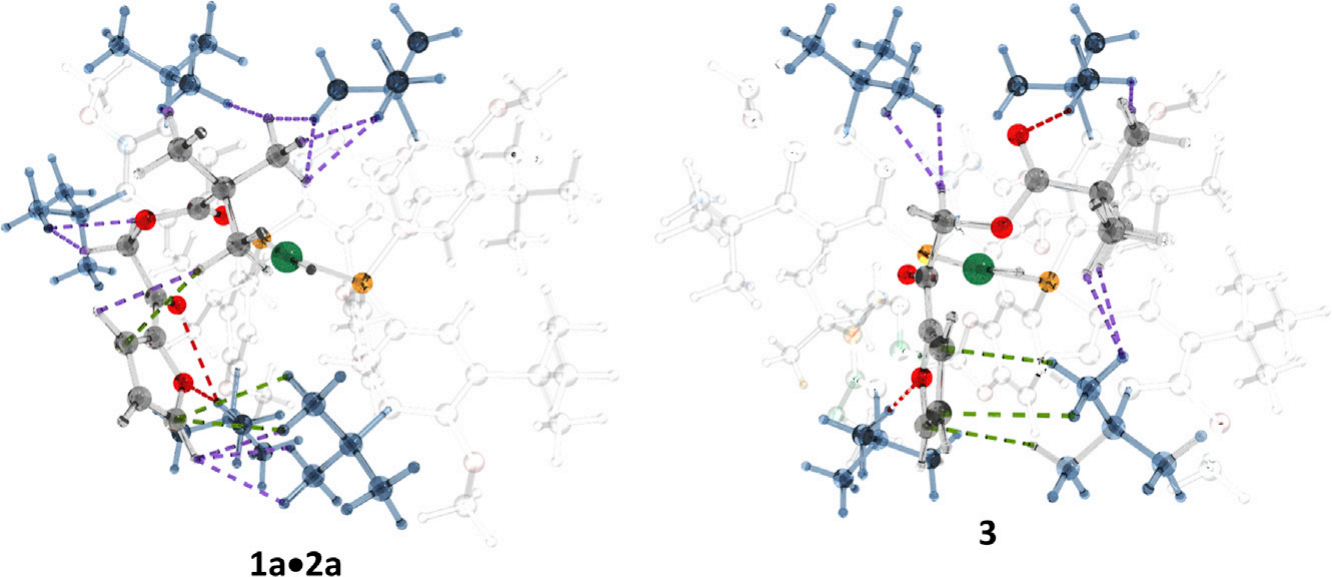
Optimized Structures of **1a·2a** and **3a** and Stabilizing Intramolecular Interactions (Shown in Dashed Lines): C-H ··· H-C (Violet), C-H ··· O (Red) and C-H ··· π (Green) See text for details

The catalytic cycle computed for the hydrogenation of **1a** is shown in Scheme 2. Hydride transfer takes place after the proper orientation of the C=O bond is achieved in the adduct **3a** via **1a·2a**. The resulting alkoxide **4a** coordinates an H_2_ molecule, and insertion of hydride into the Pd-O bond releases the product **6a** and regenerates the catalyst **2a**. Of interest is the strongly exothermic formation of the reactive catalyst-adduct **3a**. Moreover, when calculating the approach of the substrate to the catalyst, we observed the initial formation of an adduct **1a·2a**, which is approximately 12 kcal/mol more stable than **3a** in terms of electronic energy; this results in a 2.5 kcal/mol difference in free energy (Figure 1). The adduct **1a·2a** is formed via numerous weak intramolecular interactions between the *t*-Bu groups of the catalyst and the substrate (see Figure 2). Noteworthy, the highest point of the catalytic cycle, **TS2a**, is 5.5 kcal/mol more stable than the initial mixture of the catalyst and the substrate (Scheme 2, Table 1, entry 1).

**Table 1:** Relative Gibbs Free Energies (kcal/mol) of Intermediates and Transition States of the Catalytic Cycles

Entry	Substrate, Catalyst, Pathway	3	TS1	4	5	TS2	6**·**2
1	**1a, 2a (*S*)**	−19.7	−14.3	−19.4	−11.2	−5.5	−29.2
2	**1a, 2a (*R*)**	−21.2	−14.7	−15.4	−15.0	−8.0	−31.9
3	**1b, 2a (*S*)**	−8.2	−1.9	−9.1	0.2	10.1	−21.6
4	**1b, 2a (*R*)**	−7.1	−1.5	−10.2	−5.3	6.9	−23.6
5	**1c, 2a (*S*)**	−8.8	−2.4	−8.4	−2.9	7.5	−15.9
6	**1c, 2a (*R*)**	−9.8	0.2	−13.6	−0.4	0.8	−19.7
7	**1a, 2b (*S*)**	−16.1	−12.9	−14.5	−4.5	2.1	−25.6
8	**1a, 2b (*R*)**	−17.4	−6.6	−11.9	−1.7	3.4	−25.5
9	**1b, 2b (*S*)**	−8.5	−2.3	−3.5	8.1	21.8	−12.4
10	**1b, 2b (*R*)**	−5.4	3.5	−1.8	5.0	13.6	−15.3
11	**1c, 2b (*S*)**	−5.8	−0.3	−0.6	3.7	13.7	−16.8
12	**1c, 2b (*R*)**	−8.6	2.1	−1.5	5.5	13.8	−18.0

In all cases 0.0 kcal/mol is attributed to the energy of the corresponding pair 1 + 2.

The relative stability of **1a·2a,** as well as of **3a,** is due to the numerous weak intramolecular interactions between the substrate and *t*-Bu groups of the catalyst **2a** (Figure 2). There are ten C-H ··· H-C interactions in the range of 2.21–2.68 Å, two C-H ··· π interactions (2.94 and 3.10 Å), and two C-H ··· O interactions (2.70 and 2.92 Å). To achieve a configuration that is appropriate for rapid hydrogenation (**3a**), half of the C-H ··· H-C interactions must be broken, accounting for the energy difference of 2.5 kcal/mol between **1a·2a** and **3a**.

### Comparison of Three Catalytic Systems

**Figure 3:**
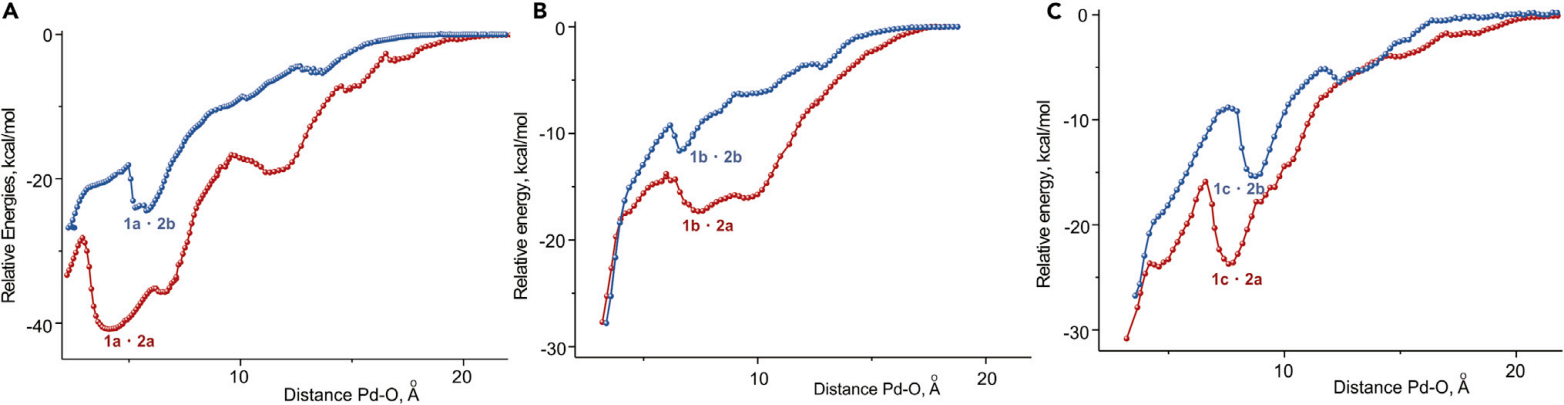
Scans of the Relative Energy Versus the Interatomic Distance Pd-O (A) For **1a** approaching **2a** (red) and **2b** (blue); (B) for **1b** approaching **2a** (red) and **2b** (blue); (C) for **1c** approaching **2a** (red) and **2b** (blue). In each case the energy of separated species is taken as the zero point.

In the case of the computed catalytic cycle for **2b** catalyzed hydrogenation of **1a**, the *t*-Bu groups in the catalyst are absent (Scheme 2, Table 1, entry 7) and the encounter complex **1a·2b** is less stable than the reactive catalyst-substrate complex **3b** (Figure 1, −13.3 kcal/mol versus −16.1 kcal/mol). Moreover, since **TS2b** is 2.1 kcal/mol less stable than **1a** + **2b**, **3b** is more likely to dissociate back to **1a** + **2b**, than to undergo two migratory insertions yielding the hydrogenation product. The importance of *t*-Bu groups within the catalyst for binding the substrate is further illustrated by the computational results for the catalytic cycles with the catalyst **2b** and the substrates **1b, c** (Table 1, entries 9–12) that lack *t*-Bu groups themselves. Graphically, this conclusion is illustrated in Figure 3, which shows that a significant difference in stabilizing energy is observed throughout the whole process for the approach of the substrate to the catalyst; this applies to all three studied examples, although the extent of this stabilization is evidently smaller in the two latter cases.

Interestingly, the observed high *S*-enantioselectivity in the hydrogenation of **1a** with **2a** as a catalyst 97% ee (*S*) cannot be satisfactorily accounted for by the computed parameters of the catalytic cycles using the common approach based on the relative stabilities of the corresponding transition states. Indeed, the relative stabilities of either **TS1** or **TS2** for *S*- and *R*-pathways do not demonstrate the regularity expected in the case if the stereoselection was to take place in either or both of these states.

**Figure 4:**
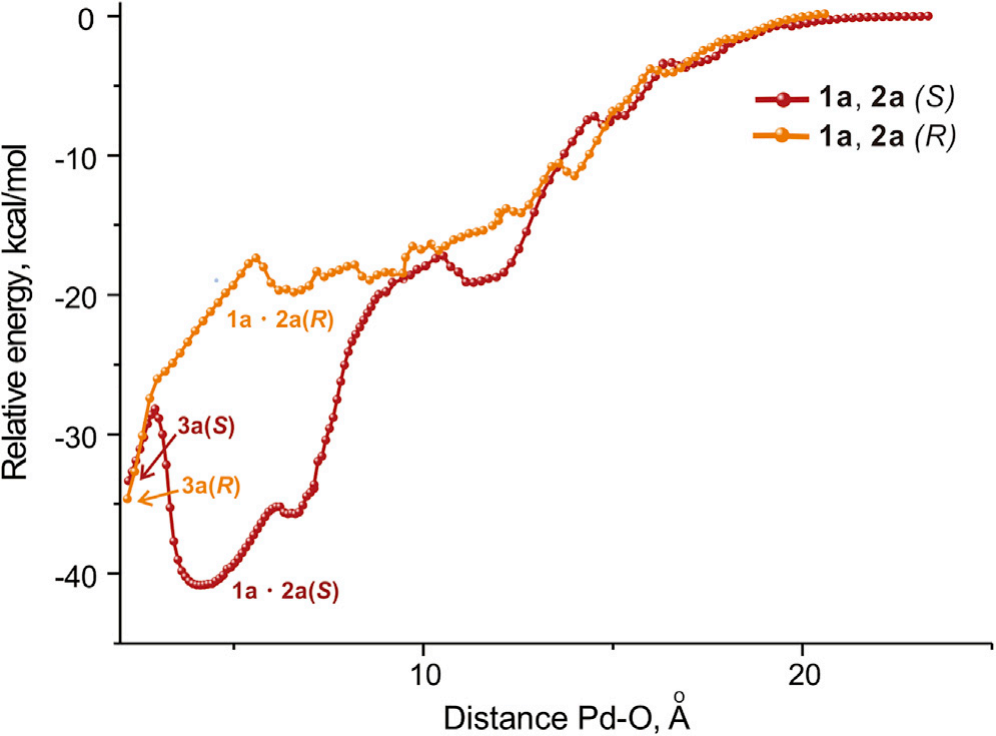
Scans of the Relative Energy Versus the Interatomic Distance Pd-O **1a** approaching **2a** to form S-product (red) and R-product (orange). Note the similarity of the orange curve to the blue curve in the Figure 3A—both belong to reactions that do not take place.

**Figure 5:**
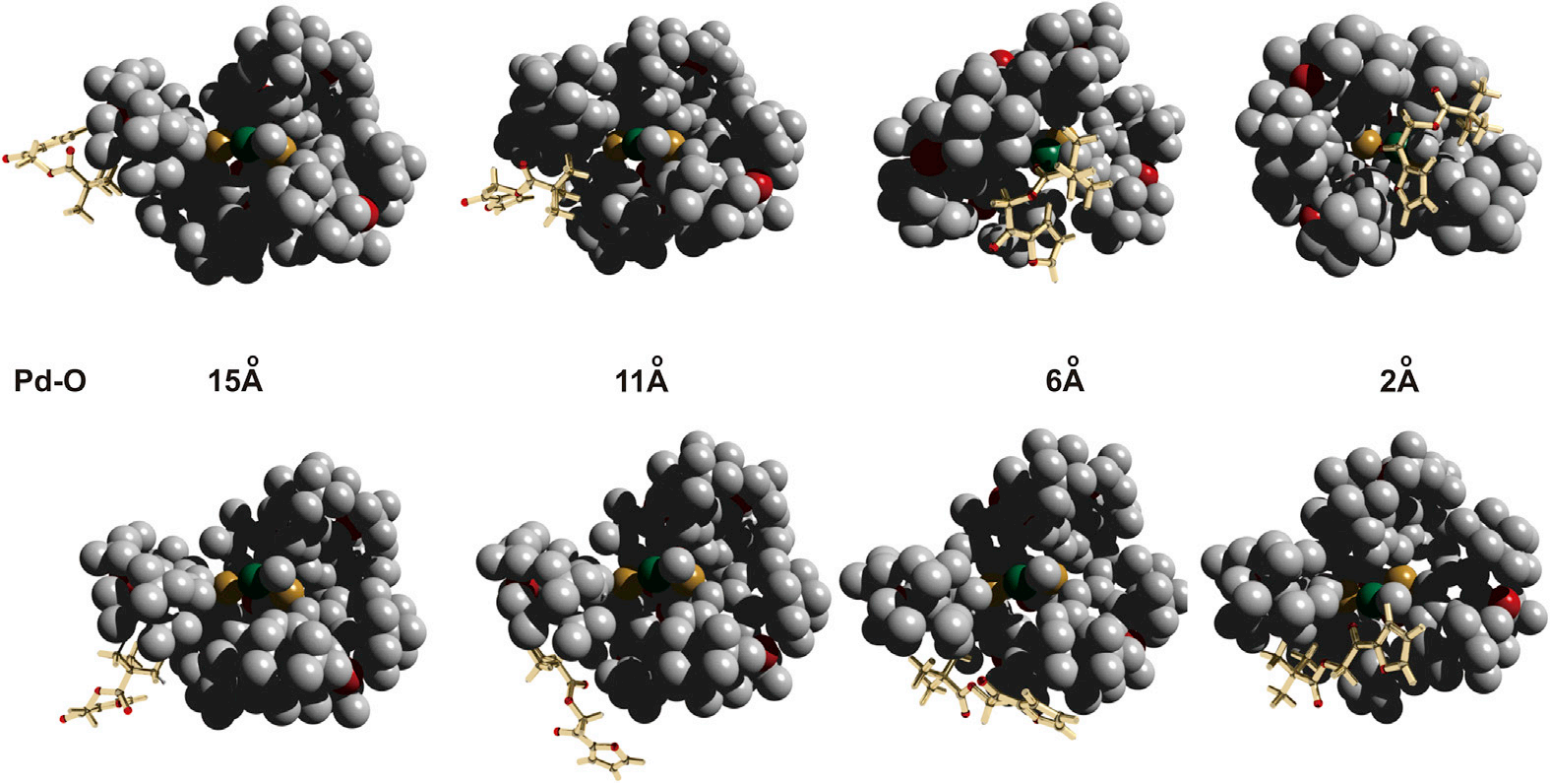
Structures of the Catalyst-Product Adducts at Different Distances Pd-O Top: In the case of the *S*-pathway, the substrate can approach the reactive site moving in the valley between several t-Bu groups keeping numerous C-H interactions to reach the deep minimum with the oxygen atom of t-BuCO group coordinated to Pd. Bottom: The same level of stabilization cannot be achieved in the R-pathway, because the C_4_H_3_OCO group (C_4_H_3_O: furyl) must be kept dissociated to be able to switch the prochiral planes. Even at 15 Å the modes of coordination are different; therefore, to cross-over the pathways, the substrate must come back at least thus far.

To gain further insight into the mechanism of enantioselection in this case, we have studied in detail the process of the formation of reactive complexes **3(*S*)** and **3(*R*)**. Inspecting Figures 4 and 5, one can see that the association pathways differ significantly starting from the Pd-O distances of approximately 15 Å. Moreover, a deep minimum corresponding to the formation of **1a·2b** is not observed in the case of the *R-*pathway. Therefore, almost complete dissociation must take place for the convergence of these pathways, and the enantioselectivity is largely determined by their relative abundance.

Since the *R*-pathway does not have really deep minima, we were unable to estimate accurately the free energy gap between two pathways. For an approximate estimation we used the coefficient 4.8 obtained above from comparing the differences in electronic energies and Gibbs free energies between **1a·2a(*S*)** and **3a(*S*)** (*vide supra*). Thus, the difference of 22 kcal/mol (see Figure 4) approximately corresponds to 4.6 kcal/mol difference in Gibbs free energy for **1a·2a(*S*)** and **1a·2a(*R*)** that gives a 2,343:1 ratio of precursors.

In the case of complete absence of exchange between the precursors, the optical yield is determined entirely by their relative abundance (Halpern, 1982, Landis and Halpern, 1987, Drexler et al., 2005, Schmidt et al., 2008, Hartwig, 2010) and the estimated value of the free energy difference attests for the perfect *S*-enantioselection, whereas the experimental ee value is 97% ee (*S*).

In the Curtin-Hammett conditions (all equilibria between the precursors are faster than productive transformations) the ee should be estimated by Equation 1 (Hartwig, 2010).

(Equation 1) $$\frac{\left[S\right]}{\left[R\right]}=K_{eq}\left(\frac{k_{i}}{k_{i^{\text{'}}}}\right)$$

Using Equation 1, data of Table 1 for **TS1** and **TS2**, and assuming instant achievement of equilibrium, we can obtain:

[*S*]/[*R*] = e^4.6/0.593^ x (1/e^0.4/0.593^ x 1/e^2.5/0.593^) = 2343 x 1/1.95 x 1/68 = 17.7

That corresponds to 89% ee (*S*), an underestimation, but still clearly indicating the strong *S*-bias.

We conclude therefore that the enantioselection in the reaction under study is determined by the relative abundance of the precursors, which is a relatively rare case, although there is precedence of such a phenomenon (Liu et al., 2014, Drexler et al., 2005, Schmidt et al., 2008).

We are convinced that the presented evidence demonstrates an important feature of bulky catalysts, approaching in size to natural enzymes and able to participate in a large number of weak intramolecular interactions with the substrate. Similarly to the enzymatic catalysis (Strauss and Wegner, 2019), the initial weak encounter complexes are formed not selectively (Pd-O distances from 20 to 15 Å in the Figure 4). But the pathways for the closer association ultimately leading to the configuration appropriate for a very fast transformation are significantly different (Figure 5), thus ensuring high selectivity of the overall reaction.

It is clear that similar differences in the ability to bind the substrate and keep it in a proper conformation must be observed in the destructive C−O bond cleavage reaction that can also be catalyzed by the palladium complex of the ligand **L1**, whereas the palladium complex of **L2** is ineffective.

### Catalytic Cycle of Pd-Catalyzed C-O Bond Cleavage

**Scheme 3:**
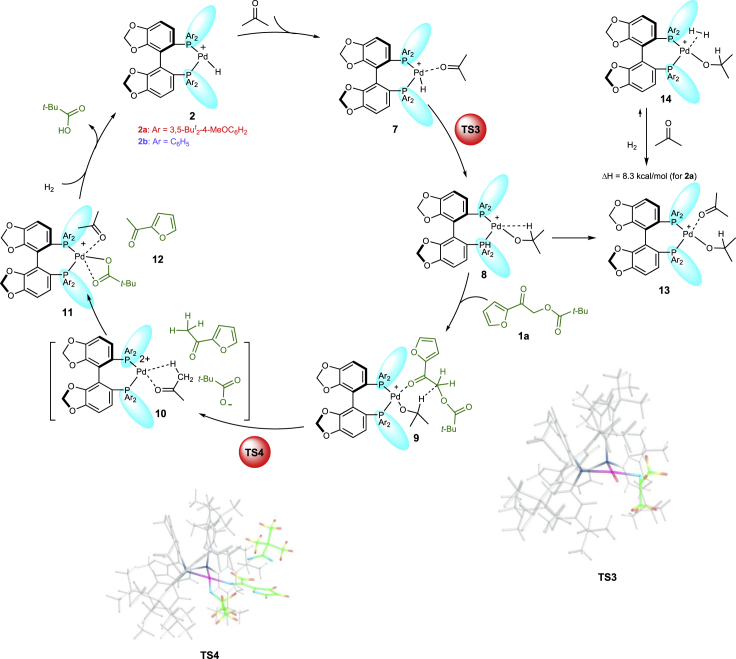
Mechanism of the C−O Bond Cleavage

We have investigated several possibilities for the hydride source in the destructive hydrogenation and have found that neither Pd−H species like **3** nor Pd−H_2_ species like **5** (Scheme 2) were capable of promoting the hydride transfer resulting in the destructive C−O bond cleavage leading to the corresponding product **12** (Scheme 3) (Transparent Methods 5). On the other hand, it was possible to locate transition states for the hydride transfer starting from the acetone alcoholate **8**, which can easily be formed from complex **7** under the reaction conditions through the transition state **TS3**. It is not further hydrogenated to 2-propanol because, in the presence of excess acetone, the formation of molecular hydrogen complex **14** is strongly endogonic (Scheme 3). On the other hand, **1a** is firmly bound via the numerous weak intramolecular interactions, similar to the previous case.

**Figure 6:**
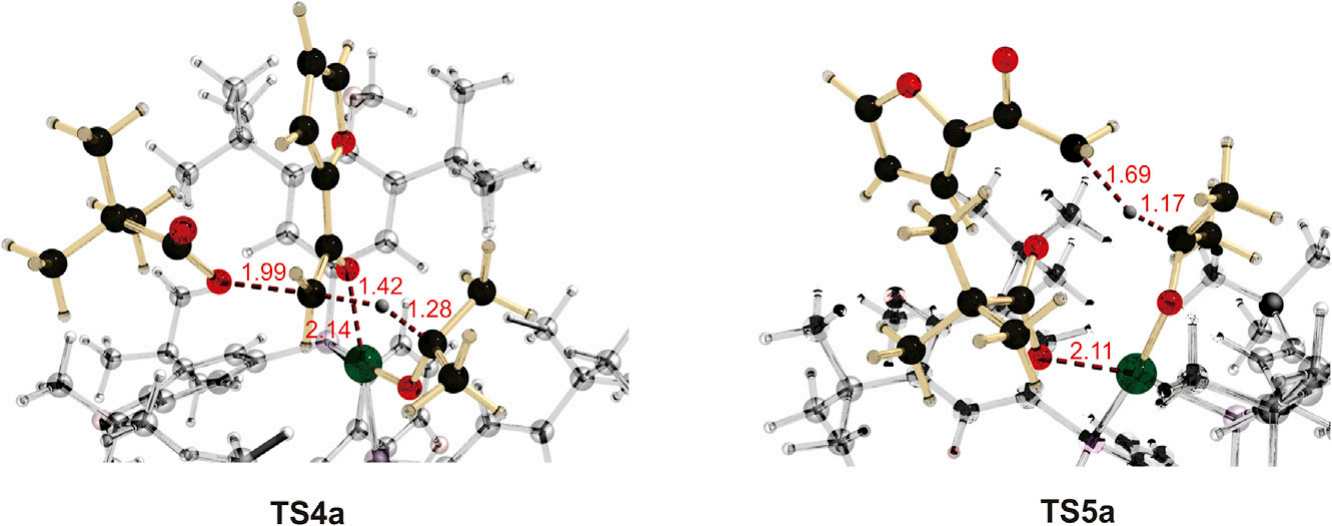
Partial Plots of the Transition States for the Destructive C−O Bond Cleavage In TS4a, the hydride originating from the isopropyl alcoholate substitutes the Opiv anion in an SN2 fashion. The alternative mode of substitution via TS5a is disfavoured by more than 30 kcal/mol (32.2 kcal/mol). Numbers indicate interatomic distances in Angström.

Initially, we expected that the OPiv-anion (Piv: pivaloyl) must remain attached to the Pd after the C−O bond is broken. However, the calculations showed that, in the case of this orientation of the substrate, the hydride must approach the carbon atom from the side of the broken C−O bond (Figure 6, **TS5a**). This makes this TS more than 30 kcal/mol less stable than another TS (Figure 6, **TS4a**) whereby the keto group of the substrate is coordinated to Pd and the OPiv group is replaced by the hydride similar to the usual S_N_2 reaction. Despite the charges separation, the transformation from **9** to **10** was computed to proceed with an activation barrier of 28.3 kcal/mol; this is in reasonable agreement with the experimental results (Lu et al., 2017) (For the mechanism experiments see Table S1). The latter structure is not a real minimum. It spontaneously rearranges to the much more stable product **11** (Scheme 3), resulting in a strongly exogonic (41.7 kcal/mol) transformation. The transition state **TS4b** for the Pd-SEGPHOS (Pd-**L2**) catalysis corresponds to an activation barrier of 44.8 kcal/mol.

We have detected the production of a small amount of isopropanol in the reaction mixture by GC-MS (Transparent Methods 6, Table S1) (The substrate **1a** [R = furyl] had not been previously tried in the destructive C−O bond cleavage reaction. Hence, the corresponding experiment was carried out. Complete conversion of **1a** was achieved after 24 h reaction at 80°C and 50 atm of H_2_. See Supplemental Information for detail.). This result revealed the possibility that acetone takes part in the hydride transfer.

**Figure 7:**
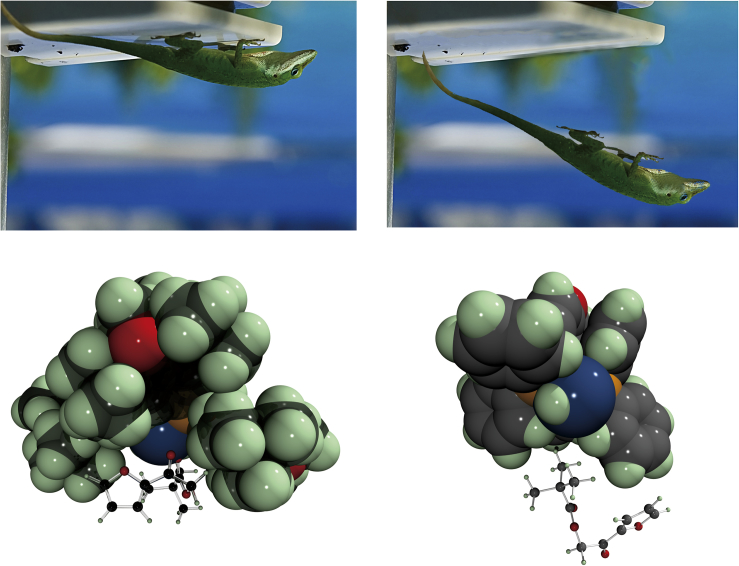
Gecko-Friendly Catalyst A Gecko-friendly catalyst like **2a** (left) allows a substrate to remain bound to the catalyst through numerous London interactions long before the appropriate conformation for effectuating the reaction is achieved. Lack of Gecko-friendly structural elements in **2b** prevents the reaction that would be otherwise possible.

It is known that numerous London dispersion forces allow the Gecko a possibility to walk on walls or ceilings keeping its body upside down. The abundance of *t*-Bu groups in the molecule of the catalyst **2a** provides opportunity for a substrate to overcome Brownian motion and remain bound to the catalyst long enough to allow the reaction to proceed. Such types of catalyst can therefore be considered to be “Gecko-friendly” catalysts, as illustrated in the Figure 7. This concept suggests that an improvement in the catalytic performance of an artificial catalyst can be achieved by increasing the size of the ligand via introducing numerous *t*-Bu groups or similar substituents capable of forming catalyst-substrate adducts stabilized by non-bonding interactions.

In summary, we have discovered a catalytic reaction in which the relative stabilities of the competing transition states are irrelevant for the handedness and order of enantioselection. Similar to natural enzymatic reactions, high enantioselectivity is achieved via the stereodivergent approach of the substrate to the reactive site of the catalyst. Initially, the encounter complex is formed, which later rearranges into active species capable for extremely rapid transformation into the product. This process occurs owing to the existence of an extensive network of C−H ··· H−C interactions provided by the numerous *t*-Bu groups of the DTBM-SegPHOS ligand. Stereoselectivity is induced by dramatic difference in the stabilities of the *S*- and *R*-pathways of the approaching substrate to the reactive site. The mechanism of stereoselection mimics highly efficient enzymatic transformations taking place in Nature.

The normal SegPHOS ligand lacking the *t*-Bu groups is unable to promote the same catalytic transformations. The extended network of C−H ··· H−C interactions is able to stabilize intermediates with significant charge separation, as is seen in the example concerning catalytic C-O bond cleavage reaction.

### Limitations of the Study

The results of a purely computational study are presented here. Evidently, experimental research on the catalyst-substrate interactions for large-sized artificial catalysts must be induced by our findings.

## Methods

All methods can be found in the accompanying Transparent Methods supplemental file.

## Supplemental Information

Document S1. Transparent Methods, Figures S1–S4, Table S1, and Data S1

## Footnotes

Supplemental Information can be found online at https://doi.org/10.1016/j.isci.2020.100960.

## References

[bib1] Andralojć W., Hiruma Y., Liu W., Ravera E., Nojiri M., Parigi G., Luchinat C., Ubbink M. (2017). Identification of productive and futile encounters in an electron transfer protein complex. Proc. Nat. Acad. Sci. U. S. A.

[bib2] Chen J., Liu D., Butt N., Li C., Fan D., Liu Y., Zhang W. (2013). Palladium-catalyzed asymmetric hydrogenation of α-Acyloxy-1-arylethanones. Angew. Chem. Int. Ed..

[bib3] Chen J., Zhang Z., Liu D., Zhang W. (2016). Palladium-catalyzed chemo- and enantioselective C-O bond cleavage of α-acyloxy ketones by hydrogenolysis. Angew. Chem. Int. Ed..

[bib4] Chen J., Zhang Z., Li B., Li F., Wang Y., Zhao M., Gridnev I.D., Imamoto T., Zhang W. (2018). Pd(OAc)2-Catalyzed asymmetric hydrogenation of sterically hindered N-tosylimines. Nat. Commun..

[bib5] Cheong P.H.-Y., Legault C.Y., Um J.M., ÇeIebi-Ölçüm N., Houk K.N. (2011). Quantum mechanical investigations of organocatalysis: mechanisms, reactivities, and selectivities. Chem. Rev..

[bib6] Crowley P.B., Ubbink M. (2003). Close encounters of the transient kind: protein interactions in the photosynthetic redox chain investigated by NMR spectroscopy. Acc. Chem. Res..

[bib7] Ding Z.-Y., Chen F., Qin J., He Y.-M., Fan Q.-H. (2012). Asymmetric hydrogenation of 2,4-disubstituted 1,5-benzodiazepines using cationic ruthenium diamine catalysts: an unusual achiral counteranion induced reversal of enantioselectivity. Angew. Chem. Int. Ed..

[bib8] Drexler H.-J., Baumann W., Schmidt T., Zhang S., Sun A., Spannenberg A., Fischer C., Buschmann H., Heller D. (2005). Are β-Aminoacrylates hydrogenated in the same way as α-aminoacrylates?. Angew. Chem. Int. Ed..

[bib9] Duan Y., Li L., Chen M.-W., Yu C.-B., Fan H.-J., Zhou Y.-G. (2014). Homogenous Pd-catalyzed asymmetric hydrogenation of unprotected indoles: scope and mechanistic studies. J. Am. Chem. Soc..

[bib10] Fabrizio A., Corminboeuf C. (2018). How do London dispersion interactions impact the photochemical processes of molecular switches?. J. Phys. Chem. Lett..

[bib11] Gridnev I.D. (2016). Attraction versus repulsion in rhodium-catalyzed asymmetric hydrogenation. ChemCatChem.

[bib12] Gridnev I.D., Imamoto T. (2015). Challenging the major/minor concept in Rh-catalyzed asymmetric hydrogenation. ACS Catal..

[bib13] Gridnev I.D., Imamoto T. (2016). Enantioselection mechanism in Rh- catalyzed asymmetric hydrogenation. Russ. Chem. Bull..

[bib14] Gridnev I.D., Liu Y., Imamoto T. (2014). Mechanism of asymmetric hydrogenation of β-Dehydroamino acids catalyzed by rhodium complexes: large-scale experimental and computational study. ACS Catal..

[bib15] Gridnev I.D., Kohrt C., Liu Y. (2014). Direct experimental and computational evidence for the Dihydride pathway in TangPHOS-Rh catalysed asymmetric hydrogenation. Dalton Trans..

[bib16] Gridnev I.D., Dub P.A. (2016). Enantioselection in Asymmetric Catalysis.

[bib17] Halpern J. (1982). Mechanism and stereoselectivity of asymmetric hydrogenation. Science.

[bib18] Hartwig J. (2010). Organotransition Metal Chemistry. From Bonding to Catalysis.

[bib19] Imamoto T., Tamura K., Zhang Z., Horiuchi Y., Sugiya M., Yoshida K., Yanagisawa A., Gridnev I.D. (2012). Rigid P-chiral phosphine ligands with tert-butylmethylphosphino groups for rhodium-catalyzed asymmetric hydrogenation of functionalized alkenes. J. Am. Chem. Soc..

[bib20] Johnson E.R., Keinan S., Mori-Sánchez P., Contreras-García J., Cohen A.J., Yang W. (2010). Revealing noncovalent interactions. J. Am. Chem. Soc..

[bib21] Landis C.R., Halpern J. (1987). Asymmetric hydrogenation of methyl (Z)-α-acetamido-cinnamate catalyzed by [1,2-bis(phenyl-o-anisoyl)phosphino)ethane]-rhodium(I): kinetics, mechanism and origin of enantioselection. J. Am. Chem. Soc..

[bib22] Li B., Chen J., Zhang Z., Gridnev I.D., Zhang W. (2019). Nickel-catalyzed asymmetric hydrogenation of N-sulfonyl imines. Angew. Chem. Int. Ed..

[bib23] Liu Y., Gridnev I.D., Zhang W. (2014). Mechanism of the asymmetric hydrogenation of exocyclic α, β-unsaturated carbonyl compounds with an iridium/BiphPhox catalyst: NMR and DFT studies. Angew. Chem. Int. Ed..

[bib24] Lu G., Liu R.Y., Yang Y., Fang C., Lambrecht D.S., Buchwald S.L., Liu P. (2017). Ligand—substrate dispersion facilitates the copper-catalyzed hydroamination of unactivated olefins. J. Am. Chem. Soc..

[bib25] Lyngvi E., Sanhueza I.A., Schoenebeck F. (2015). Dispersion makes the difference: bisligated transition states found for the oxidative addition of Pd(PtBu_3_)_2_ to Ar-OSO2R and dispersion-controlled chemoselectivity in reactions with Pd[P(iPr)(tBu_2_)]_2_. Organometallics.

[bib26] Meyer T.H., Liu W., Feldt M., Wuttke A., Mata R.A., Ackermann L. (2017). Manganese(I)-Catalyzed dispersion-enabled C-H/C-C activation. Chem. Eur. J..

[bib27] Motherwell W.B., Moreno R.B., Pavlakos I., Arendorf J.R.T., Arif T., Tizzard G.J., Coles S.J., Aliev A.E. (2018). Noncovalent interactions of p systems with sulfur: the atomic chameleon of molecular recognition. Angew. Chem. Int. Ed..

[bib28] Neuvonen A.J., Földes T., Madarász Á., Pápai I., Pihko P.M. (2017). Organocatalysts fold to generate an active site pocket for the mannich reaction. ACS Catal..

[bib29] Reddi Y., Tsai C.-C., Avila C.M., Toste F.D., Sunoj R.B. (2019). Harnessing noncovalent interactions in dual-catalytic enantioselective heck—matsuda arylation. J. Am. Chem. Soc..

[bib30] Rösel S., Becker J., Allen W.D., Schreiner P.R. (2018). Probing the delicate balance between pauli repulsion and London dispersion with triphenylmethyl derivatives. J. Am. Chem. Soc..

[bib31] Schmidt T., Dai Z., Drexler H.-J., Marko M., Preetz A., Heller D. (2008). The major/minor concept: dependence of the selectivity of homogeneously catalyzed reactions on reactivity ratio and concentration ratio of the intermediates. Chem. Asian J..

[bib32] Sperger T., Sanhueza I.A., Schoenebeck F. (2016). Computation and experiment: a powerful combination to understand and predict reactivities. Acc. Chem. Res..

[bib33] Strauss M.A., Wegner H.A. (2019). Molecular systems for the quantification of London dispersion interactions. Eur. J. Org. Chem..

[bib34] Tang C., Iwahara J., Clore G.M. (2006). Visualization of transient encounter complexes in protein–protein association. Nature.

[bib35] Thomas A.A., Speck K., Kevlishvili I., Lu Z., Liu P., Buchwald S.L. (2018). Mechanistically guided design of ligands that significantly improve the efficiency of CuH-catalyzed hydroamination reactions. J. Am. Chem. Soc..

[bib36] Wagner J.P., Schreiner P.R. (2015). London dispersion in molecular chemistry—reconsidering steric effects. Angew. Chem. Int. Ed..

[bib37] Wang T., Zhuo L.-G., Li Z., Chen F., Ding Z., He Y., Fan Q.-H., Xiang J., Yu Z.-X., Chan A.S.C. (2011). Highly enantioselective hydrogenation of quinolines using phosphine-free chiral cationic ruthenium catalysts: scope, mechanism, and origin of enantioselectivity. J. Am. Chem. Soc..

[bib38] Wheeler S.E., Seguin T.J., Guan Y., Doney A.C. (2016). Noncovalent interactions in organocatalysis and the prospect of computational catalyst design. Acc. Chem. Res..

[bib39] Wolters L.P., Koekkoek R., Bickelhaupt F.M. (2015). Role of steric attraction and bite-angle flexibility in metal-mediated C−H bond activation. ACS Catal..

[bib40] Xu X., Reinle W., Hannemann F., Konarev P.V., Svergun D.I., Bernhardt R., Ubbink M. (2008). Dynamics in a pure encounter complex of two proteins studied by solution scattering and paramagnetic NMR spectroscopy. J. Am. Chem. Soc..

