# Supplementary material for: Size is Important: Artificial Catalyst Mimics Behavior of Natural Enzymes
Source: iScience. 2020 Mar 5;23(3):100960. doi: 10.1016/j.isci.2020.100960 (PMC7076558; doi:10.1016/j.isci.2020.100960)

iScience, Volume *23*

Supplemental Information

Size is Important: Artificial Catalyst Mimics Behavior of Natural Enzymes

Jianzhong Chen and Ilya D. Gridnev

Supplemental Information

Contents

1. Computational details 2
2. Data S1. Cartesian coordinates (Related to Table 1) 3
   - 2.1 Catalysts, starting compounds and products 3
   - 2.2 Catalytic cycle **2a** – **1a** (*S*) 6
   - 2.3 Catalytic cycle **2a** – **1a** (*R*) 17
   - 2.4 Catalytic cycle **2a** – **1b** (*S*) 26
   - 2.5 Catalytic cycle **2a** – **1b** (*R*) 35
   - 2.6 Catalytic cycle **2a** – **1c** (*S*) 44
   - 2.7 Catalytic cycle **2a** – **1c** (*R*) 52
   - 2.8 Catalytic cycle **2b** – **1a** (*S*) 61
   - 2.9 Catalytic cycle **2b** – **1a** (*R*) 66
   - 2.10 Catalytic cycle **2b** – **1b** (*S*) 70
   - 2.11 Catalytic cycle **2b** – **1b** (*R*) 75
   - 2.12 Catalytic cycle **2b** – **1c** (*S*) 79
   - 2.13 Catalytic cycle **2a** – **1c** (*R*) 83
   - 2.14 Catalytic cycle for destructive hydrogenation with **2a** 87
   - 2.15 Catalytic cycle for destructive hydrogenation with **2b** 99
3. General Details 105
4. Asymmetric Hydrogenation 105
5. Hydrogenolysis 108
6. Mechanism Experiments Related to Scheme 3 109
   - *6.1 Table S1.* The results of mechanism experiments 109
   - *6.2* Figure S1. ESI mass-spectra of the reaction mixtures described in the Table S1. d_6-Acetone+H_2 (entry 3, peak, m/z = 48) 111
   - *6.3* Figure S2. ESI mass-spectra of the reaction mixtures described in the Table S1: Acetone+H_2 (entry 2 and 4, peak, m/z = 45) 112
   - *6.4* Figure S3. ESI mass-spectra of the reaction mixtures described in the Table S1: d_6-Acetone (standard sample, peak, m/z = 46) 113

6.5 Figure S4. ESI mass-spectra of the reaction mixtures described in the Table S1: d_6-Acetone+H_2 (entry 3, peak, m/z = 48) 114

Transparent Methods

1. Computational details

Computations were carried out using the range separated hybrid functional with damped atom-atom dispersion (WB97XD) (Chai, J.-D., and Head-Gordon, M., 2008) as implemented in the GAUSSIAN 09 software package. (Frisch et al. 2009). For palladium atom the SDD basis set (Bergner, A. et al. 1993) with the associated effective core potential was employed. All other atoms were described with 6-31G** basis with additional diffuse function for phosphorus (Ditchfield, R. et al., 1971; Gordon, M. S., 1980; Hariharan, P. C., and Pople, J. A., 1973; 1974; Hehre, W. J. et al, 1972). Non-specific solvation was introduced by using the SMD continuum model (Marenich, A. V. et al., 2009) (ethanol or acetone for hydrogenation and destructive cleavage respectively).

Bergner, A., Dolg, M., Kuechle, W., Stoll, H., and Preuss, H. ab Initio Energy-Adjusted Pseudopotentials For Elements Of Groups 13–17. (1993). Mol. Phys., 80, 1431-1441.

Chai, J.-D., and Head-Gordon, M. Long-Range Corrected Hybrid Density Functionals with Damped Atom–Atom Dispersion Corrections. (2008). Phys. Chem. Chem. Phys., 2008, 10, 6615-6620.

Ditchfield, R., Hehre, W. J., and Pople, J. A. Self‐Consistent Molecular‐Orbital Methods. IX. An Extended‐IX. An Extended Gaussian‐Type Basis for Molecular‐Orbital Studies of Organic Molecules. (1971). J. Chem. Phys., 54, 724-728.

Frisch, M. J., Trucks, G. W., Schlegel, H. B., Scuseria, G. E., Robb, M. A., Cheeseman, J. R., Scalmani, G., Barone, V., Mennucci, B., Petersson, G. A., Nakatsuji, H., Caricato, M., Li, X., Hratchian, H. P., Izmaylov, A. F., Bloino, J., Zheng, G., Sonnenberg, J. L., Hada, M., Ehara, M.; Toyota, K., Fukuda, R., Hasegawa, J., Ishida, M., Nakajima, T., Honda, Y., Kitao, O., Nakai, H., Vreven, T., Montgomery, Jr., J. A., Peralta, J. E., Ogliaro, F., Bearpark, M., Heyd, J. J., Brothers, E., Kudin, K. N., Staroverov, V. N., Kobayashi, R., Normand, J., Raghavachari, K., Rendell, A., Burant, J. C., Iyengar, S. S., Tomasi, J., Cossi, M., Rega, N., Millam, J. M., Klene, M., Knox, J. E., Cross, J. B., Bakken, V., Adamo, C., Jaramillo, J., Gomperts, R., Stratmann, R. E., Yazyev, O., Austin, A. J., Cammi, R., Pomelli, C., Ochterski, J. W., Martin, R. L., Morokuma, K., Zakrzewski, V. G., Voth, G. A., Salvador, P., Dannenberg, J. J., Dapprich, S., Daniels, A. D., Farkas, O., Foresman, J. B., Ortiz, J. V., Cioslowski, J., and Fox, D. J. Gaussian 09, Revision D.01, Gaussian, Inc., Wallingford CT, 2009.

Gordon, M. S. The Isomers of Silacyclopropane. (1980). Chem. Phys. Lett., 76, 163-168.

Hariharan, P. C., and Pople, J. A. The Influence Of Polarization Functions On Molecular Orbital Hydrogenation Energies (1973) Theor. Chim. Acta, 28, 213-222.

Hariharan, P. C., and Pople, J. A. Accuracy Of AH Equilibrium Geometries By Single Determinant Molecular-Orbital Theory (1974) Mol. Phys., 27, 209-214.

Hehre, W. J., Ditchfield, R., and Pople, J. A. Self—Consistent Molecular Orbital Methods. XII. Further Extensions of Gaussian—Type Basis Sets for Use in Molecular Orbital Studies of Organic Molecules. (1972) J. Chem. Phys., 56, 2257-2261.

Marenich, A. V., Cramer, C. J., and Truhlar, D. G. Universal Solvation Model Based on Solute Electron Density (2009) J. Phys. Chem. B, 2009, 113, 6378-6396.

2. Data S1. Cartesian coordinates. Related to Table 1.

2.1 Catalysts, starting compounds and products.

H_2

RwB97XD SCF energy -1.174642 a.u
RwB97XD SCF enthalpy -1.161194 a.u
RwB97XD SCF free energy -1.175987 a.u

2a

RwB97XD SCF energy -4292.091722 a.u.
RwB97XD SCF enthalpy -4290.379889 a.u.
RwB97XD SCF free energy -4290.590561 a.u.
Three lowest frequencies (cm^{-1}) 16.3, 18.4, 21.1

Cartesian coordinates:

1	6	0	-0.325089	-0.818663	1.922347
2	6	0	-0.114124	-1.784125	2.884063
3	6	0	-0.706020	-3.043061	2.852126
4	6	0	-1.531480	-3.422093	1.823313
5	6	0	-1.760300	-2.465603	0.825062
6	6	0	-1.195995	-1.188679	0.856769
7	1	0	-2.000885	-4.397834	1.791194
8	1	0	-2.426853	-2.740087	0.017172
9	6	0	0.320970	0.506569	2.135237
10	6	0	1.349322	1.072014	1.333727
11	6	0	-0.029347	1.227257	3.256810
12	6	0	1.909299	2.303448	1.665304
13	6	0	0.540630	2.452971	3.584025
14	6	0	1.509535	3.028866	2.799404
15	1	0	2.693867	2.721970	1.044978
16	1	0	1.959763	3.982391	3.048858
17	8	0	-0.327827	-3.749504	3.948817
18	8	0	0.675418	-1.689236	3.991731
19	8	0	-0.956332	0.895162	4.200102
20	8	0	-0.021378	2.912183	4.740805
21	6	0	0.289864	-2.791367	4.812019
22	1	0	1.169930	-3.230057	5.278576
23	1	0	-0.441757	-2.452718	5.555252
24	6	0	-0.686946	1.773919	5.291924
25	1	0	-0.018878	1.272111	6.002825
26	1	0	-1.622772	2.081926	5.755714
27	15	0	1.831546	0.193218	-0.193822
28	15	0	-1.583825	-0.040052	-0.517457
29	46	0	0.145091	0.296243	-1.880090
30	6	0	2.279877	-1.487413	0.303445
31	6	0	3.127788	-1.774347	1.363692
32	6	0	1.727189	-2.527655	-0.440388
33	6	0	3.422504	-3.093512	1.728190
34	1	0	3.573951	-0.952163	1.914279
35	6	0	1.931389	-3.859860	-0.100267
36	1	0	1.101810	-2.268017	-1.286224
37	6	0	2.722246	-4.112972	1.046917
38	6	0	3.308396	1.019275	-0.857053
39	6	0	4.563479	0.417559	-0.905243
40	6	0	3.115203	2.245201	-1.486795
41	6	0	5.648938	1.055126	-1.505599
42	1	0	4.684849	-0.565732	-0.470951
43	6	0	4.158133	2.941979	-2.104210
44	1	0	2.118353	2.674993	-1.491813
45	6	0	5.440093	2.359217	-2.020147
46	6	0	-3.049433	-0.769208	-1.305872
47	6	0	-4.335110	-0.368222	-0.964091
48	6	0	-2.870643	-1.766949	-2.254751
49	6	0	-5.456235	-0.983958	-1.516677
50	1	0	-4.461759	0.442410	-0.261635
51	6	0	-3.946457	-2.443587	-2.834504
52	1	0	-1.857357	-2.025819	-2.540304
53	6	0	-5.238239	-2.077530	-2.390262
54	6	0	-2.101125	1.525838	0.228896
55	6	0	-1.675443	2.719000	-0.333727
56	6	0	-2.936398	1.549849	1.349012
57	6	0	-2.123149	3.959170	0.133322
58	1	0	-0.967189	2.682962	-1.155840
59	6	0	-3.466292	2.746203	1.821895
60	1	0	-3.182913	0.614385	1.836474
61	6	0	-3.118873	3.932619	1.126226
62	6	0	-1.439530	5.209560	-0.466035
63	6	0	-1.823625	5.357532	-1.949526
64	1	0	-1.307850	6.219954	-2.386420
65	1	0	-1.542458	4.471361	-2.528973
66	1	0	-2.900355	5.511580	-2.073891
67	6	0	-1.724309	6.533303	0.264590
68	1	0	-1.544170	6.448680	1.339653
69	1	0	-1.041553	7.294054	-0.129516
70	1	0	-2.738764	6.902954	0.111967
71	6	0	0.089535	5.000827	-0.369764
72	1	0	0.405775	4.892817	0.673341
73	1	0	0.435225	4.121286	-0.919511
74	1	0	0.599578	5.871621	-0.793551
75	6	0	-4.322397	2.771382	3.103906
76	6	0	-3.649794	3.710624	4.125245
77	1	0	-3.695853	4.752190	3.801633
78	1	0	-4.150962	3.631915	5.096282
79	1	0	-2.595996	3.449103	4.259298
80	6	0	-5.765736	3.241452	2.841274
81	1	0	-6.227947	2.673305	2.026498
82	1	0	-6.368150	3.081685	3.742387
83	1	0	-5.815693	4.303511	2.599578
84	6	0	-4.412002	1.374051	3.739764
85	1	0	-4.948202	0.664310	3.099848
86	1	0	-3.425509	0.959821	3.968571
87	1	0	-4.966858	1.448223	4.680120
88	8	0	-3.775863	5.086969	1.471924
89	8	0	-6.349675	-2.748859	-2.838638
90	8	0	2.811485	-5.412986	1.480688
91	8	0	6.540531	3.040578	-2.481946
92	6	0	7.048771	4.001535	-1.559835
93	1	0	7.987087	4.371024	-1.977702
94	1	0	6.361445	4.842261	-1.430544
95	1	0	7.236155	3.550033	-0.580341
96	6	0	3.783940	4.260180	-2.824776
97	6	0	4.805270	4.742472	-3.871274
98	1	0	4.359505	5.573231	-4.428690
99	1	0	5.733875	5.109321	-3.434885
100	1	0	5.051725	3.953189	-4.587825
101	6	0	3.559388	5.363286	-1.773369
102	1	0	3.200221	6.278992	-2.256408
103	1	0	2.813081	5.052638	-1.036194
104	1	0	4.479148	5.607115	-1.233860
105	6	0	-4.792658	5.448533	0.540153
106	1	0	-5.271285	6.347112	0.933247
107	1	0	-4.376244	5.662245	-0.449140
108	1	0	-5.537500	4.651430	0.439389
109	6	0	-6.633317	-3.968826	-2.157337
110	1	0	-7.688088	-4.190164	-2.332534
111	1	0	-6.034191	-4.796959	-2.546025
112	1	0	-6.454212	-3.874293	-1.081885
113	6	0	-6.858418	-0.403029	-1.228492
114	6	0	-6.776521	0.818823	-0.293682
115	1	0	-6.384499	0.553677	0.694617
116	1	0	-6.157900	1.622454	-0.707077
117	1	0	-7.783185	1.223451	-0.149842
118	6	0	-7.474996	0.073416	-2.557889
119	1	0	-7.585078	-0.749403	-3.267549
120	1	0	-8.466280	0.504862	-2.378134
121	1	0	-6.850471	0.846108	-3.019545
122	6	0	-7.799969	-1.417140	-0.552217
123	1	0	-8.119146	-2.203547	-1.235201
124	1	0	-7.327661	-1.880218	0.320764
125	1	0	-8.701693	-0.899711	-0.206971
126	6	0	-3.612703	-3.519273	-3.895945
127	6	0	-3.255258	-4.841408	-3.188994
128	1	0	-2.414149	-4.698153	-2.501991
129	1	0	-4.091352	-5.242857	-2.611275
130	1	0	-2.963276	-5.595573	-3.928480
131	6	0	-4.733642	-3.749471	-4.926669
132	1	0	-4.347502	-4.389123	-5.727167
133	1	0	-5.611906	-4.242978	-4.513905
134	1	0	-5.056201	-2.806135	-5.379123
135	6	0	-2.373106	-3.083814	-4.711653
136	1	0	-2.221599	-3.790352	-5.533593

137	1	0	-2.505019	-2.085887	-5.143827
138	1	0	-1.449548	-3.087939	-4.125685
139	6	0	6.986247	0.301603	-1.683890
140	6	0	2.456745	4.056386	-3.594464
141	1	0	2.532006	3.226000	-4.305260
142	1	0	1.602682	3.872045	-2.937518
143	1	0	2.225919	4.964214	-4.161087
144	6	0	7.275499	0.167769	-3.192103
145	1	0	8.210509	-0.383169	-3.344087
146	1	0	6.472714	-0.382807	-3.695032
147	1	0	7.376234	1.144180	-3.671018
148	6	0	8.171371	1.000643	-0.991047
149	1	0	7.940043	1.237686	0.052954
150	1	0	9.038196	0.330551	-0.993916
151	1	0	8.467226	1.915904	-1.502749
152	6	0	6.906874	-1.118791	-1.100092
153	1	0	6.111041	-1.716695	-1.556310
154	1	0	7.853587	-1.634389	-1.289097
155	1	0	6.752952	-1.102043	-0.016613
156	6	0	1.974208	-5.684796	2.601906
157	1	0	2.121397	-6.737138	2.851630
158	1	0	2.240193	-5.065243	3.464059
159	1	0	0.921476	-5.509895	2.359928
160	6	0	4.522189	-3.286613	2.801641
161	6	0	4.010343	-2.802827	4.169549
162	1	0	4.817135	-2.846420	4.909784
163	1	0	3.647725	-1.771032	4.126554
164	1	0	3.194529	-3.436306	4.529704
165	6	0	5.062771	-4.719927	2.948591
166	1	0	5.943315	-4.687738	3.599756
167	1	0	4.351899	-5.407633	3.406955
168	1	0	5.375365	-5.136765	1.987234
169	6	0	5.741142	-2.428092	2.391113
170	1	0	6.132023	-2.754844	1.421789
171	1	0	5.514958	-1.360755	2.327187
172	1	0	6.538202	-2.545458	3.132938
173	6	0	1.383530	-4.978944	-1.011671
174	6	0	0.587885	-4.394717	-2.193584
175	1	0	0.240973	-5.215360	-2.829205
176	1	0	-0.295921	-3.840712	-1.856358
177	1	0	1.194888	-3.733660	-2.821247
178	6	0	0.441835	-5.951286	-0.277929
179	1	0	-0.338492	-5.412135	0.268434
180	1	0	-0.051476	-6.600267	-1.010001
181	1	0	0.979334	-6.594613	0.418966
182	6	0	2.577821	-5.762517	-1.590172
183	1	0	3.229815	-5.106164	-2.177021
184	1	0	3.173726	-6.222228	-0.797984
185	1	0	2.218707	-6.558612	-2.252361
186	1	0	-1.023651	0.442306	-2.920804

2b

RwB97XD SCF energy -2576.351388 a.u.
RwB97XD SCF enthalpy -2575.734158a.u.
RwB97XD SCF free energy -2575.843968 a.u.
Three lowest frequencies (cm^{-1}) 10.9, 29.4, 38.2

Cartesian coordinates:

Center Number	Atomic Number	Atomic Type	Coordinates (Angstroms) X	Y	Z
1	6	0	0.327403	1.542941	0.169377
2	6	0	0.027959	2.855070	-0.123537
3	6	0	0.616663	3.556564	-1.170765
4	6	0	1.573500	2.985701	-1.973358
5	6	0	1.922901	1.659117	-1.682855
6	6	0	1.331614	0.940690	-0.644295
7	1	0	2.037488	3.527880	-2.788491
8	1	0	2.683734	1.193053	-2.297837
9	6	0	-0.405324	0.883613	1.287197
10	6	0	-1.422247	-0.096184	1.119820
11	6	0	-0.135777	1.258079	2.584752
12	6	0	-2.053705	-0.657759	2.225909
13	6	0	-0.756850	0.675235	3.684170
14	6	0	-1.724089	-0.288863	3.540498
15	1	0	-2.825093	-1.406935	2.084092
16	1	0	-2.211308	-0.741661	4.395977
17	8	0	0.096620	4.806962	-1.232423
18	8	0	-0.876337	3.648897	0.507780
19	8	0	0.774554	2.176059	3.003721
20	8	0	-0.238674	1.198866	4.828132
21	6	0	-0.883394	4.895475	-0.192898
22	1	0	-1.868801	5.060048	-0.636918
23	1	0	-0.611571	5.698732	0.495898
24	6	0	0.625549	2.263750	4.423187
25	1	0	0.165922	3.224167	4.679169
26	1	0	1.599240	2.142088	4.900852
27	15	0	-1.744983	-0.726942	-0.568088
28	15	0	1.784307	-0.817745	-0.425524
29	46	0	0.130678	-2.118399	-1.157258
30	6	0	-2.173415	0.747518	-1.544735
31	6	0	-3.008461	1.748486	-1.035129
32	6	0	-1.593937	0.903282	-2.807537
33	6	0	-3.269764	2.886639	-1.790448
34	1	0	-3.438575	1.649553	-0.042541
35	6	0	-1.842897	2.053046	-3.552471
36	1	0	-0.928126	0.136703	-3.196023
37	6	0	-2.679740	3.043967	-3.044123
38	6	0	-3.242819	-1.759707	-0.430662
39	6	0	-4.542382	-1.269719	-0.589970
40	6	0	-3.049194	-3.118412	-0.146223
41	6	0	-5.631006	-2.127467	-0.458307
42	6	0	-4.140371	-3.971121	-0.008060
43	1	0	-2.040806	-3.512276	-0.024535
44	6	0	-5.432567	-3.474816	-0.164655
45	6	0	3.368608	-1.022912	-1.308597
46	6	0	4.600680	-0.810580	-0.685204
47	6	0	3.329832	-1.365486	-2.665849
48	6	0	5.779923	-0.941560	-1.414864
49	1	0	4.655848	-0.557437	0.367561
50	6	0	4.509247	-1.478766	-3.393261
51	1	0	2.374450	-1.542925	-3.152806
52	6	0	5.736599	-1.269608	-2.766982
53	6	0	2.071820	-1.033454	1.356769
54	6	0	1.505644	-2.123669	2.024337
55	6	0	2.796020	-0.075470	2.076408
56	6	0	1.662577	-2.254194	3.400761
57	1	0	0.924803	-2.855611	1.469443
58	6	0	2.963582	-0.220619	3.449260
59	1	0	3.206540	0.796341	1.575787
60	6	0	2.391074	-1.303925	4.112849
61	1	0	1.384411	-3.032972	-1.323759
62	1	0	4.468769	-1.738343	-4.446403
63	1	0	6.659155	-1.369349	-3.330446
64	1	0	6.733942	-0.787315	-0.920720
65	1	0	1.208798	-3.094720	3.915936
66	1	0	2.510123	-1.405435	5.187019
67	1	0	3.527377	0.523671	4.002530
68	1	0	-3.924462	3.657216	-1.395261
69	1	0	-2.870577	3.942945	-3.622229
70	1	0	-1.376380	2.177814	-4.524655
71	1	0	-4.713498	-0.223492	-0.820568
72	1	0	-6.636878	-1.739397	-0.585144
73	1	0	-6.284653	-4.139865	-0.062836
74	1	0	-3.979741	-5.021353	0.214228

1a

RwB97XD SCF energy -728.280036 a.u.
RwB97XD SCF enthalpy -728.025798 a.u.
RwB97XD SCF free energy -728.085088 a.u.
Three lowest frequencies (cm^{-1}) 30.3, 40.0, 63.3

Cartesian coordinates:

Center Number	Atomic Number	Atomic Type	Coordinates (Angstroms) X	Y	Z

1	6	0	2.564033	0.055597	-0.018582
2	6	0	2.781491	-1.285722	-0.176859
3	8	0	3.752479	0.699605	0.163847
4	6	0	4.187393	-1.475957	-0.086895
5	1	0	2.026228	-2.043077	-0.335986
6	6	0	4.718023	-0.237979	0.117521
7	1	0	4.731460	-2.405546	-0.164473
8	1	0	5.729520	0.117968	0.245440
9	6	0	1.359583	0.879079	-0.006912
10	8	0	1.394554	2.083460	0.178493
11	6	0	0.061629	0.122874	-0.244651
12	1	0	0.117136	-0.371597	-1.218903
13	1	0	-0.039005	-0.636813	0.533824
14	8	0	-0.988904	1.070142	-0.224956
15	6	0	-2.312347	0.811826	-0.167899
16	8	0	-3.027149	1.786088	-0.282968
17	6	0	-2.879112	-0.593320	0.087497
18	6	0	-4.400866	-0.511132	-0.102769
19	1	0	-4.841730	-1.487581	0.119081
20	1	0	-4.850402	0.228339	0.563666
21	1	0	-4.658214	-0.247794	-1.132965
22	6	0	-2.589286	-0.982035	1.550856
23	1	0	-1.522123	-1.075445	1.766314
24	1	0	-3.011303	-0.244596	2.240750
25	1	0	-3.055000	-1.950232	1.759274
26	6	0	-2.330230	-1.649866	-0.890458
27	1	0	-2.326360	-1.278320	-1.920230
28	1	0	-1.325825	-1.993389	-0.640076
29	1	0	-2.984077	-2.526467	-0.856131

1b

RwB97XD SCF energy -802.054407 a.u.
RwB97XD SCF enthalpy -801.832638 a.u.
RwB97XD SCF free energy -801.891375 a.u.
Three lowest frequencies (cm^{-1}) 22.4, 26.5, 38.7

Cartesian coordinates:

Center Number	Atomic Number	Atomic Type	Coordinates (Angstroms) X	Y	Z
1	8	0	3.383081	-1.659951	0.328526
2	6	0	2.441307	-1.044543	-0.152362
3	6	0	2.360304	0.404358	-0.002172
4	6	0	3.154426	1.254939	0.717854
5	8	0	1.380019	1.114108	-0.636071
6	6	0	2.630960	2.559422	0.516282
7	1	0	4.005769	0.968287	1.318061
8	6	0	1.559799	2.408143	-0.312274
9	1	0	2.997138	3.488058	0.928213
10	1	0	0.855522	3.105007	-0.741709
11	6	0	1.410727	-1.812927	-0.968693
12	1	0	1.380552	-2.835277	-0.585960
13	1	0	1.740153	-1.827082	-2.010570
14	8	0	0.104589	-1.245211	-0.998135
15	6	0	-0.508907	-1.129707	0.188391
16	8	0	-0.004876	-1.523580	1.223549
17	6	0	-1.832385	-0.467388	0.090646
18	6	0	-2.628489	-0.411988	1.237909
19	6	0	-2.277460	0.111658	-1.101666
20	6	0	-3.868404	0.212801	1.191242
21	1	0	-2.269412	-0.860394	2.158241
22	6	0	-3.517007	0.739623	-1.141213
23	1	0	-1.655206	0.076506	-1.988605
24	6	0	-4.312501	0.788697	0.002094
25	1	0	-4.488004	0.252145	2.081284
26	1	0	-3.862704	1.192362	-2.064899
27	1	0	-5.280546	1.278934	-0.033239

1c

RwB97XD SCF energy -610.375418 a.u.
RwB97XD SCF enthalpy -610.210715 a.u.
RwB97XD SCF free energy -610.260952 a.u.
Three lowest frequencies (cm^{-1}) 31.9, 55.7, 83.1

Cartesian coordinates:

Center Number	Atomic Number	Atomic Type	Coordinates (Angstroms) X	Y	Z
1	8	0	-0.605242	2.539370	0.309794
2	6	0	-0.281262	1.455062	-0.155335
3	6	0	-1.194395	0.320493	-0.066629
4	6	0	-2.399815	0.213123	0.571197
5	8	0	-0.891406	-0.860027	-0.682819
6	6	0	-2.861897	-1.109365	0.335809
7	1	0	-2.885764	0.993783	1.138377
8	6	0	-1.906678	-1.708015	-0.429296
9	1	0	-3.778460	-1.559499	0.687221
10	1	0	-1.813859	-2.695764	-0.855614
11	6	0	1.042750	1.346049	-0.899628
12	1	0	1.709358	2.114552	-0.501643
13	1	0	0.855077	1.547057	-1.957590
14	8	0	1.674339	0.068373	-0.869814
15	6	0	1.981575	-0.408312	0.348955
16	8	0	1.716880	0.196095	1.368181
17	6	0	2.663086	-1.736872	0.261810
18	1	0	3.625526	-1.620108	-0.244410
19	1	0	2.817847	-2.140583	1.261502
20	1	0	2.053502	-2.422906	-0.331870

6a

RwB97XD SCF energy -729.506698 a.u.
RwB97XD SCF enthalpy -729.228585 a.u.
RwB97XD SCF free energy -729.291024 a.u.
Three lowest frequencies (cm^{-1}) 18.2, 30.3, 35.5
Cartesian coordinates:

Center Number	Atomic Number	Atomic Type	Coordinates (Angstroms) X	Y	Z
1	6	0	-2.712054	-0.039891	0.087248
2	6	0	-3.248374	1.154515	-0.276613
3	8	0	-3.700869	-0.886942	0.492694
4	6	0	-4.663561	1.039994	-0.079997
5	1	0	-2.706274	2.016205	-0.640129
6	6	0	-4.877541	-0.216229	0.382181
7	1	0	-5.414929	1.795378	-0.259491
8	1	0	-5.762724	-0.762782	0.670320
9	6	0	-1.330549	-0.615439	0.086225
10	8	0	-1.128001	-1.504241	-1.005964
11	6	0	-0.299166	0.488269	-0.027840
12	1	0	-0.369523	0.985075	-0.999918
13	1	0	-0.434714	1.227432	0.765519
14	8	0	0.984152	-0.131908	0.100453
15	6	0	2.051915	0.663528	-0.027543
16	8	0	1.954304	1.859398	-0.222084
17	6	0	3.356457	-0.114673	0.098968
18	6	0	4.533263	0.847542	-0.070363
19	1	0	5.473085	0.293461	0.015050
20	1	0	4.511624	1.334580	-1.049701
21	1	0	4.524907	1.626308	0.697898
22	6	0	3.391060	-1.195057	-0.995861
23	1	0	2.564197	-1.901729	-0.886463
24	1	0	3.332172	-0.747456	-1.993555
25	1	0	4.331102	-1.751748	-0.928025
26	6	0	3.404832	-0.772200	1.489160
27	1	0	3.340833	-0.021375	2.283772
28	1	0	2.588795	-1.487281	1.622675
29	1	0	4.352135	-1.308430	1.604308
30	1	0	-1.672406	-2.284880	-0.852041
31	1	0	-1.172660	-1.145362	1.035617

6b

RwB97XD SCF energy -803.265890 a.u.
RwB97XD SCF enthalpy -803.018917 a.u.

RwB97XD SCF free energy	-803.078129 a.u.
Three lowest frequencies (cm^{-1})	18.7, 35.9, 49.2

Cartesian coordinates:

Center Number	Atomic Number	Atomic Type	Coordinates X	Y	(Angstroms) Z
1	8	0	3.595638	-1.674800	-0.562402
2	6	0	2.337872	-1.027377	-0.693856
3	6	0	2.347796	0.353597	-0.121269
4	6	0	1.979635	0.886870	1.074867
5	8	0	2.895812	1.322368	-0.909337
6	6	0	2.319538	2.277580	1.020352
7	1	0	1.508921	0.360002	1.892576
8	6	0	2.871050	2.480585	-0.201596
9	1	0	2.168780	3.021489	1.789279
10	1	0	3.273268	3.349396	-0.700425
11	6	0	1.254991	-1.905852	-0.070361
12	1	0	1.385917	-1.991029	1.010043
13	1	0	1.298647	-2.899842	-0.518831
14	8	0	-0.029201	-1.352904	-0.376199
15	6	0	-0.845506	-1.012555	0.627888
16	8	0	-0.597485	-1.217575	1.801280
17	1	0	2.155090	-0.953429	-1.770299
18	1	0	3.832432	-1.667191	0.373740
19	6	0	-2.091318	-0.359823	0.144660
20	6	0	-3.043782	0.024805	1.092227
21	6	0	-2.319996	-0.119697	-1.213575
22	6	0	-4.218595	0.644446	0.684281
23	1	0	-2.856655	-0.163920	2.143974
24	6	0	-3.497264	0.500104	-1.616787
25	1	0	-1.580641	-0.415460	-1.948753
26	6	0	-4.445913	0.881940	-0.670098
27	1	0	-4.956604	0.943247	1.421535
28	1	0	-3.674480	0.686032	-2.671200
29	1	0	-5.363836	1.366273	-0.988786

6c

RwB97XD SCF energy	-611.586578 a.u.
RwB97XD SCF enthalpy	-611.397074 a.u.
RwB97XD SCF free energy	-611.448738 a.u.
Three lowest frequencies (cm^{-1})	31.8, 56.1, 73.5

Cartesian coordinates:

Center Number	Atomic Number	Atomic Type	Coordinates X	Y	(Angstroms) Z
1	8	0	-1.329003	2.496792	0.255751
2	6	0	-0.604361	1.432611	-0.345070
3	6	0	-1.212480	0.097192	-0.058438
4	6	0	-0.928969	-0.900369	0.821761
5	8	0	-2.330534	-0.219660	-0.771508
6	6	0	-1.937671	-1.901215	0.638395
7	1	0	-0.097896	-0.926005	1.512654
8	6	0	-2.757100	-1.432841	-0.334880
9	1	0	-2.033779	-2.841862	1.161213
10	1	0	-3.642450	-1.823911	-0.813050
11	6	0	0.854251	1.504071	0.100337
12	1	0	0.948177	1.320856	1.172892
13	1	0	1.253723	2.493494	-0.130430
14	8	0	1.612421	0.538362	-0.636154
15	6	0	2.371481	-0.337167	0.040806
16	8	0	2.523233	-0.308828	1.245422
17	1	0	-0.656817	1.614102	-1.423030
18	1	0	-1.371201	2.323157	1.204842
19	6	0	2.965868	-1.357071	-0.880415
20	1	0	3.341311	-0.885826	-1.790765
21	1	0	3.765044	-1.895770	-0.372130
22	1	0	2.178842	-2.062837	-1.165267

2.2. Catalytic cycle **2a – 1a(*S*)**.

1a•2a (*S*)

RwB97XD SCF energy	-5020.436758 a.u.
RwB97XD SCF enthalpy	-5018.468193 a.u.
RwB97XD SCF free energy	-5018.711040 a.u.
Three lowest frequencies (cm^{-1})	16.1, 17.4, 20.8

Cartesian coordinates:

Center Number	Atomic Number	Atomic Type	Coordinates X	Y	(Angstroms) Z
1	6	0	-0.922493	-0.256276	2.606677
2	6	0	-0.822526	-0.988658	3.771887
3	6	0	-1.415213	-2.234278	3.947811
4	6	0	-2.116850	-2.847079	2.939713
5	6	0	-2.218641	-2.138534	1.735190
6	6	0	-1.658670	-0.872819	1.555734
7	1	0	-2.581797	-3.817131	3.068570
8	1	0	-2.772770	-2.599478	0.926170
9	6	0	-0.314976	1.105984	2.602700
10	6	0	0.797913	1.535690	1.827538
11	6	0	-0.804829	2.023209	3.508691
12	6	0	1.305715	2.823641	1.981467
13	6	0	-0.287795	3.305228	3.655328
14	6	0	0.766924	3.744561	2.894429
15	1	0	2.165464	3.135701	1.400939
16	1	0	1.182072	4.739381	3.005868
17	8	0	-1.170405	-2.681337	5.207839
18	8	0	-0.154633	-0.645496	4.910746
19	8	0	-1.835919	1.850386	4.384746
20	8	0	-0.987759	3.963581	4.626315
21	6	0	-0.610497	-1.563833	5.902566
22	1	0	0.228893	-1.895997	6.511924
23	1	0	-1.391502	-1.088149	6.507680
24	6	0	-1.720973	2.939073	5.300340
25	1	0	-1.159351	2.609678	6.183477
26	1	0	-2.713015	3.305746	5.560538
27	15	0	1.494410	0.361648	0.600992
28	15	0	-1.833771	-0.085819	-0.087268
29	46	0	0.045707	0.083703	-1.286482
30	6	0	1.845755	-1.111043	1.602099
31	6	0	2.596654	-1.016151	2.765052
32	6	0	1.392151	-2.354195	1.174214
33	6	0	2.913911	-2.138708	3.534789
34	1	0	2.963087	-0.041588	3.070634
35	6	0	1.618272	-3.506857	1.921308
36	1	0	0.847096	-2.403422	0.241121
37	6	0	2.328162	-3.360294	3.138726
38	6	0	3.116228	1.004463	0.079411
39	6	0	4.268645	0.230098	0.200264
40	6	0	3.171723	2.180920	-0.658521
41	6	0	5.491643	0.657821	-0.308153
42	1	0	4.200080	-0.734132	0.683264
43	6	0	4.372761	2.685230	-1.169951
44	1	0	2.248101	2.718889	-0.849908
45	6	0	5.539618	1.950018	-0.882794
46	6	0	-3.182545	-1.013687	-0.876441
47	6	0	-4.518113	-0.752303	-0.578515
48	6	0	-2.869335	-2.018710	-1.777896
49	6	0	-5.543252	-1.534153	-1.101206
50	1	0	-4.758608	0.073424	0.075897
51	6	0	-3.850518	-2.824129	-2.364749
52	1	0	-1.827189	-2.175340	-2.034325
53	6	0	-5.173747	-2.630329	-1.920952
54	6	0	-2.453077	1.595792	0.215178
55	6	0	-1.951585	2.633555	-0.555824
56	6	0	-3.386153	1.876060	1.214611
57	6	0	-2.375700	3.955490	-0.397653
58	1	0	-1.190711	2.406583	-1.294068
59	6	0	-3.921129	3.153193	1.362227
60	1	0	-3.698977	1.078453	1.877502

61	6	0	-3.452367	4.165526	0.484982
62	6	0	-1.591815	5.033745	-1.182304
63	6	0	-1.997887	5.001278	-2.666293
64	1	0	-1.381528	5.700131	-3.243040
65	1	0	-1.858220	4.001406	-3.088690
66	1	0	-3.046270	5.277826	-2.810651
67	6	0	-1.747184	6.459660	-0.625743
68	1	0	-1.541981	6.492539	0.448904
69	1	0	-1.019742	7.108839	-1.125113
70	1	0	-2.734560	6.887795	-0.796584
71	6	0	-0.079585	4.709288	-1.091194
72	1	0	0.251557	4.668751	-0.047633
73	1	0	0.184272	3.761575	-1.568412
74	1	0	0.489831	5.494033	-1.600248
75	6	0	-4.937056	3.442766	2.487073
76	6	0	-4.413446	4.595999	3.365787
77	1	0	-4.402536	5.542031	2.822514
78	1	0	-5.050379	4.714583	4.249259
79	1	0	-3.392600	4.392356	3.702747
80	6	0	-6.325772	3.801163	1.925616
81	1	0	-6.676648	3.038184	1.221474
82	1	0	-7.050125	3.852840	2.746366
83	1	0	-6.333412	4.768232	1.422656
84	6	0	-5.126678	2.216083	3.395394
85	1	0	-5.576994	1.372484	2.860354
86	1	0	-4.185389	1.882733	3.841959
87	1	0	-5.807464	2.481797	4.210099
88	8	0	-4.071673	5.390096	0.546193
89	8	0	-6.164009	-3.506443	-2.295568
90	8	0	2.466830	-4.473582	3.933358
91	8	0	6.777398	2.473437	-1.176850
92	6	0	7.230823	3.394772	-0.188444
93	1	0	8.227795	3.718120	-0.493987
94	1	0	6.572987	4.267613	-0.117614
95	1	0	7.286716	2.921331	0.797822
96	6	0	4.274972	3.964929	-2.033667
97	6	0	5.543504	4.346897	-2.815721
98	1	0	5.285526	5.160444	-3.502655
99	1	0	6.352847	4.707929	-2.180809
100	1	0	5.917882	3.511938	-3.414059
101	6	0	3.857722	5.148755	-1.142170
102	1	0	3.746823	6.057111	-1.745107
103	1	0	2.901488	4.958918	-0.643040
104	1	0	4.606838	5.347578	-0.368378
105	6	0	-4.978297	5.636841	-0.525471
106	1	0	-5.572477	6.506806	-0.238622
107	1	0	-4.454614	5.859532	-1.459874
108	1	0	-5.641125	4.780749	-0.689746
109	6	0	-6.156522	-4.713915	-1.536601
110	1	0	-7.015625	-5.298503	-1.870680
111	1	0	-5.240992	-5.290220	-1.701332
112	1	0	-6.249099	-4.508098	-0.464985
113	6	0	-7.014041	-1.146651	-0.839329
114	6	0	-7.109227	0.152285	-0.018115
115	1	0	-6.687917	0.037643	0.986975
116	1	0	-6.607708	0.992031	-0.510909
117	1	0	-8.163457	0.421735	0.099112
118	6	0	-7.710769	-0.888164	-2.189745
119	1	0	-7.696287	-1.774790	-2.827300
120	1	0	-8.756681	-0.607581	-2.021826
121	1	0	-7.223042	-0.067660	-2.727471
122	6	0	-7.777940	-2.233168	-0.059519
123	1	0	-7.970331	-3.117267	-0.667835
124	1	0	-7.229625	-2.537222	0.838677
125	1	0	-8.748051	-1.837744	0.261463
126	6	0	-3.365771	-3.798055	-3.464534
127	6	0	-2.526086	-4.918901	-2.823984
128	1	0	-1.643782	-4.514560	-2.315711
129	1	0	-3.105774	-5.488520	-2.090620
130	1	0	-2.183083	-5.619286	-3.593855
131	6	0	-4.476108	-4.422901	-4.326795
132	1	0	-4.004745	-4.937007	-5.171514
133	1	0	-5.073836	-5.163333	-3.794681
134	1	0	-5.150027	-3.663541	-4.732896
135	6	0	-2.468962	-3.002787	-4.444385
136	1	0	-2.142180	-3.659632	-5.257168
137	1	0	-3.020475	-2.165879	-4.886498
138	1	0	-1.567248	-2.604372	-3.972026
139	6	0	6.699950	-0.301949	-0.322645
140	6	0	3.182903	3.733419	-3.102064
141	1	0	3.458269	2.901218	-3.759464
142	1	0	2.206607	3.518576	-2.664865
143	1	0	3.074214	4.631296	-3.719673
144	6	0	7.212298	-0.439913	-1.770458
145	1	0	8.044677	-1.151928	-1.802830
146	1	0	6.421037	-0.819901	-2.426795
147	1	0	7.564214	0.512596	-2.170885
148	6	0	7.843704	0.175666	0.589744
149	1	0	7.482191	0.378888	1.603664
150	1	0	8.609657	-0.605007	0.657584
151	1	0	8.325631	1.074355	0.201998
152	6	0	6.292304	-1.711558	0.140873
153	1	0	5.479486	-2.124026	-0.466733
154	1	0	7.152607	-2.381113	0.043089
155	1	0	5.981373	-1.733176	1.190283
156	6	0	1.562575	-4.493068	5.034151
157	1	0	1.754834	-5.417282	5.582785
158	1	0	1.719786	-3.639547	5.700939
159	1	0	0.523838	-4.480488	4.693028
160	6	0	3.909580	-1.915610	4.700564
161	6	0	3.219761	-1.130131	5.830115
162	1	0	3.945926	-0.884534	6.613153
163	1	0	2.787135	-0.193972	5.462735
164	1	0	2.418930	-1.715178	6.291239
165	6	0	4.539936	-3.192691	5.284602
166	1	0	5.345050	-2.895456	5.965642
167	1	0	3.841704	-3.798333	5.862168
168	1	0	4.978668	-3.820867	4.504242
169	6	0	5.097097	-1.074714	4.174431
170	1	0	5.612951	-1.594958	3.360361
171	1	0	4.802511	-0.086889	3.811834
172	1	0	5.819587	-0.918807	4.982479
173	6	0	1.215400	-4.882627	1.346690
174	6	0	0.455763	-4.731931	0.015854
175	1	0	0.209465	-5.728183	-0.365203
176	1	0	-0.485075	-4.183883	0.144304
177	1	0	1.051614	-4.228213	-0.750484
178	6	0	0.311551	-5.703887	2.284057
179	1	0	-0.551376	-5.118713	2.617459
180	1	0	-0.068108	-6.578739	1.744781
181	1	0	0.847809	-6.067994	3.159937
182	6	0	2.508174	-5.672821	1.060874
183	1	0	3.144681	-5.132097	0.351827
184	1	0	3.080141	-5.842674	1.976751
185	1	0	2.266053	-6.648460	0.623967
186	8	0	2.541489	-1.909634	-1.716482
187	6	0	2.676336	-1.767100	-2.923419
188	1	0	-1.007564	-0.041462	-2.434418
189	6	0	2.031598	-2.653716	-3.877502
190	6	0	2.142981	-2.809802	-5.234346
191	8	0	1.183599	-3.597572	-3.374035
192	6	0	1.317899	-3.911850	-5.580528
193	1	0	2.748080	-2.206959	-5.896686
194	6	0	0.764609	-4.343631	-4.411492
195	1	0	1.157064	-4.332893	-6.561852
196	1	0	0.081124	-5.144533	-4.173988
197	6	0	3.542535	-0.645792	-3.499233
198	1	0	4.393259	-1.056668	-4.044069
199	1	0	3.883697	-0.002660	-2.686168
200	8	0	2.805495	0.114494	-4.475400
201	6	0	1.720896	0.721369	-4.004914
202	8	0	1.503242	0.711968	-2.799799
203	6	0	0.815149	1.317257	-5.063034
204	6	0	-0.064762	2.403029	-4.436771
205	1	0	0.531321	3.247923	-4.080524
206	1	0	-0.762129	2.776245	-5.192766

207	1	0	-0.647535	2.008726	-3.600284
208	6	0	1.629336	1.889027	-6.230208
209	1	0	2.225337	1.118093	-6.724778
210	1	0	0.941091	2.311951	-6.968202
211	1	0	2.300441	2.685828	-5.894247
212	6	0	-0.071990	0.150762	-5.554424
213	1	0	-0.779273	0.534183	-6.296260
214	1	0	0.524063	-0.637207	-6.023002
215	1	0	-0.643125	-0.286104	-4.728688

3a(*S*)

RwB97XD SCF energy -5020.428980 a.u.
RwB97XD SCF enthalpy -5018.461076 a.u.
RwB97XD SCF free energy -5018.707100 a.u.
Three lowest frequencies (cm^{-1}) 13.1, 15.6, 18.4
Cartesian coordinates:

Standard orientation:

Center Number	Atomic Number	Atomic Type	Coordinates (Angstroms) X	Y	Z
1	6	0	-0.228973	-1.605325	-2.280844
2	6	0	-0.844731	-2.713902	-2.818368
3	6	0	-0.554265	-4.017390	-2.427961
4	6	0	0.406216	-4.287621	-1.485854
5	6	0	1.053119	-3.179892	-0.919731
6	6	0	0.757702	-1.866983	-1.288990
7	1	0	0.647040	-5.300222	-1.185293
8	1	0	1.801793	-3.369416	-0.159606
9	6	0	-0.629759	-0.269964	-2.805295
10	6	0	-1.403661	0.689426	-2.098672
11	6	0	-0.317169	0.048659	-4.107617
12	6	0	-1.755342	1.895144	-2.700296
13	6	0	-0.672792	1.254890	-4.700645
14	6	0	-1.383070	2.211581	-4.017422
15	1	0	-2.354745	2.614553	-2.154251
16	1	0	-1.663754	3.152980	-4.474891
17	8	0	-1.323665	-4.884706	-3.139419
18	8	0	-1.794659	-2.735409	-3.794269
19	8	0	0.376200	-0.710548	-5.002477
20	8	0	-0.199820	1.281164	-5.980782
21	6	0	-2.308748	-4.066701	-3.776589
22	1	0	-3.224910	-4.086218	-3.180972
23	1	0	-2.469580	-4.412376	-4.796982
24	6	0	0.171439	-0.069739	-6.262794
25	1	0	-0.648968	-0.568542	-6.792893
26	1	0	1.098392	-0.086047	-6.834982
27	15	0	-1.860214	0.277620	-0.376986
28	15	0	1.520261	-0.507348	-0.326840
29	46	0	-0.000900	0.263014	1.119870
30	6	0	-2.853351	-1.234782	-0.506291
31	6	0	-3.900315	-1.335143	-1.417604
32	6	0	-2.649518	-2.245873	0.421795
33	6	0	-4.766210	-2.428872	-1.423609
34	1	0	-4.053654	-0.522820	-2.119386
35	6	0	-3.450642	-3.391063	0.449643
36	1	0	-1.838774	-2.133057	1.135091
37	6	0	-4.468784	-3.484508	-0.525309
38	6	0	-3.023002	1.551293	0.209214
39	6	0	-4.287996	1.209156	0.681480
40	6	0	-2.568573	2.854867	0.379062
41	6	0	-5.149431	2.163798	1.216371
42	1	0	-4.602162	0.176175	0.633493
43	6	0	-3.385592	3.867407	0.894383
44	1	0	-1.547162	3.090216	0.096811
45	6	0	-4.713693	3.509983	1.207137
46	6	0	2.990540	-1.266348	0.435125
47	6	0	4.216757	-1.305994	-0.225401
48	6	0	2.883593	-1.830960	1.697642
49	6	0	5.307805	-1.987079	0.307232
50	1	0	4.319108	-0.796545	-1.172339
51	6	0	3.945532	-2.510356	2.301884
52	1	0	1.946508	-1.723670	2.234256
53	6	0	5.111674	-2.672471	1.530453
54	6	0	2.143812	0.727662	-1.492097
55	6	0	2.034400	2.070513	-1.165729
56	6	0	2.786622	0.353242	-2.675394
57	6	0	2.631532	3.069087	-1.945839
58	1	0	1.472946	2.344416	-0.276668
59	6	0	3.453763	1.290124	-3.457714
60	1	0	2.775675	-0.689875	-2.967609
61	6	0	3.444945	2.634970	-3.007320
62	6	0	2.306331	4.532058	-1.571843
63	6	0	2.920588	4.857830	-0.199027
64	1	0	2.687660	5.890188	0.085509
65	1	0	2.525390	4.202013	0.582352
66	1	0	4.010028	4.749428	-0.216772
67	6	0	2.747601	5.596362	-2.590604
68	1	0	2.399918	5.361280	-3.600146
69	1	0	2.299234	6.551920	-2.297069
70	1	0	3.827342	5.744822	-2.623136
71	6	0	0.769783	4.659372	-1.473296
72	1	0	0.296316	4.452800	-2.439491
73	1	0	0.338294	3.980756	-0.733995
74	1	0	0.506983	5.679709	-1.176035
75	6	0	4.098766	0.879418	-4.797361
76	6	0	3.445094	1.703609	-5.924166
77	1	0	3.669976	2.767405	-5.823658
78	1	0	3.813878	1.366927	-6.899644
79	1	0	2.357217	1.587353	-5.908963
80	6	0	5.623383	1.096569	-4.816810
81	1	0	6.105086	0.627798	-3.951975
82	1	0	6.042947	0.638171	-5.719272
83	1	0	5.888456	2.153550	-4.835385
84	6	0	3.852952	-0.608132	-5.099120
85	1	0	4.349382	-1.261197	-4.372397
86	1	0	2.787958	-0.856152	-5.118797
87	1	0	4.269015	-0.842328	-6.083969
88	8	0	4.259534	3.528900	-3.657999
89	8	0	6.111536	-3.507494	1.966576
90	8	0	-5.214165	-4.633690	-0.580527
91	8	0	-5.631527	4.477643	1.542706
92	6	0	-6.187625	5.135017	0.407403
93	1	0	-6.946630	5.823655	0.783256
94	1	0	-5.430166	5.701333	-0.143544
95	1	0	-6.650493	4.418164	-0.279205
96	6	0	-2.735193	5.259277	1.085709
97	6	0	-3.526042	6.241106	1.968712
98	1	0	-2.886896	7.106946	2.174317
99	1	0	-4.433184	6.617323	1.495835
100	1	0	-3.796822	5.792000	2.928465
101	6	0	-2.497453	5.898111	-0.295157
102	1	0	-1.986517	6.861135	-0.184078
103	1	0	-1.878507	5.256373	-0.929760
104	1	0	-3.440829	6.076996	-0.820834
105	6	0	5.457458	3.808659	-2.936150
106	1	0	6.057960	4.466340	-3.567215
107	1	0	5.250609	4.309366	-1.984739
108	1	0	6.016891	2.889240	-2.731497
109	6	0	5.819625	-4.881457	1.719619
110	1	0	6.666406	-5.454799	2.100703
111	1	0	4.904512	-5.197903	2.231693
112	1	0	5.700321	-5.072274	0.647504
113	6	0	6.684677	-1.900043	-0.383058
114	6	0	6.629938	-0.986922	-1.621102
115	1	0	5.970045	-1.385753	-2.399619
116	1	0	6.299252	0.027178	-1.372056
117	1	0	7.633182	-0.908129	-2.051379
118	6	0	7.691610	-1.278877	0.605147
119	1	0	7.802546	-1.889186	1.504032
120	1	0	8.675116	-1.188377	0.130343
121	1	0	7.371309	-0.275496	0.907479
122	6	0	7.201181	-3.272659	-0.853462
123	1	0	7.473808	-3.917008	-0.016970
124	1	0	6.455650	-3.788883	-1.467623
125	1	0	8.099184	-3.132196	-1.465111
126	6	0	3.739456	-2.921901	3.778422
127	6	0	2.604406	-3.956214	3.882352
128	1	0	1.669787	-3.581766	3.451282
129	1	0	2.866948	-4.884646	3.363406
130	1	0	2.413648	-4.197229	4.934225
131	6	0	4.976786	-3.470619	4.508171
132	1	0	4.724817	-3.579135	5.568907
133	1	0	5.292379	-4.452245	4.153371
134	1	0	5.825823	-2.786113	4.436309
135	6	0	3.339716	-1.641706	4.546130
136	1	0	3.167224	-1.876594	5.601343
137	1	0	4.143564	-0.900421	4.490728
138	1	0	2.427956	-1.180112	4.157601
139	6	0	-6.480008	1.724251	1.864369
140	6	0	-1.373403	5.073434	1.794340
141	1	0	-1.514049	4.634189	2.787700
142	1	0	-0.676548	4.444819	1.236753
143	1	0	-0.890704	6.047828	1.923397
144	6	0	-6.503899	2.205149	3.329571
145	1	0	-7.432936	1.880929	3.811926
146	1	0	-5.666282	1.777069	3.891494
147	1	0	-6.446366	3.293328	3.399362
148	6	0	-7.710408	2.273519	1.119351
149	1	0	-7.651471	2.064209	0.045828
150	1	0	-8.615763	1.790950	1.503860
151	1	0	-7.829078	3.348295	1.261069
152	6	0	-6.610061	0.190844	1.890039
153	1	0	-5.775025	-0.288927	2.411809
154	1	0	-7.527957	-0.077211	2.422829
155	1	0	-6.680865	-0.240070	0.885440
156	6	0	-4.708407	-5.616041	-1.472856
157	1	0	-5.247909	-6.542408	-1.264320
158	1	0	-4.885238	-5.336797	-2.518273
159	1	0	-3.634681	-5.779648	-1.321633
160	6	0	-5.981313	-2.362444	-2.384685

161	6	0	-5.532227	-2.661872	-3.826803
162	1	0	-6.350279	-2.448542	-4.524224
163	1	0	-4.673237	-2.046727	-4.116149
164	1	0	-5.259455	-3.713889	-3.953981
165	6	0	-7.146704	-3.299224	-2.019173
166	1	0	-8.005699	-3.044182	-2.649753
167	1	0	-6.920222	-4.351835	-2.186819
168	1	0	-7.450625	-3.175274	-0.975204
169	6	0	-6.564251	-0.929626	-2.349884
170	1	0	-6.810666	-0.627265	-1.326599
171	1	0	-5.890725	-0.179902	-2.772497
172	1	0	-7.485153	-0.897798	-2.940915
173	6	0	-3.128819	-4.432808	1.549434
174	6	0	-1.787736	-5.107982	1.204837
175	1	0	-1.523319	-5.840343	1.976244
176	1	0	-1.848692	-5.632948	0.245404
177	1	0	-0.975721	-4.376189	1.140002
178	6	0	-4.182995	-5.531625	1.769674
179	1	0	-4.228064	-6.248381	0.949079
180	1	0	-3.907918	-6.090834	2.671269
181	1	0	-5.182193	-5.116990	1.924837
182	6	0	-2.986605	-3.699260	2.902688
183	1	0	-2.172089	-2.971949	2.912494
184	1	0	-3.912059	-3.173975	3.163352
185	1	0	-2.779670	-4.428764	3.692912
186	8	0	-1.340842	0.614248	2.858423
187	6	0	-0.547866	0.814110	3.802302
188	1	0	1.281049	0.409330	1.999095
189	6	0	-0.248520	-0.213104	4.756084
190	6	0	0.386612	-0.201791	5.974511
191	8	0	-0.672606	-1.479023	4.468319
192	6	0	0.337636	-1.529923	6.460343
193	1	0	0.827150	0.661261	6.453477
194	6	0	-0.305423	-2.256376	5.497777
195	1	0	0.736129	-1.907574	7.389960
196	1	0	-0.561129	-3.302636	5.417028
197	6	0	0.056185	2.191277	3.980612
198	1	0	-0.357924	2.669287	4.874720
199	1	0	-0.195975	2.783197	3.101883
200	8	0	1.469993	2.063492	4.120791
201	6	0	2.224079	3.013300	3.536145
202	8	0	1.738474	3.994712	3.012035
203	6	0	3.709391	2.678302	3.579751
204	6	0	3.959988	1.605413	2.500401
205	1	0	3.666744	1.965122	1.507390
206	1	0	5.027091	1.360926	2.472885
207	1	0	3.406153	0.688365	2.714135
208	6	0	4.512780	3.941340	3.259754
209	1	0	4.325050	4.729064	3.996160
210	1	0	5.581524	3.706676	3.277411
211	1	0	4.266687	4.333545	2.269889
212	6	0	4.098758	2.139210	4.963744
213	1	0	5.167304	1.901714	4.970599
214	1	0	3.913221	2.884417	5.744484
215	1	0	3.546989	1.231088	5.217213

TS1a(*S*)

RwB97XD SCF energy -5020.425887 a.u.

RwB97XD SCF enthalpy -5018.458665 a.u.

RwB97XD SCF free energy -5018.698397 a.u.

Three lowest frequencies (cm^{-1}) -428.7, 14.1, 16.7

Imaginary frequency (cm^{-1}) -428.7

Cartesian coordinates:

Center Number	Atomic Number	Atomic Type	Coordinates (Angstroms) X	Y	Z
1	6	0	0.445557	-2.045871	1.882039
2	6	0	0.910974	-3.283987	2.273357
3	6	0	0.432370	-4.481671	1.751649
4	6	0	-0.516080	-4.506254	0.759729
5	6	0	-0.996035	-3.262397	0.326537
6	6	0	-0.556136	-2.054891	0.870645
7	1	0	-0.892521	-5.433930	0.345552
8	1	0	-1.755832	-3.260270	-0.445596
9	6	0	0.956244	-0.848073	2.603778
10	6	0	1.697673	0.226675	2.040803
11	6	0	0.732801	-0.773225	3.962564
12	6	0	2.109349	1.294667	2.835924
13	6	0	1.147839	0.297429	4.746759
14	6	0	1.831464	1.360361	4.209906
15	1	0	2.679660	2.104543	2.396889
16	1	0	2.156705	2.198903	4.814308
17	8	0	1.046516	-5.523746	2.371227
18	8	0	1.869597	-3.552332	3.204628
19	8	0	0.065770	-1.670495	4.741283
20	8	0	0.749303	0.094652	6.034033
21	6	0	1.732847	-4.945012	3.483722
22	1	0	2.716382	-5.401357	3.584441
23	1	0	1.129151	-5.075678	4.389733
24	6	0	0.338624	-1.273483	6.085811
25	1	0	1.157550	-1.883730	6.484520
26	1	0	-0.568448	-1.363623	6.682717
27	15	0	2.038897	0.219458	0.245335
28	15	0	-1.255858	-0.499544	0.202836
29	46	0	0.174069	0.674801	-1.107351
30	6	0	2.750042	-1.405148	-0.114073
31	6	0	3.791226	-1.942819	0.630197
32	6	0	2.212688	-2.129118	-1.175969
33	6	0	4.301151	-3.217697	0.360770
34	1	0	4.220623	-1.353761	1.434737
35	6	0	2.624788	-3.427384	-1.456490
36	1	0	1.431904	-1.666123	-1.766757
37	6	0	3.623337	-3.977551	-0.616882
38	6	0	3.300428	1.488357	-0.073728
39	6	0	4.576300	1.180026	-0.539840
40	6	0	2.904410	2.819705	-0.004499
41	6	0	5.493938	2.183738	-0.850982
42	1	0	4.848982	0.140995	-0.669341
43	6	0	3.770065	3.874248	-0.303176
44	1	0	1.880649	3.040738	0.277759
45	6	0	5.096918	3.524906	-0.629681
46	6	0	-2.777660	-1.041631	-0.633069
47	6	0	-3.944121	-1.274682	0.094530
48	6	0	-2.768549	-1.294799	-1.996683
49	6	0	-5.059216	-1.867840	-0.486436
50	1	0	-3.976882	-0.998075	1.138143
51	6	0	-3.861999	-1.881271	-2.647401
52	1	0	-1.887725	-1.024876	-2.571404
53	6	0	-4.944846	-2.272867	-1.838369
54	6	0	-1.801350	0.496112	1.615552
55	6	0	-1.683340	1.874686	1.532495
56	6	0	-2.391617	-0.078380	2.743985
57	6	0	-2.210398	2.723096	2.513084
58	1	0	-1.164633	2.301170	0.678232
59	6	0	-3.001935	0.709395	3.715059
60	1	0	-2.385581	-1.156929	2.843762
61	6	0	-2.978830	2.113948	3.520713
62	6	0	-1.852185	4.221571	2.397716
63	6	0	-2.518096	4.816809	1.145831
64	1	0	-2.226319	5.865381	1.020079
65	1	0	-2.219432	4.280132	0.241032
66	1	0	-3.609735	4.778716	1.218497
67	6	0	-2.202429	5.086824	3.619918
68	1	0	-1.810041	4.657578	4.545792
69	1	0	-1.737403	6.069638	3.484511
70	1	0	-3.272930	5.253034	3.741921
71	6	0	-0.318045	4.321285	2.237995
72	1	0	0.194538	3.905128	3.112183
73	1	0	0.047466	3.798409	1.350894
74	1	0	-0.029310	5.372626	2.139629
75	6	0	-3.597579	0.069768	4.985931
76	6	0	-2.866026	0.652473	6.211171
77	1	0	-3.064833	1.720471	6.324255
78	1	0	-3.197661	0.145894	7.124477
79	1	0	-1.784092	0.518915	6.117735
80	6	0	-5.112151	0.316315	5.124144
81	1	0	-5.646942	0.025643	4.213562
82	1	0	-5.503592	-0.290962	5.947778
83	1	0	-5.341237	1.358194	5.348538
84	6	0	-3.389397	-1.453511	4.989792
85	1	0	-3.932702	-1.942933	4.173287
86	1	0	-2.333250	-1.726791	4.913592
87	1	0	-3.773489	-1.863399	5.929211
88	8	0	-3.728633	2.887926	4.371300
89	8	0	-5.938408	-3.064688	-2.360331
90	8	0	3.944677	-5.300967	-0.790646
91	8	0	6.049435	4.502383	-0.788696
92	6	0	6.587910	4.980571	0.441074
93	1	0	7.392019	5.671506	0.181514
94	1	0	5.837347	5.511893	1.033995
95	1	0	6.990721	4.159724	1.043352
96	6	0	3.165154	5.299345	-0.282899
97	6	0	3.985954	6.365981	-1.029005
98	1	0	3.388587	7.282520	-1.086715
99	1	0	4.920020	6.622973	-0.530734
100	1	0	4.216524	6.053402	-2.051871
101	6	0	2.952124	5.742318	1.176055
102	1	0	2.438684	6.709963	1.206835

103	1	0	2.341784	5.018308	1.724773
104	1	0	3.901914	5.849773	1.708753
105	6	0	-4.944591	3.339643	3.778556
106	1	0	-5.465898	3.922894	4.539640
107	1	0	-4.758238	3.969697	2.903256
108	1	0	-5.570998	2.495194	3.470377
109	6	0	-5.560316	-4.439011	-2.424928
110	1	0	-6.378949	-4.967044	-2.916984
111	1	0	-4.637672	-4.575377	-2.998767
112	1	0	-5.408041	-4.853847	-1.422292
113	6	0	-6.369430	-2.002281	0.315900
114	6	0	-6.250991	-1.318696	1.690271
115	1	0	-5.518828	-1.813543	2.337900
116	1	0	-5.980790	-0.260501	1.604488
117	1	0	-7.220192	-1.371954	2.195994
118	6	0	-7.502674	-1.295571	-0.452729
119	1	0	-7.662979	-1.744365	-1.435095
120	1	0	-8.439048	-1.369768	0.111466
121	1	0	-7.277356	-0.232491	-0.591524
122	6	0	-6.752024	-3.472288	0.569406
123	1	0	-7.083489	-3.972027	-0.341427
124	1	0	-5.912268	-4.032973	0.993622
125	1	0	-7.578415	-3.515532	1.287431
126	6	0	-3.797642	-1.917943	-4.192361
127	6	0	-2.529489	-2.661718	-4.648942
128	1	0	-1.615702	-2.185855	-4.282487
129	1	0	-2.536438	-3.699945	-4.298446
130	1	0	-2.474683	-2.673112	-5.743037
131	6	0	-5.002179	-2.529852	-4.926390
132	1	0	-4.864811	-2.352614	-5.998841
133	1	0	-5.085783	-3.609242	-4.789172
134	1	0	-5.944727	-2.066694	-4.628474
135	6	0	-3.732145	-0.441608	-4.640742
136	1	0	-3.627908	-0.378786	-5.729247
137	1	0	-4.653090	0.078205	-4.356174
138	1	0	-2.889170	0.091179	-4.194014
139	6	0	6.845056	1.824575	-1.507078
140	6	0	1.789088	5.260806	-0.989823
141	1	0	1.880428	4.867114	-2.007621
142	1	0	1.052627	4.657666	-0.454097
143	1	0	1.381341	6.274896	-1.052538
144	6	0	6.923836	2.509994	-2.885662
145	1	0	7.869513	2.253879	-3.376554
146	1	0	6.106634	2.174863	-3.533995
147	1	0	6.870036	3.596961	-2.798091
148	6	0	8.055249	2.241570	-0.649114
149	1	0	7.955112	1.880464	0.380185
150	1	0	8.966722	1.801325	-1.068485
151	1	0	8.196348	3.322233	-0.629551
152	6	0	6.966635	0.309718	-1.738546
153	1	0	6.158072	-0.086309	-2.361711
154	1	0	7.909780	0.103359	-2.254018
155	1	0	6.982398	-0.242247	-0.794179
156	6	0	3.353885	-6.147370	0.192985
157	1	0	3.739208	-7.152960	0.014013
158	1	0	3.616541	-5.831968	1.207914
159	1	0	2.263830	-6.152637	0.101768
160	6	0	5.587224	-3.623970	1.123633
161	6	0	5.284281	-3.785751	2.622824
162	1	0	6.212630	-3.981388	3.171107
163	1	0	4.826203	-2.887209	3.047647
164	1	0	4.609231	-4.628010	2.799142
165	6	0	6.292716	-4.897363	0.624104
166	1	0	7.264823	-4.963843	1.124959
167	1	0	5.751789	-5.814429	0.859486
168	1	0	6.474939	-4.867510	-0.453180
169	6	0	6.616808	-2.485352	0.938495
170	1	0	6.849246	-2.350865	-0.122725
171	1	0	6.273815	-1.527540	1.338383
172	1	0	7.546224	-2.740129	1.458525
173	6	0	2.069266	-4.156584	-2.698467
174	6	0	0.978936	-3.320956	-3.394987
175	1	0	0.624112	-3.864545	-4.276195
176	1	0	0.115702	-3.150393	-2.740848
177	1	0	1.352129	-2.351129	-3.738255
178	6	0	1.440917	-5.525533	-2.379518
179	1	0	0.701440	-5.447225	-1.576115
180	1	0	0.925025	-5.900472	-3.270107
181	1	0	2.188675	-6.266819	-2.098274
182	6	0	3.227095	-4.348224	-3.697549
183	1	0	3.652944	-3.382629	-3.991501
184	1	0	4.025893	-4.959334	-3.269193
185	1	0	2.863734	-4.848360	-4.602486
186	8	0	1.131260	1.900740	-2.534656
187	6	0	-0.035206	1.993197	-3.073502
188	1	0	-1.062543	1.069236	-2.080768
189	6	0	-0.289878	1.274384	-4.330965
190	6	0	-0.993964	1.596951	-5.451591
191	8	0	0.341310	0.081738	-4.496889
192	6	0	-0.773781	0.531596	-6.376138
193	1	0	-1.589585	2.486385	-5.599816
194	6	0	0.031580	-0.358697	-5.739409
195	1	0	-1.173328	0.437415	-7.375161
196	1	0	0.456822	-1.309717	-6.022308
197	6	0	-0.824720	3.269261	-2.830047
198	1	0	-0.584930	3.991814	-3.617426
199	1	0	-0.536396	3.680454	-1.861193
200	8	0	-2.208819	2.943261	-2.850121
201	6	0	-3.064742	3.899553	-2.451493
202	8	0	-2.699252	5.032355	-2.216097
203	6	0	-4.487352	3.368247	-2.347826
204	6	0	-4.483083	2.074783	-1.513182
205	1	0	-4.032163	2.237281	-0.527093
206	1	0	-5.513026	1.736160	-1.362536
207	1	0	-3.934387	1.274652	-2.015811
208	6	0	-5.370264	4.426948	-1.685243
209	1	0	-5.367629	5.362351	-2.251874
210	1	0	-6.400201	4.060542	-1.634682
211	1	0	-5.035510	4.643792	-0.666082
212	6	0	-4.987783	3.079094	-3.775143
213	1	0	-5.991148	2.644209	-3.726214
214	1	0	-5.042719	3.999743	-4.365252
215	1	0	-4.333169	2.373837	-4.293767

4a(*S*)

RwB97XD SCF energy -5020.436903 a.u.

RwB97XD SCF enthalpy -5018.465745 a.u.

RwB97XD SCF free energy -5018.706577 a.u.

Three lowest frequencies (cm^{-1}) 4.4, 18.7, 21.9

Cartesian coordinates:

Center Number	Atomic Number	Atomic Type	Coordinates (Angstroms) X	Y	Z
1	6	0	0.524614	-2.321076	1.678155
2	6	0	1.015079	-3.582846	1.937840
3	6	0	0.557179	-4.725918	1.288065
4	6	0	-0.393867	-4.664620	0.299471
5	6	0	-0.902283	-3.391592	0.001770
6	6	0	-0.478963	-2.245917	0.673397
7	1	0	-0.751462	-5.552424	-0.208261
8	1	0	-1.666850	-3.315303	-0.763149
9	6	0	0.982620	-1.184485	2.521934
10	6	0	1.633693	0.001762	2.079422
11	6	0	0.761135	-1.270505	3.879577
12	6	0	1.976345	1.002431	2.988304
13	6	0	1.107762	-0.266006	4.777697
14	6	0	1.710975	0.895183	4.361072
15	1	0	2.482420	1.895279	2.642562
16	1	0	1.984589	1.681674	5.053956
17	8	0	1.192892	-5.816221	1.792021
18	8	0	1.973557	-3.932265	2.840027
19	8	0	0.150440	-2.284240	4.552572
20	8	0	0.734387	-0.634421	6.032387
21	6	0	1.863567	-5.349766	2.965378
22	1	0	2.855888	-5.794562	3.022058
23	1	0	1.257670	-5.590007	3.847343
24	6	0	0.403135	-2.022751	5.932815
25	1	0	1.258172	-2.622622	6.265717
26	1	0	-0.495326	-2.229588	6.513217
27	15	0	1.977727	0.236064	0.305525
28	15	0	-1.191026	-0.633422	0.210597
29	46	0	0.176559	0.759452	-0.969187
30	6	0	2.729754	-1.302608	-0.271073
31	6	0	3.783209	-1.886801	0.421132
32	6	0	2.239856	-1.904362	-1.427748
33	6	0	4.342819	-3.101040	0.010111
34	1	0	4.182456	-1.384126	1.296695
35	6	0	2.705268	-3.144711	-1.851660
36	1	0	1.456908	-1.396883	-1.978319
37	6	0	3.701068	-3.763391	-1.058237
38	6	0	3.172900	1.585931	0.131777
39	6	0	4.454342	1.376814	-0.373684

40	6	0	2.734548	2.883493	0.372617
41	6	0	5.340484	2.439358	-0.546735
42	1	0	4.757760	0.372601	-0.637989
43	6	0	3.567972	3.991768	0.216009
44	1	0	1.705623	3.038542	0.677162
45	6	0	4.904779	3.726237	-0.146783
46	6	0	-2.669455	-1.032220	-0.766620
47	6	0	-3.870422	-1.341014	-0.127389
48	6	0	-2.605425	-1.063260	-2.153397
49	6	0	-4.973653	-1.800780	-0.835497
50	1	0	-3.934660	-1.233473	0.945466
51	6	0	-3.691819	-1.494037	-2.929008
52	1	0	-1.690932	-0.739823	-2.644829
53	6	0	-4.815111	-1.975874	-2.231593
54	6	0	-1.805020	0.141090	1.728126
55	6	0	-1.742673	1.521991	1.829074
56	6	0	-2.390351	-0.600318	2.756445
57	6	0	-2.325172	2.212797	2.897907
58	1	0	-1.224687	2.076317	1.051522
59	6	0	-3.042868	0.027646	3.812704
60	1	0	-2.347377	-1.681772	2.707808
61	6	0	-3.081374	1.445838	3.802202
62	6	0	-2.048140	3.730884	2.971507
63	6	0	-2.750543	4.433695	1.796889
64	1	0	-2.545682	5.509822	1.817035
65	1	0	-2.399926	4.047198	0.835222
66	1	0	-3.835262	4.295149	1.844176
67	6	0	-2.446318	4.418914	4.288517
68	1	0	-2.037483	3.898258	5.158711
69	1	0	-2.030738	5.432441	4.280069
70	1	0	-3.524201	4.517181	4.419739
71	6	0	-0.522400	3.940527	2.835511
72	1	0	0.013078	3.462679	3.663199
73	1	0	-0.119811	3.544443	1.899690
74	1	0	-0.299283	5.012029	2.857799
75	6	0	-3.625063	-0.800070	4.976659
76	6	0	-2.949792	-0.344364	6.285031
77	1	0	-3.203498	0.690136	6.525518
78	1	0	-3.273576	-0.979176	7.117352
79	1	0	-1.860617	-0.413607	6.204023
80	6	0	-5.152807	-0.650463	5.102039
81	1	0	-5.650313	-0.836859	4.143916
82	1	0	-5.531339	-1.384062	5.822558
83	1	0	-5.441255	0.337656	5.460699
84	6	0	-3.334860	-2.299062	4.794522
85	1	0	-3.847409	-2.712564	3.918579
86	1	0	-2.264835	-2.505772	4.699698
87	1	0	-3.702933	-2.838620	5.672693
88	8	0	-3.880920	2.069182	4.728032
89	8	0	-5.809042	-2.636590	-2.909311
90	8	0	4.055422	-5.051545	-1.369493
91	8	0	5.829050	4.740949	-0.161574
92	6	0	6.349156	5.054647	1.128123
93	1	0	7.133595	5.798380	0.976749
94	1	0	5.581005	5.472762	1.785781
95	1	0	6.773960	4.167630	1.609545
96	6	0	2.919745	5.383117	0.414968
97	6	0	3.714412	6.560411	-0.176933
98	1	0	3.088107	7.457923	-0.128162
99	1	0	4.634804	6.780881	0.363029
100	1	0	3.964962	6.386217	-1.227567
101	6	0	2.678018	5.624995	1.915858
102	1	0	2.144073	6.570297	2.064440
103	1	0	2.075994	4.822368	2.353528
104	1	0	3.617965	5.681083	2.473255
105	6	0	-5.113027	2.529310	4.175352
106	1	0	-5.689322	2.947668	5.002290
107	1	0	-4.955261	3.303615	3.417557
108	1	0	-5.671883	1.705246	3.718774
109	6	0	-5.463114	-3.992685	-3.186713
110	1	0	-6.295684	-4.421263	-3.746989
111	1	0	-4.546783	-4.060564	-3.782846
112	1	0	-5.316515	-4.557214	-2.259511
113	6	0	-6.318232	-2.029746	-0.116366
114	6	0	-6.225432	-1.640581	1.370023
115	1	0	-5.531008	-2.282932	1.922963
116	1	0	-5.917930	-0.598133	1.506402
117	1	0	-7.212105	-1.754535	1.829690
118	6	0	-7.384987	-1.125433	-0.763861
119	1	0	-7.519223	-1.353338	-1.823662
120	1	0	-8.348702	-1.267343	-0.261806
121	1	0	-7.109828	-0.069109	-0.669687
122	6	0	-6.780261	-3.498035	-0.176070
123	1	0	-7.100854	-3.785676	-1.178255
124	1	0	-5.986944	-4.179453	0.149256
125	1	0	-7.634646	-3.637311	0.495440
126	6	0	-3.567641	-1.319862	-4.460794
127	6	0	-2.387121	-2.154917	-4.986317
128	1	0	-1.443224	-1.853550	-4.525234
129	1	0	-2.538017	-3.222105	-4.789843
130	1	0	-2.284468	-2.019732	-6.068635
131	6	0	-4.815463	-1.656408	-5.295163
132	1	0	-4.628175	-1.325516	-6.322765
133	1	0	-5.032121	-2.724693	-5.340101
134	1	0	-5.704670	-1.135293	-4.933563
135	6	0	-3.292835	0.178402	-4.709939
136	1	0	-3.131914	0.357876	-5.777845
137	1	0	-4.152323	0.776239	-4.390180
138	1	0	-2.412053	0.539500	-4.176359
139	6	0	6.699471	2.213593	-1.243743
140	6	0	1.553460	5.397157	-0.311083
141	1	0	1.673785	5.174793	-1.376528
142	1	0	0.836284	4.686936	0.107579
143	1	0	1.103018	6.390968	-0.219610
144	6	0	6.750342	3.081718	-2.516547
145	1	0	7.706243	2.931187	-3.031162
146	1	0	5.947798	2.803463	-3.208464
147	1	0	6.653536	4.144399	-2.285684
148	6	0	7.898311	2.549245	-0.336399
149	1	0	7.810135	2.053916	0.636570
150	1	0	8.821051	2.193700	-0.807980
151	1	0	8.007506	3.621625	-0.175108
152	6	0	6.864635	0.749232	-1.681066
153	1	0	6.066901	0.421665	-2.356071
154	1	0	7.812105	0.643722	-2.218570
155	1	0	6.896773	0.068492	-0.824995
156	6	0	3.482354	-6.014454	-0.486924
157	1	0	3.908965	-6.981630	-0.758694
158	1	0	3.715792	-5.792197	0.558764
159	1	0	2.395002	-6.051867	-0.601957
160	6	0	5.636676	-3.550312	0.735027
161	6	0	5.332238	-3.883632	2.205302
162	1	0	6.263372	-4.114558	2.734730
163	1	0	4.849055	-3.049697	2.723727
164	1	0	4.679395	-4.757945	2.281516
165	6	0	6.379573	-4.741132	0.104059
166	1	0	7.352999	-4.832513	0.598428
167	1	0	5.865504	-5.693446	0.238300
168	1	0	6.560771	-4.590164	-0.963064
169	6	0	6.631214	-2.367665	0.680528
170	1	0	6.878081	-2.125593	-0.358410
171	1	0	6.250419	-1.463692	1.163010
172	1	0	7.559580	-2.643642	1.191639
173	6	0	2.197035	-3.748315	-3.177186
174	6	0	1.142053	-2.839323	-3.830267
175	1	0	0.826899	-3.286798	-4.778084
176	1	0	0.249310	-2.737200	-3.202568
177	1	0	1.531511	-1.840477	-4.049321
178	6	0	1.543619	-5.130649	-2.997429
179	1	0	0.790625	-5.111670	-2.202973
180	1	0	1.039965	-5.416667	-3.927373
181	1	0	2.275506	-5.905828	-2.770835
182	6	0	3.387612	-3.862474	-4.149098
183	1	0	3.839078	-2.881005	-4.330571
184	1	0	4.159473	-4.529452	-3.757399
185	1	0	3.048664	-4.263373	-5.111133
186	8	0	1.044485	2.102630	-2.236954
187	6	0	-0.179427	2.201156	-2.844198
188	1	0	-0.940455	1.450402	-2.343567
189	6	0	-0.175336	1.768018	-4.275873
190	6	0	-0.502630	2.367210	-5.449679
191	8	0	0.243876	0.493328	-4.500932
192	6	0	-0.263223	1.393878	-6.474728
193	1	0	-0.879461	3.372345	-5.574301
194	6	0	0.183810	0.280791	-5.842060
195	1	0	-0.409598	1.514192	-7.538395
196	1	0	0.497216	-0.694736	-6.181733
197	6	0	-0.872489	3.541065	-2.595877
198	1	0	-0.565225	4.302329	-3.318110
199	1	0	-0.619679	3.874674	-1.587915
200	8	0	-2.279185	3.299554	-2.691416
201	6	0	-3.094808	4.143107	-2.041458
202	8	0	-2.695623	5.175679	-1.542184
203	6	0	-4.527324	3.625978	-2.006808

204	6	0	-4.522393	2.222305	-1.370498
205	1	0	-4.063914	2.239148	-0.374602
206	1	0	-5.551887	1.866418	-1.261554
207	1	0	-3.979586	1.503534	-1.990127
208	6	0	-5.387263	4.580581	-1.177405
209	1	0	-5.373999	5.591681	-1.594385
210	1	0	-6.422747	4.226317	-1.171973
211	1	0	-5.038819	4.635794	-0.141871
212	6	0	-5.057468	3.553935	-3.449142
213	1	0	-6.060921	3.116617	-3.447014
214	1	0	-5.121462	4.551923	-3.895076
215	1	0	-4.415307	2.936711	-4.082233

5a(*S*)

RwB97XD SCF energy -5021.614667 a.u.

RwB97XD SCF enthalpy -5019.625569 a.u.

RwB97XD SCF free energy -5019.869487 a.u.

Three lowest frequencies (cm^{-1}) 8.7, 16.5, 20.0

Cartesian coordinates:

Center	Atomic	Atomic	Coordinates	(Angstroms)	
Number	Number	Type	X	Y	Z
1	6	0	-1.190855	-1.966821	-2.079140
2	6	0	-0.981398	-2.500789	-3.331074
3	6	0	-1.163872	-1.773579	-4.504219
4	6	0	-1.534691	-0.451345	-4.487476
5	6	0	-1.738565	0.125090	-3.225040
6	6	0	-1.586225	-0.601591	-2.045884
7	1	0	-1.683300	0.112331	-5.400675
8	1	0	-2.048874	1.162487	-3.185198
9	6	0	-1.122483	-2.848897	-0.884477
10	6	0	-0.240785	-2.711069	0.223381
11	6	0	-2.019721	-3.892297	-0.811367
12	6	0	-0.337136	-3.577947	1.311891
13	6	0	-2.110195	-4.746688	0.282368
14	6	0	-1.285590	-4.609390	1.371861
15	1	0	0.349213	-3.479271	2.143599
16	1	0	-1.347922	-5.271835	2.226975
17	8	0	-0.925928	-2.574692	-5.574018
18	8	0	-0.595844	-3.768691	-3.639436
19	8	0	-2.971164	-4.226450	-1.725863
20	8	0	-3.113129	-5.641003	0.074146
21	6	0	-0.809251	-3.898583	-5.045959
22	1	0	0.043564	-4.398668	-5.503169
23	1	0	-1.744962	-4.441224	-5.221677
24	6	0	-3.466766	-5.497951	-1.304666
25	1	0	-2.979183	-6.288601	-1.886427
26	1	0	-4.551028	-5.520288	-1.410490
27	15	0	0.984992	-1.358008	0.246794
28	15	0	-1.819516	0.214403	-0.436956
29	46	0	0.119913	0.691091	0.704166
30	6	0	1.780981	-1.369945	-1.372707
31	6	0	2.333486	-2.531367	-1.894915
32	6	0	1.827258	-0.188486	-2.107765
33	6	0	2.919161	-2.554638	-3.165410
34	1	0	2.310114	-3.439143	-1.300427
35	6	0	2.346941	-0.151517	-3.395697
36	1	0	1.427943	0.710058	-1.656661
37	6	0	2.816302	-1.375252	-3.933655
38	6	0	2.180337	-1.740064	1.551004
39	6	0	3.515067	-2.036160	1.288464
40	6	0	1.756653	-1.572943	2.864604
41	6	0	4.414375	-2.269700	2.330433
42	1	0	3.851553	-2.077969	0.260886
43	6	0	2.609999	-1.762522	3.951228
44	1	0	0.733243	-1.262427	3.046070
45	6	0	3.913565	-2.210890	3.653473
46	6	0	-2.565409	1.839240	-0.748755
47	6	0	-3.842549	2.138095	-0.288314
48	6	0	-1.764289	2.854558	-1.257271
49	6	0	-4.368939	3.423202	-0.393274
50	1	0	-4.428913	1.354353	0.168952
51	6	0	-2.220442	4.168099	-1.380137
52	1	0	-0.743681	2.617783	-1.541354
53	6	0	-3.567717	4.405269	-1.025918
54	6	0	-3.049666	-0.703212	0.506358
55	6	0	-2.938341	-0.694129	1.887156
56	6	0	-4.154072	-1.299962	-0.101803
57	6	0	-3.952054	-1.193145	2.711774
58	1	0	-2.042780	-0.270724	2.333103
59	6	0	-5.209991	-1.787701	0.661152
60	1	0	-4.187521	-1.358090	-1.183134
61	6	0	-5.128035	-1.620340	2.068275
62	6	0	-3.646631	-1.244927	4.225547
63	6	0	-3.553322	0.186081	4.787186
64	1	0	-3.312153	0.154382	5.855603
65	1	0	-2.770643	0.764995	4.284877
66	1	0	-4.499010	0.725286	4.670798
67	6	0	-4.639626	-2.058786	5.072917
68	1	0	-4.802244	-3.056693	4.656933
69	1	0	-4.214297	-2.179668	6.075148
70	1	0	-5.607965	-1.571269	5.190873
71	6	0	-2.274589	-1.933921	4.405268
72	1	0	-2.293047	-2.955503	4.009338
73	1	0	-1.461706	-1.394962	3.911547
74	1	0	-2.029864	-1.984752	5.471046
75	6	0	-6.370619	-2.550711	-0.008762
76	6	0	-6.418801	-3.972349	0.585770
77	1	0	-6.654572	-3.951413	1.652097
78	1	0	-7.186397	-4.566832	0.077703
79	1	0	-5.455349	-4.477863	0.462790
80	6	0	-7.733662	-1.860770	0.185819
81	1	0	-7.686523	-0.801539	-0.090006
82	1	0	-8.477909	-2.339076	-0.460534
83	1	0	-8.093934	-1.940135	1.211814
84	6	0	-6.146371	-2.684377	-1.524200
85	1	0	-6.182464	-1.713638	-2.031002
86	1	0	-5.192523	-3.163679	-1.762649
87	1	0	-6.944122	-3.303093	-1.946831
88	8	0	-6.249511	-1.905315	2.806785
89	8	0	-4.135719	5.633291	-1.259359
90	8	0	3.200481	-1.384190	-5.251222
91	8	0	4.766035	-2.569824	4.668315
92	6	0	4.529811	-3.881545	5.171358
93	1	0	5.321402	-4.088385	5.893812
94	1	0	3.560479	-3.957624	5.672411
95	1	0	4.564667	-4.626083	4.369347
96	6	0	2.047396	-1.412547	5.350456
97	6	0	3.114152	-1.178084	6.435402
98	1	0	2.619596	-0.766027	7.321634
99	1	0	3.626366	-2.087812	6.747854
100	1	0	3.866682	-0.455159	6.106879
101	6	0	1.078036	-2.517756	5.807117
102	1	0	0.597590	-2.233228	6.749936
103	1	0	0.293728	-2.683440	5.061884
104	1	0	1.591278	-3.470647	5.967090
105	6	0	-6.969942	-0.734143	3.188018
106	1	0	-7.854279	-1.074466	3.729562
107	1	0	-6.372820	-0.088245	3.839270
108	1	0	-7.278967	-0.158029	2.309030
109	6	0	-4.563691	5.811505	-2.607618
110	1	0	-5.069513	6.777540	-2.650976
111	1	0	-3.720295	5.816687	-3.304560
112	1	0	-5.259315	5.021765	-2.909565
113	6	0	-5.729397	3.741243	0.265027
114	6	0	-6.311353	2.504801	0.977731
115	1	0	-6.544153	1.695885	0.276087
116	1	0	-5.641485	2.111156	1.750266
117	1	0	-7.245827	2.789824	1.470787
118	6	0	-5.515625	4.830473	1.334911
119	1	0	-5.133392	5.754430	0.895691
120	1	0	-6.465462	5.055680	1.832568
121	1	0	-4.806424	4.491310	2.098170
122	6	0	-6.785924	4.214110	-0.750696
123	1	0	-6.567988	5.209739	-1.137401
124	1	0	-6.867363	3.518902	-1.593133

125	1	0	-7.764962	4.260948	-0.261293
126	6	0	-1.180811	5.214492	-1.848706
127	6	0	-0.910189	5.028167	-3.353454
128	1	0	-0.579991	4.008393	-3.575518
129	1	0	-1.803723	5.227707	-3.952923
130	1	0	-0.124340	5.717028	-3.682887
131	6	0	-1.559612	6.680292	-1.572376
132	1	0	-0.686021	7.305883	-1.786358
133	1	0	-2.375337	7.044693	-2.196507
134	1	0	-1.831177	6.835701	-0.524013
135	6	0	0.144699	4.979017	-1.084115
136	1	0	0.876840	5.723183	-1.407401
137	1	0	0.002828	5.090761	-0.003258
138	1	0	0.590910	3.999829	-1.273942
139	6	0	5.917878	-2.468261	2.038346
140	6	0	1.256951	-0.085459	5.247753
141	1	0	1.882251	0.716022	4.840120
142	1	0	0.359078	-0.166154	4.629761
143	1	0	0.925138	0.216304	6.246564
144	6	0	6.686913	-1.285991	2.661262
145	1	0	7.759545	-1.389687	2.460920
146	1	0	6.351049	-0.337575	2.227707
147	1	0	6.545660	-1.240394	3.743755
148	6	0	6.474549	-3.798674	2.582393
149	1	0	5.847908	-4.645046	2.280866
150	1	0	7.474812	-3.964934	2.167388
151	1	0	6.568461	-3.799726	3.667907
152	6	0	6.198540	-2.465117	0.528140
153	1	0	5.873844	-1.539515	0.045004
154	1	0	7.277137	-2.556747	0.366096
155	1	0	5.717828	-3.311579	0.028454
156	6	0	2.212137	-1.939577	-6.116745
157	1	0	2.628009	-1.908816	-7.125464
158	1	0	1.979135	-2.976014	-5.852101
159	1	0	1.288650	-1.355048	-6.085931
160	6	0	3.652407	-3.859761	-3.563189
161	6	0	2.628449	-4.991272	-3.760348
162	1	0	3.146532	-5.938385	-3.947614
163	1	0	1.993194	-5.123311	-2.878994
164	1	0	1.981210	-4.789674	-4.618734
165	6	0	4.545594	-3.773838	-4.813258
166	1	0	5.122513	-4.702812	-4.880948
167	1	0	3.985521	-3.679226	-5.743488
168	1	0	5.256712	-2.945622	-4.750669
169	6	0	4.602684	-4.236536	-2.403988
170	1	0	5.341099	-3.444445	-2.241205
171	1	0	4.081038	-4.416142	-1.460647
172	1	0	5.143468	-5.155350	-2.654300
173	6	0	2.485234	1.200823	-4.127824
174	6	0	1.901704	2.349581	-3.286097
175	1	0	2.029297	3.293158	-3.825528
176	1	0	0.828643	2.215663	-3.106257
177	1	0	2.408198	2.456783	-2.321769
178	6	0	1.762562	1.231913	-5.486708
179	1	0	0.727883	0.886116	-5.393592
180	1	0	1.738163	2.261133	-5.861153
181	1	0	2.270384	0.623741	-6.235182
182	6	0	3.984842	1.488504	-4.334651
183	1	0	4.503341	1.557031	-3.372247
184	1	0	4.463843	0.708973	-4.932290
185	1	0	4.114264	2.444341	-4.854782
186	8	0	1.909590	1.212015	1.535403
187	6	0	2.465224	2.332184	0.900909
188	6	0	3.896656	2.061258	0.542268
189	6	0	5.102012	2.461699	1.029834
190	8	0	4.087190	1.185945	-0.482707
191	6	0	6.100808	1.789384	0.249041
192	1	0	5.264494	3.162666	1.836487
193	6	0	5.425236	1.029715	-0.649361
194	1	0	7.173959	1.866523	0.349718
195	1	0	5.727822	0.362931	-1.442653
196	6	0	2.330708	3.536204	1.825854
197	1	0	2.865178	3.360597	2.764596
198	1	0	1.274366	3.705924	2.050305
199	8	0	2.877263	4.686663	1.165386
200	6	0	2.930568	5.817135	1.878401
201	8	0	2.447097	5.908196	2.990285
202	6	0	3.683299	6.923806	1.148480
203	6	0	3.035916	7.168743	-0.223669
204	1	0	1.981859	7.446547	-0.119636
205	1	0	3.554868	7.989522	-0.729260
206	1	0	3.101151	6.282507	-0.860230
207	6	0	3.636540	8.200487	1.989866
208	1	0	4.098726	8.050546	2.969885
209	1	0	4.180841	8.998378	1.474937
210	1	0	2.607360	8.537589	2.146642
211	6	0	5.140817	6.461672	0.964485
212	1	0	5.710724	7.249122	0.460774
213	1	0	5.617344	6.263860	1.930610
214	1	0	5.199143	5.553566	0.357557
215	1	0	-0.532398	2.518518	1.125463
216	1	0	-0.720006	2.101774	1.745530
217	1	0	1.935999	2.570383	-0.039535

TS2a(*S*)

RwB97XD SCF energy -5021.600098 a.u.
RwB97XD SCF enthalpy -5019.613175 a.u.
RwB97XD SCF free energy -5019.860389 a.u.
Three lowest frequencies (cm^{-1}) -1057, 6.0, 12.2
Imaginary frequency (cm^{-1}) -1057
Cartesian coordinates:

Center Number	Atomic Number	Atomic Type	Coordinates (Angstroms) X	Y	Z
1	6	0	1.409162	-0.879209	2.461758
2	6	0	1.275628	-0.850052	3.832449
3	6	0	1.403500	0.316851	4.583588
4	6	0	1.660976	1.531244	3.995022
5	6	0	1.811106	1.530630	2.600658
6	6	0	1.701852	0.365803	1.841691
7	1	0	1.762477	2.440971	4.574508
8	1	0	2.035372	2.472141	2.113072
9	6	0	1.309038	-2.180472	1.753064
10	6	0	0.346208	-2.520909	0.762801
11	6	0	2.220759	-3.163146	2.071130
12	6	0	0.382242	-3.771587	0.147231
13	6	0	2.248583	-4.407541	1.448490
14	6	0	1.343687	-4.741521	0.470351
15	1	0	-0.356734	-4.023779	-0.603990
16	1	0	1.358977	-5.706758	-0.021097
17	8	0	1.226219	0.027030	5.896897
18	8	0	0.994503	-1.889526	4.661698
19	8	0	3.235317	-3.087384	2.973839
20	8	0	3.278232	-5.138251	1.951559
21	6	0	1.277093	-1.400139	5.970369
22	1	0	0.525573	-1.760866	6.670594
23	1	0	2.287612	-1.710798	6.263948
24	6	0	3.702494	-4.427550	3.116932
25	1	0	3.244251	-4.880207	4.004843
26	1	0	4.790704	-4.431176	3.175625
27	15	0	-0.887582	-1.281694	0.230883
28	15	0	1.878732	0.473157	0.033623
29	46	0	-0.023869	0.309074	-1.200784
30	6	0	-1.596093	-0.560714	1.728781
31	6	0	-2.093085	-1.325257	2.776325
32	6	0	-1.657011	0.828028	1.800642
33	6	0	-2.640296	-0.730573	3.920539
34	1	0	-2.054543	-2.407111	2.702800
35	6	0	-2.146908	1.483895	2.920647
36	1	0	-1.316159	1.397717	0.947824
37	6	0	-2.556966	0.676246	4.008897
38	6	0	-2.140616	-2.174093	-0.726514
39	6	0	-3.384624	-2.551603	-0.229374
40	6	0	-1.820268	-2.451420	-2.051696
41	6	0	-4.282053	-3.276925	-1.015911
42	1	0	-3.649693	-2.277197	0.783105
43	6	0	-2.675970	-3.151649	-2.900124
44	1	0	-0.874340	-2.093317	-2.438625

45	6	0	-3.879699	-3.625976	-2.330044
46	6	0	2.575559	2.115904	-0.299200
47	6	0	3.946779	2.309215	-0.415172
48	6	0	1.712367	3.185521	-0.502049
49	6	0	4.480709	3.567573	-0.682174
50	1	0	4.606249	1.460345	-0.303715
51	6	0	2.173234	4.469650	-0.798847
52	1	0	0.645379	3.001609	-0.455629
53	6	0	3.577010	4.653174	-0.808047
54	6	0	3.104874	-0.740829	-0.499996
55	6	0	2.913848	-1.371924	-1.719646
56	6	0	4.244590	-1.024990	0.254400
57	6	0	3.866658	-2.237285	-2.264694
58	1	0	1.993099	-1.188242	-2.264333
59	6	0	5.256530	-1.831908	-0.256680
60	1	0	4.337566	-0.592198	1.242772
61	6	0	5.085175	-2.343371	-1.569573
62	6	0	3.445987	-3.005424	-3.538725
63	6	0	3.249104	-2.016559	-4.702614
64	1	0	2.913690	-2.552847	-5.597058
65	1	0	2.495532	-1.257779	-4.467198
66	1	0	4.181869	-1.499640	-4.949808
67	6	0	4.398630	-4.125987	-3.989456
68	1	0	4.637881	-4.811234	-3.172770
69	1	0	3.898070	-4.700050	-4.776755
70	1	0	5.332722	-3.753198	-4.410621
71	6	0	2.095082	-3.696640	-3.240123
72	1	0	2.200503	-4.429167	-2.432746
73	1	0	1.311632	-2.990517	-2.951051
74	1	0	1.748052	-4.221597	-4.135986
75	6	0	6.471705	-2.209693	0.614072
76	6	0	6.556872	-3.746741	0.704810
77	1	0	6.781456	-4.190621	-0.266459
78	1	0	7.345689	-4.037780	1.407291
79	1	0	5.610493	-4.169472	1.056594
80	6	0	7.791782	-1.650366	0.050459
81	1	0	7.713966	-0.575680	-0.148534
82	1	0	8.592647	-1.796379	0.783716
83	1	0	8.090744	-2.157172	-0.867316
84	6	0	6.318605	-1.664721	2.043997
85	1	0	6.334676	-0.569495	2.072561
86	1	0	5.395109	-2.010019	2.518546
87	1	0	7.159578	-2.017178	2.648952
88	8	0	6.153114	-2.974558	-2.151278
89	8	0	4.124540	5.892094	-1.013500
90	8	0	-2.903561	1.315232	5.170809
91	8	0	-4.752112	-4.388444	-3.061812
92	6	0	-4.436579	-5.762494	-3.254007
93	1	0	-5.375382	-6.320734	-3.213003
94	1	0	-3.987254	-5.928441	-4.237526
95	1	0	-3.761161	-6.133753	-2.477090
96	6	0	-2.235588	-3.326524	-4.372771
97	6	0	-3.418980	-3.276897	-5.356585
98	1	0	-3.032959	-3.322191	-6.380318
99	1	0	-4.130474	-4.090056	-5.230237
100	1	0	-3.966551	-2.334799	-5.245723
101	6	0	-1.436915	-4.635383	-4.527349
102	1	0	-1.035891	-4.714651	-5.544230
103	1	0	-0.592575	-4.649463	-3.830078
104	1	0	-2.037408	-5.525779	-4.334679
105	6	0	6.824416	-2.158477	-3.106878
106	1	0	7.654996	-2.750162	-3.495100
107	1	0	6.164189	-1.874067	-3.932488
108	1	0	7.213359	-1.244742	-2.644153
109	6	0	4.131755	6.785686	0.095810
110	1	0	5.044799	7.381525	0.025749
111	1	0	3.277353	7.468371	0.062744
112	1	0	4.128441	6.243754	1.046410
113	6	0	6.004254	3.714551	-0.884326
114	6	0	6.718750	2.352985	-0.786865
115	1	0	6.624498	1.905289	0.208792
116	1	0	6.350418	1.634567	-1.527192
117	1	0	7.785890	2.501567	-0.978110
118	6	0	6.286928	4.278354	-2.290219
119	1	0	5.822677	5.255513	-2.430723
120	1	0	7.367425	4.386617	-2.436533
121	1	0	5.909611	3.599229	-3.062559
122	6	0	6.632093	4.628256	0.184785
123	1	0	6.354807	5.672424	0.047400
124	1	0	6.339816	4.317553	1.193633
125	1	0	7.724050	4.569003	0.120198
126	6	0	1.112262	5.548842	-1.123633
127	6	0	0.664179	6.254570	0.170138
128	1	0	0.262114	5.528357	0.883819
129	1	0	1.481071	6.785525	0.662494
130	1	0	-0.125282	6.980768	-0.053710
131	6	0	1.601270	6.572922	-2.164556
132	1	0	0.762149	7.215610	-2.450502
133	1	0	2.401720	7.215556	-1.804220
134	1	0	1.958437	6.067369	-3.067837
135	6	0	-0.144549	4.890332	-1.740768
136	1	0	-0.821743	5.671615	-2.097472
137	1	0	0.111985	4.253708	-2.594993
138	1	0	-0.712339	4.293247	-1.021636
139	6	0	-5.669399	-3.659709	-0.452632
140	6	0	-1.295868	-2.171663	-4.792817
141	1	0	-1.717764	-1.192858	-4.544830
142	1	0	-0.305501	-2.246320	-4.333770
143	1	0	-1.139872	-2.219368	-5.875102
144	6	0	-6.788371	-3.155044	-1.384229
145	1	0	-7.764985	-3.363240	-0.933133
146	1	0	-6.706668	-2.073658	-1.529293
147	1	0	-6.748512	-3.635123	-2.361723
148	6	0	-5.787536	-5.183453	-0.261500
149	1	0	-4.956838	-5.569530	0.339123
150	1	0	-6.719261	-5.415822	0.266263
151	1	0	-5.807548	-5.720308	-1.208666
152	6	0	-5.904731	-3.024459	0.926777
153	1	0	-5.815725	-1.934166	0.898710
154	1	0	-6.917344	-3.267639	1.263262
155	1	0	-5.210884	-3.413927	1.676849
156	6	0	-1.872034	1.287073	6.153542
157	1	0	-2.249768	1.835108	7.018617
158	1	0	-1.627157	0.263556	6.452992
159	1	0	-0.960958	1.768367	5.784641
160	6	0	-3.315570	-1.673467	4.947177
161	6	0	-2.247207	-2.484798	5.698959
162	1	0	-2.726599	-3.222863	6.351600
163	1	0	-1.582985	-3.016657	5.011236
164	1	0	-1.633254	-1.833927	6.327802
165	6	0	-4.231612	-0.978591	5.971204
166	1	0	-4.786289	-1.751854	6.513940
167	1	0	-3.687342	-0.392740	6.711189
168	1	0	-4.958585	-0.323556	5.483705
169	6	0	-4.227102	-2.650508	4.172053
170	1	0	-4.976619	-2.101425	3.593317
171	1	0	-3.674893	-3.301874	3.489606
172	1	0	-4.755172	-3.299866	4.877681
173	6	0	-2.336784	3.015072	2.888808
174	6	0	-1.818725	3.610316	1.567791
175	1	0	-2.004776	4.688711	1.564276
176	1	0	-0.739133	3.460517	1.447685
177	1	0	-2.331026	3.188504	0.698175
178	6	0	-1.605362	3.747844	4.027512
179	1	0	-0.554622	3.444721	4.083071
180	1	0	-1.631766	4.826804	3.839567
181	1	0	-2.075355	3.571550	4.994758
182	6	0	-3.848046	3.308754	2.970599
183	1	0	-4.377661	2.846994	2.130159
184	1	0	-4.274860	2.929227	3.902594
185	1	0	-4.024211	4.389548	2.928092
186	8	0	-1.603188	0.650030	-2.625525
187	6	0	-2.417567	1.703669	-2.157868
188	6	0	-3.689036	1.208100	-1.543423
189	6	0	-4.909238	0.847168	-2.027285
190	8	0	-3.684443	1.093901	-0.188861
191	6	0	-5.705135	0.493132	-0.890487
192	1	0	-5.214120	0.861402	-3.063305
193	6	0	-4.905557	0.657529	0.195549
194	1	0	-6.735847	0.169904	-0.885329
195	1	0	-5.047417	0.517580	1.256694
196	6	0	-2.691316	2.697882	-3.286573
197	1	0	-3.366768	2.297145	-4.044988
198	1	0	-1.750763	2.993251	-3.756943
199	8	0	-3.246831	3.863817	-2.675592
200	6	0	-4.539244	4.165154	-2.891342
201	8	0	-5.202577	3.669001	-3.774284
202	6	0	-5.050002	5.159136	-1.852940
203	6	0	-4.013117	6.262398	-1.596397
204	1	0	-3.767293	6.801642	-2.517268
205	1	0	-4.419264	6.983734	-0.879682
206	1	0	-3.089361	5.852165	-1.180944
207	6	0	-6.365909	5.764804	-2.344028
208	1	0	-7.113933	4.989848	-2.529494

209	1	0	-6.760446	6.453609	-1.590353
210	1	0	-6.220537	6.322920	-3.274279
211	6	0	-5.283041	4.355534	-0.556385
212	1	0	-5.649882	5.026512	0.227276
213	1	0	-6.024851	3.564403	-0.705837
214	1	0	-4.355467	3.893226	-0.206388
215	1	0	0.567801	1.408498	-2.477229
216	1	0	-0.286963	1.219690	-2.748454
217	1	0	-1.902569	2.281728	-1.372890

6a(*S*)•2a

RwB97XD SCF energy -5021.644625 a.u.
RwB97XD SCF enthalpy -5019.652560 a.u.
RwB97XD SCF free energy -5019.898250 a.u.
Three lowest frequencies (cm^{-1}) 15.5, 16.4, 20.0
Cartesian coordinates:

Center Number	Atomic Number	Atomic Type	Coordinates (Angstroms) X	Y	Z
1	6	0	1.053763	-0.874841	2.382480
2	6	0	0.781475	-0.715777	3.724705
3	6	0	0.970480	0.481078	4.407404
4	6	0	1.420253	1.609914	3.769201
5	6	0	1.700079	1.481934	2.401669
6	6	0	1.542011	0.280926	1.708560
7	1	0	1.570206	2.546767	4.292263
8	1	0	2.073425	2.357609	1.884768
9	6	0	0.876335	-2.229748	1.791552
10	6	0	-0.082215	-2.587763	0.806109
11	6	0	1.662003	-3.256951	2.267619
12	6	0	-0.144816	-3.892975	0.323014
13	6	0	1.585210	-4.559082	1.786854
14	6	0	0.701243	-4.909409	0.795875
15	1	0	-0.878848	-4.152385	-0.431677
16	1	0	0.639780	-5.920795	0.411523
17	8	0	0.640557	0.315707	5.714837
18	8	0	0.293328	-1.653108	4.585445
19	8	0	2.626527	-3.184697	3.228053
20	8	0	2.503855	-5.332316	2.436547
21	6	0	0.464846	-1.092658	5.887186
22	1	0	-0.424627	-1.278265	6.487295
23	1	0	1.364492	-1.514585	6.350069
24	6	0	2.923871	-4.543153	3.551522
25	1	0	2.352335	-4.836946	4.440561
26	1	0	3.996935	-4.657832	3.700588
27	15	0	-1.168124	-1.272939	0.141138
28	15	0	1.890147	0.271467	-0.092940
29	46	0	0.003429	0.243906	-1.300667
30	6	0	-1.956504	-0.533520	1.599230
31	6	0	-2.582348	-1.290893	2.580132
32	6	0	-1.923601	0.854534	1.721061
33	6	0	-3.175178	-0.699718	3.702456
34	1	0	-2.615479	-2.369514	2.464471
35	6	0	-2.451717	1.506535	2.830166
36	1	0	-1.448010	1.422256	0.932020
37	6	0	-3.008782	0.693882	3.847896
38	6	0	-2.438271	-2.112969	-0.857070
39	6	0	-3.776731	-2.234709	-0.493529
40	6	0	-2.041028	-2.507301	-2.130743
41	6	0	-4.710214	-2.814158	-1.356676
42	1	0	-4.090227	-1.862671	0.472864
43	6	0	-2.923292	-3.074233	-3.052501
44	1	0	-1.006634	-2.345686	-2.416433
45	6	0	-4.242263	-3.295642	-2.603717
46	6	0	2.914723	1.756148	-0.353147
47	6	0	4.303930	1.693408	-0.321496
48	6	0	2.293680	2.976984	-0.582369
49	6	0	5.087403	2.837855	-0.449190
50	1	0	4.782288	0.732517	-0.202483
51	6	0	3.015396	4.166857	-0.716930
52	1	0	1.212092	2.998958	-0.660302
53	6	0	4.415458	4.079212	-0.555308
54	6	0	2.967063	-1.142042	-0.439530
55	6	0	2.787342	-1.822239	-1.633209
56	6	0	4.001492	-1.519714	0.420936
57	6	0	3.665565	-2.827122	-2.053340
58	1	0	1.938210	-1.557604	-2.256301
59	6	0	4.939999	-2.473674	0.038796
60	1	0	4.078283	-1.037643	1.388069
61	6	0	4.801776	-3.041504	-1.254132
62	6	0	3.261693	-3.607067	-3.324130
63	6	0	3.328665	-2.677972	-4.550122
64	1	0	2.993607	-3.213429	-5.445469
65	1	0	2.686016	-1.800113	-4.425392
66	1	0	4.349358	-2.325343	-4.730326
67	6	0	4.075045	-4.879972	-3.615210
68	1	0	4.109414	-5.547593	-2.749798
69	1	0	3.582095	-5.418361	-4.432220
70	1	0	5.097129	-4.676718	-3.935863
71	6	0	1.800754	-4.075265	-3.140487
72	1	0	1.706034	-4.742726	-2.276833
73	1	0	1.103216	-3.244678	-3.002711
74	1	0	1.483578	-4.623785	-4.032876
75	6	0	6.026064	-2.948122	1.026489
76	6	0	5.859008	-4.465960	1.236714
77	1	0	6.054261	-5.018815	0.315482
78	1	0	6.558059	-4.820504	2.002485
79	1	0	4.841671	-4.703110	1.562293
80	6	0	7.455302	-2.650830	0.535007
81	1	0	7.570906	-1.598707	0.253195
82	1	0	8.165403	-2.856929	1.343734
83	1	0	7.738872	-3.274985	-0.312561
84	6	0	5.870699	-2.262553	2.393444
85	1	0	6.045466	-1.182363	2.331605
86	1	0	4.882389	-2.428574	2.831374
87	1	0	6.613937	-2.672716	3.084506
88	8	0	5.821794	-3.838745	-1.712108
89	8	0	5.187212	5.215945	-0.535485
90	8	0	-3.426563	1.317106	4.998658
91	8	0	-5.145887	-3.953465	-3.404112
92	6	0	-5.027079	-5.373102	-3.381348
93	1	0	-5.919715	-5.770326	-3.868308
94	1	0	-4.144910	-5.715898	-3.929025
95	1	0	-4.971743	-5.752506	-2.356011
96	6	0	-2.369468	-3.367764	-4.467303
97	6	0	-3.439809	-3.442350	-5.571769
98	1	0	-2.933646	-3.477605	-6.542367
99	1	0	-4.073406	-4.325643	-5.506730
100	1	0	-4.084998	-2.558079	-5.563625
101	6	0	-1.558299	-4.675990	-4.432786
102	1	0	-1.060232	-4.839440	-5.395145
103	1	0	-0.792998	-4.636650	-3.652823
104	1	0	-2.191473	-5.544134	-4.230169
105	6	0	6.671707	-3.169624	-2.641085
106	1	0	7.451941	-3.880760	-2.917739
107	1	0	6.126598	-2.860728	-3.538690
108	1	0	7.128911	-2.282410	-2.189892
109	6	0	5.204032	5.878784	0.726314
110	1	0	5.993254	6.631251	0.676105
111	1	0	4.253098	6.377285	0.935164
112	1	0	5.417592	5.178116	1.539905
113	6	0	6.622245	2.703922	-0.559247
114	6	0	7.058070	1.228493	-0.494711
115	1	0	6.820951	0.771957	0.472629
116	1	0	6.600766	0.623933	-1.285004
117	1	0	8.143070	1.172068	-0.626586
118	6	0	7.068757	3.258917	-1.926158
119	1	0	6.825770	4.318936	-2.028233
120	1	0	8.152663	3.144807	-2.040068
121	1	0	6.585678	2.714376	-2.744954
122	6	0	7.370113	3.441867	0.567137
123	1	0	7.309699	4.524758	0.459080
124	1	0	6.980721	3.163761	1.552285
125	1	0	8.430987	3.169312	0.538849

126	6	0	2.204042	5.442652	-1.046915
127	6	0	1.505585	5.944064	0.231072
128	1	0	0.814643	5.188602	0.622412
129	1	0	2.222897	6.180692	1.022060
130	1	0	0.930128	6.851149	0.017373
131	6	0	3.026780	6.583649	-1.673420
132	1	0	2.333285	7.355638	-2.023744
133	1	0	3.714825	7.060084	-0.976088
134	1	0	3.601458	6.234674	-2.536800
135	6	0	1.113080	5.090705	-2.085883
136	1	0	0.615884	6.005825	-2.420988
137	1	0	1.544583	4.601778	-2.966004
138	1	0	0.336354	4.440622	-1.676511
139	6	0	-6.212597	-2.815195	-0.992734
140	6	0	-1.417430	-2.223340	-4.891520
141	1	0	-1.924068	-1.252773	-4.849904
142	1	0	-0.509008	-2.165704	-4.286121
143	1	0	-1.094814	-2.389611	-5.924174
144	6	0	-6.956041	-1.919939	-2.003453
145	1	0	-8.025984	-1.894694	-1.766387
146	1	0	-6.577375	-0.892734	-1.962386
147	1	0	-6.843942	-2.290969	-3.024968
148	6	0	-6.839703	-4.223171	-0.987242
149	1	0	-6.241107	-4.922671	-0.394027
150	1	0	-7.834530	-4.170794	-0.531282
151	1	0	-6.964055	-4.629093	-1.990342
152	6	0	-6.446188	-2.235112	0.410069
153	1	0	-6.057023	-1.218892	0.516399
154	1	0	-7.522252	-2.194422	0.605978
155	1	0	-5.992834	-2.864266	1.181678
156	6	0	-2.512046	1.177665	6.082451
157	1	0	-2.957466	1.693185	6.935475
158	1	0	-2.348103	0.126782	6.340235
159	1	0	-1.546729	1.632670	5.844894
160	6	0	-3.994001	-1.632634	4.630118
161	6	0	-3.047478	-2.573046	5.396381
162	1	0	-3.629091	-3.287990	5.989195
163	1	0	-2.404270	-3.141984	4.717568
164	1	0	-2.406032	-2.015574	6.084819
165	6	0	-4.922231	-0.924352	5.633592
166	1	0	-5.570302	-1.680337	6.090539
167	1	0	-4.391160	-0.426426	6.444690
168	1	0	-5.565227	-0.190474	5.140091
169	6	0	-4.928782	-2.485225	3.743164
170	1	0	-5.602761	-1.842599	3.168165
171	1	0	-4.388634	-3.129114	3.044324
172	1	0	-5.541918	-3.136266	4.375077
173	6	0	-2.520092	3.048760	2.861649
174	6	0	-1.864491	3.669861	1.614948
175	1	0	-1.941712	4.759082	1.686876
176	1	0	-0.800451	3.417125	1.533583
177	1	0	-2.369981	3.368964	0.692877
178	6	0	-1.823502	3.666081	4.088800
179	1	0	-0.814644	3.260529	4.217798
180	1	0	-1.731560	4.748459	3.948277
181	1	0	-2.385416	3.502607	5.008303
182	6	0	-4.006291	3.459246	2.846252
183	1	0	-4.503447	3.073456	1.949066
184	1	0	-4.533600	3.078957	3.724534
185	1	0	-4.096252	4.551651	2.839708
186	8	0	-1.863794	0.685930	-2.403425
187	6	0	-2.409203	1.933778	-1.944418
188	6	0	-3.883548	1.875618	-1.756537
189	6	0	-4.948570	2.328027	-2.471142
190	8	0	-4.310587	1.326898	-0.584732
191	6	0	-6.111019	2.039036	-1.687613
192	1	0	-4.911254	2.827523	-3.429023
193	6	0	-5.661303	1.434765	-0.557923
194	1	0	-7.140407	2.257977	-1.931618
195	1	0	-6.149695	1.053362	0.325508
196	6	0	-1.977770	3.088259	-2.834300
197	1	0	-2.492625	3.081530	-3.798979
198	1	0	-0.897804	3.047700	-3.002623
199	8	0	-2.323370	4.255875	-2.092162
200	6	0	-2.427210	5.415427	-2.754033
201	8	0	-2.107678	5.528440	-3.919841
202	6	0	-2.982173	6.518577	-1.860661
203	6	0	-1.890759	6.908466	-0.848113
204	1	0	-1.017268	7.333078	-1.352215
205	1	0	-2.285125	7.662119	-0.158687
206	1	0	-1.567336	6.045314	-0.259330
207	6	0	-3.350698	7.723548	-2.728678
208	1	0	-4.130188	7.467332	-3.452837
209	1	0	-3.727742	8.530082	-2.092436
210	1	0	-2.483038	8.098216	-3.278730
211	6	0	-4.222824	5.996256	-1.115424
212	1	0	-4.671109	6.815164	-0.543957
213	1	0	-4.976702	5.617312	-1.813683
214	1	0	-3.966585	5.193866	-0.418235
215	1	0	0.843033	1.133596	-2.284374
216	1	0	-1.933103	0.598654	-3.363747
217	1	0	-1.953639	2.082884	-0.964188

2.3. **Catalytic cycle 2a – 1a (*R*).**

3a(*R*)

RwB97XD SCF energy -5020.433606 a.u.
RwB97XD SCF enthalpy -5018.465266 a.u.
RwB97XD SCF free energy -5018.709479 a.u.
Three lowest frequencies (cm^{-1}) 8.0, 15.1,19.0
Cartesian coordinates:

Center Number	Atomic Number	Atomic Type	X	Y	Z
1	6	0	0.853712	-0.699372	2.578883
2	6	0	0.458372	-0.391698	3.861194
3	6	0	0.795819	0.793010	4.505125
4	6	0	1.586014	1.737212	3.897360
5	6	0	2.010909	1.449487	2.591843
6	6	0	1.664776	0.270739	1.929139
7	1	0	1.866731	2.658028	4.394425
8	1	0	2.626082	2.187286	2.090861
9	6	0	0.412448	-2.006571	2.024299
10	6	0	-0.584561	-2.169530	1.028033
11	6	0	0.929106	-3.164183	2.562313
12	6	0	-0.957529	-3.445638	0.609384
13	6	0	0.543837	-4.431944	2.143681
14	6	0	-0.393999	-4.609603	1.156052
15	1	0	-1.715033	-3.558098	-0.158084
16	1	0	-0.696343	-5.595670	0.823128
17	8	0	0.266235	0.794930	5.757684
18	8	0	-0.290932	-1.165960	4.693895
19	8	0	1.874797	-3.274757	3.538698
20	8	0	1.240780	-5.369584	2.848559
21	6	0	-0.626508	-0.322960	5.795330
22	1	0	-1.652226	0.035164	5.672045
23	1	0	-0.490739	-0.866924	6.729872
24	6	0	1.852240	-4.650734	3.922272
25	1	0	1.245071	-4.763601	4.828300
26	1	0	2.871768	-5.006665	4.068824
27	15	0	-1.272551	-0.680735	0.216888
28	15	0	2.098371	0.112312	0.156855
29	46	0	0.318807	0.558885	-1.128025
30	6	0	-1.941065	0.378978	1.539941
31	6	0	-2.756289	-0.063933	2.582035
32	6	0	-1.668177	1.733963	1.423133
33	6	0	-3.292006	0.824438	3.515089
34	1	0	-2.968074	-1.122414	2.663134
35	6	0	-2.155680	2.681456	2.326970
36	1	0	-1.044400	2.062382	0.598438
37	6	0	-2.912406	2.188211	3.405537
38	6	0	-2.679067	-1.309356	-0.749915
39	6	0	-3.847706	-1.798038	-0.165558
40	6	0	-2.563352	-1.313160	-2.131670
41	6	0	-4.859338	-2.372964	-0.926860
42	1	0	-3.956404	-1.740595	0.907919
43	6	0	-3.566487	-1.819901	-2.967206
44	1	0	-1.668278	-0.893842	-2.576615
45	6	0	-4.660289	-2.440697	-2.330994
46	6	0	3.484639	1.272497	-0.078336
47	6	0	4.810294	0.866143	-0.011673
48	6	0	3.188893	2.595114	-0.398582
49	6	0	5.860612	1.770367	-0.190951
50	1	0	5.029976	-0.178147	0.156258
51	6	0	4.183266	3.553959	-0.571633
52	1	0	2.149744	2.864207	-0.535864
53	6	0	5.516544	3.130309	-0.355808
54	6	0	2.733411	-1.552896	-0.156452
55	6	0	2.289955	-2.206108	-1.296892
56	6	0	3.650841	-2.184714	0.685260
57	6	0	2.790571	-3.452103	-1.685405
58	1	0	1.523077	-1.729410	-1.899637
59	6	0	4.236223	-3.394940	0.325251
60	1	0	3.913383	-1.707938	1.621768
61	6	0	3.857898	-3.958514	-0.921339
62	6	0	2.090108	-4.135035	-2.884675
63	6	0	2.383298	-3.352076	-4.177647
64	1	0	1.862353	-3.816081	-5.022721
65	1	0	2.042159	-2.313578	-4.103997
66	1	0	3.453478	-3.339972	-4.407735
67	6	0	2.448506	-5.616358	-3.097016
68	1	0	2.330908	-6.194371	-2.176399
69	1	0	1.761508	-6.030890	-3.843006
70	1	0	3.461102	-5.767575	-3.472711
71	6	0	0.565066	-4.106785	-2.625976
72	1	0	0.316682	-4.662804	-1.716355
73	1	0	0.164638	-3.094339	-2.521330
74	1	0	0.043664	-4.579144	-3.465427
75	6	0	5.200701	-4.115115	1.290016
76	6	0	4.649376	-5.524865	1.582106
77	1	0	4.646067	-6.147390	0.685362
78	1	0	5.266187	-6.020383	2.340245
79	1	0	3.622952	-5.470084	1.957893
80	6	0	6.627533	-4.223642	0.719194
81	1	0	6.995663	-3.250062	0.375898
82	1	0	7.307648	-4.577614	1.502148
83	1	0	6.686476	-4.932065	-0.107773
84	6	0	5.305885	-3.368200	2.630012
85	1	0	5.767394	-2.380707	2.516155
86	1	0	4.331216	-3.241512	3.110658
87	1	0	5.939901	-3.947668	3.308371
88	8	0	4.565532	-5.047788	-1.367124
89	8	0	6.519936	4.073587	-0.361705
90	8	0	-3.321290	3.051715	4.393077
91	8	0	-5.604352	-3.118695	-3.064516
92	6	0	-5.194828	-4.419702	-3.479240
93	1	0	-6.085425	-4.919476	-3.864650
94	1	0	-4.441122	-4.376553	-4.270734
95	1	0	-4.787423	-4.992379	-2.639971
96	6	0	-3.374656	-1.606278	-4.488166
97	6	0	-4.670498	-1.710948	-5.312070
98	1	0	-4.461318	-1.373413	-6.332744
99	1	0	-5.063882	-2.724248	-5.382691
100	1	0	-5.454327	-1.065919	-4.901791
101	6	0	-2.322214	-2.594203	-5.025136
102	1	0	-2.163523	-2.430520	-6.097090
103	1	0	-1.360704	-2.452768	-4.518921
104	1	0	-2.623020	-3.636204	-4.883932
105	6	0	5.513624	-4.718214	-2.379675
106	1	0	6.038547	-5.642144	-2.628901
107	1	0	5.028111	-4.324923	-3.278108
108	1	0	6.232760	-3.974114	-2.020020
109	6	0	6.776463	4.604511	0.941955
110	1	0	7.575033	5.340017	0.828930
111	1	0	5.889592	5.089994	1.356781
112	1	0	7.094819	3.822957	1.636471
113	6	0	7.298862	1.231447	-0.379998
114	6	0	7.339129	-0.304400	-0.264574
115	1	0	7.060369	-0.647657	0.738118
116	1	0	6.688372	-0.797380	-0.994290
117	1	0	8.361127	-0.646043	-0.455203
118	6	0	7.750841	1.603699	-1.806763
119	1	0	7.786680	2.687548	-1.943252
120	1	0	8.752367	1.202350	-1.998577
121	1	0	7.068716	1.184908	-2.554856
122	6	0	8.319852	1.780714	0.631805
123	1	0	8.541756	2.834269	0.459684
124	1	0	7.972952	1.653055	1.662636
125	1	0	9.261412	1.230606	0.527977
126	6	0	3.811794	4.930592	-1.172507
127	6	0	2.291555	5.056225	-1.387697
128	1	0	1.896663	4.272781	-2.041392
129	1	0	1.738880	5.031142	-0.441432
130	1	0	2.081453	6.017524	-1.866711
131	6	0	4.240350	6.137552	-0.319524
132	1	0	3.797823	7.047836	-0.738455
133	1	0	3.889223	6.044697	0.713690
134	1	0	5.321728	6.277587	-0.316032
135	6	0	4.487061	5.021599	-2.556208
136	1	0	4.223774	5.967386	-3.043053
137	1	0	5.575979	4.974243	-2.469894
138	1	0	4.156499	4.203769	-3.205881
139	6	0	-6.150427	-2.871541	-0.239634
140	6	0	-2.841231	-0.173335	-4.730387
141	1	0	-3.497509	0.578984	-4.280211
142	1	0	-1.832601	-0.023733	-4.337593
143	1	0	-2.795965	0.016118	-5.807843
144	6	0	-7.368933	-2.111182	-0.795815
145	1	0	-8.288566	-2.516606	-0.358403
146	1	0	-7.315828	-1.050424	-0.529807
147	1	0	-7.442361	-2.197626	-1.881937
148	6	0	-6.358549	-4.386403	-0.429938
149	1	0	-5.446529	-4.946367	-0.196746
150	1	0	-7.145829	-4.734344	0.247853
151	1	0	-6.670462	-4.631056	-1.445371

152	6	0	-6.111519	-2.607821	1.275243
153	1	0	-5.982430	-1.544350	1.503713
154	1	0	-7.061592	-2.924452	1.716626
155	1	0	-5.314188	-3.170418	1.773256
156	6	0	-2.323301	3.251721	5.390675
157	1	0	-2.726658	3.973625	6.103551
158	1	0	-2.092845	2.317798	5.913074
159	1	0	-1.399522	3.649260	4.957148
160	6	0	-4.412534	0.335910	4.466902
161	6	0	-4.341686	0.822194	5.927763
162	1	0	-5.165868	0.361399	6.482955
163	1	0	-3.415504	0.517631	6.423314
164	1	0	-4.447266	1.901932	6.016247
165	6	0	-5.735366	0.845392	3.855538
166	1	0	-6.587857	0.503183	4.453523
167	1	0	-5.760205	1.939513	3.823324
168	1	0	-5.864288	0.469950	2.834532
169	6	0	-4.462870	-1.200975	4.524463
170	1	0	-4.710728	-1.652390	3.560153
171	1	0	-3.512329	-1.623066	4.871565
172	1	0	-5.241020	-1.507825	5.229928
173	6	0	-1.823452	4.160290	2.012862
174	6	0	-0.322585	4.405364	2.257565
175	1	0	-0.067434	5.442900	2.012783
176	1	0	-0.053254	4.229407	3.303782
177	1	0	0.296247	3.747267	1.637577
178	6	0	-2.637781	5.205923	2.794006
179	1	0	-2.375378	5.257663	3.850947
180	1	0	-2.433481	6.191248	2.360841
181	1	0	-3.713002	5.023846	2.712717
182	6	0	-2.123825	4.424912	0.519296
183	1	0	-1.511229	3.816490	-0.150947
184	1	0	-3.178206	4.235614	0.292908
185	1	0	-1.912404	5.473841	0.286208
186	8	0	-1.226853	1.562677	-2.298504
187	6	0	-1.300103	2.637134	-2.899859
188	1	0	1.433865	1.249767	-1.947366
189	6	0	-0.212622	3.207286	-3.659079
190	6	0	0.931788	2.681072	-4.203223
191	8	0	-0.335223	4.519577	-4.023478
192	6	0	1.547000	3.732446	-4.928263
193	1	0	1.273985	1.661679	-4.104190
194	6	0	0.738496	4.821096	-4.772059
195	1	0	2.468711	3.691676	-5.489158
196	1	0	0.802240	5.840692	-5.122205
197	6	0	-2.598739	3.407135	-2.875601
198	1	0	-2.933803	3.612556	-3.898056
199	1	0	-2.440198	4.367696	-2.372768
200	8	0	-3.546226	2.610882	-2.193930
201	6	0	-4.702341	3.197366	-1.843597
202	8	0	-4.910183	4.379842	-2.021857
203	6	0	-5.676407	2.187580	-1.253903
204	6	0	-5.885839	1.081834	-2.306128
205	1	0	-4.978504	0.488488	-2.439386
206	1	0	-6.687798	0.414095	-1.982583
207	1	0	-6.172458	1.506634	-3.274531
208	6	0	-5.078550	1.586651	0.030224
209	1	0	-4.940269	2.351116	0.802613
210	1	0	-5.763191	0.826482	0.421695
211	1	0	-4.116133	1.108823	-0.163041
212	6	0	-6.999479	2.888478	-0.944850
213	1	0	-7.702478	2.165601	-0.519071
214	1	0	-6.863508	3.698003	-0.221517
215	1	0	-7.450014	3.309195	-1.848843

TS1a(*R*)

RwB97XD SCF energy -5020.418735 a.u.
RwB97XD SCF enthalpy -5018.453084 a.u.
RwB97XD SCF free energy -5018.699158 a.u.
Three lowest frequencies (cm^{-1}) -135.3, 6.3, 10.5
Imaginary frequency (cm^{-1}) -135.3
Cartesian coordinates:

Center Number	Atomic Number	Atomic Type	Coordinates (Angstroms) X	Y	Z
1	6	0	-0.674197	1.472078	2.287861
2	6	0	-1.208636	2.560520	2.943145
3	6	0	-0.704910	3.851018	2.820374
4	6	0	0.363719	4.130121	2.004770
5	6	0	0.942332	3.038487	1.341135
6	6	0	0.460392	1.735146	1.472139
7	1	0	0.761201	5.132261	1.896920
8	1	0	1.809029	3.234537	0.722214
9	6	0	-1.303646	0.147529	2.536164
10	6	0	-2.013005	-0.631466	1.583156
11	6	0	-1.283045	-0.344922	3.823421
12	6	0	-2.612768	-1.830725	1.966711
13	6	0	-1.880877	-1.544168	4.193220
14	6	0	-2.553420	-2.319821	3.281001
15	1	0	-3.164356	-2.414897	1.239948
16	1	0	-3.032721	-3.250988	3.558900
17	8	0	-1.439178	4.694630	3.598296
18	8	0	-2.287499	2.578568	3.779456
19	8	0	-0.693980	0.219995	4.914924
20	8	0	-1.677340	-1.755099	5.525560
21	6	0	-2.184089	3.827856	4.458613
22	1	0	-3.176755	4.240174	4.628522
23	1	0	-1.631328	3.688115	5.395819
24	6	0	-1.235336	-0.491337	6.026998
25	1	0	-2.096117	0.060637	6.424077
26	1	0	-0.462805	-0.639886	6.779635
27	15	0	-2.028057	-0.109089	-0.173936
28	15	0	1.270920	0.382870	0.538212
29	46	0	0.075787	-0.421649	-1.215872
30	6	0	-2.521941	1.633310	-0.205589
31	6	0	-3.585813	2.128735	0.549880
32	6	0	-1.854859	2.472277	-1.083657
33	6	0	-3.948202	3.470382	0.485549
34	1	0	-4.128741	1.446165	1.192292
35	6	0	-2.173551	3.828556	-1.206746
36	1	0	-1.051726	2.058191	-1.685461
37	6	0	-3.158525	4.320583	-0.332089
38	6	0	-3.383569	-1.041001	-0.949170
39	6	0	-4.594602	-0.439913	-1.281627
40	6	0	-3.191086	-2.387232	-1.235684
41	6	0	-5.653348	-1.177634	-1.804801
42	1	0	-4.710702	0.622708	-1.125911
43	6	0	-4.213410	-3.191443	-1.751642
44	1	0	-2.219939	-2.826164	-1.034086
45	6	0	-5.468895	-2.575095	-1.940349
46	6	0	2.895970	1.053556	0.070526
47	6	0	4.047653	0.725758	0.773976
48	6	0	2.979725	1.914766	-1.017904
49	6	0	5.280786	1.292591	0.453251
50	1	0	3.981674	0.011767	1.582118
51	6	0	4.176903	2.535349	-1.384203
52	1	0	2.081065	2.108533	-1.592638
53	6	0	5.309951	2.255021	-0.583624
54	6	0	1.534640	-0.980693	1.703686
55	6	0	1.179211	-2.261610	1.300958
56	6	0	2.010985	-0.772028	2.995633
57	6	0	1.278812	-3.362308	2.150985
58	1	0	0.775968	-2.391816	0.304471
59	6	0	2.188535	-1.833858	3.882392
60	1	0	2.233260	0.240649	3.308407
61	6	0	1.840197	-3.131520	3.426851
62	6	0	0.757380	-4.726232	1.642551
63	6	0	1.926162	-5.580793	1.118048
64	1	0	1.541009	-6.507295	0.676670
65	1	0	2.476790	-5.043410	0.338841
66	1	0	2.631908	-5.855554	1.903150
67	6	0	-0.025078	-5.488833	2.728354
68	1	0	-0.813083	-4.856347	3.152336
69	1	0	-0.503667	-6.365739	2.279319
70	1	0	0.607378	-5.832085	3.545774
71	6	0	-0.221667	-4.528378	0.465648
72	1	0	-1.043723	-3.856076	0.734860
73	1	0	0.266054	-4.138867	-0.433118
74	1	0	-0.651798	-5.497366	0.195098
75	6	0	2.753576	-1.558009	5.293410
76	6	0	1.985041	-2.328354	6.388985
77	1	0	2.292193	-3.371764	6.457103
78	1	0	2.169168	-1.861614	7.362459
79	1	0	0.907347	-2.314367	6.201453
80	6	0	4.246050	-1.938014	5.336624
81	1	0	4.819287	-1.355718	4.607728
82	1	0	4.654370	-1.728180	6.331913
83	1	0	4.413854	-2.995198	5.127354
84	6	0	2.662335	-0.059878	5.642071
85	1	0	3.301209	0.558511	5.003184

86	1	0	1.636594	0.314256	5.571053
87	1	0	3.008290	0.086430	6.669859
88	8	0	1.966525	-4.175151	4.303466
89	8	0	6.516703	2.860648	-0.834903
90	8	0	-3.402351	5.670502	-0.263388
91	8	0	-6.563604	-3.322547	-2.303217
92	6	0	-7.192329	-3.987886	-1.210320
93	1	0	-8.118971	-4.416917	-1.596096
94	1	0	-6.567128	-4.791985	-0.811837
95	1	0	-7.420593	-3.289061	-0.399340
96	6	0	-3.854033	-4.663221	-2.069145
97	6	0	-4.801061	-5.358102	-3.063919
98	1	0	-4.362574	-6.322815	-3.341112
99	1	0	-5.790495	-5.557972	-2.655111
100	1	0	-4.920833	-4.771456	-3.979918
101	6	0	-3.790053	-5.472695	-0.760081
102	1	0	-3.439841	-6.490765	-0.964887
103	1	0	-3.095559	-5.014885	-0.048014
104	1	0	-4.766987	-5.548138	-0.274239
105	6	0	3.133286	-4.988118	4.249714
106	1	0	3.609748	-4.983824	5.235003
107	1	0	2.848970	-6.017988	4.011313
108	1	0	3.845752	-4.627339	3.502741
109	6	0	6.679543	4.182611	-0.326748
110	1	0	7.745850	4.325437	-0.138245
111	1	0	6.357951	4.931476	-1.055328
112	1	0	6.125121	4.322826	0.605941
113	6	0	6.550206	0.815050	1.191014
114	6	0	6.216467	-0.260711	2.239658
115	1	0	5.570931	0.133046	3.032447
116	1	0	5.733051	-1.140481	1.802082
117	1	0	7.144859	-0.598764	2.710256
118	6	0	7.521083	0.184662	0.174322
119	1	0	7.800916	0.894809	-0.606243
120	1	0	8.435132	-0.138958	0.685252
121	1	0	7.070901	-0.694753	-0.298541
122	6	0	7.254876	1.960213	1.942978
123	1	0	7.776200	2.637962	1.268715
124	1	0	6.546209	2.540079	2.543801
125	1	0	8.003173	1.542395	2.625413
126	6	0	4.158290	3.452400	-2.630477
127	6	0	3.094417	2.959690	-3.637257
128	1	0	3.241875	1.905704	-3.890377
129	1	0	2.071598	3.090740	-3.273562
130	1	0	3.175425	3.545294	-4.558224
131	6	0	3.765914	4.881421	-2.208515
132	1	0	3.703141	5.531225	-3.088939
133	1	0	2.788770	4.881081	-1.715730
134	1	0	4.484010	5.323558	-1.514618
135	6	0	5.493879	3.460426	-3.398828
136	1	0	5.343006	3.944907	-4.369141
137	1	0	6.287693	4.000760	-2.885659
138	1	0	5.846848	2.441049	-3.584637
139	6	0	-6.930913	-0.452146	-2.281369
140	6	0	-2.451908	-4.704449	-2.717592
141	1	0	-2.401615	-4.056101	-3.599160
142	1	0	-1.659593	-4.408450	-2.026945
143	1	0	-2.227578	-5.728109	-3.034602
144	6	0	-7.110590	-0.712000	-3.789938
145	1	0	-8.003638	-0.192397	-4.155106
146	1	0	-6.248358	-0.338545	-4.352972
147	1	0	-7.226656	-1.777314	-4.001328
148	6	0	-8.194529	-0.900411	-1.523029
149	1	0	-8.044021	-0.854516	-0.439067
150	1	0	-9.023364	-0.228627	-1.772150
151	1	0	-8.502387	-1.910669	-1.792294
152	6	0	-6.813771	1.069599	-2.082449
153	1	0	-5.954867	1.497475	-2.609822
154	1	0	-7.713928	1.550244	-2.477834
155	1	0	-6.738115	1.336966	-1.022362
156	6	0	-2.462832	6.363801	0.556001
157	1	0	-2.777044	7.409018	0.576210
158	1	0	-2.456101	5.963621	1.574634
159	1	0	-1.449393	6.301839	0.147567
160	6	0	-5.228834	3.966964	1.187827
161	6	0	-4.969446	5.108597	2.188097
162	1	0	-5.858152	5.257995	2.810435
163	1	0	-4.132861	4.868593	2.848762
164	1	0	-4.755236	6.052972	1.687840
165	6	0	-6.213625	4.452723	0.105050
166	1	0	-7.145902	4.789723	0.572287
167	1	0	-5.798868	5.287403	-0.465256
168	1	0	-6.458272	3.645331	-0.593612
169	6	0	-5.910219	2.829088	1.966225
170	1	0	-6.186634	1.988979	1.320004
171	1	0	-5.274437	2.452291	2.775310
172	1	0	-6.831304	3.209378	2.418555
173	6	0	-1.423404	4.617255	-2.305067
174	6	0	0.043362	4.815529	-1.883188
175	1	0	0.598843	5.317630	-2.683216
176	1	0	0.128182	5.425539	-0.978376
177	1	0	0.529255	3.853790	-1.686130
178	6	0	-2.040304	5.977899	-2.672860
179	1	0	-1.922662	6.732875	-1.895556
180	1	0	-1.534448	6.353661	-3.568841
181	1	0	-3.105433	5.886481	-2.903701
182	6	0	-1.444884	3.781852	-3.606720
183	1	0	-0.926012	2.824575	-3.511453
184	1	0	-2.472881	3.579436	-3.926148
185	1	0	-0.945216	4.339306	-4.405761
186	8	0	-0.519832	-1.364744	-3.076545
187	6	0	0.747809	-1.462591	-3.186837
188	1	0	1.553188	-0.588226	-1.775985
189	6	0	1.384933	-2.764474	-2.873061
190	6	0	0.991416	-4.034134	-3.165129
191	8	0	2.621385	-2.771787	-2.309269
192	6	0	2.063044	-4.884555	-2.750719
193	1	0	0.069757	-4.319437	-3.649245
194	6	0	3.016973	-4.065959	-2.236774
195	1	0	2.110224	-5.961285	-2.821938
196	1	0	3.982208	-4.237475	-1.785439
197	6	0	1.430001	-0.534593	-4.187570
198	1	0	1.023890	0.471693	-4.088710
199	1	0	1.214047	-0.929779	-5.185133
200	8	0	2.829533	-0.444844	-3.977061
201	6	0	3.621569	-1.347413	-4.592988
202	8	0	3.180269	-2.197797	-5.336169
203	6	0	5.095833	-1.123997	-4.292313
204	6	0	5.308697	-0.612850	-2.861866
205	1	0	4.931532	-1.325997	-2.121862
206	1	0	6.379985	-0.472168	-2.688619
207	1	0	4.812554	0.345029	-2.698596
208	6	0	5.840942	-2.445696	-4.501696
209	1	0	5.717863	-2.819560	-5.521367
210	1	0	6.909058	-2.295181	-4.317725
211	1	0	5.479624	-3.214060	-3.809999
212	6	0	5.588844	-0.070815	-5.305192
213	1	0	6.654669	0.117380	-5.141608
214	1	0	5.455766	-0.421093	-6.333740
215	1	0	5.052641	0.876034	-5.188030

4a(*R*)

RwB97XD SCF energy -5020.433579 a.u.
RwB97XD SCF enthalpy -5018.463909 a.u.
RwB97XD SCF free energy -5018.70027 a.u.
Three lowest frequencies (cm^{-1}) -7.1, 17.3, 24.4

Standard orientation:

Center Number	Atomic Number	Atomic Type	Coordinates (Angstroms) X	Y	Z
1	6	0	-1.406564	2.616971	1.052420
2	6	0	-2.178104	3.748234	0.905925
3	6	0	-1.807085	4.821034	0.099030
4	6	0	-0.644064	4.810385	-0.631300
5	6	0	0.165557	3.672188	-0.499016
6	6	0	-0.187864	2.601546	0.319477
7	1	0	-0.352335	5.643243	-1.259847
8	1	0	1.103653	3.651152	-1.040622
9	6	0	-1.832921	1.571311	2.019890
10	6	0	-2.127740	0.209775	1.727572
11	6	0	-1.943667	1.936856	3.344329
12	6	0	-2.471928	-0.672230	2.751454
13	6	0	-2.282476	1.045644	4.357054
14	6	0	-2.547230	-0.275659	4.093998
15	1	0	-2.703816	-1.704963	2.522635
16	1	0	-2.811908	-0.976616	4.876379
17	8	0	-2.757322	5.792258	0.182743
18	8	0	-3.380146	4.019766	1.489745
19	8	0	-1.701982	3.163203	3.883419

20	8	0	-2.268630	1.692577	5.552144
21	6	0	-3.544571	5.427771	1.319897
22	1	0	-4.591652	5.657041	1.132289
23	1	0	-3.161004	5.946587	2.206676
24	6	0	-2.163751	3.082952	5.232944
25	1	0	-3.152994	3.548318	5.307131
26	1	0	-1.439126	3.555597	5.895772
27	15	0	-1.962885	-0.420622	0.022727
28	15	0	0.860328	1.119829	0.403644
29	46	0	0.168295	-0.786410	-0.626715
30	6	0	-2.744712	0.795064	-1.060577
31	6	0	-4.012307	1.298379	-0.763720
32	6	0	-2.103507	1.181786	-2.225640
33	6	0	-4.623226	2.232876	-1.591264
34	1	0	-4.517474	0.944037	0.126042
35	6	0	-2.657931	2.122698	-3.099074
36	1	0	-1.133148	0.751192	-2.450331
37	6	0	-3.874982	2.706451	-2.700886
38	6	0	-2.924771	-1.951523	-0.068988
39	6	0	-4.135600	-2.008019	-0.754485
40	6	0	-2.427942	-3.096570	0.540880
41	6	0	-4.913377	-3.162446	-0.751398
42	1	0	-4.476434	-1.135944	-1.293419
43	6	0	-3.155752	-4.288663	0.582400
44	1	0	-1.449261	-3.054742	1.005084
45	6	0	-4.443948	-4.263361	0.006765
46	6	0	2.474395	1.482438	-0.342959
47	6	0	3.633157	1.380169	0.421765
48	6	0	2.575930	1.683971	-1.714616
49	6	0	4.893573	1.543292	-0.143386
50	1	0	3.546629	1.160372	1.475373
51	6	0	3.806435	1.886806	-2.346138
52	1	0	1.668101	1.686184	-2.307054
53	6	0	4.951938	1.885486	-1.517238
54	6	0	1.216194	0.761073	2.137191
55	6	0	1.349654	-0.570594	2.494966
56	6	0	1.471016	1.760744	3.075843
57	6	0	1.818976	-0.958662	3.752545
58	1	0	1.093020	-1.333882	1.766698
59	6	0	1.971864	1.443939	4.335176
60	1	0	1.291993	2.793410	2.801864
61	6	0	2.241532	0.076335	4.605325
62	6	0	1.789767	-2.474154	4.052906
63	6	0	2.783378	-3.198426	3.127056
64	1	0	2.742048	-4.279423	3.300555
65	1	0	2.544317	-3.022053	2.073423
66	1	0	3.812680	-2.866888	3.300245
67	6	0	2.070149	-2.866943	5.512607
68	1	0	1.432673	-2.316591	6.209819
69	1	0	1.851464	-3.934133	5.628453
70	1	0	3.110259	-2.720408	5.806006
71	6	0	0.364917	-2.987642	3.736653
72	1	0	-0.381247	-2.482562	4.359459
73	1	0	0.088625	-2.840581	2.688618
74	1	0	0.309383	-4.062207	3.939921
75	6	0	2.144387	2.539494	5.407159
76	6	0	1.295668	2.154909	6.635358
77	1	0	1.664224	1.240368	7.104642
78	1	0	1.328269	2.956687	7.381515
79	1	0	0.250266	1.993070	6.353351
80	6	0	3.612488	2.732986	5.830828
81	1	0	4.259942	2.886165	4.960549
82	1	0	3.693204	3.622715	6.465214
83	1	0	3.991284	1.886580	6.403925
84	6	0	1.638780	3.898689	4.895895
85	1	0	2.245832	4.277659	4.066479
86	1	0	0.595844	3.855576	4.568153
87	1	0	1.701988	4.630547	5.707203
88	8	0	2.938548	-0.222986	5.749706
89	8	0	6.184370	2.188150	-2.040310
90	8	0	-4.390943	3.763840	-3.407234
91	8	0	-5.284443	-5.340634	0.140524
92	6	0	-5.963605	-5.380796	1.394054
93	1	0	-6.707848	-6.176051	1.323362
94	1	0	-5.279275	-5.603572	2.217753
95	1	0	-6.464240	-4.429727	1.602546
96	6	0	-2.459074	-5.501192	1.244417
97	6	0	-3.058004	-6.870743	0.877738
98	1	0	-2.384605	-7.650795	1.248794
99	1	0	-4.036045	-7.051706	1.322482
100	1	0	-3.145204	-6.993970	-0.205968
101	6	0	-2.465131	-5.320143	2.773842
102	1	0	-1.914078	-6.137520	3.252639
103	1	0	-1.985576	-4.377893	3.058462
104	1	0	-3.479881	-5.315143	3.182681
105	6	0	4.315972	-0.494057	5.493869
106	1	0	4.782111	-0.682652	6.462674
107	1	0	4.441280	-1.372985	4.853682
108	1	0	4.804546	0.361730	5.015459
109	6	0	6.424663	3.589707	-2.156045
110	1	0	7.449328	3.703135	-2.514179
111	1	0	5.740817	4.057608	-2.869659
112	1	0	6.314869	4.091395	-1.189488
113	6	0	6.147095	1.245030	0.709629
114	6	0	5.765952	0.838068	2.146063
115	1	0	5.266698	1.651221	2.684976
116	1	0	5.120443	-0.046637	2.176191
117	1	0	6.679756	0.591914	2.695908
118	6	0	6.887926	0.053089	0.074486
119	1	0	7.185028	0.268554	-0.954242
120	1	0	7.791837	-0.177415	0.649797
121	1	0	6.252760	-0.839098	0.073093
122	6	0	7.104475	2.446009	0.821683
123	1	0	7.621949	2.644762	-0.116820
124	1	0	6.574926	3.352601	1.133472
125	1	0	7.868066	2.230219	1.576874
126	6	0	3.770473	2.076978	-3.883716
127	6	0	2.782765	1.054363	-4.493021
128	1	0	3.040186	0.032418	-4.200767
129	1	0	1.742707	1.242496	-4.213992
130	1	0	2.826995	1.117128	-5.585191
131	6	0	3.251834	3.492305	-4.203837
132	1	0	3.144148	3.618696	-5.287039
133	1	0	2.272276	3.665148	-3.745826
134	1	0	3.932308	4.268761	-3.841666
135	6	0	5.113416	1.845391	-4.599785
136	1	0	4.928141	1.814343	-5.678821
137	1	0	5.841889	2.636587	-4.423450
138	1	0	5.564926	0.892823	-4.311906
139	6	0	-6.189149	-3.226352	-1.618772
140	6	0	-0.987842	-5.543756	0.770275
141	1	0	-0.930817	-5.566874	-0.322717
142	1	0	-0.395187	-4.697154	1.125816
143	1	0	-0.507074	-6.448651	1.155848
144	6	0	-6.009743	-4.339669	-2.669436
145	1	0	-6.897498	-4.397340	-3.309478
146	1	0	-5.145046	-4.133254	-3.309467
147	1	0	-5.865767	-5.315341	-2.199662
148	6	0	-7.462498	-3.492230	-0.793255
149	1	0	-7.541941	-2.797682	0.050084
150	1	0	-8.343050	-3.344594	-1.427973
151	1	0	-7.502710	-4.512680	-0.412718
152	6	0	-6.417646	-1.904383	-2.373898
153	1	0	-5.581450	-1.644312	-3.031293
154	1	0	-7.307452	-2.004895	-3.002890
155	1	0	-6.594028	-1.067440	-1.688774
156	6	0	-3.789668	5.009226	-3.054246
157	1	0	-4.272666	5.773692	-3.665481
158	1	0	-3.944222	5.234972	-1.994714
159	1	0	-2.714224	5.009075	-3.258033
160	6	0	-6.094081	2.632537	-1.354526
161	6	0	-6.282090	4.143926	-1.133526
162	1	0	-7.295850	4.336510	-0.766237
163	1	0	-5.578434	4.516148	-0.384197
164	1	0	-6.149421	4.713783	-2.052934
165	6	0	-6.914519	2.184877	-2.581041
166	1	0	-7.972279	2.429535	-2.432783
167	1	0	-6.574397	2.682120	-3.492731
168	1	0	-6.836346	1.102116	-2.730337
169	6	0	-6.671057	1.921476	-0.119088
170	1	0	-6.621788	0.830652	-0.204782
171	1	0	-6.157796	2.221425	0.801392
172	1	0	-7.725806	2.192273	-0.011847
173	6	0	-1.879275	2.390154	-4.409200
174	6	0	-0.587626	3.164065	-4.090063
175	1	0	0.005831	3.295699	-5.001693
176	1	0	-0.797149	4.154923	-3.675398
177	1	0	0.025026	2.620000	-3.364267
178	6	0	-2.663691	3.137656	-5.501193
179	1	0	-2.838317	4.187985	-5.267674
180	1	0	-2.078345	3.106761	-6.426725
181	1	0	-3.628339	2.663213	-5.701353
182	6	0	-1.492070	1.026321	-5.028664
183	1	0	-0.838274	0.430347	-4.386128

184	1	0	-2.384312	0.429443	-5.247140
185	1	0	-0.956297	1.191573	-5.969103
186	8	0	0.077394	-2.604384	-1.566229
187	6	0	1.427547	-2.476625	-1.789560
188	6	0	2.286065	-3.406299	-0.979217
189	6	0	2.175728	-4.715770	-0.634755
190	8	0	3.497966	-2.921241	-0.587569
191	6	0	3.406933	-5.065789	0.009322
192	1	0	1.336508	-5.361448	-0.846583
193	6	0	4.168758	-3.943624	0.007507
194	1	0	3.680313	-6.026000	0.422688
195	1	0	5.149944	-3.707688	0.390251
196	6	0	1.732926	-2.540187	-3.295443
197	1	0	1.141670	-1.782822	-3.812608
198	1	0	1.490239	-3.534493	-3.675555
199	8	0	3.100627	-2.222211	-3.546199
200	6	0	3.993942	-3.218028	-3.685322
201	8	0	3.684777	-4.390824	-3.668384
202	6	0	5.401858	-2.677742	-3.902690
203	6	0	5.744937	-1.653157	-2.810214
204	1	0	5.752036	-2.127004	-1.824948
205	1	0	6.742709	-1.243856	-2.996511
206	1	0	5.032881	-0.824265	-2.787072
207	6	0	6.389661	-3.844448	-3.847891
208	1	0	6.185740	-4.581073	-4.630150
209	1	0	7.407956	-3.468542	-3.987815
210	1	0	6.340757	-4.352814	-2.879536
211	6	0	5.438116	-2.010753	-5.290482
212	1	0	6.449041	-1.640779	-5.489016
213	1	0	5.175599	-2.725377	-6.077576
214	1	0	4.747430	-1.164782	-5.346997
215	1	0	1.813074	-1.423062	-1.484481

5a(*R*)

RwB97XD SCF energy -5021.616218 a.u.
RwB97XD SCF enthalpy -5019.628203 a.u.
RwB97XD SCF free energy -5019.875590 a.u.
Three lowest frequencies (cm^{-1}) 8.7, 13.2,19.6
Cartesian coordinates:

Center Number	Atomic Number	Atomic Type	Coordinates (Angstroms) X	Y	Z
1	6	0	1.629149	-0.730629	2.430206
2	6	0	1.586743	-0.636471	3.804112
3	6	0	1.901116	0.531787	4.494134
4	6	0	2.243236	1.688712	3.840081
5	6	0	2.269006	1.629190	2.439045
6	6	0	1.977554	0.460343	1.738715
7	1	0	2.488368	2.599408	4.373043
8	1	0	2.541833	2.528286	1.899486
9	6	0	1.439589	-2.056892	1.788439
10	6	0	0.426349	-2.424371	0.859106
11	6	0	2.338802	-3.046658	2.121288
12	6	0	0.389373	-3.718610	0.340822
13	6	0	2.298871	-4.329360	1.586142
14	6	0	1.330816	-4.699394	0.685765
15	1	0	-0.397670	-4.006332	-0.343683
16	1	0	1.283236	-5.697774	0.267880
17	8	0	1.810070	0.304192	5.831279
18	8	0	1.263266	-1.613602	4.697363
19	8	0	3.400155	-2.943257	2.968353
20	8	0	3.331800	-5.058843	2.086835
21	6	0	1.699861	-1.115980	5.962943
22	1	0	0.962950	-1.354868	6.727707
23	1	0	2.685519	-1.535355	6.196076
24	6	0	3.845521	-4.283920	3.174426
25	1	0	3.430911	-4.664182	4.115330
26	1	0	4.934568	-4.313268	3.164911
27	15	0	-0.794488	-1.177753	0.310946
28	15	0	1.994135	0.481892	-0.081736
29	46	0	0.011006	0.261898	-1.251231
30	6	0	-1.353113	-0.360072	1.822763
31	6	0	-1.839628	-1.155165	2.861026
32	6	0	-1.372070	1.020742	1.939125
33	6	0	-2.295677	-0.591052	4.044674
34	1	0	-1.869577	-2.229110	2.724216
35	6	0	-1.808625	1.655321	3.108331
36	1	0	-1.021843	1.612147	1.101462
37	6	0	-2.166790	0.814956	4.182388
38	6	0	-2.221809	-2.057840	-0.372448
39	6	0	-3.489919	-1.894068	0.177802
40	6	0	-2.080472	-2.776842	-1.552806
41	6	0	-4.599230	-2.548570	-0.350605
42	1	0	-3.612484	-1.244823	1.032010
43	6	0	-3.152001	-3.446067	-2.145742
44	1	0	-1.114136	-2.794243	-2.043975
45	6	0	-4.381587	-3.408887	-1.452874
46	6	0	2.667216	2.102910	-0.532619
47	6	0	4.007340	2.275598	-0.856017
48	6	0	1.793540	3.180662	-0.609369
49	6	0	4.511046	3.529269	-1.195609
50	1	0	4.661638	1.414712	-0.841695
51	6	0	2.221488	4.459871	-0.970986
52	1	0	0.741167	3.009779	-0.403479
53	6	0	3.611364	4.624689	-1.173418
54	6	0	3.151451	-0.786422	-0.645962
55	6	0	2.815736	-1.498737	-1.787825
56	6	0	4.338525	-1.076820	0.026896
57	6	0	3.667768	-2.455868	-2.344897
58	1	0	1.853612	-1.308030	-2.253284
59	6	0	5.252883	-1.984401	-0.500334
60	1	0	4.542147	-0.576458	0.965968
61	6	0	4.935505	-2.584748	-1.746800
62	6	0	3.104501	-3.278178	-3.528296
63	6	0	2.969747	-2.375786	-4.768444
64	1	0	2.542532	-2.943517	-5.602438
65	1	0	2.309778	-1.524023	-4.570076
66	1	0	3.938658	-1.981688	-5.090647
67	6	0	3.913698	-4.535205	-3.891388
68	1	0	4.104786	-5.157131	-3.012320
69	1	0	3.326194	-5.129539	-4.599497
70	1	0	4.867943	-4.314028	-4.370186
71	6	0	1.695882	-3.785646	-3.136410
72	1	0	1.744576	-4.421779	-2.246551
73	1	0	0.989463	-2.974977	-2.937343
74	1	0	1.282735	-4.379587	-3.958270
75	6	0	6.516306	-2.371040	0.294881
76	6	0	6.522342	-3.898263	0.505567
77	1	0	6.635941	-4.432233	-0.439455
78	1	0	7.351884	-4.183734	1.161942
79	1	0	5.588863	-4.230535	0.970166
80	6	0	7.810778	-1.937711	-0.417600
81	1	0	7.776727	-0.876594	-0.689216
82	1	0	8.662640	-2.082881	0.255956
83	1	0	8.001636	-2.523158	-1.317385
84	6	0	6.521183	-1.710405	1.683623
85	1	0	6.598510	-0.619318	1.620428
86	1	0	5.628440	-1.962602	2.264737
87	1	0	7.393567	-2.065493	2.241031
88	8	0	5.913127	-3.327526	-2.360602
89	8	0	4.139338	5.869258	-1.409555
90	8	0	-2.438697	1.344585	5.418315
91	8	0	-5.432357	-4.188403	-1.868325
92	6	0	-5.324103	-5.547141	-1.451280
93	1	0	-6.228512	-6.051659	-1.795690
94	1	0	-4.448381	-6.037244	-1.888177
95	1	0	-5.256590	-5.621596	-0.360594
96	6	0	-2.883877	-4.089940	-3.527526
97	6	0	-4.142894	-4.492129	-4.315482
98	1	0	-3.840170	-4.759448	-5.333728
99	1	0	-4.658117	-5.356641	-3.896606
100	1	0	-4.855772	-3.665514	-4.385578
101	6	0	-1.970705	-5.317239	-3.355006
102	1	0	-1.724262	-5.745333	-4.333190
103	1	0	-1.031119	-5.046399	-2.860578
104	1	0	-2.451099	-6.098910	-2.757777
105	6	0	6.552218	-2.630567	-3.428428

106	1	0	7.344924	-3.283352	-3.797910
107	1	0	5.854950	-2.412852	-4.243249
108	1	0	6.986419	-1.687509	-3.079087
109	6	0	4.330233	6.668845	-0.244405
110	1	0	5.018675	7.468722	-0.523513
111	1	0	3.392791	7.115730	0.098188
112	1	0	4.762779	6.082788	0.572649
113	6	0	5.980696	3.660122	-1.651066
114	6	0	6.692676	2.294002	-1.646971
115	1	0	6.774328	1.874199	-0.637996
116	1	0	6.195667	1.559521	-2.289631
117	1	0	7.709247	2.426128	-2.030222
118	6	0	6.012593	4.191264	-3.097418
119	1	0	5.535659	5.170186	-3.177269
120	1	0	7.050181	4.288705	-3.436179
121	1	0	5.500463	3.500950	-3.776650
122	6	0	6.798382	4.591107	-0.735377
123	1	0	6.542691	5.640916	-0.878599
124	1	0	6.655420	4.336867	0.320187
125	1	0	7.864456	4.480846	-0.962273
126	6	0	1.128831	5.543678	-1.143879
127	6	0	-0.134134	4.903043	-1.768945
128	1	0	0.097389	4.370953	-2.698223
129	1	0	-0.635016	4.212912	-1.086224
130	1	0	-0.858905	5.688999	-2.002952
131	6	0	0.722549	6.098558	0.234161
132	1	0	-0.072894	6.844144	0.121486
133	1	0	0.343190	5.297839	0.877705
134	1	0	1.558670	6.574644	0.753647
135	6	0	1.531565	6.694220	-2.084112
136	1	0	0.642972	7.298072	-2.295878
137	1	0	2.282066	7.360903	-1.661594
138	1	0	1.911304	6.313836	-3.037837
139	6	0	-6.004550	-2.257393	0.216053
140	6	0	-2.148638	-3.054550	-4.412356
141	1	0	-2.730845	-2.132291	-4.508494
142	1	0	-1.158452	-2.791118	-4.031522
143	1	0	-2.003833	-3.474256	-5.413365
144	6	0	-6.894521	-1.696415	-0.911329
145	1	0	-7.904729	-1.512579	-0.528043
146	1	0	-6.496595	-0.745430	-1.280741
147	1	0	-6.971406	-2.391744	-1.750226
148	6	0	-6.658471	-3.510727	0.826375
149	1	0	-5.986946	-3.999864	1.540538
150	1	0	-7.566840	-3.222718	1.367152
151	1	0	-6.947422	-4.235179	0.064558
152	6	0	-5.949672	-1.189178	1.321883
153	1	0	-5.516502	-0.247990	0.967174
154	1	0	-6.969276	-0.974942	1.657199
155	1	0	-5.383100	-1.527927	2.196616
156	6	0	-1.269661	1.633250	6.181317
157	1	0	-1.603973	1.842014	7.199398
158	1	0	-0.581688	0.782588	6.187253
159	1	0	-0.741579	2.509614	5.793707
160	6	0	-2.993271	-1.471243	5.103147
161	6	0	-2.199433	-1.547212	6.418767
162	1	0	-2.654802	-2.292332	7.080541
163	1	0	-1.167627	-1.857169	6.226744
164	1	0	-2.188024	-0.597490	6.953780
165	6	0	-4.404841	-0.910788	5.367516
166	1	0	-4.929003	-1.549858	6.086781
167	1	0	-4.368009	0.101370	5.773942
168	1	0	-4.993658	-0.891202	4.443682
169	6	0	-3.164609	-2.913411	4.595592
170	1	0	-3.720541	-2.957437	3.652428
171	1	0	-2.203770	-3.420852	4.456827
172	1	0	-3.730933	-3.484971	5.337356
173	6	0	-1.892391	3.201861	3.094884
174	6	0	-0.491139	3.816262	3.254467
175	1	0	-0.557990	4.909163	3.208468
176	1	0	-0.028679	3.547831	4.207712
177	1	0	0.177571	3.483217	2.454032
178	6	0	-2.835955	3.791104	4.159647
179	1	0	-2.445017	3.710229	5.173573
180	1	0	-2.975389	4.856649	3.948305
181	1	0	-3.819889	3.313326	4.131642
182	6	0	-2.447851	3.665212	1.728176
183	1	0	-1.745079	3.490944	0.909635
184	1	0	-3.394186	3.170181	1.485219
185	1	0	-2.626093	4.744346	1.761383
186	8	0	-1.684057	0.068518	-2.373923
187	6	0	-2.362712	1.254789	-2.631789
188	6	0	-3.663406	0.939981	-3.310223
189	6	0	-4.483147	-0.142503	-3.278704
190	8	0	-4.243215	1.942631	-4.027473
191	6	0	-5.654304	0.217650	-4.024198
192	1	0	-4.283650	-1.074221	-2.771685
193	6	0	-5.457062	1.490574	-4.447695
194	1	0	-6.524620	-0.394570	-4.214389
195	1	0	-6.039021	2.177573	-5.043243
196	6	0	-2.616669	1.992369	-1.305704
197	1	0	-1.655069	2.260745	-0.863517
198	1	0	-3.171547	1.348731	-0.619682
199	8	0	-3.301175	3.229840	-1.508879
200	6	0	-4.627728	3.264345	-1.312932
201	8	0	-5.267093	2.309175	-0.918616
202	6	0	-5.207914	4.645791	-1.592901
203	6	0	-4.651611	5.209443	-2.908757
204	1	0	-4.898112	4.560142	-3.754318
205	1	0	-5.094836	6.193114	-3.094009
206	1	0	-3.565816	5.327618	-2.870900
207	6	0	-6.732048	4.532004	-1.672555
208	1	0	-7.156342	4.161870	-0.735349
209	1	0	-7.164490	5.516086	-1.878314
210	1	0	-7.032710	3.849951	-2.474603
211	6	0	-4.800407	5.556937	-0.418950
212	1	0	-5.237221	6.550654	-0.561704
213	1	0	-5.164690	5.159910	0.534343
214	1	0	-3.713274	5.665964	-0.358920
215	1	0	-1.794616	1.945137	-3.284883
216	1	0	0.470257	1.673443	-2.495507
217	1	0	0.758972	1.043643	-2.840812

TS2a(R)

RwB97XD SCF energy	-5021.603774 a.u.
RwB97XD SCF enthalpy	-5019.618339 a.u.
RwB97XD SCF free energy	-5019.864415 a.u.
Three lowest frequencies (cm^{-1})	-1042, 8.2, 14.9
Imaginary frequency (cm^{-1})	-1042

Cartesian coordinates:

Center Number	Atomic Number	Atomic Type	Coordinates (Angstroms) X	Y	Z
1	6	0	1.443941	0.043852	2.567073
2	6	0	1.377158	0.559004	3.843141
3	6	0	1.788669	1.850475	4.163179
4	6	0	2.249337	2.721134	3.207029
5	6	0	2.313152	2.229546	1.895663
6	6	0	1.935879	0.927899	1.568476
7	1	0	2.565245	3.728153	3.451377
8	1	0	2.688318	2.892583	1.125733
9	6	0	1.111266	-1.387071	2.348771
10	6	0	0.096289	-1.901142	1.495906
11	6	0	1.853420	-2.324553	3.034896
12	6	0	-0.085182	-3.277833	1.371607
13	6	0	1.663223	-3.695641	2.902043
14	6	0	0.701414	-4.208492	2.066932
15	1	0	-0.867550	-3.664105	0.730170
16	1	0	0.544705	-5.274837	1.956013
17	8	0	1.648518	2.049681	5.499838
18	8	0	0.932943	-0.062923	4.972139
19	8	0	2.883416	-2.093648	3.895750
20	8	0	2.562835	-4.352944	3.684311
21	6	0	1.407385	0.749301	6.045895
22	1	0	0.648267	0.815049	6.823494
23	1	0	2.350015	0.336628	6.424649

24	6	0	3.118788	-3.347575	4.536628
25	1	0	2.600433	-3.364527	5.502627
26	1	0	4.190947	-3.507163	4.644184
27	15	0	-0.917162	-0.749814	0.497070
28	15	0	2.059232	0.379939	-0.163350
29	46	0	0.165250	0.032258	-1.376295
30	6	0	-1.448068	0.555798	1.629497
31	6	0	-2.005787	0.212331	2.862368
32	6	0	-1.346976	1.889196	1.263840
33	6	0	-2.403712	1.186997	3.767819
34	1	0	-2.127915	-0.835904	3.105873
35	6	0	-1.718953	2.922182	2.133841
36	1	0	-0.949679	2.131105	0.282923
37	6	0	-2.143828	2.537357	3.419094
38	6	0	-2.397517	-1.668519	-0.007515
39	6	0	-3.655175	-1.400019	0.529636
40	6	0	-2.267175	-2.647105	-0.985118
41	6	0	-4.765655	-2.167625	0.187153
42	1	0	-3.765341	-0.581328	1.225601
43	6	0	-3.341771	-3.441960	-1.391567
44	1	0	-1.295402	-2.798270	-1.441050
45	6	0	-4.563411	-3.243020	-0.714393
46	6	0	2.986418	1.675748	-1.025351
47	6	0	4.334897	1.531724	-1.318252
48	6	0	2.299229	2.801352	-1.462511
49	6	0	5.031416	2.516312	-2.017310
50	1	0	4.843730	0.632301	-1.002361
51	6	0	2.927158	3.824057	-2.173332
52	1	0	1.237122	2.865688	-1.257077
53	6	0	4.321218	3.684227	-2.391150
54	6	0	3.055925	-1.126587	-0.203711
55	6	0	2.714206	-2.116211	-1.112733
56	6	0	4.157802	-1.304917	0.633670
57	6	0	3.483401	-3.271236	-1.276724
58	1	0	1.817512	-1.986865	-1.709930
59	6	0	4.994886	-2.406664	0.485737
60	1	0	4.359638	-0.561340	1.394837
61	6	0	4.687549	-3.327155	-0.550121
62	6	0	2.906565	-4.357495	-2.214540
63	6	0	2.968358	-3.869313	-3.673311
64	1	0	2.531356	-4.621627	-4.339422
65	1	0	2.406787	-2.937764	-3.804652
66	1	0	3.998772	-3.690188	-3.996085
67	6	0	3.579413	-5.736742	-2.104987
68	1	0	3.627755	-6.079037	-1.067207
69	1	0	2.976948	-6.457880	-2.667877
70	1	0	4.585811	-5.762446	-2.523124
71	6	0	1.422577	-4.583684	-1.838814
72	1	0	1.327538	-4.910317	-0.797770
73	1	0	0.806490	-3.690372	-1.973801
74	1	0	1.001669	-5.363759	-2.481619
75	6	0	6.155818	-2.641831	1.473655
76	6	0	5.965245	-4.019410	2.138972
77	1	0	6.073516	-4.831661	1.417893
78	1	0	6.711255	-4.161576	2.928809
79	1	0	4.969948	-4.097554	2.587571
80	6	0	7.533287	-2.578485	0.787501
81	1	0	7.639997	-1.660984	0.197831
82	1	0	8.320234	-2.578954	1.549939
83	1	0	7.709321	-3.434639	0.135955
84	6	0	6.157468	-1.583521	2.589908
85	1	0	6.359176	-0.577947	2.203838
86	1	0	5.211372	-1.562344	3.139820
87	1	0	6.951986	-1.823655	3.303284
88	8	0	5.608373	-4.309185	-0.818050
89	8	0	5.030976	4.653628	-3.054320
90	8	0	-2.334464	3.488428	4.390495
91	8	0	-5.627556	-4.080890	-0.942522
92	6	0	-5.556738	-5.312639	-0.226955
93	1	0	-6.533454	-5.789423	-0.329285
94	1	0	-4.791429	-5.977370	-0.637532
95	1	0	-5.341659	-5.143255	0.832840
96	6	0	-3.064917	-4.443274	-2.539364
97	6	0	-4.318897	-4.956578	-3.269136
98	1	0	-3.996763	-5.517398	-4.153232
99	1	0	-4.932032	-5.627465	-2.669012
100	1	0	-4.944350	-4.126862	-3.610795
101	6	0	-2.256818	-5.631627	-1.984770
102	1	0	-1.998095	-6.323157	-2.794732
103	1	0	-1.324631	-5.288498	-1.523260
104	1	0	-2.817293	-6.190947	-1.229731
105	6	0	6.398521	-4.034324	-1.973583

106	1	0	7.152606	-4.821536	-2.029400
107	1	0	5.796958	-4.044547	-2.887766
108	1	0	6.891991	-3.060027	-1.890588
109	6	0	5.398576	5.821090	-2.322709
110	1	0	6.392161	6.120780	-2.664279
111	1	0	4.707460	6.643644	-2.526341
112	1	0	5.430098	5.626019	-1.246693
113	6	0	6.506886	2.264332	-2.397570
114	6	0	6.986484	0.884544	-1.907093
115	1	0	6.967714	0.806267	-0.814269
116	1	0	6.395109	0.062844	-2.325083
117	1	0	8.022328	0.738194	-2.228381
118	6	0	6.653551	2.278932	-3.931298
119	1	0	6.363653	3.241712	-4.355398
120	1	0	7.696052	2.082996	-4.206511
121	1	0	6.032817	1.499019	-4.386333
122	6	0	7.445439	3.309511	-1.766498
123	1	0	7.339895	4.291135	-2.225723
124	1	0	7.263006	3.405814	-0.690678
125	1	0	8.486216	2.995845	-1.902863
126	6	0	2.042522	4.991772	-2.671242
127	6	0	0.576232	4.527722	-2.835585
128	1	0	0.499822	3.635127	-3.466912
129	1	0	0.085364	4.322805	-1.878954
130	1	0	0.003311	5.328060	-3.313819
131	6	0	2.035289	6.124214	-1.626349
132	1	0	1.362428	6.926568	-1.949679
133	1	0	1.676310	5.749764	-0.662297
134	1	0	3.022875	6.559340	-1.466761
135	6	0	2.474967	5.517937	-4.052522
136	1	0	1.738901	6.249202	-4.402263
137	1	0	3.448665	6.004017	-4.047803
138	1	0	2.509800	4.702244	-4.783096
139	6	0	-6.159412	-1.786024	0.735858
140	6	0	-2.209717	-3.747762	-3.624159
141	1	0	-2.705960	-2.850469	-4.005796
142	1	0	-1.214277	-3.466134	-3.272109
143	1	0	-2.065445	-4.437982	-4.461726
144	6	0	-7.096063	-1.451808	-0.442003
145	1	0	-8.099023	-1.223890	-0.063225
146	1	0	-6.738302	-0.571841	-0.986841
147	1	0	-7.178949	-2.287676	-1.140296
148	6	0	-6.783572	-2.908045	1.587677
149	1	0	-6.078256	-3.273444	2.342039
150	1	0	-7.661250	-2.517037	2.114014
151	1	0	-7.116850	-3.750396	0.981626
152	6	0	-6.086834	-0.536771	1.632194
153	1	0	-5.682784	0.332624	1.105648
154	1	0	-7.099003	-0.280563	1.961027
155	1	0	-5.485425	-0.713115	2.530942
156	6	0	-1.113775	3.879894	5.016590
157	1	0	-1.373611	4.628718	5.767260
158	1	0	-0.629279	3.026808	5.500397
159	1	0	-0.413047	4.315462	4.296340
160	6	0	-3.192299	0.796038	5.034458
161	6	0	-2.479815	1.194850	6.338201
162	1	0	-3.003976	0.749342	7.190827
163	1	0	-1.452202	0.819716	6.345396
164	1	0	-2.464437	2.274156	6.488696
165	6	0	-4.574712	1.475284	4.967247
166	1	0	-5.171428	1.194449	5.842455
167	1	0	-4.485925	2.564110	4.951349
168	1	0	-5.119466	1.160053	4.070290
169	6	0	-3.422535	-0.723139	5.098083
170	1	0	-3.957270	-1.100476	4.220009
171	1	0	-2.481971	-1.276395	5.197525
172	1	0	-4.033663	-0.952303	5.976567
173	6	0	-1.652359	4.365757	1.577030
174	6	0	-0.186060	4.758399	1.326808
175	1	0	-0.141454	5.760481	0.885781
176	1	0	0.391517	4.771010	2.256289
177	1	0	0.303326	4.063389	0.635572
178	6	0	-2.317817	5.449560	2.443517
179	1	0	-1.769998	5.669595	3.359967
180	1	0	-2.351539	6.375490	1.858816
181	1	0	-3.344594	5.183561	2.707721
182	6	0	-2.406373	4.395802	0.229120
183	1	0	-1.973365	3.724303	-0.514250
184	1	0	-3.456058	4.120675	0.369556
185	1	0	-2.369419	5.407919	-0.187343
186	8	0	-1.183642	-0.273298	-3.054521
187	6	0	-2.226367	0.632800	-3.356115

188	6	0	-3.467677	-0.128679	-3.689747
189	6	0	-4.519180	-0.572559	-2.952593
190	8	0	-3.606044	-0.537373	-4.983640
191	6	0	-5.366733	-1.300768	-3.847024
192	1	0	-4.666942	-0.405070	-1.896629
193	6	0	-4.761156	-1.249377	-5.060819
194	1	0	-6.298712	-1.794724	-3.611623
195	1	0	-5.011312	-1.642069	-6.034805
196	6	0	-2.456309	1.571265	-2.172144
197	1	0	-1.553034	2.167802	-2.011522
198	1	0	-2.678991	0.995807	-1.272776
199	8	0	-3.528930	2.472794	-2.445160
200	6	0	-4.579763	2.474399	-1.610507
201	8	0	-4.610174	1.828367	-0.581189
202	6	0	-5.708578	3.355831	-2.125380
203	6	0	-6.248970	2.717144	-3.418767
204	1	0	-6.619731	1.703388	-3.234446
205	1	0	-7.079527	3.320180	-3.799417
206	1	0	-5.477792	2.665488	-4.192358
207	6	0	-6.813209	3.421322	-1.069129
208	1	0	-6.444233	3.852738	-0.133475
209	1	0	-7.631397	4.049169	-1.435333
210	1	0	-7.215922	2.428067	-0.850426
211	6	0	-5.168654	4.765276	-2.417661
212	1	0	-5.986140	5.397164	-2.779234
213	1	0	-4.758123	5.226792	-1.513583
214	1	0	-4.387766	4.746700	-3.182180
215	1	0	-1.953527	1.236883	-4.235060
216	1	0	0.940428	0.582988	-2.882169
217	1	0	0.111485	0.316427	-3.171876

6a(*R*)•2a

RwB97XD SCF energy -5021.645700 a.u.

RwB97XD SCF enthalpy -5019.653781 a.u.

RwB97XD SCF free energy -5019.902454 a.u.

Three lowest frequencies (cm^{-1}) 11.0, 15.1, 18.0

Cartesian coordinates:

Standard orientation:

Center Number	Atomic Number	Atomic Type	Coordinates (Angstroms) X	Y	Z
1	6	0	-1.688650	2.111464	-1.190042
2	6	0	-1.915355	3.466590	-1.317884
3	6	0	-2.717127	4.197247	-0.447827
4	6	0	-3.314971	3.610259	0.638815
5	6	0	-3.086946	2.237397	0.809194
6	6	0	-2.308983	1.484630	-0.071720
7	1	0	-3.938347	4.171091	1.324765
8	1	0	-3.554897	1.754928	1.658877
9	6	0	-0.911763	1.453774	-2.279321
10	6	0	0.382674	0.875734	-2.171747
11	6	0	-1.449797	1.493209	-3.548066
12	6	0	1.018587	0.366559	-3.302322
13	6	0	-0.804120	0.985793	-4.668995
14	6	0	0.434760	0.401474	-4.578564
15	1	0	2.011119	-0.058036	-3.217174
16	1	0	0.947420	0.004059	-5.446626
17	8	0	-2.775807	5.491720	-0.860801
18	8	0	-1.422333	4.300072	-2.279269
19	8	0	-2.657737	2.007790	-3.917387
20	8	0	-1.592721	1.172806	-5.767018
21	6	0	-2.197477	5.493030	-2.168796
22	1	0	-1.553320	6.364000	-2.279703
23	1	0	-2.997034	5.476909	-2.919167
24	6	0	-2.603091	2.089667	-5.340945
25	1	0	-2.317386	3.107529	-5.633505
26	1	0	-3.564867	1.797527	-5.761515
27	15	0	1.148518	0.797003	-0.509048
28	15	0	-2.023582	-0.288062	0.316654
29	46	0	0.041185	-0.534997	1.152216
30	6	0	1.175597	2.526861	0.043877
31	6	0	1.689715	3.549494	-0.753741
32	6	0	0.729184	2.816661	1.322925
33	6	0	1.694085	4.869800	-0.318273
34	1	0	2.099602	3.292100	-1.723024
35	6	0	0.712278	4.120459	1.830498
36	1	0	0.368279	2.000992	1.941748
37	6	0	1.098174	5.140522	0.942120
38	6	0	2.898790	0.345231	-0.752801
39	6	0	3.915230	1.177072	-0.288824
40	6	0	3.239169	-0.894145	-1.285489
41	6	0	5.260055	0.856865	-0.456529
42	1	0	3.647965	2.100096	0.204568
43	6	0	4.569569	-1.279120	-1.492471
44	1	0	2.446847	-1.582281	-1.558378
45	6	0	5.561245	-0.332312	-1.161334
46	6	0	-3.394280	-0.712988	1.446242
47	6	0	-4.607932	-1.205369	0.972467
48	6	0	-3.240123	-0.499654	2.808814
49	6	0	-5.695710	-1.402305	1.819172
50	1	0	-4.707371	-1.435825	-0.077662
51	6	0	-4.282663	-0.693927	3.720086
52	1	0	-2.276579	-0.161186	3.173390
53	6	0	-5.536168	-1.043896	3.179590
54	6	0	-2.337069	-1.215782	-1.208328
55	6	0	-1.518020	-2.286846	-1.529678
56	6	0	-3.412327	-0.894821	-2.042105
57	6	0	-1.785425	-3.116841	-2.626010
58	1	0	-0.643815	-2.474701	-0.912136
59	6	0	-3.772190	-1.720812	-3.101622
60	1	0	-3.981654	0.004099	-1.837497
61	6	0	-2.993367	-2.887276	-3.307432
62	6	0	-0.695539	-4.146108	-3.001622
63	6	0	-0.542810	-5.186796	-1.878152
64	1	0	0.258385	-5.891776	-2.126109
65	1	0	-0.288280	-4.712087	-0.925188
66	1	0	-1.465643	-5.758272	-1.735058
67	6	0	-0.892920	-4.877615	-4.340456
68	1	0	-1.073133	-4.179789	-5.162054
69	1	0	0.029159	-5.425531	-4.564558
70	1	0	-1.701887	-5.608954	-4.321438
71	6	0	0.631730	-3.367743	-3.141977
72	1	0	0.565271	-2.619837	-3.939326
73	1	0	0.905926	-2.851889	-2.218826
74	1	0	1.441261	-4.062529	-3.387578
75	6	0	-4.915700	-1.324575	-4.057177
76	6	0	-4.333660	-1.198700	-5.478925
77	1	0	-3.970370	-2.160389	-5.848257
78	1	0	-5.101150	-0.835909	-6.172096
79	1	0	-3.496710	-0.494393	-5.494916
80	6	0	-6.071210	-2.343318	-4.059378
81	1	0	-6.415103	-2.557313	-3.041944
82	1	0	-6.919401	-1.927907	-4.614841
83	1	0	-5.792306	-3.281095	-4.540443
84	6	0	-5.514697	0.037516	-3.670236
85	1	0	-6.001919	0.006938	-2.688866
86	1	0	-4.762022	0.830763	-3.660505
87	1	0	-6.277861	0.311026	-4.405433
88	8	0	-3.447319	-3.804080	-4.223262
89	8	0	-6.654117	-1.062571	3.978566
90	8	0	0.918063	6.458603	1.287720
91	8	0	6.873562	-0.554742	-1.502145
92	6	0	7.158355	-0.243860	-2.863938
93	1	0	8.227441	-0.411149	-3.006652
94	1	0	6.599216	-0.885179	-3.551768
95	1	0	6.917596	0.800179	-3.090566
96	6	0	4.806104	-2.708345	-2.041482
97	6	0	6.247517	-3.235169	-1.914498
98	1	0	6.241462	-4.306571	-2.144340
99	1	0	6.945222	-2.763413	-2.606987
100	1	0	6.630472	-3.111001	-0.898909
101	6	0	4.369296	-2.765483	-3.517320
102	1	0	4.474786	-3.785842	-3.903222
103	1	0	3.322859	-2.467158	-3.638711
104	1	0	4.978761	-2.107485	-4.144567
105	6	0	-4.051318	-4.939874	-3.608327
106	1	0	-4.369732	-5.602399	-4.415162
107	1	0	-3.347827	-5.471603	-2.959626
108	1	0	-4.922248	-4.648296	-3.010696
109	6	0	-7.202803	0.235617	4.196545
110	1	0	-8.140925	0.093395	4.736178
111	1	0	-6.534736	0.862495	4.795581
112	1	0	-7.399474	0.745596	3.247659
113	6	0	-6.982741	-2.057085	1.273647
114	6	0	-6.825287	-2.442404	-0.208418
115	1	0	-6.689273	-1.563488	-0.848062
116	1	0	-5.986672	-3.126682	-0.375815
117	1	0	-7.735067	-2.951580	-0.541467
118	6	0	-7.259540	-3.352066	2.061911
119	1	0	-7.408948	-3.151736	3.125012
120	1	0	-8.164373	-3.835289	1.676390

121	1	0	-6.429158	-4.059103	1.957373
122	6	0	-8.206217	-1.126247	1.365553
123	1	0	-8.533231	-0.977451	2.394753
124	1	0	-7.998449	-0.149057	0.916706
125	1	0	-9.044073	-1.572176	0.818228
126	6	0	-3.925323	-0.532669	5.216744
127	6	0	-2.634833	-1.340225	5.495819
128	1	0	-2.783390	-2.401313	5.267967
129	1	0	-1.772007	-0.989400	4.923395
130	1	0	-2.375379	-1.256050	6.556309
131	6	0	-3.655561	0.952699	5.520159
132	1	0	-3.371807	1.079805	6.570850
133	1	0	-2.840262	1.344293	4.902253
134	1	0	-4.542092	1.567607	5.334174
135	6	0	-4.971150	-1.072768	6.208378
136	1	0	-4.530537	-1.054964	7.211076
137	1	0	-5.882730	-0.476610	6.250100
138	1	0	-5.244456	-2.107527	5.983913
139	6	0	6.345949	1.761537	0.164452
140	6	0	3.933195	-3.696008	-1.235540
141	1	0	4.185550	-3.665512	-0.171465
142	1	0	2.863669	-3.504092	-1.340491
143	1	0	4.107840	-4.715572	-1.594319
144	6	0	7.208598	0.926552	1.130039
145	1	0	7.978573	1.562791	1.581151
146	1	0	6.598492	0.510363	1.938502
147	1	0	7.705902	0.102092	0.616052
148	6	0	7.249439	2.411088	-0.900625
149	1	0	6.656391	2.892964	-1.685532
150	1	0	7.869365	3.182522	-0.430806
151	1	0	7.922561	1.688020	-1.362616
152	6	0	5.719537	2.904790	0.982845
153	1	0	5.064777	2.536332	1.780073
154	1	0	6.520548	3.480900	1.456542
155	1	0	5.144029	3.596645	0.357523
156	6	0	-0.422839	6.899037	1.085912
157	1	0	-0.440007	7.966096	1.316352
158	1	0	-0.734506	6.741006	0.049061
159	1	0	-1.124155	6.375172	1.743748
160	6	0	2.415027	5.957934	-1.141059
161	6	0	1.454440	7.040658	-1.662882
162	1	0	1.988835	7.699805	-2.355917
163	1	0	0.620470	6.588027	-2.208419
164	1	0	1.056341	7.662531	-0.860754
165	6	0	3.505178	6.603690	-0.263160
166	1	0	4.049561	7.359793	-0.840145
167	1	0	3.079473	7.089319	0.617114
168	1	0	4.228689	5.852471	0.072926
169	6	0	3.118099	5.350786	-2.367040
170	1	0	3.839020	4.574894	-2.087735
171	1	0	2.407544	4.924534	-3.083512
172	1	0	3.669817	6.141139	-2.885892
173	6	0	0.317837	4.276044	3.318414
174	6	0	-1.181459	3.979401	3.498635
175	1	0	-1.445988	4.024770	4.561288
176	1	0	-1.803457	4.703851	2.964230
177	1	0	-1.435168	2.980979	3.126578
178	6	0	0.643264	5.644395	3.942258
179	1	0	0.013612	6.450709	3.566130
180	1	0	0.473791	5.578019	5.022480
181	1	0	1.689614	5.920784	3.783628
182	6	0	1.119371	3.236013	4.137494
183	1	0	0.853481	2.203878	3.890671
184	1	0	2.196722	3.357868	3.981806
185	1	0	0.913157	3.374220	5.203879
186	8	0	1.869860	-0.472766	2.465314
187	6	0	2.509682	-1.691711	2.916147
188	6	0	1.615312	-2.855457	2.684251
189	6	0	1.417813	-3.697724	1.633339
190	8	0	0.707725	-3.114935	3.665270
191	6	0	0.306467	-4.527775	1.988054
192	1	0	1.995324	-3.724513	0.720628
193	6	0	-0.083501	-4.124856	3.224502
194	1	0	-0.131667	-5.324573	1.404367
195	1	0	-0.852361	-4.455295	3.906233
196	6	0	3.820900	-1.767185	2.151589
197	1	0	4.448696	-0.913891	2.416584
198	1	0	3.641690	-1.756384	1.073144
199	8	0	4.452898	-2.991228	2.525032
200	6	0	5.703593	-3.187137	2.086620
201	8	0	6.309029	-2.345171	1.454502
202	6	0	6.233257	-4.559881	2.479708
203	6	0	5.310627	-5.636085	1.880808
204	1	0	5.299205	-5.581371	0.787223
205	1	0	5.677803	-6.626949	2.166729
206	1	0	4.284099	-5.532981	2.243490
207	6	0	7.653140	-4.722023	1.934601
208	1	0	8.325395	-3.955332	2.330895
209	1	0	8.045286	-5.702065	2.223553
210	1	0	7.669796	-4.657467	0.842300
211	6	0	6.234237	-4.661657	4.015307
212	1	0	6.641598	-5.632435	4.314691
213	1	0	6.857544	-3.880160	4.462471
214	1	0	5.224016	-4.573335	4.424226
215	1	0	2.707664	-1.596832	3.987006
216	1	0	1.646226	0.069829	3.232394
217	1	0	-0.729834	-1.382488	2.206130

2.4. Catalytic cycle 2a –1b (*S*).

3a(*S*)-1b

RwB97XD SCF energy	-5094.190888 a.u.
RwB97XD SCF enthalpy	-5092.254020 a.u.
RwB97XD SCF free energy	-5092.494971 a.u.
Three lowest frequencies (cm^{-1})	16.7, 17.0, 22.7

Cartesian coordinates:

Center Number	Atomic Number	Atomic Type	Coordinates (Angstroms) X	Y	Z
1	6	0	-0.093825	-0.596982	2.572418
2	6	0	0.328614	-1.473368	3.549850
3	6	0	-0.138481	-2.780533	3.661852
4	6	0	-1.074204	-3.283822	2.793810
5	6	0	-1.505419	-2.423625	1.774904
6	6	0	-1.040804	-1.113196	1.645088
7	1	0	-1.457454	-4.293480	2.880503
8	1	0	-2.224546	-2.813911	1.066257
9	6	0	0.439409	0.793514	2.636683
10	6	0	1.419588	1.346354	1.767963
11	6	0	0.061176	1.581052	3.701622
12	6	0	1.937354	2.616029	2.008868
13	6	0	0.578007	2.853289	3.928760
14	6	0	1.524789	3.401595	3.098268
15	1	0	2.694146	3.021461	1.347415
16	1	0	1.943449	4.385663	3.273527
17	8	0	0.436329	-3.376220	4.738242
18	8	0	1.206331	-1.223610	4.559405
19	8	0	-0.836567	1.276896	4.679640
20	8	0	0.005079	3.376334	5.048518
21	6	0	1.514487	-2.508782	5.094175
22	1	0	2.433781	-2.879374	4.630356
23	1	0	1.597499	-2.446116	6.178816
24	6	0	-0.621350	2.262657	5.687347
25	1	0	0.052360	1.856998	6.453265
26	1	0	-1.576432	2.568555	6.112968
27	15	0	1.911202	0.351383	0.311374
28	15	0	-1.557906	-0.138821	0.175349
29	46	0	0.133180	0.048862	-1.266854
30	6	0	2.646733	-1.153171	1.011744
31	6	0	3.628450	-1.086701	1.996282
32	6	0	2.305566	-2.381944	0.467453
33	6	0	4.295989	-2.224446	2.448728
34	1	0	3.891894	-0.114136	2.396427
35	6	0	2.893621	-3.572275	0.905870
36	1	0	1.551460	-2.405902	-0.314010
37	6	0	3.853753	-3.471944	1.937136
38	6	0	3.289179	1.218696	-0.504191
39	6	0	4.520951	0.603450	-0.715332
40	6	0	3.036155	2.444875	-1.108053
41	6	0	5.538952	1.236871	-1.425173
42	1	0	4.684196	-0.389795	-0.320504
43	6	0	4.013860	3.140969	-1.824777
44	1	0	2.043902	2.874182	-1.015337
45	6	0	5.296414	2.556331	-1.876386
46	6	0	-2.971046	-1.108972	-0.435995
47	6	0	-4.239502	-1.018879	0.134184
48	6	0	-2.716967	-2.115713	-1.356385
49	6	0	-5.206350	-1.996057	-0.089153
50	1	0	-4.464822	-0.186126	0.785195
51	6	0	-3.645704	-3.121170	-1.640181
52	1	0	-1.749490	-2.136590	-1.846652
53	6	0	-4.835436	-3.107154	-0.887278
54	6	0	-2.160165	1.457060	0.788346
55	6	0	-1.773424	2.640322	0.168814
56	6	0	-2.991167	1.505847	1.910196
57	6	0	-2.272317	3.883149	0.582807
58	1	0	-1.062034	2.592646	-0.651226
59	6	0	-3.585245	2.695957	2.313676
60	1	0	-3.178341	0.592659	2.461600
61	6	0	-3.294634	3.853947	1.552579
62	6	0	-1.621412	5.165217	0.004931
63	6	0	-2.000878	5.384007	-1.468836
64	1	0	-1.509950	6.290106	-1.842337
65	1	0	-1.673349	4.548974	-2.094257
66	1	0	-3.079266	5.517639	-1.601630
67	6	0	-1.930023	6.448597	0.798281
68	1	0	-1.765301	6.311129	1.870566
69	1	0	-1.249986	7.234441	0.451270
70	1	0	-2.947647	6.811697	0.651620
71	6	0	-0.088449	4.988303	0.073594
72	1	0	0.247185	4.847591	1.105415
73	1	0	0.265133	4.140286	-0.518203
74	1	0	0.395472	5.885431	-0.325669
75	6	0	-4.462412	2.747902	3.579501
76	6	0	-3.933805	3.849680	4.520285
77	1	0	-4.072114	4.842407	4.089845
78	1	0	-4.462550	3.810121	5.478850
79	1	0	-2.865173	3.712935	4.712146
80	6	0	-5.938949	3.019436	3.238311
81	1	0	-6.313502	2.296089	2.505154
82	1	0	-6.548309	2.924907	4.144138
83	1	0	-6.087030	4.025614	2.843328
84	6	0	-4.401147	1.417299	4.348015
85	1	0	-4.850463	0.592072	3.784741
86	1	0	-3.372318	1.144053	4.603814
87	1	0	-4.964788	1.518593	5.280750
88	8	0	-4.031070	4.979827	1.814998
89	8	0	-5.675419	-4.191868	-0.907075
90	8	0	4.398504	-4.626257	2.432527
91	8	0	6.356643	3.255317	-2.400276
92	6	0	6.921012	4.183144	-1.479643
93	1	0	7.805138	4.605917	-1.960590
94	1	0	6.222685	4.991259	-1.238791
95	1	0	7.214431	3.689116	-0.546803
96	6	0	3.569812	4.455606	-2.509569
97	6	0	4.529311	4.988110	-3.588600
98	1	0	4.031449	5.810130	-4.114778
99	1	0	5.461183	5.381477	-3.183035
100	1	0	4.776123	4.218109	-4.324817
101	6	0	3.355219	5.538983	-1.436565
102	1	0	2.963080	6.454322	-1.894283
103	1	0	2.638707	5.205841	-0.679432
104	1	0	4.290009	5.791034	-0.926201
105	6	0	-5.010191	5.251459	0.817911
106	1	0	-5.539913	6.150630	1.137548
107	1	0	-4.555014	5.429104	-0.161225
108	1	0	-5.723369	4.423556	0.729757
109	6	0	-5.214616	-5.251191	-0.072520
110	1	0	-5.983116	-6.025842	-0.095124
111	1	0	-4.268315	-5.669077	-0.431971
112	1	0	-5.070953	-4.909126	0.958387
113	6	0	-6.616399	-1.844443	0.519526
114	6	0	-6.801785	-0.449280	1.142657
115	1	0	-6.163941	-0.301263	2.020822
116	1	0	-6.596239	0.351851	0.424517
117	1	0	-7.839119	-0.341188	1.474568
118	6	0	-7.685086	-2.007508	-0.578827
119	1	0	-7.626599	-2.989818	-1.049830
120	1	0	-8.683129	-1.893781	-0.141157
121	1	0	-7.571444	-1.244184	-1.355015
122	6	0	-6.867786	-2.880948	1.631269
123	1	0	-6.977801	-3.888769	1.228758
124	1	0	-6.055379	-2.880725	2.365887
125	1	0	-7.796350	-2.635097	2.158754
126	6	0	-3.254672	-4.120967	-2.753185
127	6	0	-2.031528	-4.944015	-2.303319
128	1	0	-1.168607	-4.302419	-2.095185
129	1	0	-2.248949	-5.520195	-1.397588
130	1	0	-1.742325	-5.647954	-3.091523
131	6	0	-4.367810	-5.080511	-3.208383
132	1	0	-4.027742	-5.588835	-4.117245
133	1	0	-4.598774	-5.853582	-2.475052
134	1	0	-5.291020	-4.546462	-3.446545

135	6	0	-2.867383	-3.303095	-4.005956
136	1	0	-2.539094	-3.983103	-4.798582
137	1	0	-3.728187	-2.738556	-4.379374
138	1	0	-2.053345	-2.597737	-3.818951
139	6	0	6.836131	0.471858	-1.763514
140	6	0	2.223908	4.203577	-3.229620
141	1	0	2.320298	3.399919	-3.967931
142	1	0	1.410912	3.942121	-2.549073
143	1	0	1.917294	5.112473	-3.757890
144	6	0	7.029540	0.470163	-3.293240
145	1	0	7.936369	-0.087451	-3.554184
146	1	0	6.181654	-0.014769	-3.789953
147	1	0	7.127504	1.483909	-3.686006
148	6	0	8.074371	1.088608	-1.086475
149	1	0	7.913147	1.225097	-0.011781
150	1	0	8.933273	0.420389	-1.214112
151	1	0	8.338652	2.050676	-1.526120
152	6	0	6.752903	-0.999185	-1.318514
153	1	0	5.901220	-1.519512	-1.769581
154	1	0	7.662503	-1.516749	-1.638931
155	1	0	6.683544	-1.106441	-0.230978
156	6	0	3.736051	-5.140781	3.576528
157	1	0	4.121926	-6.149242	3.739041
158	1	0	3.945725	-4.537698	4.467930
159	1	0	2.650533	-5.187696	3.426151
160	6	0	5.470998	-2.006679	3.438069
161	6	0	4.927588	-1.727943	4.851607
162	1	0	5.745977	-1.421268	5.512766
163	1	0	4.181842	-0.926179	4.840762
164	1	0	4.469582	-2.619126	5.290507
165	6	0	6.479067	-3.168379	3.508845
166	1	0	7.348405	-2.836155	4.086986
167	1	0	6.077849	-4.054266	3.999722
168	1	0	6.828972	-3.459208	2.513985
169	6	0	6.284109	-0.769021	2.989944
170	1	0	6.633541	-0.875574	1.957814
171	1	0	5.724095	0.166419	3.064334
172	1	0	7.163480	-0.664557	3.633289
173	6	0	2.440881	-4.859448	0.172309
174	6	0	0.941331	-5.088192	0.443822
175	1	0	0.593158	-5.973529	-0.100560
176	1	0	0.758720	-5.252920	1.511020
177	1	0	0.330086	-4.237290	0.126379
178	6	0	3.187360	-6.155696	0.528849
179	1	0	2.985297	-6.494803	1.546074
180	1	0	2.837618	-6.941829	-0.150145
181	1	0	4.267368	-6.057106	0.404497
182	6	0	2.650772	-4.643175	-1.343260
183	1	0	2.084840	-3.791644	-1.727724
184	1	0	3.708986	-4.473380	-1.569667
185	1	0	2.326127	-5.535231	-1.890076
186	8	0	1.425082	0.031808	-3.069549
187	6	0	0.839795	-0.215992	-4.136698
188	1	0	-1.097603	-0.083935	-2.205403
189	6	0	0.710563	-1.554787	-4.635030
190	6	0	0.213135	-2.068032	-5.811976
191	8	0	1.133526	-2.575304	-3.834159
192	6	0	0.342151	-3.472853	-5.720595
193	1	0	-0.191422	-1.500742	-6.638236
194	6	0	0.899172	-3.715501	-4.495600
195	1	0	0.055723	-4.211087	-6.454186
196	1	0	1.176894	-4.627793	-3.988050
197	6	0	0.195743	0.905559	-4.946502
198	1	0	0.346525	0.765863	-6.017360
199	1	0	0.610086	1.861184	-4.625666
200	8	0	-1.213038	0.849369	-4.702661
201	6	0	-1.732382	1.766434	-3.858009
202	8	0	-1.119805	2.745518	-3.488501
203	6	0	-3.117632	1.431467	-3.439148
204	6	0	-3.753954	0.255716	-3.847820
205	6	0	-3.762324	2.303959	-2.558108
206	6	0	-5.030800	-0.037044	-3.380444
207	1	0	-3.247182	-0.427922	-4.518502
208	6	0	-5.034797	2.006565	-2.089014
209	1	0	-3.258549	3.207147	-2.240494
210	6	0	-5.670982	0.838131	-2.504141
211	1	0	-5.523560	-0.952759	-3.691712
212	1	0	-5.527361	2.684911	-1.399121
213	1	0	-6.668617	0.611663	-2.142307

TS1a (S) -1b

RwB97XD SCF energy -5094.180672 a.u.
RwB97XD SCF enthalpy -5092.245983 a.u.
RwB97XD SCF free energy -5092.484916 a.u.
Three lowest frequencies (cm^{-1}) -617.5, 9.4, 16.7
Imaginary frequency (cm^{-1}) -617.5
Cartesian coordinates:

Center Number	Atomic Number	Atomic Type	Coordinates (Angstroms) X	Y	Z
1	6	0	-0.032458	-0.530011	2.523491
2	6	0	0.393322	-1.389045	3.515228
3	6	0	-0.042272	-2.707115	3.626414
4	6	0	-0.945483	-3.241143	2.742329
5	6	0	-1.384662	-2.395858	1.714256
6	6	0	-0.953863	-1.074469	1.585573
7	1	0	-1.303505	-4.260224	2.828179
8	1	0	-2.086198	-2.805606	0.998869
9	6	0	0.481364	0.867235	2.564591
10	6	0	1.391054	1.440056	1.634986
11	6	0	0.148490	1.657244	3.642179
12	6	0	1.874723	2.732698	1.817650
13	6	0	0.631634	2.951495	3.815115
14	6	0	1.496021	3.524271	2.913926
15	1	0	2.580881	3.148441	1.108406
16	1	0	1.884020	4.527270	3.046728
17	8	0	0.527074	-3.281181	4.717181
18	8	0	1.252149	-1.113100	4.532978
19	8	0	-0.673061	1.339910	4.679577
20	8	0	0.114290	3.473180	4.960782
21	6	0	1.576970	-2.386195	5.087660
22	1	0	2.513980	-2.740053	4.647423
23	1	0	1.634172	-2.310951	6.173174
24	6	0	-0.435105	2.351603	5.656144
25	1	0	0.295550	1.985759	6.388608
26	1	0	-1.375307	2.634098	6.128925
27	15	0	1.894211	0.416244	0.213885
28	15	0	-1.469812	-0.130924	0.093279
29	46	0	0.257030	-0.110960	-1.383684
30	6	0	2.638056	-1.063843	0.951096
31	6	0	3.578582	-0.949639	1.970130
32	6	0	2.376320	-2.306551	0.394739
33	6	0	4.287713	-2.054519	2.441385
34	1	0	3.787134	0.034332	2.375094
35	6	0	2.980286	-3.470422	0.877028
36	1	0	1.685763	-2.363452	-0.440960
37	6	0	3.908485	-3.323358	1.932975
38	6	0	3.254187	1.254760	-0.652991
39	6	0	4.479254	0.617444	-0.837768
40	6	0	3.011036	2.457538	-1.305614
41	6	0	5.503563	1.209799	-1.571269
42	1	0	4.630469	-0.361226	-0.405222
43	6	0	3.999609	3.114354	-2.045262
44	1	0	2.023052	2.899581	-1.233361
45	6	0	5.276386	2.514738	-2.070151
46	6	0	-2.945863	-1.058132	-0.440187
47	6	0	-4.184450	-0.858666	0.172447
48	6	0	-2.784875	-2.143125	-1.290945
49	6	0	-5.200850	-1.805149	0.083746
50	1	0	-4.344412	0.037896	0.753762
51	6	0	-3.774959	-3.122850	-1.441471
52	1	0	-1.840331	-2.257140	-1.814707
53	6	0	-4.915483	-2.995884	-0.627584

54	6	0	-2.027209	1.493860	0.677862
55	6	0	-1.697705	2.651573	-0.015929
56	6	0	-2.793315	1.582607	1.844163
57	6	0	-2.185931	3.906849	0.378219
58	1	0	-1.052668	2.576619	-0.886331
59	6	0	-3.377860	2.781635	2.232937
60	1	0	-2.942693	0.691695	2.441854
61	6	0	-3.140880	3.911475	1.412839
62	6	0	-1.594454	5.167719	-0.300252
63	6	0	-2.064793	5.292752	-1.759191
64	1	0	-1.613746	6.181273	-2.215921
65	1	0	-1.766202	4.422978	-2.350502
66	1	0	-3.151805	5.403278	-1.830660
67	6	0	-1.881315	6.490250	0.434667
68	1	0	-1.656100	6.418970	1.502301
69	1	0	-1.235770	7.264328	0.005127
70	1	0	-2.911764	6.829582	0.324592
71	6	0	-0.057495	5.019693	-0.304948
72	1	0	0.337030	4.969290	0.714770
73	1	0	0.277305	4.128894	-0.840994
74	1	0	0.388514	5.887088	-0.801766
75	6	0	-4.180709	2.879326	3.544863
76	6	0	-3.562138	3.982488	4.426630
77	1	0	-3.684551	4.967516	3.973178
78	1	0	-4.044600	3.992588	5.410162
79	1	0	-2.491444	3.809646	4.570640
80	6	0	-5.667474	3.192455	3.291857
81	1	0	-6.103949	2.487119	2.576546
82	1	0	-6.223477	3.102366	4.231782
83	1	0	-5.813992	4.206207	2.917499
84	6	0	-4.115926	1.560861	4.333431
85	1	0	-4.609218	0.737179	3.805045
86	1	0	-3.084639	1.266238	4.549968
87	1	0	-4.635800	1.692105	5.287661
88	8	0	-3.865657	5.044027	1.678574
89	8	0	-5.789761	-4.044748	-0.499732
90	8	0	4.489454	-4.450985	2.447124
91	8	0	6.345696	3.183196	-2.612433
92	6	0	6.921134	4.131365	-1.718775
93	1	0	7.812160	4.525913	-2.210546
94	1	0	6.233742	4.956253	-1.504800
95	1	0	7.205148	3.662047	-0.770583
96	6	0	3.576731	4.410895	-2.775926
97	6	0	4.550258	4.901297	-3.862677
98	1	0	4.066224	5.716740	-4.411252
99	1	0	5.485746	5.291174	-3.462536
100	1	0	4.789230	4.109733	-4.578282
101	6	0	3.368067	5.527860	-1.736553
102	1	0	2.986895	6.433075	-2.222550
103	1	0	2.646889	5.224448	-0.971829
104	1	0	4.303768	5.785637	-1.230781
105	6	0	-4.916040	5.265167	0.742922
106	1	0	-5.439706	6.165534	1.068993
107	1	0	-4.530524	5.418501	-0.269715
108	1	0	-5.617135	4.422474	0.729737
109	6	0	-5.312635	-5.043989	0.398162
110	1	0	-6.084877	-5.813587	0.448259
111	1	0	-4.377211	-5.491670	0.046336
112	1	0	-5.143583	-4.627975	1.397581
113	6	0	-6.571633	-1.543339	0.741682
114	6	0	-6.676340	-0.088767	1.233007
115	1	0	-5.982802	0.119394	2.054561
116	1	0	-6.486608	0.631800	0.430645
117	1	0	-7.688042	0.089154	1.610836
118	6	0	-7.698952	-1.770930	-0.283927
119	1	0	-7.710364	-2.800893	-0.643420
120	1	0	-8.668695	-1.558487	0.179690
121	1	0	-7.584228	-1.105909	-1.144761
122	6	0	-6.801410	-2.455057	1.962174
123	1	0	-6.963866	-3.493983	1.672313
124	1	0	-5.955586	-2.411844	2.657062
125	1	0	-7.694731	-2.122394	2.502519
126	6	0	-3.507608	-4.231810	-2.483773
127	6	0	-2.277260	-5.053529	-2.054449
128	1	0	-1.387771	-4.424931	-1.950361
129	1	0	-2.448940	-5.554760	-1.095684
130	1	0	-2.056050	-5.821336	-2.803815
131	6	0	-4.675477	-5.193093	-2.764830
132	1	0	-4.411332	-5.797455	-3.639988
133	1	0	-4.877248	-5.885442	-1.947130
134	1	0	-5.598389	-4.655215	-2.994757
135	6	0	-3.214897	-3.533576	-3.828964
136	1	0	-2.948579	-4.280222	-4.584065
137	1	0	-4.102478	-2.997668	-4.182334
138	1	0	-2.389214	-2.822319	-3.765462
139	6	0	6.790121	0.414867	-1.880556
140	6	0	2.235260	4.152871	-3.502502
141	1	0	2.331622	3.330306	-4.219764
142	1	0	1.412225	3.919652	-2.823371
143	1	0	1.944049	5.051266	-4.056786
144	6	0	6.989749	0.361877	-3.408475
145	1	0	7.888315	-0.218658	-3.646880
146	1	0	6.136315	-0.124533	-3.893898
147	1	0	7.106748	1.360596	-3.832999
148	6	0	8.034666	1.033484	-1.217227
149	1	0	7.872295	1.201599	-0.147125
150	1	0	8.884320	0.349873	-1.323405
151	1	0	8.313228	1.979253	-1.682960
152	6	0	6.681058	-1.040371	-1.390407
153	1	0	5.820899	-1.559543	-1.826537
154	1	0	7.582169	-1.582708	-1.693337
155	1	0	6.609439	-1.112923	-0.300095
156	6	0	3.860160	-4.952812	3.616083
157	1	0	4.254725	-5.957148	3.781848
158	1	0	4.088864	-4.335181	4.492868
159	1	0	2.771888	-5.006430	3.493348
160	6	0	5.462089	-1.767230	3.414544
161	6	0	4.927613	-1.436432	4.819617
162	1	0	5.744468	-1.072471	5.452796
163	1	0	4.156016	-0.660337	4.783006
164	1	0	4.505473	-2.319913	5.307540
165	6	0	6.502447	-2.896358	3.528282
166	1	0	7.359959	-2.516568	4.094405
167	1	0	6.128406	-3.775091	4.052362
168	1	0	6.863398	-3.212966	2.545486
169	6	0	6.240009	-0.532778	2.897665
170	1	0	6.588408	-0.685474	1.870970
171	1	0	5.653977	0.389189	2.923650
172	1	0	7.118465	-0.369900	3.530035
173	6	0	2.655928	-4.766727	0.092927
174	6	0	1.127691	-4.908505	-0.077429
175	1	0	0.904487	-5.823900	-0.636506
176	1	0	0.631668	-4.981202	0.896371
177	1	0	0.677820	-4.075369	-0.624099
178	6	0	3.156785	-6.088642	0.696187
179	1	0	2.707719	-6.292485	1.671603
180	1	0	2.857569	-6.901173	0.024548
181	1	0	4.241240	-6.116143	0.801611
182	6	0	3.313435	-4.615853	-1.296326
183	1	0	2.937915	-3.737588	-1.829251
184	1	0	4.400113	-4.515650	-1.200212
185	1	0	3.107238	-5.500676	-1.909480
186	8	0	1.569864	-0.279629	-3.026169
187	6	0	0.523178	-0.626157	-3.689766
188	1	0	-0.734190	-0.547613	-2.589860
189	6	0	0.369574	-2.041809	-4.071324
190	6	0	-0.102238	-2.646940	-5.198661
191	8	0	0.859346	-2.971234	-3.210800
192	6	0	0.120058	-4.045246	-5.019484
193	1	0	-0.547016	-2.160663	-6.055082
194	6	0	0.695855	-4.179073	-3.795858
195	1	0	-0.124882	-4.841033	-5.707219
196	1	0	1.033417	-5.031595	-3.226270
197	6	0	-0.104956	0.407226	-4.636633
198	1	0	0.111989	0.129791	-5.669934
199	1	0	0.335244	1.378668	-4.416181

200	8	0	-1.522209	0.446558	-4.498656
201	6	0	-2.012164	1.439177	-3.723500
202	8	0	-1.343879	2.392216	-3.379905
203	6	0	-3.425764	1.227163	-3.332747
204	6	0	-4.127131	0.068351	-3.678293
205	6	0	-4.034249	2.205560	-2.541058
206	6	0	-5.435822	-0.100783	-3.239983
207	1	0	-3.646837	-0.696215	-4.277148
208	6	0	-5.341739	2.034823	-2.107158
209	1	0	-3.477663	3.093686	-2.271032
210	6	0	-6.044038	0.884673	-2.462922
211	1	0	-5.980073	-1.003592	-3.498946
212	1	0	-5.811922	2.797477	-1.494091
213	1	0	-7.069123	0.756692	-2.130266

4a(*S*) -1b

RwB97XD SCF energy -5094.196518 a.u.

RwB97XD SCF enthalpy -5092.257156 a.u.

RwB97XD SCF free energy -5092.496500 a.u.

Three lowest frequencies (cm^{-1}) 17.9, 18.5, 21.7

Cartesian coordinates:

Standard orientation:

Center Number	Atomic Number	Atomic Type	Coordinates (Angstroms) X	Y	Z
1	6	0	0.382735	-0.942133	2.648145
2	6	0	0.826744	-1.876768	3.559979
3	6	0	0.365772	-3.190834	3.587239
4	6	0	-0.547025	-3.657326	2.674487
5	6	0	-1.020793	-2.728448	1.736379
6	6	0	-0.593693	-1.401916	1.721082
7	1	0	-0.902402	-4.680789	2.689339
8	1	0	-1.758738	-3.065426	1.017428
9	6	0	0.857704	0.463991	2.752798
10	6	0	1.532406	1.209551	1.743021
11	6	0	0.614979	1.140417	3.928536
12	6	0	1.863816	2.547502	1.951223
13	6	0	0.949585	2.477117	4.124932
14	6	0	1.565683	3.217829	3.146519
15	1	0	2.387910	3.099832	1.180761
16	1	0	1.823324	4.259989	3.291983
17	8	0	0.958970	-3.856744	4.613616
18	8	0	1.742154	-1.703459	4.553126
19	8	0	-0.015284	0.666497	5.038091
20	8	0	0.545314	2.865941	5.363009
21	6	0	1.591174	-2.843587	5.398768
22	1	0	2.568316	-3.194364	5.728588
23	1	0	0.943257	-2.583937	6.244634
24	6	0	0.192172	1.658364	6.043986
25	1	0	1.021195	1.352991	6.692476
26	1	0	-0.731636	1.808091	6.602188
27	15	0	1.959454	0.411171	0.162858
28	15	0	-1.239436	-0.277397	0.434699
29	46	0	0.261424	0.018788	-1.264352
30	6	0	2.780803	-1.133711	0.611811
31	6	0	3.759230	-1.142751	1.602361
32	6	0	2.455104	-2.310221	-0.048425
33	6	0	4.387687	-2.324876	1.992094
34	1	0	4.039501	-0.206280	2.074751
35	6	0	3.004450	-3.536275	0.329898
36	1	0	1.741261	-2.269898	-0.865395
37	6	0	3.908885	-3.524273	1.412942
38	6	0	3.154070	1.453378	-0.710525
39	6	0	4.449789	1.004860	-0.954246
40	6	0	2.718609	2.644373	-1.281353
41	6	0	5.355292	1.779832	-1.675389
42	1	0	4.748829	0.036523	-0.577894
43	6	0	3.574257	3.467828	-2.016442
44	1	0	1.679620	2.929623	-1.156855
45	6	0	4.921855	3.056858	-2.105579
46	6	0	-2.726950	-1.107117	-0.201222
47	6	0	-3.956818	-0.964500	0.442186
48	6	0	-2.629058	-1.950213	-1.299291
49	6	0	-5.054973	-1.742151	0.092691
50	1	0	-4.047763	-0.245319	1.243418
51	6	0	-3.710660	-2.726907	-1.736481
52	1	0	-1.675705	-2.028370	-1.813527
53	6	0	-4.871901	-2.695226	-0.940398
54	6	0	-1.843271	1.216730	1.260840
55	6	0	-1.748592	2.426527	0.590986
56	6	0	-2.473445	1.161326	2.505794
57	6	0	-2.346608	3.593331	1.084274
58	1	0	-1.216891	2.455505	-0.353178
59	6	0	-3.131359	2.271130	3.026170
60	1	0	-2.459929	0.228360	3.056574
61	6	0	-3.134746	3.453086	2.241471
62	6	0	-2.060865	4.905643	0.317420
63	6	0	-2.800833	4.898374	-1.033006
64	1	0	-2.595783	5.828697	-1.575056
65	1	0	-2.473879	4.063893	-1.660575
66	1	0	-3.884336	4.819441	-0.902470
67	6	0	-2.405044	6.194474	1.084702
68	1	0	-1.968860	6.195584	2.087791
69	1	0	-1.982347	7.043437	0.536480
70	1	0	-3.476570	6.372127	1.175545
71	6	0	-0.543111	4.977594	0.036187
72	1	0	0.030965	4.967793	0.969203
73	1	0	-0.190092	4.157195	-0.593236
74	1	0	-0.316881	5.908855	-0.492883
75	6	0	-3.751798	2.221703	4.437450
76	6	0	-3.075301	3.302234	5.304280
77	1	0	-3.299845	4.305950	4.937463
78	1	0	-3.427870	3.230034	6.339335
79	1	0	-1.988234	3.178632	5.300916
80	6	0	-5.275430	2.448405	4.426424
81	1	0	-5.771306	1.780749	3.713613
82	1	0	-5.681379	2.233554	5.421113
83	1	0	-5.536044	3.478554	4.183308
84	6	0	-3.505479	0.859192	5.105831
85	1	0	-4.013222	0.043737	4.577965
86	1	0	-2.440618	0.618661	5.174195
87	1	0	-3.906752	0.887480	6.123747
88	8	0	-3.927122	4.490281	2.668014
89	8	0	-5.880604	-3.601123	-1.155710
90	8	0	4.330474	-4.719897	1.931795
91	8	0	5.862460	3.889932	-2.658529
92	6	0	6.330602	4.890994	-1.758020
93	1	0	7.125748	5.430464	-2.275877
94	1	0	5.538109	5.594193	-1.485316
95	1	0	6.730357	4.443341	-0.842056
96	6	0	2.943042	4.713251	-2.684080
97	6	0	3.784451	5.346111	-3.806974
98	1	0	3.167522	6.092128	-4.319418
99	1	0	4.674901	5.858762	-3.443872
100	1	0	4.092950	4.602252	-4.547222
101	6	0	2.644653	5.774151	-1.609252
102	1	0	2.110490	6.622070	-2.052604
103	1	0	2.022673	5.362569	-0.808946
104	1	0	3.562822	6.159360	-1.155269
105	6	0	-5.147986	4.604415	1.941072
106	1	0	-5.720549	5.402319	2.417234
107	1	0	-4.973613	4.863479	0.892506
108	1	0	-5.718296	3.669963	1.977910
109	6	0	-5.611745	-4.886077	-0.598537
110	1	0	-6.511796	-5.484253	-0.750859
111	1	0	-4.765581	-5.374897	-1.091002
112	1	0	-5.396592	-4.815386	0.472753
113	6	0	-6.407433	-1.531859	0.807032
114	6	0	-6.352587	-0.310660	1.743137
115	1	0	-5.656499	-0.460121	2.575813
116	1	0	-6.066547	0.604682	1.213330
117	1	0	-7.345248	-0.147428	2.174304
118	6	0	-7.515425	-1.266893	-0.229349
119	1	0	-7.624166	-2.099120	-0.926701
120	1	0	-8.473612	-1.122466	0.282041
121	1	0	-7.300800	-0.361653	-0.804387
122	6	0	-6.792671	-2.748609	1.670095
123	1	0	-7.091758	-3.603734	1.062937
124	1	0	-5.967188	-3.051441	2.323282
125	1	0	-7.644176	-2.487606	2.308543
126	6	0	-3.512403	-3.515157	-3.052571
127	6	0	-2.472467	-4.629608	-2.834447
128	1	0	-1.523940	-4.225513	-2.466900
129	1	0	-2.823532	-5.370962	-2.109280
130	1	0	-2.273604	-5.147194	-3.779437
131	6	0	-4.787162	-4.121141	-3.664248
132	1	0	-4.541347	-4.485095	-4.667898
133	1	0	-5.180821	-4.969121	-3.103654
134	1	0	-5.579312	-3.374192	-3.766229
135	6	0	-2.973583	-2.527885	-4.111133

136	1	0	-2.791119	-3.057159	-5.052127
137	1	0	-3.700419	-1.731859	-4.302449
138	1	0	-2.034139	-2.064458	-3.811704
139	6	0	6.733933	1.200997	-2.059949
140	6	0	1.610906	4.291771	-3.348143
141	1	0	1.779696	3.503870	-4.090095
142	1	0	0.863884	3.936353	-2.634636
143	1	0	1.173392	5.153569	-3.862708
144	6	0	6.847515	1.179118	-3.597387
145	1	0	7.813553	0.755023	-3.893662
146	1	0	6.058883	0.556146	-4.033636
147	1	0	6.769859	2.180730	-4.025287
148	6	0	7.903982	2.001783	-1.458564
149	1	0	7.773890	2.143104	-0.380211
150	1	0	8.839151	1.451723	-1.611329
151	1	0	8.021529	2.978711	-1.927996
152	6	0	6.885702	-0.248808	-1.569206
153	1	0	6.106025	-0.909088	-1.963739
154	1	0	7.849090	-0.639570	-1.911545
155	1	0	6.875656	-0.315959	-0.476448
156	6	0	3.526113	-5.135965	3.028534
157	1	0	3.930629	-6.088629	3.375861
158	1	0	3.563742	-4.403720	3.843214
159	1	0	2.481680	-5.272191	2.723676
160	6	0	5.571702	-2.187546	2.981366
161	6	0	5.068168	-1.640947	4.328405
162	1	0	5.916313	-1.468027	5.000212
163	1	0	4.531374	-0.694173	4.217072
164	1	0	4.399606	-2.358576	4.811286
165	6	0	6.374209	-3.469809	3.259328
166	1	0	7.256467	-3.195075	3.848689
167	1	0	5.814431	-4.204431	3.840340
168	1	0	6.722792	-3.946556	2.340067
169	6	0	6.572482	-1.179984	2.370395
170	1	0	6.963203	-1.553067	1.417939
171	1	0	6.129878	-0.195271	2.197018
172	1	0	7.418866	-1.042758	3.051874
173	6	0	2.615042	-4.751201	-0.545107
174	6	0	1.078919	-4.875205	-0.612357
175	1	0	0.803973	-5.717846	-1.256439
176	1	0	0.659224	-5.061010	0.381786
177	1	0	0.599708	-3.979678	-1.018600
178	6	0	3.164924	-6.119034	-0.112425
179	1	0	2.774301	-6.444599	0.854352
180	1	0	2.848508	-6.858615	-0.856876
181	1	0	4.255313	-6.133621	-0.068522
182	6	0	3.174285	-4.473088	-1.958048
183	1	0	2.775088	-3.550244	-2.387845
184	1	0	4.266098	-4.387415	-1.930025
185	1	0	2.916987	-5.296824	-2.633038
186	8	0	1.398325	0.055521	-2.966104
187	6	0	0.299764	-0.299399	-3.720428
188	6	0	0.446256	-1.618437	-4.416497
189	6	0	0.411123	-2.037549	-5.708385
190	8	0	0.584660	-2.689451	-3.585919
191	6	0	0.543300	-3.465439	-5.672438
192	1	0	0.295960	-1.418131	-6.586240
193	6	0	0.642141	-3.801894	-4.363255
194	1	0	0.559399	-4.144591	-6.512733
195	1	0	0.758831	-4.740449	-3.843979
196	6	0	-0.214370	0.831510	-4.631698
197	1	0	0.029846	0.675914	-5.681651
198	1	0	0.211262	1.774530	-4.288780
199	8	0	-1.650434	0.887679	-4.574730
200	6	0	-2.162842	1.473675	-3.486143
201	8	0	-1.470192	2.039279	-2.654729
202	6	0	-3.633254	1.339925	-3.375036
203	6	0	-4.421756	0.924609	-4.452337
204	6	0	-4.221703	1.621922	-2.138319
205	6	0	-5.796883	0.799299	-4.289041
206	1	0	-3.962576	0.707253	-5.410376
207	6	0	-5.595425	1.490978	-1.980582
208	1	0	-3.598644	1.929951	-1.305140
209	6	0	-6.382702	1.081177	-3.055811
210	1	0	-6.412657	0.481716	-5.124329
211	1	0	-6.051855	1.704345	-1.019063
212	1	0	-7.456302	0.977449	-2.931308
213	1	0	-0.594761	-0.495053	-3.025854

5a(*S*) -1b

RwB97XD SCF energy -5095.370706 a.u.
RwB97XD SCF enthalpy -5093.413666 a.u.
RwB97XD SCF free energy -5093.657583 a.u.
Three lowest frequencies (cm^{-1}) 14.9, 17.3, 19.1
Cartesian coordinates:

Center Number	Atomic Number	Atomic Type	Coordinates X	(Angstroms) Y	Z
1	6	0	-0.433387	-1.868167	-2.420776
2	6	0	-0.123413	-2.073826	-3.746720
3	6	0	-0.575776	-1.244410	-4.770004
4	6	0	-1.365973	-0.151611	-4.517830
5	6	0	-1.692557	0.086934	-3.174726
6	6	0	-1.247315	-0.735017	-2.142335
7	1	0	-1.729882	0.490186	-5.311014
8	1	0	-2.328591	0.934778	-2.953666
9	6	0	-0.039777	-2.889567	-1.415911
10	6	0	0.790995	-2.695011	-0.278523
11	6	0	-0.555043	-4.158475	-1.574054
12	6	0	1.015200	-3.743644	0.614769
13	6	0	-0.327600	-5.194081	-0.674242
14	6	0	0.450405	-5.015447	0.443164
15	1	0	1.657533	-3.594180	1.473201
16	1	0	0.632603	-5.814350	1.151922
17	8	0	-0.120970	-1.710763	-5.962964
18	8	0	0.630021	-3.075186	-4.278289
19	8	0	-1.383788	-4.594288	-2.562964
20	8	0	-1.000328	-6.303187	-1.083331
21	6	0	0.389179	-3.017039	-5.684950
22	1	0	1.322494	-3.168405	-6.224996
23	1	0	-0.364995	-3.765383	-5.954434
24	6	0	-1.420739	-6.014939	-2.419816
25	1	0	-0.716020	-6.467572	-3.127155
26	1	0	-2.439446	-6.371036	-2.569605
27	15	0	1.505264	-1.050890	0.073471
28	15	0	-1.672785	-0.341678	-0.413943
29	46	0	0.023659	0.447807	0.935842
30	6	0	2.193232	-0.419460	-1.474203
31	6	0	3.077221	-1.180599	-2.233407
32	6	0	1.841321	0.856851	-1.899125
33	6	0	3.590632	-0.706115	-3.442009
34	1	0	3.363714	-2.164973	-1.876424
35	6	0	2.304227	1.388155	-3.101344
36	1	0	1.174472	1.446012	-1.278637
37	6	0	3.107562	0.542872	-3.897713
38	6	0	2.820695	-1.306287	1.293518
39	6	0	4.169750	-1.123845	1.004260
40	6	0	2.427088	-1.549048	2.603957
41	6	0	5.142686	-1.267900	1.995122
42	1	0	4.459607	-0.859053	-0.003842
43	6	0	3.341805	-1.667550	3.649166
44	1	0	1.367689	-1.617098	2.823932
45	6	0	4.706939	-1.612381	3.298086
46	6	0	-2.928750	0.968378	-0.464622
47	6	0	-4.211677	0.743408	0.019298
48	6	0	-2.553112	2.253178	-0.838670
49	6	0	-5.153848	1.767424	0.078255
50	1	0	-4.473687	-0.245531	0.366293
51	6	0	-3.448651	3.324737	-0.823234
52	1	0	-1.526217	2.425322	-1.140961
53	6	0	-4.775812	3.035061	-0.428259
54	6	0	-2.504759	-1.776616	0.295509
55	6	0	-2.338452	-2.021507	1.648965
56	6	0	-3.382239	-2.562158	-0.452561
57	6	0	-3.096442	-2.985322	2.321981
58	1	0	-1.606404	-1.435695	2.197756
59	6	0	-4.190527	-3.512191	0.162811
60	1	0	-3.443271	-2.402905	-1.522137
61	6	0	-4.103070	-3.623667	1.574894
62	6	0	-2.724554	-3.250169	3.798021
63	6	0	-3.070739	-2.012397	4.646768
64	1	0	-2.790412	-2.182398	5.692186
65	1	0	-2.537483	-1.121986	4.296722
66	1	0	-4.143359	-1.794463	4.617732
67	6	0	-3.371400	-4.493425	4.432402
68	1	0	-3.224950	-5.385878	3.818221
69	1	0	-2.891512	-4.670892	5.401052
70	1	0	-4.438920	-4.375379	4.620854

71	6	0	-1.198118	-3.486609	3.865752
72	1	0	-0.908765	-4.363428	3.275863
73	1	0	-0.619427	-2.631888	3.506240
74	1	0	-0.904731	-3.664022	4.905394
75	6	0	-5.080880	-4.446455	-0.680914
76	6	0	-4.673006	-5.903478	-0.384842
77	1	0	-4.874069	-6.169861	0.654980
78	1	0	-5.236405	-6.588630	-1.028016
79	1	0	-3.604838	-6.054143	-0.572542
80	6	0	-6.581642	-4.258836	-0.390426
81	1	0	-6.870890	-3.204495	-0.460352
82	1	0	-7.168269	-4.814226	-1.130731
83	1	0	-6.859896	-4.634365	0.594489
84	6	0	-4.876847	-4.199240	-2.184890
85	1	0	-5.219812	-3.204052	-2.489509
86	1	0	-3.830538	-4.310752	-2.484086
87	1	0	-5.464009	-4.931476	-2.747867
88	8	0	-5.042148	-4.397308	2.210600
89	8	0	-5.747837	4.004002	-0.471482
90	8	0	3.419084	0.942035	-5.169456
91	8	0	5.671172	-1.852294	4.246384
92	6	0	5.891179	-3.236503	4.507141
93	1	0	6.735805	-3.293866	5.196230
94	1	0	5.019342	-3.706495	4.971397
95	1	0	6.130999	-3.779724	3.587426
96	6	0	2.750660	-1.796576	5.074408
97	6	0	3.723174	-1.439320	6.212432
98	1	0	3.155677	-1.387171	7.147870
99	1	0	4.512057	-2.176358	6.358825
100	1	0	4.189509	-0.462796	6.050983
101	6	0	2.213128	-3.224613	5.277927
102	1	0	1.704746	-3.304150	6.245463
103	1	0	1.494842	-3.487956	4.495368
104	1	0	3.014081	-3.969755	5.259360
105	6	0	-6.074702	-3.622809	2.818705
106	1	0	-6.779378	-4.331950	3.256743
107	1	0	-5.682311	-2.968651	3.603642
108	1	0	-6.592894	-3.004931	2.076890
109	6	0	-6.331088	4.215955	-1.755151
110	1	0	-7.289669	4.711913	-1.589596
111	1	0	-5.706763	4.861824	-2.379260
112	1	0	-6.495454	3.268170	-2.277405
113	6	0	-6.516585	1.503295	0.754919
114	6	0	-6.605206	0.062938	1.296132
115	1	0	-6.565953	-0.682702	0.493996
116	1	0	-5.814222	-0.164190	2.019242
117	1	0	-7.563039	-0.061323	1.810863
118	6	0	-6.668801	2.455717	1.956964
119	1	0	-6.627697	3.502041	1.647801
120	1	0	-7.631964	2.283376	2.450749
121	1	0	-5.876632	2.281666	2.693804
122	6	0	-7.698932	1.692994	-0.213601
123	1	0	-7.874128	2.743232	-0.446108
124	1	0	-7.539113	1.147922	-1.149936
125	1	0	-8.613651	1.303513	0.246629
126	6	0	-2.888969	4.712701	-1.219766
127	6	0	-2.880696	4.847951	-2.753974
128	1	0	-2.313561	4.029777	-3.210599
129	1	0	-3.887145	4.833315	-3.179668
130	1	0	-2.405318	5.791684	-3.044495
131	6	0	-3.644988	5.891937	-0.581112
132	1	0	-3.081206	6.812773	-0.763212
133	1	0	-4.644515	6.035800	-0.988990
134	1	0	-3.733591	5.763287	0.502272
135	6	0	-1.424287	4.832100	-0.738422
136	1	0	-1.077873	5.860799	-0.879376
137	1	0	-1.336465	4.587119	0.323450
138	1	0	-0.740573	4.192412	-1.302500
139	6	0	6.619474	-0.935131	1.685783
140	6	0	1.566483	-0.809120	5.216248
141	1	0	1.876020	0.217471	4.993141
142	1	0	0.718343	-1.056214	4.571945
143	1	0	1.196219	-0.837215	6.246201
144	6	0	7.004984	0.321850	2.491083
145	1	0	8.048869	0.589360	2.290397
146	1	0	6.375442	1.172370	2.204791
147	1	0	6.896861	0.157997	3.566150
148	6	0	7.588712	-2.086219	2.018977
149	1	0	7.247037	-3.031577	1.583878
150	1	0	8.572533	-1.864042	1.591593
151	1	0	7.723092	-2.221045	3.091327
152	6	0	6.822551	-0.619232	0.196232
153	1	0	6.225389	0.233461	-0.137257
154	1	0	7.873349	-0.365500	0.024842
155	1	0	6.584613	-1.482026	-0.433497
156	6	0	2.466017	0.492226	-6.124456
157	1	0	2.810880	0.836288	-7.101542
158	1	0	2.393185	-0.601874	-6.125943
159	1	0	1.473701	0.913399	-5.921114
160	6	0	4.650891	-1.586141	-4.148262
161	6	0	3.993870	-2.880932	-4.653728
162	1	0	4.753838	-3.546964	-5.077771
163	1	0	3.480480	-3.422068	-3.853183
164	1	0	3.267639	-2.658274	-5.440543
165	6	0	5.387010	-0.932836	-5.330482
166	1	0	6.208462	-1.595560	-5.625585
167	1	0	4.749411	-0.797781	-6.205184
168	1	0	5.819036	0.034945	-5.063370
169	6	0	5.738500	-1.940225	-3.110561
170	1	0	6.214928	-1.032256	-2.726355
171	1	0	5.347087	-2.506144	-2.261157
172	1	0	6.512534	-2.555124	-3.581781
173	6	0	1.879816	2.839741	-3.439112
174	6	0	0.378248	2.863873	-3.770220
175	1	0	0.052550	3.894743	-3.949270
176	1	0	0.159505	2.277790	-4.668273
177	1	0	-0.219192	2.455745	-2.948846
178	6	0	2.642597	3.502576	-4.599018
179	1	0	2.391843	3.077477	-5.571474
180	1	0	2.370251	4.563701	-4.623639
181	1	0	3.725817	3.439537	-4.463339
182	6	0	2.124464	3.735933	-2.203704
183	1	0	1.578877	3.405857	-1.315963
184	1	0	3.188879	3.774633	-1.949995
185	1	0	1.793750	4.755873	-2.426659
186	8	0	1.538550	1.216529	2.072959
187	6	0	1.694218	2.588374	1.884155
188	6	0	3.068821	2.904473	1.361205
189	6	0	4.022902	3.825131	1.669777
190	8	0	3.488746	2.145581	0.316317
191	6	0	5.099804	3.619376	0.743589
192	1	0	3.968795	4.566726	2.454271
193	6	0	4.716654	2.588769	-0.051303
194	1	0	6.030687	4.165231	0.684817
195	1	0	5.168529	2.086422	-0.893159
196	6	0	1.417511	3.328375	3.198494
197	1	0	2.265632	3.274482	3.883231
198	1	0	0.536401	2.895792	3.672853
199	8	0	1.203546	4.725155	2.936355
200	6	0	-0.051695	5.106911	2.680846
201	8	0	-1.025149	4.399495	2.878065
202	6	0	-0.135331	6.474478	2.109298
203	6	0	0.969671	7.078131	1.502448
204	6	0	-1.377001	7.115184	2.104160
205	6	0	0.826052	8.319940	0.891661
206	1	0	1.927614	6.569487	1.489521
207	6	0	-1.512625	8.360256	1.502859
208	1	0	-2.229997	6.630012	2.566848
209	6	0	-0.411708	8.960849	0.892372
210	1	0	1.680396	8.785808	0.410928
211	1	0	-2.476564	8.858918	1.502610
212	1	0	-0.520182	9.929270	0.413471
213	1	0	0.961122	2.976914	1.149897
214	1	0	-1.188724	1.337181	2.132801
215	1	0	-1.148540	1.874291	1.578250

TS2a(*S*) -1b

RwB97XD SCF energy	-5095.353354 a.u.
RwB97XD SCF enthalpy	-5093.398981 a.u.
RwB97XD SCF free energy	-5093.641841 a.u.
Three lowest frequencies (cm^{-1})	-1069, 12.1, 15.5
Imaginary frequency (cm^{-1})	-1069

Cartesian coordinates:
Standard orientation:

Center Number	Atomic Number	Atomic Type	Coordinates (Angstroms) X	Y	Z
1	6	0	0.915344	-1.938522	2.298039
2	6	0	0.703226	-2.314126	3.605760
3	6	0	1.003314	-1.493743	4.691319
4	6	0	1.524820	-0.235478	4.525345
5	6	0	1.741108	0.180810	3.203759

6	6	0	1.451788	-0.633567	2.111033
7	1	0	1.769241	0.400660	5.367424
8	1	0	2.164431	1.165670	3.047942
9	6	0	0.705584	-2.932732	1.212991
10	6	0	-0.185911	-2.814698	0.111328
11	6	0	1.463470	-4.084253	1.252361
12	6	0	-0.229811	-3.808623	-0.866457
13	6	0	1.414974	-5.064991	0.267702
14	6	0	0.580148	-4.952937	-0.816649
15	1	0	-0.920011	-3.720053	-1.696501
16	1	0	0.535453	-5.712096	-1.588463
17	8	0	0.706311	-2.146083	5.844894
18	8	0	0.200087	-3.494329	4.060213
19	8	0	2.387722	-4.432283	2.190180
20	8	0	2.306516	-6.049332	0.568700
21	6	0	0.479937	-3.504894	5.460360
22	1	0	-0.375511	-3.901502	6.004737
23	1	0	1.390060	-4.087802	5.644144
24	6	0	2.686717	-5.803368	1.924980
25	1	0	2.088433	-6.438986	2.588483
26	1	0	3.756020	-5.973260	2.045670
27	15	0	-1.198507	-1.302423	-0.081647
28	15	0	1.757984	-0.012138	0.431293
29	46	0	0.006699	0.453781	-0.933621
30	6	0	-1.956628	-0.978113	1.527928
31	6	0	-2.655757	-1.973451	2.204459
32	6	0	-1.839697	0.288915	2.090339
33	6	0	-3.212742	-1.744210	3.463820
34	1	0	-2.754624	-2.951383	1.743978
35	6	0	-2.375214	0.589272	3.342968
36	1	0	-1.303573	1.053309	1.537158
37	6	0	-2.973411	-0.478841	4.047973
38	6	0	-2.470141	-1.696033	-1.315504
39	6	0	-3.814819	-1.890414	-1.005607
40	6	0	-2.060267	-1.747764	-2.644771
41	6	0	-4.737633	-2.233340	-1.993691
42	1	0	-4.140749	-1.773412	0.019557
43	6	0	-2.932181	-2.078717	-3.684102
44	1	0	-1.023196	-1.527518	-2.875215
45	6	0	-4.250673	-2.413125	-3.311754
46	6	0	2.647663	1.560522	0.574656
47	6	0	3.954297	1.675356	0.120520
48	6	0	1.946375	2.697786	0.957560
49	6	0	4.597370	2.910224	0.073995
50	1	0	4.467573	0.789115	-0.223594
51	6	0	2.533405	3.963179	0.964053
52	1	0	0.904220	2.589916	1.234461
53	6	0	3.893057	4.035187	0.569019
54	6	0	2.885752	-1.152579	-0.390456
55	6	0	2.784493	-1.286237	-1.765857
56	6	0	3.900700	-1.813144	0.302158
57	6	0	3.724037	-2.005850	-2.508838
58	1	0	1.955307	-0.802277	-2.273577
59	6	0	4.890052	-2.511971	-0.382190
60	1	0	3.920797	-1.754836	1.383759
61	6	0	4.835535	-2.498302	-1.800522
62	6	0	3.404741	-2.202703	-4.007457
63	6	0	3.466904	-0.846927	-4.735148
64	1	0	3.231539	-0.981316	-5.796762
65	1	0	2.746682	-0.132631	-4.321149
66	1	0	4.463253	-0.398644	-4.664446
67	6	0	4.297324	-3.212519	-4.747821
68	1	0	4.340151	-4.173360	-4.228086
69	1	0	3.865016	-3.386308	-5.739195
70	1	0	5.316255	-2.855511	-4.900070
71	6	0	1.963192	-2.754836	-4.110430
72	1	0	1.872048	-3.713576	-3.588116
73	1	0	1.215616	-2.071400	-3.697202
74	1	0	1.709625	-2.915271	-5.163374
75	6	0	5.941089	-3.335405	0.389116
76	6	0	5.821208	-4.806855	-0.055542
77	1	0	6.075549	-4.931698	-1.110354
78	1	0	6.497183	-5.434699	0.535830
79	1	0	4.799585	-5.173083	0.091343
80	6	0	7.378964	-2.835221	0.158524
81	1	0	7.462607	-1.760940	0.355729
82	1	0	8.057282	-3.352490	0.846095
83	1	0	7.727403	-3.033072	-0.855246
84	6	0	5.682940	-3.286760	1.903969
85	1	0	5.812969	-2.278740	2.313040
86	1	0	4.680427	-3.639210	2.163459
87	1	0	6.404667	-3.938295	2.406519
88	8	0	5.916195	-2.999441	-2.482401
89	8	0	4.565805	5.231944	0.581483
90	8	0	-3.320023	-0.285673	5.357961
91	8	0	-5.131879	-2.898110	-4.246826
92	6	0	-4.968199	-4.286177	-4.527093
93	1	0	-5.782279	-4.567380	-5.197535
94	1	0	-4.013700	-4.490448	-5.019926
95	1	0	-5.023542	-4.883732	-3.611442
96	6	0	-2.364637	-2.019855	-5.124032
97	6	0	-3.432345	-1.902054	-6.226827
98	1	0	-2.928976	-1.685863	-7.175217
99	1	0	-4.009549	-2.814850	-6.369962
100	1	0	-4.128571	-1.082677	-6.023630
101	6	0	-1.482603	-3.255071	-5.384912
102	1	0	-0.999978	-3.170852	-6.365108
103	1	0	-0.697911	-3.344054	-4.626864
104	1	0	-2.060485	-4.183807	-5.376090
105	6	0	6.767744	-1.972949	-2.991014
106	1	0	7.603173	-2.474907	-3.482275
107	1	0	6.247279	-1.340261	-3.716863
108	1	0	7.146265	-1.338181	-2.182262
109	6	0	5.077471	5.661526	1.840795
110	1	0	5.967362	6.261380	1.637713
111	1	0	4.355448	6.288769	2.370634
112	1	0	5.350361	4.810739	2.472720
113	6	0	5.990146	3.005409	-0.586484
114	6	0	6.462026	1.633104	-1.107183
115	1	0	6.604245	0.908376	-0.297800
116	1	0	5.770438	1.202454	-1.840010
117	1	0	7.427892	1.759933	-1.606052
118	6	0	5.903911	3.946424	-1.804443
119	1	0	5.597674	4.953815	-1.516186
120	1	0	6.881942	4.014523	-2.293890
121	1	0	5.185564	3.564735	-2.538649
122	6	0	7.066955	3.509125	0.392253
123	1	0	6.947790	4.566626	0.624358
124	1	0	7.050956	2.941085	1.328574
125	1	0	8.057802	3.383373	-0.058128
126	6	0	1.645582	5.157383	1.388858
127	6	0	1.725931	5.328846	2.918533
128	1	0	1.380406	4.418628	3.421127
129	1	0	2.740098	5.534937	3.267740
130	1	0	1.083090	6.156695	3.238658
131	6	0	2.001641	6.469576	0.665560
132	1	0	1.238568	7.219160	0.899708
133	1	0	2.966464	6.882440	0.954610
134	1	0	2.005761	6.323044	-0.419858
135	6	0	0.165041	4.877828	1.044280
136	1	0	-0.425098	5.772953	1.264568
137	1	0	0.035440	4.642247	-0.016769
138	1	0	-0.263463	4.068495	1.640204
139	6	0	-6.243588	-2.319704	-1.661422
140	6	0	-1.476004	-0.762458	-5.266171
141	1	0	-2.031600	0.147239	-5.017405
142	1	0	-0.576971	-0.795798	-4.644899
143	1	0	-1.136179	-0.679646	-6.303690
144	6	0	-6.990697	-1.261460	-2.496521
145	1	0	-8.059317	-1.277950	-2.253434
146	1	0	-6.607818	-0.258520	-2.281607
147	1	0	-6.883306	-1.447953	-3.567084
148	6	0	-6.842318	-3.713183	-1.935910
149	1	0	-6.223745	-4.505735	-1.501445
150	1	0	-7.834657	-3.778703	-1.476519
151	1	0	-6.964576	-3.907021	-3.001512
152	6	0	-6.504668	-2.014056	-0.178357
153	1	0	-6.131956	-1.029410	0.117729
154	1	0	-7.584096	-2.024342	0.002527
155	1	0	-6.055426	-2.769133	0.473363
156	6	0	-2.264044	-0.617333	6.251330
157	1	0	-2.644028	-0.456426	7.261970
158	1	0	-1.963015	-1.665306	6.134391
159	1	0	-1.388906	0.022894	6.083888
160	6	0	-4.041142	-2.893126	4.088756
161	6	0	-3.108895	-4.061278	4.452679
162	1	0	-3.697126	-4.905901	4.828724
163	1	0	-2.531488	-4.409018	3.590665
164	1	0	-2.408378	-3.764528	5.238393
165	6	0	-4.860975	-2.525503	5.337928
166	1	0	-5.518810	-3.369804	5.573106
167	1	0	-4.240293	-2.342635	6.215761
168	1	0	-5.490499	-1.647470	5.171611
169	6	0	-5.063911	-3.374894	3.036520

170	1	0	-5.739106	-2.561120	2.751830
171	1	0	-4.592503	-3.762018	2.129520
172	1	0	-5.670126	-4.184280	3.456519
173	6	0	-2.275355	2.060242	3.820640
174	6	0	-0.804044	2.418932	4.089999
175	1	0	-0.726714	3.470167	4.389373
176	1	0	-0.388290	1.805034	4.894727
177	1	0	-0.182795	2.276886	3.200615
178	6	0	-3.090232	2.413282	5.076888
179	1	0	-2.683741	1.967754	5.985722
180	1	0	-3.059735	3.501214	5.205038
181	1	0	-4.138332	2.116870	4.984806
182	6	0	-2.810543	2.974396	2.695569
183	1	0	-2.271954	2.853908	1.753167
184	1	0	-3.870607	2.778700	2.502205
185	1	0	-2.710393	4.022316	2.998010
186	8	0	-1.233347	1.327762	-2.494758
187	6	0	-1.945386	2.467259	-2.068523
188	6	0	-3.273738	2.109026	-1.483581
189	6	0	-4.571016	2.326222	-1.829920
190	8	0	-3.223171	1.444641	-0.298259
191	6	0	-5.369758	1.757224	-0.782519
192	1	0	-4.923028	2.841911	-2.712135
193	6	0	-4.497163	1.231197	0.113072
194	1	0	-6.447896	1.745544	-0.711086
195	1	0	-4.611350	0.707165	1.050087
196	6	0	-2.081264	3.429271	-3.249809
197	1	0	-2.862388	3.120888	-3.946181
198	1	0	-1.127939	3.487976	-3.776165
199	8	0	-2.461759	4.727596	-2.774115
200	6	0	-1.473428	5.510522	-2.323283
201	8	0	-0.292940	5.231950	-2.442324
202	6	0	-1.971224	6.737766	-1.651930
203	6	0	-3.332728	6.946935	-1.412166
204	6	0	-1.030891	7.677692	-1.220094
205	6	0	-3.746028	8.094576	-0.743864
206	1	0	-4.060980	6.212999	-1.738781
207	6	0	-1.448762	8.821982	-0.551741
208	1	0	0.022621	7.502360	-1.408864
209	6	0	-2.806771	9.030375	-0.313430
210	1	0	-4.802421	8.257811	-0.555756
211	1	0	-0.717144	9.550154	-0.216303
212	1	0	-3.134028	9.923953	0.209502
213	1	0	-1.375549	2.995388	-1.283558
214	1	0	0.905748	1.726159	-1.788672
215	1	0	0.109778	1.681980	-2.262384

6b(S)•2a

RwB97XD SCF energy -5095.412238 a.u.

RwB97XD SCF enthalpy -5093.451140 a.u.

RwB97XD SCF free energy -5093.692322 a.u.

Three lowest frequencies (cm^{-1}) 11.3, 15.0, 18.5

Cartesian coordinates:

Center Number	Atomic Number	Atomic Type	Coordinates (Angstroms) X	Y	Z
1	6	0	0.154338	1.387441	2.506835
2	6	0	-0.147877	2.474149	3.299758
3	6	0	0.345678	3.754395	3.071446
4	6	0	1.184270	4.021968	2.017713
5	6	0	1.539717	2.929434	1.213138
6	6	0	1.060691	1.638269	1.439496
7	1	0	1.576502	5.014569	1.831090
8	1	0	2.228223	3.110506	0.396009
9	6	0	-0.434835	0.076463	2.897602
10	6	0	-1.399412	-0.666997	2.163170
11	6	0	-0.088956	-0.436902	4.129938
12	6	0	-1.900904	-1.862632	2.677318
13	6	0	-0.600651	-1.626501	4.633983
14	6	0	-1.506003	-2.375181	3.923127
15	1	0	-2.632558	-2.425205	2.108629
16	1	0	-1.911361	-3.303843	4.307375
17	8	0	-0.137645	4.598229	4.027531
18	8	0	-0.951759	2.493753	4.401847
19	8	0	0.790338	0.091746	5.028484
20	8	0	-0.052614	-1.868541	5.861021
21	6	0	-0.639990	3.727491	5.044666
22	1	0	-1.538047	4.152141	5.487859
23	1	0	0.143977	3.556902	5.793292
24	6	0	0.557993	-0.632425	6.235950
25	1	0	-0.131914	-0.063474	6.871394
26	1	0	1.507585	-0.823662	6.734519
27	15	0	-1.857819	-0.139145	0.467045
28	15	0	1.567639	0.305135	0.291001
29	46	0	-0.053145	-0.367684	-1.097186
30	6	0	-2.378472	1.598510	0.547783
31	6	0	-3.220236	2.137291	1.516898
32	6	0	-1.900077	2.419386	-0.467004
33	6	0	-3.556073	3.494216	1.516848
34	1	0	-3.611154	1.486165	2.292620
35	6	0	-2.166748	3.786609	-0.512506
36	1	0	-1.265261	1.977287	-1.228916
37	6	0	-2.931101	4.314690	0.547440
38	6	0	-3.317773	-1.129590	0.018211
39	6	0	-4.621633	-0.639534	0.034785
40	6	0	-3.084950	-2.418083	-0.447822
41	6	0	-5.704516	-1.448808	-0.311274
42	1	0	-4.790093	0.389419	0.324417
43	6	0	-4.118286	-3.275733	-0.833410
44	1	0	-2.059060	-2.762647	-0.521578
45	6	0	-5.432266	-2.795253	-0.655293
46	6	0	3.066221	0.941028	-0.530206
47	6	0	4.335823	0.710652	-0.015610
48	6	0	2.933184	1.652058	-1.715168
49	6	0	5.478618	1.205130	-0.641364
50	1	0	4.437107	0.122780	0.884274
51	6	0	4.028926	2.193287	-2.391096
52	1	0	1.939449	1.777481	-2.125786
53	6	0	5.300151	2.001149	-1.799182
54	6	0	2.117968	-1.107184	1.287440
55	6	0	1.802125	-2.380885	0.838416
56	6	0	2.881141	-0.959487	2.447847
57	6	0	2.290172	-3.533200	1.461189
58	1	0	1.152368	-2.479887	-0.025425
59	6	0	3.452751	-2.062782	3.076330
60	1	0	3.040000	0.036135	2.844467
61	6	0	3.221234	-3.336792	2.496705
62	6	0	1.718849	-4.880887	0.965347
63	6	0	2.204830	-5.164908	-0.467667
64	1	0	1.826788	-6.136446	-0.805929
65	1	0	1.842150	-4.408269	-1.171284
66	1	0	3.297699	-5.188382	-0.529831
67	6	0	2.030945	-6.093823	1.858988
68	1	0	1.780535	-5.900193	2.905576
69	1	0	1.419121	-6.936139	1.518226
70	1	0	3.072754	-6.412005	1.808508
71	6	0	0.177276	-4.761470	0.940276
72	1	0	-0.220128	-4.554233	1.940033
73	1	0	-0.177518	-3.976267	0.266223
74	1	0	-0.251524	-5.706461	0.591700
75	6	0	4.232857	-1.898112	4.396946
76	6	0	3.544596	-2.750880	5.481251
77	1	0	3.620937	-3.817214	5.258688
78	1	0	4.014041	-2.573195	6.455400
79	1	0	2.482425	-2.499889	5.560748
80	6	0	5.709029	-2.318692	4.269437
81	1	0	6.196989	-1.810641	3.430615
82	1	0	6.245655	-2.040380	5.183420
83	1	0	5.821727	-3.394886	4.138703
84	6	0	4.223702	-0.435498	4.871095
85	1	0	4.758155	0.223728	4.177603
86	1	0	3.209482	-0.048508	5.004951
87	1	0	4.734692	-0.372750	5.837018
88	8	0	3.931207	-4.402546	2.993482
89	8	0	6.431346	2.535563	-2.367827
90	8	0	-3.063757	5.675470	0.649796
91	8	0	-6.510808	-3.626276	-0.848260
92	6	0	-6.775870	-4.498476	0.246657
93	1	0	-7.762719	-4.931750	0.072058
94	1	0	-6.041909	-5.306806	0.309013
95	1	0	-6.779390	-3.954574	1.196989
96	6	0	-3.705884	-4.639042	-1.438871
97	6	0	-4.792269	-5.310775	-2.297768
98	1	0	-4.346543	-6.167749	-2.814125
99	1	0	-5.635396	-5.685048	-1.718471
100	1	0	-5.177827	-4.624931	-3.058764
101	6	0	-3.261156	-5.589991	-0.313355
102	1	0	-2.853858	-6.515074	-0.737213
103	1	0	-2.482651	-5.126265	0.299945
104	1	0	-4.087956	-5.860632	0.349408
105	6	0	5.008487	-4.791094	2.144110
106	1	0	5.527197	-5.608267	2.648807

107	1	0	4.651113	-5.139138	1.169738
108	1	0	5.704879	-3.960527	1.983936
109	6	0	6.689169	3.915083	-2.118340
110	1	0	7.772444	4.038038	-2.044840
111	1	0	6.332659	4.538815	-2.943105
112	1	0	6.224030	4.246824	-1.185424
113	6	0	6.866942	0.832458	-0.075927
114	6	0	6.747264	-0.128397	1.122177
115	1	0	6.240713	0.333499	1.976913
116	1	0	6.219778	-1.052797	0.863184
117	1	0	7.753346	-0.406850	1.451539
118	6	0	7.696832	0.104842	-1.150858
119	1	0	7.804732	0.704935	-2.055725
120	1	0	8.698064	-0.110660	-0.760433
121	1	0	7.229519	-0.849207	-1.415905
122	6	0	7.628879	2.073531	0.425000
123	1	0	7.976768	2.704878	-0.391602
124	1	0	7.005676	2.679297	1.091633
125	1	0	8.512474	1.756558	0.989954
126	6	0	3.743922	2.954473	-3.707977
127	6	0	3.493511	4.442110	-3.393793
128	1	0	2.660884	4.548325	-2.690563
129	1	0	4.363682	4.932106	-2.953051
130	1	0	3.232326	4.981363	-4.311601
131	6	0	4.861453	2.798192	-4.755557
132	1	0	4.531284	3.248258	-5.697644
133	1	0	5.796905	3.276953	-4.472503
134	1	0	5.067446	1.739990	-4.943746
135	6	0	2.459230	2.413951	-4.377288
136	1	0	2.356817	2.867907	-5.368000
137	1	0	2.494302	1.327275	-4.507209
138	1	0	1.550033	2.669293	-3.824175
139	6	0	-7.130575	-0.857990	-0.368347
140	6	0	-2.500364	-4.423068	-2.384851
141	1	0	-2.729259	-3.670691	-3.147011
142	1	0	-1.591273	-4.119493	-1.857722
143	1	0	-2.268202	-5.363576	-2.894515
144	6	0	-7.711363	-1.049420	-1.782586
145	1	0	-8.710245	-0.602077	-1.838635
146	1	0	-7.078811	-0.560055	-2.529363
147	1	0	-7.796047	-2.105664	-2.044008
148	6	0	-8.071892	-1.501723	0.667518
149	1	0	-7.627067	-1.490979	1.668390
150	1	0	-9.007722	-0.933259	0.713018
151	1	0	-8.327642	-2.529535	0.410007
152	6	0	-7.117100	0.651091	-0.076022
153	1	0	-6.466994	1.205768	-0.760664
154	1	0	-8.130707	1.047243	-0.193597
155	1	0	-6.801437	0.861842	0.950163
156	6	0	-2.041696	6.264774	1.443894
157	1	0	-2.243318	7.337434	1.474778
158	1	0	-2.048446	5.863883	2.464121
159	1	0	-1.051006	6.096187	1.003906
160	6	0	-4.581926	3.970317	2.573927
161	6	0	-3.936087	3.919091	3.967785
162	1	0	-4.669341	4.192426	4.734836
163	1	0	-3.551080	2.922612	4.202340
164	1	0	-3.107283	4.631024	4.025692
165	6	0	-5.156755	5.382480	2.364073
166	1	0	-5.979679	5.522136	3.074262
167	1	0	-4.427291	6.170589	2.552559
168	1	0	-5.559201	5.517695	1.356303
169	6	0	-5.794415	3.014576	2.539489
170	1	0	-6.279213	3.042911	1.558221
171	1	0	-5.532162	1.976938	2.761449
172	1	0	-6.530693	3.329141	3.286659
173	6	0	-1.577001	4.563116	-1.713139
174	6	0	-0.043088	4.608777	-1.579989
175	1	0	0.389407	5.121929	-2.446228
176	1	0	0.262787	5.149143	-0.678046
177	1	0	0.387889	3.602543	-1.530928
178	6	0	-2.094642	5.999077	-1.898800
179	1	0	-1.750870	6.681744	-1.120737
180	1	0	-1.715753	6.378242	-2.854724
181	1	0	-3.186762	6.038232	-1.933321
182	6	0	-1.935964	3.802030	-3.010484
183	1	0	-1.491564	2.804043	-3.054358
184	1	0	-3.020533	3.693787	-3.119287
185	1	0	-1.561657	4.358424	-3.876494
186	8	0	-1.305523	-1.205708	-2.659455
187	6	0	-1.460909	-0.453426	-3.883875
188	6	0	-2.911975	-0.203319	-4.086465
189	6	0	-3.869316	-0.684128	-4.921061
190	8	0	-3.459671	0.677440	-3.203983
191	6	0	-5.093652	-0.048785	-4.531088
192	1	0	-3.726441	-1.401887	-5.716698
193	6	0	-4.783604	0.758253	-3.484845
194	1	0	-6.069576	-0.179499	-4.975815
195	1	0	-5.357571	1.432749	-2.867697
196	6	0	-0.806938	-1.169598	-5.053775
197	1	0	-1.013635	-0.632788	-5.979079
198	1	0	-1.154201	-2.202428	-5.145298
199	8	0	0.624395	-1.134660	-4.915747
200	6	0	1.223552	-2.036057	-4.134334
201	8	0	0.619562	-2.914507	-3.533510
202	1	0	-0.953114	0.505013	-3.733457
203	1	0	1.175241	-0.480254	-2.044257
204	1	0	-0.908189	-2.071812	-2.869001
205	6	0	2.685163	-1.810237	-4.041650
206	6	0	3.330697	-2.123289	-2.841176
207	6	0	3.398973	-1.237398	-5.097588
208	6	0	4.683157	-1.841528	-2.690583
209	1	0	2.767106	-2.553305	-2.019928
210	6	0	4.759642	-0.990810	-4.952062
211	1	0	2.894682	-0.996577	-6.027284
212	6	0	5.397332	-1.275743	-3.745825
213	1	0	5.177537	-2.060217	-1.749231
214	1	0	5.320668	-0.565290	-5.777663
215	1	0	6.454375	-1.058782	-3.629166

2.5. Catalytic cycle 2a + 1b (*R*)

3a(*R*) -1b

RwB97XD SCF energy -5094.188846 a.u.
RwB97XD SCF enthalpy -5092.251307 a.u.
RwB97XD SCF free energy -5092.493311 a.u.
Three lowest frequencies (cm^{-1}) 10.0, 13.8, 17.7
Cartesian coordinates:

Center Number	Atomic Number	Atomic Type	Coordinates (Angstroms) X	Y	Z
1	6	0	-0.901914	0.449851	2.610320
2	6	0	-0.473916	0.111580	3.875940
3	6	0	-0.751608	-1.108679	4.482095
4	6	0	-1.514084	-2.064041	3.856450
5	6	0	-1.984054	-1.742467	2.575559
6	6	0	-1.700570	-0.525880	1.952157
7	1	0	-1.744688	-3.013445	4.324762
8	1	0	-2.584500	-2.482563	2.058822
9	6	0	-0.515686	1.792773	2.095945
10	6	0	0.405865	2.038016	1.042371
11	6	0	-1.011519	2.907099	2.736011
12	6	0	0.721762	3.344597	0.676784
13	6	0	-0.674852	4.205660	2.373611
14	6	0	0.179432	4.462529	1.330230
15	1	0	1.421154	3.521163	-0.131741
16	1	0	0.437897	5.473089	1.036329
17	8	0	-0.199103	-1.133196	5.725441
18	8	0	0.250429	0.887130	4.729659
19	8	0	-1.894540	2.939238	3.774712
20	8	0	-1.328723	5.085992	3.183231
21	6	0	0.669369	0.001929	5.766194
22	1	0	1.689723	-0.325872	5.555417
23	1	0	0.581221	0.498246	6.731910
24	6	0	-1.896524	4.293528	4.229005
25	1	0	-1.273993	4.376222	5.127318
26	1	0	-2.922439	4.614141	4.412840
27	15	0	1.147152	0.608872	0.168801
28	15	0	-2.179609	-0.345381	0.195130
29	46	0	-0.381154	-0.864900	-1.034400
30	6	0	2.045689	-0.284327	1.484333
31	6	0	2.778861	0.351656	2.488550
32	6	0	2.025254	-1.669102	1.435805
33	6	0	3.484847	-0.369852	3.452246
34	1	0	2.787363	1.433847	2.511520
35	6	0	2.698767	-2.462442	2.370870
36	1	0	1.457589	-2.148069	0.644383
37	6	0	3.370110	-1.785919	3.409131
38	6	0	2.408778	1.341431	-0.922578
39	6	0	3.566320	1.949262	-0.431917
40	6	0	2.204835	1.284477	-2.294442
41	6	0	4.470848	2.583387	-1.276403
42	1	0	3.747584	1.937783	0.634097
43	6	0	3.097590	1.861129	-3.208619
44	1	0	1.326686	0.766719	-2.665925
45	6	0	4.169069	2.593380	-2.663015
46	6	0	-3.653688	-1.409363	-0.004897
47	6	0	-4.938477	-0.909613	0.183352
48	6	0	-3.496314	-2.740051	-0.385489
49	6	0	-6.066489	-1.727298	0.094094
50	1	0	-5.070767	0.142532	0.385367
51	6	0	-4.577620	-3.614627	-0.483041
52	1	0	-2.500679	-3.092993	-0.618691
53	6	0	-5.843019	-3.104902	-0.114466
54	6	0	-2.749767	1.350768	-0.097883
55	6	0	-2.326771	1.994142	-1.251111
56	6	0	-3.622363	2.007339	0.773388
57	6	0	-2.811305	3.253963	-1.620661
58	1	0	-1.585108	1.502476	-1.874072
59	6	0	-4.197157	3.227553	0.431698
60	1	0	-3.860875	1.540160	1.721037
61	6	0	-3.843604	3.783166	-0.825169
62	6	0	-2.126448	3.932210	-2.831419
63	6	0	-2.428215	3.137510	-4.115320
64	1	0	-1.926025	3.602353	-4.971174
65	1	0	-2.075928	2.102675	-4.042767
66	1	0	-3.502100	3.112968	-4.328027
67	6	0	-2.488483	5.410494	-3.059340
68	1	0	-2.366639	6.001114	-2.147661
69	1	0	-1.805347	5.814821	-3.814491
70	1	0	-3.502293	5.556883	-3.433629
71	6	0	-0.601754	3.914999	-2.577520
72	1	0	-0.354452	4.496906	-1.684150
73	1	0	-0.199333	2.907170	-2.446341
74	1	0	-0.082983	4.365759	-3.429874
75	6	0	-5.122023	3.964305	1.423308
76	6	0	-4.508065	5.341237	1.742191
77	1	0	-4.453639	5.969795	0.850644
78	1	0	-5.120456	5.861245	2.487769
79	1	0	-3.496875	5.234933	2.146053
80	6	0	-6.547980	4.155657	0.871367
81	1	0	-6.957946	3.217131	0.483877
82	1	0	-7.206826	4.496569	1.677853
83	1	0	-6.583622	4.906709	0.082187
84	6	0	-5.249503	3.189415	2.745190
85	1	0	-5.745294	2.221437	2.609072
86	1	0	-4.279234	3.016469	3.219436
87	1	0	-5.860229	3.774464	3.439928
88	8	0	-4.537748	4.889202	-1.249952
89	8	0	-6.902879	-3.976643	-0.004839
90	8	0	3.966207	-2.512498	4.412414
91	8	0	4.989038	3.331658	-3.481731
92	6	0	4.427170	4.582640	-3.869960
93	1	0	5.182580	5.091892	-4.471061
94	1	0	3.520407	4.451133	-4.467308
95	1	0	4.180745	5.194635	-2.995857
96	6	0	2.828427	1.600539	-4.711621
97	6	0	4.019969	1.878008	-5.646274
98	1	0	3.780061	1.475712	-6.636315
99	1	0	4.233024	2.938769	-5.775848
100	1	0	4.930196	1.382412	-5.296955
101	6	0	1.607329	2.416202	-5.175141
102	1	0	1.400132	2.213025	-6.232011
103	1	0	0.714232	2.148283	-4.600820
104	1	0	1.768488	3.492693	-5.065015
105	6	0	-5.519575	4.582333	-2.237005
106	1	0	-6.018042	5.520995	-2.485190
107	1	0	-5.067533	4.161867	-3.140968
108	1	0	-6.255325	3.866946	-1.853134
109	6	0	-7.032418	-4.509784	1.316624
110	1	0	-7.852702	-5.229011	1.284002
111	1	0	-6.116854	-5.015180	1.634641
112	1	0	-7.263036	-3.726195	2.042987
113	6	0	-7.474519	-1.089484	0.061254
114	6	0	-7.388148	0.445746	0.137442
115	1	0	-6.980099	0.789899	1.094318
116	1	0	-6.780816	0.867822	-0.670233
117	1	0	-8.395419	0.863119	0.042349
118	6	0	-8.125324	-1.448586	-1.289672
119	1	0	-8.254401	-2.528165	-1.397342
120	1	0	-9.112152	-0.978657	-1.367103
121	1	0	-7.514333	-1.088732	-2.124674
122	6	0	-8.395783	-1.546418	1.205924
123	1	0	-8.720578	-2.580358	1.083293
124	1	0	-7.908482	-1.439835	2.180860
125	1	0	-9.297277	-0.923625	1.216003
126	6	0	-4.387807	-4.999886	-1.143463
127	6	0	-2.929732	-5.208417	-1.591616
128	1	0	-2.593854	-4.436297	-2.292657
129	1	0	-2.234226	-5.234892	-0.745150
130	1	0	-2.852922	-6.170545	-2.107506
131	6	0	-4.755516	-6.187928	-0.236545
132	1	0	-4.426846	-7.118834	-0.711123
133	1	0	-4.259743	-6.118400	0.737407

134	1	0	-5.831853	-6.268069	-0.080264
135	6	0	-5.267234	-5.039173	-2.410049
136	1	0	-5.143262	-6.001165	-2.920213
137	1	0	-6.325590	-4.918395	-2.166821
138	1	0	-4.980133	-4.246380	-3.109542
139	6	0	5.788233	3.157272	-0.710873
140	6	0	2.492367	0.102634	-4.890263
141	1	0	3.278160	-0.532896	-4.468652
142	1	0	1.541976	-0.161394	-4.422319
143	1	0	2.396941	-0.129408	-5.955888
144	6	0	6.959733	2.357976	-1.314158
145	1	0	7.911355	2.724578	-0.912558
146	1	0	6.873783	1.294402	-1.065911
147	1	0	6.989259	2.455625	-2.402289
148	6	0	5.977521	4.656664	-1.011388
149	1	0	5.093052	5.235572	-0.724052
150	1	0	6.825303	5.037097	-0.431220
151	1	0	6.193355	4.843643	-2.062939
152	6	0	5.853779	3.006664	0.818643
153	1	0	5.793934	1.962846	1.139223
154	1	0	6.810819	3.398712	1.176655
155	1	0	5.058509	3.570201	1.319458
156	6	0	3.069170	-2.918380	5.443110
157	1	0	3.593416	-3.664734	6.043179
158	1	0	2.794901	-2.077551	6.087012
159	1	0	2.154174	-3.356452	5.033103
160	6	0	4.469962	0.378790	4.389931
161	6	0	4.434984	-0.018763	5.878027
162	1	0	5.135330	0.623385	6.422701
163	1	0	3.451470	0.133187	6.330485
164	1	0	4.745944	-1.049858	6.038234
165	6	0	5.893618	0.095714	3.868255
166	1	0	6.634133	0.576643	4.517522
167	1	0	6.103645	-0.977882	3.848788
168	1	0	6.026848	0.494914	2.858860
169	6	0	4.234692	1.898562	4.339792
170	1	0	4.424288	2.322581	3.349000
171	1	0	3.212796	2.158021	4.639586
172	1	0	4.923201	2.390430	5.033612
173	6	0	2.611762	-3.992425	2.138814
174	6	0	1.159318	-4.442333	2.392319
175	1	0	1.063104	-5.520769	2.222301
176	1	0	0.851501	-4.233603	3.422226
177	1	0	0.458146	-3.933019	1.721982
178	6	0	3.559687	-4.863403	2.981346
179	1	0	3.280303	-4.917060	4.033344
180	1	0	3.519521	-5.885496	2.588571
181	1	0	4.594791	-4.519265	2.911386
182	6	0	2.982311	-4.299463	0.669357
183	1	0	2.383995	-3.747874	-0.059983
184	1	0	4.036473	-4.071574	0.486953
185	1	0	2.830216	-5.366584	0.474987
186	8	0	1.171830	-1.811119	-2.205221
187	6	0	1.263692	-2.527229	-3.207078
188	1	0	-1.424544	-1.770516	-1.725409
189	6	0	0.206517	-2.721834	-4.164531
190	6	0	-0.985887	-2.085612	-4.410751
191	8	0	0.411784	-3.692177	-5.106837
192	6	0	-1.543440	-2.713323	-5.551797
193	1	0	-1.393352	-1.256596	-3.851248
194	6	0	-0.653843	-3.680256	-5.922323
195	1	0	-2.478381	-2.480345	-6.039317
196	1	0	-0.650395	-4.411456	-6.717335
197	6	0	2.573302	-3.242160	-3.467765
198	1	0	2.880151	-3.123555	-4.510982
199	1	0	2.447254	-4.312255	-3.264385
200	8	0	3.517447	-2.659974	-2.596327
201	6	0	4.651154	-3.323999	-2.344815
202	8	0	4.949514	-4.364965	-2.895638
203	6	0	5.447646	-2.637404	-1.298181
204	6	0	6.660896	-3.200339	-0.896341
205	6	0	4.952722	-1.497410	-0.657392
206	6	0	7.377663	-2.623361	0.146277
207	1	0	7.029210	-4.091207	-1.394664
208	6	0	5.663796	-0.937995	0.394660
209	1	0	4.009594	-1.060833	-0.965733
210	6	0	6.876608	-1.496166	0.795828
211	1	0	8.320957	-3.058734	0.460039
212	1	0	5.257513	-0.071920	0.904594
213	1	0	7.429359	-1.054584	1.619342

TS1a(*R*) -1b

RwB97XD SCF energy -5094.176478 a.u.
RwB97XD SCF enthalpy -5092.242672 a.u.
RwB97XD SCF free energy -5092.484306 a.u.
Three lowest frequencies (cm^{-1}) -618.9, 9.0, 11.4
Imaginary frequency (cm^{-1}) -618.9
Cartesian coordinates:

Center Number	Atomic Number	Atomic Type	Coordinates (Angstroms) X	Y	Z
1	6	0	0.759524	1.001259	2.450487
2	6	0	0.626386	1.993481	3.397272
3	6	0	1.203220	3.252507	3.273738
4	6	0	1.942567	3.597290	2.169516
5	6	0	2.101445	2.605148	1.190839
6	6	0	1.537356	1.334575	1.307650
7	1	0	2.394320	4.576001	2.060695
8	1	0	2.697094	2.851850	0.320566
9	6	0	0.134982	-0.315455	2.743568
10	6	0	-0.909030	-0.936755	2.008358
11	6	0	0.553628	-0.986140	3.873906
12	6	0	-1.414575	-2.173612	2.410037
13	6	0	0.038120	-2.214930	4.269441
14	6	0	-0.944006	-2.847763	3.546938
15	1	0	-2.211628	-2.638209	1.840278
16	1	0	-1.351687	-3.806266	3.845538
17	8	0	0.890332	3.999672	4.369249
18	8	0	-0.084053	1.936789	4.560203
19	8	0	1.513926	-0.596739	4.761259
20	8	0	0.662970	-2.614565	5.415343
21	6	0	0.366874	3.059696	5.313646
22	1	0	-0.469773	3.502214	5.851183
23	1	0	1.169463	2.745986	5.991944
24	6	0	1.305562	-1.432936	5.898067
25	1	0	0.640070	-0.922312	6.605372
26	1	0	2.263376	-1.687805	6.348094
27	15	0	-1.511692	-0.187916	0.450796
28	15	0	1.794677	0.125095	-0.044881
29	46	0	0.008238	-0.533431	-1.303969
30	6	0	-1.736483	1.588325	0.768570
31	6	0	-2.392593	2.057908	1.906137
32	6	0	-1.251437	2.497747	-0.161489
33	6	0	-2.549236	3.420646	2.143011
34	1	0	-2.763476	1.337922	2.624426
35	6	0	-1.387760	3.880660	-0.001072
36	1	0	-0.727074	2.117028	-1.032655
37	6	0	-1.973166	4.316279	1.204620
38	6	0	-3.154928	-0.922153	0.176238
39	6	0	-4.327037	-0.337851	0.659287
40	6	0	-3.235624	-2.118619	-0.526078
41	6	0	-5.552393	-0.990951	0.572507
42	1	0	-4.277836	0.639472	1.117284
43	6	0	-4.443348	-2.812481	-0.674103
44	1	0	-2.334635	-2.527411	-0.968371
45	6	0	-5.557500	-2.287709	0.004506
46	6	0	3.151596	0.848921	-1.007257
47	6	0	4.477300	0.633443	-0.647879
48	6	0	2.853801	1.675384	-2.087781
49	6	0	5.522065	1.310382	-1.275846
50	1	0	4.698418	-0.081424	0.131787
51	6	0	3.849292	2.410464	-2.735651
52	1	0	1.821980	1.749591	-2.412679
53	6	0	5.160841	2.296606	-2.220457
54	6	0	2.433063	-1.373960	0.758563
55	6	0	1.993075	-2.611301	0.311174
56	6	0	3.323041	-1.310557	1.830941
57	6	0	2.442870	-3.810677	0.870166
58	1	0	1.258329	-2.641222	-0.485858

59	6	0	3.871526	-2.465680	2.380878
60	1	0	3.591293	-0.339960	2.228914
61	6	0	3.469986	-3.709873	1.830025
62	6	0	1.764470	-5.114220	0.390812
63	6	0	2.407447	-5.576630	-0.928397
64	1	0	1.881057	-6.454429	-1.320336
65	1	0	2.350330	-4.783967	-1.680071
66	1	0	3.460476	-5.841264	-0.804850
67	6	0	1.796943	-6.243771	1.436356
68	1	0	1.415824	-5.895036	2.402118
69	1	0	1.147478	-7.057055	1.095689
70	1	0	2.788859	-6.663872	1.595503
71	6	0	0.265650	-4.845428	0.114249
72	1	0	-0.235369	-4.434135	0.997180
73	1	0	0.097986	-4.162001	-0.722516
74	1	0	-0.223637	-5.789454	-0.145789
75	6	0	4.846347	-2.368344	3.572682
76	6	0	4.347058	-3.253623	4.732425
77	1	0	4.450666	-4.315048	4.506297
78	1	0	4.918910	-3.039738	5.642052
79	1	0	3.290112	-3.059726	4.937168
80	6	0	6.270720	-2.786137	3.160839
81	1	0	6.606642	-2.224028	2.282308
82	1	0	6.968252	-2.574465	3.979043
83	1	0	6.343633	-3.850531	2.936769
84	6	0	4.936277	-0.926044	4.101594
85	1	0	5.367635	-0.239535	3.364681
86	1	0	3.959838	-0.538567	4.408219
87	1	0	5.592119	-0.910538	4.977748
88	8	0	4.093979	-4.833604	2.310454
89	8	0	6.133887	3.148688	-2.690169
90	8	0	-2.021678	5.656774	1.496694
91	8	0	-6.702140	-3.037785	0.132672
92	6	0	-6.591773	-4.020827	1.159687
93	1	0	-7.549406	-4.542892	1.199635
94	1	0	-5.793591	-4.739914	0.946995
95	1	0	-6.390224	-3.555105	2.130726
96	6	0	-4.429989	-4.040766	-1.614598
97	6	0	-5.803118	-4.657818	-1.929478
98	1	0	-5.667644	-5.402140	-2.722173
99	1	0	-6.252637	-5.173910	-1.080360
100	1	0	-6.511466	-3.909229	-2.293818
101	6	0	-3.514525	-5.132546	-1.032420
102	1	0	-3.453525	-5.980505	-1.723891
103	1	0	-2.497331	-4.761150	-0.870235
104	1	0	-3.895091	-5.504521	-0.074945
105	6	0	5.076171	-5.411918	1.454497
106	1	0	5.812139	-5.899856	2.097733
107	1	0	4.637679	-6.167487	0.796219
108	1	0	5.573525	-4.649903	0.846270
109	6	0	6.252411	4.328440	-1.888794
110	1	0	7.033963	4.937541	-2.346124
111	1	0	5.315424	4.890836	-1.866672
112	1	0	6.530167	4.086899	-0.859798
113	6	0	6.980648	0.855492	-1.042464
114	6	0	7.041696	-0.319150	-0.048649
115	1	0	6.701168	-0.028475	0.951588
116	1	0	6.450250	-1.178555	-0.381150
117	1	0	8.079687	-0.653258	0.043136
118	6	0	7.525874	0.352785	-2.395040
119	1	0	7.540588	1.152471	-3.140141
120	1	0	8.549568	-0.018682	-2.272481
121	1	0	6.912521	-0.468042	-2.782304
122	6	0	7.915728	1.950444	-0.498852
123	1	0	8.130372	2.716490	-1.244867
124	1	0	7.500901	2.428500	0.394689
125	1	0	8.871332	1.495314	-0.217248
126	6	0	3.527009	3.185677	-4.033131
127	6	0	2.059958	2.986175	-4.446397
128	1	0	1.820616	1.928252	-4.587222
129	1	0	1.358150	3.410681	-3.720922
130	1	0	1.888860	3.488528	-5.403314
131	6	0	3.760881	4.703663	-3.924807
132	1	0	3.315219	5.199069	-4.794157
133	1	0	3.289276	5.119598	-3.028027
134	1	0	4.821655	4.957852	-3.917551
135	6	0	4.404193	2.612741	-5.164699
136	1	0	4.177836	3.124064	-6.107056
137	1	0	5.467585	2.746613	-4.954138
138	1	0	4.211474	1.543478	-5.305926
139	6	0	-6.848700	-0.289613	1.029026
140	6	0	-3.856786	-3.569114	-2.971151
141	1	0	-4.479124	-2.776437	-3.400550
142	1	0	-2.838064	-3.186781	-2.887864
143	1	0	-3.840871	-4.406506	-3.677506
144	6	0	-7.863742	-0.291168	-0.130819
145	1	0	-8.773241	0.241104	0.170157
146	1	0	-7.449359	0.215474	-1.008130
147	1	0	-8.143891	-1.305134	-0.421612
148	6	0	-7.469746	-0.967093	2.264566
149	1	0	-6.728379	-1.093399	3.061203
150	1	0	-8.278272	-0.340637	2.657962
151	1	0	-7.896874	-1.942097	2.026245
152	6	0	-6.580332	1.179013	1.398497
153	1	0	-6.112569	1.729598	0.575927
154	1	0	-7.531155	1.670078	1.627721
155	1	0	-5.945258	1.270550	2.285352
156	6	0	-0.800617	6.169958	2.026750
157	1	0	-0.974153	7.226522	2.238794
158	1	0	-0.519199	5.653233	2.948634
159	1	0	0.019092	6.080347	1.308296
160	6	0	-3.436441	3.896311	3.314300
161	6	0	-2.775060	4.953448	4.218172
162	1	0	-3.367699	5.070443	5.131608
163	1	0	-1.768062	4.647361	4.512374
164	1	0	-2.711828	5.929628	3.738772
165	6	0	-4.724961	4.484574	2.704684
166	1	0	-5.400678	4.820342	3.499632
167	1	0	-4.503495	5.342128	2.062635
168	1	0	-5.253017	3.733714	2.106370
169	6	0	-3.830667	2.723500	4.228763
170	1	0	-4.400101	1.950045	3.704846
171	1	0	-2.952125	2.255978	4.687257
172	1	0	-4.468709	3.099686	5.034202
173	6	0	-0.892111	4.772521	-1.169151
174	6	0	0.647576	4.790239	-1.193495
175	1	0	0.996626	5.392689	-2.039930
176	1	0	1.070418	5.217643	-0.279416
177	1	0	1.053711	3.779961	-1.308891
178	6	0	-1.420216	6.219064	-1.158385
179	1	0	-0.978827	6.838662	-0.378563
180	1	0	-1.166033	6.682674	-2.117600
181	1	0	-2.508110	6.251715	-1.050778
182	6	0	-1.379646	4.168538	-2.505827
183	1	0	-0.972405	3.174022	-2.703714
184	1	0	-2.471690	4.100479	-2.535504
185	1	0	-1.059957	4.814733	-3.329807
186	8	0	-1.129195	-1.625013	-2.721851
187	6	0	-0.135929	-1.426871	-3.509614
188	1	0	1.027724	-0.688458	-2.554464
189	6	0	0.719036	-2.567947	-3.864376
190	6	0	0.582094	-3.905880	-3.657004
191	8	0	1.831914	-2.307906	-4.600640
192	6	0	1.694416	-4.516736	-4.314791
193	1	0	-0.218475	-4.392630	-3.118863
194	6	0	2.416918	-3.503770	-4.859889
195	1	0	1.923343	-5.570920	-4.368816
196	1	0	3.327632	-3.467621	-5.438208
197	6	0	-0.304104	-0.336615	-4.580409
198	1	0	-0.472173	-0.829288	-5.541897
199	1	0	0.565070	0.312175	-4.654287
200	8	0	-1.409931	0.473131	-4.226009
201	6	0	-2.629389	-0.072372	-4.450535
202	8	0	-2.801597	-1.018581	-5.190764
203	6	0	-3.710658	0.630456	-3.721565
204	6	0	-5.025805	0.508450	-4.179160
205	6	0	-3.434272	1.370657	-2.572260
206	6	0	-6.050890	1.160526	-3.504502
207	1	0	-5.233400	-0.081972	-5.065800
208	6	0	-4.462522	2.012271	-1.891749
209	1	0	-2.417260	1.430106	-2.209349
210	6	0	-5.768678	1.917845	-2.366339
211	1	0	-7.071458	1.080798	-3.865075
212	1	0	-4.240793	2.582480	-0.993969
213	1	0	-6.573133	2.427919	-1.844989

4a(*R*) -1b

RwB97XD SCF energy	-5094.196376 a.u.
RwB97XD SCF enthalpy	-5092.256638 a.u.
RwB97XD SCF free energy	-5092.498130 a.u.
Three lowest frequencies (cm^{-1})	9.8, 14.8, 19.4

Cartesian coordinates:

Center Number	Atomic Number	Atomic Type	Coordinates (Angstroms) X	Y	Z
1	6	0	-0.829747	-0.455981	-2.755539
2	6	0	-0.563885	-0.119226	-4.063670
3	6	0	-0.967179	1.086607	-4.630246
4	6	0	-1.648626	2.035626	-3.906902
5	6	0	-1.954404	1.708196	-2.576793
6	6	0	-1.571267	0.494921	-2.007568
7	1	0	-1.957914	2.977211	-4.344623
8	1	0	-2.518889	2.424916	-1.990619
9	6	0	-0.402972	-1.778980	-2.238350
10	6	0	0.473531	-2.007866	-1.140866
11	6	0	-0.907290	-2.904104	-2.851083
12	6	0	0.754467	-3.310096	-0.725223
13	6	0	-0.620973	-4.197601	-2.426683
14	6	0	0.206941	-4.435947	-1.356865
15	1	0	1.423917	-3.477273	0.110470
16	1	0	0.432954	-5.440214	-1.019123
17	8	0	-0.562379	1.123729	-5.930688
18	8	0	0.117744	-0.848006	-4.992144
19	8	0	-1.776340	-2.953187	-3.897269
20	8	0	-1.294776	-5.085648	-3.203810
21	6	0	-0.211350	-0.229104	-6.235311
22	1	0	0.651310	-0.247942	-6.898331
23	1	0	-1.078891	-0.736258	-6.674661
24	6	0	-1.794739	-4.321249	-4.305867
25	1	0	-1.129920	-4.452806	-5.167132
26	1	0	-2.818199	-4.621490	-4.527729
27	15	0	1.200157	-0.622099	-0.202217
28	15	0	-1.938470	0.152248	-0.258969
29	46	0	-0.216468	0.398666	1.213177
30	6	0	1.781941	0.604013	-1.398964
31	6	0	2.466457	0.241443	-2.560092
32	6	0	1.544775	1.943148	-1.132226
33	6	0	2.873429	1.200759	-3.483012
34	1	0	2.659459	-0.807002	-2.747956
35	6	0	1.928084	2.958841	-2.010327
36	1	0	1.024470	2.204318	-0.216555
37	6	0	2.511911	2.549861	-3.224590
38	6	0	2.609112	-1.339628	0.678913
39	6	0	3.847561	-1.466658	0.049408
40	6	0	2.436308	-1.833595	1.964680
41	6	0	4.882528	-2.187994	0.635446
42	1	0	3.995294	-1.010962	-0.919155
43	6	0	3.463061	-2.502034	2.639703
44	1	0	1.486705	-1.675942	2.461854
45	6	0	4.632318	-2.771907	1.902030
46	6	0	-3.144859	1.395239	0.267419
47	6	0	-4.509087	1.124299	0.261522
48	6	0	-2.683769	2.624558	0.739621
49	6	0	-5.444946	2.096822	0.609351
50	1	0	-4.842741	0.134118	-0.016299
51	6	0	-3.567897	3.640441	1.104961
52	1	0	-1.613106	2.784569	0.817754
53	6	0	-4.943949	3.386371	0.902808
54	6	0	-2.744263	-1.459075	-0.127890
55	6	0	-2.433723	-2.245107	0.972344
56	6	0	-3.680251	-1.909252	-1.059880
57	6	0	-3.097291	-3.448203	1.233803
58	1	0	-1.648131	-1.912324	1.644967
59	6	0	-4.407723	-3.074019	-0.834284
60	1	0	-3.844071	-1.327011	-1.958868
61	6	0	-4.169440	-3.767608	0.380251
62	6	0	-2.553896	-4.303251	2.403002
63	6	0	-2.805387	-3.581510	3.739350
64	1	0	-2.409957	-4.179367	4.567972
65	1	0	-2.310968	-2.604683	3.768486
66	1	0	-3.874205	-3.424020	3.916977
67	6	0	-3.110287	-5.735127	2.490289
68	1	0	-3.027309	-6.259576	1.534684
69	1	0	-2.516111	-6.286956	3.226765
70	1	0	-4.148334	-5.778599	2.820427
71	6	0	-1.029223	-4.461614	2.204424
72	1	0	-0.812065	-4.991731	1.271796
73	1	0	-0.500434	-3.504925	2.179112
74	1	0	-0.611055	-5.044574	3.031736
75	6	0	-5.369196	-3.617510	-1.909917
76	6	0	-4.937157	-5.051309	-2.277709
77	1	0	-5.085413	-5.741581	-1.445037
78	1	0	-5.522104	-5.413560	-3.130309
79	1	0	-3.877900	-5.079236	-2.551831
80	6	0	-6.836303	-3.620109	-1.441067
81	1	0	-7.130060	-2.640794	-1.046924
82	1	0	-7.490836	-3.849246	-2.289455
83	1	0	-7.020858	-4.373827	-0.675235
84	6	0	-5.304082	-2.768543	-3.190370
85	1	0	-5.668046	-1.748120	-3.026842
86	1	0	-4.290701	-2.713696	-3.600580
87	1	0	-5.946279	-3.223794	-3.950574
88	8	0	-5.018462	-4.797128	0.700173
89	8	0	-5.832684	4.426411	1.041375
90	8	0	2.763452	3.476666	-4.203656
91	8	0	5.587792	-3.613351	2.414701
92	6	0	5.241235	-4.991397	2.294746
93	1	0	6.076703	-5.560245	2.706338
94	1	0	4.330176	-5.231370	2.852019
95	1	0	5.090376	-5.269006	1.246234
96	6	0	3.218547	-2.815962	4.135241
97	6	0	4.457865	-3.267553	4.927686
98	1	0	4.195036	-3.283525	5.991130
99	1	0	4.800916	-4.269303	4.668164
100	1	0	5.291516	-2.571415	4.801756
101	6	0	2.110015	-3.873826	4.274718
102	1	0	1.927008	-4.088016	5.333682
103	1	0	1.170260	-3.520232	3.839874
104	1	0	2.379964	-4.812876	3.780304
105	6	0	-5.984588	-4.440118	1.686657
106	1	0	-6.611525	-5.320219	1.839622
107	1	0	-5.512253	-4.161107	2.633815
108	1	0	-6.605286	-3.603326	1.348201
109	6	0	-6.067712	5.110347	-0.194427
110	1	0	-6.754652	5.928014	0.029575
111	1	0	-5.140327	5.512412	-0.610137
112	1	0	-6.517345	4.446786	-0.937207
113	6	0	-6.923680	1.695181	0.814351
114	6	0	-7.137966	0.198369	0.522779
115	1	0	-6.941927	-0.045141	-0.527609
116	1	0	-6.513761	-0.445188	1.151528
117	1	0	-8.181873	-0.055709	0.730429
118	6	0	-7.266220	1.939200	2.298040
119	1	0	-7.147699	2.992443	2.564858
120	1	0	-8.305991	1.654045	2.493664
121	1	0	-6.621477	1.341235	2.951321
122	6	0	-7.920850	2.468973	-0.065913
123	1	0	-8.018837	3.510480	0.241698
124	1	0	-7.638741	2.432843	-1.123395
125	1	0	-8.910726	2.009668	0.028676
126	6	0	-3.042496	4.899983	1.830957
127	6	0	-1.533795	4.781167	2.102210
128	1	0	-1.292872	3.870603	2.655693
129	1	0	-0.945092	4.798146	1.178887
130	1	0	-1.211977	5.631276	2.711373
131	6	0	-3.261438	6.208322	1.051488
132	1	0	-2.698516	7.013852	1.535483
133	1	0	-2.902552	6.126131	0.019827
134	1	0	-4.309227	6.510765	1.039157
135	6	0	-3.747828	4.995757	3.199191
136	1	0	-3.354271	5.851788	3.758589
137	1	0	-4.825628	5.130587	3.085564
138	1	0	-3.568642	4.093527	3.793627
139	6	0	6.258814	-2.273338	-0.057149
140	6	0	2.744100	-1.510287	4.814018
141	1	0	3.498369	-0.723434	4.708293
142	1	0	1.805426	-1.132469	4.402381
143	1	0	2.585845	-1.690583	5.882751
144	6	0	7.311853	-1.618235	0.856296
145	1	0	8.295973	-1.648904	0.374730
146	1	0	7.061650	-0.569563	1.045968
147	1	0	7.387558	-2.134664	1.816062
148	6	0	6.671881	-3.723808	-0.373076
149	1	0	5.867013	-4.264913	-0.882548
150	1	0	7.540828	-3.716938	-1.040426
151	1	0	6.954220	-4.275496	0.523505
152	6	0	6.257210	-1.509610	-1.391823
153	1	0	6.005285	-0.453155	-1.262701
154	1	0	7.258782	-1.555629	-1.830660
155	1	0	5.561810	-1.952734	-2.113003
156	6	0	1.609294	3.795724	-4.982116
157	1	0	1.926759	4.532877	-5.721490
158	1	0	1.218323	2.910998	-5.492534
159	1	0	0.813428	4.222463	-4.364460
160	6	0	3.782661	0.796959	-4.663862

161	6	0	3.267038	1.266462	-6.036542
162	1	0	3.818775	0.747795	-6.827869
163	1	0	2.207242	1.033221	-6.162593
164	1	0	3.406595	2.336890	-6.185554
165	6	0	5.175312	1.407919	-4.409840
166	1	0	5.847915	1.165774	-5.240571
167	1	0	5.123878	2.496955	-4.320894
168	1	0	5.616967	1.004282	-3.491653
169	6	0	3.946355	-0.729367	-4.746722
170	1	0	4.393474	-1.150555	-3.841113
171	1	0	2.990085	-1.231532	-4.930695
172	1	0	4.616410	-0.970362	-5.577505
173	6	0	1.654450	4.406354	-1.534435
174	6	0	0.134089	4.655292	-1.544092
175	1	0	-0.083675	5.661247	-1.168074
176	1	0	-0.282832	4.573688	-2.553064
177	1	0	-0.390059	3.934607	-0.906348
178	6	0	2.359101	5.514114	-2.336043
179	1	0	1.942483	5.660479	-3.332360
180	1	0	2.234037	6.458842	-1.795947
181	1	0	3.431573	5.324415	-2.431384
182	6	0	2.169798	4.555001	-0.084010
183	1	0	1.697385	3.863512	0.618647
184	1	0	3.251669	4.395651	-0.034715
185	1	0	1.961522	5.569402	0.271744
186	8	0	1.183652	0.742719	2.629808
187	6	0	1.026640	1.994361	3.215982
188	6	0	-0.161629	2.018982	4.141351
189	6	0	-1.177815	1.137240	4.353541
190	8	0	-0.374895	3.151593	4.864627
191	6	0	-2.083211	1.776241	5.266639
192	1	0	-1.270920	0.152360	3.915265
193	6	0	-1.546294	2.989889	5.540711
194	1	0	-3.002832	1.374695	5.667669
195	1	0	-1.845163	3.813150	6.171617
196	6	0	2.317183	2.356087	3.946752
197	1	0	2.604556	1.563881	4.641147
198	1	0	2.234280	3.306201	4.480782
199	8	0	3.302695	2.500306	2.917675
200	6	0	4.525954	2.004459	3.099773
201	8	0	4.947774	1.596403	4.166110
202	6	0	5.311308	2.043614	1.837226
203	6	0	6.704466	2.109800	1.898534
204	6	0	4.663330	2.025771	0.599593
205	6	0	7.443642	2.187572	0.721812
206	1	0	7.200031	2.116302	2.864234
207	6	0	5.404971	2.094480	-0.573693
208	1	0	3.583145	1.954831	0.557104
209	6	0	6.795278	2.183329	-0.512715
210	1	0	8.526080	2.252968	0.768275
211	1	0	4.894690	2.081231	-1.531916
212	1	0	7.373649	2.243366	-1.429891
213	1	0	0.866268	2.786050	2.457127

5a(*R*) -1b

RwB97XD SCF energy -5095.379436 a.u.
RwB97XD SCF enthalpy -5093.422731 a.u.
RwB97XD SCF free energy -5093.666336 a.u.
Three lowest frequencies (cm^{-1}) 13.6, 15.9, 18.9
Cartesian coordinates:

Center Number	Atomic Number	Atomic Type	Coordinates (Angstroms) X	Y	Z
1	6	0	-0.956418	-0.337917	2.657804
2	6	0	-0.709739	-1.028763	3.822544
3	6	0	-1.087498	-2.354146	4.012810
4	6	0	-1.718489	-3.072062	3.026138
5	6	0	-1.989973	-2.389045	1.830909
6	6	0	-1.633404	-1.054742	1.637378
7	1	0	-2.008837	-4.106480	3.166054
8	1	0	-2.506150	-2.928605	1.045678
9	6	0	-0.577452	1.092575	2.575449
10	6	0	0.331022	1.665307	1.645215
11	6	0	-1.150164	1.962558	3.477742
12	6	0	0.565970	3.041663	1.656885
13	6	0	-0.905749	3.330905	3.479606
14	6	0	-0.055758	3.907429	2.567599
15	1	0	1.265719	3.476171	0.954297
16	1	0	0.140790	4.972851	2.557777
17	8	0	-0.716686	-2.748430	5.262992
18	8	0	-0.071120	-0.577589	4.939731
19	8	0	-2.049982	1.663292	4.455433
20	8	0	-1.636337	3.917742	4.465731
21	6	0	-0.409940	-1.529189	5.946870
22	1	0	0.437934	-1.680307	6.611970
23	1	0	-1.301032	-1.184606	6.485615
24	6	0	-2.113938	2.834492	5.269502
25	1	0	-1.455583	2.709743	6.137044
26	1	0	-3.146728	3.021549	5.560373
27	15	0	1.144573	0.636770	0.365806
28	15	0	-1.995022	-0.243707	0.042568
29	46	0	-0.219542	0.158799	-1.396432
30	6	0	1.689860	-0.904743	1.137378
31	6	0	2.378761	-0.912266	2.350014
32	6	0	1.430652	-2.101495	0.485451
33	6	0	2.763887	-2.107413	2.949559
34	1	0	2.598314	0.033652	2.829041
35	6	0	1.791817	-3.339220	1.024391
36	1	0	0.919219	-2.070802	-0.470408
37	6	0	2.378890	-3.313745	2.306052
38	6	0	2.594479	1.593931	-0.145318
39	6	0	3.875944	1.312204	0.322831
40	6	0	2.400334	2.658754	-1.015954
41	6	0	4.946716	2.160228	0.039661
42	1	0	4.035491	0.428997	0.926056
43	6	0	3.439892	3.514842	-1.382844
44	1	0	1.410181	2.819901	-1.426139
45	6	0	4.679905	3.308155	-0.744337
46	6	0	-3.131803	-1.381612	-0.795070
47	6	0	-4.508942	-1.270136	-0.649655
48	6	0	-2.599995	-2.371508	-1.618444
49	6	0	-5.379000	-2.193317	-1.231560
50	1	0	-4.907204	-0.441687	-0.080119
51	6	0	-3.411021	-3.330065	-2.223699
52	1	0	-1.529111	-2.383130	-1.790238
53	6	0	-4.789245	-3.285555	-1.907689
54	6	0	-2.912050	1.276954	0.379418
55	6	0	-2.679681	2.359304	-0.455784
56	6	0	-3.850948	1.385504	1.406341
57	6	0	-3.418187	3.542170	-0.363229
58	1	0	-1.898320	2.280108	-1.204773
59	6	0	-4.652647	2.517375	1.523787
60	1	0	-3.953309	0.566455	2.107846
61	6	0	-4.483993	3.541547	0.555463
62	6	0	-2.957930	4.715862	-1.258539
63	6	0	-3.267245	4.398848	-2.733041
64	1	0	-2.908656	5.210330	-3.376094
65	1	0	-2.772632	3.475058	-3.053191
66	1	0	-4.341550	4.281884	-2.906139
67	6	0	-3.551386	6.086815	-0.891225
68	1	0	-3.409488	6.312681	0.169602
69	1	0	-3.026765	6.855125	-1.469516
70	1	0	-4.612544	6.178253	-1.122802
71	6	0	-1.425292	4.864144	-1.104022
72	1	0	-1.151214	5.044271	-0.059007
73	1	0	-0.875833	3.985860	-1.454892
74	1	0	-1.082122	5.717167	-1.698346
75	6	0	-5.620507	2.670499	2.715031
76	6	0	-5.250732	3.954717	3.484375
77	1	0	-5.437158	4.849814	2.887595
78	1	0	-5.843342	4.027464	4.403284
79	1	0	-4.191023	3.948909	3.759082
80	6	0	-7.094907	2.732000	2.274570
81	1	0	-7.350525	1.891738	1.619320
82	1	0	-7.741288	2.673963	3.157432
83	1	0	-7.330882	3.662534	1.758184
84	6	0	-5.491251	1.489969	3.692263
85	1	0	-5.802632	0.543297	3.237079
86	1	0	-4.470772	1.373878	4.069781
87	1	0	-6.145750	1.669063	4.550856
88	8	0	-5.399428	4.565164	0.548726
89	8	0	-5.593486	-4.324376	-2.316773
90	8	0	2.634327	-4.489824	2.964225
91	8	0	5.692086	4.224185	-0.893898
92	6	0	5.537989	5.381985	-0.077640
93	1	0	6.444619	5.975707	-0.207223
94	1	0	4.671542	5.979660	-0.376535
95	1	0	5.424405	5.112145	0.977540
96	6	0	3.120690	4.568813	-2.470275

97	6	0	4.349138	5.217642	-3.131929
98	1	0	4.005877	5.805711	-3.989998
99	1	0	4.893728	5.894980	-2.474566
100	1	0	5.049916	4.463941	-3.502906
101	6	0	2.212337	5.661941	-1.878814
102	1	0	1.916846	6.372692	-2.658823
103	1	0	1.300704	5.228804	-1.455121
104	1	0	2.714760	6.223406	-1.084828
105	6	0	-6.372800	4.429037	-0.485310
106	1	0	-7.092590	5.237922	-0.348151
107	1	0	-5.921785	4.514520	-1.478723
108	1	0	-6.887353	3.464606	-0.416611
109	6	0	-5.700265	-5.352081	-1.326144
110	1	0	-6.358721	-6.117095	-1.741281
111	1	0	-4.725759	-5.792108	-1.101130
112	1	0	-6.128706	-4.967893	-0.397174
113	6	0	-6.897847	-1.903705	-1.251773
114	6	0	-7.220780	-0.581260	-0.531719
115	1	0	-6.983799	-0.628090	0.537227
116	1	0	-6.694535	0.274108	-0.967872
117	1	0	-8.293355	-0.383820	-0.621285
118	6	0	-7.323385	-1.751082	-2.726013
119	1	0	-7.137830	-2.668526	-3.290461
120	1	0	-8.394738	-1.528510	-2.785176
121	1	0	-6.779841	-0.931332	-3.208349
122	6	0	-7.760253	-2.992652	-0.589425
123	1	0	-7.800784	-3.906207	-1.183764
124	1	0	-7.398322	-3.236192	0.415014
125	1	0	-8.787128	-2.624481	-0.490059
126	6	0	-2.822345	-4.268057	-3.301961
127	6	0	-1.330602	-3.976060	-3.540344
128	1	0	-1.148362	-2.935822	-3.828347
129	1	0	-0.719638	-4.210368	-2.662235
130	1	0	-0.974806	-4.606329	-4.361336
131	6	0	-2.934345	-5.763184	-2.955953
132	1	0	-2.324582	-6.343183	-3.656974
133	1	0	-2.561666	-5.967762	-1.946346
134	1	0	-3.958380	-6.129363	-3.038468
135	6	0	-3.563197	-3.988941	-4.625544
136	1	0	-3.159443	-4.625624	-5.420659
137	1	0	-4.633005	-4.192841	-4.538065
138	1	0	-3.433790	-2.944297	-4.929626
139	6	0	6.369914	1.803688	0.520705
140	6	0	2.354036	3.862821	-3.613886
141	1	0	2.940380	3.033257	-4.022852
142	1	0	1.383614	3.471536	-3.298654
143	1	0	2.165614	4.578118	-4.421391
144	6	0	7.291399	1.673286	-0.708140
145	1	0	8.281234	1.317146	-0.400216
146	1	0	6.883996	0.957946	-1.428900
147	1	0	7.415913	2.632504	-1.214934
148	6	0	6.945512	2.852497	1.491240
149	1	0	6.245585	3.063422	2.307175
150	1	0	7.868260	2.464761	1.937239
151	1	0	7.194150	3.788188	0.990840
152	6	0	6.382786	0.459059	1.266203
153	1	0	5.994090	-0.355705	0.652929
154	1	0	7.414287	0.210026	1.533226
155	1	0	5.804125	0.501373	2.196166
156	6	0	1.492148	-5.036157	3.623498
157	1	0	1.829825	-5.944014	4.126353
158	1	0	1.088530	-4.338056	4.362379
159	1	0	0.699789	-5.291875	2.914568
160	6	0	3.664513	-2.082600	4.204019
161	6	0	3.103636	-2.900937	5.381451
162	1	0	3.655158	-2.649907	6.293824
163	1	0	2.049555	-2.670347	5.554268
164	1	0	3.201571	-3.974577	5.222581
165	6	0	5.047278	-2.639148	3.811403
166	1	0	5.709457	-2.645311	4.684879
167	1	0	4.973071	-3.663760	3.436105
168	1	0	5.513290	-2.019537	3.037613
169	6	0	3.864927	-0.646486	4.716335
170	1	0	4.335670	0.003040	3.971261
171	1	0	2.918983	-0.189956	5.028761
172	1	0	4.527161	-0.668987	5.587173
173	6	0	1.504092	-4.588241	0.151736
174	6	0	-0.012380	-4.859832	0.137541
175	1	0	-0.226036	-5.748707	-0.466820
176	1	0	-0.407345	-5.032276	1.143084
177	1	0	-0.561113	-4.017021	-0.296552
178	6	0	2.255513	-5.864576	0.571589
179	1	0	1.870286	-6.319569	1.483864
180	1	0	2.145466	-6.605625	-0.227696
181	1	0	3.324476	-5.675816	0.704153
182	6	0	1.953443	-4.317976	-1.302855
183	1	0	1.495085	-3.431649	-1.749203
184	1	0	3.039442	-4.203430	-1.363911
185	1	0	1.673707	-5.173660	-1.926316
186	8	0	1.401250	0.427136	-2.583637
187	6	0	1.527311	-0.445419	-3.665058
188	6	0	0.667332	-0.042786	-4.830965
189	6	0	0.481249	1.131635	-5.491491
190	8	0	-0.192843	-0.982740	-5.314836
191	6	0	-0.564420	0.900349	-6.445164
192	1	0	1.019238	2.052925	-5.317329
193	6	0	-0.934102	-0.395790	-6.292024
194	1	0	-0.975850	1.606767	-7.152242
195	1	0	-1.661010	-1.028250	-6.778976
196	6	0	2.999327	-0.464088	-4.063275
197	1	0	3.365594	0.555877	-4.207607
198	1	0	3.168759	-1.047464	-4.973750
199	8	0	3.697241	-1.090190	-2.976991
200	6	0	5.016876	-0.924775	-2.897496
201	8	0	5.660693	-0.257633	-3.687431
202	6	0	5.607966	-1.677783	-1.759000
203	6	0	6.995609	-1.827625	-1.699862
204	6	0	4.801896	-2.244543	-0.769181
205	6	0	7.572155	-2.548947	-0.659882
206	1	0	7.613701	-1.381704	-2.472229
207	6	0	5.380302	-2.978378	0.259861
208	1	0	3.728939	-2.103358	-0.800445
209	6	0	6.764503	-3.129416	0.317317
210	1	0	8.650386	-2.664449	-0.614574
211	1	0	4.748350	-3.423753	1.019959
212	1	0	7.213803	-3.695569	1.127770
213	1	0	1.247948	-1.482417	-3.409125
214	1	0	-1.375485	0.396851	-2.896823
215	1	0	-1.332468	-0.377284	-2.887933

TS2a(*R*) -1b

RwB97XD SCF energy -5095.359187 a.u.
RwB97XD SCF enthalpy -5093.404003 a.u.
RwB97XD SCF free energy -5093.646900 a.u.
Three lowest frequencies (cm^{-1}) -1095, 5.8, 12.0
Imaginary frequency (cm^{-1}) -1095
Cartesian coordinates:

Center Number	Atomic Number	Atomic Type	Coordinates (Angstroms) X	Y	Z
1	6	0	1.022497	-1.415205	2.452721
2	6	0	0.880406	-1.568339	3.830846
3	6	0	1.350939	-0.639597	4.769196
4	6	0	2.000264	0.520127	4.381653
5	6	0	2.181173	0.698276	2.995724
6	6	0	1.725063	-0.230995	2.039480
7	1	0	2.367246	1.246855	5.100886
8	1	0	2.719364	1.581635	2.665146
9	6	0	0.524802	-2.500491	1.561105
10	6	0	-0.439167	-2.362192	0.505283
11	6	0	0.981780	-3.799674	1.784011
12	6	0	-0.819229	-3.483018	-0.258938
13	6	0	0.583337	-4.904555	1.021090
14	6	0	-0.311802	-4.777431	-0.026728
15	1	0	-1.548279	-3.361878	-1.054469
16	1	0	-0.622024	-5.626352	-0.629548
17	8	0	1.034493	-1.058921	6.039796
18	8	0	0.235459	-2.594908	4.487849
19	8	0	1.910685	-4.201251	2.720756
20	8	0	1.235507	-6.034812	1.457749
21	6	0	0.615084	-2.439593	5.876713
22	1	0	-0.248779	-2.635385	6.512559
23	1	0	1.465021	-3.099616	6.095905
24	6	0	1.843428	-5.648710	2.717046
25	1	0	1.201914	-5.982211	3.543793
26	1	0	2.852699	-6.058289	2.773343
27	15	0	-1.188477	-0.728220	0.090161
28	15	0	2.061021	0.106969	0.265216
29	46	0	0.272105	0.705587	-1.022058

30	6	0	-1.857310	-0.030212	1.647943
31	6	0	-2.446816	-0.830052	2.642238
32	6	0	-1.831430	1.362740	1.808471
33	6	0	-2.991606	-0.263069	3.806975
34	1	0	-2.475557	-1.906510	2.502674
35	6	0	-2.355190	2.001770	2.951196
36	1	0	-1.391199	1.958436	1.011866
37	6	0	-2.883308	1.152812	3.968591
38	6	0	-2.613835	-1.180779	-0.985886
39	6	0	-3.827410	-1.630473	-0.429150
40	6	0	-2.454455	-1.192803	-2.380544
41	6	0	-4.818250	-2.230202	-1.222506
42	1	0	-3.983091	-1.540449	0.641097
43	6	0	-3.434222	-1.740639	-3.239943
44	1	0	-1.544516	-0.772921	-2.805394
45	6	0	-4.539868	-2.374345	-2.614327
46	6	0	3.256698	1.507859	0.239205
47	6	0	4.611708	1.284085	-0.051184
48	6	0	2.802490	2.826120	0.435617
49	6	0	5.553654	2.330457	-0.033389
50	1	0	4.937124	0.280617	-0.302380
51	6	0	3.696063	3.911345	0.496925
52	1	0	1.734810	2.999794	0.542663
53	6	0	5.084224	3.607245	0.380662
54	6	0	2.969241	-1.324315	-0.418554
55	6	0	2.810600	-1.638191	-1.774505
56	6	0	3.882249	-2.057776	0.364204
57	6	0	3.592659	-2.625816	-2.408711
58	1	0	2.056303	-1.097722	-2.345453
59	6	0	4.719088	-3.026389	-0.213819
60	1	0	3.952394	-1.848605	1.427541
61	6	0	4.618086	-3.220941	-1.625581
62	6	0	3.221913	-2.979419	-3.877538
63	6	0	3.612556	-1.807056	-4.814987
64	1	0	3.309638	-2.036024	-5.846502
65	1	0	3.113863	-0.875584	-4.513787
66	1	0	4.695739	-1.627624	-4.811551
67	6	0	3.827175	-4.296433	-4.423764
68	1	0	3.634629	-5.141067	-3.750955
69	1	0	3.341229	-4.515718	-5.385085
70	1	0	4.903028	-4.239405	-4.610561
71	6	0	1.679130	-3.169808	-3.955366
72	1	0	1.346442	-3.975261	-3.286630
73	1	0	1.128283	-2.258803	-3.696454
74	1	0	1.401989	-3.441872	-4.982718
75	6	0	5.642418	-3.896030	0.680092
76	6	0	5.218998	-5.382380	0.523768
77	1	0	5.365312	-5.731563	-0.504493
78	1	0	5.824808	-6.012898	1.190233
79	1	0	4.162231	-5.520603	0.787848
80	6	0	7.146550	-3.756894	0.327865
81	1	0	7.464102	-2.705572	0.332529
82	1	0	7.740445	-4.287286	1.085827
83	1	0	7.384431	-4.194161	-0.645219
84	6	0	5.499412	-3.518596	2.173728
85	1	0	5.829345	-2.488932	2.370860
86	1	0	4.469675	-3.629197	2.535887
87	1	0	6.135638	-4.188393	2.767337
88	8	0	5.545978	-4.070062	-2.217545
89	8	0	6.014447	4.619271	0.613298
90	8	0	-3.407535	1.673557	5.145916
91	8	0	-5.424958	-3.141790	-3.364248
92	6	0	-4.903724	-4.456770	-3.686193
93	1	0	-5.696046	-4.963257	-4.245509
94	1	0	-3.999033	-4.391732	-4.302670
95	1	0	-4.674869	-5.018869	-2.771456
96	6	0	-3.226776	-1.560764	-4.772156
97	6	0	-4.447041	-1.901579	-5.663432
98	1	0	-4.220721	-1.557728	-6.682928
99	1	0	-4.660702	-2.972548	-5.723184
100	1	0	-5.353456	-1.382692	-5.329155
101	6	0	-2.006810	-2.390390	-5.247519
102	1	0	-1.839185	-2.225371	-6.321364
103	1	0	-1.094217	-2.095542	-4.714164
104	1	0	-2.160660	-3.466229	-5.090190
105	6	0	6.589786	-3.368093	-2.941401
106	1	0	7.260329	-4.143461	-3.323777
107	1	0	6.179168	-2.789241	-3.777227
108	1	0	7.140751	-2.694277	-2.272874
109	6	0	6.471308	4.643017	1.996278
110	1	0	7.215659	5.443175	2.049283
111	1	0	5.642882	4.857592	2.680294
112	1	0	6.927593	3.687512	2.276228
113	6	0	6.982528	2.093606	-0.598023
114	6	0	7.139001	0.648791	-1.130150
115	1	0	7.064511	-0.096108	-0.326252
116	1	0	6.395310	0.406363	-1.901042
117	1	0	8.133593	0.548680	-1.584244
118	6	0	7.188519	3.054717	-1.801156
119	1	0	7.122538	4.103243	-1.489777
120	1	0	8.182495	2.888559	-2.240327
121	1	0	6.437367	2.873778	-2.582192
122	6	0	8.124467	2.321540	0.424996
123	1	0	8.256511	3.380603	0.664946
124	1	0	7.953323	1.762689	1.354805
125	1	0	9.068123	1.963669	-0.010297
126	6	0	3.157486	5.368647	0.524257
127	6	0	1.611690	5.395827	0.534462
128	1	0	1.180733	4.880782	-0.332592
129	1	0	1.200604	4.951158	1.450395
130	1	0	1.278894	6.441390	0.495512
131	6	0	3.624992	6.200140	1.746165
132	1	0	3.061715	7.143644	1.768731
133	1	0	3.432444	5.674899	2.691406
134	1	0	4.687189	6.453779	1.688432
135	6	0	3.629601	6.079001	-0.774424
136	1	0	3.243211	7.108112	-0.793321
137	1	0	4.723224	6.123522	-0.829709
138	1	0	3.255210	5.559208	-1.666787
139	6	0	-6.168743	-2.671956	-0.600386
140	6	0	-2.929498	-0.057062	-5.037722
141	1	0	-3.773456	0.569811	-4.719300
142	1	0	-2.028770	0.292566	-4.522551
143	1	0	-2.779515	0.095479	-6.115693
144	6	0	-7.333394	-1.956650	-1.337005
145	1	0	-8.291201	-2.255878	-0.887774
146	1	0	-7.243332	-0.866163	-1.246729
147	1	0	-7.357464	-2.216923	-2.400545
148	6	0	-6.380945	-4.207083	-0.672415
149	1	0	-5.523443	-4.748449	-0.249946
150	1	0	-7.269557	-4.475156	-0.083228
151	1	0	-6.545548	-4.551513	-1.697061
152	6	0	-6.255386	-2.275899	0.891792
153	1	0	-6.125862	-1.196011	1.039090
154	1	0	-7.249007	-2.548397	1.271549
155	1	0	-5.511289	-2.805902	1.501500
156	6	0	-2.473151	2.113255	6.165442
157	1	0	-2.933526	1.879774	7.131258
158	1	0	-1.514724	1.589746	6.077503
159	1	0	-2.317009	3.196536	6.105891
160	6	0	-3.730328	-1.165116	4.831383
161	6	0	-3.127438	-1.064453	6.258925
162	1	0	-3.492809	-1.905246	6.865246
163	1	0	-2.031620	-1.114828	6.236761
164	1	0	-3.422651	-0.142361	6.764938
165	6	0	-5.228527	-0.759819	4.878562
166	1	0	-5.758619	-1.386441	5.610202
167	1	0	-5.349325	0.288738	5.172364
168	1	0	-5.705672	-0.906264	3.899845
169	6	0	-3.666289	-2.657090	4.424274
170	1	0	-4.124133	-2.844302	3.444235
171	1	0	-2.635083	-3.034908	4.405145
172	1	0	-4.223926	-3.245577	5.164717
173	6	0	-2.315106	3.556310	3.001135
174	6	0	-1.015956	4.035789	3.705081
175	1	0	-0.966471	5.133762	3.681855
176	1	0	-0.952407	3.718955	4.750518
177	1	0	-0.132939	3.643091	3.183237
178	6	0	-3.568418	4.173059	3.675083
179	1	0	-3.667694	3.920318	4.732704
180	1	0	-3.510842	5.267044	3.590582
181	1	0	-4.483415	3.846129	3.162212
182	6	0	-2.278190	4.153318	1.566865
183	1	0	-1.336577	3.942006	1.045655
184	1	0	-3.109507	3.791091	0.948507
185	1	0	-2.363111	5.245526	1.642226
186	8	0	-0.905764	1.719539	-2.639587
187	6	0	-1.227233	3.115713	-2.555513
188	6	0	-0.249058	3.925811	-3.370212
189	6	0	0.439924	3.687053	-4.534351
190	8	0	0.020235	5.206457	-2.924469
191	6	0	1.185459	4.880308	-4.824835
192	1	0	0.414709	2.772760	-5.115403
193	6	0	0.896702	5.770231	-3.827944

194	1	0	1.846220	5.050818	-5.666774
195	1	0	1.200278	6.785144	-3.608551
196	6	0	-2.632505	3.335197	-3.128738
197	1	0	-2.743242	2.822120	-4.088962
198	1	0	-2.824216	4.406912	-3.262015
199	8	0	-3.608538	2.816820	-2.173303
200	6	0	-4.904115	2.951361	-2.569468
201	8	0	-5.213901	3.364886	-3.688651
202	6	0	-5.886072	2.587626	-1.512272
203	6	0	-7.254937	2.727854	-1.817290
204	6	0	-5.495182	2.159787	-0.227903
205	6	0	-8.222188	2.451132	-0.846363
206	1	0	-7.542423	3.060453	-2.812532
207	6	0	-6.467761	1.889847	0.742495
208	1	0	-4.441427	2.043477	0.008971
209	6	0	-7.829899	2.036319	0.436312
210	1	0	-9.278267	2.563159	-1.085528
211	1	0	-6.162739	1.568216	1.736835
212	1	0	-8.582904	1.828529	1.194873
213	1	0	-1.214385	3.486804	-1.520116
214	1	0	1.322685	1.672882	-2.036130
215	1	0	0.494139	1.773560	-2.473245

6b(*R*)•2a

RwB97XD SCF energy -5095.406432 a.u.
RwB97XD SCF enthalpy -5093.447145 a.u.
RwB97XD SCF free energy -5093.695537 a.u.
Three lowest frequencies (cm^{-1}) 9.3, 13.7, 18.1
Cartesian coordinates:

Center Number	Atomic Number	Atomic Type	Coordinates (Angstroms) X	Y	Z
1	6	0	-1.066326	0.273892	2.494410
2	6	0	-0.798623	-0.202128	3.759047
3	6	0	-1.094926	-1.496959	4.169117
4	6	0	-1.679791	-2.400516	3.317135
5	6	0	-1.979988	-1.938568	2.026897
6	6	0	-1.694869	-0.639332	1.604092
7	1	0	-1.908677	-3.415038	3.620711
8	1	0	-2.460734	-2.630801	1.345996
9	6	0	-0.705033	1.685174	2.202563
10	6	0	0.307054	2.103536	1.299375
11	6	0	-1.335764	2.683845	2.912261
12	6	0	0.572423	3.460977	1.123734
13	6	0	-1.062216	4.034138	2.731365
14	6	0	-0.116230	4.459983	1.830781
15	1	0	1.341115	3.773539	0.426000
16	1	0	0.101854	5.510951	1.681510
17	8	0	-0.697600	-1.660061	5.463106
18	8	0	-0.193678	0.466831	4.782615
19	8	0	-2.307318	2.541092	3.858003
20	8	0	-1.866130	4.768946	3.555099
21	6	0	-0.457639	-0.330213	5.935095
22	1	0	0.407618	-0.321782	6.595475
23	1	0	-1.360199	0.041713	6.435304
24	6	0	-2.398932	3.821488	4.482846
25	1	0	-1.790411	3.823436	5.395628
26	1	0	-3.443008	4.052027	4.689917
27	15	0	1.150030	0.845087	0.267302
28	15	0	-2.083628	-0.177545	-0.131673
29	46	0	-0.272247	-0.007904	-1.445676
30	6	0	1.740825	-0.460203	1.378365
31	6	0	2.383746	-0.217655	2.587432
32	6	0	1.521040	-1.768560	0.970080
33	6	0	2.793610	-1.265825	3.408624
34	1	0	2.547529	0.809784	2.886220
35	6	0	1.912227	-2.872875	1.730358
36	1	0	1.025124	-1.922048	0.019782
37	6	0	2.510707	-2.589971	2.980927
38	6	0	2.600329	1.692901	-0.425806
39	6	0	3.885496	1.578009	0.097584
40	6	0	2.397713	2.456616	-1.569106
41	6	0	4.950727	2.302462	-0.435699
42	1	0	4.050470	0.915575	0.936460
43	6	0	3.426658	3.180408	-2.177919
44	1	0	1.400996	2.491140	-1.999451
45	6	0	4.673723	3.170260	-1.520404
46	6	0	-3.236155	-1.483522	-0.658049
47	6	0	-4.592351	-1.430947	-0.356965
48	6	0	-2.732882	-2.572331	-1.362856
49	6	0	-5.448580	-2.490227	-0.661368
50	1	0	-4.992426	-0.544338	0.114053
51	6	0	-3.530258	-3.667139	-1.692942
52	1	0	-1.694266	-2.548220	-1.666494
53	6	0	-4.859338	-3.650257	-1.212736
54	6	0	-3.016712	1.372981	-0.057117
55	6	0	-2.762335	2.344446	-1.013022
56	6	0	-3.978354	1.612073	0.926586
57	6	0	-3.494682	3.534496	-1.076373
58	1	0	-1.963452	2.169072	-1.726325
59	6	0	-4.790208	2.740872	0.877891
60	1	0	-4.095814	0.893269	1.728440
61	6	0	-4.589974	3.642394	-0.199173
62	6	0	-2.993202	4.602475	-2.075530
63	6	0	-3.250652	4.131999	-3.518495
64	1	0	-2.868685	4.873106	-4.229684
65	1	0	-2.747934	3.180152	-3.722180
66	1	0	-4.318480	3.994758	-3.715075
67	6	0	-3.588470	6.007192	-1.880636
68	1	0	-3.485116	6.347983	-0.846365
69	1	0	-3.034185	6.704665	-2.517994
70	1	0	-4.639077	6.078568	-2.162129
71	6	0	-1.466924	4.764214	-1.878036
72	1	0	-1.234123	5.069483	-0.852467
73	1	0	-0.910580	3.847770	-2.093616
74	1	0	-1.093794	5.537237	-2.557889
75	6	0	-5.816003	3.011374	1.998038
76	6	0	-5.571026	4.416788	2.583523
77	1	0	-5.841957	5.199475	1.873661
78	1	0	-6.167945	4.555589	3.491451
79	1	0	-4.516149	4.552593	2.840858
80	6	0	-7.265939	2.903564	1.490611
81	1	0	-7.440335	1.940723	0.997502
82	1	0	-7.956936	2.971966	2.338505
83	1	0	-7.522691	3.703117	0.795259
84	6	0	-5.662983	1.996872	3.144324
85	1	0	-5.909291	0.976438	2.830394
86	1	0	-4.651988	1.996953	3.562406
87	1	0	-6.357565	2.263327	3.947118
88	8	0	-5.502136	4.657452	-0.353750
89	8	0	-5.619004	-4.792576	-1.329174
90	8	0	2.922110	-3.600388	3.815022
91	8	0	5.684020	4.002300	-1.940850
92	6	0	5.541215	5.343480	-1.480208
93	1	0	6.454204	5.870224	-1.764085
94	1	0	4.682880	5.842204	-1.940570
95	1	0	5.420603	5.378904	-0.392442
96	6	0	3.087551	3.885348	-3.513265
97	6	0	4.305388	4.314127	-4.351247
98	1	0	3.949700	4.627704	-5.338759
99	1	0	4.849211	5.156440	-3.925062
100	1	0	5.007598	3.487979	-4.496149
101	6	0	2.194809	5.108404	-3.235225
102	1	0	1.885707	5.573802	-4.177886
103	1	0	1.290491	4.820584	-2.689102
104	1	0	2.716153	5.865543	-2.641710
105	6	0	-6.434977	4.417883	-1.404469
106	1	0	-7.186491	5.207215	-1.342942
107	1	0	-5.956319	4.455435	-2.387800
108	1	0	-6.918578	3.441726	-1.288796
109	6	0	-5.499753	-5.642187	-0.184354
110	1	0	-6.084665	-6.538573	-0.397907
111	1	0	-4.458355	-5.918069	0.000205
112	1	0	-5.890275	-5.158013	0.714518
113	6	0	-6.977382	-2.300283	-0.532931
114	6	0	-7.320307	-0.882109	-0.039356
115	1	0	-6.956329	-0.701842	0.978261
116	1	0	-6.918044	-0.103666	-0.696125
117	1	0	-8.408396	-0.765413	-0.022786
118	6	0	-7.593783	-2.464773	-1.936592
119	1	0	-7.413909	-3.465018	-2.338180
120	1	0	-8.677239	-2.307665	-1.888328
121	1	0	-7.176925	-1.731928	-2.636129
122	6	0	-7.652443	-3.290717	0.432322
123	1	0	-7.683330	-4.301469	0.023262
124	1	0	-7.147312	-3.314713	1.403558
125	1	0	-8.688370	-2.978527	0.604011
126	6	0	-2.995651	-4.736058	-2.673259
127	6	0	-1.595838	-4.358777	-3.191321
128	1	0	-1.583276	-3.370614	-3.663351
129	1	0	-0.838922	-4.378685	-2.399340

130	1	0	-1.292448	-5.087889	-3.948777
131	6	0	-2.888090	-6.143926	-2.061007
132	1	0	-2.322103	-6.791887	-2.739196
133	1	0	-2.358641	-6.124897	-1.102234
134	1	0	-3.866242	-6.604822	-1.915008
135	6	0	-3.934728	-4.778028	-3.895763
136	1	0	-3.571145	-5.516040	-4.619658
137	1	0	-4.953116	-5.053709	-3.612528
138	1	0	-3.967138	-3.803507	-4.395085
139	6	0	6.380878	2.087904	0.104932
140	6	0	2.293806	2.900255	-4.405261
141	1	0	2.864961	1.980655	-4.579497
142	1	0	1.318304	2.635737	-3.985664
143	1	0	2.102850	3.363103	-5.378661
144	6	0	7.276419	1.599573	-1.051265
145	1	0	8.274005	1.349148	-0.673037
146	1	0	6.857034	0.703610	-1.518791
147	1	0	7.386011	2.364861	-1.822286
148	6	0	6.980960	3.365728	0.720796
149	1	0	6.299633	3.810119	1.454508
150	1	0	7.912215	3.115619	1.241331
151	1	0	7.221004	4.114648	-0.033834
152	6	0	6.402933	1.010828	1.202875
153	1	0	5.996250	0.058314	0.854946
154	1	0	7.438426	0.836211	1.510392
155	1	0	5.844229	1.322604	2.092852
156	6	0	1.920456	-4.286201	4.564010
157	1	0	2.309444	-4.444804	5.572240
158	1	0	0.997805	-3.702129	4.625842
159	1	0	1.709225	-5.263097	4.120053
160	6	0	3.584432	-0.956480	4.697795
161	6	0	3.008047	-1.680752	5.933856
162	1	0	3.266604	-1.130169	6.844343
163	1	0	1.919180	-1.760840	5.884984
164	1	0	3.415044	-2.686736	6.038426
165	6	0	5.052659	-1.380381	4.500323
166	1	0	5.631529	-1.143327	5.400724
167	1	0	5.136574	-2.455593	4.322119
168	1	0	5.507398	-0.851491	3.655275
169	6	0	3.576326	0.551601	5.006586
170	1	0	4.065534	1.142540	4.225663
171	1	0	2.559530	0.934533	5.147313
172	1	0	4.129658	0.726293	5.934213
173	6	0	1.668151	-4.280670	1.129034
174	6	0	0.319216	-4.844672	1.618125
175	1	0	0.161061	-5.843074	1.194002
176	1	0	0.252784	-4.927188	2.703263
177	1	0	-0.501971	-4.203893	1.280871
178	6	0	2.820855	-5.257298	1.426816
179	1	0	2.967133	-5.452321	2.487273
180	1	0	2.618843	-6.213369	0.932452
181	1	0	3.763125	-4.869137	1.025470
182	6	0	1.565610	-4.213846	-0.411815
183	1	0	0.671190	-3.682096	-0.751678
184	1	0	2.443309	-3.744462	-0.867187
185	1	0	1.492644	-5.233177	-0.804391
186	8	0	1.433697	-0.267382	-2.793239
187	6	0	1.689137	-1.561408	-3.330633
188	6	0	0.835249	-1.823035	-4.531902
189	6	0	-0.019801	-1.054639	-5.255090
190	8	0	0.867202	-3.088525	-5.024336
191	6	0	-0.559897	-1.913026	-6.270475
192	1	0	-0.247048	-0.011698	-5.085337
193	6	0	0.010640	-3.127524	-6.082039
194	1	0	-1.278144	-1.650830	-7.033672
195	1	0	-0.076818	-4.079603	-6.582461
196	6	0	3.171204	-1.703299	-3.631363
197	1	0	3.532660	-0.840302	-4.197645
198	1	0	3.362170	-2.615035	-4.204166
199	8	0	3.841640	-1.796308	-2.370302
200	6	0	5.177666	-1.704154	-2.387537
201	8	0	5.806941	-1.424345	-3.390230
202	6	0	5.792108	-2.013054	-1.070788
203	6	0	7.186456	-1.996638	-0.971151
204	6	0	5.017816	-2.350520	0.042209
205	6	0	7.801534	-2.310223	0.235099
206	1	0	7.779872	-1.738663	-1.841909
207	6	0	5.638013	-2.681031	1.241449
208	1	0	3.937044	-2.354764	-0.025031
209	6	0	7.027556	-2.656499	1.341813
210	1	0	8.884002	-2.289750	0.311234
211	1	0	5.033276	-2.962043	2.095849
212	1	0	7.506659	-2.906009	2.283781
213	1	0	1.422827	-2.265192	-2.535589
214	1	0	-1.290481	-0.471470	-2.543759
215	1	0	1.604830	0.418669	-3.454046

2.6. Catalytic cycle 2a – 1c (*S*)

3a(*S*) -1c

RwB97XD SCF energy -4902.505584 a.u.
RwB97XD SCF enthalpy -4900.627323 a.u.
RwB97XD SCF free energy -4900.865109 a.u.
Three lowest frequencies (cm^{-1}) 13.2, 14.0, 18.4
Cartesian coordinates:
Standard orientation:

Center Number	Atomic Number	Atomic Type	Coordinates (Angstroms) X	Y	Z
1	6	0	-0.183389	-0.363954	2.579650
2	6	0	0.315453	-1.113821	3.622487
3	6	0	-0.043544	-2.437371	3.857376
4	6	0	-0.949497	-3.089926	3.058857
5	6	0	-1.478031	-2.354148	1.988734
6	6	0	-1.121375	-1.028445	1.740063
7	1	0	-1.240101	-4.117711	3.240467
8	1	0	-2.185236	-2.850461	1.333853
9	6	0	0.274983	1.050039	2.464387
10	6	0	1.144173	1.559861	1.461424
11	6	0	-0.083963	1.936910	3.454672
12	6	0	1.538452	2.895022	1.479966
13	6	0	0.317489	3.267913	3.467433
14	6	0	1.119527	3.787172	2.481128
15	1	0	2.210250	3.267242	0.714955
16	1	0	1.435465	4.823754	2.484292
17	8	0	0.603115	-2.892515	4.964470
18	8	0	1.197792	-0.708342	4.577206
19	8	0	-0.870477	1.689191	4.539855
20	8	0	-0.215486	3.886946	4.561071
21	6	0	1.597625	-1.904543	5.245741
22	1	0	2.553884	-2.241063	4.837886
23	1	0	1.645480	-1.725314	6.319333
24	6	0	-0.701589	2.826547	5.387099
25	1	0	0.043232	2.594224	6.157474
26	1	0	-1.662626	3.107105	5.817719
27	15	0	1.686098	0.396476	0.158627
28	15	0	-1.724767	-0.260752	0.194676
29	46	0	-0.074699	-0.280285	-1.312174
30	6	0	2.541000	-0.913148	1.081211
31	6	0	3.481731	-0.599731	2.058598
32	6	0	2.367187	-2.232815	0.697866
33	6	0	4.274245	-1.576181	2.659287
34	1	0	3.626704	0.442179	2.319563
35	6	0	3.075952	-3.275656	1.304284
36	1	0	1.664500	-2.448456	-0.101860
37	6	0	3.997143	-2.925256	2.315138
38	6	0	3.014975	1.204209	-0.792890
39	6	0	4.284235	0.634977	-0.876794
40	6	0	2.713921	2.298315	-1.596669
41	6	0	5.290645	1.202353	-1.653399
42	1	0	4.488625	-0.272021	-0.326902
43	6	0	3.679235	2.924002	-2.394247
44	1	0	1.697110	2.677265	-1.602830
45	6	0	4.992660	2.416645	-2.315364
46	6	0	-3.219383	-1.208451	-0.255053
47	6	0	-4.467955	-0.889468	0.277640
48	6	0	-3.122493	-2.247693	-1.167768
49	6	0	-5.603542	-1.632394	-0.030706
50	1	0	-4.555733	-0.038809	0.937151
51	6	0	-4.226494	-3.019560	-1.543574
52	1	0	-2.157002	-2.453479	-1.615723
53	6	0	-5.437567	-2.757092	-0.876183
54	6	0	-2.315893	1.408227	0.583527
55	6	0	-2.095052	2.424351	-0.333843
56	6	0	-3.033646	1.676171	1.752433
57	6	0	-2.639400	3.703587	-0.162966
58	1	0	-1.482931	2.212088	-1.205798
59	6	0	-3.660060	2.903533	1.946646
60	1	0	-3.113469	0.899826	2.503677
61	6	0	-3.523875	3.870782	0.918112
62	6	0	-2.177262	4.796829	-1.153226
63	6	0	-2.717291	4.493420	-2.562870
64	1	0	-2.396024	5.274356	-3.261524
65	1	0	-2.339292	3.538180	-2.940153
66	1	0	-3.811609	4.459194	-2.576904
67	6	0	-2.550811	6.238885	-0.768480
68	1	0	-2.269221	6.469998	0.262354
69	1	0	-2.000280	6.919617	-1.426967
70	1	0	-3.611883	6.457868	-0.893563
71	6	0	-0.632670	4.763649	-1.199645
72	1	0	-0.205508	4.996714	-0.218003
73	1	0	-0.241624	3.794533	-1.517550
74	1	0	-0.273505	5.510229	-1.915507
75	6	0	-4.396216	3.209820	3.267942
76	6	0	-3.728184	4.431285	3.929751
77	1	0	-3.858087	5.333887	3.328823
78	1	0	-4.171551	4.612365	4.915516
79	1	0	-2.654746	4.265797	4.061570
80	6	0	-5.896731	3.490971	3.062807
81	1	0	-6.376789	2.691752	2.488067
82	1	0	-6.391593	3.540389	4.039147
83	1	0	-6.074388	4.441718	2.560701
84	6	0	-4.294769	2.029332	4.248046
85	1	0	-4.805378	1.137178	3.868254
86	1	0	-3.257634	1.765589	4.472222
87	1	0	-4.779652	2.307188	5.189068
88	8	0	-4.281063	5.012579	1.015136
89	8	0	-6.516453	-3.589252	-1.050842
90	8	0	4.679678	-3.932709	2.947943
91	8	0	6.033946	3.085685	-2.915387
92	6	0	6.514319	4.185546	-2.147508
93	1	0	7.402349	4.559558	-2.660508
94	1	0	5.772518	4.987686	-2.080535
95	1	0	6.781786	3.874984	-1.131574
96	6	0	3.191521	4.077496	-3.302941
97	6	0	4.159173	4.481658	-4.429710
98	1	0	3.634357	5.172171	-5.098811
99	1	0	5.051674	4.995088	-4.073113
100	1	0	4.472744	3.617216	-5.022345
101	6	0	2.872702	5.310754	-2.438323
102	1	0	2.453283	6.110075	-3.059837
103	1	0	2.142633	5.069739	-1.659438
104	1	0	3.769749	5.701629	-1.947574
105	6	0	-5.417241	4.998528	0.154197
106	1	0	-5.961115	5.927036	0.337212
107	1	0	-5.127709	4.952609	-0.900196
108	1	0	-6.067453	4.144555	0.373790
109	6	0	-6.432728	-4.776332	-0.266111
110	1	0	-7.362982	-5.324249	-0.426167
111	1	0	-5.587236	-5.403356	-0.567457
112	1	0	-6.326126	-4.540708	0.798147
113	6	0	-6.988859	-1.158596	0.457965
114	6	0	-6.881246	0.150359	1.260649
115	1	0	-6.313954	0.020235	2.189089
116	1	0	-6.419235	0.956446	0.680872
117	1	0	-7.888059	0.480126	1.535489
118	6	0	-7.871171	-0.871826	-0.773335
119	1	0	-8.020773	-1.769927	-1.376646
120	1	0	-8.854035	-0.508260	-0.452542
121	1	0	-7.416895	-0.101626	-1.406373
122	6	0	-7.684277	-2.189952	1.365696
123	1	0	-8.016285	-3.067565	0.810428
124	1	0	-7.023235	-2.515860	2.175868
125	1	0	-8.570891	-1.736071	1.822106
126	6	0	-3.992267	-4.029373	-2.691974
127	6	0	-3.081640	-5.166259	-2.193250
128	1	0	-2.132643	-4.778674	-1.807582
129	1	0	-3.561397	-5.735785	-1.390254
130	1	0	-2.853411	-5.859485	-3.010638
131	6	0	-5.262522	-4.634090	-3.314912
132	1	0	-4.971532	-5.186709	-4.215184
133	1	0	-5.776832	-5.337722	-2.660015
134	1	0	-5.973895	-3.860150	-3.615650
135	6	0	-3.281369	-3.276160	-3.842340
136	1	0	-3.093726	-3.965883	-4.671937
137	1	0	-3.910672	-2.461209	-4.216163
138	1	0	-2.318371	-2.852612	-3.548835
139	6	0	6.640126	0.470294	-1.817138
140	6	0	1.893525	3.622850	-4.009487
141	1	0	2.061663	2.705202	-4.583317
142	1	0	1.071086	3.442235	-3.315530
143	1	0	1.561864	4.402250	-4.703437
144	6	0	6.921721	0.251336	-3.316826
145	1	0	7.865458	-0.291428	-3.441755
146	1	0	6.125933	-0.344447	-3.777617
147	1	0	7.000632	1.197265	-3.855275
148	6	0	7.803923	1.245536	-1.173582

149	1	0	7.582167	1.496407	-0.130461
150	1	0	8.707438	0.625145	-1.181225
151	1	0	8.035911	2.165437	-1.711771
152	6	0	6.603212	-0.921314	-1.159682
153	1	0	5.803599	-1.550307	-1.565809
154	1	0	7.553249	-1.429553	-1.352902
155	1	0	6.479628	-0.868317	-0.072707
156	6	0	4.106887	-4.362708	4.174475
157	1	0	4.589242	-5.307155	4.434310
158	1	0	4.292464	-3.640572	4.977589
159	1	0	3.026182	-4.520889	4.078691
160	6	0	5.411461	-1.085093	3.593835
161	6	0	4.855380	-0.744226	4.989059
162	1	0	5.632153	-0.255879	5.588514
163	1	0	4.003050	-0.059616	4.920385
164	1	0	4.536447	-1.638134	5.532612
165	6	0	6.581258	-2.074206	3.741158
166	1	0	7.399452	-1.570304	4.267244
167	1	0	6.322478	-2.963966	4.314003
168	1	0	6.960895	-2.395128	2.765688
169	6	0	6.020771	0.209729	3.004407
170	1	0	6.321562	0.072692	1.960241
171	1	0	5.338165	1.062072	3.052318
172	1	0	6.911076	0.484833	3.578748
173	6	0	2.823539	-4.684103	0.711526
174	6	0	1.311383	-4.988757	0.754657
175	1	0	1.121599	-5.978217	0.323493
176	1	0	0.946654	-4.995956	1.787498
177	1	0	0.713890	-4.263616	0.193693
178	6	0	3.536527	-5.866632	1.385855
179	1	0	3.208068	-6.020570	2.416109
180	1	0	3.283176	-6.774795	0.826826
181	1	0	4.621953	-5.759662	1.377531
182	6	0	3.305123	-4.651813	-0.755624
183	1	0	2.804986	-3.875412	-1.340262
184	1	0	4.383598	-4.464156	-0.803334
185	1	0	3.110533	-5.616130	-1.238638
186	8	0	1.276885	-0.506124	-3.068705
187	6	0	0.754308	-1.125706	-4.012262
188	1	0	-1.280699	-0.645925	-2.224506
189	6	0	0.542970	-2.543166	-3.974935
190	6	0	0.127858	-3.446389	-4.925396
191	8	0	0.772263	-3.193235	-2.795264
192	6	0	0.106260	-4.711142	-4.293178
193	1	0	-0.127710	-3.222040	-5.951466
194	6	0	0.498938	-4.489696	-3.003257
195	1	0	-0.169470	-5.660542	-4.726972
196	1	0	0.628258	-5.145062	-2.154449
197	6	0	0.247825	-0.366374	-5.229962
198	1	0	0.429680	-0.907830	-6.157804
199	1	0	0.714673	0.617593	-5.262528
200	8	0	-1.177245	-0.261048	-5.095702
201	6	0	-1.660824	0.809797	-4.431274
202	8	0	-0.957808	1.722159	-4.049423
203	6	0	-3.133916	0.694167	-4.203399
204	1	0	-3.626472	0.172283	-5.025012
205	1	0	-3.281650	0.114695	-3.283902
206	1	0	-3.565499	1.686074	-4.063826

TS1a(S) -1c

RwB97XD SCF energy -4902.496336 a.u.
RwB97XD SCF enthalpy -4900.619680 a.u.
RwB97XD SCF free energy -4900.855354 a.u.
Three lowest frequencies (cm^{-1}) -455.7, 11.2, 13.7
Imaginary frequency (cm^{-1}) -455.7
Cartesian coordinates:

Center Number	Atomic Number	Atomic Type	Coordinates (Angstroms) X	Y	Z
1	6	0	-0.155995	-0.315953	2.549660
2	6	0	0.312359	-1.071465	3.603977
3	6	0	-0.074787	-2.390059	3.834055
4	6	0	-0.973088	-3.030031	3.017078
5	6	0	-1.462607	-2.290762	1.930971
6	6	0	-1.080250	-0.972078	1.685890
7	1	0	-1.287262	-4.051539	3.195105
8	1	0	-2.161526	-2.780072	1.262770
9	6	0	0.315135	1.094096	2.441297
10	6	0	1.156347	1.607754	1.416703
11	6	0	-0.007683	1.980280	3.444890
12	6	0	1.561632	2.939527	1.422987
13	6	0	0.412104	3.307974	3.452208
14	6	0	1.183006	3.828719	2.441474
15	1	0	2.212940	3.306691	0.637872
16	1	0	1.507109	4.862627	2.440263
17	8	0	0.536202	-2.851498	4.954806
18	8	0	1.181170	-0.683098	4.574014
19	8	0	-0.770708	1.742718	4.546062
20	8	0	-0.072955	3.926146	4.562978
21	6	0	1.553014	-1.888834	5.238565
22	1	0	2.503377	-2.247555	4.831738
23	1	0	1.607227	-1.714451	6.312788
24	6	0	-0.553610	2.868147	5.395498
25	1	0	0.208962	2.617003	6.143171
26	1	0	-1.494764	3.164336	5.859011
27	15	0	1.707308	0.450530	0.124954
28	15	0	-1.632275	-0.201467	0.119549
29	46	0	0.078712	-0.334957	-1.367159
30	6	0	2.554432	-0.865501	1.041076
31	6	0	3.465298	-0.537632	2.041580
32	6	0	2.422648	-2.184709	0.638482
33	6	0	4.276010	-1.498197	2.643124
34	1	0	3.573138	0.503616	2.321267
35	6	0	3.147315	-3.214160	1.248546
36	1	0	1.742164	-2.411354	-0.177140
37	6	0	4.045691	-2.850427	2.276874
38	6	0	3.014113	1.246789	-0.860007
39	6	0	4.291599	0.693566	-0.920585
40	6	0	2.683764	2.285888	-1.722798
41	6	0	5.276181	1.221330	-1.751114
42	1	0	4.518402	-0.170412	-0.313430
43	6	0	3.622115	2.858337	-2.588443
44	1	0	1.662426	2.653156	-1.730424
45	6	0	4.944542	2.374302	-2.501295
46	6	0	-3.140889	-1.120334	-0.342125
47	6	0	-4.383540	-0.740285	0.167445
48	6	0	-3.063964	-2.245499	-1.151756
49	6	0	-5.520606	-1.518032	-0.021230
50	1	0	-4.462719	0.179897	0.726868
51	6	0	-4.172522	-3.068934	-1.391246
52	1	0	-2.111105	-2.497970	-1.606632
53	6	0	-5.362280	-2.743529	-0.713061
54	6	0	-2.203521	1.474579	0.510708
55	6	0	-2.009288	2.492800	-0.409802
56	6	0	-2.899491	1.734704	1.694707
57	6	0	-2.542819	3.774277	-0.213087
58	1	0	-1.437236	2.280408	-1.307302
59	6	0	-3.523350	2.959287	1.910572
60	1	0	-2.966343	0.951917	2.440139
61	6	0	-3.397464	3.939320	0.893227
62	6	0	-2.113731	4.879127	-1.206361
63	6	0	-2.790425	4.658147	-2.571163
64	1	0	-2.428452	5.401255	-3.291087
65	1	0	-2.570391	3.662697	-2.967725
66	1	0	-3.877913	4.762240	-2.504470
67	6	0	-2.371670	6.319876	-0.728786
68	1	0	-1.971784	6.487334	0.275423
69	1	0	-1.857636	7.003668	-1.413153
70	1	0	-3.425995	6.595012	-0.726134
71	6	0	-0.584821	4.774173	-1.401254
72	1	0	-0.056389	4.911069	-0.451263
73	1	0	-0.279993	3.814987	-1.824788
74	1	0	-0.251873	5.552933	-2.094490
75	6	0	-4.248122	3.247598	3.242479
76	6	0	-3.560534	4.444971	3.926944
77	1	0	-3.676339	5.357269	3.337926
78	1	0	-4.002926	4.616573	4.914921
79	1	0	-2.490251	4.261045	4.057130
80	6	0	-5.744793	3.561355	3.052533
81	1	0	-6.239671	2.797914	2.443456
82	1	0	-6.237019	3.575163	4.031183
83	1	0	-5.905139	4.536340	2.593234
84	6	0	-4.163595	2.042093	4.193508
85	1	0	-4.687911	1.167489	3.791451
86	1	0	-3.130084	1.757515	4.409167
87	1	0	-4.643712	2.303892	5.141496
88	8	0	-4.127881	5.090924	1.035088
89	8	0	-6.419815	-3.615820	-0.722864
90	8	0	4.753462	-3.842845	2.899523
91	8	0	5.957865	3.000077	-3.182983

92	6	0	6.435773	4.169718	-2.527455
93	1	0	7.303393	4.514934	-3.092804
94	1	0	5.680966	4.962144	-2.509569
95	1	0	6.735248	3.953099	-1.496198
96	6	0	3.098496	3.922402	-3.582805
97	6	0	4.037248	4.244405	-4.759694
98	1	0	3.488505	4.873609	-5.468904
99	1	0	4.931720	4.792634	-4.465339
100	1	0	4.349827	3.338156	-5.285902
101	6	0	2.776968	5.220532	-2.820160
102	1	0	2.323288	5.954151	-3.496109
103	1	0	2.076121	5.036324	-2.000764
104	1	0	3.679594	5.669202	-2.393608
105	6	0	-5.283061	5.132349	0.203263
106	1	0	-5.822219	6.045903	0.460631
107	1	0	-5.020259	5.160046	-0.858209
108	1	0	-5.929654	4.265914	0.379472
109	6	0	-6.264783	-4.676305	0.217568
110	1	0	-7.138016	-5.321786	0.108882
111	1	0	-5.356923	-5.257463	0.024514
112	1	0	-6.218894	-4.292303	1.242516
113	6	0	-6.897241	-1.004543	0.449360
114	6	0	-6.786227	0.402658	1.060827
115	1	0	-6.186760	0.406266	1.978112
116	1	0	-6.355782	1.126994	0.361103
117	1	0	-7.788046	0.755977	1.324086
118	6	0	-7.829777	-0.910181	-0.774277
119	1	0	-7.970355	-1.886710	-1.242827
120	1	0	-8.811818	-0.533127	-0.467035
121	1	0	-7.422651	-0.219853	-1.521483
122	6	0	-7.536339	-1.913886	1.515594
123	1	0	-7.869310	-2.862781	1.094291
124	1	0	-6.838914	-2.115286	2.335829
125	1	0	-8.414883	-1.415860	1.940344
126	6	0	-3.971273	-4.216146	-2.409101
127	6	0	-2.930194	-5.208555	-1.857849
128	1	0	-1.969807	-4.724075	-1.654718
129	1	0	-3.281820	-5.668019	-0.927762
130	1	0	-2.751324	-6.008730	-2.584554
131	6	0	-5.231448	-5.002383	-2.811567
132	1	0	-4.966858	-5.649005	-3.655833
133	1	0	-5.608807	-5.650635	-2.020412
134	1	0	-6.039217	-4.342036	-3.134480
135	6	0	-3.445155	-3.583224	-3.715373
136	1	0	-3.202113	-4.370300	-4.437062
137	1	0	-4.208842	-2.938649	-4.162408
138	1	0	-2.544567	-2.985905	-3.567332
139	6	0	6.635876	0.502883	-1.886818
140	6	0	1.797748	3.386503	-4.227480
141	1	0	1.979627	2.434455	-4.737875
142	1	0	0.990804	3.239293	-3.506814
143	1	0	1.434900	4.105800	-4.969009
144	6	0	6.873856	0.151176	-3.368866
145	1	0	7.828137	-0.376724	-3.476657
146	1	0	6.081422	-0.505812	-3.744331
147	1	0	6.906260	1.046864	-3.991681
148	6	0	7.803267	1.361927	-1.366727
149	1	0	7.606489	1.729686	-0.353938
150	1	0	8.716797	0.757728	-1.331233
151	1	0	8.000816	2.215108	-2.016240
152	6	0	6.651870	-0.819248	-1.098497
153	1	0	5.858332	-1.503007	-1.418458
154	1	0	7.608940	-1.321902	-1.269841
155	1	0	6.555555	-0.664360	-0.018588
156	6	0	4.187059	-4.307586	4.114464
157	1	0	4.706581	-5.231928	4.374351
158	1	0	4.327875	-3.584647	4.926089
159	1	0	3.115829	-4.513581	4.005021
160	6	0	5.379845	-0.985213	3.606372
161	6	0	4.786984	-0.682855	4.995483
162	1	0	5.536406	-0.179068	5.616320
163	1	0	3.914340	-0.025800	4.918492
164	1	0	4.486999	-1.594165	5.520164
165	6	0	6.578272	-1.939277	3.759754
166	1	0	7.370910	-1.416537	4.306089
167	1	0	6.338719	-2.845357	4.314685
168	1	0	6.981391	-2.234772	2.786325
169	6	0	5.959569	0.337832	3.050829
170	1	0	6.298492	0.224471	2.015745
171	1	0	5.246883	1.165430	3.087826
172	1	0	6.821123	0.635293	3.656834
173	6	0	2.933617	-4.625726	0.646627
174	6	0	1.428746	-4.966698	0.678819
175	1	0	1.267039	-5.962465	0.250944
176	1	0	1.055336	-4.978291	1.708488
177	1	0	0.819356	-4.260211	0.107462
178	6	0	3.670588	-5.792689	1.322947
179	1	0	3.324875	-5.966607	2.344381
180	1	0	3.457019	-6.702440	0.750366
181	1	0	4.751100	-5.647617	1.342536
182	6	0	3.421532	-4.579855	-0.817969
183	1	0	2.901495	-3.820015	-1.406899
184	1	0	4.494958	-4.364848	-0.860545
185	1	0	3.254833	-5.550193	-1.299312
186	8	0	1.400031	-0.672472	-2.974254
187	6	0	0.369686	-1.155884	-3.572147
188	1	0	-0.913966	-0.944780	-2.493784
189	6	0	0.250216	-2.617813	-3.714522
190	6	0	-0.182948	-3.411007	-4.734195
191	8	0	0.700733	-3.380222	-2.685051
192	6	0	0.020352	-4.756644	-4.302217
193	1	0	-0.601300	-3.076548	-5.672505
194	6	0	0.548679	-4.672323	-3.052627
195	1	0	-0.204995	-5.662361	-4.845765
196	1	0	0.858826	-5.409088	-2.327472
197	6	0	-0.281982	-0.304558	-4.660590
198	1	0	0.130717	-0.605462	-5.627040
199	1	0	-0.045316	0.741214	-4.467240
200	8	0	-1.689482	-0.509568	-4.722078
201	6	0	-2.465090	0.434678	-4.141202
202	8	0	-2.017663	1.457326	-3.671712
203	6	0	-3.907609	0.037936	-4.134490
204	1	0	-4.173314	-0.514830	-5.036706
205	1	0	-4.077446	-0.614142	-3.270444
206	1	0	-4.528766	0.927446	-4.030349

4a(*S*) -1c

RwB97XD SCF energy -4902.511949 a.u.
RwB97XD SCF enthalpy -4900.629224 a.u.
RwB97XD SCF free energy -4900.864894 a.u.
Three lowest frequencies (cm^{-1}) 11.0, 16.4, 20.2
Cartesian coordinates:

Center	Atomic	Atomic	Coordinates	(Angstroms)	
Number	Number	Type	X	Y	Z
1	6	0	0.193429	-2.256732	1.912154
2	6	0	-0.288129	-2.834816	3.065387
3	6	0	0.043766	-2.372441	4.336834
4	6	0	0.855737	-1.280972	4.527965
5	6	0	1.339469	-0.652423	3.371120
6	6	0	1.031768	-1.123057	2.098045
7	1	0	1.119670	-0.929908	5.518372
8	1	0	1.987693	0.208971	3.486546
9	6	0	-0.060682	-2.905107	0.595625
10	6	0	-0.745399	-2.347281	-0.523015
11	6	0	0.449655	-4.172133	0.412184
12	6	0	-0.853813	-3.065634	-1.712761
13	6	0	0.344864	-4.873996	-0.784832
14	6	0	-0.301236	-4.344541	-1.874254
15	1	0	-1.392868	-2.644030	-2.551649
16	1	0	-0.392308	-4.884778	-2.808845
17	8	0	-0.545589	-3.160576	5.271447
18	8	0	-1.115337	-3.908587	3.173725
19	8	0	1.159073	-4.910956	1.308769
20	8	0	0.981153	-6.068890	-0.664812
21	6	0	-1.098613	-4.269517	4.556180
22	1	0	-2.115476	-4.453401	4.901557
23	1	0	-0.455767	-5.145576	4.695091
24	6	0	1.261592	-6.213338	0.730555
25	1	0	0.511926	-6.872060	1.183555
26	1	0	2.274268	-6.593248	0.863891
27	15	0	-1.423609	-0.656458	-0.413649
28	15	0	1.610355	-0.242451	0.628356
29	46	0	0.126136	0.949168	-0.589415
30	6	0	-2.321134	-0.618376	1.155232
31	6	0	-3.277289	-1.595578	1.398931
32	6	0	-2.070331	0.364864	2.108163
33	6	0	-3.996976	-1.636848	2.596413
34	1	0	-3.472933	-2.340190	0.634380
35	6	0	-2.703808	0.344211	3.348222

36	1	0	-1.345812	1.131757	1.867782
37	6	0	-3.611824	-0.716322	3.594126
38	6	0	-2.589498	-0.402965	-1.773689
39	6	0	-3.920057	-0.059778	-1.543822
40	6	0	-2.076772	-0.338349	-3.064334
41	6	0	-4.775914	0.239634	-2.602834
42	1	0	-4.284542	-0.016615	-0.526365
43	6	0	-2.875654	-0.037587	-4.167265
44	1	0	-1.014260	-0.497506	-3.213689
45	6	0	-4.250637	0.145328	-3.914771
46	6	0	2.734568	1.094098	1.098367
47	6	0	4.038557	1.118720	0.608969
48	6	0	2.227851	2.207295	1.762867
49	6	0	4.876510	2.203889	0.838156
50	1	0	4.395989	0.275013	0.036592
51	6	0	3.013360	3.333005	2.025822
52	1	0	1.190542	2.196667	2.080813
53	6	0	4.367701	3.265164	1.629396
54	6	0	2.570796	-1.374687	-0.394870
55	6	0	2.543347	-1.183173	-1.767712
56	6	0	3.391953	-2.359317	0.154355
57	6	0	3.390074	-1.886864	-2.628987
58	1	0	1.851190	-0.454316	-2.179843
59	6	0	4.275594	-3.076742	-0.645853
60	1	0	3.341060	-2.549934	1.219666
61	6	0	4.334430	-2.733804	-2.021804
62	6	0	3.162486	-1.670991	-4.142122
63	6	0	3.508641	-0.217516	-4.514902
64	1	0	3.323144	-0.050224	-5.581709
65	1	0	2.899912	0.499774	-3.954205
66	1	0	4.562244	0.004875	-4.316345
67	6	0	3.929317	-2.622967	-5.074853
68	1	0	3.769749	-3.670821	-4.807158
69	1	0	3.550872	-2.477975	-6.092784
70	1	0	5.002496	-2.431072	-5.101053
71	6	0	1.662806	-1.916402	-4.428819
72	1	0	1.387155	-2.952419	-4.203322
73	1	0	1.007803	-1.259619	-3.850581
74	1	0	1.459148	-1.732100	-5.488655
75	6	0	5.078414	-4.256623	-0.062440
76	6	0	4.708827	-5.526298	-0.856202
77	1	0	5.022156	-5.452021	-1.899941
78	1	0	5.197404	-6.402250	-0.414997
79	1	0	3.626572	-5.693233	-0.838190
80	6	0	6.600386	-4.030071	-0.116156
81	1	0	6.873843	-3.073045	0.341091
82	1	0	7.105885	-4.823740	0.445453
83	1	0	6.986197	-4.054549	-1.135290
84	6	0	4.710198	-4.503640	1.409599
85	1	0	5.009594	-3.670318	2.054989
86	1	0	3.638384	-4.676495	1.545618
87	1	0	5.238530	-5.394885	1.762294
88	8	0	5.355390	-3.267464	-2.768672
89	8	0	5.240019	4.266777	1.972390
90	8	0	-4.146087	-0.810063	4.854292
91	8	0	-5.128837	0.279839	-4.961569
92	6	0	-5.499476	-0.966103	-5.546874
93	1	0	-6.219823	-0.738842	-6.334626
94	1	0	-4.637397	-1.479865	-5.983382
95	1	0	-5.962418	-1.628943	-4.808224
96	6	0	-2.149827	0.111930	-5.525867
97	6	0	-2.966695	0.788126	-6.641073
98	1	0	-2.294944	0.984209	-7.483883
99	1	0	-3.782744	0.171875	-7.018427
100	1	0	-3.379650	1.746527	-6.314213
101	6	0	-1.681124	-1.273823	-6.006861
102	1	0	-1.108047	-1.175790	-6.935741
103	1	0	-1.040894	-1.758851	-5.262886
104	1	0	-2.527622	-1.939144	-6.205040
105	6	0	6.420485	-2.339700	-2.973943
106	1	0	7.190440	-2.867075	-3.539996
107	1	0	6.088809	-1.464232	-3.541489
108	1	0	6.837679	-2.001171	-2.019108
109	6	0	5.742906	4.161904	3.302879
110	1	0	6.496882	4.943260	3.412567
111	1	0	4.956422	4.316366	4.047407
112	1	0	6.201214	3.182864	3.476473
113	6	0	6.257291	2.255208	0.148574
114	6	0	6.495726	1.006185	-0.722406
115	1	0	6.535327	0.087783	-0.125779
116	1	0	5.731827	0.883116	-1.497907
117	1	0	7.460355	1.110001	-1.228688
118	6	0	6.289534	3.482046	-0.784134
119	1	0	6.153220	4.412763	-0.229092
120	1	0	7.254003	3.532754	-1.301869
121	1	0	5.502246	3.414467	-1.543232
122	6	0	7.424638	2.332658	1.149749
123	1	0	7.478158	3.301374	1.646770
124	1	0	7.349247	1.549799	1.912118
125	1	0	8.370877	2.186715	0.617254
126	6	0	2.301791	4.536147	2.692264
127	6	0	2.069530	4.235171	4.183989
128	1	0	1.485507	3.317160	4.310908
129	1	0	3.010189	4.111695	4.728691
130	1	0	1.514949	5.056689	4.651183
131	6	0	3.029688	5.882422	2.528961
132	1	0	2.359449	6.679362	2.868485
133	1	0	3.944994	5.959835	3.114647
134	1	0	3.271918	6.074640	1.479142
135	6	0	0.922691	4.733949	2.022928
136	1	0	0.433760	5.614143	2.452460
137	1	0	1.040645	4.895248	0.949069
138	1	0	0.242804	3.893135	2.170611
139	6	0	-6.200282	0.767555	-2.327661
140	6	0	-0.907947	1.012628	-5.321723
141	1	0	-1.197719	1.999988	-4.946769
142	1	0	-0.174558	0.591358	-4.629853
143	1	0	-0.399109	1.148687	-6.281883
144	6	0	-6.294719	2.201322	-2.887053
145	1	0	-7.291825	2.614328	-2.696413
146	1	0	-5.559668	2.852974	-2.401138
147	1	0	-6.118094	2.224452	-3.964984
148	6	0	-7.302641	-0.110172	-2.950167
149	1	0	-7.166626	-1.165295	-2.689411
150	1	0	-8.277650	0.205002	-2.562192
151	1	0	-7.338980	-0.018849	-4.035763
152	6	0	-6.487683	0.842550	-0.820034
153	1	0	-5.778383	1.489638	-0.295609
154	1	0	-7.486227	1.264689	-0.669309
155	1	0	-6.472965	-0.146345	-0.350855
156	6	0	-3.547372	-1.827340	5.654471
157	1	0	-4.039393	-1.785194	6.627745
158	1	0	-3.695454	-2.819694	5.219634
159	1	0	-2.475195	-1.655064	5.778053
160	6	0	-5.151968	-2.665492	2.673383
161	6	0	-4.575606	-4.091207	2.743308
162	1	0	-5.389360	-4.824694	2.722709
163	1	0	-3.911402	-4.301115	1.898822
164	1	0	-4.008652	-4.250257	3.665342
165	6	0	-6.147091	-2.461617	3.830532
166	1	0	-6.999332	-3.129660	3.665868
167	1	0	-5.735444	-2.704155	4.809622
168	1	0	-6.528194	-1.436985	3.859627
169	6	0	-6.000536	-2.535753	1.387033
170	1	0	-6.447197	-1.538367	1.322626
171	1	0	-5.436423	-2.718270	0.469288
172	1	0	-6.814483	-3.267518	1.414934
173	6	0	-2.469784	1.487572	4.360707
174	6	0	-1.915375	0.990106	5.709222
175	1	0	-1.560031	1.846918	6.291986
176	1	0	-2.674806	0.482757	6.304489
177	1	0	-1.069248	0.311562	5.564987
178	6	0	-3.800175	2.226590	4.601732
179	1	0	-4.569591	1.550877	4.981926
180	1	0	-3.653883	3.024079	5.339338
181	1	0	-4.167082	2.688385	3.679530
182	6	0	-1.459960	2.507971	3.810835
183	1	0	-0.473558	2.054600	3.659928
184	1	0	-1.792060	2.952976	2.867678
185	1	0	-1.341391	3.322605	4.532033
186	8	0	-0.897073	2.193730	-1.815348
187	6	0	-0.941604	3.487501	-1.287788
188	6	0	-2.289248	3.768243	-0.689990
189	6	0	-3.274802	4.681603	-0.898862
190	8	0	-2.676911	2.892600	0.277525
191	6	0	-4.343323	4.339533	-0.002516
192	1	0	-3.248259	5.501563	-1.602717
193	6	0	-3.924979	3.244130	0.679280
194	1	0	-5.294958	4.839697	0.107729
195	1	0	-4.371477	2.617318	1.436061
196	6	0	-0.589207	4.475359	-2.402895
197	1	0	-1.416625	4.594785	-3.104783
198	1	0	0.286793	4.098777	-2.932907
199	8	0	-0.320664	5.788789	-1.887526

200	6	0	0.916443	6.014987	-1.424284
201	8	0	1.804860	5.183442	-1.454870
202	1	0	-0.182036	3.620235	-0.494041
203	6	0	1.048903	7.390170	-0.845921
204	1	0	2.099437	7.614399	-0.663682
205	1	0	0.610854	8.133811	-1.515501
206	1	0	0.500784	7.425099	0.101247

5a(*S*) -1c

RwB97XD SCF energy -4903.694458 a.u.
RwB97XD SCF enthalpy -4901.794956 a.u.
RwB97XD SCF free energy -4902.032167 a.u.
Three lowest frequencies (cm^{-1}) 11.0, 16.4, 20.2
Cartesian coordinates:

Center Number	Atomic Number	Atomic Type	Coordinates (Angstroms) X	Y	Z
1	6	0	0.659438	-0.401367	2.691802
2	6	0	0.443453	-0.046224	4.005357
3	6	0	0.939415	1.128113	4.563993
4	6	0	1.650294	2.038093	3.821161
5	6	0	1.865558	1.714604	2.473337
6	6	0	1.399595	0.527455	1.911070
7	1	0	2.044436	2.949047	4.255401
8	1	0	2.439420	2.409416	1.872010
9	6	0	0.241817	-1.750647	2.229060
10	6	0	-0.668417	-2.053895	1.178695
11	6	0	0.809666	-2.834182	2.864113
12	6	0	-0.922691	-3.381281	0.832773
13	6	0	0.555692	-4.152768	2.503529
14	6	0	-0.306716	-4.462102	1.480740
15	1	0	-1.632204	-3.609382	0.047531
16	1	0	-0.515417	-5.486116	1.195084
17	8	0	0.609726	1.177466	5.880317
18	8	0	-0.234873	-0.747302	4.954308
19	8	0	1.714815	-2.810552	3.880800
20	8	0	1.291268	-4.985142	3.289067
21	6	0	0.087485	-0.116356	6.194472
22	1	0	-0.813418	-0.008941	6.797814
23	1	0	0.857694	-0.701369	6.710023
24	6	0	1.795447	-4.159185	4.342790
25	1	0	1.163339	-4.277867	5.230254
26	1	0	2.836130	-4.412071	4.543995
27	15	0	-1.462323	-0.699431	0.241231
28	15	0	1.709088	0.170653	0.150997
29	46	0	-0.088986	0.297140	-1.287616
30	6	0	-2.050514	0.482841	1.475723
31	6	0	-2.841644	0.067927	2.539102
32	6	0	-1.704168	1.826275	1.354048
33	6	0	-3.273021	0.963382	3.524302
34	1	0	-3.128755	-0.976805	2.603383
35	6	0	-2.062149	2.760209	2.318812
36	1	0	-1.132327	2.134068	0.489306
37	6	0	-2.768929	2.278995	3.447223
38	6	0	-2.864446	-1.418325	-0.652778
39	6	0	-4.185274	-1.061446	-0.395895
40	6	0	-2.571206	-2.226437	-1.745379
41	6	0	-5.229706	-1.580792	-1.162808
42	1	0	-4.396321	-0.363153	0.403016
43	6	0	-3.563114	-2.762251	-2.565593
44	1	0	-1.530631	-2.418083	-1.983091
45	6	0	-4.897051	-2.495307	-2.191371
46	6	0	2.920316	1.404055	-0.405381
47	6	0	4.248529	1.060679	-0.631866
48	6	0	2.490165	2.696579	-0.682376
49	6	0	5.180405	2.007191	-1.049561
50	1	0	4.558000	0.036865	-0.478085
51	6	0	3.369757	3.698913	-1.100254
52	1	0	1.436859	2.928002	-0.562297
53	6	0	4.735281	3.344959	-1.184297
54	6	0	2.546349	-1.426713	0.048909
55	6	0	2.275390	-2.238285	-1.041185
56	6	0	3.504036	-1.819030	0.985622
57	6	0	3.001539	-3.407766	-1.290955
58	1	0	1.479784	-1.948485	-1.720888
59	6	0	4.287089	-2.949325	0.775605
60	1	0	3.644702	-1.213732	1.872769
61	6	0	4.085872	-3.664866	-0.433147
62	6	0	2.509861	-4.293434	-2.458433
63	6	0	2.737867	-3.567911	-3.797341
64	1	0	2.360426	-4.180515	-4.623681
65	1	0	2.213895	-2.606417	-3.828566
66	1	0	3.801283	-3.379144	-3.976565
67	6	0	3.135391	-5.696715	-2.532852
68	1	0	3.056615	-6.224534	-1.578429
69	1	0	2.585513	-6.276508	-3.282221
70	1	0	4.182395	-5.688149	-2.837004
71	6	0	0.991686	-4.517446	-2.271260
72	1	0	0.785287	-5.037621	-1.329784
73	1	0	0.425524	-3.582689	-2.271923
74	1	0	0.609921	-5.131780	-3.092974
75	6	0	5.268848	-3.435204	1.860799
76	6	0	4.887109	-4.877156	2.251541
77	1	0	5.060561	-5.573899	1.428850
78	1	0	5.485040	-5.206105	3.108809
79	1	0	3.829515	-4.938709	2.526997
80	6	0	6.736902	-3.393123	1.396700
81	1	0	6.997964	-2.408480	0.993158
82	1	0	7.394459	-3.589111	2.250977
83	1	0	6.951633	-4.148436	0.640656
84	6	0	5.172027	-2.565167	3.125154
85	1	0	5.493454	-1.534304	2.939475
86	1	0	4.158389	-2.542305	3.536717
87	1	0	5.834457	-2.978739	3.891862
88	8	0	4.994231	-4.645065	-0.748620
89	8	0	5.687838	4.301372	-1.437210
90	8	0	-2.972779	3.151313	4.486870
91	8	0	-5.933752	-3.095443	-2.863547
92	6	0	-6.189652	-4.432840	-2.442384
93	1	0	-7.075132	-4.767939	-2.985592
94	1	0	-5.353196	-5.098334	-2.675921
95	1	0	-6.382704	-4.480601	-1.365669
96	6	0	-3.087079	-3.541597	-3.816250
97	6	0	-4.147791	-3.708529	-4.919336
98	1	0	-3.656679	-4.112953	-5.810938
99	1	0	-4.946342	-4.399677	-4.652520
100	1	0	-4.598398	-2.749795	-5.192617
101	6	0	-2.565327	-4.926178	-3.389659
102	1	0	-2.137167	-5.449145	-4.252249
103	1	0	-1.785690	-4.836689	-2.626122
104	1	0	-3.362646	-5.553705	-2.980032
105	6	0	5.945329	-4.217917	-1.722663
106	1	0	6.645154	-5.044053	-1.860544
107	1	0	5.466685	-3.984234	-2.678917
108	1	0	6.489138	-3.330572	-1.379602
109	6	0	6.077237	5.047343	-0.286279
110	1	0	6.968280	5.612450	-0.566373
111	1	0	5.297414	5.747722	0.026302
112	1	0	6.311808	4.385808	0.553822
113	6	0	6.612913	1.557070	-1.409390
114	6	0	6.762237	0.028056	-1.293052
115	1	0	6.635923	-0.324216	-0.262999
116	1	0	6.053774	-0.509291	-1.932632
117	1	0	7.770714	-0.253703	-1.611574
118	6	0	6.903229	1.934752	-2.875089
119	1	0	6.843247	3.013499	-3.031899
120	1	0	7.910526	1.604863	-3.153206
121	1	0	6.190910	1.447783	-3.550160
122	6	0	7.676308	2.183957	-0.488418
123	1	0	7.806928	3.250071	-0.674811
124	1	0	7.422924	2.039960	0.567333
125	1	0	8.643331	1.700227	-0.666028
126	6	0	2.752299	5.080442	-1.428283
127	6	0	2.432432	5.819676	-0.115062
128	1	0	1.783509	5.215921	0.527369
129	1	0	3.337754	6.053997	0.452442
130	1	0	1.916071	6.762583	-0.327815
131	6	0	3.624457	5.972615	-2.330354
132	1	0	3.028684	6.837552	-2.641717
133	1	0	4.515289	6.356167	-1.834277
134	1	0	3.937443	5.440985	-3.234269
135	6	0	1.428567	4.875009	-2.202420
136	1	0	1.023068	5.853365	-2.479864
137	1	0	1.591873	4.303954	-3.121158
138	1	0	0.655619	4.371472	-1.615899
139	6	0	-6.671086	-1.059967	-0.967276
140	6	0	-1.921030	-2.765447	-4.474710
141	1	0	-2.212296	-1.736507	-4.710208
142	1	0	-1.021155	-2.730725	-3.855069

143	1	0	-1.640158	-3.262854	-5.408658
144	6	0	-7.074337	-0.298992	-2.245866
145	1	0	-8.080199	0.121875	-2.132858
146	1	0	-6.380528	0.526984	-2.437818
147	1	0	-7.076974	-0.957264	-3.118465
148	6	0	-7.697198	-2.173956	-0.682653
149	1	0	-7.358765	-2.835160	0.122349
150	1	0	-8.640466	-1.718789	-0.361511
151	1	0	-7.910619	-2.775917	-1.565235
152	6	0	-6.755326	-0.077629	0.211402
153	1	0	-6.108348	0.793732	0.077250
154	1	0	-7.782225	0.291600	0.296121
155	1	0	-6.500621	-0.564222	1.157740
156	6	0	-2.075780	2.935182	5.574008
157	1	0	-2.365572	3.633151	6.361524
158	1	0	-2.140157	1.910034	5.952275
159	1	0	-1.042426	3.132475	5.275199
160	6	0	-4.297905	0.425069	4.552649
161	6	0	-3.639030	-0.632482	5.454442
162	1	0	-4.387275	-1.070053	6.124856
163	1	0	-3.191659	-1.445085	4.873731
164	1	0	-2.857381	-0.186146	6.075629
165	6	0	-4.967008	1.485558	5.445046
166	1	0	-5.783539	1.001456	5.992007
167	1	0	-4.292210	1.912616	6.187594
168	1	0	-5.397469	2.300466	4.857175
169	6	0	-5.449022	-0.236303	3.761220
170	1	0	-5.933961	0.495472	3.106856
171	1	0	-5.118575	-1.078137	3.146964
172	1	0	-6.202000	-0.618429	4.458588
173	6	0	-1.761212	4.257878	2.093657
174	6	0	-0.821812	4.845403	3.162370
175	1	0	-0.497539	5.844992	2.851636
176	1	0	-1.313342	4.948317	4.130294
177	1	0	0.073781	4.228136	3.285427
178	6	0	-3.088263	5.041727	2.088263
179	1	0	-3.624961	4.928650	3.032808
180	1	0	-2.888945	6.108351	1.934933
181	1	0	-3.739361	4.703302	1.275063
182	6	0	-1.087079	4.481610	0.729717
183	1	0	-0.111425	3.987074	0.675706
184	1	0	-1.706088	4.125613	-0.100217
185	1	0	-0.920449	5.553006	0.581866
186	8	0	-1.653702	0.454580	-2.585612
187	6	0	-1.863641	1.756311	-3.043407
188	6	0	-3.155053	2.302746	-2.502360
189	6	0	-4.281222	2.846407	-3.037975
190	8	0	-3.268825	2.262406	-1.148918
191	6	0	-5.145025	3.160498	-1.934334
192	1	0	-4.474698	3.014957	-4.087847
193	6	0	-4.478294	2.779140	-0.815930
194	1	0	-6.130096	3.602815	-1.977474
195	1	0	-4.707748	2.802047	0.238927
196	6	0	-1.848034	1.756866	-4.575182
197	1	0	-2.776915	1.357585	-4.986921
198	1	0	-1.007889	1.150892	-4.917504
199	8	0	-1.730850	3.093244	-5.088777
200	6	0	-0.492814	3.600461	-5.166842
201	8	0	0.509074	2.956376	-4.916180
202	1	0	-1.055384	2.438108	-2.717979
203	1	0	1.088814	0.501484	-2.795036
204	1	0	0.984176	1.249158	-2.629099
205	6	0	-0.509842	5.043557	-5.566453
206	1	0	0.493913	5.364001	-5.844066
207	1	0	-1.207344	5.208770	-6.390454
208	1	0	-0.855395	5.635601	-4.712665

TS2a(*S*) -1c

RwB97XD SCF energy -4903.675113 a.u.
RwB97XD SCF enthalpy -4901.778009 a.u.
RwB97XD SCF free energy -4902.015600 a.u.
Three lowest frequencies (cm^{-1}) -1046, 6.6, 8.6
Imaginary frequency (cm^{-1}) -1046
Cartesian coordinates:

Center Number	Atomic Number	Atomic Type	Coordinates (Angstroms) X	Y	Z
1	6	0	0.980307	-0.858541	2.490991
2	6	0	0.986353	-0.724569	3.862247
3	6	0	1.660963	0.298549	4.525071
4	6	0	2.356133	1.265517	3.844316
5	6	0	2.354928	1.164680	2.445786
6	6	0	1.694150	0.139603	1.771676
7	1	0	2.887351	2.059486	4.355091
8	1	0	2.904394	1.911582	1.885924
9	6	0	0.363714	-2.070625	1.890547
10	6	0	-0.706526	-2.114503	0.954333
11	6	0	0.877714	-3.292008	2.274052
12	6	0	-1.157597	-3.339699	0.463230
13	6	0	0.418354	-4.506622	1.776643
14	6	0	-0.599428	-4.564941	0.857169
15	1	0	-1.978673	-3.369353	-0.242085
16	1	0	-0.967013	-5.504738	0.462802
17	8	0	1.507199	0.144694	5.866683
18	8	0	0.385034	-1.531658	4.779727
19	8	0	1.903568	-3.521263	3.140921
20	8	0	1.139662	-5.521481	2.328425
21	6	0	0.972897	-1.171829	6.029239
22	1	0	0.211859	-1.164903	6.807539
23	1	0	1.788783	-1.867543	6.259007
24	6	0	1.848666	-4.920635	3.415439
25	1	0	1.293334	-5.085367	4.346550
26	1	0	2.859053	-5.324618	3.464594
27	15	0	-1.428124	-0.557349	0.312926
28	15	0	1.770069	0.091476	-0.047333
29	46	0	-0.105575	0.437658	-1.285933
30	6	0	-1.752463	0.486735	1.753959
31	6	0	-2.453007	-0.003371	2.852295
32	6	0	-1.283980	1.796304	1.763773
33	6	0	-2.660217	0.776413	3.991350
34	1	0	-2.830368	-1.020663	2.824307
35	6	0	-1.452745	2.630475	2.869142
36	1	0	-0.754718	2.163577	0.891027
37	6	0	-2.061705	2.057317	4.007370
38	6	0	-2.989055	-1.019686	-0.490318
39	6	0	-4.244161	-0.756609	0.054603
40	6	0	-2.898996	-1.654669	-1.725584
41	6	0	-5.408079	-1.203543	-0.571855
42	1	0	-4.309221	-0.203335	0.981673
43	6	0	-4.022576	-2.124843	-2.408016
44	1	0	-1.918042	-1.796326	-2.163993
45	6	0	-5.264477	-1.964579	-1.758142
46	6	0	2.964639	1.377782	-0.498902
47	6	0	4.327072	1.101523	-0.577559
48	6	0	2.503714	2.659068	-0.771991
49	6	0	5.252591	2.108258	-0.835532
50	1	0	4.666687	0.085632	-0.427134
51	6	0	3.375774	3.708608	-1.075487
52	1	0	1.433375	2.841171	-0.765440
53	6	0	4.755029	3.426696	-0.985839
54	6	0	2.480912	-1.508260	-0.505724
55	6	0	2.022294	-2.134338	-1.655098
56	6	0	3.479645	-2.115190	0.258655
57	6	0	2.590535	-3.319427	-2.130872
58	1	0	1.192782	-1.691964	-2.197534
59	6	0	4.118388	-3.269127	-0.184508
60	1	0	3.761127	-1.664136	1.202260
61	6	0	3.723658	-3.790086	-1.443645
62	6	0	1.877386	-3.991052	-3.326150
63	6	0	2.005068	-3.100159	-4.575368
64	1	0	1.490632	-3.566275	-5.423094
65	1	0	1.555790	-2.113970	-4.414878
66	1	0	3.052717	-2.951393	-4.855811
67	6	0	2.350821	-5.413005	-3.672523
68	1	0	2.350397	-6.067614	-2.796889
69	1	0	1.652129	-5.834466	-4.403670
70	1	0	3.342737	-5.440793	-4.125158
71	6	0	0.379896	-4.123589	-2.963520
72	1	0	0.245579	-4.762610	-2.084012
73	1	0	-0.095096	-3.160661	-2.756690

74	1	0	-0.156188	-4.579785	-3.801983
75	6	0	5.146868	-3.988571	0.710990
76	6	0	4.666510	-5.436099	0.938038
77	1	0	4.674753	-6.010720	0.009984
78	1	0	5.318970	-5.941008	1.658945
79	1	0	3.645143	-5.446656	1.331637
80	6	0	6.558711	-3.993635	0.096113
81	1	0	6.871191	-2.981187	-0.183282
82	1	0	7.276598	-4.372195	0.832386
83	1	0	6.623643	-4.634317	-0.783869
84	6	0	5.255241	-3.313201	2.088435
85	1	0	5.666651	-2.299947	2.019608
86	1	0	4.289090	-3.261340	2.599423
87	1	0	5.933809	-3.898234	2.717152
88	8	0	4.479946	-4.801956	-1.981097
89	8	0	5.673265	4.443612	-1.070770
90	8	0	-2.056376	2.761383	5.181840
91	8	0	-6.404265	-2.519163	-2.286941
92	6	0	-6.582200	-3.905172	-2.006697
93	1	0	-7.632611	-4.129593	-2.202747
94	1	0	-5.963326	-4.533309	-2.653322
95	1	0	-6.348352	-4.133916	-0.962133
96	6	0	-3.787548	-2.777538	-3.791231
97	6	0	-5.016642	-2.757524	-4.718431
98	1	0	-4.702070	-3.067997	-5.720660
99	1	0	-5.810653	-3.434234	-4.406399
100	1	0	-5.437643	-1.750381	-4.799932
101	6	0	-3.297116	-4.224252	-3.590490
102	1	0	-3.064743	-4.683160	-4.558120
103	1	0	-2.390075	-4.245256	-2.977818
104	1	0	-4.045268	-4.848510	-3.094643
105	6	0	5.355317	-4.346778	-3.010989
106	1	0	5.911409	-5.219893	-3.356503
107	1	0	4.803347	-3.912951	-3.850869
108	1	0	6.055384	-3.594748	-2.630386
109	6	0	5.812191	5.176910	0.144515
110	1	0	6.604887	5.908748	-0.020397
111	1	0	4.888277	5.699537	0.411005
112	1	0	6.090539	4.517116	0.973184
113	6	0	6.741987	1.754205	-1.027372
114	6	0	6.972438	0.235395	-0.923511
115	1	0	6.750609	-0.146888	0.078931
116	1	0	6.377332	-0.328203	-1.649881
117	1	0	8.026464	0.023324	-1.128070
118	6	0	7.180288	2.192537	-2.438582
119	1	0	7.049016	3.266743	-2.584810
120	1	0	8.239168	1.955545	-2.591468
121	1	0	6.602288	1.667166	-3.206912
122	6	0	7.645402	2.421470	0.026804
123	1	0	7.740809	3.495280	-0.135953
124	1	0	7.266666	2.251012	1.040184
125	1	0	8.650816	1.990307	-0.031089
126	6	0	2.728405	5.038573	-1.531578
127	6	0	2.035866	5.718943	-0.337275
128	1	0	1.260957	5.073514	0.086717
129	1	0	2.746112	5.963351	0.458760
130	1	0	1.556539	6.649705	-0.660763
131	6	0	3.688348	6.035835	-2.203640
132	1	0	3.091413	6.847646	-2.632875
133	1	0	4.398138	6.490025	-1.512060
134	1	0	4.249770	5.569232	-3.018140
135	6	0	1.651511	4.708787	-2.592804
136	1	0	1.220397	5.637776	-2.978884
137	1	0	2.083844	4.161291	-3.436879
138	1	0	0.824318	4.117009	-2.190490
139	6	0	-6.794316	-0.819810	-0.007142
140	6	0	-2.681811	-2.006448	-4.547806
141	1	0	-2.932170	-0.946206	-4.652350
142	1	0	-1.701368	-2.080879	-4.070835
143	1	0	-2.573863	-2.431180	-5.551070
144	6	0	-7.589722	-0.056399	-1.084492
145	1	0	-8.557879	0.257038	-0.677470
146	1	0	-7.050991	0.841422	-1.402433
147	1	0	-7.773994	-0.675352	-1.964160
148	6	0	-7.601324	-2.047149	0.459854
149	1	0	-7.001468	-2.691749	1.111368
150	1	0	-8.470841	-1.710422	1.035178
151	1	0	-7.975472	-2.642265	-0.372601
152	6	0	-6.661830	0.104458	1.214007
153	1	0	-6.095034	1.013250	0.992264
154	1	0	-7.661683	0.409591	1.538775
155	1	0	-6.186537	-0.405775	2.056587
156	6	0	-0.909213	2.476937	5.974417
157	1	0	-1.025268	3.028861	6.909076
158	1	0	-0.832762	1.404621	6.188614
159	1	0	0.009151	2.803626	5.471133
160	6	0	-3.524295	0.161631	5.119011
161	6	0	-2.759222	-0.996081	5.781986
162	1	0	-3.396220	-1.486562	6.526551
163	1	0	-2.445020	-1.749922	5.054023
164	1	0	-1.869036	-0.622511	6.296052
165	6	0	-3.974213	1.133708	6.224012
166	1	0	-4.706125	0.614948	6.853256
167	1	0	-3.155751	1.451616	6.871036
168	1	0	-4.457353	2.025012	5.814860
169	6	0	-4.821464	-0.386946	4.485993
170	1	0	-5.385542	0.419704	4.005990
171	1	0	-4.638611	-1.166674	3.741945
172	1	0	-5.455364	-0.824823	5.264148
173	6	0	-0.950086	4.089882	2.739539
174	6	0	0.586938	4.101962	2.717350
175	1	0	0.952013	5.128593	2.601300
176	1	0	0.998928	3.698803	3.647113
177	1	0	0.981145	3.507689	1.886729
178	6	0	-1.426762	5.052918	3.840135
179	1	0	-0.954035	4.862272	4.804238
180	1	0	-1.160513	6.072800	3.540312
181	1	0	-2.511293	5.016967	3.975138
182	6	0	-1.467277	4.675910	1.407073
183	1	0	-1.143723	4.108035	0.531147
184	1	0	-2.561498	4.719002	1.395189
185	1	0	-1.088907	5.696962	1.289144
186	8	0	-1.430234	0.831616	-2.948453
187	6	0	-1.981088	2.126564	-3.041691
188	6	0	-3.146782	2.321260	-2.125218
189	6	0	-4.485361	2.482345	-2.303836
190	8	0	-2.847031	2.425070	-0.801136
191	6	0	-5.043876	2.705499	-1.002850
192	1	0	-5.012712	2.469046	-3.246969
193	6	0	-4.006567	2.647219	-0.131447
194	1	0	-6.080101	2.884787	-0.755413
195	1	0	-3.925055	2.736943	0.941115
196	6	0	-2.372431	2.341024	-4.504631
197	1	0	-3.214291	1.703212	-4.780707
198	1	0	-1.516608	2.106984	-5.140073
199	8	0	-2.810402	3.684973	-4.742486
200	6	0	-1.860356	4.612440	-4.936628
201	8	0	-0.672970	4.355305	-4.959246
202	1	0	-1.222867	2.882188	-2.777181
203	1	0	0.823042	1.101605	-2.637196
204	1	0	-0.029999	1.024997	-2.991831
205	6	0	-2.457356	5.975024	-5.113190
206	1	0	-1.677770	6.690499	-5.372810
207	1	0	-3.223134	5.952427	-5.892609
208	1	0	-2.940891	6.279823	-4.180230

6c(*S*)•2a

RwB97XD SCF energy -4903.694458 a.u.
RwB97XD SCF enthalpy -4901.815530 a.u.
RwB97XD SCF free energy -4902.052870 a.u.
Three lowest frequencies (cm^{-1}) 10.8, 18.4, 20.8
Cartesian coordinates:

Center Atomic Atomic Coordinates (Angstroms)

Number	Number	Type	X	Y	Z
1	6	0	-0.897433	0.645784	2.417459
2	6	0	-0.883177	0.401263	3.774450
3	6	0	-1.614257	-0.616581	4.379575
4	6	0	-2.381438	-1.482771	3.643684
5	6	0	-2.393777	-1.278538	2.256602
6	6	0	-1.687463	-0.246512	1.638301
7	1	0	-2.956301	-2.276857	4.104752
8	1	0	-2.998837	-1.949276	1.659540
9	6	0	-0.223111	1.882564	1.932931
10	6	0	0.888038	1.972673	1.050472
11	6	0	-0.694682	3.079699	2.432834
12	6	0	1.409632	3.221019	0.711934
13	6	0	-0.164715	4.316887	2.087521
14	6	0	0.889715	4.423966	1.215130
15	1	0	2.260442	3.283637	0.044267
16	1	0	1.314726	5.382066	0.940041
17	8	0	-1.435100	-0.565211	5.727143
18	8	0	-0.209121	1.097278	4.734572
19	8	0	-1.729996	3.262897	3.303045
20	8	0	-0.858109	5.300070	2.734130
21	6	0	-0.806910	0.697013	5.965456
22	1	0	-0.041762	0.586160	6.732228
23	1	0	-1.568816	1.431258	6.253829
24	6	0	-1.591466	4.610952	3.748112
25	1	0	-1.018949	4.626036	4.684072
26	1	0	-2.575457	5.062183	3.865791
27	15	0	1.552591	0.438575	0.281807
28	15	0	-1.821028	-0.058330	-0.180533
29	46	0	0.044339	-0.353392	-1.393855
30	6	0	1.871182	-0.673874	1.679623
31	6	0	2.593433	-0.265312	2.796300
32	6	0	1.351819	-1.963549	1.636016
33	6	0	2.773847	-1.102418	3.898814
34	1	0	3.011338	0.736571	2.811604
35	6	0	1.491605	-2.855432	2.701050
36	1	0	0.805190	-2.269143	0.750179
37	6	0	2.130338	-2.360691	3.859361
38	6	0	3.144668	0.929948	-0.454209
39	6	0	4.393208	0.569644	0.046443
40	6	0	3.076003	1.660739	-1.636317
41	6	0	5.571154	1.007018	-0.562807
42	1	0	4.442871	-0.059239	0.925301
43	6	0	4.211288	2.124692	-2.303193
44	1	0	2.098345	1.880496	-2.050915
45	6	0	5.451844	1.862754	-1.684971
46	6	0	-3.146036	-1.220261	-0.626725
47	6	0	-4.487812	-0.851785	-0.607112
48	6	0	-2.798156	-2.518098	-0.972698
49	6	0	-5.496924	-1.780555	-0.848546
50	1	0	-4.743940	0.177266	-0.398333
51	6	0	-3.757249	-3.498799	-1.241518
52	1	0	-1.744789	-2.770353	-1.040371
53	6	0	-5.106260	-3.125053	-1.067881
54	6	0	-2.456215	1.618104	-0.453553
55	6	0	-1.965843	2.355357	-1.520464
56	6	0	-3.413049	2.186005	0.389808
57	6	0	-2.455072	3.626865	-1.834567
58	1	0	-1.171127	1.928056	-2.123285
59	6	0	-3.984767	3.420711	0.099423
60	1	0	-3.719683	1.642225	1.274971
61	6	0	-3.554473	4.077834	-1.081791
62	6	0	-1.704938	4.397571	-2.944909
63	6	0	-1.960486	3.730181	-4.308121
64	1	0	-1.403016	4.252990	-5.093529
65	1	0	-1.637735	2.683238	-4.307464
66	1	0	-3.021626	3.754206	-4.575471
67	6	0	-2.038708	5.896323	-3.041217
68	1	0	-1.938544	6.395369	-2.073161
69	1	0	-1.326772	6.361661	-3.731768
70	1	0	-3.038585	6.092626	-3.429101
71	6	0	-0.190051	4.324053	-2.638680
72	1	0	0.035907	4.780593	-1.668969
73	1	0	0.196778	3.300883	-2.627923
74	1	0	0.361938	4.870446	-3.410573
75	6	0	-4.985766	4.066021	1.079461
76	6	0	-4.457366	5.456397	1.485937
77	1	0	-4.470215	6.154312	0.646878
78	1	0	-5.075649	5.874469	2.288017
79	1	0	-3.426755	5.387097	1.847238
80	6	0	-6.393675	4.200810	0.470460
81	1	0	-6.747232	3.243604	0.070300
82	1	0	-7.099168	4.517630	1.246817
83	1	0	-6.427524	4.946377	-0.324384
84	6	0	-5.123219	3.230514	2.363531
85	1	0	-5.568760	2.247751	2.173815
86	1	0	-4.161462	3.085335	2.864829
87	1	0	-5.784978	3.757342	3.058047
88	8	0	-4.240474	5.202848	-1.468463
89	8	0	-6.097856	-4.074288	-1.132229
90	8	0	2.107209	-3.118411	5.001621
91	8	0	6.609838	2.409630	-2.185714
92	6	0	6.832700	3.765973	-1.809785
93	1	0	7.884823	3.977367	-2.011770
94	1	0	6.217673	4.454892	-2.395878
95	1	0	6.625323	3.924181	-0.746590
96	6	0	3.987563	2.865109	-3.643839
97	6	0	5.212827	2.873525	-4.576394
98	1	0	4.899804	3.242240	-5.559182
99	1	0	6.017504	3.521887	-4.232184
100	1	0	5.619282	1.866437	-4.712237
101	6	0	3.531151	4.307992	-3.357949
102	1	0	3.323879	4.833144	-4.297431
103	1	0	2.616011	4.313444	-2.756861
104	1	0	4.289042	4.878301	-2.813857
105	6	0	-5.152998	4.961695	-2.537052
106	1	0	-5.708358	5.889367	-2.686578
107	1	0	-4.633557	4.698186	-3.463827
108	1	0	-5.849022	4.153824	-2.286425
109	6	0	-6.237420	-4.816388	0.077618
110	1	0	-7.073510	-5.502121	-0.070309
111	1	0	-5.334220	-5.390775	0.307019
112	1	0	-6.451308	-4.154657	0.923723
113	6	0	-6.962840	-1.307918	-0.958140
114	6	0	-7.075237	0.215512	-0.762910
115	1	0	-6.783527	0.521184	0.248136
116	1	0	-6.471263	0.776778	-1.483460
117	1	0	-8.118107	0.514506	-0.907200
118	6	0	-7.477693	-1.633853	-2.374059
119	1	0	-7.448547	-2.707450	-2.573607
120	1	0	-8.514215	-1.295089	-2.483415
121	1	0	-6.875148	-1.124744	-3.134286
122	6	0	-7.882488	-1.961313	0.090342
123	1	0	-8.057213	-3.017278	-0.116041
124	1	0	-7.466786	-1.862142	1.098854
125	1	0	-8.856311	-1.459664	0.081762
126	6	0	-3.222857	-4.864722	-1.738052
127	6	0	-2.504167	-5.583282	-0.580698
128	1	0	-1.661510	-4.991048	-0.210128
129	1	0	-3.180364	-5.772146	0.259337
130	1	0	-2.110163	-6.547317	-0.921953
131	6	0	-4.277963	-5.805986	-2.345067
132	1	0	-3.757512	-6.653316	-2.804697
133	1	0	-4.969274	-6.215856	-1.608671
134	1	0	-4.860277	-5.310008	-3.126706
135	6	0	-2.194638	-4.602088	-2.864124
136	1	0	-1.821899	-5.556495	-3.250454
137	1	0	-2.653166	-4.057475	-3.696158
138	1	0	-1.325486	-4.033376	-2.524039
139	6	0	6.943315	0.510062	-0.054743
140	6	0	2.862402	2.161885	-4.438684
141	1	0	3.094389	1.105254	-4.606783
142	1	0	1.887166	2.227407	-3.948842
143	1	0	2.755837	2.646400	-5.414547
144	6	0	7.682364	-0.200773	-1.205978
145	1	0	8.637982	-0.598647	-0.845653
146	1	0	7.090226	-1.039091	-1.586165
147	1	0	7.886545	0.480149	-2.034177
148	6	0	7.815673	1.655933	0.493343
149	1	0	7.264943	2.258768	1.223664
150	1	0	8.691489	1.236190	1.000851
151	1	0	8.181408	2.312744	-0.295552
152	6	0	6.781436	-0.506301	1.086948
153	1	0	6.167685	-1.365033	0.798650
154	1	0	7.768835	-0.885565	1.368426
155	1	0	6.341820	-0.048079	1.977300
156	6	0	0.976715	-2.825167	5.813462
157	1	0	1.083005	-3.407805	6.730658
158	1	0	0.934778	-1.757650	6.060074
159	1	0	0.044242	-3.107232	5.308872
160	6	0	3.671390	-0.567376	5.041529
161	6	0	2.979032	0.622372	5.728079
162	1	0	3.648248	1.061719	6.476521

163	1	0	2.705398	1.406541	5.016047
164	1	0	2.071745	0.293746	6.242521
165	6	0	4.059822	-1.583326	6.129079
166	1	0	4.806484	-1.113748	6.779646
167	1	0	3.218468	-1.875200	6.758961
168	1	0	4.506547	-2.487480	5.707452
169	6	0	4.998871	-0.086755	4.415813
170	1	0	5.512241	-0.917543	3.919932
171	1	0	4.860092	0.713965	3.684352
172	1	0	5.660620	0.299342	5.198387
173	6	0	0.931662	-4.286873	2.504363
174	6	0	-0.604420	-4.236339	2.461679
175	1	0	-1.008310	-5.240216	2.287347
176	1	0	-1.013306	-3.867074	3.406902
177	1	0	-0.965397	-3.582406	1.661549
178	6	0	1.350983	-5.316260	3.567950
179	1	0	0.881122	-5.141831	4.536645
180	1	0	1.034553	-6.307829	3.224572
181	1	0	2.434766	-5.342332	3.710142
182	6	0	1.442939	-4.836663	1.154084
183	1	0	1.148923	-4.220763	0.300582
184	1	0	2.534884	-4.920139	1.151015
185	1	0	1.029781	-5.837908	0.991085
186	8	0	1.520402	-0.643872	-3.026511
187	6	0	1.896656	-2.021537	-3.252007
188	6	0	3.056084	-2.344648	-2.381211
189	6	0	4.381749	-2.544720	-2.607412
190	8	0	2.781006	-2.518362	-1.059803
191	6	0	4.960392	-2.866305	-1.338511
192	1	0	4.888899	-2.486395	-3.559739
193	6	0	3.946282	-2.822638	-0.438094
194	1	0	5.994986	-3.095201	-1.129449
195	1	0	3.888809	-2.975078	0.628802
196	6	0	2.211487	-2.219676	-4.733463
197	1	0	3.158924	-1.754467	-5.007740
198	1	0	1.410646	-1.795402	-5.345029
199	8	0	2.350969	-3.611947	-5.015703
200	6	0	1.212514	-4.306111	-5.208460
201	8	0	0.112855	-3.792564	-5.174602
202	1	0	1.046833	-2.656622	-2.978458
203	1	0	-1.038588	-0.752626	-2.457904
204	1	0	1.000630	-0.336449	-3.782824
205	6	0	1.493021	-5.755442	-5.452509
206	1	0	0.569075	-6.271603	-5.711002
207	1	0	2.227110	-5.867555	-6.254223
208	1	0	1.919495	-6.195601	-4.546053

2.7. Catalytic cycle 2a–1c (*R*)

3a(*R*) -1c

RwB97XD SCF energy -4902.509297 a.u.

RwB97XD SCF enthalpy -4900.630657 a.u.

RwB97XD SCF free energy -4900.867181 a.u.

Three lowest frequencies (cm^{-1}) 8.5, 15.2, 19.2

Cartesian coordinates:

Center Number	Atomic Number	Atomic Type	Coordinates X	(Angstroms) Y	Z
1	6	0	-0.774756	-0.145635	2.565142
2	6	0	-0.359745	-0.791056	3.709851
3	6	0	-0.658301	-2.118127	3.996195
4	6	0	-1.444615	-2.871741	3.159925
5	6	0	-1.903368	-2.236966	1.996936
6	6	0	-1.586864	-0.913404	1.685022
7	1	0	-1.693756	-3.903234	3.378214
8	1	0	-2.516029	-2.816869	1.315868
9	6	0	-0.324519	1.262543	2.389059
10	6	0	0.655576	1.686843	1.452997
11	6	0	-0.785135	2.225521	3.257988
12	6	0	1.068934	3.015991	1.415944
13	6	0	-0.357619	3.547872	3.220971
14	6	0	0.562663	3.983027	2.299458
15	1	0	1.813256	3.325497	0.691129
16	1	0	0.895146	5.013799	2.263122
17	8	0	-0.093658	-2.463658	5.184413
18	8	0	0.381064	-0.262291	4.722705
19	8	0	-1.705460	2.072534	4.252014
20	8	0	-0.997026	4.256111	4.195148
21	6	0	0.776514	-1.378052	5.518613
22	1	0	1.803174	-1.646690	5.256701
23	1	0	0.667929	-1.135534	6.575402
24	6	0	-1.642075	3.278495	5.015125
25	1	0	-1.042040	3.107527	5.916385
26	1	0	-2.652574	3.610491	5.254078
27	15	0	1.320156	0.452909	0.283039
28	15	0	-2.033209	-0.311773	0.012438
29	46	0	-0.215003	-0.638711	-1.248750
30	6	0	2.165588	-0.774640	1.338582
31	6	0	2.985629	-0.432210	2.415841
32	6	0	2.010512	-2.112657	1.007583
33	6	0	3.643874	-1.403068	3.170538
34	1	0	3.108226	0.615313	2.661674
35	6	0	2.629202	-3.142944	1.724229
36	1	0	1.380476	-2.365281	0.162493
37	6	0	3.382780	-2.762628	2.851955
38	6	0	2.598408	1.332061	-0.675096
39	6	0	3.855189	1.652510	-0.160554
40	6	0	2.314564	1.642803	-1.998829
41	6	0	4.797550	2.345688	-0.914731
42	1	0	4.094699	1.358730	0.851904
43	6	0	3.229877	2.299071	-2.830567
44	1	0	1.353429	1.348794	-2.408086
45	6	0	4.426430	2.731024	-2.228053
46	6	0	-3.537944	-1.243323	-0.445448
47	6	0	-4.809354	-0.751833	-0.166113
48	6	0	-3.415045	-2.457801	-1.116698
49	6	0	-5.961640	-1.484603	-0.455901
50	1	0	-4.908766	0.230553	0.269418
51	6	0	-4.523054	-3.242763	-1.433799
52	1	0	-2.426112	-2.784632	-1.408624
53	6	0	-5.779373	-2.783211	-0.978486
54	6	0	-2.524396	1.428681	0.128663
55	6	0	-2.031566	2.316481	-0.815478
56	6	0	-3.405633	1.889642	1.111079
57	6	0	-2.466838	3.645384	-0.882552
58	1	0	-1.279893	1.961644	-1.514991
59	6	0	-3.926635	3.178899	1.061560
60	1	0	-3.692639	1.215737	1.909361
61	6	0	-3.515386	4.003795	-0.017074
62	6	0	-1.716030	4.573953	-1.866339
63	6	0	-1.949604	4.102687	-3.313213
64	1	0	-1.379235	4.729713	-4.007712
65	1	0	-1.630206	3.065755	-3.461510
66	1	0	-3.007765	4.171052	-3.587287

67	6	0	-2.060009	6.070447	-1.770733
68	1	0	-1.965966	6.444830	-0.747997
69	1	0	-1.348839	6.622619	-2.395007
70	1	0	-3.059170	6.310103	-2.135917
71	6	0	-0.210408	4.473363	-1.533772
72	1	0	-0.020828	4.827071	-0.515613
73	1	0	0.180274	3.456168	-1.616700
74	1	0	0.364820	5.100687	-2.222730
75	6	0	-4.851891	3.697826	2.181963
76	6	0	-4.200631	4.937731	2.824741
77	1	0	-4.109331	5.756830	2.108172
78	1	0	-4.809556	5.287331	3.666199
79	1	0	-3.200281	4.704513	3.201167
80	6	0	-6.257731	4.064968	1.669553
81	1	0	-6.694508	3.249701	1.083656
82	1	0	-6.917623	4.250348	2.524400
83	1	0	-6.251853	4.970028	1.061461
84	6	0	-5.036612	2.640436	3.282702
85	1	0	-5.563168	1.752926	2.914157
86	1	0	-4.083883	2.321307	3.715125
87	1	0	-5.641414	3.068483	4.088204
88	8	0	-4.174983	5.195155	-0.196931
89	8	0	-6.873513	-3.609460	-1.100103
90	8	0	3.927011	-3.729831	3.662874
91	8	0	5.298543	3.531844	-2.924919
92	6	0	4.904173	4.901111	-2.950906
93	1	0	5.681021	5.440743	-3.495669
94	1	0	3.945154	5.037442	-3.459968
95	1	0	4.818397	5.308537	-1.937791
96	6	0	2.851546	2.418495	-4.328527
97	6	0	4.008141	2.795260	-5.271721
98	1	0	3.662615	2.663089	-6.302858
99	1	0	4.329789	3.832022	-5.172689
100	1	0	4.877649	2.148718	-5.124688
101	6	0	1.701312	3.425910	-4.504043
102	1	0	1.412612	3.484632	-5.559649
103	1	0	0.819201	3.121259	-3.932792
104	1	0	1.985143	4.431300	-4.177635
105	6	0	-5.130582	5.133282	-1.254171
106	1	0	-5.618198	6.108742	-1.295684
107	1	0	-4.654219	4.924756	-2.217614
108	1	0	-5.880635	4.358053	-1.062755
109	6	0	-7.073974	-4.423823	0.058975
110	1	0	-7.974012	-5.012459	-0.127031
111	1	0	-6.227926	-5.095100	0.227031
112	1	0	-7.212948	-3.815261	0.955917
113	6	0	-7.347246	-0.805767	-0.352977
114	6	0	-7.215682	0.656233	0.111061
115	1	0	-6.815550	0.730823	1.128324
116	1	0	-6.581394	1.251199	-0.554515
117	1	0	-8.208232	1.117001	0.114444
118	6	0	-7.971000	-0.788102	-1.763405
119	1	0	-8.120193	-1.799576	-2.148802
120	1	0	-8.945512	-0.287897	-1.733917
121	1	0	-7.331708	-0.241391	-2.465044
122	6	0	-8.317470	-1.496565	0.622385
123	1	0	-8.679267	-2.450811	0.237470
124	1	0	-7.855299	-1.659557	1.601735
125	1	0	-9.192933	-0.855417	0.772190
126	6	0	-4.364800	-4.438281	-2.402020
127	6	0	-2.900284	-4.609829	-2.844118
128	1	0	-2.501629	-3.711821	-3.327986
129	1	0	-2.242589	-4.875038	-2.008600
130	1	0	-2.846524	-5.422419	-3.575229
131	6	0	-4.820300	-5.789210	-1.822480
132	1	0	-4.514860	-6.592724	-2.501355
133	1	0	-4.359183	-5.984888	-0.848565
134	1	0	-5.904235	-5.847725	-1.718775
135	6	0	-5.192999	-4.122343	-3.664524
136	1	0	-5.093302	-4.934924	-4.393105
137	1	0	-6.253925	-4.007481	-3.429414
138	1	0	-4.842613	-3.198777	-4.138130
139	6	0	6.222778	2.562822	-0.362302
140	6	0	2.356388	1.034698	-4.808957
141	1	0	3.128663	0.271447	-4.660985
142	1	0	1.446042	0.710148	-4.300103
143	1	0	2.128582	1.077217	-5.879315
144	6	0	7.202299	1.768040	-1.248834
145	1	0	8.224407	1.873387	-0.867503
146	1	0	6.949419	0.701664	-1.248718
147	1	0	7.185685	2.125260	-2.281561
148	6	0	6.643152	4.044542	-0.323311
149	1	0	5.891788	4.657456	0.186199
150	1	0	7.581440	4.139084	0.234350
151	1	0	6.812833	4.453530	-1.318884
152	6	0	6.355249	2.030640	1.074607
153	1	0	6.135230	0.961519	1.149087
154	1	0	7.386021	2.172811	1.413606
155	1	0	5.702801	2.570005	1.770289
156	6	0	3.011461	-4.268709	4.613986
157	1	0	3.480758	-5.160864	5.033155
158	1	0	2.816137	-3.558900	5.423715
159	1	0	2.060194	-4.540875	4.146995
160	6	0	4.744626	-0.976121	4.172081
161	6	0	4.649093	-1.608273	5.572604
162	1	0	5.412413	-1.156950	6.215680
163	1	0	3.679908	-1.420585	6.043902
164	1	0	4.831351	-2.681839	5.553852
165	6	0	6.096506	-1.378902	3.547376
166	1	0	6.920085	-1.082215	4.206977
167	1	0	6.158325	-2.460344	3.394306
168	1	0	6.243864	-0.884693	2.581305
169	6	0	4.750458	0.548119	4.375422
170	1	0	4.957988	1.092653	3.449739
171	1	0	3.799582	0.907340	4.785190
172	1	0	5.539279	0.810869	5.086915
173	6	0	2.421972	-4.574887	1.169167
174	6	0	0.953935	-4.986332	1.388447
175	1	0	0.778995	-5.986792	0.976484
176	1	0	0.703023	-5.009949	2.453868
177	1	0	0.264847	-4.292952	0.894314
178	6	0	3.338482	-5.662525	1.754913
179	1	0	3.102439	-5.916518	2.787855
180	1	0	3.204899	-6.575145	1.163718
181	1	0	4.393475	-5.381015	1.698434
182	6	0	2.714118	-4.570215	-0.349837
183	1	0	2.095020	-3.866590	-0.910524
184	1	0	3.765992	-4.335633	-0.542560
185	1	0	2.519715	-5.568290	-0.756678
186	8	0	1.547669	-1.362548	-2.273271
187	6	0	2.084343	-1.950626	-3.210966
188	1	0	-1.216669	-1.302062	-2.234787
189	6	0	1.393512	-2.287778	-4.427889
190	6	0	0.127421	-2.023594	-4.886132
191	8	0	2.098369	-2.969138	-5.380057
192	6	0	0.053412	-2.580893	-6.187127
193	1	0	-0.642997	-1.487223	-4.350553
194	6	0	1.276241	-3.138361	-6.427873
195	1	0	-0.789851	-2.569525	-6.861636
196	1	0	1.688751	-3.668650	-7.273433
197	6	0	3.542780	-2.329367	-3.094330
198	1	0	4.105049	-1.923924	-3.941960
199	1	0	3.639678	-3.420916	-3.114335
200	8	0	4.017840	-1.796656	-1.870842
201	6	0	5.219117	-2.227207	-1.453810
202	8	0	5.908522	-2.981347	-2.108974
203	6	0	5.546697	-1.704390	-0.090603
204	1	0	5.159933	-2.418243	0.645139
205	1	0	6.628028	-1.634067	0.028730
206	1	0	5.068827	-0.740829	0.087029

TS1a(*R*) -1c

RwB97XD SCF energy -4902.495479 a.u.
RwB97XD SCF enthalpy -4900.618618 a.u.
RwB97XD SCF free energy -4900.851259 a.u.
Three lowest frequencies (cm^{-1}) -646.1, 16.0, 20.1
Imaginary frequency (cm^{-1}) -646.1
Cartesian coordinates:

Center Number	Atomic Number	Atomic Type	Coordinates (Angstroms) X	Y	Z
1	6	0	0.474387	1.137245	2.360877
2	6	0	0.303241	2.186101	3.239483
3	6	0	0.924958	3.421219	3.092175
4	6	0	1.740328	3.689286	2.021050
5	6	0	1.930473	2.643251	1.106467
6	6	0	1.331090	1.391633	1.254088
7	1	0	2.226542	4.648838	1.892191
8	1	0	2.582956	2.829042	0.262100
9	6	0	-0.198841	-0.144501	2.703393

10	6	0	-1.226762	-0.785680	1.960620
11	6	0	0.126065	-0.732758	3.908044
12	6	0	-1.816803	-1.955391	2.439515
13	6	0	-0.473308	-1.895001	4.378849
14	6	0	-1.446606	-2.542959	3.658652
15	1	0	-2.609024	-2.431256	1.874046
16	1	0	-1.922594	-3.447348	4.018492
17	8	0	0.573807	4.227931	4.132804
18	8	0	-0.476242	2.209835	4.360089
19	8	0	1.053519	-0.307975	4.812417
20	8	0	0.062141	-2.218793	5.590576
21	6	0	-0.006988	3.339055	5.091979
22	1	0	-0.841028	3.827015	5.592261
23	1	0	0.766461	3.018731	5.800768
24	6	0	0.771238	-1.046643	5.999985
25	1	0	0.129710	-0.444766	6.655347
26	1	0	1.703658	-1.330298	6.486423
27	15	0	-1.689643	-0.121522	0.320949
28	15	0	1.631240	0.122840	-0.033010
29	46	0	-0.111907	-0.518940	-1.346783
30	6	0	-1.957432	1.659504	0.528524
31	6	0	-2.724826	2.160915	1.580060
32	6	0	-1.412072	2.534109	-0.400642
33	6	0	-2.898139	3.529403	1.755874
34	1	0	-3.183637	1.459726	2.266168
35	6	0	-1.545764	3.922848	-0.285228
36	1	0	-0.848441	2.122193	-1.232443
37	6	0	-2.211745	4.392842	0.862245
38	6	0	-3.288180	-0.860340	-0.114225
39	6	0	-4.459531	-0.109693	-0.169599
40	6	0	-3.318919	-2.200089	-0.482690
41	6	0	-5.682166	-0.697393	-0.483230
42	1	0	-4.411967	0.950580	0.034383
43	6	0	-4.510254	-2.854052	-0.809130
44	1	0	-2.385730	-2.752792	-0.508523
45	6	0	-5.695587	-2.097013	-0.700735
46	6	0	3.069086	0.757729	-0.937755
47	6	0	4.359683	0.525661	-0.475091
48	6	0	2.873208	1.526293	-2.081288
49	6	0	5.467158	1.127046	-1.070613
50	1	0	4.503538	-0.146700	0.358532
51	6	0	3.933940	2.191692	-2.700326
52	1	0	1.869920	1.608244	-2.483148
53	6	0	5.204924	2.066528	-2.093761
54	6	0	2.165476	-1.377089	0.839880
55	6	0	1.731828	-2.602130	0.355256
56	6	0	2.964431	-1.336678	1.983552
57	6	0	2.097713	-3.813457	0.947150
58	1	0	1.067991	-2.614890	-0.502979
59	6	0	3.432989	-2.507512	2.573558
60	1	0	3.224017	-0.373193	2.404876
61	6	0	3.047992	-3.739563	1.983466
62	6	0	1.385600	-5.083091	0.424144
63	6	0	1.976576	-5.495001	-0.935404
64	1	0	1.402814	-6.326592	-1.360044
65	1	0	1.940468	-4.663168	-1.645225
66	1	0	3.018479	-5.816609	-0.850145
67	6	0	1.421791	-6.274932	1.396401
68	1	0	1.074129	-5.986227	2.393392
69	1	0	0.746948	-7.051133	1.019877
70	1	0	2.409901	-6.723714	1.496658
71	6	0	-0.114247	-4.755252	0.212777
72	1	0	-0.567091	-4.370456	1.133446
73	1	0	-0.285082	-4.023270	-0.582262
74	1	0	-0.646280	-5.667754	-0.075152
75	6	0	4.270068	-2.446161	3.868175
76	6	0	3.553958	-3.257887	4.966049
77	1	0	3.541796	-4.324377	4.734729
78	1	0	4.062696	-3.122442	5.927027
79	1	0	2.516710	-2.927972	5.077074
80	6	0	5.697151	-2.987456	3.661601
81	1	0	6.184918	-2.501614	2.808933
82	1	0	6.298852	-2.777963	4.553189
83	1	0	5.712659	-4.065876	3.503845
84	6	0	4.408715	-1.000721	4.375619
85	1	0	4.979070	-0.373221	3.681605
86	1	0	3.437678	-0.529892	4.553526
87	1	0	4.952806	-1.010646	5.325229
88	8	0	3.615905	-4.883398	2.487623
89	8	0	6.237132	2.855095	-2.547624
90	8	0	-2.234553	5.737666	1.137426
91	8	0	-6.919659	-2.707755	-0.831200
92	6	0	-7.349959	-3.366537	0.357546
93	1	0	-8.355435	-3.742813	0.160778
94	1	0	-6.697313	-4.205898	0.616242
95	1	0	-7.378519	-2.674436	1.205332
96	6	0	-4.385431	-4.325466	-1.273268
97	6	0	-5.626306	-4.898161	-1.980343
98	1	0	-5.358863	-5.869906	-2.409305
99	1	0	-6.469459	-5.064885	-1.310355
100	1	0	-5.958245	-4.252276	-2.798292
101	6	0	-4.025727	-5.209249	-0.064332
102	1	0	-3.867628	-6.244869	-0.386061
103	1	0	-3.104757	-4.862147	0.416444
104	1	0	-4.817561	-5.208407	0.691035
105	6	0	4.648627	-5.426371	1.668455
106	1	0	5.147691	-6.192927	2.264211
107	1	0	4.247845	-5.886668	0.760972
108	1	0	5.372304	-4.656181	1.381734
109	6	0	6.353714	4.077788	-1.813029
110	1	0	7.181727	4.629865	-2.260794
111	1	0	5.437865	4.670296	-1.878942
112	1	0	6.566299	3.890490	-0.757544
113	6	0	6.889599	0.634813	-0.715148
114	6	0	6.841526	-0.482063	0.343991
115	1	0	6.450439	-0.121648	1.302198
116	1	0	6.240269	-1.338983	0.022375
117	1	0	7.858387	-0.846110	0.520334
118	6	0	7.500838	0.035201	-1.998125
119	1	0	7.593270	0.787586	-2.785568
120	1	0	8.500507	-0.361007	-1.787710
121	1	0	6.883634	-0.786584	-2.376949
122	6	0	7.827390	1.726551	-0.170589
123	1	0	8.112130	2.445563	-0.939154
124	1	0	7.373868	2.262893	0.669685
125	1	0	8.748112	1.258658	0.194365
126	6	0	3.715486	2.907739	-4.052585
127	6	0	2.277139	2.703441	-4.556375
128	1	0	2.042251	1.641271	-4.673221
129	1	0	1.531098	3.158996	-3.896197
130	1	0	2.173319	3.172088	-5.539653
131	6	0	3.963046	4.426120	-3.992493
132	1	0	3.584961	4.890165	-4.909786
133	1	0	3.440162	4.886156	-3.146930
134	1	0	5.024888	4.666418	-3.924575
135	6	0	4.656122	2.277792	-5.099279
136	1	0	4.497105	2.751887	-6.074398
137	1	0	5.705843	2.407120	-4.828254
138	1	0	4.456595	1.206154	-5.209753
139	6	0	-6.936783	0.186315	-0.650929
140	6	0	-3.231126	-4.419999	-2.298710
141	1	0	-3.390505	-3.739824	-3.141932
142	1	0	-2.259508	-4.192569	-1.854527
143	1	0	-3.173234	-5.441184	-2.689575
144	6	0	-7.488183	0.006563	-2.078756
145	1	0	-8.383123	0.624523	-2.213280
146	1	0	-6.748189	0.318574	-2.823855
147	1	0	-7.758935	-1.032585	-2.276818
148	6	0	-8.038029	-0.146308	0.373275
149	1	0	-7.637722	-0.172063	1.392587
150	1	0	-8.812032	0.628186	0.339288
151	1	0	-8.522299	-1.100330	0.162703
152	6	0	-6.596819	1.677052	-0.472669
153	1	0	-5.827110	2.017259	-1.173750
154	1	0	-7.497545	2.269487	-0.661223
155	1	0	-6.263463	1.902978	0.546532
156	6	0	-1.040566	6.200124	1.766504
157	1	0	-1.172502	7.270638	1.933987
158	1	0	-0.877417	5.698080	2.724673
159	1	0	-0.162297	6.039340	1.133602
160	6	0	-3.892763	4.055557	2.810885
161	6	0	-3.254510	5.017448	3.829737
162	1	0	-3.952973	5.187402	4.656306
163	1	0	-2.337544	4.593410	4.247612
164	1	0	-3.021818	5.987408	3.390796
165	6	0	-5.033615	4.780898	2.069423
166	1	0	-5.773376	5.148966	2.789375
167	1	0	-4.659346	5.636034	1.501131
168	1	0	-5.543798	4.102505	1.376448
169	6	0	-4.514924	2.899559	3.611725
170	1	0	-5.053874	2.191623	2.973184
171	1	0	-3.760883	2.348010	4.184443
172	1	0	-5.237420	3.307910	4.325177
173	6	0	-0.967238	4.778416	-1.441240

174	6	0	0.570999	4.709318	-1.424471
175	1	0	0.975566	5.280703	-2.267678
176	1	0	0.985462	5.130074	-0.503185
177	1	0	0.928990	3.678249	-1.514296
178	6	0	-1.400383	6.255595	-1.457068
179	1	0	-0.948318	6.849183	-0.662707
180	1	0	-1.076551	6.693747	-2.407531
181	1	0	-2.486462	6.363016	-1.393259
182	6	0	-1.466618	4.188382	-2.778815
183	1	0	-1.154076	3.154187	-2.939009
184	1	0	-2.559354	4.225240	-2.835003
185	1	0	-1.066178	4.780196	-3.608671
186	8	0	-1.288066	-1.563142	-2.756202
187	6	0	-0.306299	-1.355286	-3.560624
188	1	0	0.895671	-0.693766	-2.615660
189	6	0	0.512753	-2.503977	-3.976560
190	6	0	0.378417	-3.841280	-3.764178
191	8	0	1.616646	-2.247487	-4.728370
192	6	0	1.481231	-4.455440	-4.434091
193	1	0	-0.412635	-4.325180	-3.210246
194	6	0	2.196520	-3.444620	-4.992812
195	1	0	1.707852	-5.510220	-4.488176
196	1	0	3.097712	-3.411182	-5.586119
197	6	0	-0.466497	-0.214926	-4.572726
198	1	0	-0.859553	-0.647218	-5.497348
199	1	0	0.473034	0.292646	-4.777639
200	8	0	-1.363226	0.746185	-4.041783
201	6	0	-2.683669	0.448139	-4.135752
202	8	0	-3.104499	-0.445632	-4.836201
203	6	0	-3.525549	1.373363	-3.315397
204	1	0	-3.068613	1.553093	-2.340256
205	1	0	-3.613173	2.332816	-3.834224
206	1	0	-4.518404	0.939346	-3.197634

4a(*R*) -1c

RwB97XD SCF energy -4902.523889 a.u.

RwB97XD SCF enthalpy -4900.641546 a.u.

RwB97XD SCF free energy -4900.873138 a.u.

Three lowest frequencies (cm^{-1}) 19.0, 22.7, 24.8

Cartesian coordinates:

Center Number	Atomic Number	Atomic Type	Coordinates (Angstroms) X	Y	Z
1	6	0	0.057030	0.992989	2.746118
2	6	0	-0.483145	1.849688	3.677821
3	6	0	-0.223792	3.217795	3.689406
4	6	0	0.593273	3.807265	2.755329
5	6	0	1.172249	2.954204	1.802962
6	6	0	0.925673	1.582883	1.789156
7	1	0	0.796164	4.871522	2.761128
8	1	0	1.840751	3.388513	1.068197
9	6	0	-0.238540	-0.459414	2.831213
10	6	0	-0.912053	-1.226209	1.841768
11	6	0	0.174773	-1.148691	3.949454
12	6	0	-1.080890	-2.601005	2.008379
13	6	0	-0.007236	-2.518288	4.109260
14	6	0	-0.621202	-3.280824	3.145486
15	1	0	-1.596392	-3.178096	1.250189
16	1	0	-0.760344	-4.348963	3.261122
17	8	0	-0.898748	3.790267	4.724324
18	8	0	-1.338176	1.542096	4.692888
19	8	0	0.844352	-0.651526	5.025196
20	8	0	0.529243	-2.909444	5.295265
21	6	0	-1.312685	2.690915	5.540340
22	1	0	-2.308248	2.877711	5.937231
23	1	0	-0.576483	2.536678	6.338417
24	6	0	0.828127	-1.698820	5.997257
25	1	0	0.038289	-1.499089	6.730062
26	1	0	1.809621	-1.781173	6.463774
27	15	0	-1.493873	-0.432883	0.305796
28	15	0	1.607646	0.553659	0.453524
29	46	0	0.121010	-0.004623	-1.190100
30	6	0	-2.337500	1.099434	0.751364
31	6	0	-3.289645	1.147177	1.770054
32	6	0	-2.067147	2.235146	0.005058
33	6	0	-3.942196	2.335159	2.084075
34	1	0	-3.514187	0.238529	2.314841
35	6	0	-2.685193	3.460500	0.264820
36	1	0	-1.337015	2.169960	-0.794521
37	6	0	-3.552224	3.501572	1.373059
38	6	0	-2.694788	-1.570030	-0.429275
39	6	0	-4.067633	-1.365315	-0.319914
40	6	0	-2.217917	-2.654110	-1.155303
41	6	0	-4.976758	-2.292946	-0.823729
42	1	0	-4.430523	-0.474817	0.173455
43	6	0	-3.076078	-3.595425	-1.726556
44	1	0	-1.147505	-2.759028	-1.294409
45	6	0	-4.449777	-3.451077	-1.442907
46	6	0	2.881953	1.500817	-0.426187
47	6	0	4.199443	1.057671	-0.421053
48	6	0	2.483173	2.472403	-1.344605
49	6	0	5.158446	1.603678	-1.274078
50	1	0	4.471505	0.245966	0.237545
51	6	0	3.391055	3.065259	-2.219307
52	1	0	1.430944	2.727527	-1.401913
53	6	0	4.748467	2.685089	-2.083690
54	6	0	2.468931	-0.868242	1.148393
55	6	0	2.493082	-2.011333	0.365158
56	6	0	3.192292	-0.822908	2.338910
57	6	0	3.289032	-3.113732	0.686777
58	1	0	1.883838	-2.036185	-0.533565
59	6	0	4.025790	-1.876498	2.705219
60	1	0	3.108160	0.056516	2.965831
61	6	0	4.133210	-2.969238	1.804223
62	6	0	3.128698	-4.361075	-0.211797
63	6	0	3.744199	-4.086964	-1.596915
64	1	0	3.579196	-4.945679	-2.257236
65	1	0	3.288769	-3.209687	-2.070505
66	1	0	4.823097	-3.913967	-1.534562
67	6	0	3.718418	-5.658879	0.365958
68	1	0	3.353535	-5.847840	1.379703
69	1	0	3.399473	-6.494561	-0.266153
70	1	0	4.808248	-5.666575	0.386421
71	6	0	1.616584	-4.628736	-0.399445
72	1	0	1.115777	-4.760658	0.566168
73	1	0	1.105549	-3.826952	-0.940583
74	1	0	1.480954	-5.545754	-0.981542
75	6	0	4.727136	-1.874675	4.079309
76	6	0	4.232776	-3.099214	4.874415
77	1	0	4.514330	-4.034717	4.385955
78	1	0	4.669382	-3.094224	5.879436
79	1	0	3.142564	-3.083124	4.975937
80	6	0	6.262973	-1.913796	3.971742
81	1	0	6.634935	-1.138386	3.293454
82	1	0	6.700178	-1.728397	4.959111
83	1	0	6.628348	-2.882457	3.631008
84	6	0	4.368084	-0.616967	4.887930
85	1	0	4.750476	0.295882	4.417872
86	1	0	3.289742	-0.505493	5.030989
87	1	0	4.828317	-0.692378	5.878138
88	8	0	5.090411	-3.917162	2.069056
89	8	0	5.698601	3.356986	-2.816607
90	8	0	-4.072560	4.701209	1.789694
91	8	0	-5.334923	-4.442982	-1.788763
92	6	0	-5.319198	-5.553345	-0.895902
93	1	0	-6.134043	-6.212735	-1.200495
94	1	0	-4.375601	-6.104648	-0.948977
95	1	0	-5.476947	-5.232581	0.139356
96	6	0	-2.427169	-4.655531	-2.647934
97	6	0	-3.406511	-5.393851	-3.576863
98	1	0	-2.822581	-5.990714	-4.285863
99	1	0	-4.071177	-6.079023	-3.051263
100	1	0	-4.018448	-4.693162	-4.152706
101	6	0	-1.649386	-5.675332	-1.798199
102	1	0	-1.087072	-6.357480	-2.445750
103	1	0	-0.936538	-5.171293	-1.138920
104	1	0	-2.314217	-6.278066	-1.171850
105	6	0	6.262005	-3.770987	1.268693
106	1	0	6.971229	-4.525924	1.612967
107	1	0	6.052127	-3.935413	0.207694
108	1	0	6.703489	-2.776152	1.390596
109	6	0	6.251135	4.468858	-2.105277
110	1	0	7.002435	4.913975	-2.759826
111	1	0	5.484597	5.211517	-1.869647
112	1	0	6.720193	4.150566	-1.170953
113	6	0	6.520717	0.889304	-1.436868
114	6	0	6.603959	-0.355531	-0.532775
115	1	0	6.572047	-0.097306	0.531567
116	1	0	5.804194	-1.075146	-0.738256
117	1	0	7.555225	-0.863657	-0.718484
118	6	0	6.621423	0.402230	-2.897179

119	1	0	6.592530	1.237046	-3.601656
120	1	0	7.564697	-0.135235	-3.045946
121	1	0	5.800085	-0.281784	-3.138811
122	6	0	7.739939	1.768496	-1.107194
123	1	0	7.916071	2.531162	-1.866976
124	1	0	7.631786	2.255885	-0.132572
125	1	0	8.637912	1.142235	-1.066093
126	6	0	2.874357	3.908628	-3.408835
127	6	0	1.345286	4.068548	-3.357726
128	1	0	0.825093	3.105330	-3.370746
129	1	0	1.020326	4.629212	-2.476139
130	1	0	1.016211	4.624020	-4.241417
131	6	0	3.469553	5.324777	-3.492459
132	1	0	2.935809	5.893601	-4.261436
133	1	0	3.358154	5.862950	-2.545181
134	1	0	4.523425	5.311992	-3.771495
135	6	0	3.214197	3.135093	-4.699113
136	1	0	2.855121	3.690648	-5.572836
137	1	0	4.291659	2.989633	-4.810324
138	1	0	2.730019	2.152309	-4.701988
139	6	0	-6.492221	-2.011985	-0.743050
140	6	0	-1.427204	-3.932630	-3.582522
141	1	0	-1.924031	-3.130123	-4.137025
142	1	0	-0.576532	-3.499933	-3.049061
143	1	0	-1.021212	-4.649385	-4.304195
144	6	0	-7.098077	-2.054660	-2.159128
145	1	0	-8.156926	-1.775189	-2.120741
146	1	0	-6.582396	-1.355524	-2.824176
147	1	0	-7.024228	-3.050936	-2.598260
148	6	0	-7.221155	-3.024336	0.160314
149	1	0	-6.733005	-3.109388	1.137236
150	1	0	-8.250221	-2.687726	0.329309
151	1	0	-7.272532	-4.014942	-0.292625
152	6	0	-6.765957	-0.613176	-0.162687
153	1	0	-6.286820	0.180264	-0.745246
154	1	0	-7.844377	-0.427361	-0.176603
155	1	0	-6.435103	-0.527150	0.878234
156	6	0	-3.174389	5.442292	2.615296
157	1	0	-3.703039	6.348099	2.917557
158	1	0	-2.889201	4.870981	3.504013
159	1	0	-2.264957	5.720242	2.073486
160	6	0	-5.131243	2.324184	3.069244
161	6	0	-4.990531	3.329443	4.226794
162	1	0	-5.760295	3.125532	4.978796
163	1	0	-4.017111	3.234763	4.715048
164	1	0	-5.117772	4.359911	3.897263
165	6	0	-6.404958	2.650207	2.263228
166	1	0	-7.280364	2.638107	2.922532
167	1	0	-6.341604	3.639152	1.801598
168	1	0	-6.566755	1.909774	1.471854
169	6	0	-5.313703	0.935517	3.704280
170	1	0	-5.511066	0.156479	2.960645
171	1	0	-4.437990	0.640399	4.293607
172	1	0	-6.174006	0.962123	4.380017
173	6	0	-2.340463	4.617732	-0.702810
174	6	0	-0.907832	5.105072	-0.419420
175	1	0	-0.630635	5.888636	-1.133430
176	1	0	-0.810435	5.519522	0.588499
177	1	0	-0.185626	4.287041	-0.516169
178	6	0	-3.304827	5.815691	-0.658281
179	1	0	-3.224833	6.398210	0.259086
180	1	0	-3.060140	6.484874	-1.490165
181	1	0	-4.345499	5.500231	-0.777594
182	6	0	-2.394920	4.085497	-2.155419
183	1	0	-1.662244	3.299607	-2.357568
184	1	0	-3.388006	3.690731	-2.392791
185	1	0	-2.179507	4.904883	-2.849091
186	8	0	-1.199061	-0.268032	-2.717333
187	6	0	-0.715077	0.435213	-3.826378
188	1	0	-0.664791	1.520131	-3.612717
189	6	0	0.680375	-0.013224	-4.138534
190	6	0	1.402450	-0.533043	-5.157939
191	8	0	1.478236	0.056747	-3.017336
192	6	0	2.718969	-0.806318	-4.639302
193	1	0	1.049057	-0.705892	-6.164538
194	6	0	2.713639	-0.439532	-3.338066
195	1	0	3.559448	-1.218635	-5.179019
196	1	0	3.445148	-0.432727	-2.544856
197	6	0	-1.694390	0.286737	-4.981234
198	1	0	-1.892370	-0.761536	-5.212619
199	1	0	-1.349522	0.811944	-5.873933
200	8	0	-2.894445	0.949472	-4.560364
201	6	0	-3.850498	0.226647	-3.951290
202	8	0	-3.933841	-0.982573	-4.013915
203	6	0	-4.780306	1.117335	-3.182944
204	1	0	-4.333458	1.287024	-2.196378
205	1	0	-4.899763	2.084450	-3.674185
206	1	0	-5.749038	0.636001	-3.053465

5a(*R*) -1c

RwB97XD SCF energy -4903.694786 a.u.
RwB97XD SCF enthalpy -4901.794460 a.u.
RwB97XD SCF free energy -4902.028199 a.u.
Three lowest frequencies (cm^{-1}) 7.3, 19.1, 21.9
Cartesian coordinates:

Center Number	Atomic Number	Atomic Type	Coordinates (Angstroms) X	Y	Z
1	6	0	-0.717470	-1.356410	2.327656
2	6	0	-0.589974	-2.445858	3.159067
3	6	0	-1.183775	-3.675670	2.894797
4	6	0	-1.920439	-3.890632	1.755160
5	6	0	-2.075606	-2.793593	0.894274
6	6	0	-1.505942	-1.549366	1.163587
7	1	0	-2.376659	-4.849372	1.539271
8	1	0	-2.671553	-2.931370	-0.000160
9	6	0	-0.094194	-0.070815	2.718959
10	6	0	0.869126	0.653543	1.966293
11	6	0	-0.477805	0.495209	3.916316
12	6	0	1.327684	1.889828	2.425647
13	6	0	-0.006237	1.723174	4.365424
14	6	0	0.890054	2.459073	3.629429
15	1	0	2.063457	2.440716	1.853780
16	1	0	1.259796	3.420244	3.966088
17	8	0	-0.880066	-4.543382	3.899879
18	8	0	0.123176	-2.529645	4.317885
19	8	0	-1.377135	0.003568	4.813713
20	8	0	-0.589772	2.021119	5.558307
21	6	0	-0.357382	-3.716934	4.945280
22	1	0	0.464729	-4.226110	5.445115
23	1	0	-1.166339	-3.459628	5.639550
24	6	0	-1.198219	0.798995	5.985415
25	1	0	-0.521926	0.280836	6.675596
26	1	0	-2.166687	1.005826	6.438770
27	15	0	1.472959	0.039638	0.347085
28	15	0	-1.749212	-0.176809	-0.012872
29	46	0	0.025392	0.519191	-1.347613
30	6	0	1.715073	-1.750097	0.458422
31	6	0	2.370498	-2.331431	1.544116
32	6	0	1.275224	-2.549475	-0.586587
33	6	0	2.543390	-3.708885	1.626232
34	1	0	2.736817	-1.689766	2.335810
35	6	0	1.414158	-3.941037	-0.569235
36	1	0	0.789536	-2.078824	-1.435247
37	6	0	1.972524	-4.501129	0.595408
38	6	0	3.093516	0.814842	0.125798
39	6	0	4.275134	0.117338	0.367383
40	6	0	3.151338	2.143521	-0.273547
41	6	0	5.510793	0.757550	0.330406
42	1	0	4.227122	-0.938221	0.593053
43	6	0	4.359806	2.837324	-0.364466
44	1	0	2.228056	2.649403	-0.528922
45	6	0	5.516263	2.145959	0.051273
46	6	0	-3.076384	-0.740100	-1.111647
47	6	0	-4.411641	-0.494896	-0.816549
48	6	0	-2.736250	-1.378841	-2.300154
49	6	0	-5.435495	-0.955073	-1.645263
50	1	0	-4.653090	0.074800	0.069885
51	6	0	-3.707677	-1.862496	-3.174399
52	1	0	-1.685621	-1.485087	-2.545564
53	6	0	-5.053681	-1.742153	-2.755846
54	6	0	-2.394693	1.239704	0.909140
55	6	0	-2.009894	2.504484	0.491096
56	6	0	-3.266067	1.105996	1.990525
57	6	0	-2.517336	3.668411	1.073232
58	1	0	-1.285522	2.591147	-0.311030
59	6	0	-3.849081	2.223501	2.580953
60	1	0	-3.489904	0.112584	2.360193
61	6	0	-3.528087	3.494729	2.036853
62	6	0	-1.886352	5.001076	0.607452
63	6	0	-2.353352	5.320870	-0.823980

64	1	0	-1.857765	6.228470	-1.186696
65	1	0	-2.109259	4.501589	-1.509031
66	1	0	-3.433978	5.487287	-0.873666
67	6	0	-2.168711	6.203653	1.524411
68	1	0	-1.931883	5.978189	2.568407
69	1	0	-1.530512	7.036115	1.208995
70	1	0	-3.200609	6.552488	1.475914
71	6	0	-0.346255	4.833454	0.587183
72	1	0	0.031167	4.537718	1.571948
73	1	0	-0.004543	4.096707	-0.146041
74	1	0	0.116206	5.788306	0.317172
75	6	0	-4.736731	2.070110	3.833344
76	6	0	-4.125040	2.906208	4.974455
77	1	0	-4.175750	3.975441	4.760594
78	1	0	-4.663880	2.720076	5.910234
79	1	0	-3.073281	2.644593	5.126545
80	6	0	-6.189453	2.513258	3.581441
81	1	0	-6.608426	2.018251	2.698077
82	1	0	-6.809349	2.238642	4.442446
83	1	0	-6.274162	3.591897	3.449330
84	6	0	-4.785518	0.606488	4.303118
85	1	0	-5.276286	-0.045381	3.571838
86	1	0	-3.788700	0.206104	4.510388
87	1	0	-5.365920	0.551063	5.229451
88	8	0	-4.229099	4.578322	2.504619
89	8	0	-6.030611	-2.374486	-3.489351
90	8	0	2.000847	-5.863918	0.754922
91	8	0	6.711908	2.811630	0.170112
92	6	0	6.816643	3.570970	1.371809
93	1	0	7.817652	4.005738	1.381224
94	1	0	6.075054	4.374582	1.409246
95	1	0	6.686143	2.935394	2.254018
96	6	0	4.290157	4.267252	-0.952967
97	6	0	5.640143	4.856171	-1.397366
98	1	0	5.446181	5.784296	-1.945911
99	1	0	6.302379	5.106956	-0.568843
100	1	0	6.172096	4.176175	-2.068859
101	6	0	3.623860	5.213030	0.062311
102	1	0	3.517658	6.215117	-0.368611
103	1	0	2.623030	4.859573	0.335139
104	1	0	4.210303	5.301544	0.982521
105	6	0	-5.246923	5.018698	1.608918
106	1	0	-5.805841	5.798552	2.129424
107	1	0	-4.824984	5.435066	0.689473
108	1	0	-5.923210	4.198748	1.343943
109	6	0	-6.293934	-3.701714	-3.022996
110	1	0	-7.026881	-4.130894	-3.708358
111	1	0	-5.386980	-4.311979	-3.023901
112	1	0	-6.702707	-3.693806	-2.009297
113	6	0	-6.879966	-0.449704	-1.421928
114	6	0	-6.958624	0.486617	-0.200911
115	1	0	-6.714007	-0.033183	0.732174
116	1	0	-6.299586	1.355885	-0.297435
117	1	0	-7.981964	0.863047	-0.108144
118	6	0	-7.278657	0.371053	-2.665360
119	1	0	-7.278261	-0.245684	-3.568010
120	1	0	-8.285269	0.784426	-2.536106
121	1	0	-6.586799	1.206437	-2.818634
122	6	0	-7.915582	-1.566536	-1.200882
123	1	0	-8.115551	-2.124823	-2.116032
124	1	0	-7.596904	-2.264790	-0.419891
125	1	0	-8.862558	-1.119328	-0.880049
126	6	0	-3.296076	-2.334580	-4.587346
127	6	0	-1.786524	-2.135695	-4.817392
128	1	0	-1.475911	-1.096015	-4.662927
129	1	0	-1.177832	-2.779564	-4.173038
130	1	0	-1.551086	-2.395306	-5.854129
131	6	0	-3.601750	-3.817360	-4.863716
132	1	0	-3.101033	-4.122838	-5.788822
133	1	0	-3.231821	-4.460718	-4.058222
134	1	0	-4.668502	-3.999830	-5.000560
135	6	0	-4.034855	-1.457883	-5.619101
136	1	0	-3.733521	-1.741785	-6.633592
137	1	0	-5.117850	-1.575354	-5.541697
138	1	0	-3.791321	-0.399122	-5.477084
139	6	0	6.808762	-0.059704	0.503552
140	6	0	3.415657	4.219699	-2.227985
141	1	0	3.803795	3.481142	-2.935619
142	1	0	2.371771	3.975304	-2.020441
143	1	0	3.425100	5.200068	-2.715320
144	6	0	7.641811	0.056664	-0.787911
145	1	0	8.565894	-0.524573	-0.691667
146	1	0	7.084570	-0.326071	-1.648506
147	1	0	7.914244	1.094083	-0.994743
148	6	0	7.652513	0.406882	1.705321
149	1	0	7.049544	0.463417	2.618177
150	1	0	8.456743	-0.315307	1.884560
151	1	0	8.118448	1.377019	1.532150
152	6	0	6.504735	-1.551480	0.730817
153	1	0	5.933269	-1.993792	-0.091041
154	1	0	7.449464	-2.099193	0.804487
155	1	0	5.957232	-1.718730	1.664768
156	6	0	0.767934	-6.404864	1.229328
157	1	0	0.933356	-7.474079	1.373277
158	1	0	0.476179	-5.951726	2.180714
159	1	0	-0.040201	-6.260528	0.506104
160	6	0	3.424006	-4.309114	2.742418
161	6	0	2.714092	-5.399164	3.566396
162	1	0	3.284322	-5.596088	4.480521
163	1	0	1.711152	-5.081077	3.860561
164	1	0	2.631484	-6.339778	3.022495
165	6	0	4.680207	-4.907640	2.078524
166	1	0	5.336974	-5.342180	2.840702
167	1	0	4.414985	-5.695278	1.368206
168	1	0	5.246293	-4.136156	1.544538
169	6	0	3.880571	-3.226260	3.733716
170	1	0	4.459790	-2.431956	3.252063
171	1	0	3.031262	-2.769705	4.254357
172	1	0	4.526779	-3.684977	4.488187
173	6	0	0.929670	-4.699802	-1.829272
174	6	0	-0.609546	-4.664334	-1.879307
175	1	0	-0.965916	-5.191195	-2.771707
176	1	0	-1.053950	-5.146031	-1.002778
177	1	0	-0.984314	-3.636306	-1.922666
178	6	0	1.410155	-6.157642	-1.948183
179	1	0	0.928705	-6.835027	-1.243247
180	1	0	1.167384	-6.515232	-2.954709
181	1	0	2.492845	-6.237921	-1.818272
182	6	0	1.480630	-3.988712	-3.086424
183	1	0	1.196392	-2.935939	-3.159589
184	1	0	2.572926	-4.047329	-3.113373
185	1	0	1.096503	-4.489338	-3.981392
186	8	0	1.600637	1.074333	-2.508851
187	6	0	1.378517	1.260779	-3.870846
188	1	0	0.584940	0.590967	-4.245996
189	6	0	1.010139	2.689151	-4.191132
190	6	0	1.368499	3.555369	-5.176883
191	8	0	0.172875	3.316680	-3.317167
192	6	0	0.716874	4.800224	-4.887254
193	1	0	2.029380	3.343902	-6.005711
194	6	0	0.012899	4.598028	-3.747860
195	1	0	0.778667	5.721541	-5.448922
196	1	0	-0.617697	5.227269	-3.138800
197	6	0	2.658696	0.910786	-4.648286
198	1	0	3.429371	1.666919	-4.494323
199	1	0	2.453946	0.803303	-5.715458
200	8	0	3.137721	-0.357519	-4.194378
201	6	0	4.142555	-0.348196	-3.300426
202	8	0	4.779161	0.644507	-3.011224
203	6	0	4.368887	-1.710978	-2.722870
204	1	0	3.618772	-1.885532	-1.943706
205	1	0	4.252225	-2.481260	-3.487303
206	1	0	5.362718	-1.766988	-2.279603
207	1	0	-1.196955	1.437915	-2.475683
208	1	0	-1.171261	0.739781	-2.815362

TS2a(*R*) -1c

RwB97XD SCF energy -4903.677710 a.u.
RwB97XD SCF enthalpy -4901.782482 a.u.
RwB97XD SCF free energy -4902.026212 a.u.
Three lowest frequencies (cm^{-1}) -1181, 5.2, 10.9
Imaginary frequency (cm^{-1}) -1181
Cartesian coordinates:

Center Number	Atomic Number	Atomic Type	Coordinates (Angstroms) X	Y	Z
1	6	0	0.980307	-0.858541	2.490991
2	6	0	0.986353	-0.724569	3.862247
3	6	0	1.660963	0.298549	4.525071
4	6	0	2.356133	1.265517	3.844316
5	6	0	2.354928	1.164680	2.445786

6	6	0	1.694150	0.139603	1.771676
7	1	0	2.887351	2.059486	4.355091
8	1	0	2.904394	1.911582	1.885924
9	6	0	0.363714	-2.070625	1.890547
10	6	0	-0.706526	-2.114503	0.954333
11	6	0	0.877714	-3.292008	2.274052
12	6	0	-1.157597	-3.339699	0.463230
13	6	0	0.418354	-4.506622	1.776643
14	6	0	-0.599428	-4.564941	0.857169
15	1	0	-1.978673	-3.369353	-0.242085
16	1	0	-0.967013	-5.504738	0.462802
17	8	0	1.507199	0.144694	5.866683
18	8	0	0.385034	-1.531658	4.779727
19	8	0	1.903568	-3.521263	3.140921
20	8	0	1.139662	-5.521481	2.328425
21	6	0	0.972897	-1.171829	6.029239
22	1	0	0.211859	-1.164903	6.807539
23	1	0	1.788783	-1.867543	6.259007
24	6	0	1.848666	-4.920635	3.415439
25	1	0	1.293334	-5.085367	4.346550
26	1	0	2.859053	-5.324618	3.464594
27	15	0	-1.428124	-0.557349	0.312926
28	15	0	1.770069	0.091476	-0.047333
29	46	0	-0.105575	0.437658	-1.285933
30	6	0	-1.752463	0.486735	1.753959
31	6	0	-2.453007	-0.003371	2.852295
32	6	0	-1.283980	1.796304	1.763773
33	6	0	-2.660217	0.776413	3.991350
34	1	0	-2.830368	-1.020663	2.824307
35	6	0	-1.452745	2.630475	2.869142
36	1	0	-0.754718	2.163577	0.891027
37	6	0	-2.061705	2.057317	4.007370
38	6	0	-2.989055	-1.019686	-0.490318
39	6	0	-4.244161	-0.756609	0.054603
40	6	0	-2.898996	-1.654669	-1.725584
41	6	0	-5.408079	-1.203543	-0.571855
42	1	0	-4.309221	-0.203335	0.981673
43	6	0	-4.022576	-2.124843	-2.408016
44	1	0	-1.918042	-1.796326	-2.163993
45	6	0	-5.264477	-1.964579	-1.758142
46	6	0	2.964639	1.377782	-0.498902
47	6	0	4.327072	1.101523	-0.577559
48	6	0	2.503714	2.659068	-0.771991
49	6	0	5.252591	2.108258	-0.835532
50	1	0	4.666687	0.085632	-0.427134
51	6	0	3.375774	3.708608	-1.075487
52	1	0	1.433375	2.841171	-0.765440
53	6	0	4.755029	3.426696	-0.985839
54	6	0	2.480912	-1.508260	-0.505724
55	6	0	2.022294	-2.134338	-1.655098
56	6	0	3.479645	-2.115190	0.258655
57	6	0	2.590535	-3.319427	-2.130872
58	1	0	1.192782	-1.691964	-2.197534
59	6	0	4.118388	-3.269127	-0.184508
60	1	0	3.761127	-1.664136	1.202260
61	6	0	3.723658	-3.790086	-1.443645
62	6	0	1.877386	-3.991052	-3.326150
63	6	0	2.005068	-3.100159	-4.575368
64	1	0	1.490632	-3.566275	-5.423094
65	1	0	1.555790	-2.113970	-4.414878
66	1	0	3.052717	-2.951393	-4.855811
67	6	0	2.350821	-5.413005	-3.672523
68	1	0	2.350397	-6.067614	-2.796889
69	1	0	1.652129	-5.834466	-4.403670
70	1	0	3.342737	-5.440793	-4.125158
71	6	0	0.379896	-4.123589	-2.963520
72	1	0	0.245579	-4.762610	-2.084012
73	1	0	-0.095096	-3.160661	-2.756690
74	1	0	-0.156188	-4.579785	-3.801983
75	6	0	5.146868	-3.988571	0.710990
76	6	0	4.666510	-5.436099	0.938038
77	1	0	4.674753	-6.010720	0.009984
78	1	0	5.318970	-5.941008	1.658945
79	1	0	3.645143	-5.446656	1.331637
80	6	0	6.558711	-3.993635	0.096113
81	1	0	6.871191	-2.981187	-0.183282
82	1	0	7.276598	-4.372195	0.832386
83	1	0	6.623643	-4.634317	-0.783869
84	6	0	5.255241	-3.313201	2.088435
85	1	0	5.666651	-2.299947	2.019608
86	1	0	4.289090	-3.261340	2.599423
87	1	0	5.933809	-3.898234	2.717152
88	8	0	4.479946	-4.801956	-1.981097
89	8	0	5.673265	4.443612	-1.070770
90	8	0	-2.056376	2.761383	5.181840
91	8	0	-6.404265	-2.519163	-2.286941
92	6	0	-6.582200	-3.905172	-2.006697
93	1	0	-7.632611	-4.129593	-2.202747
94	1	0	-5.963326	-4.533309	-2.653322
95	1	0	-6.348352	-4.133916	-0.962133
96	6	0	-3.787548	-2.777538	-3.791231
97	6	0	-5.016642	-2.757524	-4.718431
98	1	0	-4.702070	-3.067997	-5.720660
99	1	0	-5.810653	-3.434234	-4.406399
100	1	0	-5.437643	-1.750381	-4.799932
101	6	0	-3.297116	-4.224252	-3.590490
102	1	0	-3.064743	-4.683160	-4.558120
103	1	0	-2.390075	-4.245256	-2.977818
104	1	0	-4.045268	-4.848510	-3.094643
105	6	0	5.355317	-4.346778	-3.010989
106	1	0	5.911409	-5.219893	-3.356503
107	1	0	4.803347	-3.912951	-3.850869
108	1	0	6.055384	-3.594748	-2.630386
109	6	0	5.812191	5.176910	0.144515
110	1	0	6.604887	5.908748	-0.020397
111	1	0	4.888277	5.699537	0.411005
112	1	0	6.090539	4.517116	0.973184
113	6	0	6.741987	1.754205	-1.027372
114	6	0	6.972438	0.235395	-0.923511
115	1	0	6.750609	-0.146888	0.078931
116	1	0	6.377332	-0.328203	-1.649881
117	1	0	8.026464	0.023324	-1.128070
118	6	0	7.180288	2.192537	-2.438582
119	1	0	7.049016	3.266743	-2.584810
120	1	0	8.239168	1.955545	-2.591468
121	1	0	6.602288	1.667166	-3.206912
122	6	0	7.645402	2.421470	0.026804
123	1	0	7.740809	3.495280	-0.135953
124	1	0	7.266666	2.251012	1.040184
125	1	0	8.650816	1.990307	-0.031089
126	6	0	2.728405	5.038573	-1.531578
127	6	0	2.035866	5.718943	-0.337275
128	1	0	1.260957	5.073514	0.086717
129	1	0	2.746112	5.963351	0.458760
130	1	0	1.556539	6.649705	-0.660763
131	6	0	3.688348	6.035835	-2.203640
132	1	0	3.091413	6.847646	-2.632875
133	1	0	4.398138	6.490025	-1.512060
134	1	0	4.249770	5.569232	-3.018140
135	6	0	1.651511	4.708787	-2.592804
136	1	0	1.220397	5.637776	-2.978884
137	1	0	2.083844	4.161291	-3.436879
138	1	0	0.824318	4.117009	-2.190490
139	6	0	-6.794316	-0.819810	-0.007142
140	6	0	-2.681811	-2.006448	-4.547806
141	1	0	-2.932170	-0.946206	-4.652350
142	1	0	-1.701368	-2.080879	-4.070835
143	1	0	-2.573863	-2.431180	-5.551070
144	6	0	-7.589722	-0.056399	-1.084492
145	1	0	-8.557879	0.257038	-0.677470
146	1	0	-7.050991	0.841422	-1.402433
147	1	0	-7.773994	-0.675352	-1.964160
148	6	0	-7.601324	-2.047149	0.459854
149	1	0	-7.001468	-2.691749	1.111368
150	1	0	-8.470841	-1.710422	1.035178
151	1	0	-7.975472	-2.642265	-0.372601
152	6	0	-6.661830	0.104458	1.214007
153	1	0	-6.095034	1.013250	0.992264
154	1	0	-7.661683	0.409591	1.538775
155	1	0	-6.186537	-0.405775	2.056587
156	6	0	-0.909213	2.476937	5.974417
157	1	0	-1.025268	3.028861	6.909076
158	1	0	-0.832762	1.404621	6.188614
159	1	0	0.009151	2.803626	5.471133
160	6	0	-3.524295	0.161631	5.119011
161	6	0	-2.759222	-0.996081	5.781986
162	1	0	-3.396220	-1.486562	6.526551
163	1	0	-2.445020	-1.749922	5.054023
164	1	0	-1.869036	-0.622511	6.296052
165	6	0	-3.974213	1.133708	6.224012
166	1	0	-4.706125	0.614948	6.853256
167	1	0	-3.155751	1.451616	6.871036
168	1	0	-4.457353	2.025012	5.814860
169	6	0	-4.821464	-0.386946	4.485993

170	1	0	-5.385542	0.419704	4.005990
171	1	0	-4.638611	-1.166674	3.741945
172	1	0	-5.455364	-0.824823	5.264148
173	6	0	-0.950086	4.089882	2.739539
174	6	0	0.586938	4.101962	2.717350
175	1	0	0.952013	5.128593	2.601300
176	1	0	0.998928	3.698803	3.647113
177	1	0	0.981145	3.507689	1.886729
178	6	0	-1.426762	5.052918	3.840135
179	1	0	-0.954035	4.862272	4.804238
180	1	0	-1.160513	6.072800	3.540312
181	1	0	-2.511293	5.016967	3.975138
182	6	0	-1.467277	4.675910	1.407073
183	1	0	-1.143723	4.108035	0.531147
184	1	0	-2.561498	4.719002	1.395189
185	1	0	-1.088907	5.696962	1.289144
186	8	0	-1.430234	0.831616	-2.948453
187	6	0	-1.981088	2.126564	-3.041691
188	6	0	-3.146782	2.321260	-2.125218
189	6	0	-4.485361	2.482345	-2.303836
190	8	0	-2.847031	2.425070	-0.801136
191	6	0	-5.043876	2.705499	-1.002850
192	1	0	-5.012712	2.469046	-3.246969
193	6	0	-4.006567	2.647219	-0.131447
194	1	0	-6.080101	2.884787	-0.755413
195	1	0	-3.925055	2.736943	0.941115
196	6	0	-2.372431	2.341024	-4.504631
197	1	0	-3.214291	1.703212	-4.780707
198	1	0	-1.516608	2.106984	-5.140073
199	8	0	-2.810402	3.684973	-4.742486
200	6	0	-1.860356	4.612440	-4.936628
201	8	0	-0.672970	4.355305	-4.959246
202	1	0	-1.222867	2.882188	-2.777181
203	1	0	0.823042	1.101605	-2.637196
204	1	0	-0.029999	1.024997	-2.991831
205	6	0	-2.457356	5.975024	-5.113190
206	1	0	-1.677770	6.690499	-5.372810
207	1	0	-3.223134	5.952427	-5.892609
208	1	0	-2.940891	6.279823	-4.180230

6c(*R*)•2a

RwB97XD SCF energy -4903.726097 a.u.
RwB97XD SCF enthalpy -4901.822114 a.u.
RwB97XD SCF free energy -4902.058888 a.u.
Three lowest frequencies (cm^{-1}) 14.2, 17.6, 20.0
Cartesian coordinates:
Standard orientation:

Center Number	Atomic Number	Atomic Type	Coordinates (Angstroms) X	Y	Z
1	6	0	-0.859476	0.056645	2.499473
2	6	0	-0.709023	-0.560051	3.722282
3	6	0	-1.243794	-1.806676	4.024283
4	6	0	-1.958627	-2.520161	3.094387
5	6	0	-2.137409	-1.911949	1.842813
6	6	0	-1.616582	-0.655260	1.528819
7	1	0	-2.379748	-3.494047	3.313429
8	1	0	-2.714531	-2.455698	1.105328
9	6	0	-0.249660	1.402157	2.343461
10	6	0	0.837756	1.722593	1.490994
11	6	0	-0.705440	2.419882	3.153939
12	6	0	1.350373	3.019543	1.465224
13	6	0	-0.181491	3.706053	3.127528
14	6	0	0.845521	4.045000	2.280293
15	1	0	2.174567	3.258522	0.802470
16	1	0	1.259800	5.045876	2.250489
17	8	0	-0.919901	-2.136350	5.306218
18	8	0	-0.016042	-0.093919	4.801930
19	8	0	-1.710540	2.358814	4.073111
20	8	0	-0.850805	4.482293	4.029974
21	6	0	-0.447510	-0.914825	5.883885
22	1	0	0.389927	-1.119518	6.548118
23	1	0	-1.276294	-0.422857	6.407764
24	6	0	-1.577744	3.555331	4.840147
25	1	0	-1.004632	3.340529	5.750485
26	1	0	-2.564335	3.959038	5.063952
27	15	0	1.469636	0.463151	0.320850
28	15	0	-1.869144	0.003068	-0.169657
29	46	0	-0.033553	-0.041149	-1.473582
30	6	0	1.770954	-1.062445	1.265587
31	6	0	2.420787	-1.099819	2.498757
32	6	0	1.339107	-2.250669	0.689124
33	6	0	2.611945	-2.300518	3.178464
34	1	0	2.768958	-0.169645	2.930068
35	6	0	1.486382	-3.491578	1.315918
36	1	0	0.854573	-2.205151	-0.280644
37	6	0	2.058487	-3.473922	2.602416
38	6	0	3.076158	1.120637	-0.229818
39	6	0	4.275574	0.845327	0.422483
40	6	0	3.091810	1.912722	-1.371124
41	6	0	5.481756	1.395434	-0.005041
42	1	0	4.261871	0.191268	1.282404
43	6	0	4.270164	2.466969	-1.883618
44	1	0	2.151911	2.103800	-1.878922
45	6	0	5.443610	2.259786	-1.128200
46	6	0	-3.243089	-1.011377	-0.795493
47	6	0	-4.570691	-0.678549	-0.556856
48	6	0	-2.942344	-2.172973	-1.500403
49	6	0	-5.613815	-1.528645	-0.928926
50	1	0	-4.797443	0.266774	-0.084180
51	6	0	-3.931956	-3.077013	-1.882467
52	1	0	-1.907230	-2.366959	-1.750324
53	6	0	-5.252816	-2.781999	-1.472413
54	6	0	-2.475986	1.700953	0.006095
55	6	0	-1.985138	2.657429	-0.869706
56	6	0	-3.399988	2.075458	0.983960
57	6	0	-2.438749	3.979623	-0.861138
58	1	0	-1.216906	2.362635	-1.577446
59	6	0	-3.945827	3.355760	1.000958
60	1	0	-3.697209	1.346540	1.727828
61	6	0	-3.515517	4.263273	-0.001521
62	6	0	-1.675922	4.977010	-1.764055
63	6	0	-1.963027	4.670641	-3.244968
64	1	0	-1.374594	5.336285	-3.886410
65	1	0	-1.696835	3.638626	-3.498149
66	1	0	-3.020320	4.814360	-3.488389
67	6	0	-1.964523	6.461772	-1.483402
68	1	0	-1.841235	6.700762	-0.423259
69	1	0	-1.245895	7.065107	-2.048710
70	1	0	-2.960840	6.777205	-1.793957
71	6	0	-0.161291	4.789417	-1.507633
72	1	0	0.084110	4.978033	-0.457340
73	1	0	0.194870	3.787390	-1.764401
74	1	0	0.400617	5.500841	-2.121669
75	6	0	-4.920327	3.776644	2.120097
76	6	0	-4.356978	5.023318	2.831380
77	1	0	-4.363127	5.896159	2.176311
78	1	0	-4.958437	5.256741	3.717058
79	1	0	-3.324815	4.853622	3.152477
80	6	0	-6.329942	4.077914	1.577660
81	1	0	-6.707688	3.246406	0.972028
82	1	0	-7.021734	4.220195	2.415486
83	1	0	-6.354806	4.987659	0.976627
84	6	0	-5.069485	2.666534	3.174288
85	1	0	-5.532624	1.763952	2.760259
86	1	0	-4.109262	2.390541	3.620001
87	1	0	-5.721389	3.026114	3.976422
88	8	0	-4.176116	5.463299	-0.096488
89	8	0	-6.229564	-3.735681	-1.652032
90	8	0	2.114236	-4.629763	3.342632
91	8	0	6.616380	2.879695	-1.486133
92	6	0	6.703002	4.238603	-1.064503
93	1	0	7.715585	4.574539	-1.295355
94	1	0	5.988093	4.874179	-1.594207
95	1	0	6.522863	4.334499	0.010728
96	6	0	4.161098	3.224403	-3.231340
97	6	0	5.491648	3.397197	-3.987694
98	1	0	5.271052	3.775138	-4.991564
99	1	0	6.171627	4.108371	-3.520933
100	1	0	6.015797	2.443148	-4.098265
101	6	0	3.508025	4.598999	-2.997023
102	1	0	3.365781	5.116712	-3.952399
103	1	0	2.527102	4.486623	-2.523436
104	1	0	4.118147	5.240376	-2.354347
105	6	0	-5.093213	5.515362	-1.186170
106	1	0	-5.602604	6.478142	-1.117387
107	1	0	-4.582541	5.444359	-2.151343
108	1	0	-5.830148	4.707007	-1.123291
109	6	0	-6.371163	-4.594462	-0.516375
110	1	0	-7.126125	-5.336256	-0.782425

111	1	0	-5.430903	-5.097225	-0.276072
112	1	0	-6.699170	-4.040230	0.366650
113	6	0	-7.071447	-1.014247	-0.881166
114	6	0	-7.131274	0.435572	-0.365305
115	1	0	-6.802956	0.515039	0.677102
116	1	0	-6.528633	1.118599	-0.973060
117	1	0	-8.167450	0.784804	-0.410525
118	6	0	-7.613862	-1.017169	-2.324730
119	1	0	-7.627094	-2.026213	-2.744322
120	1	0	-8.638130	-0.627554	-2.341961
121	1	0	-6.999521	-0.382970	-2.973151
122	6	0	-8.005474	-1.848888	0.012968
123	1	0	-8.229671	-2.821709	-0.425842
124	1	0	-7.578959	-1.997369	1.010661
125	1	0	-8.957475	-1.320711	0.134265
126	6	0	-3.577537	-4.243583	-2.833268
127	6	0	-2.100194	-4.178271	-3.263869
128	1	0	-1.844343	-3.222467	-3.732680
129	1	0	-1.415628	-4.350071	-2.425632
130	1	0	-1.912850	-4.964625	-4.002040
131	6	0	-3.803900	-5.635961	-2.218330
132	1	0	-3.357981	-6.395336	-2.869897
133	1	0	-3.330898	-5.722685	-1.234051
134	1	0	-4.863529	-5.876638	-2.123950
135	6	0	-4.433310	-4.105664	-4.108307
136	1	0	-4.174007	-4.899328	-4.817815
137	1	0	-5.499764	-4.184567	-3.885612
138	1	0	-4.253004	-3.142514	-4.598440
139	6	0	6.801989	0.972839	0.679051
140	6	0	3.246562	2.426503	-4.191083
141	1	0	3.620578	1.406733	-4.338594
142	1	0	2.207426	2.385670	-3.849774
143	1	0	3.228685	2.918454	-5.168311
144	6	0	7.630346	0.163694	-0.337767
145	1	0	8.564073	-0.176999	0.124172
146	1	0	7.079475	-0.718531	-0.680376
147	1	0	7.885648	0.767243	-1.212916
148	6	0	7.637621	2.161536	1.192598
149	1	0	7.030504	2.843939	1.797185
150	1	0	8.442909	1.783934	1.832097
151	1	0	8.101951	2.724414	0.383759
152	6	0	6.538285	0.074126	1.899824
153	1	0	5.996023	-0.839828	1.644807
154	1	0	7.496938	-0.229804	2.331293
155	1	0	5.980727	0.605875	2.678830
156	6	0	0.892967	-4.924386	4.018169
157	1	0	1.065219	-5.838201	4.589955
158	1	0	0.609191	-4.115223	4.697452
159	1	0	0.074150	-5.094794	3.312920
160	6	0	3.519316	-2.341727	4.427185
161	6	0	2.879171	-3.019107	5.653248
162	1	0	3.501598	-2.832861	6.535102
163	1	0	1.885328	-2.611348	5.856898
164	1	0	2.792767	-4.098287	5.532602
165	6	0	4.800740	-3.104162	4.033041
166	1	0	5.496023	-3.139366	4.879758
167	1	0	4.577080	-4.132124	3.734393
168	1	0	5.309966	-2.606514	3.199383
169	6	0	3.926938	-0.925713	4.865888
170	1	0	4.479372	-0.390382	4.087828
171	1	0	3.057016	-0.323721	5.152165
172	1	0	4.585335	-0.997828	5.736812
173	6	0	1.012610	-4.738969	0.525612
174	6	0	-0.527349	-4.781830	0.501082
175	1	0	-0.864554	-5.651760	-0.074453
176	1	0	-0.953107	-4.859451	1.505727
177	1	0	-0.939781	-3.882580	0.030485
178	6	0	1.553084	-6.084449	1.043853
179	1	0	1.105720	-6.396126	1.987128
180	1	0	1.314476	-6.856489	0.304435
181	1	0	2.639625	-6.063679	1.167263
182	6	0	1.496799	-4.630256	-0.937596
183	1	0	1.059454	-3.779540	-1.464019
184	1	0	2.585869	-4.544638	-0.998357
185	1	0	1.197856	-5.531485	-1.482820
186	8	0	1.617441	-0.324513	-2.854299
187	6	0	1.883715	-1.593729	-3.483634
188	1	0	1.262277	-2.314645	-2.948353
189	6	0	1.491275	-1.569174	-4.915730
190	6	0	2.163108	-1.327440	-6.072938
191	8	0	0.164219	-1.744634	-5.175510
192	6	0	1.185949	-1.362054	-7.119210
193	1	0	3.224522	-1.148307	-6.172764
194	6	0	-0.002453	-1.615516	-6.515763
195	1	0	1.352909	-1.219285	-8.176903
196	1	0	-1.010961	-1.738403	-6.880920
197	6	0	3.346242	-1.942643	-3.283765
198	1	0	3.996113	-1.147901	-3.661083
199	1	0	3.586575	-2.880491	-3.791845
200	8	0	3.535127	-2.098541	-1.872889
201	6	0	4.769766	-2.429487	-1.468303
202	8	0	5.709680	-2.499238	-2.235309
203	6	0	4.820206	-2.743876	-0.004315
204	1	0	4.217613	-2.042179	0.574079
205	1	0	4.404265	-3.745074	0.149704
206	1	0	5.855898	-2.738370	0.334281
207	1	0	-1.075902	-0.338392	-2.608554
208	1	0	1.811306	0.393225	-3.471421

2.8. **Catalytic cycle 2b–1a (*S*)**

1a•2b

RwB97XD SCF energy -3304.679746

RwB97XD SCF enthalpy -3303.806043

RwB97XD SCF free energy -3303.950296

Three lowest frequencies (cm^{-1}) 12.9, 25.0, 31.5

Cartesian coordinates:

Center Number	Atomic Number	Atomic Type	Coordinates (Angstroms) X	Y	Z
1	6	0	-2.941906	-0.127433	0.046451
2	6	0	-4.089045	-0.842060	-0.221302
3	6	0	-4.781872	-0.753832	-1.423275
4	6	0	-4.377950	0.096585	-2.422785
5	6	0	-3.227687	0.859391	-2.170157
6	6	0	-2.512737	0.765129	-0.977293
7	1	0	-4.916994	0.180221	-3.358935
8	1	0	-2.901503	1.544345	-2.943367
9	6	0	-2.282967	-0.331576	1.366753
10	6	0	-1.066501	-1.037981	1.572950
11	6	0	-2.903660	0.150035	2.498017
12	6	0	-0.553493	-1.192915	2.858385
13	6	0	-2.365140	0.016638	3.772462
14	6	0	-1.189467	-0.657934	3.989979
15	1	0	0.369194	-1.739978	3.010360
16	1	0	-0.769369	-0.773249	4.982223
17	8	0	-5.845941	-1.596196	-1.401009
18	8	0	-4.705682	-1.734222	0.598259
19	8	0	-4.066444	0.851558	2.560708
20	8	0	-3.164656	0.648552	4.672759
21	6	0	-5.801058	-2.276903	-0.142327
22	1	0	-5.633830	-3.344043	-0.313668
23	1	0	-6.731472	-2.097221	0.401200
24	6	0	-4.315363	1.091132	3.948197
25	1	0	-5.190376	0.513745	4.263934
26	1	0	-4.459996	2.160554	4.113926
27	15	0	-0.129275	-1.609641	0.104362
28	15	0	-0.932604	1.680593	-0.798665
29	46	0	0.763029	0.224480	-1.180917
30	6	0	-1.307848	-2.664904	-0.797717
31	6	0	-2.099520	-3.610331	-0.136773
32	6	0	-1.471515	-2.461809	-2.171476
33	6	0	-3.040552	-4.347201	-0.848095
34	1	0	-1.989759	-3.761461	0.933355
35	6	0	-2.425981	-3.189206	-2.877485
36	1	0	-0.868843	-1.713111	-2.680359
37	6	0	-3.211724	-4.129550	-2.215066
38	6	0	1.179980	-2.694817	0.760695
39	6	0	1.172584	-4.080958	0.594827
40	6	0	2.277349	-2.081359	1.380849
41	6	0	2.243892	-4.844113	1.055467
42	6	0	3.333284	-2.848171	1.858451
43	1	0	2.311007	-0.999199	1.486837
44	6	0	3.319395	-4.231851	1.691063
45	6	0	-1.030343	3.048488	-2.013793
46	6	0	-1.382410	4.350788	-1.653779
47	6	0	-0.735317	2.764176	-3.354367
48	6	0	-1.442994	5.351444	-2.622739
49	1	0	-1.596238	4.602349	-0.621376
50	6	0	-0.815247	3.760033	-4.319918
51	1	0	-0.441792	1.758259	-3.643107
52	6	0	-1.166906	5.058903	-3.954113
53	6	0	-1.002221	2.425281	0.858399
54	6	0	0.082224	2.283900	1.726902
55	6	0	-2.161150	3.079751	1.292716
56	6	0	0.008381	2.787342	3.022222
57	1	0	0.972458	1.758960	1.395818
58	6	0	-2.224149	3.596520	2.581948
59	1	0	-3.022907	3.162763	0.636953
60	6	0	-1.143837	3.443459	3.449543
61	8	0	5.662737	-2.646129	-0.410591
62	6	0	5.382618	-1.509710	-0.750428
63	1	0	1.267309	1.524218	-1.862141
64	6	0	4.144563	-1.215720	-1.478694
65	6	0	3.711112	-0.142318	-2.195170
66	8	0	3.239274	-2.238605	-1.570188
67	6	0	2.446045	-0.518073	-2.764270
68	1	0	4.223957	0.798859	-2.323683
69	6	0	2.222767	-1.808279	-2.326075
70	1	0	1.920802	-0.018649	-3.566429
71	1	0	1.455182	-2.532773	-2.555318
72	6	0	6.364505	-0.370987	-0.514467
73	1	0	6.928759	-0.198297	-1.434219
74	1	0	7.046327	-0.672437	0.282942
75	8	0	5.745611	0.878809	-0.212213
76	6	0	5.067159	0.921957	0.949060
77	8	0	4.969322	-0.054604	1.663957
78	6	0	4.456799	2.287470	1.220192
79	6	0	3.471191	2.604856	0.083540
80	1	0	2.728111	1.807396	-0.027512
81	1	0	2.938438	3.534629	0.308185
82	1	0	3.983719	2.728449	-0.874283
83	6	0	3.719827	2.239506	2.560672
84	1	0	4.407371	2.025494	3.384190
85	1	0	3.248778	3.208579	2.750166
86	1	0	2.941122	1.471162	2.561475
87	6	0	5.575877	3.340819	1.266239
88	1	0	5.138645	4.320067	1.484762
89	1	0	6.304468	3.109388	2.049857
90	1	0	6.103060	3.407862	0.310861
91	1	0	-0.593349	3.524918	-5.356139
92	1	0	-1.216744	5.840409	-4.705939
93	1	0	-1.705171	6.363246	-2.329633
94	1	0	0.846812	2.658183	3.699573
95	1	0	-1.203575	3.832140	4.461401
96	1	0	-3.124253	4.103476	2.914667
97	1	0	-3.650485	-5.082654	-0.332780
98	1	0	-3.960482	-4.693429	-2.762991
99	1	0	-2.562342	-3.014736	-3.940114
100	1	0	0.341105	-4.572651	0.100933
101	1	0	2.233524	-5.920595	0.914549
102	1	0	4.152751	-4.829465	2.047485
103	1	0	4.177683	-2.361044	2.333129

3b(*S*)$_{seg}$

RwB97XD SCF energy -3304.683413 a.u.

RwB97XD SCF enthalpy -3303.809924 a.u.

RwB97XD SCF free energy -3303.954702 a.u.

Three lowest frequencies (cm^{-1}) 17.5, 19.0, 27.5

Cartesian coordinates:

Center Number	Atomic Number	Atomic Type	Coordinates (Angstroms) X	Y	Z
1	6	0	-2.502710	-0.810336	0.834009
2	6	0	-3.643587	-0.613644	1.579986
3	6	0	-3.651320	-0.594123	2.970448
4	6	0	-2.508213	-0.812766	3.699609
5	6	0	-1.334884	-1.051761	2.968638
6	6	0	-1.312198	-1.055665	1.574925
7	1	0	-2.508798	-0.802322	4.783190
8	1	0	-0.422063	-1.236399	3.522802
9	6	0	-2.613106	-0.755842	-0.650838
10	6	0	-2.148688	0.316417	-1.462171
11	6	0	-3.250430	-1.783001	-1.310418
12	6	0	-2.315994	0.275864	-2.843948
13	6	0	-3.393734	-1.822305	-2.692388
14	6	0	-2.937937	-0.803675	-3.492403
15	1	0	-1.958196	1.096879	-3.454973
16	1	0	-3.055175	-0.826681	-4.569480
17	8	0	-4.905360	-0.328735	3.416319
18	8	0	-4.895494	-0.371869	1.110577
19	8	0	-3.788958	-2.902749	-0.759499
20	8	0	-4.017585	-2.975774	-3.053431
21	6	0	-5.738318	-0.228941	2.256541
22	1	0	-6.216879	0.752868	2.236661
23	1	0	-6.476554	-1.035891	2.266824
24	6	0	-4.355214	-3.651022	-1.838696
25	1	0	-5.443337	-3.679187	-1.725962
26	1	0	-3.927015	-4.655623	-1.850161
27	15	0	-1.167001	1.648220	-0.672474
28	15	0	0.288969	-1.235407	0.709995
29	46	0	0.970749	0.753480	-0.055132
30	6	0	-2.227468	2.299490	0.655561
31	6	0	-3.596654	2.520897	0.471930
32	6	0	-1.652069	2.511807	1.912365
33	6	0	-4.379961	2.951703	1.537798
34	1	0	-4.054577	2.337561	-0.495935

35	6	0	-2.441698	2.931312	2.979595
36	1	0	-0.590377	2.327467	2.059262
37	6	0	-3.805249	3.147176	2.793148
38	6	0	-1.002137	2.952829	-1.937788
39	6	0	-1.813065	4.089766	-1.977300
40	6	0	0.011074	2.795795	-2.894205
41	6	0	-1.618167	5.052011	-2.965499
42	6	0	0.193784	3.752591	-3.886909
43	1	0	0.657338	1.921312	-2.862954
44	6	0	-0.620326	4.883523	-3.921898
45	6	0	1.433476	-1.956795	1.941304
46	6	0	1.664712	-3.329627	2.040786
47	6	0	2.124269	-1.078424	2.785664
48	6	0	2.582695	-3.814915	2.970714
49	1	0	1.156488	-4.028044	1.385431
50	6	0	3.025959	-1.567201	3.722951
51	1	0	1.963639	-0.007429	2.697303
52	6	0	3.260777	-2.938715	3.812589
53	6	0	0.011147	-2.465394	-0.597825
54	6	0	0.468634	-2.200417	-1.892406
55	6	0	-0.711388	-3.635397	-0.338966
56	6	0	0.199995	-3.098345	-2.921500
57	1	0	1.017824	-1.284872	-2.095593
58	6	0	-0.962066	-4.537036	-1.367869
59	1	0	-1.101587	-3.831114	0.655527
60	6	0	-0.514797	-4.265335	-2.659697
61	8	0	2.066303	2.746253	-0.233028
62	6	0	3.225214	2.354950	0.004492
63	1	0	2.293453	-0.022477	0.209156
64	6	0	3.722802	2.224707	1.350375
65	6	0	4.939484	1.847139	1.860475
66	8	0	2.847374	2.463076	2.370506
67	6	0	4.800377	1.856563	3.270797
68	1	0	5.820867	1.593806	1.288272
69	6	0	3.512027	2.235312	3.516424
70	1	0	5.550962	1.616176	4.008727
71	1	0	2.952826	2.385968	4.428088
72	6	0	4.180645	2.078987	-1.148288
73	1	0	5.038411	2.751419	-1.073107
74	1	0	3.651889	2.250473	-2.084994
75	8	0	4.698042	0.751464	-1.085117
76	6	0	4.115608	-0.182762	-1.863402
77	8	0	3.199774	0.072663	-2.619073
78	6	0	4.731626	-1.561189	-1.657193
79	6	0	4.136073	-2.146159	-0.361531
80	1	0	3.057323	-2.293339	-0.461014
81	1	0	4.593889	-3.121030	-0.165310
82	1	0	4.323755	-1.499237	0.501534
83	6	0	4.356110	-2.452386	-2.844631
84	1	0	4.740301	-2.044814	-3.785092
85	1	0	4.790415	-3.446777	-2.702629
86	1	0	3.272181	-2.565017	-2.934704
87	6	0	6.258575	-1.455314	-1.532282
88	1	0	6.682001	-2.460713	-1.444152
89	1	0	6.699191	-0.978281	-2.413621
90	1	0	6.554462	-0.886419	-0.647396
91	1	0	3.555922	-0.876031	4.370913
92	1	0	3.976937	-3.321278	4.533273
93	1	0	2.767937	-4.882727	3.031363
94	1	0	0.543524	-2.881771	-3.928128
95	1	0	-0.728086	-4.963076	-3.463566
96	1	0	-1.519520	-5.445563	-1.162866
97	1	0	-5.441754	3.123208	1.390928
98	1	0	-4.422959	3.466456	3.627026
99	1	0	-1.993128	3.079592	3.956910
100	1	0	-2.593865	4.234851	-1.237972
101	1	0	-2.249902	5.934717	-2.986382
102	1	0	-0.473345	5.635214	-4.691446
103	1	0	0.976755	3.619177	-4.626974

TS1b(*S*)$_{seg}$

RwB97XD SCF energy -3304.675698 a.u.

RwB97XD SCF enthalpy -3303.804219 a.u.

RwB97XD SCF free energy -3303.949629 a.u.

Three lowest frequencies (cm^{-1}) -600.5, 13.4, 15.7

Imaginary frequency (cm^{-1}) -600.5

Cartesian coordinates:

Center Number	Atomic Number	Atomic Type	Coordinates (Angstroms) X	Y	Z
1	6	0	-2.445148	0.709042	-1.055839
2	6	0	-3.573729	0.394979	-1.780415
3	6	0	-3.537636	0.030884	-3.122392
4	6	0	-2.361427	0.000763	-3.830580
5	6	0	-1.197613	0.345936	-3.127723
6	6	0	-1.218882	0.690666	-1.777564
7	1	0	-2.330120	-0.274428	-4.878082
8	1	0	-0.257900	0.334639	-3.667693
9	6	0	-2.590118	1.020255	0.393005
10	6	0	-2.194523	0.149541	1.445476
11	6	0	-3.174996	2.205454	0.778437
12	6	0	-2.386101	0.512574	2.775826
13	6	0	-3.340153	2.568810	2.110909
14	6	0	-2.962197	1.739843	3.138495
15	1	0	-2.083227	-0.161697	3.568475
16	1	0	-3.100946	2.015263	4.177200
17	8	0	-4.790752	-0.267100	-3.547923
18	8	0	-4.852772	0.344859	-1.325017
19	8	0	-3.639063	3.194478	-0.029350
20	8	0	-3.906585	3.800949	2.180413
21	6	0	-5.662776	-0.061962	-2.431722
22	1	0	-6.169227	-0.999705	-2.190228
23	1	0	-6.375942	0.731236	-2.670853
24	6	0	-4.106430	4.236458	0.832324
25	1	0	-5.173011	4.399353	0.657147
26	1	0	-3.527525	5.146362	0.654621
27	15	0	-1.281857	-1.374655	1.024317
28	15	0	0.362923	0.945058	-0.897688
29	46	0	0.899779	-0.936734	0.260160
30	6	0	-2.344269	-2.288279	-0.130330
31	6	0	-3.726257	-2.380957	0.067772
32	6	0	-1.762778	-2.847893	-1.272026
33	6	0	-4.517998	-3.032722	-0.871624
34	1	0	-4.184864	-1.928859	0.942444
35	6	0	-2.562099	-3.485851	-2.216422
36	1	0	-0.689735	-2.764936	-1.428832
37	6	0	-3.938141	-3.575286	-2.017808
38	6	0	-1.155643	-2.326013	2.573000
39	6	0	-2.021209	-3.374128	2.894800
40	6	0	-0.132557	-1.974110	3.464790
41	6	0	-1.868682	-4.055535	4.100425
42	6	0	0.008142	-2.649903	4.671281
43	1	0	0.553274	-1.167637	3.215877
44	6	0	-0.860185	-3.692938	4.989286
45	6	0	1.595639	1.296941	-2.200319
46	6	0	1.909020	2.597998	-2.600542
47	6	0	2.255398	0.216538	-2.797707
48	6	0	2.879812	2.812075	-3.575744
49	1	0	1.424785	3.452395	-2.141587
50	6	0	3.214155	0.433265	-3.781508
51	1	0	2.020482	-0.799143	-2.493167
52	6	0	3.532785	1.733768	-4.166856
53	6	0	0.150368	2.449251	0.096791
54	6	0	0.570035	2.443274	1.429836
55	6	0	-0.467499	3.586466	-0.435991
56	6	0	0.367563	3.566039	2.227693
57	1	0	1.043976	1.558106	1.843728
58	6	0	-0.650584	4.711457	0.360640
59	1	0	-0.827715	3.587633	-1.460619
60	6	0	-0.242345	4.698891	1.693695
61	8	0	1.846981	-2.743110	0.856017
62	6	0	2.914307	-2.161317	0.428734
63	1	0	2.447832	-0.632774	-0.082501
64	6	0	3.476969	-2.549572	-0.875276
65	6	0	4.702040	-2.370981	-1.445004
66	8	0	2.633311	-3.158639	-1.750352
67	6	0	4.609593	-2.919954	-2.759366
68	1	0	5.561832	-1.906564	-0.983673
69	6	0	3.335751	-3.376505	-2.888310
70	1	0	5.387878	-2.967898	-3.506872
71	1	0	2.801858	-3.870562	-3.685943
72	6	0	3.895163	-1.676153	1.495668
73	1	0	4.678230	-2.432036	1.599088
74	1	0	3.351125	-1.576644	2.433773
75	8	0	4.540564	-0.458778	1.148419
76	6	0	4.111234	0.663156	1.762662
77	8	0	3.230735	0.655252	2.598991
78	6	0	4.826425	1.904706	1.251818
79	6	0	4.190589	2.248306	-0.108357
80	1	0	3.122212	2.450281	0.007465
81	1	0	4.663869	3.148368	-0.513378
82	1	0	4.318096	1.438461	-0.833661

83	6	0	4.590355	3.052904	2.236279
84	1	0	5.012335	2.827693	3.220719
85	1	0	5.072735	3.959282	1.858198
86	1	0	3.523506	3.260199	2.358379
87	6	0	6.329329	1.641927	1.084116
88	1	0	6.819486	2.564481	0.758042
89	1	0	6.787763	1.328687	2.027657
90	1	0	6.525280	0.872442	0.333383
91	1	0	3.714737	-0.415440	-4.236697
92	1	0	4.291220	1.905710	-4.924367
93	1	0	3.127403	3.827800	-3.867697
94	1	0	0.682134	3.551214	3.266437
95	1	0	-0.403723	5.573342	2.316435
96	1	0	-1.125158	5.593797	-0.056812
97	1	0	-5.589320	-3.107645	-0.714200
98	1	0	-4.561546	-4.067899	-2.757688
99	1	0	-2.110909	-3.906482	-3.109536
100	1	0	-2.810862	-3.666963	2.210688
101	1	0	-2.543424	-4.870179	4.344189
102	1	0	-0.747162	-4.224738	5.929007
103	1	0	0.798913	-2.366750	5.358772

4b(*S*)$_{seg}$

RwB97XD SCF energy -3304.684477 a.u.
RwB97XD SCF enthalpy -3303.808844 a.u.
RwB97XD SCF free energy -3303.952127 a.u.
Three lowest frequencies (cm^{-1}) 17.9, 22.9, 28.5
Cartesian coordinates:

Center Number	Atomic Number	Atomic Type	Coordinates (Angstroms) X	Y	Z
1	6	0	2.393057	1.317000	-0.424487
2	6	0	2.519935	2.592357	-0.929098
3	6	0	2.576952	2.873371	-2.289289
4	6	0	2.543169	1.875046	-3.231237
5	6	0	2.430830	0.562261	-2.748382
6	6	0	2.353467	0.268727	-1.387872
7	1	0	2.598162	2.084991	-4.292779
8	1	0	2.405365	-0.239387	-3.476865
9	6	0	2.335651	1.154016	1.055367
10	6	0	1.169987	0.824182	1.800265
11	6	0	3.478075	1.387985	1.788713
12	6	0	1.228018	0.729810	3.188251
13	6	0	3.532229	1.254629	3.170808
14	6	0	2.417768	0.933943	3.905627
15	1	0	0.334223	0.490622	3.751622
16	1	0	2.451053	0.842147	4.984895
17	8	0	2.672552	4.214670	-2.475441
18	8	0	2.588473	3.749420	-0.218505
19	8	0	4.702965	1.733921	1.311380
20	8	0	4.799761	1.488008	3.603269
21	6	0	2.624531	4.811189	-1.174774
22	1	0	1.716271	5.414100	-1.084449
23	1	0	3.524682	5.409479	-1.017098
24	6	0	5.565797	1.838342	2.446923
25	1	0	5.917240	2.869870	2.540810
26	1	0	6.398233	1.139163	2.337283
27	15	0	-0.343298	0.367172	0.869409
28	15	0	2.073112	-1.463526	-0.855896
29	46	0	-0.041853	-1.793781	-0.161338
30	6	0	-0.667032	1.809575	-0.194268
31	6	0	-0.674373	3.107924	0.327911
32	6	0	-0.817440	1.616255	-1.570156
33	6	0	-0.841187	4.198192	-0.520056
34	1	0	-0.535323	3.267756	1.393558
35	6	0	-0.962897	2.709724	-2.418490
36	1	0	-0.778027	0.612948	-1.983728
37	6	0	-0.974576	4.000112	-1.893987
38	6	0	-1.684754	0.282029	2.105661
39	6	0	-2.738911	1.195940	2.159423
40	6	0	-1.676176	-0.804753	2.992688
41	6	0	-3.756489	1.038523	3.099905
42	1	0	-2.775521	2.035974	1.474957
43	6	0	-2.682085	-0.947816	3.940814
44	1	0	-0.880266	-1.543629	2.940727
45	6	0	-3.727458	-0.026130	3.994111
46	6	0	2.542958	-2.478098	-2.302991
47	6	0	3.824081	-3.009796	-2.464011
48	6	0	1.573836	-2.705528	-3.288604
49	6	0	4.130116	-3.759573	-3.598258
50	1	0	4.587235	-2.859405	-1.708575
51	6	0	1.888535	-3.438881	-4.426707
52	1	0	0.570376	-2.306938	-3.163701
53	6	0	3.168076	-3.970322	-4.581269
54	6	0	3.303259	-1.754416	0.453645
55	6	0	2.910826	-2.373457	1.644280
56	6	0	4.618591	-1.298671	0.307642
57	6	0	3.825088	-2.526706	2.682877
58	1	0	1.885677	-2.714717	1.764541
59	6	0	5.532765	-1.470930	1.340971
60	1	0	4.924419	-0.787028	-0.600226
61	6	0	5.134174	-2.074868	2.532302
62	8	0	-2.042352	-2.446771	0.401301
63	6	0	-3.120350	-1.998289	-0.020138
64	1	0	0.263531	-3.221542	-0.724236
65	6	0	-4.327823	-2.249962	0.712371
66	6	0	-4.555347	-2.861154	1.921364
67	8	0	-5.512086	-1.821919	0.181052
68	6	0	-5.951269	-2.795427	2.142158
69	1	0	-3.803033	-3.296044	2.562723
70	6	0	-6.473538	-2.155993	1.053187
71	1	0	-6.502421	-3.171015	2.991277
72	1	0	-7.482393	-1.886826	0.776828
73	6	0	-3.196527	-1.207569	-1.307188
74	1	0	-3.785746	-1.767812	-2.039979
75	1	0	-2.188325	-1.054169	-1.697983
76	8	0	-3.797611	0.052625	-1.037323
77	6	0	-4.819508	0.445762	-1.828605
78	8	0	-5.180799	-0.195095	-2.790798
79	6	0	-5.436010	1.749840	-1.342081
80	6	0	-6.066609	1.492504	0.038293
81	1	0	-5.321284	1.149946	0.760496
82	1	0	-6.507581	2.422025	0.412259
83	1	0	-6.859096	0.740102	-0.024077
84	6	0	-4.342532	2.824402	-1.232889
85	1	0	-3.875290	3.007941	-2.204963
86	1	0	-4.790884	3.761348	-0.887741
87	1	0	-3.561189	2.536835	-0.525744
88	6	0	-6.509820	2.194406	-2.337263
89	1	0	-6.967114	3.124401	-1.986063
90	1	0	-6.082807	2.375914	-3.328239
91	1	0	-7.298160	1.442823	-2.437347
92	1	0	1.131834	-3.603265	-5.187388
93	1	0	3.411602	-4.552156	-5.464960
94	1	0	5.124675	-4.180059	-3.709351
95	1	0	6.552069	-1.117690	1.221124
96	1	0	5.845613	-2.190011	3.344232
97	1	0	3.511619	-2.993561	3.611225
98	1	0	-1.061533	2.553271	-3.487942
99	1	0	-1.085329	4.853162	-2.556322
100	1	0	-0.850892	5.203061	-0.109768
101	1	0	-4.568975	1.757905	3.130666
102	1	0	-4.519033	-0.143200	4.727797
103	1	0	-2.657229	-1.785205	4.631113

5b(*S*)$_{seg}$

RwB97XD SCF energy -3305.860132 a.u.
RwB97XD SCF enthalpy -3304.965278 a.u.
RwB97XD SCF free energy -3305.112224 a.u.
Three lowest frequencies (cm^{-1}) 7.8, .9,7 26.2
Cartesian coordinates:

Center Number	Atomic Number	Atomic Type	Coordinates X	Y	(Angstroms) Z
1	6	0	3.045898	-0.261908	0.945545
2	6	0	3.667164	-0.929924	1.975843
3	6	0	3.700609	-0.446730	3.280439
4	6	0	3.136271	0.758612	3.619919
5	6	0	2.500430	1.465859	2.588517
6	6	0	2.442129	0.978904	1.285631
7	1	0	3.175693	1.144881	4.631346
8	1	0	2.045627	2.418436	2.832307
9	6	0	3.088391	-0.838068	-0.425687
10	6	0	1.979296	-1.359202	-1.150533
11	6	0	4.304169	-0.883043	-1.070935
12	6	0	2.157724	-1.878508	-2.431570
13	6	0	4.463650	-1.373546	-2.362217
14	6	0	3.406905	-1.892947	-3.069056
15	1	0	1.316167	-2.295978	-2.969249
16	1	0	3.527429	-2.295236	-4.067791

17	8	0	4.362667	-1.325687	4.073511
18	8	0	4.315420	-2.121533	1.910411
19	8	0	5.498645	-0.448245	-0.593770
20	8	0	5.760047	-1.251266	-2.739733
21	6	0	4.697499	-2.447731	3.249902
22	1	0	4.138097	-3.326067	3.585516
23	1	0	5.775445	-2.617707	3.287912
24	6	0	6.452804	-0.650538	-1.639666
25	1	0	7.234822	-1.328932	-1.289970
26	1	0	6.863527	0.313163	-1.952046
27	15	0	0.301085	-1.227321	-0.439683
28	15	0	1.497877	1.862176	0.004917
29	46	0	-0.522798	0.900691	-0.633734
30	6	0	0.468238	-1.826391	1.265876
31	6	0	1.102572	-3.052253	1.499053
32	6	0	0.028946	-1.051162	2.340598
33	6	0	1.279327	-3.503322	2.802299
34	1	0	1.467986	-3.648156	0.667518
35	6	0	0.221382	-1.500501	3.643215
36	1	0	-0.444421	-0.091496	2.159583
37	6	0	0.844384	-2.725368	3.874234
38	6	0	-0.758253	-2.389891	-1.350649
39	6	0	-1.281567	-3.535916	-0.750351
40	6	0	-1.100210	-2.084451	-2.675984
41	6	0	-2.121360	-4.378877	-1.476150
42	1	0	-1.058877	-3.773058	0.283404
43	6	0	-1.921546	-2.937778	-3.399631
44	1	0	-0.731444	-1.173729	-3.138303
45	6	0	-2.435655	-4.086866	-2.798622
46	6	0	1.108005	3.499624	0.698136
47	6	0	1.780518	4.656799	0.300763
48	6	0	0.071587	3.585379	1.638401
49	6	0	1.424709	5.887896	0.848613
50	1	0	2.575539	4.608729	-0.435987
51	6	0	-0.270735	4.814347	2.189452
52	1	0	-0.467226	2.690721	1.941770
53	6	0	0.405869	5.967734	1.793149
54	6	0	2.660562	2.083280	-1.371398
55	6	0	2.241502	1.814543	-2.677276
56	6	0	3.990338	2.441001	-1.121260
57	6	0	3.154033	1.885185	-3.726113
58	1	0	1.210088	1.529337	-2.868663
59	6	0	4.893955	2.521877	-2.174641
60	1	0	4.323673	2.635934	-0.106062
61	6	0	4.479549	2.231651	-3.474045
62	8	0	-2.315389	0.192592	-1.264010
63	6	0	-3.390860	0.333999	-0.379259
64	6	0	-3.668071	-0.942438	0.362162
65	6	0	-4.538592	-1.974144	0.191633
66	8	0	-2.849316	-1.206137	1.416663
67	6	0	-4.232309	-2.937509	1.209362
68	1	0	-5.309982	-2.041011	-0.562893
69	6	0	-3.196033	-2.421526	1.916060
70	1	0	-4.718731	-3.887122	1.381597
71	1	0	-2.619883	-2.768110	2.761056
72	6	0	-4.606337	0.745137	-1.205719
73	1	0	-4.861986	-0.032074	-1.930863
74	1	0	-4.393921	1.674354	-1.741396
75	8	0	-5.698173	0.954799	-0.301487
76	6	0	-6.937359	0.902393	-0.798646
77	8	0	-7.163776	0.772744	-1.986147
78	6	0	-7.992814	0.980991	0.298784
79	6	0	-7.687924	2.157335	1.239455
80	1	0	-7.667437	3.106469	0.693541
81	1	0	-8.468071	2.222279	2.004539
82	1	0	-6.726841	2.029479	1.744089
83	6	0	-9.371107	1.157516	-0.341352
84	1	0	-9.603279	0.334955	-1.023436
85	1	0	-10.137199	1.180118	0.439775
86	1	0	-9.430877	2.093789	-0.905060
87	6	0	-7.938852	-0.345060	1.082084
88	1	0	-8.704009	-0.335046	1.864827
89	1	0	-8.135933	-1.199672	0.426192
90	1	0	-6.963429	-0.490227	1.555598
91	1	0	-1.462170	2.596491	-0.628639
92	1	0	-1.079805	2.632135	-1.298809
93	1	0	-3.209529	1.124997	0.369283
94	1	0	-1.067976	4.872224	2.923653
95	1	0	0.134597	6.928505	2.219384
96	1	0	1.948504	6.784997	0.534090
97	1	0	5.926006	2.795147	-1.979296
98	1	0	5.193613	2.274779	-4.290662
99	1	0	2.831943	1.659704	-4.737740
100	1	0	-2.172710	-2.699174	-4.428197
101	1	0	-3.087477	-4.747390	-3.362080
102	1	0	-2.531223	-5.263262	-0.998772
103	1	0	-0.107658	-0.888974	4.477317
104	1	0	0.996436	-3.072354	4.891622
105	1	0	1.767194	-4.456376	2.979557

TS2b(*S*)$_{seg}$

RwB97XD SCF energy -3305.848518 a.u.
RwB97XD SCF enthalpy -3304.957271 a.u.
RwB97XD SCF free energy -3305.101713 a.u.
Three lowest frequencies (cm^{-1}) -1096, -104.2, 17.1
Imaginary frequency (cm^{-1}) -1096
Standard orientation:

Center Number	Atomic Number	Atomic Type	Coordinates (Angstroms) X	Y	Z
1	6	0	2.922746	-0.366406	1.062592
2	6	0	3.398780	-0.985277	2.195791
3	6	0	3.342086	-0.401049	3.457963
4	6	0	2.832417	0.859868	3.650814
5	6	0	2.352742	1.522623	2.511642
6	6	0	2.386558	0.937858	1.248169
7	1	0	2.793846	1.318214	4.631607
8	1	0	1.940941	2.517119	2.637334
9	6	0	3.006782	-1.071607	-0.245059
10	6	0	1.895327	-1.562145	-0.985345
11	6	0	4.244744	-1.277514	-0.811918
12	6	0	2.084281	-2.193205	-2.212481
13	6	0	4.419953	-1.884612	-2.051188
14	6	0	3.357024	-2.363528	-2.777293
15	1	0	1.230496	-2.570326	-2.762961
16	1	0	3.490989	-2.846387	-3.737885
17	8	0	3.845292	-1.258764	4.379334
18	8	0	3.944824	-2.224906	2.287231
19	8	0	5.448530	-0.896696	-0.311995
20	8	0	5.738773	-1.897448	-2.369557
21	6	0	4.224449	-2.444740	3.672476
22	1	0	3.632092	-3.289061	4.034665
23	1	0	5.295342	-2.617415	3.804216
24	6	0	6.433160	-1.318565	-1.260155
25	1	0	7.077989	-2.073118	-0.800947
26	1	0	7.004792	-0.452736	-1.601966
27	15	0	0.204979	-1.214274	-0.392639
28	15	0	1.625987	1.793438	-0.165448
29	46	0	-0.396963	0.979999	-0.845573
30	6	0	0.189166	-1.703068	1.354169
31	6	0	0.750998	-2.920251	1.759772
32	6	0	-0.327668	-0.821795	2.305077
33	6	0	0.787462	-3.248615	3.110079
34	1	0	1.176837	-3.600124	1.027368
35	6	0	-0.273455	-1.148381	3.657200
36	1	0	-0.755061	0.125648	1.989403
37	6	0	0.285656	-2.358349	4.059298
38	6	0	-0.909276	-2.321160	-1.309365
39	6	0	-1.427176	-3.491197	-0.746681
40	6	0	-1.281911	-1.952910	-2.608273
41	6	0	-2.302782	-4.285446	-1.480790
42	1	0	-1.170403	-3.779428	0.266434
43	6	0	-2.155872	-2.751532	-3.338050
44	1	0	-0.893329	-1.039033	-3.047092
45	6	0	-2.669364	-3.917194	-2.773365
46	6	0	1.386152	3.518972	0.369049
47	6	0	2.353506	4.503134	0.149731
48	6	0	0.207122	3.844953	1.051900
49	6	0	2.141021	5.799647	0.611715
50	1	0	3.269926	4.272357	-0.382735
51	6	0	0.005109	5.138725	1.518941
52	1	0	-0.554759	3.087319	1.216309
53	6	0	0.972151	6.117858	1.297728
54	6	0	2.869910	1.757075	-1.487960
55	6	0	2.482413	1.441711	-2.793510
56	6	0	4.220446	1.979247	-1.193497
57	6	0	3.442999	1.339061	-3.795570
58	1	0	1.435664	1.254402	-3.019253
59	6	0	5.172872	1.890387	-2.202262
60	1	0	4.532070	2.195325	-0.175743
61	6	0	4.786205	1.561112	-3.500551
62	8	0	-2.358314	0.817402	-1.708638

63	6	0	-3.460352	1.010563	-0.849673
64	6	0	-3.562665	-0.013370	0.238513
65	6	0	-3.803952	-1.351600	0.244958
66	8	0	-3.430335	0.435173	1.521750
67	6	0	-3.813668	-1.758629	1.617573
68	1	0	-3.965760	-1.969430	-0.626684
69	6	0	-3.588123	-0.633922	2.343298
70	1	0	-3.970613	-2.754013	2.007886
71	1	0	-3.510955	-0.434263	3.401070
72	6	0	-4.703919	0.985774	-1.743754
73	1	0	-4.781619	0.030808	-2.266446
74	1	0	-4.661734	1.799476	-2.470254
75	8	0	-5.857657	1.204327	-0.926391
76	6	0	-6.733552	0.200981	-0.757081
77	8	0	-6.696527	-0.832039	-1.394264
78	6	0	-7.740225	0.522478	0.340831
79	6	0	-6.966191	0.581402	1.671965
80	1	0	-6.209369	1.370135	1.659563
81	1	0	-7.665085	0.786899	2.489317
82	1	0	-6.469347	-0.372033	1.879777
83	6	0	-8.403538	1.879121	0.055498
84	1	0	-8.925717	1.870026	-0.907029
85	1	0	-9.138052	2.097296	0.837251
86	1	0	-7.668543	2.687762	0.041658
87	6	0	-8.794635	-0.584084	0.397818
88	1	0	-9.516138	-0.361910	1.190138
89	1	0	-9.340034	-0.665186	-0.547475
90	1	0	-8.339700	-1.555403	0.612114
91	1	0	-0.912080	2.576076	-1.398363
92	1	0	-1.609060	2.029682	-1.654278
93	1	0	-3.407829	1.993194	-0.355082
94	1	0	-0.910092	5.382221	2.049196
95	1	0	0.812040	7.129536	1.657367
96	1	0	2.893952	6.560816	0.433701
97	1	0	6.219013	2.064365	-1.971612
98	1	0	5.534588	1.475054	-4.282233
99	1	0	3.142285	1.078753	-4.805454
100	1	0	-2.705059	-5.190374	-1.036525
101	1	0	-3.356039	-4.537664	-3.340907
102	1	0	-2.439055	-2.459581	-4.344387
103	1	0	1.221819	-4.192752	3.423036
104	1	0	0.334810	-2.608071	5.114693
105	1	0	-0.660416	-0.452350	4.394673

6a(*S*)$_{seg}$•2b

RwB97XD SCF energy -3304.999750 a.u.
RwB97XD SCF enthalpy -3304.999750 a.u.
RwB97XD SCF free energy -3305.145827 a.u.
Three lowest frequencies (cm^{-1}) 14.2, 16.8,25.7
Standard orientation:

Center Number	Atomic Number	Atomic Type	Coordinates (Angstroms) X	Y	Z
1	6	0	2.888956	-0.247728	1.047267
2	6	0	3.373571	-0.835853	2.194760
3	6	0	3.289632	-0.241991	3.449142
4	6	0	2.736486	1.002877	3.620867
5	6	0	2.251804	1.637223	2.467301
6	6	0	2.315114	1.045631	1.206963
7	1	0	2.670835	1.472286	4.595087
8	1	0	1.811055	2.620338	2.581703
9	6	0	3.014505	-0.997507	-0.233993
10	6	0	1.937761	-1.618841	-0.925520
11	6	0	4.265389	-1.163105	-0.787411
12	6	0	2.172462	-2.320708	-2.104887
13	6	0	4.482689	-1.840305	-1.981532
14	6	0	3.454229	-2.439517	-2.665877
15	1	0	1.349588	-2.800952	-2.621081
16	1	0	3.619737	-2.979023	-3.591021
17	8	0	3.814333	-1.073225	4.384839
18	8	0	3.961745	-2.056663	2.303628
19	8	0	5.444352	-0.685851	-0.308111
20	8	0	5.804081	-1.795615	-2.298141
21	6	0	4.248331	-2.247392	3.691280
22	1	0	3.694599	-3.114143	4.061946
23	1	0	5.325713	-2.371914	3.826840
24	6	0	6.456484	-1.112561	-1.223888
25	1	0	7.138145	-1.799937	-0.714658
26	1	0	6.984230	-0.240201	-1.616474
27	15	0	0.230704	-1.316751	-0.324790
28	15	0	1.550549	1.898677	-0.223085
29	46	0	-0.373066	0.949451	-0.896098
30	6	0	0.261825	-1.832424	1.420784
31	6	0	0.838410	-3.045658	1.814371
32	6	0	-0.230664	-0.953281	2.388510
33	6	0	0.909171	-3.376491	3.163599
34	1	0	1.244025	-3.724792	1.069572
35	6	0	-0.146237	-1.281225	3.738829
36	1	0	-0.660598	-0.003349	2.085906
37	6	0	0.424518	-2.491392	4.126282
38	6	0	-0.833061	-2.500931	-1.220487
39	6	0	-1.426491	-3.610513	-0.616891
40	6	0	-1.126527	-2.213514	-2.564056
41	6	0	-2.299493	-4.418326	-1.344438
42	1	0	-1.230010	-3.842665	0.423944
43	6	0	-1.995138	-3.023614	-3.287016
44	1	0	-0.665145	-1.353853	-3.047872
45	6	0	-2.587062	-4.127606	-2.674030
46	6	0	1.354681	3.628135	0.334784
47	6	0	2.358364	4.587001	0.178885
48	6	0	0.164928	3.975455	0.986794
49	6	0	2.171236	5.877128	0.670390
50	1	0	3.284335	4.344912	-0.330997
51	6	0	-0.010785	5.259406	1.490326
52	1	0	-0.624343	3.236597	1.101049
53	6	0	0.992659	6.213507	1.330199
54	6	0	2.826065	1.853697	-1.521561
55	6	0	2.469830	1.499689	-2.826681
56	6	0	4.169544	2.094922	-1.210775
57	6	0	3.449248	1.378888	-3.808061
58	1	0	1.428788	1.298260	-3.066143
59	6	0	5.141577	1.993550	-2.199912
60	1	0	4.462843	2.333490	-0.192556
61	6	0	4.784452	1.624996	-3.495663
62	8	0	-2.377428	0.531445	-1.723650
63	6	0	-3.496337	0.716002	-0.834486
64	6	0	-3.746656	-0.483418	0.009160
65	6	0	-4.429632	-1.643699	-0.187541
66	8	0	-3.171761	-0.486981	1.243792
67	6	0	-4.266540	-2.413010	1.008294
68	1	0	-4.988505	-1.914269	-1.071741
69	6	0	-3.495686	-1.663012	1.836156
70	1	0	-4.669987	-3.393367	1.215911
71	1	0	-3.105467	-1.814442	2.831145
72	6	0	-4.713311	1.104601	-1.669394
73	1	0	-5.023154	0.294673	-2.333104
74	1	0	-4.490772	1.995842	-2.258451
75	8	0	-5.754869	1.437437	-0.752006
76	6	0	-6.858427	0.670761	-0.711761
77	8	0	-7.108829	-0.173524	-1.546785
78	6	0	-7.701165	0.968399	0.521818
79	6	0	-7.854877	2.483340	0.718984
80	1	0	-8.336265	2.948816	-0.147428
81	1	0	-8.481277	2.671725	1.596549
82	1	0	-6.889799	2.970478	0.878738
83	6	0	-9.075021	0.314342	0.359765
84	1	0	-8.989811	-0.767326	0.225809
85	1	0	-9.676670	0.502906	1.254145
86	1	0	-9.608923	0.724481	-0.503116
87	6	0	-6.958103	0.350135	1.724261
88	1	0	-7.541783	0.516381	2.635309
89	1	0	-6.824743	-0.728667	1.594621
90	1	0	-5.972280	0.804690	1.862045
91	1	0	-0.600440	2.424739	-1.361638
92	1	0	-2.447084	-0.343568	-2.135782
93	1	0	-3.219264	1.554428	-0.191396
94	1	0	3.169335	1.088701	-4.815921
95	1	0	5.548022	1.527500	-4.261247
96	1	0	6.181216	2.185425	-1.954183
97	1	0	2.952146	6.619181	0.536554
98	1	0	0.853246	7.218548	1.716356
99	1	0	-0.934303	5.515781	1.999877
100	1	0	-2.760741	-5.274762	-0.862557
101	1	0	-3.270271	-4.758069	-3.234471
102	1	0	-2.210311	-2.792011	-4.325411
103	1	0	1.354817	-4.319328	3.464827
104	1	0	0.497915	-2.743693	5.179743
105	1	0	-0.515633	-0.586035	4.486245

2.9. **Catalytic cycle 2b–1a (*R*)**

3a(*R*)$_{seg}$

RwB97XD SCF energy	-3304.688615 a.u.
RwB97XD SCF enthalpy	-3303.814381 a.u.
RwB97XD SCF free energy	-3303.956798 a.u.
Three lowest frequencies (cm^{-1})	23.9, 27.1 28.8

Cartesian coordinates:

Center Number	Atomic Number	Atomic Type	Coordinates (Angstroms) X	Y	Z
1	6	0	2.393057	1.317000	-0.424487
2	6	0	2.519935	2.592357	-0.929098
3	6	0	2.576952	2.873371	-2.289289
4	6	0	2.543169	1.875046	-3.231237
5	6	0	2.430830	0.562261	-2.748382
6	6	0	2.353467	0.268727	-1.387872
7	1	0	2.598162	2.084991	-4.292779
8	1	0	2.405365	-0.239387	-3.476865
9	6	0	2.335651	1.154016	1.055367
10	6	0	1.169987	0.824182	1.800265
11	6	0	3.478075	1.387985	1.788713
12	6	0	1.228018	0.729810	3.188251
13	6	0	3.532229	1.254629	3.170808
14	6	0	2.417768	0.933943	3.905627
15	1	0	0.334223	0.490622	3.751622
16	1	0	2.451053	0.842147	4.984895
17	8	0	2.672552	4.214670	-2.475441
18	8	0	2.588473	3.749420	-0.218505
19	8	0	4.702965	1.733921	1.311380
20	8	0	4.799761	1.488008	3.603269
21	6	0	2.624531	4.811189	-1.174774
22	1	0	1.716271	5.414100	-1.084449
23	1	0	3.524682	5.409479	-1.017098
24	6	0	5.565797	1.838342	2.446923
25	1	0	5.917240	2.869870	2.540810
26	1	0	6.398233	1.139163	2.337283
27	15	0	-0.343298	0.367172	0.869409
28	15	0	2.073112	-1.463526	-0.855896
29	46	0	-0.041853	-1.793781	-0.161338
30	6	0	-0.667032	1.809575	-0.194268
31	6	0	-0.674373	3.107924	0.327911
32	6	0	-0.817440	1.616255	-1.570156
33	6	0	-0.841187	4.198192	-0.520056
34	1	0	-0.535323	3.267756	1.393558
35	6	0	-0.962897	2.709724	-2.418490
36	1	0	-0.778027	0.612948	-1.983728
37	6	0	-0.974576	4.000112	-1.893987
38	6	0	-1.684754	0.282029	2.105661
39	6	0	-2.738911	1.195940	2.159423
40	6	0	-1.676176	-0.804753	2.992688
41	6	0	-3.756489	1.038523	3.099905
42	1	0	-2.775521	2.035974	1.474957
43	6	0	-2.682085	-0.947816	3.940814
44	1	0	-0.880266	-1.543629	2.940727
45	6	0	-3.727458	-0.026130	3.994111
46	6	0	2.542958	-2.478098	-2.302991
47	6	0	3.824081	-3.009796	-2.464011
48	6	0	1.573836	-2.705528	-3.288604
49	6	0	4.130116	-3.759573	-3.598258
50	1	0	4.587235	-2.859405	-1.708575
51	6	0	1.888535	-3.438881	-4.426707
52	1	0	0.570376	-2.306938	-3.163701
53	6	0	3.168076	-3.970322	-4.581269
54	6	0	3.303259	-1.754416	0.453645
55	6	0	2.910826	-2.373457	1.644280
56	6	0	4.618591	-1.298671	0.307642
57	6	0	3.825088	-2.526706	2.682877
58	1	0	1.885677	-2.714717	1.764541
59	6	0	5.532765	-1.470930	1.340971
60	1	0	4.924419	-0.787028	-0.600226
61	6	0	5.134174	-2.074868	2.532302
62	8	0	-2.042352	-2.446771	0.401301
63	6	0	-3.120350	-1.998289	-0.020138
64	1	0	0.263531	-3.221542	-0.724236
65	6	0	-4.327823	-2.249962	0.712371
66	6	0	-4.555347	-2.861154	1.921364
67	8	0	-5.512086	-1.821919	0.181052
68	6	0	-5.951269	-2.795427	2.142158
69	1	0	-3.803033	-3.296044	2.562723
70	6	0	-6.473538	-2.155993	1.053187
71	1	0	-6.502421	-3.171015	2.991277
72	1	0	-7.482393	-1.886826	0.776828
73	6	0	-3.196527	-1.207569	-1.307188
74	1	0	-3.785746	-1.767812	-2.039979
75	1	0	-2.188325	-1.054169	-1.697983
76	8	0	-3.797611	0.052625	-1.037323
77	6	0	-4.819508	0.445762	-1.828605
78	8	0	-5.180799	-0.195095	-2.790798
79	6	0	-5.436010	1.749840	-1.342081
80	6	0	-6.066609	1.492504	0.038293
81	1	0	-5.321284	1.149946	0.760496
82	1	0	-6.507581	2.422025	0.412259
83	1	0	-6.859096	0.740102	-0.024077
84	6	0	-4.342532	2.824402	-1.232889
85	1	0	-3.875290	3.007941	-2.204963
86	1	0	-4.790884	3.761348	-0.887741
87	1	0	-3.561189	2.536835	-0.525744
88	6	0	-6.509820	2.194406	-2.337263
89	1	0	-6.967114	3.124401	-1.986063
90	1	0	-6.082807	2.375914	-3.328239
91	1	0	-7.298160	1.442823	-2.437347
92	1	0	1.131834	-3.603265	-5.187388
93	1	0	3.411602	-4.552156	-5.464960
94	1	0	5.124675	-4.180059	-3.709351
95	1	0	6.552069	-1.117690	1.221124
96	1	0	5.845613	-2.190011	3.344232
97	1	0	3.511619	-2.993561	3.611225
98	1	0	-1.061533	2.553271	-3.487942
99	1	0	-1.085329	4.853162	-2.556322
100	1	0	-0.850892	5.203061	-0.109768
101	1	0	-4.568975	1.757905	3.130666
102	1	0	-4.519033	-0.143200	4.727797
103	1	0	-2.657229	-1.785205	4.631113

TS1a(*R*)$_{seg}$

RwB97XD SCF energy	-3304.668411 a.u.
RwB97XD SCF enthalpy	-3303.796755 a.u.
RwB97XD SCF free energy	-3303.939552 a.u.
Three lowest frequencies (cm^{-1})	-590.9, 13.7, 24.2
Imaginary frequency (cm^{-1})	-500.9

Cartesian coordinates:

Center Number	Atomic Number	Atomic Type	Coordinates (Angstroms) X	Y	Z
1	6	0	2.717518	-0.537071	0.496526
2	6	0	3.650791	-1.447198	0.939117
3	6	0	3.891023	-1.697200	2.286462
4	6	0	3.225199	-1.018152	3.277447
5	6	0	2.277882	-0.072136	2.858912
6	6	0	2.019793	0.173849	1.512340
7	1	0	3.411424	-1.208395	4.327629
8	1	0	1.733290	0.473102	3.621339
9	6	0	2.530092	-0.366696	-0.972960
10	6	0	1.417542	-0.838698	-1.727226
11	6	0	3.524769	0.256070	-1.694593
12	6	0	1.363985	-0.642696	-3.104565
13	6	0	3.444493	0.477011	-3.065101
14	6	0	2.378176	0.029417	-3.805401
15	1	0	0.518630	-1.015790	-3.670820
16	1	0	2.318644	0.184751	-4.876118
17	8	0	4.826972	-2.671573	2.409408
18	8	0	4.433624	-2.252727	0.175563
19	8	0	4.684354	0.773495	-1.212371
20	8	0	4.542310	1.161385	-3.480947
21	6	0	5.201495	-3.050444	1.080984
22	1	0	4.969401	-4.106837	0.923886
23	1	0	6.264736	-2.846280	0.930156
24	6	0	5.391945	1.299567	-2.338480
25	1	0	6.308364	0.721985	-2.491588
26	1	0	5.605252	2.357720	-2.171721
27	15	0	-0.036494	-1.482325	-0.820528
28	15	0	0.684767	1.322039	1.050809
29	46	0	-1.134379	0.408699	0.043071
30	6	0	0.621668	-2.737085	0.318842
31	6	0	1.504506	-3.716757	-0.151328
32	6	0	0.276041	-2.703141	1.671233
33	6	0	2.029456	-4.657071	0.727954
34	1	0	1.789253	-3.737761	-1.199539
35	6	0	0.810272	-3.642031	2.549676

36	1	0	-0.386960	-1.927399	2.042431
37	6	0	1.687291	-4.616267	2.079320
38	6	0	-1.121208	-2.377057	-1.984140
39	6	0	-1.347424	-3.752806	-1.875124
40	6	0	-1.841964	-1.637258	-2.932360
41	6	0	-2.268999	-4.377995	-2.711439
42	1	0	-0.819160	-4.344337	-1.135778
43	6	0	-2.751912	-2.268325	-3.772921
44	1	0	-1.702518	-0.563401	-3.003990
45	6	0	-2.970078	-3.640258	-3.660779
46	6	0	0.255248	2.219918	2.577802
47	6	0	1.069358	3.241306	3.077363
48	6	0	-0.901249	1.852365	3.274666
49	6	0	0.722919	3.891505	4.257448
50	1	0	1.971589	3.537000	2.552763
51	6	0	-1.239614	2.501017	4.458792
52	1	0	-1.540084	1.062404	2.887538
53	6	0	-0.429197	3.522306	4.948849
54	6	0	1.446339	2.506749	-0.107310
55	6	0	0.716082	2.942356	-1.218210
56	6	0	2.762962	2.945236	0.070894
57	6	0	1.298922	3.799840	-2.145736
58	1	0	-0.302469	2.596505	-1.371058
59	6	0	3.337545	3.812183	-0.853220
60	1	0	3.351120	2.591624	0.912471
61	6	0	2.611153	4.231599	-1.966161
62	8	0	-3.029251	0.401481	-0.969098
63	6	0	-3.189183	1.529341	-0.375677
64	1	0	-1.843147	1.760595	0.546812
65	6	0	-3.006029	2.758105	-1.164693
66	6	0	-2.793391	2.953438	-2.495517
67	8	0	-2.965848	3.941470	-0.496346
68	6	0	-2.612900	4.360408	-2.660297
69	1	0	-2.773217	2.188441	-3.258115
70	6	0	-2.721435	4.903864	-1.419275
71	1	0	-2.428950	4.893688	-3.581513
72	1	0	-2.660553	5.913318	-1.041737
73	6	0	-4.159948	1.578857	0.812690
74	1	0	-5.142256	1.869841	0.429462
75	1	0	-3.842489	2.283762	1.579023
76	8	0	-4.197297	0.290137	1.397354
77	6	0	-4.945155	-0.629431	0.742918
78	8	0	-5.812441	-0.314922	-0.044003
79	6	0	-4.622379	-2.069485	1.121026
80	6	0	-4.659465	-2.895267	-0.175604
81	1	0	-3.906184	-2.539638	-0.886467
82	1	0	-4.449018	-3.944793	0.051744
83	1	0	-5.638998	-2.834952	-0.656448
84	6	0	-3.242513	-2.200630	1.769114
85	1	0	-3.176348	-1.659568	2.717039
86	1	0	-3.031536	-3.256995	1.964412
87	1	0	-2.466579	-1.817664	1.098351
88	6	0	-5.722046	-2.547186	2.086907
89	1	0	-5.556581	-3.600445	2.334301
90	1	0	-5.711931	-1.970565	3.017730
91	1	0	-6.712309	-2.451896	1.631375
92	1	0	-2.139322	2.211651	4.992661
93	1	0	-0.696252	4.032987	5.868892
94	1	0	1.355408	4.687962	4.636475
95	1	0	4.359653	4.148159	-0.709611
96	1	0	3.068574	4.895721	-2.693026
97	1	0	0.727959	4.124015	-3.010305
98	1	0	0.551927	-3.602285	3.603168
99	1	0	2.107352	-5.343806	2.767077
100	1	0	2.713530	-5.415570	0.360606
101	1	0	-2.440309	-5.445340	-2.613317
102	1	0	-3.690014	-4.130864	-4.308539
103	1	0	-3.301233	-1.684284	-4.504582

4a(*R*)$_{seg}$

RwB97XD SCF energy -3304.677468 a.u.

RwB97XD SCF enthalpy -3303.802430 a.u.

RwB97XD SCF free energy -3303.947994 a.u.

Three lowest frequencies (cm^{-1}) 11.7, 20.4 25.0

Cartesian coordinates:

Center Number	Atomic Number	Atomic Type	Coordinates (Angstroms) X	Y	Z
1	6	0	2.833441	-1.163488	-0.302064
2	6	0	3.949618	-1.440596	-1.057604
3	6	0	3.920204	-2.272177	-2.173053
4	6	0	2.771285	-2.913071	-2.568637
5	6	0	1.621331	-2.671080	-1.801438
6	6	0	1.636615	-1.819791	-0.700124
7	1	0	2.750393	-3.569776	-3.430220
8	1	0	0.701334	-3.166697	-2.090028
9	6	0	2.945387	-0.237019	0.857677
10	6	0	2.393983	1.074279	0.922257
11	6	0	3.630420	-0.654727	1.975807
12	6	0	2.550127	1.854239	2.066008
13	6	0	3.762432	0.124183	3.121389
14	6	0	3.238765	1.391724	3.197220
15	1	0	2.130164	2.852232	2.102763
16	1	0	3.347164	2.003661	4.084604
17	8	0	5.150176	-2.309475	-2.744162
18	8	0	5.199827	-0.940159	-0.889019
19	8	0	4.235947	-1.854878	2.163742
20	8	0	4.454666	-0.563821	4.062306
21	6	0	6.008295	-1.510983	-1.922184
22	1	0	6.447636	-0.712359	-2.524065
23	1	0	6.775306	-2.147093	-1.471528
24	6	0	4.756236	-1.843652	3.496366
25	1	0	5.839633	-1.981139	3.462855
26	1	0	4.268048	-2.625286	4.084224
27	15	0	1.343417	1.667261	-0.441572
28	15	0	0.089634	-1.377056	0.147088
29	46	0	-0.700965	0.684381	-0.441409
30	6	0	2.285814	1.385153	-1.964477
31	6	0	3.651111	1.690951	-2.013072
32	6	0	1.656895	0.804331	-3.068776
33	6	0	4.380015	1.409641	-3.163171
34	1	0	4.147364	2.128115	-1.151506
35	6	0	2.395561	0.513599	-4.211782
36	1	0	0.597449	0.564628	-3.025525
37	6	0	3.755854	0.811284	-4.256802
38	6	0	1.117082	3.456943	-0.216560
39	6	0	1.803793	4.388384	-0.999472
40	6	0	0.210605	3.899629	0.756953
41	6	0	1.592872	5.750683	-0.799811
42	1	0	2.499812	4.063486	-1.765233
43	6	0	0.011777	5.260223	0.956325
44	1	0	-0.343330	3.181685	1.355636
45	6	0	0.701892	6.187130	0.176195
46	6	0	-1.183951	-2.559421	-0.400138
47	6	0	-1.564710	-3.659299	0.372827
48	6	0	-1.801333	-2.333152	-1.638891
49	6	0	-2.544853	-4.530392	-0.096979
50	1	0	-1.106209	-3.839956	1.339479
51	6	0	-2.769117	-3.214039	-2.107847
52	1	0	-1.521061	-1.469227	-2.238051
53	6	0	-3.142311	-4.313391	-1.335760
54	6	0	0.383732	-1.623844	1.918638
55	6	0	-0.051946	-0.651204	2.823253
56	6	0	1.084962	-2.745065	2.375929
57	6	0	0.226071	-0.793547	4.179577
58	1	0	-0.587700	0.224222	2.464022
59	6	0	1.346386	-2.888630	3.734109
60	1	0	1.441640	-3.491991	1.672817
61	6	0	0.926453	-1.909195	4.633389
62	8	0	-1.892670	2.238296	-1.044037
63	6	0	-2.926555	1.445788	-0.634172
64	6	0	-3.543798	1.911776	0.651367
65	6	0	-3.701913	3.143274	1.201213
66	8	0	-4.088828	0.961985	1.457063
67	6	0	-4.409968	2.939298	2.431608
68	1	0	-3.362693	4.078313	0.779228
69	6	0	-4.620665	1.603552	2.532171
70	1	0	-4.719090	3.692197	3.142377
71	1	0	-5.097512	0.975077	3.268767
72	6	0	-3.928362	1.142947	-1.762642
73	1	0	-3.370064	0.940792	-2.677456
74	1	0	-4.609270	1.983464	-1.909905
75	8	0	-4.647740	-0.053215	-1.463137
76	6	0	-5.833785	0.035275	-0.833010
77	8	0	-6.382311	1.093055	-0.605894
78	6	0	-6.364423	-1.344471	-0.460113
79	6	0	-5.379881	-1.996722	0.528436
80	1	0	-5.325598	-1.431206	1.463420
81	1	0	-5.722224	-3.010111	0.761824
82	1	0	-4.373974	-2.067683	0.105852
83	6	0	-7.739773	-1.192077	0.190914
84	1	0	-8.458136	-0.734696	-0.496027

85	1	0	-8.121127	-2.177901	0.474873
86	1	0	-7.687776	-0.574705	1.092587
87	6	0	-6.469622	-2.200344	-1.733812
88	1	0	-6.873568	-3.184585	-1.476445
89	1	0	-7.140434	-1.739143	-2.466417
90	1	0	-5.492752	-2.344207	-2.201663
91	1	0	-2.559193	0.353122	-0.387157
92	1	0	2.128139	6.469421	-1.412103
93	1	0	0.539355	7.249802	0.327093
94	1	0	-0.690552	5.595464	1.712750
95	1	0	1.910331	0.047585	-5.063382
96	1	0	4.331604	0.575989	-5.146625
97	1	0	5.439031	1.644398	-3.200033
98	1	0	-3.237031	-3.037457	-3.070993
99	1	0	-3.905338	-4.995725	-1.697169
100	1	0	-2.840010	-5.380893	0.509420
101	1	0	-0.099144	-0.031104	4.880097
102	1	0	1.145788	-2.017324	5.691079
103	1	0	1.887384	-3.759798	4.089531

5a(*R*)$_{seg}$

RwB97XD SCF energy -3305.859031 a.u.

RwB97XD SCF enthalpy -3304.964062 a.u.

RwB97XD SCF free energy -3305.107794 a.u.

Three lowest frequencies (cm^{-1}) 15.8, 19.7 28.6

Cartesian coordinates:

Center Number	Atomic Number	Atomic Type	Coordinates (Angstroms) X	Y	Z
1	6	0	2.801525	0.264045	1.268372
2	6	0	3.175909	0.149238	2.587817
3	6	0	2.913737	1.131813	3.536949
4	6	0	2.294306	2.311271	3.202629
5	6	0	1.918825	2.465676	1.859561
6	6	0	2.151605	1.476030	0.907572
7	1	0	2.101743	3.084249	3.936847
8	1	0	1.433881	3.391437	1.574015
9	6	0	3.119705	-0.841432	0.325595
10	6	0	2.168954	-1.712606	-0.274999
11	6	0	4.435643	-1.061082	-0.012640
12	6	0	2.583567	-2.715804	-1.148877
13	6	0	4.835194	-2.046309	-0.909720
14	6	0	3.930997	-2.902496	-1.489162
15	1	0	1.856197	-3.386750	-1.588078
16	1	0	4.238398	-3.679721	-2.178347
17	8	0	3.361253	0.723814	4.750202
18	8	0	3.805142	-0.901127	3.174347
19	8	0	5.518220	-0.363019	0.415793
20	8	0	6.179143	-1.992532	-1.077262
21	6	0	3.934864	-0.574243	4.561109
22	1	0	3.387073	-1.304203	5.162549
23	1	0	4.994378	-0.545282	4.827713
24	6	0	6.653815	-0.928375	-0.245163
25	1	0	7.345901	-1.329651	0.499575
26	1	0	7.128393	-0.166230	-0.868299
27	15	0	0.390817	-1.429269	0.007952
28	15	0	1.487368	1.639456	-0.782753
29	46	0	-0.444845	0.393156	-1.109759
30	6	0	0.197709	-1.284519	1.805978
31	6	0	0.835241	-2.199565	2.652405
32	6	0	-0.534380	-0.225253	2.346871
33	6	0	0.734874	-2.051189	4.030985
34	1	0	1.421123	-3.015047	2.238609
35	6	0	-0.617681	-0.072805	3.727406
36	1	0	-1.018612	0.489452	1.688498
37	6	0	0.018641	-0.982584	4.568628
38	6	0	-0.467568	-2.933648	-0.544677
39	6	0	-1.011642	-3.842363	0.364908
40	6	0	-0.598456	-3.165420	-1.920528
41	6	0	-1.659832	-4.985671	-0.099133
42	1	0	-0.940109	-3.669070	1.432922
43	6	0	-1.241714	-4.308638	-2.376675
44	1	0	-0.208396	-2.445954	-2.634793
45	6	0	-1.771959	-5.221895	-1.465698
46	6	0	1.079202	3.402489	-1.010590
47	6	0	1.874175	4.251650	-1.783981
48	6	0	-0.084171	3.903077	-0.409022
49	6	0	1.514492	5.588414	-1.942237
50	1	0	2.770040	3.882012	-2.270718
51	6	0	-0.432893	5.239647	-0.562481
52	1	0	-0.718386	3.248458	0.183194
53	6	0	0.366979	6.084225	-1.331376
54	6	0	2.862417	1.251136	-1.901545
55	6	0	2.641187	0.404017	-2.990004
56	6	0	4.142917	1.762208	-1.658812
57	6	0	3.700356	0.057069	-3.824330
58	1	0	1.649048	-0.002445	-3.168532
59	6	0	5.193380	1.424366	-2.504710
60	1	0	4.322857	2.408646	-0.804768
61	6	0	4.974315	0.564569	-3.580694
62	8	0	-2.146067	-0.690108	-1.329721
63	6	0	-3.366116	-0.031632	-1.203310
64	6	0	-4.454509	-1.052417	-1.372649
65	6	0	-4.502199	-2.392761	-1.156624
66	8	0	-5.685049	-0.592862	-1.731935
67	6	0	-5.861338	-2.787716	-1.390008
68	1	0	-3.674753	-3.019714	-0.855596
69	6	0	-6.531730	-1.659133	-1.729184
70	1	0	-6.278824	-3.781860	-1.314401
71	1	0	-7.554815	-1.452435	-2.004534
72	6	0	-3.468181	0.649786	0.175368
73	1	0	-2.695454	1.420223	0.254211
74	1	0	-3.340754	-0.088686	0.970008
75	8	0	-4.714678	1.338672	0.323067
76	6	0	-5.721499	0.709856	0.950397
77	8	0	-5.602056	-0.382315	1.468097
78	6	0	-6.992527	1.551778	0.979445
79	6	0	-7.233423	2.225191	-0.379169
80	1	0	-7.301415	1.484468	-1.181512
81	1	0	-8.177611	2.778092	-0.345070
82	1	0	-6.436753	2.929924	-0.629581
83	6	0	-8.173915	0.644502	1.333893
84	1	0	-8.033071	0.164244	2.305912
85	1	0	-9.093876	1.236142	1.374472
86	1	0	-8.305608	-0.141048	0.582223
87	6	0	-6.800143	2.622966	2.070718
88	1	0	-7.706166	3.232822	2.146039
89	1	0	-6.616345	2.163070	3.047283
90	1	0	-5.960972	3.284414	1.834765
91	1	0	-3.512899	0.754323	-1.967201
92	1	0	-1.444467	1.823986	-1.844280
93	1	0	-0.967168	1.616265	-2.426210
94	1	0	2.136166	6.240082	-2.547927
95	1	0	0.092242	7.126832	-1.457485
96	1	0	-1.331852	5.619751	-0.087748
97	1	0	3.530706	-0.616123	-4.658583
98	1	0	5.799780	0.288836	-4.229651
99	1	0	6.184797	1.824929	-2.318599
100	1	0	-1.340368	-4.481258	-3.443587
101	1	0	-2.278424	-6.112752	-1.823958
102	1	0	-2.077268	-5.689424	0.613939
103	1	0	-1.172187	0.761567	4.144684
104	1	0	-0.042828	-0.858315	5.645236
105	1	0	1.227568	-2.763189	4.685356

TS2a(*R*)$_{seg}$

RwB97XD SCF energy -3305.849237 a.u.

RwB97XD SCF enthalpy -3304.956108 a.u.

RwB97XD SCF free energy -3305.099631 a.u.

Three lowest frequencies (cm^{-1}) -1057, 16.3, 21.1

Imaginary frequency (cm^{-1}) -1057

Cartesian coordinates:

Center Number	Atomic Number	Atomic Type	Coordinates (Angstroms) X	Y	Z
1	6	0	2.258682	0.444388	1.475925
2	6	0	2.367301	0.409970	2.847148
3	6	0	1.816372	1.382735	3.675492
4	6	0	1.160974	2.477747	3.167041
5	6	0	1.054661	2.553036	1.770669
6	6	0	1.577367	1.568877	0.934278
7	1	0	0.740237	3.241572	3.809948
8	1	0	0.545191	3.410251	1.347017
9	6	0	2.842265	-0.662491	0.672939
10	6	0	2.088284	-1.620488	-0.058629
11	6	0	4.210976	-0.794889	0.609321
12	6	0	2.730664	-2.613793	-0.794521
13	6	0	4.842329	-1.775586	-0.148577
14	6	0	4.128855	-2.709900	-0.858750
15	1	0	2.147024	-3.341087	-1.346335

16	1	0	4.617651	-3.479931	-1.443575
17	8	0	2.032325	1.051811	4.972026
18	8	0	2.956036	-0.554845	3.599039
19	8	0	5.140114	-0.002251	1.202882
20	8	0	6.187476	-1.623516	-0.060061
21	6	0	2.787874	-0.164422	4.964935
22	1	0	2.233574	-0.939481	5.499585
23	1	0	3.768102	0.013705	5.415512
24	6	0	6.418286	-0.522795	0.825228
25	1	0	6.942185	-0.877647	1.716932
26	1	0	6.986813	0.250460	0.303252
27	15	0	0.271736	-1.451379	-0.146571
28	15	0	1.274179	1.643077	-0.859142
29	46	0	-0.377181	0.249315	-1.588545
30	6	0	-0.279640	-1.266755	1.573494
31	6	0	0.227671	-2.094129	2.581958
32	6	0	-1.165510	-0.237142	1.898834
33	6	0	-0.170397	-1.901310	3.900524
34	1	0	0.950882	-2.868784	2.345236
35	6	0	-1.554411	-0.041577	3.220161
36	1	0	-1.535804	0.424143	1.121863
37	6	0	-1.057182	-0.874124	4.220373
38	6	0	-0.314314	-3.043484	-0.804428
39	6	0	-0.657703	-4.115278	0.023971
40	6	0	-0.328698	-3.211392	-2.195429
41	6	0	-0.993111	-5.345324	-0.534584
42	1	0	-0.672007	-3.999418	1.101668
43	6	0	-0.672111	-4.440451	-2.747542
44	1	0	-0.063443	-2.383637	-2.846510
45	6	0	-0.999044	-5.510344	-1.916682
46	6	0	0.738720	3.349323	-1.214036
47	6	0	1.622215	4.312387	-1.706004
48	6	0	-0.601042	3.689807	-0.979736
49	6	0	1.168452	5.605679	-1.956942
50	1	0	2.659901	4.066118	-1.902858
51	6	0	-1.044543	4.984113	-1.223067
52	1	0	-1.300409	2.946696	-0.605026
53	6	0	-0.159629	5.943084	-1.714312
54	6	0	2.875804	1.357288	-1.659727
55	6	0	2.957288	0.461633	-2.729403
56	6	0	4.032869	1.976301	-1.172063
57	6	0	4.193778	0.180369	-3.303168
58	1	0	2.059110	-0.029162	-3.096541
59	6	0	5.262868	1.700482	-1.758602
60	1	0	3.978482	2.655721	-0.326302
61	6	0	5.344655	0.796353	-2.816837
62	8	0	-2.113895	-0.487096	-2.665156
63	6	0	-3.378724	-0.392343	-2.040900
64	6	0	-3.601950	-1.524488	-1.088676
65	6	0	-3.723077	-1.642729	0.262062
66	8	0	-3.747412	-2.750899	-1.667707
67	6	0	-3.960814	-3.028842	0.532464
68	1	0	-3.664761	-0.844836	0.986957
69	6	0	-3.960569	-3.648680	-0.673420
70	1	0	-4.104731	-3.494205	1.496885
71	1	0	-4.090423	-4.674727	-0.981828
72	6	0	-3.543160	0.964108	-1.352164
73	1	0	-3.416392	1.765926	-2.084377
74	1	0	-2.799267	1.081935	-0.560241
75	8	0	-4.858775	1.070870	-0.806564
76	6	0	-4.990735	1.493904	0.458266
77	8	0	-4.049974	1.861983	1.136412
78	6	0	-6.440831	1.435007	0.922401
79	6	0	-6.909396	-0.030702	0.869262
80	1	0	-6.299961	-0.665855	1.520268
81	1	0	-7.947218	-0.090577	1.212301
82	1	0	-6.858940	-0.433247	-0.145891
83	6	0	-6.529720	1.961955	2.355990
84	1	0	-6.197799	3.002374	2.422139
85	1	0	-7.567462	1.912139	2.699791
86	1	0	-5.915711	1.364297	3.036235
87	6	0	-7.302150	2.295797	-0.017350
88	1	0	-8.340892	2.279736	0.327478
89	1	0	-6.962509	3.336913	-0.023951
90	1	0	-7.276643	1.917277	-1.042655
91	1	0	-4.141178	-0.467177	-2.830453
92	1	0	-0.838794	1.426757	-2.810747
93	1	0	-1.483385	0.780260	-2.913683
94	1	0	1.859460	6.348295	-2.343023
95	1	0	-0.507907	6.952459	-1.910265
96	1	0	-2.082466	5.239753	-1.035053
97	1	0	4.257908	-0.529261	-4.121833
98	1	0	6.308508	0.571215	-3.262518
99	1	0	6.158879	2.181493	-1.379630
100	1	0	-0.681517	-4.562331	-3.825950
101	1	0	-1.263252	-6.470746	-2.348373
102	1	0	-1.258481	-6.172852	0.115726
103	1	0	-2.240192	0.763224	3.464945
104	1	0	-1.353891	-0.718124	5.252917
105	1	0	0.221475	-2.547385	4.679614

6a(*R*)$_{seg}$•2b

RwB97XD SCF energy -3305.895695 a.u.

RwB97XD` SCF enthalpy -3304.997650a.u.

RwB97XD SCF free energy -3305.145719 a.u.

Three lowest frequencies (cm^{-1}) 11.9, 18.8 20.2

Cartesian coordinates:¥

Center Number	Atomic Number	Atomic Type	Coordinates X	(Angstroms) Y	Z
1	6	0	-3.269583	-0.078333	-0.061975
2	6	0	-4.424523	-0.644133	-0.556792
3	6	0	-5.043490	-0.217753	-1.726116
4	6	0	-4.554835	0.838951	-2.454367
5	6	0	-3.395925	1.455935	-1.960303
6	6	0	-2.754798	1.025932	-0.799096
7	1	0	-5.036377	1.181828	-3.362365
8	1	0	-2.996598	2.296572	-2.515759
9	6	0	-2.687523	-0.647589	1.185792
10	6	0	-1.507213	-1.437537	1.252506
11	6	0	-3.346235	-0.441109	2.377438
12	6	0	-1.061110	-1.931170	2.476164
13	6	0	-2.873628	-0.911931	3.596854
14	6	0	-1.730804	-1.667869	3.681654
15	1	0	-0.167161	-2.541796	2.519783
16	1	0	-1.361595	-2.045471	4.627922
17	8	0	-6.131691	-0.989595	-1.975343
18	8	0	-5.113315	-1.690618	-0.028951
19	8	0	-4.480686	0.283571	2.574196
20	8	0	-3.691598	-0.481716	4.596280
21	6	0	-6.198162	-1.955960	-0.921301
22	1	0	-6.089431	-2.959146	-1.342394
23	1	0	-7.144097	-1.844000	-0.385590
24	6	0	-4.824766	0.102605	3.949890
25	1	0	-5.678189	-0.580954	4.022363
26	1	0	-5.042817	1.070563	4.402317
27	15	0	-0.517313	-1.646619	-0.277985
28	15	0	-1.153961	1.793465	-0.345338
29	46	0	0.542303	0.435375	-0.904717
30	6	0	-1.686785	-2.365731	-1.474791
31	6	0	-2.551739	-3.407633	-1.121864
32	6	0	-1.761666	-1.805314	-2.753236
33	6	0	-3.479443	-3.882901	-2.042554
34	1	0	-2.509724	-3.838267	-0.125297
35	6	0	-2.702083	-2.273186	-3.667511
36	1	0	-1.099694	-0.985524	-3.022443
37	6	0	-3.562930	-3.308639	-3.310866
38	6	0	0.712824	-2.939796	0.108166
39	6	0	0.652270	-4.236338	-0.407794
40	6	0	1.795159	-2.576297	0.920996
41	6	0	1.652936	-5.157443	-0.101999
42	1	0	-0.167412	-4.536541	-1.051978
43	6	0	2.783283	-3.501556	1.237803
44	1	0	1.864812	-1.562193	1.305122
45	6	0	2.714372	-4.795205	0.722181
46	6	0	-1.176251	3.423104	-1.179947
47	6	0	-1.779944	4.540690	-0.596330
48	6	0	-0.617594	3.536301	-2.458726
49	6	0	-1.817290	5.752686	-1.280559
50	1	0	-2.214943	4.482767	0.394759
51	6	0	-0.669245	4.745246	-3.144929
52	1	0	-0.140387	2.675966	-2.919261
53	6	0	-1.266440	5.856961	-2.554756
54	6	0	-1.232870	2.100211	1.448439
55	6	0	-0.115522	1.805156	2.235418
56	6	0	-2.402791	2.572127	2.054467
57	6	0	-0.168584	1.980462	3.615311
58	1	0	0.788596	1.422516	1.768945
59	6	0	-2.447532	2.755916	3.432284
60	1	0	-3.291399	2.764127	1.460371
61	6	0	-1.333223	2.455449	4.213677
62	8	0	2.340785	-0.600017	-1.666126

63	6	0	3.684969	-0.100416	-1.505700
64	6	0	3.704283	0.925907	-0.430995
65	6	0	3.610034	0.869291	0.926180
66	8	0	3.788500	2.223090	-0.835787
67	6	0	3.622519	2.223767	1.387069
68	1	0	3.557308	-0.028032	1.524850
69	6	0	3.733771	2.996655	0.275367
70	1	0	3.564535	2.570538	2.408722
71	1	0	3.799877	4.061884	0.113595
72	6	0	4.581656	-1.295625	-1.185451
73	1	0	4.583341	-2.003704	-2.016456
74	1	0	4.243744	-1.799810	-0.279857
75	8	0	5.913507	-0.808551	-1.028605
76	6	0	6.465062	-0.827272	0.198258
77	8	0	5.978892	-1.424975	1.136187
78	6	0	7.735856	0.010953	0.247025
79	6	0	7.324865	1.481889	0.037961
80	1	0	6.630105	1.813425	0.817047
81	1	0	8.215483	2.116856	0.084490
82	1	0	6.848472	1.630016	-0.935137
83	6	0	8.397449	-0.160938	1.615448
84	1	0	8.665651	-1.205696	1.799558
85	1	0	9.311981	0.438742	1.659175
86	1	0	7.734963	0.167864	2.421194
87	6	0	8.693559	-0.434775	-0.869706
88	1	0	9.614854	0.153822	-0.816109
89	1	0	8.959989	-1.491466	-0.763051
90	1	0	8.252818	-0.288588	-1.859177
91	1	0	4.007157	0.372569	-2.437766
92	1	0	2.237798	-0.995082	-2.542079
93	1	0	1.183270	1.822730	-1.185093
94	1	0	-2.278462	6.616836	-0.812824
95	1	0	-1.298292	6.803718	-3.085074
96	1	0	-0.233327	4.819985	-4.136235
97	1	0	0.699205	1.739115	4.221450
98	1	0	-1.376257	2.586944	5.290469
99	1	0	-3.358535	3.120210	3.896383
100	1	0	-4.147938	-4.692173	-1.765540
101	1	0	-4.300765	-3.669022	-4.021174
102	1	0	-2.769866	-1.821230	-4.652107
103	1	0	1.597763	-6.161424	-0.511311
104	1	0	3.490247	-5.516537	0.959106
105	1	0	3.617270	-3.204830	1.866368

2.10. **Catalytic cycle 2b–1b (*S*)**

3b(*S*)$_{seg}$

RwB97XD SCF energy -3378.446298 a.u.

RwB97XD SCF enthalpy -3377.604319 a.u.

RwB97XD SCF free energy -3377.748936 a.u.

Three lowest frequencies (cm^{-1}) 14.1, 18.8 29.0

Cartesian coordinates:

Center Number	Atomic Number	Atomic Type	Coordinates (Angstroms) X	Y	Z
1	6	0	2.326292	1.110622	0.917311
2	6	0	3.416487	1.125727	1.760510
3	6	0	3.306924	1.203143	3.146442
4	6	0	2.085897	1.290291	3.769271
5	6	0	0.956919	1.294554	2.936941
6	6	0	1.053547	1.212813	1.548509
7	1	0	1.994001	1.357657	4.846761
8	1	0	-0.017116	1.367904	3.406237
9	6	0	2.566156	1.008340	-0.550552
10	6	0	2.348593	-0.161126	-1.332262
11	6	0	3.086694	2.095368	-1.218485
12	6	0	2.635235	-0.160058	-2.694811
13	6	0	3.349555	2.090578	-2.584900
14	6	0	3.138770	0.972839	-3.354327
15	1	0	2.468225	-1.056527	-3.280679
16	1	0	3.350516	0.960690	-4.417078
17	8	0	4.541300	1.195821	3.703462
18	8	0	4.726115	1.064637	1.410175
19	8	0	3.386303	3.312720	-0.698729
20	8	0	3.817600	3.305746	-2.964590
21	6	0	5.467479	1.007238	2.629254
22	1	0	5.941807	0.025583	2.726593
23	1	0	6.208762	1.809407	2.641891
24	6	0	3.910246	4.093056	-1.774726
25	1	0	4.960756	4.327727	-1.574448
26	1	0	3.315861	5.002704	-1.891578
27	15	0	1.513615	-1.595841	-0.552481
28	15	0	-0.483215	1.087480	0.565000
29	46	0	-0.779948	-1.014324	-0.146272
30	6	0	2.548248	-2.018571	0.884795
31	6	0	3.944805	-2.052775	0.803849
32	6	0	1.918972	-2.260248	2.109473
33	6	0	4.700070	-2.340543	1.935923
34	1	0	4.441630	-1.842643	-0.138874
35	6	0	2.678755	-2.529059	3.244956
36	1	0	0.834888	-2.217804	2.176910
37	6	0	4.068510	-2.569557	3.157974
38	6	0	1.668854	-2.963413	-1.752548
39	6	0	2.631206	-3.971029	-1.649603
40	6	0	0.751765	-2.995537	-2.812327
41	6	0	2.682547	-4.988648	-2.599795
42	6	0	0.814267	-4.005587	-3.766258
43	1	0	-0.011901	-2.226040	-2.891542
44	6	0	1.780010	-5.005241	-3.659696
45	6	0	-1.796980	1.688296	1.688967
46	6	0	-2.042326	3.051222	1.874075
47	6	0	-2.530125	0.753080	2.429093
48	6	0	-3.007352	3.469879	2.786608
49	1	0	-1.496021	3.795449	1.306066
50	6	0	-3.485389	1.174224	3.347575
51	1	0	-2.355054	-0.309102	2.285061
52	6	0	-3.725189	2.534841	3.527875
53	6	0	-0.307099	2.274124	-0.801347
54	6	0	-0.669483	1.881561	-2.093196
55	6	0	0.235506	3.546321	-0.587991
56	6	0	-0.493383	2.756725	-3.161213
57	1	0	-1.087397	0.892667	-2.263094
58	6	0	0.392876	4.422849	-1.655739
59	1	0	0.565841	3.844064	0.402266
60	6	0	0.034554	4.027016	-2.943356
61	8	0	-1.598657	-3.079509	-0.532968
62	6	0	-2.817122	-2.944964	-0.323612
63	1	0	-2.224116	-0.455812	0.016730
64	6	0	-3.377650	-2.990635	1.002594
65	6	0	-4.662073	-2.851365	1.470530
66	8	0	-2.515413	-3.107300	2.052056
67	6	0	-4.576578	-2.882868	2.884037
68	1	0	-5.552938	-2.735779	0.869741
69	6	0	-3.250084	-3.037896	3.171969

70	1	0	-5.383453	-2.805411	3.597340
71	1	0	-2.709086	-3.121724	4.103074
72	6	0	-3.778297	-2.729727	-1.489416
73	1	0	-4.570610	-3.480732	-1.474520
74	1	0	-3.220689	-2.785395	-2.423761
75	8	0	-4.399470	-1.454051	-1.334242
76	6	0	-3.871382	-0.420656	-2.027082
77	8	0	-3.115976	-0.565180	-2.964654
78	1	0	-4.045633	0.436917	3.914125
79	1	0	-4.475145	2.865617	4.239541
80	1	0	-3.196956	4.530699	2.915710
81	1	0	-0.768449	2.443845	-4.163619
82	1	0	0.173084	4.708566	-3.776937
83	1	0	0.813584	5.408523	-1.484457
84	1	0	5.782944	-2.374679	1.866139
85	1	0	4.660828	-2.777795	4.043756
86	1	0	2.185686	-2.700231	4.196689
87	1	0	3.342938	-3.973621	-0.831079
88	1	0	3.433385	-5.767595	-2.510485
89	1	0	1.824568	-5.797741	-4.400421
90	1	0	0.101758	-4.016911	-4.585217
91	6	0	-4.313246	0.891787	-1.493538
92	6	0	-4.962928	1.002179	-0.259504
93	6	0	-3.984336	2.040866	-2.217802
94	6	0	-5.276544	2.259377	0.244017
95	1	0	-5.204153	0.110811	0.308753
96	6	0	-4.293728	3.295550	-1.706936
97	1	0	-3.476820	1.940073	-3.170624
98	6	0	-4.936814	3.404754	-0.474674
99	1	0	-5.770692	2.347374	1.205718
100	1	0	-4.027600	4.187362	-2.265324
101	1	0	-5.169805	4.384864	-0.069851

TS1b(*S*)$_{seg}$

RwB97XD SCF energy --3378.434628 a.u.

RwB97XD SCF enthalpy --3377.596079 a.u.

RwB97XD SCF free energy -3377.739016 a.u.

Three lowest frequencies (cm^{-1}) -550.3, 15.2, 16.5

Imaginary frequency (cm^{-1}) -550.3

Standard orientation:

Center Number	Atomic Number	Atomic Type	Coordinates (Angstroms) X	Y	Z
1	6	0	2.148778	1.152829	1.147393
2	6	0	3.183413	1.166512	2.056117
3	6	0	3.000593	0.964030	3.419689
4	6	0	1.751450	0.778911	3.959736
5	6	0	0.672467	0.792819	3.063069
6	6	0	0.846887	0.970880	1.692331
7	1	0	1.603539	0.627300	5.022338
8	1	0	-0.323897	0.656476	3.467331
9	6	0	2.468752	1.324499	-0.297153
10	6	0	2.416487	0.287636	-1.269061
11	6	0	2.877535	2.557688	-0.752785
12	6	0	2.744274	0.544754	-2.597618
13	6	0	3.176405	2.810609	-2.087156
14	6	0	3.127584	1.821134	-3.038301
15	1	0	2.702259	-0.254601	-3.328665
16	1	0	3.368530	2.013596	-4.077051
17	8	0	4.203470	0.976980	4.047942
18	8	0	4.505721	1.323565	1.787877
19	8	0	3.010689	3.698246	-0.026764
20	8	0	3.492512	4.122357	-2.242471
21	6	0	5.185912	1.252504	3.043906
22	1	0	5.915621	0.439692	3.018370
23	1	0	5.661156	2.215018	3.253890
24	6	0	3.435569	4.713384	-0.940515
25	1	0	4.431918	5.062927	-0.655342
26	1	0	2.708425	5.529138	-0.943783
27	15	0	1.740308	-1.337420	-0.780444
28	15	0	-0.588689	0.804080	0.572481
29	46	0	-0.581264	-1.253230	-0.398609
30	6	0	2.719802	-1.865006	0.656954
31	6	0	4.107084	-1.687615	0.698144
32	6	0	2.051434	-2.397036	1.763884
33	6	0	4.817044	-2.040427	1.840882
34	1	0	4.629491	-1.254749	-0.150188
35	6	0	2.765179	-2.736210	2.909858
36	1	0	0.972042	-2.526615	1.734068
37	6	0	4.145821	-2.553983	2.949777
38	6	0	2.100606	-2.474083	-2.158172
39	6	0	3.191224	-3.347504	-2.154357
40	6	0	1.226164	-2.463729	-3.253387
41	6	0	3.407135	-4.194815	-3.238489
42	6	0	1.452635	-3.303833	-4.338171
43	1	0	0.366996	-1.796890	-3.257535
44	6	0	2.543280	-4.171640	-4.330320
45	6	0	-2.061391	1.041464	1.630043
46	6	0	-2.656380	2.290910	1.820229
47	6	0	-2.575909	-0.074757	2.302711
48	6	0	-3.742175	2.420157	2.682561
49	1	0	-2.294631	3.166166	1.293219
50	6	0	-3.658926	0.059880	3.163990
51	1	0	-2.121139	-1.051014	2.160972
52	6	0	-4.241887	1.310332	3.357466
53	6	0	-0.474636	2.206990	-0.575800
54	6	0	-0.587791	1.976034	-1.948537
55	6	0	-0.206759	3.498152	-0.105612
56	6	0	-0.440533	3.029517	-2.846344
57	1	0	-0.780293	0.971993	-2.312883
58	6	0	-0.079467	4.550411	-1.005501
59	1	0	-0.072601	3.680915	0.956615
60	6	0	-0.189532	4.316091	-2.375493
61	8	0	-1.173062	-3.219774	-0.985936
62	6	0	-2.343205	-2.809358	-0.643248
63	1	0	-2.166098	-1.195382	-0.206452
64	6	0	-2.910254	-3.223699	0.649850
65	6	0	-4.183118	-3.245137	1.137931
66	8	0	-2.029746	-3.604464	1.613255
67	6	0	-4.079401	-3.680115	2.492933
68	1	0	-5.081430	-2.984420	0.596951
69	6	0	-2.753950	-3.875971	2.724515
70	1	0	-4.883907	-3.828168	3.198296
71	1	0	-2.191968	-4.206127	3.585103
72	6	0	-3.341181	-2.482188	-1.757418
73	1	0	-4.019789	-3.329813	-1.877262
74	1	0	-2.788149	-2.311314	-2.679929
75	8	0	-4.138888	-1.354004	-1.416231
76	6	0	-3.778629	-0.168636	-1.950251
77	8	0	-2.966794	-0.068573	-2.849525
78	1	0	-4.045079	-0.814189	3.678918
79	1	0	-5.090736	1.417145	4.025780
80	1	0	-4.203555	3.393323	2.816488
81	1	0	-0.517785	2.841930	-3.912629
82	1	0	-0.072306	5.136950	-3.076254
83	1	0	0.122164	5.551219	-0.636996
84	1	0	5.893186	-1.901332	1.870365
85	1	0	4.700743	-2.811315	3.846742
86	1	0	2.241731	-3.134353	3.773430
87	1	0	3.871365	-3.377848	-1.309557
88	1	0	4.254183	-4.873610	-3.227396
89	1	0	2.716063	-4.833683	-5.173237
90	1	0	0.772867	-3.286436	-5.184267
91	6	0	-4.470982	0.977347	-1.315180
92	6	0	-5.407702	0.800279	-0.291361
93	6	0	-4.145752	2.264589	-1.754704
94	6	0	-6.027635	1.909252	0.272111
95	1	0	-5.649619	-0.196107	0.060743
96	6	0	-4.758239	3.369687	-1.176506
97	1	0	-3.413965	2.390956	-2.545131
98	6	0	-5.704662	3.191548	-0.168864
99	1	0	-6.756403	1.773710	1.064609
100	1	0	-4.500170	4.368328	-1.514209
101	1	0	-6.186607	4.054701	0.280192

4b(*S*)$_{seg}$

RwB97XD SCF energy -3378.441735 a.u.

RwB97XD SCF enthalpy -3377.598761 a.u.

RwB97XD SCF free energy -3377.740933 a.u.

Three lowest frequencies (cm^{-1}) 15.0, 20.3 26.5

Cartesian coordinates:

Center Number	Atomic Number	Atomic Type	Coordinates (Angstroms) X	Y	Z
1	6	0	-2.371055	0.969334	-1.183820
2	6	0	-3.419898	0.843752	-2.066416
3	6	0	-3.239856	0.594278	-3.423800
4	6	0	-1.989427	0.498279	-3.983987
5	6	0	-0.898628	0.650582	-3.113994
6	6	0	-1.069658	0.876783	-1.751482

7	1	0	-1.849044	0.315212	-5.042555
8	1	0	0.099933	0.582732	-3.530943
9	6	0	-2.650347	1.204685	0.259815
10	6	0	-2.417023	0.266140	1.304453
11	6	0	-3.163956	2.420071	0.651672
12	6	0	-2.689670	0.593558	2.630607
13	6	0	-3.405706	2.747964	1.982730
14	6	0	-3.187797	1.851350	3.000113
15	1	0	-2.513485	-0.134061	3.414373
16	1	0	-3.383365	2.100474	4.036126
17	8	0	-4.448780	0.484062	-4.029990
18	8	0	-4.745927	0.910269	-1.782702
19	8	0	-3.456403	3.480645	-0.143646
20	8	0	-3.846816	4.027422	2.062531
21	6	0	-5.436284	0.632994	-3.004836
22	1	0	-5.996940	-0.299980	-2.902509
23	1	0	-6.091089	1.472373	-3.249662
24	6	0	-3.954018	4.508188	0.718474
25	1	0	-5.005208	4.700570	0.485610
26	1	0	-3.344059	5.406981	0.605143
27	15	0	-1.617659	-1.322100	0.918732
28	15	0	0.366211	0.876514	-0.633182
29	46	0	0.592375	-1.138446	0.424055
30	6	0	-2.578230	-2.049916	-0.436433
31	6	0	-3.976626	-1.991090	-0.419430
32	6	0	-1.912476	-2.611361	-1.529917
33	6	0	-4.700834	-2.490412	-1.496252
34	1	0	-4.497090	-1.539647	0.420194
35	6	0	-2.643404	-3.092976	-2.611764
36	1	0	-0.826117	-2.654341	-1.540622
37	6	0	-4.035115	-3.028153	-2.596862
38	6	0	-1.771282	-2.376596	2.391062
39	6	0	-2.760454	-3.357199	2.502518
40	6	0	-0.851411	-2.192251	3.432576
41	6	0	-2.831714	-4.139698	3.652428
42	6	0	-0.934952	-2.970281	4.581190
43	1	0	-0.069224	-1.442495	3.343610
44	6	0	-1.924136	-3.946334	4.690381
45	6	0	1.867445	1.064447	-1.654785
46	6	0	2.529263	2.288517	-1.783302
47	6	0	2.388352	-0.073553	-2.288493
48	6	0	3.695634	2.369827	-2.539572
49	1	0	2.151066	3.177695	-1.290824
50	6	0	3.554958	0.013089	-3.038837
51	1	0	1.883138	-1.031384	-2.190231
52	6	0	4.210938	1.236547	-3.162529
53	6	0	0.202454	2.359221	0.398141
54	6	0	0.406665	2.262663	1.776570
55	6	0	-0.178500	3.580052	-0.171126
56	6	0	0.225626	3.382824	2.583722
57	1	0	0.691975	1.313253	2.218264
58	6	0	-0.349318	4.697030	0.639336
59	1	0	-0.360032	3.655159	-1.239446
60	6	0	-0.154960	4.596751	2.016544
61	8	0	1.348695	-2.957590	1.047639
62	6	0	2.536719	-2.472516	0.596987
63	6	0	3.080558	-3.125587	-0.637371
64	6	0	4.338807	-3.345273	-1.101810
65	8	0	2.165991	-3.472625	-1.581178
66	6	0	4.189276	-3.877630	-2.424286
67	1	0	5.261649	-3.147400	-0.575123
68	6	0	2.854768	-3.929056	-2.660548
69	1	0	4.973822	-4.183507	-3.101328
70	1	0	2.256972	-4.264030	-3.494587
71	6	0	3.597680	-2.251795	1.691223
72	1	0	4.278146	-3.100732	1.761774
73	1	0	3.091284	-2.096275	2.643548
74	8	0	4.405761	-1.118052	1.354669
75	6	0	3.923279	0.086026	1.704884
76	8	0	2.957772	0.224803	2.434416
77	1	0	3.952305	-0.876730	-3.516993
78	1	0	5.129449	1.304830	-3.737138
79	1	0	4.208588	3.321806	-2.628698
80	1	0	0.373547	3.301700	3.655838
81	1	0	-0.304290	5.466980	2.648118
82	1	0	-0.645675	5.642493	0.196523
83	1	0	-5.785231	-2.445291	-1.481815
84	1	0	-4.602961	-3.397781	-3.445039
85	1	0	-2.126022	-3.513371	-3.468139
86	1	0	-3.473901	-3.519348	1.701521
87	1	0	-3.599318	-4.902811	3.732703
88	1	0	-1.983533	-4.559786	5.584127
89	1	0	-0.220128	-2.822259	5.384324
90	6	0	4.671578	1.212743	1.097681
91	6	0	5.754739	1.002556	0.238571
92	6	0	4.246043	2.513629	1.386950
93	6	0	6.416274	2.093385	-0.314544
94	1	0	6.075466	-0.006226	0.003815
95	6	0	4.908082	3.599008	0.826979
96	1	0	3.398276	2.665857	2.047021
97	6	0	5.995675	3.389058	-0.020015
98	1	0	7.256191	1.931755	-0.982533
99	1	0	4.576894	4.608233	1.049860
100	1	0	6.511636	4.238158	-0.457874
101	1	0	2.398030	-1.349883	0.223531

5b(*S*)$_{seg}$

RwB97XD SCF energy -3379.614481 a.u.
RwB97XD SCF enthalpy -3378.751486 a.u.
RwB97XD SCF free energy -3378.898419 a.u.
Three lowest frequencies (cm^{-1}) 8.5, 9.9 16.3
Cartesian coordinates:

Center Number	Atomic Number	Atomic Type	Coordinates (Angstroms) X	Y	Z
1	6	0	3.357550	-0.373357	-0.325881
2	6	0	4.548500	0.113737	-0.815809
3	6	0	4.942337	-0.041151	-2.141116
4	6	0	4.178327	-0.738242	-3.044826
5	6	0	2.969801	-1.267222	-2.568254
6	6	0	2.553475	-1.093295	-1.250568
7	1	0	4.487909	-0.871851	-4.074459
8	1	0	2.353674	-1.826683	-3.261675
9	6	0	3.018991	-0.145493	1.104996
10	6	0	1.995203	0.717409	1.587664
11	6	0	3.753828	-0.803583	2.065074
12	6	0	1.791054	0.869717	2.957666
13	6	0	3.516335	-0.671015	3.429105
14	6	0	2.547323	0.173791	3.911537
15	1	0	1.030305	1.548434	3.320843
16	1	0	2.371079	0.298583	4.973145
17	8	0	6.129609	0.583552	-2.339206
18	8	0	5.482804	0.829688	-0.139115
19	8	0	4.769599	-1.681129	1.859189
20	8	0	4.370936	-1.463956	4.120206
21	6	0	6.526216	1.121345	-1.073439
22	1	0	6.646532	2.203582	-1.161426
23	1	0	7.450302	0.636717	-0.746766
24	6	0	5.165618	-2.154549	3.149490
25	1	0	6.220658	-1.923460	3.313767
26	1	0	4.972538	-3.228372	3.220457
27	15	0	0.880316	1.533049	0.395949
28	15	0	0.893108	-1.635268	-0.732905
29	46	0	-0.622043	0.120312	-0.635757
30	6	0	1.977763	2.335648	-0.807213
31	6	0	3.089778	3.057755	-0.358232
32	6	0	1.748825	2.181723	-2.176399
33	6	0	3.971314	3.611656	-1.279380
34	1	0	3.276922	3.172688	0.705453
35	6	0	2.642635	2.727307	-3.093400
36	1	0	0.886178	1.618107	-2.521183
37	6	0	3.755390	3.435570	-2.645622
38	6	0	-0.006324	2.837603	1.302787
39	6	0	0.208994	4.188687	1.027589
40	6	0	-0.966092	2.468471	2.253761
41	6	0	-0.521234	5.161397	1.708710
42	6	0	-1.682718	3.441711	2.937448
43	1	0	-1.163963	1.419495	2.452654
44	6	0	-1.462895	4.791538	2.663047
45	6	0	0.326369	-2.830507	-1.990000
46	6	0	0.328168	-4.207996	-1.760380
47	6	0	-0.145486	-2.334863	-3.213945
48	6	0	-0.130799	-5.078634	-2.746819
49	1	0	0.678109	-4.613103	-0.817381
50	6	0	-0.594240	-3.208354	-4.197280
51	1	0	-0.162241	-1.263834	-3.401322
52	6	0	-0.588913	-4.582786	-3.963433
53	6	0	1.112985	-2.534819	0.827799
54	6	0	0.261107	-2.256691	1.899760
55	6	0	2.149272	-3.463452	0.979948
56	6	0	0.453386	-2.892207	3.123064
57	1	0	-0.535476	-1.526093	1.784453

58	6	0	2.326396	-4.107211	2.199495
59	1	0	2.826645	-3.670450	0.156457
60	6	0	1.485728	-3.815069	3.273120
61	8	0	-1.888732	1.707779	-0.722460
62	6	0	-3.253531	1.425052	-0.561568
63	6	0	-4.032747	2.676114	-0.822780
64	6	0	-4.481226	3.700402	-0.047118
65	8	0	-4.308708	2.938046	-2.132547
66	6	0	-5.074817	4.654920	-0.936605
67	1	0	-4.395229	3.777884	1.027620
68	6	0	-4.939833	4.139726	-2.183907
69	1	0	-5.542489	5.593914	-0.676638
70	1	0	-5.231594	4.480893	-3.165931
71	6	0	-3.550496	0.865622	0.837201
72	1	0	-3.604983	1.648320	1.593726
73	1	0	-2.771929	0.152448	1.116031
74	8	0	-4.830142	0.216497	0.867200
75	6	0	-4.866663	-1.070409	0.513366
76	8	0	-3.875717	-1.710365	0.199520
77	1	0	-0.955329	-2.814211	-5.141874
78	1	0	-0.946738	-5.265501	-4.727801
79	1	0	-0.130106	-6.147517	-2.558206
80	1	0	-0.199263	-2.660631	3.958789
81	1	0	1.637765	-4.307396	4.228620
82	1	0	3.127331	-4.830692	2.314112
83	1	0	4.834583	4.168855	-0.929753
84	1	0	4.457155	3.850929	-3.362296
85	1	0	2.473366	2.589735	-4.156516
86	1	0	0.935008	4.494072	0.282129
87	1	0	-0.350082	6.210167	1.488061
88	1	0	-2.029809	5.552197	3.190860
89	1	0	-2.421963	3.145899	3.675007
90	6	0	-6.237705	-1.641584	0.555463
91	6	0	-7.350757	-0.861798	0.886533
92	6	0	-6.398348	-2.996590	0.251154
93	6	0	-8.614856	-1.440270	0.914556
94	1	0	-7.227219	0.190380	1.116875
95	6	0	-7.663641	-3.570185	0.282551
96	1	0	-5.529240	-3.592502	-0.007381
97	6	0	-8.772027	-2.792512	0.614849
98	1	0	-9.478947	-0.835441	1.170971
99	1	0	-7.787133	-4.622992	0.049491
100	1	0	-9.760126	-3.241783	0.639990
101	1	0	-3.598337	0.671559	-1.290520
102	1	0	-2.044205	-1.040307	-0.994402
103	1	0	-1.788121	-0.842338	-1.709687

TS2b(S)$_{seg}$

RwB97XD SCF energy -3379.590743 a.u.

RwB97XD SCF enthalpy -3378.729511 a.u.

RwB97XD SCF free energy -3378.876537 a.u.

Three lowest frequencies (cm^{-1}) -1058, 8.5, 12.2

Imaginary frequency (cm^{-1}) -1058

Cartesian coordinates:

Center	Atomic	Atomic	Coordinates		(Angstroms)
Number	Number	Type	X	Y	Z
1	6	0	3.366652	-0.431277	-0.126704
2	6	0	4.629383	0.020032	-0.439740
3	6	0	5.182410	-0.088116	-1.711687
4	6	0	4.508328	-0.695179	-2.742622
5	6	0	3.223366	-1.176734	-2.452890
6	6	0	2.650558	-1.050034	-1.189383
7	1	0	4.942570	-0.795933	-3.730003
8	1	0	2.673897	-1.659578	-3.252047
9	6	0	2.886621	-0.256447	1.272833
10	6	0	1.879493	0.655557	1.696143
11	6	0	3.490956	-0.987860	2.269970
12	6	0	1.567404	0.788660	3.046418
13	6	0	3.145875	-0.873827	3.612636
14	6	0	2.195931	0.021401	4.038773
15	1	0	0.822968	1.507705	3.363755
16	1	0	1.942319	0.132957	5.086164
17	8	0	6.416918	0.472341	-1.721161
18	8	0	5.504575	0.643518	0.389995
19	8	0	4.465135	-1.921928	2.123385
20	8	0	3.887217	-1.738769	4.348773
21	6	0	6.656815	0.959236	-0.396336
22	1	0	6.793389	2.043252	-0.427704
23	1	0	7.530732	0.454578	0.022937
24	6	0	4.744465	-2.427538	3.432359
25	1	0	5.788074	-2.220663	3.682938
26	1	0	4.526364	-3.497915	3.464748
27	15	0	0.934167	1.534713	0.412205
28	15	0	0.913237	-1.526353	-0.928131
29	46	0	-0.505748	0.252055	-0.884216
30	6	0	2.189722	2.362094	-0.608198
31	6	0	3.250729	3.038541	0.004369
32	6	0	2.129403	2.269171	-2.000914
33	6	0	4.251033	3.606100	-0.776872
34	1	0	3.304419	3.108830	1.087039
35	6	0	3.140557	2.828238	-2.777169
36	1	0	1.305871	1.741435	-2.475054
37	6	0	4.203252	3.489419	-2.165735
38	6	0	-0.059693	2.833222	1.207112
39	6	0	0.104599	4.180326	0.871051
40	6	0	-1.073181	2.462609	2.100343
41	6	0	-0.726814	5.144013	1.436795
42	6	0	-1.886955	3.430908	2.675884
43	1	0	-1.234579	1.415741	2.340786
44	6	0	-1.717553	4.773232	2.341068
45	6	0	0.454625	-2.644990	-2.293736
46	6	0	0.370895	-4.027913	-2.115990
47	6	0	0.167046	-2.088601	-3.547533
48	6	0	0.010639	-4.844535	-3.186224
49	1	0	0.571284	-4.478511	-1.150150
50	6	0	-0.177771	-2.909532	-4.614127
51	1	0	0.210266	-1.012263	-3.690208
52	6	0	-0.258649	-4.289774	-4.433344
53	6	0	0.893087	-2.485044	0.612385
54	6	0	-0.068672	-2.210691	1.587738
55	6	0	1.878166	-3.450682	0.849674
56	6	0	-0.033931	-2.888079	2.803147
57	1	0	-0.826644	-1.453283	1.403607
58	6	0	1.897919	-4.135076	2.059546
59	1	0	2.640872	-3.652408	0.103359
60	6	0	0.949778	-3.846008	3.039846
61	8	0	-2.125146	1.638942	-1.301775
62	6	0	-3.445203	1.429087	-0.823204
63	6	0	-4.160124	2.739728	-0.839685
64	6	0	-4.595844	3.605256	0.115775
65	8	0	-4.426044	3.248311	-2.076504
66	6	0	-5.167723	4.721301	-0.578146
67	1	0	-4.527018	3.473445	1.185885
68	6	0	-5.033641	4.449698	-1.899782
69	1	0	-5.617346	5.602834	-0.144112
70	1	0	-5.307784	4.978440	-2.800392
71	6	0	-3.484779	0.781665	0.566523
72	1	0	-3.430253	1.514909	1.368366
73	1	0	-2.652674	0.079257	0.661358
74	8	0	-4.725827	0.094711	0.765380
75	6	0	-4.792209	-1.168912	0.329234
76	8	0	-3.853180	-1.747365	-0.189320
77	1	0	-0.391550	-2.469733	-5.582959
78	1	0	-0.538908	-4.930531	-5.263680
79	1	0	-0.062194	-5.917150	-3.037766
80	1	0	-0.770975	-2.661616	3.566845
81	1	0	0.979308	-4.367223	3.991566
82	1	0	2.662181	-4.883801	2.241733
83	1	0	5.075223	4.127865	-0.300894
84	1	0	4.995932	3.916789	-2.772057
85	1	0	3.101426	2.737935	-3.858054
86	1	0	0.873132	4.484836	0.168643
87	1	0	-0.594227	6.187621	1.169907
88	1	0	-2.363142	5.527506	2.779956
89	1	0	-2.662502	3.134189	3.374906
90	6	0	-6.124345	-1.786386	0.554415
91	6	0	-7.198140	-1.055113	1.072731
92	6	0	-6.287957	-3.136331	0.229851
93	6	0	-8.427387	-1.676261	1.263948
94	1	0	-7.073064	-0.006476	1.318270
95	6	0	-7.517542	-3.753063	0.426517
96	1	0	-5.448558	-3.693323	-0.173141
97	6	0	-8.587314	-3.023484	0.943541
98	1	0	-9.261860	-1.109105	1.663838
99	1	0	-7.643370	-4.802165	0.178544
100	1	0	-9.548137	-3.505863	1.095642
101	1	0	-3.973149	0.744288	-1.507772
102	1	0	-1.632586	-0.504973	-1.981646
103	1	0	-1.941225	0.378059	-1.914110

6b(*S*)$_{seg}$•2b

RwB97XD SCF energy -3379.646893 a.u.

RwB97XD` SCF enthalpy -3378.780903 a.u.

RwB97XD SCF free energy -3378.931033 a.u.

Three lowest frequencies (cm^{-1}) 5.5, 11.2 13.6

Cartesian coordinates:

Center Number	Atomic Number	Atomic Type	Coordinates X	(Angstroms) Y	Z
1	6	0	3.368483	-0.450337	-0.233866
2	6	0	4.598474	-0.027606	-0.689071
3	6	0	5.032575	-0.208499	-1.997363
4	6	0	4.263257	-0.866637	-2.924945
5	6	0	3.017443	-1.337398	-2.484028
6	6	0	2.562534	-1.145208	-1.180295
7	1	0	4.596850	-1.016219	-3.944915
8	1	0	2.397731	-1.866696	-3.198407
9	6	0	3.008420	-0.150816	1.181256
10	6	0	2.069082	0.835335	1.592857
11	6	0	3.662685	-0.821473	2.190770
12	6	0	1.835143	1.062377	2.946252
13	6	0	3.399205	-0.606392	3.538431
14	6	0	2.492223	0.337182	3.953238
15	1	0	1.125148	1.821495	3.252099
16	1	0	2.293966	0.517615	5.003195
17	8	0	6.256902	0.355455	-2.155356
18	8	0	5.544140	0.645078	0.018740
19	8	0	4.606205	-1.790626	2.055236
20	8	0	4.158231	-1.445699	4.291671
21	6	0	6.630725	0.884658	-0.878945
22	1	0	6.801448	1.960262	-0.968442
23	1	0	7.521608	0.364069	-0.516985
24	6	0	4.978236	-2.181844	3.379396
25	1	0	6.029337	-1.931528	3.549881
26	1	0	4.795689	-3.250965	3.510567
27	15	0	1.068924	1.666004	0.301742
28	15	0	0.855583	-1.656815	-0.753130
29	46	0	-0.505591	0.126955	-0.683354
30	6	0	2.293784	2.421362	-0.813174
31	6	0	3.416381	3.098638	-0.323966
32	6	0	2.130079	2.254766	-2.191287
33	6	0	4.365792	3.599076	-1.208893
34	1	0	3.557134	3.221029	0.746200
35	6	0	3.087408	2.748340	-3.073804
36	1	0	1.264670	1.716724	-2.570929
37	6	0	4.207257	3.415197	-2.582316
38	6	0	0.190458	3.032740	1.133797
39	6	0	0.492239	4.378389	0.913470
40	6	0	-0.895929	2.698843	1.954634
41	6	0	-0.277041	5.374015	1.514184
42	6	0	-1.651692	3.693360	2.563868
43	1	0	-1.151448	1.654776	2.115885
44	6	0	-1.345248	5.035252	2.339466
45	6	0	0.414529	-2.908741	-2.011905
46	6	0	0.659396	-4.271699	-1.828388
47	6	0	-0.159656	-2.469346	-3.211409
48	6	0	0.330770	-5.181267	-2.831210
49	1	0	1.092197	-4.640356	-0.905461
50	6	0	-0.471139	-3.378519	-4.216260
51	1	0	-0.363882	-1.412155	-3.358235
52	6	0	-0.229565	-4.737780	-4.025409
53	6	0	0.989810	-2.507946	0.849973
54	6	0	0.065755	-2.216466	1.857784
55	6	0	2.035049	-3.403594	1.104416
56	6	0	0.192445	-2.807065	3.111886
57	1	0	-0.739820	-1.512728	1.663988
58	6	0	2.146271	-4.005365	2.353101
59	1	0	2.778934	-3.610697	0.340863
60	6	0	1.231792	-3.700753	3.359923
61	8	0	-2.043798	1.684903	-0.839957
62	6	0	-3.433760	1.308468	-0.718093
63	6	0	-4.324384	2.472368	-0.966013
64	6	0	-4.818259	3.467581	-0.180504
65	8	0	-4.732908	2.651499	-2.254813
66	6	0	-5.586287	4.314822	-1.040382
67	1	0	-4.657148	3.586616	0.881678
68	6	0	-5.496917	3.770962	-2.280981
69	1	0	-6.132643	5.205963	-0.766926
70	1	0	-5.905275	4.043273	-3.242571
71	6	0	-3.590398	0.748347	0.691842
72	1	0	-3.527525	1.537943	1.441192
73	1	0	-2.816685	0.000101	0.881253
74	8	0	-4.888080	0.173634	0.835403
75	6	0	-5.040873	-1.106498	0.464589
76	8	0	-4.125687	-1.797212	0.057282
77	1	0	-0.912208	-3.024847	-5.142859
78	1	0	-0.484481	-5.450004	-4.803994
79	1	0	0.513318	-6.239494	-2.673036
80	1	0	-0.519184	-2.564898	3.894819
81	1	0	1.332712	-4.158581	4.339062
82	1	0	2.956649	-4.701757	2.543901
83	1	0	5.236872	4.121455	-0.825777
84	1	0	4.958470	3.791744	-3.269831
85	1	0	2.963200	2.601365	-4.142057
86	1	0	1.320207	4.660067	0.271075
87	1	0	-0.038309	6.417531	1.333657
88	1	0	-1.940800	5.814212	2.805518
89	1	0	-2.484695	3.421438	3.205066
90	6	0	-6.441020	-1.577542	0.613303
91	6	0	-7.472487	-0.714209	0.997015
92	6	0	-6.713667	-2.923159	0.350988
93	6	0	-8.768989	-1.201859	1.118636
94	1	0	-7.262693	0.331389	1.192717
95	6	0	-8.010688	-3.405745	0.477313
96	1	0	-5.906275	-3.583294	0.051921
97	6	0	-9.038209	-2.545182	0.861249
98	1	0	-9.570401	-0.532396	1.414519
99	1	0	-8.220303	-4.451585	0.277089
100	1	0	-10.051421	-2.922948	0.960280
101	1	0	-3.647747	0.522660	-1.449985
102	1	0	-1.506690	-0.948391	-1.207633
103	1	0	-1.915900	2.154979	-1.675734

2.11. **Catalytic cycle 2b–1b (*R*)**

3b(*R*)$_{seg}$

RwB97XD SCF energy -3378.442921 a.u.
RwB97XD SCF enthalpy -3377.600762 a.u.
RwB97XD SCF free energy -3377.743934 a.u.
Three lowest frequencies (cm^{-1}) 17.9, 20.2 26.0
Cartesian coordinates:

Center Number	Atomic Number	Atomic Type	Coordinates (Angstroms) X	Y	Z
1	6	0	2.340480	1.478451	-0.306494
2	6	0	2.312675	2.798951	-0.698242
3	6	0	2.316248	3.200770	-2.029268
4	6	0	2.374062	2.289507	-3.054594
5	6	0	2.420233	0.936035	-2.688201
6	6	0	2.404477	0.520177	-1.357846
7	1	0	2.382456	2.595134	-4.094048
8	1	0	2.469312	0.200518	-3.482412
9	6	0	2.321486	1.185035	1.154506
10	6	0	1.209704	0.661448	1.871229
11	6	0	3.434658	1.493096	1.904948
12	6	0	1.289211	0.467128	3.247485
13	6	0	3.514642	1.261127	3.272890
14	6	0	2.452095	0.755088	3.979885
15	1	0	0.433657	0.082167	3.789086
16	1	0	2.504835	0.586503	5.049056
17	8	0	2.255189	4.555010	-2.098153
18	8	0	2.255506	3.890222	0.110499
19	8	0	4.606681	2.016486	1.457725
20	8	0	4.748977	1.609448	3.723582
21	6	0	2.188445	5.030594	-0.749733
22	1	0	1.237917	5.548826	-0.595201
23	1	0	3.041244	5.684395	-0.551074
24	6	0	5.449944	2.157967	2.603692
25	1	0	5.643790	3.220227	2.780269
26	1	0	6.376938	1.601503	2.447797
27	15	0	-0.246850	0.111633	0.904379
28	15	0	2.337340	-1.272841	-0.984069
29	46	0	0.277321	-1.899752	-0.333971
30	6	0	-0.745220	1.596321	-0.028117
31	6	0	-0.889834	2.836684	0.604101
32	6	0	-0.912467	1.502447	-1.412380
33	6	0	-1.208037	3.966421	-0.143175
34	1	0	-0.741817	2.922011	1.676949
35	6	0	-1.220334	2.636122	-2.159310
36	1	0	-0.771377	0.548043	-1.910556
37	6	0	-1.367874	3.867505	-1.525518
38	6	0	-1.573270	-0.234305	2.111827
39	6	0	-2.756076	0.505705	2.163657
40	6	0	-1.433898	-1.357957	2.940239
41	6	0	-3.778625	0.138743	3.037178
42	1	0	-2.890451	1.372559	1.528495
43	6	0	-2.442344	-1.702508	3.832124
44	1	0	-0.535105	-1.967075	2.885735
45	6	0	-3.620572	-0.956967	3.878260
46	6	0	2.916954	-2.085107	-2.516933
47	6	0	4.263369	-2.374854	-2.751260
48	6	0	1.967387	-2.386044	-3.501383
49	6	0	4.652002	-2.958726	-3.955129
50	1	0	5.016519	-2.162995	-2.000940
51	6	0	2.361435	-2.954993	-4.707343
52	1	0	0.916245	-2.176666	-3.321642
53	6	0	3.705713	-3.244225	-4.934855
54	6	0	3.601051	-1.525029	0.301290
55	6	0	3.290748	-2.283747	1.433702
56	6	0	4.851168	-0.903116	0.202480
57	6	0	4.221011	-2.412524	2.461384
58	1	0	2.314247	-2.753438	1.519301
59	6	0	5.784141	-1.051245	1.222800
60	1	0	5.089407	-0.281179	-0.655368
61	6	0	5.466239	-1.796702	2.356631
62	8	0	-1.643366	-2.812580	0.157470
63	6	0	-2.768318	-2.423559	-0.196389
64	1	0	0.722599	-3.223762	-1.031616
65	6	0	-3.921194	-2.819840	0.558151
66	6	0	-4.054598	-3.604706	1.678190
67	8	0	-5.149113	-2.371544	0.160007
68	6	0	-5.435828	-3.630711	1.981822
69	1	0	-3.250492	-4.097936	2.204069
70	6	0	-6.043688	-2.862278	1.028881
71	1	0	-5.922298	-4.144659	2.797286
72	1	0	-7.074325	-2.591833	0.852077
73	6	0	-2.941687	-1.568354	-1.436358
74	1	0	-3.524071	-2.124945	-2.177021
75	1	0	-1.955527	-1.335221	-1.842594
76	8	0	-3.589477	-0.347087	-1.103608
77	6	0	-4.765258	-0.066827	-1.700679
78	8	0	-5.307172	-0.810290	-2.491102
79	1	0	1.617308	-3.180585	-5.464579
80	1	0	4.013338	-3.697925	-5.871909
81	1	0	5.699420	-3.188196	-4.123981
82	1	0	6.752665	-0.568807	1.138632
83	1	0	6.189725	-1.893785	3.160239
84	1	0	3.969486	-2.987952	3.346775
85	1	0	-1.339075	2.556772	-3.235211
86	1	0	-1.606385	4.752805	-2.106987
87	1	0	-1.322758	4.925811	0.351619
88	1	0	-4.696952	0.717438	3.057712
89	1	0	-4.415473	-1.240475	4.560991
90	1	0	-2.316686	-2.565844	4.477892
91	6	0	-5.307317	1.245384	-1.269786
92	6	0	-4.503833	2.203795	-0.645819
93	6	0	-6.655430	1.515742	-1.520604
94	6	0	-5.051587	3.423605	-0.265880
95	1	0	-3.453325	2.002425	-0.476961
96	6	0	-7.201413	2.732221	-1.128330
97	1	0	-7.268270	0.767893	-2.013195
98	6	0	-6.400315	3.685631	-0.500688
99	1	0	-4.422717	4.169385	0.210278
100	1	0	-8.250933	2.937666	-1.313035
101	1	0	-6.828346	4.636322	-0.197502

TS1b(*R*)$_{seg}$

RwB97XD SCF energy -3378.427681 a.u.
RwB97XD SCF enthalpy -3377.588170 a.u.
RwB97XD SCF free energy -3377.729826 a.u.
Three lowest frequencies (cm^{-1}) -535.1, 15.6, 22.6
Imaginary frequency (cm^{-1}) -535.1
Cartesian coordinates:

Center Number	Atomic Number	Atomic Type	Coordinates (Angstroms) X	Y	Z
1	6	0	2.529956	0.409723	-0.709475
2	6	0	3.343598	1.389596	-1.234928
3	6	0	3.299666	1.784141	-2.567691
4	6	0	2.452103	1.188054	-3.469143
5	6	0	1.626293	0.169957	-2.970878
6	6	0	1.652158	-0.223040	-1.633810
7	1	0	2.415484	1.490111	-4.508921
8	1	0	0.944934	-0.315864	-3.660404
9	6	0	2.656845	0.100430	0.743161
10	6	0	1.694209	0.408034	1.745497
11	6	0	3.819883	-0.487641	1.189053
12	6	0	1.939736	0.096961	3.080414
13	6	0	4.043804	-0.818568	2.520649
14	6	0	3.122285	-0.533629	3.497892
15	1	0	1.207134	0.349193	3.837741
16	1	0	3.296609	-0.777142	4.539249
17	8	0	4.181053	2.795040	-2.773851
18	8	0	4.262073	2.134138	-0.565559
19	8	0	4.878002	-0.885607	0.434047
20	8	0	5.244947	-1.444903	2.637268
21	6	0	4.820561	3.043354	-1.517216
22	1	0	4.618416	4.070975	-1.204461
23	1	0	5.893043	2.854599	-1.612728
24	6	0	5.870323	-1.344663	1.355300
25	1	0	6.683648	-0.612416	1.402562
26	1	0	6.229897	-2.327366	1.047023
27	15	0	0.079494	1.087101	1.218514
28	15	0	0.425114	-1.442805	-1.061297
29	46	0	-1.244473	-0.578135	0.216497
30	6	0	0.507785	2.521382	0.187805
31	6	0	1.451484	3.454325	0.634182
32	6	0	-0.058726	2.655539	-1.080670
33	6	0	1.817414	4.515884	-0.185329
34	1	0	1.909843	3.343293	1.612953
35	6	0	0.321149	3.712628	-1.903265
36	1	0	-0.777847	1.922694	-1.434409
37	6	0	1.258336	4.640747	-1.457089

38	6	0	-0.762002	1.729748	2.702564
39	6	0	-0.980520	3.095399	2.905571
40	6	0	-1.274715	0.806200	3.623974
41	6	0	-1.688552	3.528853	4.024235
42	1	0	-0.611393	3.825740	2.194171
43	6	0	-1.967323	1.244849	4.746695
44	1	0	-1.133190	-0.258145	3.460750
45	6	0	-2.177533	2.608049	4.946677
46	6	0	-0.111233	-2.333774	-2.562307
47	6	0	0.755927	-3.223350	-3.208844
48	6	0	-1.384173	-2.106442	-3.091489
49	6	0	0.345964	-3.882605	-4.361890
50	1	0	1.750503	-3.408965	-2.817809
51	6	0	-1.788109	-2.765034	-4.250676
52	1	0	-2.063380	-1.411466	-2.607900
53	6	0	-0.926353	-3.655081	-4.884473
54	6	0	1.344689	-2.650427	-0.047792
55	6	0	0.725092	-3.215861	1.071933
56	6	0	2.664147	-2.997691	-0.357095
57	6	0	1.423562	-4.111012	1.877360
58	1	0	-0.299772	-2.950492	1.322668
59	6	0	3.357254	-3.893796	0.449420
60	1	0	3.166626	-2.543483	-1.205630
61	6	0	2.740320	-4.445910	1.570165
62	8	0	-2.977774	-0.517972	1.437873
63	6	0	-3.429682	-1.410835	0.629234
64	1	0	-2.215974	-1.702323	-0.424821
65	6	0	-3.483802	-2.808639	1.089264
66	6	0	-3.103262	-3.400807	2.254687
67	8	0	-3.951427	-3.736052	0.212127
68	6	0	-3.359386	-4.795499	2.084525
69	1	0	-2.694906	-2.903567	3.122518
70	6	0	-3.869340	-4.938526	0.832875
71	1	0	-3.192113	-5.584072	2.803550
72	1	0	-4.215232	-5.788467	0.264370
73	6	0	-4.492878	-0.972463	-0.388928
74	1	0	-5.470122	-1.230418	0.026794
75	1	0	-4.372509	-1.452939	-1.360014
76	8	0	-4.456966	0.440716	-0.486037
77	6	0	-3.791286	1.010106	-1.502231
78	8	0	-3.446732	0.410753	-2.502824
79	1	0	-2.778944	-2.581130	-4.654156
80	1	0	-1.243673	-4.172011	-5.784868
81	1	0	1.022058	-4.575681	-4.852479
82	1	0	4.384276	-4.149546	0.208818
83	1	0	3.288157	-5.134259	2.206270
84	1	0	0.939587	-4.540117	2.749161
85	1	0	-0.112619	3.805520	-2.893491
86	1	0	1.557099	5.461625	-2.101840
87	1	0	2.547292	5.239601	0.163593
88	1	0	-1.855629	4.591128	4.172973
89	1	0	-2.726065	2.950957	5.818573
90	1	0	-2.350539	0.521030	5.459121
91	6	0	-3.535895	2.450655	-1.258590
92	6	0	-3.442929	2.955493	0.042490
93	6	0	-3.310777	3.284304	-2.357051
94	6	0	-3.122399	4.294906	0.237834
95	1	0	-3.601546	2.299824	0.892195
96	6	0	-3.012210	4.626005	-2.154724
97	1	0	-3.376932	2.876282	-3.360596
98	6	0	-2.911745	5.129667	-0.857976
99	1	0	-3.035749	4.686307	1.246347
100	1	0	-2.846536	5.277945	-3.006369
101	1	0	-2.667002	6.175721	-0.701331

4b(*R*)$_{seg}$

RwB97XD SCF energy -3378.439960 a.u.

RwB97XD SCF enthalpy -3377.595368 a.u.

RwB97XD SCF free energy -3377.738241 a.u.

Three lowest frequencies (cm^{-1}) 13.9, 23.7 25.8

Cartesian coordinates:

Center Number	Atomic Number	Atomic Type	Coordinates X	(Angstroms) Y	Z
1	6	0	-2.452263	1.050011	0.055685
2	6	0	-3.033503	2.148656	-0.536122
3	6	0	-2.660678	3.455001	-0.233711
4	6	0	-1.701497	3.735604	0.709113
5	6	0	-1.111125	2.635910	1.351407
6	6	0	-1.468599	1.326044	1.044167
7	1	0	-1.413387	4.753150	0.945002
8	1	0	-0.353128	2.827033	2.103335
9	6	0	-2.886663	-0.317599	-0.341058
10	6	0	-2.095239	-1.262489	-1.055632
11	6	0	-4.145054	-0.741911	0.018662
12	6	0	-2.596106	-2.529318	-1.349767
13	6	0	-4.623917	-2.020021	-0.253872
14	6	0	-3.875664	-2.938403	-0.948829
15	1	0	-1.993113	-3.235416	-1.907256
16	1	0	-4.249304	-3.930697	-1.171020
17	8	0	-3.374731	4.319302	-0.999016
18	8	0	-3.996799	2.153674	-1.491867
19	8	0	-5.072690	-0.045191	0.723242
20	8	0	-5.862869	-2.164239	0.274001
21	6	0	-4.241311	3.525490	-1.815763
22	1	0	-4.008664	3.699719	-2.869433
23	1	0	-5.282379	3.768276	-1.587492
24	6	0	-6.201392	-0.910180	0.877007
25	1	0	-7.062427	-0.479275	0.359261
26	1	0	-6.402579	-1.062604	1.939724
27	15	0	-0.384062	-0.839504	-1.505263
28	15	0	-0.527277	-0.066691	1.742139
29	46	0	1.059625	-0.771923	0.224686
30	6	0	-0.505501	0.766016	-2.341222
31	6	0	-1.509861	0.961100	-3.297610
32	6	0	0.333303	1.822751	-1.978709
33	6	0	-1.670133	2.209905	-3.886371
34	1	0	-2.176060	0.147880	-3.570620
35	6	0	0.150158	3.075806	-2.555318
36	1	0	1.108005	1.673822	-1.230946
37	6	0	-0.850055	3.270192	-3.504829
38	6	0	0.188739	-2.090187	-2.689710
39	6	0	0.318481	-1.807299	-4.050718
40	6	0	0.525504	-3.361756	-2.206185
41	6	0	0.763939	-2.797556	-4.924004
42	1	0	0.081142	-0.822924	-4.439081
43	6	0	0.955759	-4.348146	-3.084497
44	1	0	0.456507	-3.577963	-1.143569
45	6	0	1.077211	-4.065667	-4.444917
46	6	0	0.359424	0.553425	3.206392
47	6	0	-0.164108	0.458306	4.499721
48	6	0	1.619006	1.132105	3.011410
49	6	0	0.563838	0.949254	5.579362
50	1	0	-1.130826	-0.001602	4.672997
51	6	0	2.343817	1.620084	4.093955
52	1	0	2.039201	1.203761	2.011991
53	6	0	1.814336	1.530620	5.379079
54	6	0	-1.744097	-1.279046	2.329440
55	6	0	-1.521755	-2.637268	2.090070
56	6	0	-2.905164	-0.866205	2.993680
57	6	0	-2.460777	-3.577589	2.505611
58	1	0	-0.625723	-2.956716	1.563996
59	6	0	-3.834667	-1.810473	3.414889
60	1	0	-3.094576	0.189973	3.162350
61	6	0	-3.616221	-3.164699	3.164756
62	8	0	2.582667	-1.288604	-0.991422
63	6	0	3.629622	-1.289113	-0.083270
64	6	0	4.145173	-2.677978	0.162687
65	6	0	3.963875	-3.858807	-0.484089
66	8	0	4.967756	-2.851322	1.234201
67	6	0	4.729347	-4.832870	0.240724
68	1	0	3.364805	-4.015186	-1.369694
69	6	0	5.314115	-4.167980	1.266927
70	1	0	4.827866	-5.886563	0.021392
71	1	0	5.971968	-4.463215	2.070291
72	6	0	4.766293	-0.370786	-0.554718
73	1	0	5.141191	-0.724538	-1.516554
74	1	0	5.578726	-0.336393	0.174389
75	8	0	4.274890	0.951521	-0.809779
76	6	0	4.112457	1.760848	0.240299
77	8	0	4.372140	1.432399	1.384319
78	1	0	3.321262	2.061921	3.928573
79	1	0	2.378089	1.908456	6.226387
80	1	0	0.151781	0.872207	6.580560
81	1	0	-4.733932	-1.487946	3.929962
82	1	0	-4.349750	-3.898678	3.483292
83	1	0	-2.291898	-4.631330	2.307784
84	1	0	0.784800	3.901631	-2.253829
85	1	0	-0.991809	4.250736	-3.948723
86	1	0	-2.446958	2.357667	-4.629746
87	1	0	0.866908	-2.572584	-5.980676
88	1	0	1.423007	-4.833624	-5.129787

89	1	0	1.208002	-5.332960	-2.704364
90	6	0	3.577451	3.094522	-0.140278
91	6	0	3.678139	3.578079	-1.448494
92	6	0	2.982571	3.878375	0.852996
93	6	0	3.200405	4.848865	-1.751767
94	1	0	4.140548	2.970634	-2.218818
95	6	0	2.476898	5.133765	0.536450
96	1	0	2.912827	3.499265	1.866985
97	6	0	2.593692	5.623260	-0.763620
98	1	0	3.293508	5.232241	-2.762845
99	1	0	2.002165	5.734660	1.305617
100	1	0	2.210701	6.609423	-1.007553
101	1	0	3.298685	-0.895095	0.913530

5b(*R*)$_{seg}$

RwB97XD SCF energy -3379.622481 a.u.
RwB97XD SCF enthalpy -3378.759462 a.u.
RwB97XD SCF free energy -3378.903359 a.u.
Three lowest frequencies (cm^{-1}) 12.1, 23.4 26.5
Cartesian coordinates:

Center Number	Atomic Number	Atomic Type	Coordinates (Angstroms) X	Y	Z
1	6	0	2.478601	0.485476	0.753092
2	6	0	2.987655	0.944309	1.947749
3	6	0	2.742053	2.223358	2.436134
4	6	0	2.000586	3.135928	1.725600
5	6	0	1.501089	2.710613	0.485421
6	6	0	1.726788	1.425432	-0.005158
7	1	0	1.810305	4.134926	2.099287
8	1	0	0.919600	3.419674	-0.092313
9	6	0	2.746631	-0.918778	0.345982
10	6	0	1.761794	-1.940532	0.249805
11	6	0	4.029952	-1.285761	0.015788
12	6	0	2.111278	-3.222783	-0.168319
13	6	0	4.362834	-2.561552	-0.428889
14	6	0	3.424678	-3.559181	-0.526883
15	1	0	1.360039	-4.000440	-0.222343
16	1	0	3.680174	-4.555077	-0.868516
17	8	0	3.327327	2.366752	3.651432
18	8	0	3.750176	0.252650	2.832039
19	8	0	5.127299	-0.486247	-0.023402
20	8	0	5.678269	-2.597809	-0.757460
21	6	0	4.004285	1.135767	3.927997
22	1	0	3.604491	0.699414	4.846543
23	1	0	5.078966	1.321242	4.003197
24	6	0	6.226526	-1.337129	-0.359562
25	1	0	6.859503	-1.478701	0.522265
26	1	0	6.781716	-0.902680	-1.191636
27	15	0	0.028681	-1.542089	0.650026
28	15	0	0.847913	0.871917	-1.506731
29	46	0	-1.110686	-0.134688	-0.780375
30	6	0	0.120779	-0.854941	2.328490
31	6	0	0.942364	-1.465292	3.284099
32	6	0	-0.545284	0.333315	2.634602
33	6	0	1.094022	-0.883641	4.537828
34	1	0	1.476205	-2.380674	3.045690
35	6	0	-0.375878	0.918718	3.885709
36	1	0	-1.174320	0.806450	1.886475
37	6	0	0.445965	0.314442	4.834031
38	6	0	-0.844577	-3.136159	0.690966
39	6	0	-1.208528	-3.744515	1.892458
40	6	0	-1.137570	-3.763896	-0.527056
41	6	0	-1.844017	-4.985442	1.874708
42	1	0	-1.005247	-3.261044	2.842038
43	6	0	-1.762337	-5.004348	-0.537299
44	1	0	-0.877649	-3.281439	-1.465713
45	6	0	-2.113615	-5.617976	0.664986
46	6	0	0.511104	2.354153	-2.522515
47	6	0	1.259267	2.634481	-3.670722
48	6	0	-0.530009	3.216245	-2.157183
49	6	0	0.973857	3.765188	-4.431928
50	1	0	2.060446	1.976288	-3.985939
51	6	0	-0.804585	4.348425	-2.914844
52	1	0	-1.129137	3.006630	-1.276379
53	6	0	-0.053841	4.624029	-4.055557
54	6	0	1.984972	-0.138992	-2.496749
55	6	0	1.494463	-1.284117	-3.126566
56	6	0	3.328067	0.224272	-2.649964
57	6	0	2.345086	-2.069911	-3.899583
58	1	0	0.452773	-1.567577	-3.001580
59	6	0	4.172476	-0.562182	-3.425276
60	1	0	3.717390	1.107514	-2.151898
61	6	0	3.682205	-1.710880	-4.045987
62	8	0	-2.754594	-0.922982	0.122874
63	6	0	-4.039822	-0.620146	-0.323647
64	6	0	-4.825906	-1.888052	-0.514846
65	6	0	-4.741905	-3.115466	0.061346
66	8	0	-5.854598	-1.861805	-1.408440
67	6	0	-5.786055	-3.907069	-0.522309
68	1	0	-4.019549	-3.420436	0.804794
69	6	0	-6.427339	-3.097610	-1.400926
70	1	0	-6.018524	-4.942014	-0.314480
71	1	0	-7.261524	-3.235784	-2.072157
72	6	0	-4.777746	0.249890	0.708013
73	1	0	-4.914064	-0.310618	1.634627
74	1	0	-5.750221	0.574135	0.325771
75	8	0	-4.004221	1.392979	1.105221
76	6	0	-3.872594	2.404735	0.249648
77	8	0	-4.403200	2.435818	-0.847418
78	1	0	-1.609346	5.012975	-2.619410
79	1	0	-0.274423	5.504328	-4.651340
80	1	0	1.557832	3.969294	-5.323838
81	1	0	5.214991	-0.283024	-3.539831
82	1	0	4.345538	-2.328209	-4.643787
83	1	0	1.962815	-2.965251	-4.379385
84	1	0	-0.876830	1.852626	4.119494
85	1	0	0.583387	0.779001	5.805566
86	1	0	1.733047	-1.356845	5.276444
87	1	0	-2.125691	-5.455570	2.811506
88	1	0	-2.606906	-6.584821	0.656091
89	1	0	-1.982861	-5.488480	-1.483217
90	6	0	-2.986616	3.479195	0.776591
91	6	0	-2.350844	3.361410	2.015824
92	6	0	-2.763351	4.607950	-0.015812
93	6	0	-1.461576	4.343837	2.436032
94	1	0	-2.558803	2.505660	2.646817
95	6	0	-1.877011	5.591456	0.408349
96	1	0	-3.274189	4.698121	-0.968956
97	6	0	-1.216674	5.454044	1.628923
98	1	0	-0.960302	4.243494	3.393487
99	1	0	-1.696764	6.462286	-0.213716
100	1	0	-0.517297	6.217533	1.955726
101	1	0	-4.046623	-0.087219	-1.282718
102	1	0	-1.958599	0.528496	-2.284304
103	1	0	-2.219519	1.017302	-1.728883

TS2b(*R*)$_{seg}$

RwB97XD SCF energy -3379.605276 a.u.
RwB97XD SCF enthalpy -3378.745744 a.u.
RwB97XD SCF free energy -3378.889828 a.u.
Three lowest frequencies (cm^{-1}) -1094, 16.1, 21.3
Imaginary frequency (cm^{-1}) -1094
Cartesian coordinates:

Center Number	Atomic Number	Atomic Type	Coordinates (Angstroms) X	Y	Z
1	6	0	2.419553	0.498405	0.981311
2	6	0	2.818332	0.833530	2.255665
3	6	0	2.450475	2.023023	2.877650
4	6	0	1.683845	2.967344	2.238757
5	6	0	1.279430	2.663900	0.930260
6	6	0	1.625275	1.467102	0.306735
7	1	0	1.406851	3.899135	2.717381
8	1	0	0.676542	3.392494	0.400517
9	6	0	2.850301	-0.803053	0.403503
10	6	0	1.986926	-1.893371	0.104829
11	6	0	4.182407	-0.994484	0.116660
12	6	0	2.491445	-3.063175	-0.457572
13	6	0	4.671240	-2.154670	-0.475547
14	6	0	3.850457	-3.215282	-0.770966
15	1	0	1.829805	-3.895126	-0.664473
16	1	0	4.229371	-4.123167	-1.224718
17	8	0	2.968122	2.060419	4.129990
18	8	0	3.585264	0.093565	3.096890
19	8	0	5.192996	-0.100324	0.275238
20	8	0	6.001196	-2.016224	-0.710781
21	6	0	3.661024	0.822760	4.324912
22	1	0	3.171447	0.253832	5.120361
23	1	0	4.708236	1.026846	4.559195

24	6	0	6.392282	-0.798732	-0.070378
25	1	0	6.952038	-1.030821	0.842105
26	1	0	6.979742	-0.194382	-0.762273
27	15	0	0.198461	-1.677986	0.391927
28	15	0	0.916807	1.082851	-1.325703
29	46	0	-0.862850	-0.332974	-1.172921
30	6	0	0.085858	-1.143406	2.122738
31	6	0	0.827316	-1.794358	3.115945
32	6	0	-0.681298	-0.023755	2.444806
33	6	0	0.792356	-1.320037	4.422569
34	1	0	1.439658	-2.657044	2.869077
35	6	0	-0.703126	0.455133	3.750894
36	1	0	-1.242034	0.482155	1.666943
37	6	0	0.036908	-0.190521	4.738542
38	6	0	-0.571298	-3.319897	0.253933
39	6	0	-1.121345	-3.972630	1.360440
40	6	0	-0.632915	-3.925590	-1.009199
41	6	0	-1.692915	-5.233949	1.209229
42	1	0	-1.104149	-3.507576	2.340303
43	6	0	-1.185623	-5.193542	-1.149414
44	1	0	-0.242999	-3.409938	-1.882670
45	6	0	-1.713472	-5.850585	-0.038322
46	6	0	0.369739	2.679129	-2.019003
47	6	0	1.254138	3.519014	-2.704269
48	6	0	-0.950944	3.089829	-1.811465
49	6	0	0.814764	4.753266	-3.173819
50	1	0	2.281055	3.218277	-2.881760
51	6	0	-1.382843	4.328288	-2.274060
52	1	0	-1.644493	2.441947	-1.286032
53	6	0	-0.500030	5.160503	-2.957298
54	6	0	2.284591	0.472224	-2.353028
55	6	0	2.068669	-0.601191	-3.221375
56	6	0	3.558317	1.043643	-2.250962
57	6	0	3.125117	-1.106822	-3.974032
58	1	0	1.082380	-1.051851	-3.296758
59	6	0	4.607355	0.539630	-3.011516
60	1	0	3.741258	1.857904	-1.556139
61	6	0	4.392798	-0.539621	-3.866930
62	8	0	-2.743287	-1.344761	-1.453093
63	6	0	-3.930724	-0.990460	-0.787281
64	6	0	-4.784700	-2.221449	-0.692953
65	6	0	-4.511665	-3.548999	-0.766090
66	8	0	-6.107991	-2.027600	-0.436953
67	6	0	-5.756882	-4.226656	-0.541913
68	1	0	-3.546474	-3.991727	-0.960507
69	6	0	-6.684583	-3.258026	-0.347811
70	1	0	-5.930128	-5.293491	-0.530815
71	1	0	-7.745969	-3.268602	-0.150448
72	6	0	-3.726060	-0.447752	0.629926
73	1	0	-2.985358	-1.057144	1.154389
74	1	0	-4.668804	-0.457487	1.181055
75	8	0	-3.197935	0.890279	0.644347
76	6	0	-4.077873	1.892800	0.503260
77	8	0	-5.251177	1.712187	0.240088
78	1	0	-2.409017	4.636067	-2.100627
79	1	0	-0.837569	6.124090	-3.326526
80	1	0	1.503104	5.396195	-3.713071
81	1	0	5.594358	0.982413	-2.924305
82	1	0	5.217035	-0.942265	-4.447399
83	1	0	2.957656	-1.948382	-4.638657
84	1	0	-1.288385	1.337273	3.991968
85	1	0	0.028023	0.186650	5.756523
86	1	0	1.365853	-1.824761	5.193551
87	1	0	-2.118945	-5.733955	2.073147
88	1	0	-2.154660	-6.836075	-0.150233
89	1	0	-1.217881	-5.661478	-2.127900
90	6	0	-3.470782	3.235253	0.705255
91	6	0	-2.224219	3.406414	1.314826
92	6	0	-4.194660	4.351351	0.274882
93	6	0	-1.706412	4.685064	1.486504
94	1	0	-1.656583	2.548318	1.654256
95	6	0	-3.667869	5.627600	0.438661
96	1	0	-5.163445	4.209980	-0.192860
97	6	0	-2.423033	5.795065	1.043376
98	1	0	-0.739516	4.813117	1.963262
99	1	0	-4.227084	6.491469	0.093881
100	1	0	-2.011235	6.791486	1.171172
101	1	0	-4.484431	-0.228609	-1.357661
102	1	0	-2.319645	-0.211591	-2.215048
103	1	0	-1.754925	0.469773	-2.468192

6b(*R*)$_{seg}$•2b

RwB97XD SCF energy -3379.661183 a.u.

RwB97XD` SCF enthalpy -3378.794609 a.u.

RwB97XD SCF free energy -3378.935756 a.u.

Three lowest frequencies (cm^{-1}) 21.4, 26.5, 32.9

Cartesian coordinates:

Center Number	Atomic Number	Atomic Type	Coordinates X	(Angstroms) Y	Z
1	6	0	2.400428	-0.599394	0.927591
2	6	0	2.895687	-0.824629	2.194587
3	6	0	2.968850	0.159940	3.171989
4	6	0	2.581887	1.453247	2.918410
5	6	0	2.118355	1.721679	1.623554
6	6	0	2.031798	0.741891	0.634928
7	1	0	2.626540	2.225768	3.676873
8	1	0	1.824218	2.736728	1.392955
9	6	0	2.240480	-1.774625	0.028111
10	6	0	0.989663	-2.391541	-0.255261
11	6	0	3.354426	-2.378927	-0.508342
12	6	0	0.939246	-3.539450	-1.041346
13	6	0	3.287280	-3.510186	-1.312988
14	6	0	2.091429	-4.124082	-1.592029
15	1	0	-0.013410	-4.014195	-1.244186
16	1	0	2.036160	-5.016804	-2.203873
17	8	0	3.427760	-0.377351	4.330664
18	8	0	3.318277	-2.011225	2.706441
19	8	0	4.649831	-1.989826	-0.367324
20	8	0	4.538051	-3.860894	-1.713009
21	6	0	3.703590	-1.755231	4.059334
22	1	0	3.112948	-2.383025	4.730594
23	1	0	4.775331	-1.939843	4.174607
24	6	0	5.432187	-2.896283	-1.149530
25	1	0	6.152146	-3.403167	-0.502039
26	1	0	5.932157	-2.350610	-1.954651
27	15	0	-0.545174	-1.546226	0.298010
28	15	0	1.254806	1.175652	-0.964942
29	46	0	-0.887603	0.504833	-0.962061
30	6	0	-0.377169	-1.438886	2.109998
31	6	0	0.129861	-2.505341	2.861678
32	6	0	-0.731295	-0.248491	2.749658
33	6	0	0.283028	-2.375555	4.237861
34	1	0	0.416191	-3.430754	2.370112
35	6	0	-0.565423	-0.117384	4.126465
36	1	0	-1.124487	0.581009	2.169430
37	6	0	-0.053145	-1.178014	4.869327
38	6	0	-1.891887	-2.741704	-0.009749
39	6	0	-2.533719	-3.444641	1.012610
40	6	0	-2.319506	-2.917148	-1.333380
41	6	0	-3.572489	-4.324114	0.712744
42	1	0	-2.232045	-3.312243	2.046213
43	6	0	-3.350096	-3.801278	-1.630243
44	1	0	-1.841077	-2.362634	-2.135845
45	6	0	-3.976977	-4.508829	-0.605591
46	6	0	1.641082	2.951870	-1.219038
47	6	0	2.903988	3.457223	-0.873402
48	6	0	0.713957	3.808861	-1.816851
49	6	0	3.221645	4.787785	-1.119347
50	1	0	3.640638	2.822612	-0.392111
51	6	0	1.038995	5.138811	-2.072022
52	1	0	-0.273465	3.444391	-2.078512
53	6	0	2.290764	5.632429	-1.720745
54	6	0	2.210838	0.295555	-2.254028
55	6	0	1.542442	-0.545139	-3.148984
56	6	0	3.602010	0.418891	-2.332527
57	6	0	2.258658	-1.273528	-4.094538
58	1	0	0.462035	-0.647465	-3.089085
59	6	0	4.312862	-0.300345	-3.287158
60	1	0	4.142395	1.055698	-1.640498
61	6	0	3.644429	-1.155199	-4.162040
62	8	0	-3.003406	0.039692	-1.358451
63	6	0	-4.169867	0.421568	-0.607061
64	6	0	-5.166930	-0.688772	-0.613719
65	6	0	-5.767300	-1.432496	0.352291
66	8	0	-5.639815	-1.045838	-1.839942
67	6	0	-6.677270	-2.308974	-0.324185
68	1	0	-5.591714	-1.368524	1.416799
69	6	0	-6.550484	-2.035517	-1.645763
70	1	0	-7.327366	-3.048756	0.120012
71	1	0	-7.009391	-2.436379	-2.536769
72	6	0	-3.772666	0.760599	0.820762

73	1	0	-3.311139	-0.101519	1.306509
74	1	0	-4.660799	1.065767	1.378279
75	8	0	-2.779842	1.788523	0.888858
76	6	0	-3.158933	3.041653	0.592927
77	8	0	-4.284964	3.321136	0.229526
78	1	0	0.302585	5.790095	-2.531695
79	1	0	2.538969	6.672459	-1.908671
80	1	0	4.200053	5.164515	-0.837979
81	1	0	5.392177	-0.198494	-3.341349
82	1	0	4.204290	-1.726760	-4.895863
83	1	0	1.732052	-1.936394	-4.773905
84	1	0	-0.826282	0.816556	4.614735
85	1	0	0.085201	-1.073022	5.940991
86	1	0	0.674961	-3.206159	4.816736
87	1	0	-4.065264	-4.863352	1.515866
88	1	0	-4.787355	-5.193450	-0.835879
89	1	0	-3.669278	-3.931455	-2.659602
90	6	0	-2.070036	4.037119	0.765087
91	6	0	-0.913924	3.759652	1.501155
92	6	0	-2.252362	5.303464	0.199958
93	6	0	0.040417	4.756053	1.683895
94	1	0	-0.765724	2.780632	1.944238
95	6	0	-1.283094	6.284846	0.363877
96	1	0	-3.156613	5.510158	-0.362977
97	6	0	-0.138251	6.013068	1.111423
98	1	0	0.926061	4.554679	2.278201
99	1	0	-1.421886	7.262209	-0.086973
100	1	0	0.616288	6.781715	1.247181
101	1	0	-4.600952	1.305293	-1.084441
102	1	0	-3.173190	0.204066	-2.293398
103	1	0	-1.028580	1.730173	-1.907440

2.12. **Catalytic cycle 2b–1c(*S*)**

3c(*S*)$_{seg}$

RwB97XD SCF energy -3186.757722 a.u.
RwB97XD SCF enthalpy -3185.973717 a.u.
RwB97XD SCF free energy -3186.114192 a.u.
Three lowest frequencies (cm^{-1}) 11.5, 14.8 29.8
Cartesian coordinates:

Center Number	Atomic Number	Atomic Type	Coordinates X	(Angstroms) Y	Z
1	6	0	-2.317207	-0.789135	0.713928
2	6	0	-3.316728	-0.725265	1.660516
3	6	0	-3.208289	-1.305354	2.921709
4	6	0	-2.094415	-2.016488	3.296092
5	6	0	-1.069956	-2.123153	2.343821
6	6	0	-1.160401	-1.535113	1.083418
7	1	0	-2.008234	-2.475787	4.273572
8	1	0	-0.183021	-2.684216	2.614633
9	6	0	-2.523454	-0.095979	-0.589485
10	6	0	-1.878645	1.113775	-0.970055
11	6	0	-3.418464	-0.616561	-1.498658
12	6	0	-2.136254	1.691605	-2.210009
13	6	0	-3.650136	-0.042279	-2.745241
14	6	0	-3.025920	1.118193	-3.132470
15	1	0	-1.637788	2.612947	-2.488455
16	1	0	-3.207296	1.572347	-4.099503
17	8	0	-4.330642	-1.057218	3.636954
18	8	0	-4.515081	-0.099640	1.547230
19	8	0	-4.154943	-1.749370	-1.372043
20	8	0	-4.532536	-0.802810	-3.440419
21	6	0	-5.168527	-0.245429	2.808877
22	1	0	-5.295787	0.737469	3.272473
23	1	0	-6.130267	-0.744504	2.664195
24	6	0	-4.943729	-1.850066	-2.558541
25	1	0	-6.001125	-1.718247	-2.305680
26	1	0	-4.767673	-2.817711	-3.034243
27	15	0	-0.570904	1.792164	0.118105
28	15	0	0.295472	-1.600320	-0.024008
29	46	0	1.359211	0.370517	0.029800
30	6	0	-1.373020	2.030796	1.734581
31	6	0	-2.664707	2.556377	1.850682
32	6	0	-0.686814	1.618590	2.881200
33	6	0	-3.256300	2.677471	3.103704
34	1	0	-3.213750	2.854586	0.962228
35	6	0	-1.288242	1.726753	4.132191
36	1	0	0.309594	1.193095	2.790154
37	6	0	-2.572311	2.255549	4.243414
38	6	0	-0.187313	3.443532	-0.558377
39	6	0	-0.756246	4.623987	-0.073577
40	6	0	0.736097	3.505724	-1.611443
41	6	0	-0.413659	5.849327	-0.641330
42	6	0	1.064674	4.730125	-2.183853
43	1	0	1.203707	2.597992	-1.985839
44	6	0	0.491654	5.904219	-1.697921
45	6	0	1.312869	-2.990808	0.596212
46	6	0	1.125559	-4.305302	0.161868
47	6	0	2.299617	-2.718402	1.552580
48	6	0	1.921467	-5.328561	0.672869
49	1	0	0.376249	-4.542801	-0.584520
50	6	0	3.082713	-3.743500	2.070027
51	1	0	2.459220	-1.697326	1.887405
52	6	0	2.896779	-5.051945	1.626749
53	6	0	-0.341460	-2.058805	-1.663840
54	6	0	0.096186	-1.352797	-2.788083
55	6	0	-1.305324	-3.064210	-1.803821
56	6	0	-0.426400	-1.651883	-4.043207
57	1	0	0.828860	-0.557257	-2.681349
58	6	0	-1.812984	-3.367910	-3.062262
59	1	0	-1.685559	-3.587266	-0.931549
60	6	0	-1.379507	-2.658406	-4.181121
61	8	0	2.772297	2.051328	0.454081
62	6	0	3.869380	1.518629	0.208031
63	1	0	2.528830	-0.634870	-0.215184
64	6	0	4.586807	0.751368	1.194186
65	6	0	5.792800	0.095364	1.156722
66	8	0	3.974246	0.523381	2.390046
67	6	0	5.920999	-0.573024	2.399252
68	1	0	6.495675	0.093225	0.335606
69	6	0	4.786327	-0.275751	3.099087

70	1	0	6.740577	-1.190615	2.734905
71	1	0	4.441271	-0.550069	4.085353
72	6	0	4.518161	1.709107	-1.160068
73	1	0	5.541895	2.070170	-1.044687
74	1	0	3.924266	2.425298	-1.727633
75	8	0	4.598550	0.455653	-1.840789
76	6	0	3.594847	0.150360	-2.690569
77	8	0	2.705340	0.924051	-2.974360
78	1	0	3.844884	-3.515662	2.808660
79	1	0	3.516257	-5.852453	2.019065
80	1	0	1.777707	-6.344208	0.318081
81	1	0	-0.093150	-1.091383	-4.910997
82	1	0	-1.792944	-2.884411	-5.159081
83	1	0	-2.561658	-4.147008	-3.164951
84	1	0	-4.255976	3.091880	3.190462
85	1	0	-3.043274	2.337698	5.218103
86	1	0	-0.757312	1.392022	5.017793
87	1	0	-1.464256	4.597953	0.748005
88	1	0	-0.857951	6.761295	-0.254881
89	1	0	0.753446	6.860554	-2.140193
90	1	0	1.778230	4.766557	-3.001408
91	6	0	3.723992	-1.251888	-3.196174
92	1	0	3.327873	-1.927088	-2.428768
93	1	0	3.141690	-1.371910	-4.109285
94	1	0	4.769628	-1.511761	-3.370007

TS1c(*S*)$_{seg}$

RwB97XD SCF energy -3186.752423 a.u.

RwB97XD SCF enthalpy -3185.969864 a.u.

RwB97XD SCF free energy -3186.105342 a.u.

Three lowest frequencies (cm^{-1}) -488.3, 17.6, 26.3

Imaginary frequency (cm^{-1}) -488.3

Cartesian coordinates:

Center	Atomic	Atomic	Coordinates	(Angstroms)	
Number	Number	Type	X	Y	Z
1	6	0	-2.048332	-0.818546	1.115404
2	6	0	-2.933237	-0.624588	2.153038
3	6	0	-2.576532	-0.746709	3.491707
4	6	0	-1.307058	-1.110242	3.868464
5	6	0	-0.387158	-1.338252	2.834006
6	6	0	-0.728471	-1.199011	1.490428
7	1	0	-1.027687	-1.218248	4.909751
8	1	0	0.620099	-1.629628	3.108082
9	6	0	-2.544726	-0.631447	-0.277973
10	6	0	-2.222632	0.468322	-1.120830
11	6	0	-3.421075	-1.554455	-0.803572
12	6	0	-2.767958	0.567338	-2.397998
13	6	0	-3.943167	-1.457644	-2.089193
14	6	0	-3.642927	-0.401395	-2.913563
15	1	0	-2.515356	1.411758	-3.029386
16	1	0	-4.057842	-0.317716	-3.911026
17	8	0	-3.647462	-0.467668	4.277157
18	8	0	-4.242491	-0.273044	2.057613
19	8	0	-3.887018	-2.679623	-0.201244
20	8	0	-4.744143	-2.525913	-2.337028
21	6	0	-4.721403	-0.121316	3.396613
22	1	0	-5.008930	0.919648	3.567277
23	1	0	-5.558991	-0.803315	3.560286
24	6	0	-4.716409	-3.340626	-1.161383
25	1	0	-5.728219	-3.436631	-0.759367
26	1	0	-4.286410	-4.315924	-1.404034
27	15	0	-0.957098	1.658835	-0.563899
28	15	0	0.566762	-1.332002	0.208734
29	46	0	1.205838	0.733989	-0.474096
30	6	0	-1.536104	2.272028	1.046893
31	6	0	-2.884131	2.568758	1.275567
32	6	0	-0.611799	2.382368	2.090313
33	6	0	-3.299527	2.972947	2.540102
34	1	0	-3.611385	2.465375	0.475241
35	6	0	-1.035372	2.772615	3.357415
36	1	0	0.435503	2.149514	1.913953
37	6	0	-2.378718	3.063613	3.583186
38	6	0	-1.001531	3.044587	-1.744062
39	6	0	-1.703664	4.226838	-1.496202
40	6	0	-0.287567	2.901123	-2.941231
41	6	0	-1.700484	5.248202	-2.443003
42	6	0	-0.299187	3.918388	-3.889178
43	1	0	0.273330	1.989786	-3.134449
44	6	0	-1.005371	5.093890	-3.639675
45	6	0	1.964655	-2.231670	0.971692
46	6	0	2.204258	-3.587998	0.741127
47	6	0	2.834275	-1.510272	1.801982
48	6	0	3.295091	-4.214396	1.342120
49	1	0	1.557827	-4.164367	0.088689
50	6	0	3.914662	-2.141057	2.406646
51	1	0	2.664333	-0.451745	1.981133
52	6	0	4.147233	-3.496682	2.176021
53	6	0	-0.136762	-2.387232	-1.089574
54	6	0	-0.065906	-1.975136	-2.422500
55	6	0	-0.804898	-3.571630	-0.757631
56	6	0	-0.668070	-2.739033	-3.417888
57	1	0	0.437557	-1.046324	-2.675157
58	6	0	-1.389001	-4.340110	-1.758398
59	1	0	-0.889372	-3.882584	0.279680
60	6	0	-1.329452	-3.919408	-3.086405
61	8	0	2.407947	2.453075	-0.891429
62	6	0	3.375826	1.650087	-0.611714
63	1	0	2.690234	0.138239	-0.414414
64	6	0	3.992612	1.711133	0.725179
65	6	0	5.181612	1.279086	1.234438
66	8	0	3.219933	2.231665	1.717131
67	6	0	5.140632	1.562741	2.632571
68	1	0	5.982762	0.810145	0.682546
69	6	0	3.928033	2.131992	2.865129
70	1	0	5.911145	1.368348	3.364096
71	1	0	3.446903	2.518895	3.750758
72	6	0	4.246487	1.169505	-1.770002
73	1	0	5.050777	1.896089	-1.910855
74	1	0	3.634958	1.113223	-2.670416
75	8	0	4.868274	-0.078596	-1.484556
76	6	0	4.286760	-1.200344	-1.962472
77	8	0	3.314587	-1.187579	-2.688097
78	1	0	4.576638	-1.570018	3.049879
79	1	0	4.996460	-3.988282	2.640210
80	1	0	3.477663	-5.267038	1.150805
81	1	0	-0.625928	-2.407686	-4.450563
82	1	0	-1.803501	-4.511144	-3.863353
83	1	0	-1.903686	-5.259961	-1.499431
84	1	0	-4.345868	3.203209	2.714540
85	1	0	-2.710317	3.361595	4.573185
86	1	0	-0.315971	2.842886	4.167302
87	1	0	-2.247001	4.360983	-0.566661
88	1	0	-2.243117	6.166718	-2.242358
89	1	0	-1.008508	5.890606	-4.377139
90	1	0	0.247644	3.795128	-4.818716
91	6	0	4.993416	-2.419806	-1.465383
92	1	0	4.888122	-2.471859	-0.377144
93	1	0	4.563651	-3.310275	-1.921627
94	1	0	6.060399	-2.353286	-1.693034

4c(*S*)$_{seg}$

RwB97XD SCF energy -3186.758619 a.u.

RwB97XD SCF enthalpy -3185.970683 a.u.

RwB97XD SCF free energy -3186.105921 a.u.

Three lowest frequencies (cm^{-1}) 22.5, 27.8. 31.2

Cartesian coordinates:

Center	Atomic	Atomic	Coordinates	(Angstroms)	
Number	Number	Type	X	Y	Z
1	6	0	-2.289771	-0.104305	-1.285182
2	6	0	-2.635437	-0.548223	-2.540626
3	6	0	-2.396477	0.192300	-3.695216
4	6	0	-1.826123	1.441451	-3.651554
5	6	0	-1.476285	1.928394	-2.382525
6	6	0	-1.692528	1.184196	-1.227017
7	1	0	-1.650335	2.020007	-4.550548
8	1	0	-1.021828	2.910478	-2.315904
9	6	0	-2.580126	-0.950155	-0.094287
10	6	0	-1.609848	-1.600322	0.721961
11	6	0	-3.892191	-1.127053	0.281680
12	6	0	-2.002113	-2.363015	1.820189
13	6	0	-4.272195	-1.874483	1.392992
14	6	0	-3.348644	-2.517691	2.180338
15	1	0	-1.255842	-2.860548	2.427873
16	1	0	-3.640507	-3.110370	3.038993
17	8	0	-2.818681	-0.507593	-4.778141
18	8	0	-3.220955	-1.729170	-2.864446
19	8	0	-4.993530	-0.613416	-0.323403
20	8	0	-5.621492	-1.848734	1.518042

21	6	0	-3.282579	-1.772002	-4.293091
22	1	0	-2.627637	-2.565447	-4.663995
23	1	0	-4.317704	-1.924269	-4.605540
24	6	0	-6.108467	-0.968729	0.498765
25	1	0	-6.852892	-1.489961	-0.105900
26	1	0	-6.522126	-0.068700	0.963127
27	15	0	0.160867	-1.322461	0.407663
28	15	0	-1.067807	1.748053	0.381060
29	46	0	0.855927	0.712904	1.044525
30	6	0	0.392391	-1.599669	-1.369757
31	6	0	-0.172576	-2.731332	-1.969942
32	6	0	1.079223	-0.663180	-2.145691
33	6	0	-0.036126	-2.925769	-3.339856
34	1	0	-0.729022	-3.449580	-1.374799
35	6	0	1.196331	-0.854837	-3.518508
36	1	0	1.506665	0.219219	-1.678072
37	6	0	0.639451	-1.984101	-4.114776
38	6	0	1.092696	-2.579832	1.329727
39	6	0	1.669573	-3.680180	0.693030
40	6	0	1.263205	-2.402258	2.709575
41	6	0	2.400215	-4.604108	1.437368
42	6	0	1.984322	-3.332250	3.445824
43	1	0	0.841661	-1.530202	3.201372
44	6	0	2.556899	-4.433162	2.808579
45	6	0	-0.504319	3.466097	0.210664
46	6	0	-1.215637	4.549894	0.733423
47	6	0	0.738536	3.678636	-0.405542
48	6	0	-0.690647	5.834881	0.626590
49	1	0	-2.172316	4.397744	1.222726
50	6	0	1.255695	4.965103	-0.509863
51	1	0	1.298474	2.838776	-0.811619
52	6	0	0.540508	6.043177	0.008012
53	6	0	-2.479726	1.696547	1.515247
54	6	0	-2.316103	1.130050	2.782070
55	6	0	-3.737676	2.153580	1.105571
56	6	0	-3.411451	1.007240	3.631954
57	1	0	-1.340891	0.762681	3.091602
58	6	0	-4.824733	2.040925	1.965286
59	1	0	-3.871485	2.581853	0.116271
60	6	0	-4.664210	1.458098	3.222114
61	8	0	2.681007	0.155187	1.646562
62	6	0	3.689577	0.283281	0.678868
63	6	0	4.308335	-1.038905	0.330678
64	6	0	5.165259	-1.882203	0.968226
65	8	0	3.933019	-1.585861	-0.860387
66	6	0	5.329305	-3.020576	0.113580
67	1	0	5.630306	-1.713558	1.929366
68	6	0	4.557312	-2.785797	-0.977060
69	1	0	5.937537	-3.895479	0.294274
70	1	0	4.352828	-3.338861	-1.881403
71	6	0	4.724851	1.271164	1.224984
72	1	0	5.263844	0.844280	2.072868
73	1	0	4.216008	2.184097	1.540517
74	8	0	5.728149	1.586310	0.247965
75	6	0	5.405250	2.499294	-0.678690
76	8	0	4.321255	3.049723	-0.725624
77	1	0	2.217661	5.119756	-0.987569
78	1	0	0.945589	7.047437	-0.068602
79	1	0	-1.244800	6.675617	1.031633
80	1	0	-3.287402	0.549827	4.608314
81	1	0	-5.518811	1.355804	3.883760
82	1	0	-5.799936	2.397927	1.649592
83	1	0	-0.470118	-3.805755	-3.803627
84	1	0	0.730921	-2.131037	-5.186429
85	1	0	1.718625	-0.119281	-4.121884
86	1	0	1.567318	-3.820729	-0.377032
87	1	0	2.854906	-5.452970	0.937077
88	1	0	3.132413	-5.152342	3.383118
89	1	0	2.113963	-3.189784	4.513826
90	6	0	6.540093	2.748619	-1.624704
91	1	0	6.805451	1.817590	-2.133454
92	1	0	6.251651	3.502390	-2.356363
93	1	0	7.418991	3.084943	-1.067933
94	1	0	3.301892	0.706642	-0.259642

5c(*S*)$_{seg}$

RwB97XD SCF energy -3187.942587 a.u.

RwB97XD SCF enthalpy -3187.137230 a.u.

RwB97XD SCF free energy -3187.274935 a.u.

Three lowest frequencies (cm^{-1}) 12.2, 17.2 30.9

Cartesian coordinates:

Center Number	Atomic Number	Atomic Type	Coordinates (Angstroms) X	Y	Z
1	6	0	2.443072	0.054114	1.050549
2	6	0	2.971876	-0.299082	2.271412
3	6	0	2.787515	0.457362	3.424035
4	6	0	2.088712	1.639748	3.405647
5	6	0	1.553680	2.035540	2.170050
6	6	0	1.710921	1.272318	1.015826
7	1	0	1.953490	2.237448	4.299105
8	1	0	1.004769	2.968598	2.132775
9	6	0	2.688613	-0.824727	-0.123400
10	6	0	1.703366	-1.616193	-0.776077
11	6	0	3.963242	-0.905526	-0.635758
12	6	0	2.045824	-2.411801	-1.867875
13	6	0	4.289000	-1.684100	-1.741631
14	6	0	3.351318	-2.460243	-2.377885
15	1	0	1.294176	-3.022799	-2.351581
16	1	0	3.602938	-3.077336	-3.232141
17	8	0	3.394690	-0.153831	4.472071
18	8	0	3.705875	-1.404523	2.558897
19	8	0	5.066232	-0.247173	-0.196113
20	8	0	5.604260	-1.535090	-2.032327
21	6	0	3.959205	-1.368616	3.966021
22	1	0	3.474774	-2.222609	4.447200
23	1	0	5.037849	-1.366846	4.139274
24	6	0	6.135717	-0.615166	-1.072132
25	1	0	6.924232	-1.106151	-0.496490
26	1	0	6.506481	0.273821	-1.588735
27	15	0	-0.034392	-1.493935	-0.240365
28	15	0	0.847067	1.729824	-0.526138
29	46	0	-1.072212	0.458711	-0.818320
30	6	0	-0.012987	-1.752032	1.553518
31	6	0	0.741212	-2.796224	2.101666
32	6	0	-0.698559	-0.868358	2.389447
33	6	0	0.802211	-2.951338	3.481721
34	1	0	1.292489	-3.473448	1.455796
35	6	0	-0.620043	-1.019223	3.770417
36	1	0	-1.272166	-0.051863	1.961279
37	6	0	0.131069	-2.057544	4.315960
38	6	0	-0.911503	-2.878989	-1.024968
39	6	0	-1.318313	-3.997762	-0.296569
40	6	0	-1.195666	-2.799200	-2.394505
41	6	0	-1.989299	-5.036586	-0.938936
42	6	0	-1.857314	-3.841894	-3.030717
43	1	0	-0.908213	-1.918189	-2.962039
44	6	0	-2.254938	-4.962868	-2.302717
45	6	0	0.354888	3.475882	-0.339790
46	6	0	1.015111	4.511221	-1.005765
47	6	0	-0.737671	3.772111	0.487266
48	6	0	0.592494	5.827901	-0.834124
49	1	0	1.852901	4.303088	-1.662281
50	6	0	-1.149028	5.087840	0.661568
51	1	0	-1.268578	2.971573	0.994824
52	6	0	-0.483243	6.118533	-0.000457
53	6	0	2.097400	1.647646	-1.837362
54	6	0	1.792656	1.005413	-3.039761
55	6	0	3.374778	2.181724	-1.630422
56	6	0	2.767303	0.885441	-4.026684
57	1	0	0.804707	0.578460	-3.191402
58	6	0	4.339503	2.070542	-2.624996
59	1	0	3.618967	2.669387	-0.691039
60	6	0	4.038890	1.414715	-3.818538
61	8	0	-2.776517	-0.650114	-0.901112
62	6	0	-3.948605	-0.034980	-0.438257
63	6	0	-3.898471	0.327371	1.017944
64	6	0	-4.284458	-0.287331	2.168043
65	8	0	-3.290680	1.513362	1.318727
66	6	0	-3.887206	0.569828	3.246438
67	1	0	-4.799833	-1.234397	2.243689
68	6	0	-3.285097	1.641295	2.673676
69	1	0	-4.036595	0.406142	4.304122
70	1	0	-2.837268	2.542707	3.064274
71	6	0	-5.074752	-1.035267	-0.715011
72	1	0	-4.924864	-1.947459	-0.134410
73	1	0	-5.078222	-1.281771	-1.778285
74	8	0	-6.359410	-0.533995	-0.320944
75	6	0	-6.979066	0.305085	-1.163778
76	8	0	-6.512498	0.643412	-2.233516
77	1	0	-1.992683	5.306679	1.308616
78	1	0	-0.806750	7.146435	0.130840

79	1	0	1.108662	6.627396	-1.356190
80	1	0	2.535434	0.371878	-4.954322
81	1	0	4.799093	1.316377	-4.587325
82	1	0	5.328207	2.488789	-2.465211
83	1	0	1.383928	-3.763159	3.906573
84	1	0	0.196893	-2.172174	5.393440
85	1	0	-1.143437	-0.321219	4.415862
86	1	0	-1.124576	-4.068288	0.768141
87	1	0	-2.303224	-5.904350	-0.367442
88	1	0	-2.776170	-5.775576	-2.799188
89	1	0	-2.070721	-3.773998	-4.092727
90	6	0	-8.299316	0.753454	-0.613586
91	1	0	-8.145126	1.263531	0.341472
92	1	0	-8.784918	1.426262	-1.319567
93	1	0	-8.936415	-0.115384	-0.426438
94	1	0	-4.167009	0.891102	-0.996024
95	1	0	-2.179782	1.964131	-1.180570
96	1	0	-1.811663	1.847582	-1.856104

TS2c(*S*)$_{seg}$

RwB97XD SCF energy -3187.925627 a.u.

RwB97XD SCF enthalpy -3187.122451 a.u.

RwB97XD SCF free energy -3187.259043 a.u.

Three lowest frequencies (cm^{-1}) -1059, 17.9, 24.5

Imaginary frequency (cm^{-1}) -1059

Cartesian coordinates:

Center Number	Atomic Number	Atomic Type	Coordinates (Angstroms) X	Y	Z
1	6	0	2.538644	-0.293770	0.947479
2	6	0	3.110916	-0.735875	2.118612
3	6	0	3.199200	0.049597	3.263975
4	6	0	2.749911	1.347482	3.287208
5	6	0	2.173866	1.832178	2.103780
6	6	0	2.058735	1.045275	0.960377
7	1	0	2.827004	1.963867	4.174789
8	1	0	1.809142	2.852527	2.098910
9	6	0	2.477039	-1.219488	-0.216913
10	6	0	1.290215	-1.803546	-0.742515
11	6	0	3.651148	-1.577390	-0.840577
12	6	0	1.351577	-2.676333	-1.825786
13	6	0	3.697942	-2.426353	-1.941228
14	6	0	2.562690	-3.004955	-2.453630
15	1	0	0.445876	-3.128989	-2.210466
16	1	0	2.595591	-3.682183	-3.298812
17	8	0	3.773995	-0.665267	4.262263
18	8	0	3.634597	-1.964583	2.362265
19	8	0	4.904609	-1.161718	-0.526232
20	8	0	4.981723	-2.560065	-2.358901
21	6	0	4.027984	-1.973287	3.737220
22	1	0	3.429756	-2.706725	4.284790
23	1	0	5.096365	-2.190046	3.807648
24	6	0	5.783081	-1.756671	-1.486120
25	1	0	6.506230	-2.392538	-0.969531
26	1	0	6.276935	-0.971431	-2.064310
27	15	0	-0.316558	-1.262205	-0.066948
28	15	0	1.178863	1.679743	-0.499720
29	46	0	-0.907529	0.828986	-0.858104
30	6	0	-0.164860	-1.449312	1.730263
31	6	0	0.358788	-2.630708	2.269371
32	6	0	-0.520386	-0.393090	2.569410
33	6	0	0.522457	-2.747579	3.644499
34	1	0	0.649498	-3.449243	1.617056
35	6	0	-0.346458	-0.512380	3.945289
36	1	0	-0.913560	0.526464	2.147368
37	6	0	0.178014	-1.685642	4.481330
38	6	0	-1.583882	-2.433324	-0.637968
39	6	0	-2.250828	-3.288322	0.244847
40	6	0	-1.941413	-2.422342	-1.993406
41	6	0	-3.242450	-4.142485	-0.231682
42	6	0	-2.920740	-3.288934	-2.465167
43	1	0	-1.459252	-1.731817	-2.679381
44	6	0	-3.572189	-4.150138	-1.584611
45	6	0	1.023133	3.479264	-0.271058
46	6	0	1.881912	4.384998	-0.897497
47	6	0	-0.003214	3.952446	0.557611
48	6	0	1.715895	5.752850	-0.690518
49	1	0	2.673730	4.038635	-1.552809
50	6	0	-0.156575	5.317249	0.768230
51	1	0	-0.682590	3.253481	1.038503
52	6	0	0.703068	6.219253	0.142244
53	6	0	2.282336	1.359916	-1.905720
54	6	0	1.764745	0.798975	-3.076714
55	6	0	3.656172	1.602723	-1.792445
56	6	0	2.620169	0.467853	-4.123315
57	1	0	0.699230	0.598497	-3.156708
58	6	0	4.503206	1.284209	-2.848030
59	1	0	4.066627	2.019147	-0.877146
60	6	0	3.987826	0.706424	-4.007417
61	8	0	-2.912030	0.537762	-1.569608
62	6	0	-4.055361	0.644230	-0.741287
63	6	0	-3.764140	0.963603	0.690574
64	6	0	-3.853774	0.270814	1.858013
65	8	0	-3.355426	2.241152	0.946520
66	6	0	-3.476654	1.178069	2.901198
67	1	0	-4.164373	-0.758328	1.969179
68	6	0	-3.177648	2.350774	2.290232
69	1	0	-3.429241	0.975707	3.961564
70	1	0	-2.850723	3.315391	2.648616
71	6	0	-4.808599	-0.681133	-0.867907
72	1	0	-4.243761	-1.489532	-0.402328
73	1	0	-4.962830	-0.902342	-1.925041
74	8	0	-6.069201	-0.658938	-0.183751
75	6	0	-7.104476	-0.081167	-0.811217
76	8	0	-7.019431	0.424051	-1.912541
77	1	0	-0.950758	5.675903	1.415313
78	1	0	0.577320	7.285824	0.300343
79	1	0	2.382543	6.452679	-1.184272
80	1	0	2.218225	0.015639	-5.024279
81	1	0	4.655204	0.439597	-4.821000
82	1	0	5.568087	1.473442	-2.758253
83	1	0	0.929332	-3.662564	4.062937
84	1	0	0.322296	-1.775247	5.553528
85	1	0	-0.611624	0.315492	4.595352
86	1	0	-2.008758	-3.292938	1.301940
87	1	0	-3.756238	-4.803498	0.458996
88	1	0	-4.342336	-4.821454	-1.951494
89	1	0	-3.183640	-3.279482	-3.518047
90	6	0	-8.351000	-0.156041	0.017247
91	1	0	-8.182850	0.327864	0.983336
92	1	0	-9.173798	0.331493	-0.504746
93	1	0	-8.599855	-1.203566	0.209525
94	1	0	-4.698066	1.447663	-1.130761
95	1	0	-1.469588	2.340691	-1.581705
96	1	0	-2.200237	1.788818	-1.663624

6c(*S*)$_{seg}$•2a

RwB97XD SCF energy -3187.977658 a.u.

RwB97XD` SCF enthalpy -3187.168643 a.u.

RwB97XD SCF free energy -3187.307734 a.u.

Three lowest frequencies (cm^{-1}) 14.5, 21.9, 30.0

Cartesian coordinates:

Center Number	Atomic Number	Atomic Type	Coordinates (Angstroms) X	Y	Z
1	6	0	2.240622	-0.342825	1.177531
2	6	0	2.580220	-0.798667	2.432246
3	6	0	2.435418	-0.036684	3.585865
4	6	0	1.969389	1.254276	3.539960
5	6	0	1.644739	1.759098	2.271915
6	6	0	1.769779	0.998872	1.109750
7	1	0	1.856704	1.854431	4.434935
8	1	0	1.283968	2.779362	2.213926
9	6	0	2.401511	-1.272348	0.024693
10	6	0	1.331410	-1.894675	-0.674388
11	6	0	3.671412	-1.604860	-0.391863
12	6	0	1.591403	-2.757570	-1.736121
13	6	0	3.918621	-2.443094	-1.472655
14	6	0	2.896987	-3.043506	-2.166706
15	1	0	0.768785	-3.232047	-2.258875
16	1	0	3.085861	-3.706217	-3.003046
17	8	0	2.807614	-0.768130	4.667069
18	8	0	3.056820	-2.030294	2.752708
19	8	0	4.847609	-1.149129	0.115328
20	8	0	5.259555	-2.521353	-1.689326
21	6	0	3.238101	-2.040113	4.171103
22	1	0	2.622998	-2.828208	4.612231
23	1	0	4.298256	-2.181402	4.398321
24	6	0	5.880019	-1.831663	-0.600998
25	1	0	6.361951	-2.556769	0.063291

26	1	0	6.595200	-1.104857	-0.990057
27	15	0	-0.383423	-1.397014	-0.261542
28	15	0	1.186765	1.695649	-0.481670
29	46	0	-0.802074	0.837006	-1.079403
30	6	0	-0.530878	-1.689992	1.529748
31	6	0	-0.036285	-2.851712	2.133182
32	6	0	-1.105027	-0.690480	2.319888
33	6	0	-0.124384	-3.009736	3.512429
34	1	0	0.431765	-3.623947	1.529201
35	6	0	-1.183395	-0.846995	3.701054
36	1	0	-1.471001	0.220284	1.853454
37	6	0	-0.690815	-2.005446	4.297273
38	6	0	-1.471955	-2.608447	-1.085067
39	6	0	-2.145136	-3.623375	-0.400605
40	6	0	-1.705862	-2.433070	-2.455668
41	6	0	-3.036536	-4.451456	-1.079370
42	6	0	-2.585856	-3.269566	-3.133120
43	1	0	-1.205113	-1.631150	-2.992443
44	6	0	-3.257437	-4.277538	-2.443081
45	6	0	1.151814	3.502948	-0.210859
46	6	0	2.238295	4.333173	-0.494359
47	6	0	-0.016528	4.055957	0.326675
48	6	0	2.150707	5.701694	-0.245587
49	1	0	3.149086	3.929855	-0.922789
50	6	0	-0.094127	5.418351	0.588994
51	1	0	-0.871094	3.418907	0.538431
52	6	0	0.990461	6.244919	0.298612
53	6	0	2.503367	1.327799	-1.682243
54	6	0	2.160276	0.795873	-2.928971
55	6	0	3.851611	1.498485	-1.347235
56	6	0	3.157024	0.430100	-3.828606
57	1	0	1.113167	0.648068	-3.181692
58	6	0	4.843589	1.148328	-2.257086
59	1	0	4.130373	1.875000	-0.367380
60	6	0	4.497753	0.605400	-3.493200
61	8	0	-2.841333	0.423971	-1.829330
62	6	0	-3.984478	0.639837	-0.974914
63	6	0	-3.792399	1.825074	-0.092955
64	6	0	-3.624454	1.996614	1.246394
65	8	0	-3.781436	3.035444	-0.719773
66	6	0	-3.497643	3.407744	1.459617
67	1	0	-3.616791	1.217870	1.995851
68	6	0	-3.594669	3.982880	0.234599
69	1	0	-3.355334	3.918501	2.401012
70	1	0	-3.559524	5.003513	-0.114864
71	6	0	-4.178390	-0.657687	-0.196810
72	1	0	-3.345516	-0.832426	0.485806
73	1	0	-4.258203	-1.487994	-0.900891
74	8	0	-5.337943	-0.592024	0.634153
75	6	0	-6.526970	-0.832883	0.051311
76	8	0	-6.644931	-1.082185	-1.130182
77	1	0	-1.004686	5.833368	1.010294
78	1	0	0.927485	7.311681	0.490257
79	1	0	2.994295	6.342611	-0.482325
80	1	0	2.886116	0.000143	-4.787674
81	1	0	5.274068	0.313049	-4.193376
82	1	0	5.887709	1.283625	-1.993306
83	1	0	0.260897	-3.912128	3.976799
84	1	0	-0.746216	-2.127386	5.374663
85	1	0	-1.618958	-0.060609	4.309780
86	1	0	-1.992858	-3.766097	0.663802
87	1	0	-3.559499	-5.233872	-0.538092
88	1	0	-3.954164	-4.923751	-2.968108
89	1	0	-2.757144	-3.125596	-4.195309
90	6	0	-7.641313	-0.761126	1.048742
91	1	0	-7.612444	0.199878	1.568752
92	1	0	-8.598656	-0.885426	0.543927
93	1	0	-7.513327	-1.548594	1.797240
94	1	0	-4.847223	0.801533	-1.627579
95	1	0	-0.906035	2.253567	-1.725928
96	1	0	-2.957226	0.939578	-2.637408

2.13. **Catalytic cycle 2b–1c(*R*)**

3c(*R*)$_{seg}$

RwB97XD SCF energy -3186.767217 a.u.
RwB97XD SCF enthalpy -3185.983013 a.u.
RwB97XD SCF free energy -3186.118672 a.u.
Three lowest frequencies (cm^{-1}) 22.8, 25.3 28.0
Cartesian coordinates:

Center Number	Atomic Number	Atomic Type	Coordinates (Angstroms) X	Y	Z
1	6	0	2.237346	-0.954570	0.665718
2	6	0	2.575067	-1.943836	1.563307
3	6	0	2.794373	-1.706250	2.915259
4	6	0	2.712362	-0.442658	3.445857
5	6	0	2.385358	0.589947	2.554207
6	6	0	2.149051	0.362708	1.199485
7	1	0	2.887856	-0.249824	4.497459
8	1	0	2.315665	1.594442	2.953976
9	6	0	2.017122	-1.350098	-0.754435
10	6	0	0.750323	-1.423743	-1.398130
11	6	0	3.101075	-1.749569	-1.505066
12	6	0	0.655893	-1.862401	-2.715896
13	6	0	2.997432	-2.156066	-2.830091
14	6	0	1.783431	-2.233131	-3.466090
15	1	0	-0.313038	-1.930464	-3.195650
16	1	0	1.694983	-2.566621	-4.493370
17	8	0	3.084048	-2.873834	3.543985
18	8	0	2.727826	-3.269337	1.300364
19	8	0	4.401374	-1.792049	-1.111290
20	8	0	4.232886	-2.447723	-3.316278
21	6	0	3.003643	-3.902628	2.552125
22	1	0	2.188863	-4.587473	2.803860
23	1	0	3.962222	-4.422891	2.489899
24	6	0	5.152852	-2.251188	-2.238304
25	1	0	5.631909	-3.202459	-1.990454
26	1	0	5.887670	-1.493562	-2.521153
27	15	0	-0.709367	-0.776166	-0.498020
28	15	0	1.602383	1.753941	0.139079
29	46	0	-0.591720	1.631562	-0.345931
30	6	0	-0.735095	-1.735549	1.050268
31	6	0	-0.614966	-3.129871	1.045108
32	6	0	-0.794032	-1.051154	2.266189
33	6	0	-0.561511	-3.827485	2.247621
34	1	0	-0.548310	-3.667955	0.103521
35	6	0	-0.730687	-1.750198	3.468328
36	1	0	-0.868137	0.032411	2.273490
37	6	0	-0.610819	-3.137717	3.459112
38	6	0	-2.178635	-1.271709	-1.463272
39	6	0	-3.128654	-2.175439	-0.983101
40	6	0	-2.405990	-0.625853	-2.687485
41	6	0	-4.280948	-2.440757	-1.721065
42	1	0	-2.983071	-2.670952	-0.029764
43	6	0	-3.545032	-0.910174	-3.431582
44	1	0	-1.692796	0.106446	-3.057272
45	6	0	-4.487947	-1.816021	-2.946437
46	6	0	2.072143	3.257955	1.067602
47	6	0	3.302998	3.894514	0.894329
48	6	0	1.163000	3.760577	2.007325
49	6	0	3.618058	5.020618	1.652301
50	1	0	4.018738	3.532420	0.164968
51	6	0	1.487904	4.874728	2.772710
52	1	0	0.198243	3.277715	2.139577
53	6	0	2.716376	5.508624	2.593272
54	6	0	2.655641	1.647102	-1.341319
55	6	0	2.071318	1.740235	-2.607950
56	6	0	4.026977	1.390854	-1.226011
57	6	0	2.850140	1.568863	-3.748871
58	1	0	1.003392	1.921993	-2.698577
59	6	0	4.804421	1.241186	-2.369341
60	1	0	4.485520	1.279164	-0.247822
61	6	0	4.215524	1.319092	-3.630088
62	8	0	-2.692483	1.888612	-0.867192
63	6	0	-3.674502	1.539207	-0.193817
64	1	0	-0.415989	3.185093	-0.358776
65	6	0	-4.956771	1.415064	-0.820605
66	6	0	-5.359979	1.584471	-2.123473
67	8	0	-6.023614	1.045186	-0.049940
68	8	0	-6.745277	1.300351	-2.149452
69	1	0	-4.726092	1.875921	-2.948205

70	6	0	-7.085632	0.979650	-0.864860
71	1	0	-7.409490	1.326210	-3.000448
72	1	0	-8.019844	0.694742	-0.403554
73	6	0	-3.549838	1.241982	1.285708
74	1	0	-4.247869	1.865074	1.853892
75	1	0	-2.526236	1.453171	1.608040
76	8	0	-3.861524	-0.139636	1.447648
77	6	0	-3.875121	-0.599444	2.712573
78	8	0	-3.660501	0.119029	3.665161
79	1	0	0.777447	5.252440	3.501361
80	1	0	2.966509	6.385927	3.182038
81	1	0	4.572602	5.515528	1.503212
82	1	0	5.867733	1.045755	-2.273204
83	1	0	4.820297	1.179945	-4.520812
84	1	0	2.388001	1.625971	-4.729223
85	1	0	-0.771107	-1.210393	4.408878
86	1	0	-0.554556	-3.683079	4.396150
87	1	0	-0.467985	-4.909015	2.239418
88	1	0	-5.014829	-3.140947	-1.333978
89	1	0	-5.386749	-2.024028	-3.518801
90	1	0	-3.703810	-0.412196	-4.382948
91	6	0	-4.170552	-2.065946	2.752287
92	1	0	-3.338850	-2.611300	2.295288
93	1	0	-4.292335	-2.389318	3.785504
94	1	0	-5.073810	-2.283972	2.177069

TS1c(*R*)$_{seg}$

RwB97XD SCF energy -3186.746891 a.u.
RwB97XD SCF enthalpy -3185.964823 a.u.
RwB97XD SCF free energy -3186.101631 a.u.
Three lowest frequencies (cm^{-1}) -500.0, 17.6, 26.3
Imaginary frequency (cm^{-1}) -500.0
Cartesian coordinates:

Center	Atomic	Atomic	Coordinates		(Angstroms)
Number	Number	Type	X	Y	Z
1	6	0	-2.378523	0.415124	-0.727370
2	6	0	-3.388104	-0.057260	-1.537432
3	6	0	-3.359799	0.039049	-2.924330
4	6	0	-2.328827	0.663008	-3.583357
5	6	0	-1.301653	1.186515	-2.784568
6	6	0	-1.308399	1.074900	-1.395296
7	1	0	-2.305526	0.746628	-4.663364
8	1	0	-0.476726	1.686343	-3.280263
9	6	0	-2.493953	0.205812	0.743347
10	6	0	-1.703483	-0.697404	1.505945
11	6	0	-3.445994	0.910083	1.445025
12	6	0	-1.891975	-0.819176	2.880084
13	6	0	-3.608349	0.801764	2.822004
14	6	0	-2.848446	-0.061056	3.572906
15	1	0	-1.287100	-1.515566	3.449235
16	1	0	-2.977471	-0.153761	4.644671
17	8	0	-4.458675	-0.562751	-3.446146
18	8	0	-4.514313	-0.708061	-1.144842
19	8	0	-4.301578	1.844950	0.953945
20	8	0	-4.565237	1.673791	3.235920
21	6	0	-5.232611	-1.033011	-2.337730
22	1	0	-5.350118	-2.116956	-2.411407
23	1	0	-6.199374	-0.522982	-2.327750
24	6	0	-5.131425	2.238496	2.050049
25	1	0	-6.140191	1.838975	1.901891
26	1	0	-5.136728	3.326398	2.131912
27	15	0	-0.349103	-1.590343	0.668527
28	15	0	0.181077	1.597129	-0.476233
29	46	0	1.456309	-0.213058	0.025902
30	6	0	-1.148306	-2.453257	-0.717943
31	6	0	-2.374846	-3.107408	-0.556651
32	6	0	-0.552853	-2.389703	-1.980316
33	6	0	-2.993906	-3.697631	-1.653495
34	1	0	-2.854268	-3.140468	0.417468
35	6	0	-1.181757	-2.971498	-3.077363
36	1	0	0.387786	-1.861946	-2.107516
37	6	0	-2.403180	-3.621095	-2.914540
38	6	0	0.247299	-2.844585	1.848162
39	6	0	-0.101074	-4.194612	1.760950
40	6	0	1.119725	-2.420291	2.859922
41	6	0	0.411080	-5.106157	2.681852
42	1	0	-0.766308	-4.544049	0.978302
43	6	0	1.617815	-3.331461	3.784109
44	1	0	1.409006	-1.374520	2.925127
45	6	0	1.265727	-4.677268	3.693551
46	6	0	1.008024	2.844438	-1.529649
47	6	0	0.637963	4.193049	-1.490521
48	6	0	2.012406	2.432917	-2.413675
49	6	0	1.267674	5.114819	-2.321939
50	1	0	-0.130147	4.539205	-0.808349
51	6	0	2.630198	3.357353	-3.250819
52	1	0	2.305917	1.388612	-2.463269
53	6	0	2.262063	4.699544	-3.204120
54	6	0	-0.396830	2.471338	1.011332
55	6	0	0.250491	2.241383	2.228511
56	6	0	-1.491925	3.341202	0.959659
57	6	0	-0.198118	2.872680	3.385544
58	1	0	1.091510	1.553064	2.272514
59	6	0	-1.930227	3.976828	2.115903
60	1	0	-2.022851	3.498313	0.025516
61	6	0	-1.287493	3.738753	3.329690
62	8	0	3.073426	-1.540006	0.415741
63	6	0	3.802774	-0.547363	0.054299
64	1	0	2.743087	0.669043	-0.363350
65	6	0	4.448497	0.253987	1.109195
66	6	0	4.433412	0.148141	2.466520
67	8	0	5.185943	1.327255	0.720765
68	6	0	5.224213	1.234875	2.950258
69	1	0	3.928188	-0.614248	3.042126
70	6	0	5.650374	1.913184	1.852356
71	1	0	5.449256	1.472734	3.979645
72	1	0	6.265252	2.789030	1.711358
73	6	0	4.540343	-0.627384	-1.294725
74	1	0	5.606237	-0.733635	-1.081535
75	1	0	4.382497	0.262644	-1.905078
76	8	0	4.171252	-1.810387	-1.985278
77	6	0	3.103184	-1.777786	-2.797321
78	8	0	2.478675	-0.762842	-3.035381
79	1	0	3.403223	3.025332	-3.936905
80	1	0	2.750536	5.421114	-3.851808
81	1	0	0.980510	6.160600	-2.275431
82	1	0	-2.781208	4.649180	2.071171
83	1	0	-1.641348	4.224254	4.233879
84	1	0	0.300134	2.682584	4.330953
85	1	0	-0.723593	-2.904811	-4.059073
86	1	0	-2.897455	-4.068211	-3.771571
87	1	0	-3.945341	-4.204484	-1.526416
88	1	0	0.140276	-6.154506	2.604545
89	1	0	1.660806	-5.391682	4.409316
90	1	0	2.289320	-2.992359	4.566720
91	6	0	2.784676	-3.135911	-3.335732
92	1	0	2.314575	-3.719783	-2.537146
93	1	0	2.091455	-3.049298	-4.171732
94	1	0	3.695124	-3.655028	-3.641781

4c(*R*)$_{seg}$

RwB97XD SCF energy -3186.760305 a.u.
RwB97XD SCF enthalpy -3185.972019 a.u.
RwB97XD SCF free energy -3186.107243 a.u.
Three lowest frequencies (cm^{-1}) 16.6, 25.7 31.1
Cartesian coordinates:

Center	Atomic	Atomic	Coordinates		(Angstroms)
Number	Number	Type	X	Y	Z
1	6	0	2.506430	0.453067	0.676842
2	6	0	3.276366	0.497809	1.816899
3	6	0	3.178164	1.522425	2.753663
4	6	0	2.330564	2.588891	2.574948
5	6	0	1.557074	2.588289	1.403436
6	6	0	1.630666	1.554215	0.474504
7	1	0	2.259972	3.389189	3.301988
8	1	0	0.882834	3.420495	1.233012
9	6	0	2.636384	-0.699033	-0.256365
10	6	0	1.645977	-1.699503	-0.471966
11	6	0	3.786796	-0.824711	-1.000260
12	6	0	1.858126	-2.720216	-1.397506
13	6	0	3.977234	-1.832503	-1.941298
14	6	0	3.032327	-2.805566	-2.158387
15	1	0	1.102264	-3.481499	-1.547700
16	1	0	3.179608	-3.595392	-2.885058
17	8	0	4.010615	1.266231	3.794349
18	8	0	4.179174	-0.424655	2.234957
19	8	0	4.860872	0.004649	-0.991907
20	8	0	5.173462	-1.663482	-2.553499

21	6	0	4.712939	0.062555	3.469864
22	1	0	4.549053	-0.678324	4.255356
23	1	0	5.776094	0.284126	3.339898
24	6	0	5.788194	-0.521788	-1.945465
25	1	0	6.700901	-0.833051	-1.430258
26	1	0	5.991822	0.230640	-2.710528
27	15	0	0.043745	-1.578117	0.377467
28	15	0	0.452550	1.479008	-0.911615
29	46	0	-1.289542	0.068932	-0.387028
30	6	0	0.421472	-1.333568	2.135282
31	6	0	1.446047	-2.065606	2.746442
32	6	0	-0.273170	-0.362030	2.859896
33	6	0	1.769442	-1.818333	4.076389
34	1	0	2.002852	-2.809720	2.184747
35	6	0	0.068190	-0.106533	4.183511
36	1	0	-1.068606	0.200290	2.381211
37	6	0	1.091576	-0.831272	4.790234
38	6	0	-0.801943	-3.169844	0.162010
39	6	0	-0.835887	-4.121604	1.183382
40	6	0	-1.432585	-3.435047	-1.060911
41	6	0	-1.482652	-5.337725	0.974430
42	1	0	-0.365810	-3.925651	2.141149
43	6	0	-2.068268	-4.653417	-1.265023
44	1	0	-1.432323	-2.687171	-1.848829
45	6	0	-2.094198	-5.605565	-0.246464
46	6	0	-0.282128	3.134201	-1.091686
47	6	0	0.190520	4.074777	-2.011764
48	6	0	-1.375203	3.453027	-0.275824
49	6	0	-0.422176	5.321251	-2.102371
50	1	0	1.027478	3.842160	-2.661617
51	6	0	-1.982047	4.700587	-0.367039
52	1	0	-1.750654	2.722759	0.435213
53	6	0	-1.503847	5.635903	-1.282093
54	6	0	1.427681	1.140041	-2.402937
55	6	0	0.959178	0.198690	-3.322788
56	6	0	2.655748	1.776977	-2.615283
57	6	0	1.722332	-0.113555	-4.444518
58	1	0	0.010129	-0.303150	-3.152251
59	6	0	3.408915	1.468246	-3.742700
60	1	0	3.032109	2.495573	-1.892965
61	6	0	2.946840	0.517368	-4.651857
62	8	0	-2.904627	-0.983816	0.193848
63	6	0	-3.961157	-0.086429	0.087333
64	6	0	-4.981629	-0.603099	-0.887094
65	6	0	-5.104913	-1.785065	-1.545374
66	8	0	-6.016023	0.222533	-1.208787
67	6	0	-6.301188	-1.682482	-2.331516
68	1	0	-4.429117	-2.625347	-1.477196
69	6	0	-6.810116	-0.450238	-2.087741
70	1	0	-6.719892	-2.430726	-2.989514
71	1	0	-7.682479	0.080424	-2.438210
72	6	0	-4.608490	0.165690	1.454880
73	1	0	-4.881875	-0.789287	1.906870
74	1	0	-5.489044	0.806085	1.370241
75	8	0	-3.666747	0.755985	2.365352
76	6	0	-3.516314	2.086389	2.319280
77	8	0	-4.139396	2.799664	1.555784
78	1	0	-2.827335	4.932924	0.272527
79	1	0	-1.978436	6.609002	-1.361031
80	1	0	-0.053312	6.048215	-2.819028
81	1	0	4.361812	1.962083	-3.904959
82	1	0	3.543852	0.267098	-5.523451
83	1	0	1.363888	-0.854991	-5.151464
84	1	0	-0.460547	0.663696	4.736217
85	1	0	1.361271	-0.628289	5.821959
86	1	0	2.564036	-2.385903	4.550047
87	1	0	-1.506544	-6.075058	1.770384
88	1	0	-2.597313	-6.554506	-0.404286
89	1	0	-2.552382	-4.854243	-2.215449
90	6	0	-2.481073	2.570906	3.289093
91	1	0	-2.509231	1.996387	4.216637
92	1	0	-1.489830	2.443303	2.838896
93	1	0	-2.637403	3.631219	3.488485
94	1	0	-3.619143	0.906111	-0.292832

5c(*R*)$_{seg}$

RwB97XD SCF energy	-3187.943043 a.u.
RwB97XD SCF enthalpy	-3187.137164 a.u.
RwB97XD SCF free energy	-3187.272132 a.u.
Three lowest frequencies (cm^{-1})	20.0, 28.5, 29.2

Cartesian coordinates:

Center Number	Atomic Number	Atomic Type	Coordinates (Angstroms) X	Y	Z
1	6	0	2.371137	-0.333012	1.079099
2	6	0	2.794805	-0.663947	2.346551
3	6	0	2.875858	0.261085	3.382312
4	6	0	2.585113	1.589021	3.187821
5	6	0	2.181343	1.961744	1.896826
6	6	0	2.064042	1.038621	0.860460
7	1	0	2.660106	2.315663	3.988026
8	1	0	1.957148	3.006703	1.721342
9	6	0	2.272601	-1.397723	0.045957
10	6	0	1.053863	-1.906106	-0.480404
11	6	0	3.425812	-1.948835	-0.462856
12	6	0	1.063547	-2.896056	-1.461554
13	6	0	3.425104	-2.920536	-1.458560
14	6	0	2.256554	-3.423859	-1.976103
15	1	0	0.129991	-3.281821	-1.853226
16	1	0	2.253228	-4.185871	-2.746253
17	8	0	3.263137	-0.364309	4.521764
18	8	0	3.146372	-1.894698	2.799915
19	8	0	4.703199	-1.624730	-0.137121
20	8	0	4.701593	-3.234061	-1.789185
21	6	0	3.488974	-1.735583	4.179657
22	1	0	2.847209	-2.373043	4.792273
23	1	0	4.546872	-1.973297	4.321188
24	6	0	5.549976	-2.430895	-0.961546
25	1	0	6.160294	-3.080005	-0.328238
26	1	0	6.167757	-1.786931	-1.592454
27	15	0	-0.524539	-1.170005	0.045762
28	15	0	1.324873	1.541225	-0.734389
29	46	0	-0.917239	0.955024	-0.696220
30	6	0	-0.521839	-1.216032	1.862319
31	6	0	-0.056740	-2.351410	2.535478
32	6	0	-0.913319	-0.085709	2.588199
33	6	0	0.010933	-2.354373	3.924813
34	1	0	0.275326	-3.223629	1.979954
35	6	0	-0.829160	-0.090931	3.976997
36	1	0	-1.264831	0.803573	2.072692
37	6	0	-0.364483	-1.222074	4.645047
38	6	0	-1.818158	-2.279958	-0.590831
39	6	0	-2.358601	-3.300347	0.192101
40	6	0	-2.265964	-2.098873	-1.904862
41	6	0	-3.338775	-4.136490	-0.339014
42	1	0	-2.034491	-3.445231	1.217084
43	6	0	-3.241492	-2.937294	-2.429940
44	1	0	-1.861241	-1.292537	-2.511021
45	6	0	-3.780455	-3.956908	-1.645926
46	6	0	1.468312	3.358727	-0.821123
47	6	0	2.413867	3.989973	-1.633493
48	6	0	0.587612	4.136302	-0.055533
49	6	0	2.484835	5.380885	-1.667931
50	1	0	3.093901	3.408236	-2.245885
51	6	0	0.669864	5.523503	-0.086509
52	1	0	-0.165572	3.659567	0.566009
53	6	0	1.619893	6.147916	-0.893262
54	6	0	2.388791	0.855627	-2.033704
55	6	0	1.808748	0.211051	-3.128535
56	6	0	3.782113	0.944703	-1.929228
57	6	0	2.620031	-0.352703	-4.109880
58	1	0	0.727409	0.130832	-3.202972
59	6	0	4.586415	0.390523	-2.918234
60	1	0	4.237405	1.430292	-1.070837
61	6	0	4.005856	-0.265119	-4.003549
62	8	0	-2.811889	0.471916	-0.185056
63	6	0	-3.879382	0.789406	-1.015030
64	6	0	-4.944386	-0.259488	-0.841258
65	6	0	-5.280382	-1.081023	0.187179
66	8	0	-5.792314	-0.453518	-1.889190
67	6	0	-6.412085	-1.841994	-0.259142
68	1	0	-4.777145	-1.147442	1.140578
69	6	0	-6.676655	-1.422393	-1.520594
70	1	0	-6.947009	-2.605717	0.287733
71	1	0	-7.414859	-1.695040	-2.259332
72	6	0	-4.426357	2.189302	-0.678799
73	1	0	-5.397225	2.362398	-1.147923
74	1	0	-3.729641	2.969400	-0.989744
75	8	0	-4.640892	2.263046	0.736191
76	6	0	-3.641244	2.712562	1.502425
77	8	0	-2.664656	3.303782	1.076452
78	1	0	-0.011412	6.115612	0.516302

79	1	0	1.681393	7.231444	-0.921747
80	1	0	3.220703	5.862523	-2.304078
81	1	0	5.666154	0.463079	-2.836142
82	1	0	4.637067	-0.709319	-4.766951
83	1	0	2.167997	-0.864656	-4.953386
84	1	0	-1.118778	0.794135	4.534816
85	1	0	-0.291136	-1.220693	5.728181
86	1	0	0.371973	-3.237475	4.442166
87	1	0	-3.761022	-4.923901	0.277158
88	1	0	-4.550147	-4.605195	-2.052920
89	1	0	-3.590836	-2.786491	-3.446388
90	6	0	-3.883350	2.403981	2.948721
91	1	0	-3.684000	1.338993	3.109763
92	1	0	-3.216941	2.996169	3.575743
93	1	0	-4.925798	2.591843	3.215154
94	1	0	-3.615706	0.807331	-2.085884
95	1	0	-1.275961	2.384765	-1.879490
96	1	0	-1.555659	2.675346	-1.213309

TS2c(*R*)$_{seg}$

RwB97XD SCF energy -3187.922202 a.u.
RwB97XD SCF enthalpy -3187.120455 a.u.
RwB97XD SCF free energy -3187.258904 a.u.
Three lowest frequencies (cm^{-1}) -1098, 17.2, 19.1
Imaginary frequency (cm^{-1}) -1098
Cartesian coordinates:

Center Number	Atomic Number	Atomic Type	Coordinates (Angstroms) X	Y	Z
1	6	0	2.692449	-0.219321	0.822556
2	6	0	3.406975	-0.701582	1.895894
3	6	0	3.562159	0.005550	3.083806
4	6	0	3.036784	1.263961	3.248286
5	6	0	2.320408	1.791387	2.163864
6	6	0	2.139661	1.082055	0.978281
7	1	0	3.164782	1.822086	4.167985
8	1	0	1.899815	2.784124	2.271069
9	6	0	2.561460	-1.068338	-0.391984
10	6	0	1.359999	-1.696740	-0.818354
11	6	0	3.670283	-1.292335	-1.175557
12	6	0	1.340761	-2.483252	-1.967540
13	6	0	3.633186	-2.052006	-2.340132
14	6	0	2.482791	-2.674161	-2.759736
15	1	0	0.423664	-2.970067	-2.275858
16	1	0	2.454068	-3.277211	-3.659375
17	8	0	4.278847	-0.735224	3.964641
18	8	0	4.028048	-1.905053	1.992639
19	8	0	4.918641	-0.790830	-0.992352
20	8	0	4.855413	-2.041785	-2.928884
21	6	0	4.578285	-1.973718	3.311427
22	1	0	4.110273	-2.795299	3.860002
23	1	0	5.662005	-2.097323	3.245681
24	6	0	5.720372	-1.299986	-2.062360
25	1	0	6.486724	-1.966312	-1.655836
26	1	0	6.161228	-0.467662	-2.615174
27	15	0	-0.180074	-1.333214	0.085729
28	15	0	1.042592	1.751152	-0.315904
29	46	0	-1.030927	0.806887	-0.213550
30	6	0	0.174515	-1.711626	1.825387
31	6	0	0.882738	-2.867608	2.172786
32	6	0	-0.208259	-0.800918	2.814106
33	6	0	1.207299	-3.104892	3.503863
34	1	0	1.196205	-3.569910	1.405765
35	6	0	0.131750	-1.037274	4.143011
36	1	0	-0.750653	0.100487	2.540144
37	6	0	0.842450	-2.185187	4.486478
38	6	0	-1.426949	-2.507570	-0.519102
39	6	0	-1.858428	-3.598253	0.240126
40	6	0	-1.980575	-2.279010	-1.785251
41	6	0	-2.827536	-4.456757	-0.271076
42	1	0	-1.448560	-3.780739	1.227824
43	6	0	-2.946551	-3.141655	-2.291014
44	1	0	-1.655749	-1.426345	-2.376557
45	6	0	-3.371085	-4.230960	-1.533105
46	6	0	0.906750	3.542856	0.005633
47	6	0	1.674606	4.477854	-0.693040
48	6	0	0.011204	3.980194	0.990924
49	6	0	1.547673	5.835024	-0.405238
50	1	0	2.365132	4.162889	-1.467512
51	6	0	-0.102772	5.334504	1.281135
52	1	0	-0.599410	3.261192	1.530642
53	6	0	0.664445	6.264221	0.580740
54	6	0	1.913168	1.528287	-1.892066
55	6	0	1.220852	1.020150	-2.993833
56	6	0	3.279942	1.813675	-1.993280
57	6	0	1.895603	0.787313	-4.188840
58	1	0	0.162728	0.786616	-2.908186
59	6	0	3.945169	1.591702	-3.193695
60	1	0	3.828782	2.184920	-1.132636
61	6	0	3.255860	1.070640	-4.287755
62	8	0	-3.133770	0.552503	0.124914
63	6	0	-4.026003	0.201047	-0.901425
64	6	0	-4.827650	-0.978039	-0.433283
65	6	0	-4.912875	-1.632469	0.753281
66	8	0	-5.698035	-1.516572	-1.329976
67	6	0	-5.906386	-2.653585	0.575983
68	1	0	-4.339696	-1.418001	1.643136
69	6	0	-6.343963	-2.537642	-0.701029
70	1	0	-6.240578	-3.377848	1.305207
71	1	0	-7.070748	-3.072826	-1.293121
72	6	0	-4.930560	1.383657	-1.261823
73	1	0	-5.677279	1.083824	-1.999494
74	1	0	-4.337284	2.209986	-1.659013
75	8	0	-5.661018	1.813069	-0.106874
76	6	0	-5.158921	2.813260	0.628513
77	8	0	-4.176286	3.459021	0.316563
78	1	0	-0.797033	5.663332	2.047763
79	1	0	0.568642	7.322960	0.800890
80	1	0	2.141909	6.555961	-0.957706
81	1	0	5.004855	1.813343	-3.270206
82	1	0	3.781900	0.881603	-5.218386
83	1	0	1.360061	0.377034	-5.039023
84	1	0	-0.151063	-0.320696	4.907502
85	1	0	1.113674	-2.364366	5.522310
86	1	0	1.756497	-4.001771	3.772415
87	1	0	-3.162810	-5.300427	0.323764
88	1	0	-4.132614	-4.898973	-1.923066
89	1	0	-3.375062	-2.955951	-3.270553
90	6	0	-5.964805	3.024282	1.874159
91	1	0	-7.030986	3.056492	1.638119
92	1	0	-5.797012	2.178364	2.548345
93	1	0	-5.656469	3.947171	2.364547
94	1	0	-3.509421	-0.076239	-1.832493
95	1	0	-1.801513	2.332219	-0.497100
96	1	0	-2.516500	1.786334	-0.239590

6c(*R*)$_{seg}$•2b

RwB97XD SCF energy -3187.977536 a.u.
RwB97XD` SCF enthalpy -3187.169557 a.u.
RwB97XD SCF free energy -3187.309597 a.u.
Three lowest frequencies (cm^{-1}) 11.3, 17.5, 26.1
Cartesian coordinates

Center Number	Atomic Number	Atomic Type	Coordinates (Angstroms) X	Y	Z
1	6	0	2.694623	-0.293831	0.749394
2	6	0	3.409190	-0.818925	1.803771
3	6	0	3.638666	-0.130365	2.989167
4	6	0	3.191162	1.155104	3.170536
5	6	0	2.486461	1.730397	2.102309
6	6	0	2.232491	1.042831	0.916461
7	1	0	3.369904	1.699006	4.090488
8	1	0	2.131349	2.747137	2.223397
9	6	0	2.477518	-1.161500	-0.442672
10	6	0	1.244092	-1.784253	-0.780315
11	6	0	3.545780	-1.451794	-1.261796
12	6	0	1.157042	-2.613835	-1.895134
13	6	0	3.439034	-2.255701	-2.390746
14	6	0	2.255705	-2.861744	-2.733659
15	1	0	0.217104	-3.095309	-2.137629
16	1	0	2.171190	-3.500650	-3.604811
17	8	0	4.326734	-0.920566	3.852032
18	8	0	3.956929	-2.060308	1.881276
19	8	0	4.819222	-0.990119	-1.144809
20	8	0	4.638724	-2.307782	-3.028502
21	6	0	4.557154	-2.160460	3.175108
22	1	0	4.086341	-2.973761	3.733199
23	1	0	5.632869	-2.319771	3.063287
24	6	0	5.564951	-1.593723	-2.205072
25	1	0	6.292378	-2.294725	-1.783401

26	1	0	6.052255	-0.816420	-2.797206
27	15	0	-0.241971	-1.324662	0.187046
28	15	0	1.159632	1.818635	-0.352212
29	46	0	-0.911550	0.953080	-0.227299
30	6	0	0.175717	-1.745013	1.908444
31	6	0	0.833352	-2.935490	2.237667
32	6	0	-0.120866	-0.816023	2.909965
33	6	0	1.191044	-3.188946	3.557785
34	1	0	1.078627	-3.656322	1.462815
35	6	0	0.250485	-1.067221	4.228304
36	1	0	-0.624314	0.112994	2.653191
37	6	0	0.910247	-2.250772	4.551007
38	6	0	-1.564119	-2.472599	-0.329801
39	6	0	-2.077586	-3.460100	0.514080
40	6	0	-2.134479	-2.289724	-1.596958
41	6	0	-3.134544	-4.262631	0.089042
42	1	0	-1.661149	-3.607330	1.505113
43	6	0	-3.180337	-3.101049	-2.023125
44	1	0	-1.760206	-1.508111	-2.253782
45	6	0	-3.682738	-4.089737	-1.178552
46	6	0	1.242618	3.612928	-0.002046
47	6	0	2.179765	4.455179	-0.605115
48	6	0	0.344756	4.143071	0.933663
49	6	0	2.215250	5.808759	-0.276050
50	1	0	2.877639	4.073958	-1.341812
51	6	0	0.394139	5.490866	1.270999
52	1	0	-0.397447	3.498783	1.397327
53	6	0	1.328808	6.327199	0.663185
54	6	0	1.998951	1.517597	-1.938025
55	6	0	1.259742	1.031053	-3.020199
56	6	0	3.381506	1.696367	-2.065276
57	6	0	1.898549	0.721287	-4.217263
58	1	0	0.189769	0.872349	-2.913326
59	6	0	4.012103	1.403681	-3.269481
60	1	0	3.971886	2.034727	-1.218828
61	6	0	3.273474	0.908029	-4.342524
62	8	0	-2.993604	0.423328	0.106769
63	6	0	-3.943495	0.466721	-0.958012
64	6	0	-4.982130	-0.581171	-0.708497
65	6	0	-5.374880	-1.240708	0.410757
66	8	0	-5.735692	-0.956362	-1.773137
67	6	0	-6.452036	-2.097010	0.004597
68	1	0	-4.947707	-1.136753	1.397430
69	6	0	-6.624831	-1.885204	-1.322881
70	1	0	-7.015025	-2.780448	0.623905
71	1	0	-7.299766	-2.288809	-2.062226
72	6	0	-4.538635	1.863361	-1.147959
73	1	0	-4.975209	1.931082	-2.143945
74	1	0	-3.754498	2.619996	-1.044980
75	8	0	-5.637419	2.178019	-0.271845
76	6	0	-5.415521	2.353829	1.026008
77	8	0	-4.333854	2.143890	1.561871
78	1	0	-0.304771	5.888067	2.000482
79	1	0	1.361155	7.382523	0.915996
80	1	0	2.939481	6.457126	-0.759190
81	1	0	5.083586	1.547836	-3.364730
82	1	0	3.771612	0.663493	-5.275586
83	1	0	1.323269	0.328024	-5.049413
84	1	0	0.032055	-0.335302	4.999527
85	1	0	1.204323	-2.444740	5.577953
86	1	0	1.699744	-4.114294	3.809773
87	1	0	-3.528799	-5.023942	0.755081
88	1	0	-4.508574	-4.714462	-1.504229
89	1	0	-3.615766	-2.948536	-3.005532
90	6	0	-6.628421	2.843085	1.746466
91	1	0	-7.498245	2.247120	1.460620
92	1	0	-6.466855	2.789393	2.822202
93	1	0	-6.821671	3.880248	1.455734
94	1	0	-3.403543	0.236058	-1.880654
95	1	0	-1.313699	2.411228	-0.630707
96	1	0	-3.329772	1.035591	0.803125

---:

2.14. **Catalytic cycle for destructive hydrogenation with 2a**

Acetone

RwB97XD SCF energy	-193.109404 a.u.
RwB97XD` SCF enthalpy	-193.018624a.u.
RwB97XD SCF free energy	-193.051893 a.u.
Three lowest frequencies (cm^{-1})	161.5, 182.5, 384.0

Standard orientation:

Center Number	Atomic Number	Atomic Type	Coordinates (Angstroms) X	Y	Z
1	6	0	-0.000002	0.183999	-0.000002
2	8	0	-0.000003	1.400929	0.000000
3	6	0	-1.281461	-0.613887	-0.002610
4	1	0	-2.139041	0.041420	-0.162865
5	1	0	-1.251023	-1.390083	-0.773814
6	1	0	-1.389983	-1.123754	0.961529
7	6	0	1.281462	-0.613883	0.002608
8	1	0	1.251042	-1.390057	0.773836
9	1	0	1.389991	-1.123765	-0.961519
10	1	0	2.139038	0.041434	0.162856

7

RwB97XD SCF energy	-4485.238995 a.u.
RwB97XD` SCF enthalpy	-4483.434913 a.u.
RwB97XD SCF free energy	-4483.660677 a.u.
Three lowest frequencies (cm^{-1})	11.4, 14.3, 18,1

Cartesian coordinates

Standard orientation:

Center Number	Atomic Number	Atomic Type	Coordinates (Angstroms) X	Y	Z
1	6	0	-0.170687	1.348651	2.045798
2	6	0	-0.591992	2.372736	2.868323
3	6	0	-0.002866	3.633909	2.875537
4	6	0	1.024917	3.955940	2.023990
5	6	0	1.466719	2.937515	1.165382
6	6	0	0.906956	1.661507	1.171402
7	1	0	1.487905	4.935547	2.027724
8	1	0	2.291366	3.158782	0.498243
9	6	0	-0.726216	-0.017098	2.269119
10	6	0	-1.474962	-0.826775	1.366879
11	6	0	-0.485120	-0.570398	3.510757
12	6	0	-1.886259	-2.102874	1.755158
13	6	0	-0.900824	-1.844397	3.881832
14	6	0	-1.600541	-2.646891	3.015746
15	1	0	-2.466739	-2.710094	1.072678
16	1	0	-1.930213	-3.641859	3.289966
17	8	0	-0.596436	4.405231	3.824825
18	8	0	-1.583877	2.334474	3.799184
19	8	0	0.206736	-0.008271	4.541310
20	8	0	-0.487486	-2.105832	5.150245
21	6	0	-1.394388	3.502497	4.592518
22	1	0	-2.358473	3.962484	4.809724
23	1	0	-0.858007	3.236240	5.512418
24	6	0	-0.056220	-0.844754	5.665451
25	1	0	-0.859878	-0.403597	6.268440
26	1	0	0.857892	-0.978123	6.244231
27	15	0	-1.859807	-0.241469	-0.334634
28	15	0	1.495445	0.383324	0.006439
29	46	0	-0.059298	-0.265182	-1.691790
30	6	0	-2.508930	1.435294	-0.116687
31	6	0	-3.511309	1.719397	0.802365
32	6	0	-1.946948	2.468408	-0.864845
33	6	0	-3.956670	3.027407	1.021324
34	1	0	-3.959074	0.903819	1.361439
35	6	0	-2.286674	3.798464	-0.640689
36	1	0	-1.201292	2.212242	-1.607654
37	6	0	-3.241230	4.056526	0.372462
38	6	0	-3.185134	-1.343171	-0.929443
39	6	0	-4.511274	-0.949606	-1.081725
40	6	0	-2.802286	-2.617877	-1.332854
41	6	0	-5.476479	-1.842213	-1.555018
42	1	0	-4.789690	0.068593	-0.842811
43	6	0	-3.716919	-3.566454	-1.792168
44	1	0	-1.749642	-2.877233	-1.284785
45	6	0	-5.072779	-3.176439	-1.799362

46	6	0	2.998945	1.037340	-0.782086
47	6	0	4.235692	0.422920	-0.611954
48	6	0	2.867413	2.041937	-1.731750
49	6	0	5.363221	0.850450	-1.308595
50	1	0	4.315636	-0.408824	0.073289
51	6	0	3.953893	2.523860	-2.465550
52	1	0	1.879399	2.449624	-1.918382
53	6	0	5.216718	1.971191	-2.161215
54	6	0	2.085790	-1.013657	0.996976
55	6	0	1.969422	-2.276289	0.436959
56	6	0	2.724168	-0.861434	2.226758
57	6	0	2.551468	-3.407466	1.018666
58	1	0	1.413323	-2.375823	-0.490149
59	6	0	3.340306	-1.941011	2.852940
60	1	0	2.743327	0.120469	2.683544
61	6	0	3.329115	-3.185853	2.170997
62	6	0	2.259737	-4.767285	0.341951
63	6	0	3.035938	-4.864397	-0.985112
64	1	0	2.769854	-5.788286	-1.510779
65	1	0	2.800793	-4.021684	-1.643459
66	1	0	4.117833	-4.870602	-0.821827
67	6	0	2.568561	-6.001744	1.207332
68	1	0	2.098447	-5.927892	2.191923
69	1	0	2.162392	-6.886024	0.704463
70	1	0	3.635012	-6.172019	1.351429
71	6	0	0.745997	-4.841669	0.026974
72	1	0	0.149955	-4.704116	0.935544
73	1	0	0.422958	-4.097758	-0.706705
74	1	0	0.508484	-5.824474	-0.392454
75	6	0	3.928313	-1.790105	4.271165
76	6	0	3.206259	-2.784839	5.201993
77	1	0	3.407659	-3.817347	4.909176
78	1	0	3.546618	-2.647551	6.234611
79	1	0	2.123101	-2.628303	5.172853
80	6	0	5.446464	-2.044355	4.323576
81	1	0	5.975237	-1.446175	3.573458
82	1	0	5.828577	-1.755266	5.308887
83	1	0	5.691739	-3.095383	4.171990
84	6	0	3.691077	-0.373783	4.821588
85	1	0	4.242788	0.383851	4.254035
86	1	0	2.631817	-0.101035	4.819121
87	1	0	4.047725	-0.331454	5.855533
88	8	0	4.097959	-4.197016	2.690487
89	8	0	6.355106	2.499895	-2.718530
90	8	0	-3.475348	5.365331	0.706789
91	8	0	-6.053520	-4.091744	-2.088352
92	6	0	-6.412104	-4.904999	-0.976195
93	1	0	-7.273980	-5.498461	-1.286993
94	1	0	-5.599123	-5.578266	-0.687145
95	1	0	-6.684187	-4.294402	-0.108450
96	6	0	-3.141641	-4.922442	-2.268075
97	6	0	-4.084457	-5.749314	-3.160492
98	1	0	-3.517583	-6.590873	-3.572948
99	1	0	-4.936165	-6.164356	-2.623166
100	1	0	-4.464975	-5.157304	-3.997637
101	6	0	-2.722964	-5.758634	-1.044310
102	1	0	-2.240952	-6.687485	-1.369592
103	1	0	-2.011657	-5.209698	-0.418456
104	1	0	-3.581414	-6.026837	-0.421200
105	6	0	5.330793	-4.370656	1.999354
106	1	0	5.891237	-5.132189	2.544968
107	1	0	5.174594	-4.707857	0.969776
108	1	0	5.908563	-3.439950	1.976572
109	6	0	6.810192	3.671744	-2.050176
110	1	0	7.740675	3.969111	-2.537470
111	1	0	6.085819	4.489137	-2.127050
112	1	0	7.000401	3.475633	-0.989332
113	6	0	6.676978	0.046250	-1.204941
114	6	0	6.507797	-1.189771	-0.300934
115	1	0	6.292259	-0.914116	0.737398
116	1	0	5.714741	-1.859156	-0.651995
117	1	0	7.442572	-1.759095	-0.302508
118	6	0	7.055328	-0.457880	-2.611736
119	1	0	7.212912	0.373845	-3.301620
120	1	0	7.979926	-1.044144	-2.563500
121	1	0	6.267569	-1.102275	-3.017791
122	6	0	7.839592	0.874043	-0.626492
123	1	0	8.183864	1.635290	-1.326777
124	1	0	7.552282	1.360896	0.311602
125	1	0	8.687748	0.213839	-0.414192
126	6	0	3.635327	3.569550	-3.562062
127	6	0	3.237780	4.900498	-2.896822
128	1	0	2.377521	4.772335	-2.231036
129	1	0	4.060883	5.316152	-2.306610
130	1	0	2.964066	5.636973	-3.660683
131	6	0	4.762395	3.825151	-4.578411
132	1	0	4.358539	4.441536	-5.388925
133	1	0	5.612209	4.362083	-4.157852
134	1	0	5.129272	2.892611	-5.015537
135	6	0	2.435763	3.057461	-4.394176
136	1	0	2.206085	3.781762	-5.182665
137	1	0	2.671837	2.100123	-4.870801
138	1	0	1.526484	2.924915	-3.803760
139	6	0	-6.901873	-1.350577	-1.890461
140	6	0	-1.879613	-4.654923	-3.121855
141	1	0	-2.106501	-3.990963	-3.962775
142	1	0	-1.062738	-4.213154	-2.546750
143	1	0	-1.507624	-5.601930	-3.525846
144	6	0	-7.176111	-1.616552	-3.384348
145	1	0	-8.177540	-1.257280	-3.647060
146	1	0	-6.451856	-1.085425	-4.011612
147	1	0	-7.123199	-2.681876	-3.617081
148	6	0	-7.987580	-2.033435	-1.036910
149	1	0	-7.743590	-1.984482	0.029835
150	1	0	-8.942535	-1.516970	-1.184107
151	1	0	-8.133385	-3.076498	-1.316853
152	6	0	-7.039056	0.162231	-1.658957
153	1	0	-8.036656	0.481187	-1.976444
154	1	0	-6.933891	0.419667	-0.601560
155	6	0	-2.848745	5.746884	1.927705
156	1	0	-3.137071	6.782430	2.118065
157	1	0	-3.175737	5.119898	2.763387
158	1	0	-1.760038	5.679400	1.848914
159	6	0	-5.215296	3.200089	1.907895
160	6	0	-4.894137	2.848372	3.370160
161	1	0	-5.810962	2.877195	3.969512
162	1	0	-4.457229	1.849713	3.462311
163	1	0	-4.189646	3.564547	3.801747
164	6	0	-5.872178	4.591729	1.860258
165	1	0	-6.840446	4.527552	2.368648
166	1	0	-5.292559	5.362460	2.367927
167	1	0	-6.052538	4.918931	0.833064
168	6	0	-6.290654	2.219586	1.387825
169	1	0	-6.536639	2.439843	0.344091
170	1	0	-5.981767	1.172973	1.453659
171	1	0	-7.204701	2.326682	1.981026
172	6	0	-1.685342	4.912330	-1.523392
173	6	0	-0.709995	4.333141	-2.562390
174	1	0	-0.330297	5.145700	-3.189482
175	1	0	0.153047	3.858530	-2.082221
176	1	0	-1.185309	3.602004	-3.224630
177	6	0	-0.896190	5.955925	-0.710147
178	1	0	-0.179563	5.472200	-0.037916
179	1	0	-0.332627	6.599124	-1.395014
180	1	0	-1.552745	6.597308	-0.122396
181	6	0	-2.829133	5.610004	-2.284389
182	1	0	-3.365500	4.896773	-2.919661
183	1	0	-3.542809	6.064885	-1.593653
184	1	0	-2.423778	6.398849	-2.928107
185	8	0	1.532255	-0.372469	-3.128043
186	6	0	1.946736	-1.368597	-3.726942
187	6	0	1.205810	-2.666902	-3.678522
188	1	0	0.957684	-2.907629	-2.641116
189	1	0	0.258802	-2.529929	-4.214242
190	1	0	1.764324	-3.485758	-4.133209
191	6	0	3.205086	-1.272787	-4.521514
192	1	0	3.920020	-2.005587	-4.130685
193	1	0	3.002262	-1.553291	-5.560964
194	1	0	3.628463	-0.269224	-4.470058
195	1	0	-1.119907	-0.630542	-2.791921
196	1	0	-6.309409	0.742111	-2.233747

TS3

RwB97XD SCF energy	-4485.225774 a.u.
RwB97XD` SCF enthalpy	-4483.423888 a.u.
RwB97XD SCF free energy	-4483.647177 a.u.
Three lowest frequencies (cm^{-1})	-490.3, 9.5, 13.5
Imaginary frequency (cm^{-1})	-490.3

Standard orientation:

Center Number	Atomic Number	Atomic Type	Coordinates (Angstroms) X	Y	Z

1	6	0	-0.273192	-0.904615	2.181984
2	6	0	-0.057214	-1.837214	3.175004
3	6	0	-0.715200	-3.062936	3.225366
4	6	0	-1.616226	-3.436335	2.258583
5	6	0	-1.861738	-2.503949	1.239319
6	6	0	-1.229249	-1.262110	1.192807
7	1	0	-2.131061	-4.389286	2.289155
8	1	0	-2.589844	-2.765063	0.480717
9	6	0	0.392827	0.420664	2.303742
10	6	0	1.336522	0.999702	1.408758
11	6	0	0.085166	1.170154	3.420334
12	6	0	1.866180	2.262928	1.674401
13	6	0	0.621002	2.428825	3.672312
14	6	0	1.514627	3.011258	2.807555
15	1	0	2.590163	2.696596	0.996322
16	1	0	1.940057	3.990879	2.990318
17	8	0	-0.315276	-3.744416	4.331432
18	8	0	0.782714	-1.734530	4.240396
19	8	0	-0.784413	0.845622	4.416515
20	8	0	0.106804	2.915404	4.832776
21	6	0	0.377182	-2.776751	5.123523
22	1	0	1.251585	-3.235030	5.582825
23	1	0	-0.308036	-2.368282	5.877369
24	6	0	-0.530542	1.794045	5.450253
25	1	0	0.148115	1.353429	6.191356
26	1	0	-1.472419	2.106554	5.900594
27	15	0	1.798980	0.143758	-0.146204
28	15	0	-1.582973	-0.109905	-0.183605
29	46	0	0.161952	0.142428	-1.726904
30	6	0	2.207555	-1.563292	0.301019
31	6	0	3.022765	-1.890945	1.377621
32	6	0	1.661703	-2.579217	-0.481761
33	6	0	3.270290	-3.223897	1.727666
34	1	0	3.477662	-1.093439	1.957250
35	6	0	1.806218	-3.919858	-0.145472
36	1	0	1.079747	-2.296984	-1.351067
37	6	0	2.539746	-4.210162	1.029039
38	6	0	3.307529	0.973893	-0.741556
39	6	0	4.564036	0.376563	-0.699461
40	6	0	3.165528	2.223380	-1.336177
41	6	0	5.695104	1.043206	-1.173876
42	1	0	4.656718	-0.626173	-0.303284
43	6	0	4.258573	2.959959	-1.799497
44	1	0	2.168576	2.641793	-1.428159
45	6	0	5.531477	2.376947	-1.620474
46	6	0	-3.078492	-0.768173	-0.979687
47	6	0	-4.337759	-0.217590	-0.759020
48	6	0	-2.947706	-1.836165	-1.857432
49	6	0	-5.482424	-0.771851	-1.326003
50	1	0	-4.420955	0.655773	-0.128296
51	6	0	-4.051712	-2.441698	-2.464972
52	1	0	-1.953211	-2.212626	-2.068187
53	6	0	-5.322567	-1.946241	-2.102668
54	6	0	-2.075062	1.475462	0.541217
55	6	0	-1.681365	2.631566	-0.115039
56	6	0	-2.875000	1.569604	1.680910
57	6	0	-2.128857	3.898276	0.275281
58	1	0	-1.005525	2.543738	-0.960771
59	6	0	-3.387502	2.794249	2.098643
60	1	0	-3.109162	0.664756	2.228186
61	6	0	-3.080517	3.934246	1.310741
62	6	0	-1.504935	5.108247	-0.457167
63	6	0	-1.990276	5.139861	-1.918231
64	1	0	-1.521914	5.975303	-2.450622
65	1	0	-1.728244	4.215989	-2.445427
66	1	0	-3.075769	5.265713	-1.979392
67	6	0	-1.763757	6.476902	0.196045
68	1	0	-1.506972	6.470393	1.258729
69	1	0	-1.128671	7.218647	-0.300654
70	1	0	-2.794974	6.815198	0.095914
71	6	0	0.030679	4.927100	-0.456223
72	1	0	0.419415	4.885482	0.567011
73	1	0	0.351824	4.021440	-0.977779
74	1	0	0.496935	5.776797	-0.965337
75	6	0	-4.191123	2.899685	3.410858
76	6	0	-3.488631	3.910706	4.338728
77	1	0	-3.516238	4.918016	3.919155
78	1	0	-3.984057	3.930761	5.315947
79	1	0	-2.439985	3.636449	4.490418
80	6	0	-5.648558	3.340787	3.178617
81	1	0	-6.134385	2.721767	2.416235
82	1	0	-6.214929	3.230000	4.110069
83	1	0	-5.716475	4.385817	2.875938
84	6	0	-4.237709	1.546979	4.141135
85	1	0	-4.796775	0.793841	3.574598
86	1	0	-3.236740	1.154150	4.343711
87	1	0	-4.747852	1.679318	5.100425
88	8	0	-3.738116	5.102494	1.602683
89	8	0	-6.461700	-2.585694	-2.524172
90	8	0	2.536626	-5.506693	1.473527
91	8	0	6.668502	3.083803	-1.919893
92	6	0	7.084110	3.970590	-0.885222
93	1	0	8.090960	4.303900	-1.144246
94	1	0	6.429434	4.844217	-0.812094
95	1	0	7.106387	3.466246	0.086442
96	6	0	3.956517	4.329073	-2.455017
97	6	0	5.078182	4.876679	-3.356391
98	1	0	4.688581	5.742195	-3.903029
99	1	0	5.955872	5.210799	-2.805177
100	1	0	5.399577	4.132575	-4.090970
101	6	0	3.627509	5.351222	-1.350672
102	1	0	3.346378	6.312003	-1.796637
103	1	0	2.791172	5.005156	-0.735167
104	1	0	4.480388	5.523673	-0.687859
105	6	0	-4.811026	5.373031	0.704874
106	1	0	-5.312968	6.267866	1.077621
107	1	0	-4.452674	5.558181	-0.312900
108	1	0	-5.523106	4.540637	0.676388
109	6	0	-6.793167	-3.724896	-1.736246
110	1	0	-7.768896	-4.071821	-2.081882
111	1	0	-6.062750	-4.530660	-1.858715
112	1	0	-6.853375	-3.469175	-0.672969
113	6	0	-6.842110	-0.058806	-1.158785
114	6	0	-6.694109	1.242888	-0.349058
115	1	0	-6.368513	1.050789	0.679537
116	1	0	-5.989552	1.942828	-0.810483
117	1	0	-7.667395	1.740894	-0.298342
118	6	0	-7.375851	0.319798	-2.554209
119	1	0	-7.528982	-0.566546	-3.173620
120	1	0	-8.334761	0.841759	-2.458117
121	1	0	-6.676990	0.988282	-3.069018
122	6	0	-7.884753	-0.924891	-0.427161
123	1	0	-8.234595	-1.752237	-1.044489
124	1	0	-7.481152	-1.327787	0.508046
125	1	0	-8.756365	-0.310016	-0.176853
126	6	0	-3.750883	-3.574612	-3.476680
127	6	0	-3.295095	-4.831793	-2.711484
128	1	0	-2.417217	-4.619335	-2.092362
129	1	0	-4.083140	-5.215170	-2.055994
130	1	0	-3.025572	-5.626304	-3.416665
131	6	0	-4.913809	-3.936188	-4.417943
132	1	0	-4.529934	-4.605653	-5.195496
133	1	0	-5.731339	-4.456590	-3.920314
134	1	0	-5.321997	-3.048761	-4.909624
135	6	0	-2.593263	-3.122781	-4.397934
136	1	0	-2.394902	-3.904079	-5.138973
137	1	0	-2.852580	-2.203224	-4.932896
138	1	0	-1.660681	-2.947939	-3.857830
139	6	0	7.045512	0.302020	-1.286138
140	6	0	2.718515	4.193092	-3.370755
141	1	0	2.884950	3.437343	-4.145113
142	1	0	1.806273	3.934259	-2.828176
143	1	0	2.530697	5.150672	-3.866878
144	6	0	7.492464	0.309950	-2.761605
145	1	0	8.441349	-0.228661	-2.864204
146	1	0	6.750730	-0.190486	-3.394034
147	1	0	7.635048	1.327424	-3.130692
148	6	0	8.146886	0.926563	-0.408559
149	1	0	7.801669	1.067084	0.621424
150	1	0	9.012462	0.255494	-0.381373
151	1	0	8.488649	1.884961	-0.798506
152	6	0	6.911504	-1.166547	-0.853268
153	1	0	7.868642	-1.672642	-1.013059
154	1	0	6.667571	-1.253346	0.209271
155	6	0	1.682311	-5.705723	2.596558
156	1	0	1.771342	-6.757531	2.874716
157	1	0	1.973956	-5.079303	3.444551
158	1	0	0.641146	-5.482094	2.342211
159	6	0	4.360759	-3.481525	2.797147
160	6	0	3.888602	-3.001316	4.178989
161	1	0	4.710855	-3.075642	4.899457
162	1	0	3.544672	-1.963473	4.156100
163	1	0	3.067787	-3.623142	4.546469

164	6	0	4.834106	-4.941489	2.914885
165	1	0	5.719728	-4.960308	3.559628
166	1	0	4.091940	-5.600642	3.365176
167	1	0	5.116934	-5.355432	1.943348
168	6	0	5.612978	-2.672698	2.388908
169	1	0	5.974240	-2.997602	1.407904
170	1	0	5.432784	-1.595279	2.348844
171	1	0	6.413000	-2.842314	3.117046
172	6	0	1.218642	-5.013674	-1.060427
173	6	0	0.610629	-4.401192	-2.334498
174	1	0	0.237380	-5.205907	-2.975511
175	1	0	-0.235869	-3.743052	-2.111117
176	1	0	1.348822	-3.833210	-2.910914
177	6	0	0.102489	-5.811714	-0.361553
178	1	0	-0.645798	-5.142055	0.076087
179	1	0	-0.403183	-6.452103	-1.092863
180	1	0	0.496084	-6.457216	0.424419
181	6	0	2.344322	-5.969117	-1.500865
182	1	0	3.124227	-5.425036	-2.045083
183	1	0	2.802025	-6.466904	-0.643976
184	1	0	1.939388	-6.737091	-2.169525
185	8	0	-0.968126	0.184579	-3.490696
186	6	0	0.168176	0.206423	-4.096801
187	6	0	0.609592	1.498119	-4.748044
188	1	0	0.318616	2.356846	-4.140784
189	1	0	1.686117	1.512019	-4.926811
190	1	0	0.098317	1.560121	-5.716424
191	6	0	0.674069	-1.078934	-4.716565
192	1	0	0.158982	-1.199464	-5.677301
193	1	0	1.749093	-1.039776	-4.903434
194	1	0	0.436558	-1.935153	-4.082962
195	1	0	1.310562	0.269709	-2.855274
196	1	0	6.153073	-1.705971	-1.430385

8

RwB97XD SCF energy -4485.232658 a.u.

RwB97XD` SCF enthalpy -4483.426630 a.u.

RwB97XD SCF free energy -4483.650204 a.u.

Three lowest frequencies (cm^{-1}) 11.5, 16.1, 20.3

Cartesian coordinates

Standard orientation:

Center Number	Atomic Number	Atomic Type	Coordinates (Angstroms) X	Y	Z
1	6	0	-0.250414	-0.974313	2.185833
2	6	0	0.007018	-1.940103	3.136114
3	6	0	-0.572952	-3.206020	3.113310
4	6	0	-1.429229	-3.588458	2.110402
5	6	0	-1.703310	-2.630266	1.124000
6	6	0	-1.148236	-1.350998	1.147547
7	1	0	-1.886162	-4.570545	2.087545
8	1	0	-2.393084	-2.903810	0.335400
9	6	0	0.336523	0.378320	2.384908
10	6	0	1.258723	1.038264	1.526477
11	6	0	-0.010644	1.061004	3.531480
12	6	0	1.737451	2.307116	1.847577
13	6	0	0.469698	2.330222	3.839670
14	6	0	1.345582	2.987630	3.010599
15	1	0	2.450047	2.795175	1.193907
16	1	0	1.727960	3.974352	3.243346
17	8	0	-0.153715	-3.915769	4.190368
18	8	0	0.825305	-1.840403	4.217216
19	8	0	-0.868033	0.655163	4.506861
20	8	0	-0.073883	2.744821	5.014727
21	6	0	0.504392	-2.965016	5.032271
22	1	0	1.416568	-3.403351	5.435185
23	1	0	-0.180513	-2.654293	5.831062
24	6	0	-0.670263	1.574473	5.579465
25	1	0	0.015777	1.135113	6.314401
26	1	0	-1.631677	1.829149	6.025221
27	15	0	1.736487	0.239587	-0.046076
28	15	0	-1.550773	-0.177567	-0.193602
29	46	0	0.126530	0.228264	-1.673899
30	6	0	2.278730	-1.432764	0.380492
31	6	0	3.162040	-1.680484	1.422819
32	6	0	1.792785	-2.493971	-0.379921
33	6	0	3.542250	-2.982458	1.765706
34	1	0	3.568012	-0.840483	1.978216
35	6	0	2.077446	-3.814813	-0.052656
36	1	0	1.149618	-2.270027	-1.222061
37	6	0	2.885833	-4.033696	1.088872
38	6	0	3.168419	1.145512	-0.706084
39	6	0	4.420209	0.550104	-0.845474
40	6	0	2.963349	2.427211	-1.207282
41	6	0	5.495588	1.250563	-1.390277
42	1	0	4.549788	-0.476138	-0.528313
43	6	0	4.002610	3.193527	-1.741888
44	1	0	1.963973	2.848148	-1.168165
45	6	0	5.287146	2.610641	-1.726842
46	6	0	-2.978033	-0.881669	-1.062564
47	6	0	-4.255234	-0.352029	-0.905843
48	6	0	-2.782615	-1.954136	-1.923638
49	6	0	-5.360791	-0.936256	-1.518023
50	1	0	-4.384773	0.526999	-0.291025
51	6	0	-3.846240	-2.594564	-2.564918
52	1	0	-1.770782	-2.304406	-2.091998
53	6	0	-5.142612	-2.119513	-2.265987
54	6	0	-2.129751	1.352238	0.579703
55	6	0	-1.803551	2.562384	-0.012079
56	6	0	-2.947421	1.334493	1.711670
57	6	0	-2.334351	3.775352	0.438748
58	1	0	-1.117058	2.561020	-0.852712
59	6	0	-3.545199	2.499949	2.180687
60	1	0	-3.128914	0.390219	2.210332
61	6	0	-3.303580	3.695085	1.455034
62	6	0	-1.773116	5.061572	-0.209379
63	6	0	-2.218443	5.141346	-1.681140
64	1	0	-1.793336	6.034102	-2.153266
65	1	0	-1.881434	4.267928	-2.249991
66	1	0	-3.308248	5.199124	-1.767991
67	6	0	-2.140757	6.373855	0.504961
68	1	0	-1.913187	6.328333	1.573250
69	1	0	-1.541994	7.179628	0.066369
70	1	0	-3.188550	6.650847	0.389408
71	6	0	-0.229799	4.979766	-0.164256
72	1	0	0.128644	4.918911	0.869087
73	1	0	0.165808	4.119585	-0.710210
74	1	0	0.196419	5.878214	-0.622242
75	6	0	-4.370942	2.489024	3.483058
76	6	0	-3.748855	3.499826	4.467039
77	1	0	-3.837103	4.521817	4.093698
78	1	0	-4.257009	3.441437	5.436088
79	1	0	-2.686611	3.286927	4.622761
80	6	0	-5.851368	2.842804	3.247391
81	1	0	-6.286368	2.223600	2.455093
82	1	0	-6.419725	2.658126	4.165803
83	1	0	-5.984433	3.892306	2.984138
84	6	0	-4.334886	1.103547	4.149134
85	1	0	-4.833271	0.341904	3.539072
86	1	0	-3.311972	0.771740	4.351375
87	1	0	-4.866573	1.155861	5.104392
88	8	0	-4.046118	4.798866	1.786566
89	8	0	-6.250246	-2.785007	-2.726853
90	8	0	3.032030	-5.325430	1.523854
91	8	0	6.387301	3.350674	-2.076799
92	6	0	6.876603	4.170036	-1.018645
93	1	0	7.815770	4.603019	-1.368050
94	1	0	6.179000	4.977015	-0.773643
95	1	0	7.060984	3.581656	-0.113507
96	6	0	3.626652	4.587884	-2.297674
97	6	0	4.676178	5.227962	-3.224155
98	1	0	4.227516	6.111821	-3.690427
99	1	0	5.572822	5.558633	-2.701444
100	1	0	4.978018	4.543751	-4.022095
101	6	0	3.332518	5.535818	-1.120196
102	1	0	2.994832	6.508965	-1.494095
103	1	0	2.547841	5.130294	-0.473559
104	1	0	4.221983	5.701092	-0.504599
105	6	0	-5.109450	5.041321	0.868686
106	1	0	-5.673633	5.889645	1.260366
107	1	0	-4.734476	5.286470	-0.130322
108	1	0	-5.768906	4.169614	0.788752
109	6	0	-6.605160	-3.915409	-1.936062
110	1	0	-7.576577	-4.257971	-2.297685
111	1	0	-5.879222	-4.727551	-2.039488
112	1	0	-6.683873	-3.650410	-0.876357
113	6	0	-6.739911	-0.247717	-1.423644
114	6	0	-6.650351	1.075852	-0.641162
115	1	0	-6.366776	0.916362	0.405356
116	1	0	-5.939386	1.777297	-1.090350

117	1	0	-7.633809	1.556052	-0.645167
118	6	0	-7.224055	0.087298	-2.847981
119	1	0	-7.333752	-0.816004	-3.451749
120	1	0	-8.196272	0.591137	-2.802493
121	1	0	-6.518439	0.758575	-3.349727
122	6	0	-7.792708	-1.118869	-0.712953
123	1	0	-8.100317	-1.968130	-1.322990
124	1	0	-7.418462	-1.490744	0.246971
125	1	0	-8.686269	-0.517435	-0.512413
126	6	0	-3.482668	-3.744815	-3.536208
127	6	0	-3.076551	-4.989792	-2.724018
128	1	0	-2.238140	-4.767750	-2.055221
129	1	0	-3.902872	-5.364737	-2.112693
130	1	0	-2.764236	-5.794361	-3.399466
131	6	0	-4.589106	-4.118696	-4.539261
132	1	0	-4.164030	-4.803860	-5.280659
133	1	0	-5.438139	-4.624957	-4.081860
134	1	0	-4.961487	-3.237854	-5.070071
135	6	0	-2.268839	-3.314896	-4.393678
136	1	0	-2.050046	-4.098153	-5.126561
137	1	0	-2.479153	-2.388450	-4.937848
138	1	0	-1.359463	-3.168457	-3.805902
139	6	0	6.825911	0.525362	-1.687199
140	6	0	2.346015	4.453043	-3.153285
141	1	0	2.505612	3.758979	-3.984653
142	1	0	1.478653	4.111059	-2.583780
143	1	0	2.085694	5.430090	-3.573010
144	6	0	7.139042	0.660268	-3.190918
145	1	0	8.071287	0.133496	-3.423930
146	1	0	6.340575	0.214588	-3.794631
147	1	0	7.254217	1.705763	-3.483053
148	6	0	8.002246	1.079632	-0.861865
149	1	0	7.753900	1.121110	0.204208
150	1	0	8.869439	0.420117	-0.977164
151	1	0	8.301264	2.074489	-1.192222
152	6	0	6.723103	-0.976409	-1.373672
153	1	0	7.660350	-1.463335	-1.660432
154	1	0	6.570781	-1.161713	-0.306038
155	6	0	2.232893	-5.615802	2.667432
156	1	0	2.436591	-6.654046	2.936235
157	1	0	2.484452	-4.966466	3.511794
158	1	0	1.167964	-5.498307	2.443961
159	6	0	4.676239	-3.125200	2.810581
160	6	0	4.185785	-2.665342	4.194089
161	1	0	5.018869	-2.674176	4.905859
162	1	0	3.772771	-1.652889	4.165132
163	1	0	3.411939	-3.335516	4.578616
164	6	0	5.283918	-4.533419	2.939782
165	1	0	6.183575	-4.459444	3.560606
166	1	0	4.617846	-5.249166	3.421755
167	1	0	5.578494	-4.938050	1.968037
168	6	0	5.841789	-2.211863	2.365152
169	1	0	6.223254	-2.528241	1.388838
170	1	0	5.560344	-1.157883	2.298262
171	1	0	6.661392	-2.285758	3.087522
172	6	0	1.571051	-4.959406	-0.954602
173	6	0	0.803185	-4.409107	-2.169652
174	1	0	0.486632	-5.244949	-2.801173
175	1	0	-0.099361	-3.865219	-1.869329
176	1	0	1.420346	-3.746649	-2.786116
177	6	0	0.615720	-5.914550	-0.215490
178	1	0	-0.182951	-5.361011	0.289415
179	1	0	0.149443	-6.594297	-0.937290
180	1	0	1.139133	-6.525202	0.520475
181	6	0	2.783137	-5.748945	-1.485050
182	1	0	3.447578	-5.098348	-2.064422
183	1	0	3.356143	-6.190710	-0.666946
184	1	0	2.443366	-6.557109	-2.142582
185	8	0	-0.939543	0.298734	-3.384832
186	6	0	0.256586	0.441481	-4.026808
187	6	0	0.389891	1.764587	-4.776341
188	1	0	0.141859	2.600694	-4.117753
189	1	0	1.402271	1.902636	-5.166397
190	1	0	-0.310479	1.762036	-5.618440
191	6	0	0.654397	-0.776951	-4.858732
192	1	0	-0.038498	-0.874149	-5.701118
193	1	0	1.670154	-0.674698	-5.252633
194	1	0	0.597833	-1.685954	-4.253632
195	1	0	1.137167	0.509945	-3.206050
196	1	0	5.914837	-1.463387	-1.929547

9a(DTBM-Segphos)

RwB97XD SCF energy	-5213.551305 a.u.
RwB97XD SCF enthalpy	-5211.485047 a.u.
RwB97XD SCF free energy	-5211.736837 a.u.
Three lowest frequencies (cm^{-1})	13.4, 17.8, 21.3

Cartesian coordinates

Standard orientation:

Center Number	Atomic Number	Atomic Type	Coordinates (Angstroms) X	Y	Z
1	6	0	-0.836715	-0.501342	2.725579
2	6	0	-0.512047	-1.207236	3.864585
3	6	0	-0.807576	-2.555575	4.033682
4	6	0	-1.429735	-3.282961	3.049300
5	6	0	-1.728504	-2.601741	1.860532
6	6	0	-1.445344	-1.248579	1.679036
7	1	0	-1.681772	-4.328665	3.178767
8	1	0	-2.221037	-3.160063	1.075309
9	6	0	-0.639787	0.972207	2.747011
10	6	0	0.241158	1.722637	1.924304
11	6	0	-1.346777	1.690278	3.687403
12	6	0	0.355940	3.101631	2.088133
13	6	0	-1.231188	3.068742	3.838503
14	6	0	-0.381655	3.807653	3.051634
15	1	0	1.041197	3.663577	1.464893
16	1	0	-0.275898	4.879450	3.169384
17	8	0	-0.398141	-2.961486	5.262089
18	8	0	0.109435	-0.735859	4.977298
19	8	0	-2.255452	1.213807	4.581868
20	8	0	-2.064173	3.484025	4.829896
21	6	0	0.000888	-1.771426	5.951272
22	1	0	0.971212	-1.931860	6.423662
23	1	0	-0.766976	-1.505008	6.686729
24	6	0	-2.459760	2.285033	5.500800
25	1	0	-1.823860	2.135242	6.382544
26	1	0	-3.513951	2.342938	5.769301
27	15	0	1.186301	0.843468	0.630618
28	15	0	-1.825774	-0.436152	0.080925
29	46	0	-0.006835	0.040650	-1.180351
30	6	0	2.081785	-0.454380	1.523204
31	6	0	2.904112	-0.167206	2.603675
32	6	0	1.886202	-1.780429	1.139738
33	6	0	3.539571	-1.177709	3.334337
34	1	0	3.052100	0.870044	2.886094
35	6	0	2.439107	-2.837941	1.854631
36	1	0	1.269735	-1.971583	0.270137
37	6	0	3.209398	-2.508944	2.998110
38	6	0	2.324829	2.068823	-0.076631
39	6	0	3.706330	2.028448	0.041874
40	6	0	1.737331	3.011585	-0.916444
41	6	0	4.521334	2.948224	-0.630398
42	1	0	4.155628	1.257364	0.654782
43	6	0	2.481338	3.945778	-1.630050
44	1	0	0.659971	2.991566	-1.036030
45	6	0	3.884569	3.924715	-1.434448
46	6	0	-2.870207	-1.596909	-0.836941
47	6	0	-4.167427	-1.277228	-1.200930
48	6	0	-2.308618	-2.783443	-1.295952
49	6	0	-4.951463	-2.144029	-1.967548
50	1	0	-4.585595	-0.328291	-0.890209
51	6	0	-3.042340	-3.717322	-2.020862
52	1	0	-1.267265	-2.984735	-1.073569
53	6	0	-4.394960	-3.399272	-2.293643
54	6	0	-2.895677	0.962299	0.509660
55	6	0	-2.758317	2.198324	-0.102355
56	6	0	-3.921826	0.761620	1.436892
57	6	0	-3.689861	3.227016	0.098773
58	1	0	-1.907511	2.364835	-0.755509
59	6	0	-4.896500	1.728038	1.652542
60	1	0	-3.960926	-0.177450	1.975290
61	6	0	-4.820944	2.908467	0.871800
62	6	0	-3.358945	4.610265	-0.507666
63	6	0	-3.366142	4.526503	-2.043900
64	1	0	-3.106129	5.499919	-2.474524
65	1	0	-2.640799	3.796633	-2.412043
66	1	0	-4.352676	4.240506	-2.423347
67	6	0	-4.273362	5.769398	-0.073609

68	1	0	-4.379937	5.821264	1.012536
69	1	0	-3.815084	6.704762	-0.413464
70	1	0	-5.269168	5.719454	-0.515179
71	6	0	-1.943444	4.999674	-0.025618
72	1	0	-1.936011	5.151536	1.058460
73	1	0	-1.187704	4.246108	-0.260001
74	1	0	-1.639466	5.936365	-0.504356
75	6	0	-5.962353	1.539670	2.749948
76	6	0	-5.916699	2.753393	3.699370
77	1	0	-6.209003	3.671619	3.187399
78	1	0	-6.599105	2.595499	4.541791
79	1	0	-4.907343	2.893685	4.098852
80	6	0	-7.380313	1.390123	2.169101
81	1	0	-7.414391	0.608804	1.402280
82	1	0	-8.075106	1.104692	2.966862
83	1	0	-7.744289	2.321851	1.734696
84	6	0	-5.670000	0.284891	3.589258
85	1	0	-5.764197	-0.636750	3.004824
86	1	0	-4.669131	0.316415	4.032158
87	1	0	-6.396985	0.226960	4.405493
88	8	0	-5.894966	3.759897	0.903530
89	8	0	-5.188394	-4.338805	-2.897548
90	8	0	3.665547	-3.547031	3.768109
91	8	0	4.678719	4.825803	-2.093615
92	6	0	4.802266	6.115134	-1.500103
93	1	0	5.835490	6.445068	-1.630861
94	1	0	4.146088	6.837885	-1.994855
95	1	0	4.569467	6.088102	-0.431200
96	6	0	1.709016	4.901394	-2.571758
97	6	0	2.488403	5.275464	-3.846335
98	1	0	1.832034	5.855129	-4.504015
99	1	0	3.377460	5.871873	-3.654191
100	1	0	2.799663	4.377731	-4.389905
101	6	0	1.312408	6.167610	-1.786927
102	1	0	0.696146	6.822192	-2.413934
103	1	0	0.728227	5.900467	-0.900159
104	1	0	2.179881	6.740315	-1.452734
105	6	0	-6.723408	3.637415	-0.248662
106	1	0	-7.550827	4.336594	-0.115753
107	1	0	-6.181266	3.888357	-1.166638
108	1	0	-7.118551	2.619563	-0.347113
109	6	0	-5.879948	-5.159162	-1.968018
110	1	0	-6.504074	-5.840724	-2.549378
111	1	0	-5.184923	-5.743535	-1.351871
112	1	0	-6.516936	-4.561459	-1.303987
113	6	0	-6.350564	-1.614082	-2.363827
114	6	0	-6.176833	-0.214265	-2.998215
115	1	0	-5.740797	0.516697	-2.311673
116	1	0	-5.540598	-0.266378	-3.888552
117	1	0	-7.154280	0.174079	-3.304143
118	6	0	-7.124545	-2.454295	-3.393594
119	1	0	-7.466751	-3.408926	-2.992224
120	1	0	-8.014664	-1.889777	-3.693721
121	1	0	-6.531808	-2.651665	-4.289953
122	6	0	-7.211524	-1.484082	-1.092827
123	1	0	-7.368815	-2.460005	-0.621038
124	1	0	-6.742296	-0.824381	-0.354931
125	1	0	-8.193053	-1.064626	-1.340869
126	6	0	-2.295547	-5.012180	-2.424786
127	6	0	-2.062051	-5.854160	-1.155141
128	1	0	-1.479523	-5.302665	-0.409138
129	1	0	-3.011473	-6.145675	-0.693597
130	1	0	-1.510746	-6.768831	-1.402349
131	6	0	-2.998509	-5.889768	-3.474682
132	1	0	-2.302838	-6.678626	-3.782117
133	1	0	-3.894626	-6.378031	-3.090987
134	1	0	-3.275259	-5.318295	-4.364363
135	6	0	-0.922210	-4.638944	-3.029780
136	1	0	-0.393500	-5.551795	-3.324057
137	1	0	-1.044154	-4.013918	-3.920531
138	1	0	-0.275695	-4.105394	-2.328275
139	6	0	6.054257	2.733464	-0.577815
140	6	0	0.399572	4.239018	-3.059780
141	1	0	0.581898	3.259091	-3.513510
142	1	0	-0.337204	4.117599	-2.262975
143	1	0	-0.063763	4.880943	-3.815354
144	6	0	6.415567	1.833879	-1.777276
145	1	0	7.493495	1.635578	-1.794059
146	1	0	5.895261	0.871226	-1.713986
147	1	0	6.142771	2.312643	-2.723451
148	6	0	6.908712	4.012870	-0.642360
149	1	0	6.592914	4.747302	0.105179

150	1	0	7.949981	3.751107	-0.426406
151	1	0	6.880897	4.478422	-1.625647
152	6	0	6.470171	2.016623	0.719929
153	1	0	7.559704	1.917947	0.744167
154	1	0	6.164011	2.583834	1.605752
155	6	0	2.909318	-3.757477	4.955961
156	1	0	3.307890	-4.659146	5.424770
157	1	0	3.010568	-2.921891	5.655453
158	1	0	1.848048	-3.902840	4.733465
159	6	0	4.545341	-0.721787	4.419247
160	6	0	3.776988	-0.125673	5.613835
161	1	0	4.481701	0.228770	6.374320
162	1	0	3.159950	0.724008	5.303551
163	1	0	3.117279	-0.863098	6.081804
164	6	0	5.513793	-1.811338	4.914743
165	1	0	6.288224	-1.329671	5.521544
166	1	0	5.040704	-2.568421	5.538480
167	1	0	6.008144	-2.315772	4.079647
168	6	0	5.449259	0.381100	3.820751
169	1	0	6.013057	-0.000559	2.963235
170	1	0	4.896134	1.266422	3.499331
171	1	0	6.170494	0.709564	4.575928
172	6	0	2.274939	-4.287351	1.343832
173	6	0	1.417995	-4.323847	0.066150
174	1	0	1.352322	-5.354469	-0.295981
175	1	0	0.397012	-3.970025	0.250018
176	1	0	1.855801	-3.721646	-0.736709
177	6	0	1.602024	-5.217218	2.370584
178	1	0	0.697232	-4.768466	2.788230
179	1	0	1.314611	-6.151490	1.875899
180	1	0	2.276749	-5.473868	3.187126
181	6	0	3.658826	-4.866927	0.994148
182	1	0	4.108678	-4.334787	0.152997
183	1	0	4.340640	-4.819338	1.845958
184	1	0	3.552574	-5.917878	0.701863
185	8	0	-0.972892	-0.677043	-2.790658
186	6	0	-1.168540	0.154103	-3.894210
187	6	0	-2.213739	1.237201	-3.625114
188	1	0	-1.862981	1.895792	-2.825601
189	1	0	-2.400970	1.853204	-4.512663
190	1	0	-3.159801	0.785251	-3.303306
191	6	0	-1.581401	-0.735308	-5.066834
192	1	0	-2.518794	-1.253697	-4.830982
193	1	0	-1.727499	-0.151233	-5.982757
194	1	0	-0.813266	-1.491254	-5.259932
195	1	0	-0.233394	0.672740	-4.179871
196	8	0	2.111085	-1.656301	-2.828240
197	8	0	1.638555	0.432984	-2.555309
198	6	0	2.226852	-0.401976	-3.240856
199	6	0	3.009051	0.048262	-4.479842
200	6	0	2.208048	-0.341546	-5.740713
201	1	0	2.069472	-1.418761	-5.856141
202	1	0	1.223217	0.133193	-5.740927
203	1	0	2.757275	0.015833	-6.616880
204	6	0	4.438026	-0.521604	-4.533811
205	1	0	4.988905	0.027144	-5.302781
206	1	0	4.961932	-0.380464	-3.584224
207	1	0	4.481988	-1.575268	-4.813254
208	6	0	3.111878	1.579610	-4.424332
209	1	0	3.640878	1.928056	-5.315986
210	1	0	2.125040	2.044349	-4.402261
211	1	0	3.666538	1.910256	-3.541763
212	6	0	2.639485	-2.829978	-3.440204
213	1	0	1.916747	-3.613695	-3.197731
214	1	0	2.685564	-2.752408	-4.524748
215	6	0	3.993484	-3.302287	-2.924368
216	8	0	4.491961	-4.260315	-3.489657
217	6	0	4.628499	-2.670312	-1.770900
218	6	0	4.308429	-1.649484	-0.911908
219	8	0	5.800978	-3.244784	-1.372757
220	6	0	5.329010	-1.614991	0.072862
221	1	0	3.444255	-1.008538	-0.953579
222	6	0	6.203470	-2.603917	-0.263959
223	1	0	5.382646	-0.964174	0.932147
224	1	0	7.121665	-2.955671	0.183056
225	1	0	6.065631	1.007749	0.802829

TS4a(DTBM-Segphos)

RwB97XD SCF energy	-5213.493751 a.u.
RwB97XD SCF enthalpy	-5211.435854 a.u.
RwB97XD SCF free energy	-5211.691792 a.u.

Three lowest frequencies (cm-1) -1004, 11.7, 11.9

Imaginary frequency (cm-1) -1004

Cartesian coordinates:

Standard orientation:

Center Number	Atomic Number	Atomic Type	Coordinates (Angstroms) X	Y	Z
1	6	0	1.252674	-0.899454	2.716742
2	6	0	1.127758	-0.845612	4.088420
3	6	0	1.462720	0.280148	4.836787
4	6	0	1.910201	1.434982	4.243459
5	6	0	2.024047	1.417032	2.845922
6	6	0	1.716217	0.288362	2.087018
7	1	0	2.178767	2.311405	4.821070
8	1	0	2.392847	2.311089	2.359907
9	6	0	1.020680	-2.196179	2.029534
10	6	0	0.040415	-2.467181	1.035810
11	6	0	1.825742	-3.256065	2.388609
12	6	0	-0.059043	-3.741167	0.478071
13	6	0	1.723793	-4.520692	1.816758
14	6	0	0.786421	-4.796925	0.851653
15	1	0	-0.820824	-3.946907	-0.263193
16	1	0	0.692640	-5.779302	0.404423
17	8	0	1.280226	0.020584	6.153965
18	8	0	0.699506	-1.830033	4.920081
19	8	0	2.840248	-3.252300	3.294834
20	8	0	2.668203	-5.336169	2.354653
21	6	0	1.006146	-1.381431	6.238829
22	1	0	0.148909	-1.545082	6.893072
23	1	0	1.897922	-1.903306	6.605612
24	6	0	3.163083	-4.627450	3.493983
25	1	0	2.658572	-4.993382	4.396920
26	1	0	4.244569	-4.743433	3.559193
27	15	0	-1.032336	-1.111699	0.434753
28	15	0	1.907849	0.338738	0.275294
29	46	0	0.035777	0.300715	-1.011435
30	6	0	-1.719105	-0.338487	1.915939
31	6	0	-2.290723	-1.122601	2.909310
32	6	0	-1.677716	1.046468	2.061309
33	6	0	-2.787504	-0.563505	4.089666
34	1	0	-2.355922	-2.195800	2.758587
35	6	0	-2.090309	1.659984	3.241954
36	1	0	-1.288629	1.638515	1.241704
37	6	0	-2.558101	0.815798	4.278898
38	6	0	-2.387973	-1.872663	-0.500080
39	6	0	-3.718851	-1.722476	-0.119991
40	6	0	-2.089099	-2.451428	-1.728062
41	6	0	-4.754038	-2.223174	-0.909373
42	1	0	-3.945201	-1.198994	0.799163
43	6	0	-3.073596	-2.964730	-2.571120
44	1	0	-1.055112	-2.474548	-2.053885
45	6	0	-4.399603	-2.915292	-2.092946
46	6	0	2.777004	1.880722	-0.110377
47	6	0	4.038936	1.853388	-0.690299
48	6	0	2.108559	3.090919	0.023838
49	6	0	4.691208	3.027133	-1.058439
50	1	0	4.512946	0.898206	-0.864975
51	6	0	2.699674	4.306781	-0.325805
52	1	0	1.091642	3.085857	0.401621
53	6	0	4.037737	4.251415	-0.777694
54	6	0	3.003532	-1.017548	-0.199196
55	6	0	2.750282	-1.689899	-1.384734
56	6	0	4.120004	-1.358895	0.566106
57	6	0	3.630332	-2.648919	-1.894845

58	1	0	1.842944	-1.456899	-1.932768
59	6	0	5.053362	-2.276759	0.095350
60	1	0	4.257762	-0.883933	1.529811
61	6	0	4.838664	-2.822256	-1.197097
62	6	0	3.155110	-3.426014	-3.143368
63	6	0	3.057142	-2.466293	-4.343287
64	1	0	2.696711	-3.004834	-5.226851
65	1	0	2.359582	-1.646218	-4.143472
66	1	0	4.031579	-2.029286	-4.585356
67	6	0	4.012750	-4.640656	-3.536725
68	1	0	4.177637	-5.310775	-2.689224
69	1	0	3.475418	-5.199819	-4.310601
70	1	0	4.983106	-4.367004	-3.951671
71	6	0	1.746785	-3.986884	-2.837527
72	1	0	1.783495	-4.696583	-2.004141
73	1	0	1.026967	-3.205607	-2.580053
74	1	0	1.362168	-4.511542	-3.718362
75	6	0	6.223269	-2.735445	0.988515
76	6	0	6.167142	-4.270712	1.120351
77	1	0	6.351131	-4.758987	0.161589
78	1	0	6.925157	-4.613864	1.833234
79	1	0	5.185202	-4.592825	1.481973
80	6	0	7.593232	-2.312723	0.426805
81	1	0	7.620211	-1.237853	0.216398
82	1	0	8.372573	-2.527926	1.166422
83	1	0	7.846231	-2.854524	-0.484950
84	6	0	6.104332	-2.142382	2.402210
85	1	0	6.217992	-1.052784	2.402553
86	1	0	5.147054	-2.391497	2.870767
87	1	0	6.902779	-2.554080	3.027463
88	8	0	5.857263	-3.548083	-1.758362
89	8	0	4.745812	5.407602	-0.989268
90	8	0	-2.806137	1.385695	5.500773
91	8	0	-5.410383	-3.512867	-2.803193
92	6	0	-5.499866	-4.917603	-2.587561
93	1	0	-6.391506	-5.260077	-3.116257
94	1	0	-4.625572	-5.445604	-2.980362
95	1	0	-5.596953	-5.150838	-1.521894
96	6	0	-2.601966	-3.481356	-3.952439
97	6	0	-3.710268	-3.612465	-5.012106
98	1	0	-3.238165	-3.817945	-5.978975
99	1	0	-4.409241	-4.424173	-4.814814
100	1	0	-4.280692	-2.684192	-5.108063
101	6	0	-1.890723	-4.835800	-3.776774
102	1	0	-1.479377	-5.173057	-4.735110
103	1	0	-1.063316	-4.755567	-3.063661
104	1	0	-2.573574	-5.609750	-3.413089
105	6	0	6.584247	-2.802089	-2.731680
106	1	0	7.398055	-3.443590	-3.074498
107	1	0	5.954133	-2.528598	-3.584637
108	1	0	6.999256	-1.885054	-2.297578
109	6	0	5.282494	5.967397	0.205487
110	1	0	5.922847	6.797911	-0.097850
111	1	0	4.495905	6.344830	0.866384
112	1	0	5.877340	5.231401	0.756986
113	6	0	6.028163	2.949462	-1.826840
114	6	0	6.434230	1.488195	-2.096094
115	1	0	6.631433	0.937267	-1.169436
116	1	0	5.674462	0.943758	-2.667219
117	1	0	7.356464	1.480584	-2.685368
118	6	0	5.851003	3.638627	-3.194176
119	1	0	5.586382	4.691443	-3.076420
120	1	0	6.784339	3.580347	-3.765657
121	1	0	5.065583	3.145450	-3.777601
122	6	0	7.193008	3.608275	-1.064774

123	1	0	7.098476	4.693750	-1.033782
124	1	0	7.260559	3.230679	-0.038763
125	1	0	8.136959	3.374481	-1.569569
126	6	0	1.814390	5.572753	-0.207840
127	6	0	1.630140	5.929714	1.279196
128	1	0	1.188559	5.095838	1.834594
129	1	0	2.582091	6.183454	1.755649
130	1	0	0.961477	6.792104	1.379421
131	6	0	2.333060	6.803841	-0.972550
132	1	0	1.547653	7.567419	-0.964704
133	1	0	3.221956	7.249927	-0.528016
134	1	0	2.556398	6.562121	-2.015588
135	6	0	0.422173	5.270661	-0.810650
136	1	0	-0.202292	6.167840	-0.748480
137	1	0	0.504281	4.990941	-1.866269
138	1	0	-0.114470	4.476744	-0.287458
139	6	0	-6.222896	-1.907941	-0.549184
140	6	0	-1.587092	-2.471606	-4.538648
141	1	0	-2.023841	-1.470036	-4.584968
142	1	0	-0.659370	-2.415494	-3.963885
143	1	0	-1.312450	-2.778761	-5.553111
144	6	0	-6.807143	-1.017444	-1.664211
145	1	0	-7.848145	-0.763039	-1.434012
146	1	0	-6.241884	-0.083022	-1.752496
147	1	0	-6.785852	-1.530633	-2.628627
148	6	0	-7.097196	-3.166069	-0.386013
149	1	0	-6.628794	-3.890892	0.288583
150	1	0	-8.060821	-2.881263	0.050531
151	1	0	-7.300735	-3.652254	-1.339435
152	6	0	-6.322123	-1.134286	0.775599
153	1	0	-5.766742	-0.192956	0.758874
154	1	0	-7.371828	-0.890136	0.966572
155	1	0	-5.962731	-1.734200	1.617582
156	6	0	-1.780957	1.117700	6.453274
157	1	0	-2.099198	1.572489	7.393264
158	1	0	-1.639242	0.042293	6.601322
159	1	0	-0.828383	1.554970	6.140163
160	6	0	-3.577008	-1.510275	5.026016
161	6	0	-2.638667	-2.580373	5.610374
162	1	0	-3.214893	-3.289741	6.214958
163	1	0	-2.124907	-3.144100	4.826102
164	1	0	-1.877924	-2.130830	6.254720
165	6	0	-4.336050	-0.829728	6.178839
166	1	0	-4.989820	-1.577369	6.641460
167	1	0	-3.680761	-0.448392	6.962047
168	1	0	-4.963084	-0.008517	5.823161
169	6	0	-4.662013	-2.206690	4.171927
170	1	0	-5.353140	-1.466054	3.756435
171	1	0	-4.249014	-2.790276	3.344943
172	1	0	-5.239544	-2.893051	4.800079
173	6	0	-2.086714	3.198568	3.364025
174	6	0	-1.611454	3.850704	2.055336
175	1	0	-1.662994	4.939558	2.157245
176	1	0	-0.567459	3.594155	1.840802
177	1	0	-2.217160	3.566976	1.188892
178	6	0	-1.149748	3.700841	4.478299
179	1	0	-0.152602	3.258491	4.385608
180	1	0	-1.042700	4.788370	4.397902
181	1	0	-1.540995	3.481617	5.471884
182	6	0	-3.520553	3.684114	3.651754
183	1	0	-4.206760	3.377168	2.856912
184	1	0	-3.888051	3.286668	4.600217
185	1	0	-3.538040	4.778526	3.708458
186	8	0	-1.705068	0.395683	-2.238369
187	6	0	-2.140178	1.437856	-2.757843
188	6	0	-1.449521	2.738793	-2.559617
189	1	0	-1.785721	3.634319	-3.071654
190	1	0	-0.718336	2.808556	-1.762827
191	8	0	-2.823290	2.969651	-1.139229
192	6	0	-3.734784	3.838223	-1.434246
193	8	0	-3.626880	4.694806	-2.318055
194	6	0	-5.026972	3.750729	-0.593773
195	6	0	-5.044061	4.960399	0.354221
196	1	0	-5.031446	5.895787	-0.213672
197	1	0	-5.947473	4.945360	0.974500
198	1	0	-4.176167	4.954255	1.021515
199	6	0	-6.223967	3.825303	-1.552016
200	1	0	-6.234367	2.968143	-2.235519
201	1	0	-7.162335	3.817205	-0.986098
202	1	0	-6.184305	4.737368	-2.152556
203	6	0	-5.087849	2.441819	0.197670
204	1	0	-6.020536	2.392368	0.771764
205	1	0	-5.056601	1.581073	-0.478381
206	1	0	-4.248844	2.343353	0.891533
207	8	0	1.140705	1.403171	-2.400119
208	6	0	0.725628	1.802728	-3.586303
209	1	0	-0.400097	2.390222	-3.450158
210	6	0	1.549310	2.960545	-4.133774
211	1	0	1.090876	3.396259	-5.025720
212	1	0	1.671757	3.730380	-3.368052
213	1	0	2.542284	2.580455	-4.400044
214	6	0	0.393412	0.725829	-4.609185
215	1	0	1.336680	0.305065	-4.976082
216	1	0	-0.186356	-0.078863	-4.153743
217	1	0	-0.157897	1.133192	-5.461704
218	6	0	-3.293515	1.361806	-3.616229
219	6	0	-3.999103	2.294049	-4.337086
220	8	0	-3.870925	0.135315	-3.755180
221	6	0	-5.067747	1.592338	-4.949084
222	1	0	-3.791418	3.353160	-4.380252
223	6	0	-4.934198	0.290898	-4.554751
224	1	0	-5.836189	1.994393	-5.592272
225	1	0	-5.505883	-0.600648	-4.766280

TS5a(DTBM-Segphos)

RwB97XD SCF energy -5213.443785 a.u.

RwB97XD SCF enthalpy -5211.384672 a.u.

RwB97XD SCF free energy -5211.640436 a.u.

Three lowest frequencies (cm^{-1}) -674.9, 8.2, 9.6 a.u

Imaginary frequency (cm^{-1}) -674.9

Cartesian coordinates:

Standard orientation:

Center Number	Atomic Number	Atomic Type	Coordinates (Angstroms) X	Y	Z
1	6	0	1.282538	2.517062	1.315915
2	6	0	2.035490	3.063184	2.333145
3	6	0	1.582768	3.157934	3.645567
4	6	0	0.342449	2.699104	4.014930
5	6	0	-0.438402	2.121569	3.002710
6	6	0	0.003233	2.016823	1.684238
7	1	0	-0.025697	2.787931	5.030192
8	1	0	-1.425135	1.768032	3.272287
9	6	0	1.813916	2.596938	-0.070061
10	6	0	2.168925	1.503532	-0.907301
11	6	0	2.041997	3.854682	-0.586056
12	6	0	2.700304	1.738900	-2.174273
13	6	0	2.569963	4.075753	-1.854416
14	6	0	2.904604	3.031901	-2.681336
15	1	0	2.996649	0.906694	-2.799201
16	1	0	3.326227	3.189317	-3.667037
17	8	0	2.531849	3.753945	4.410801
18	8	0	3.287327	3.582679	2.245867
19	8	0	1.800534	5.053022	0.014572
20	8	0	2.671497	5.414927	-2.071384
21	6	0	3.522873	4.230334	3.493924

22	1	0	4.515406	3.969864	3.862184
23	1	0	3.409070	5.313798	3.371190
24	6	0	2.512811	6.000893	-0.777254
25	1	0	3.501754	6.173999	-0.332970
26	1	0	1.937780	6.922671	-0.855239
27	15	0	1.916192	-0.202743	-0.273420
28	15	0	-1.020947	1.177434	0.425957
29	46	0	-0.264865	-0.801930	-0.403734
30	6	0	2.785475	-0.187720	1.314359
31	6	0	4.113196	0.218470	1.347031
32	6	0	2.159819	-0.600863	2.485472
33	6	0	4.853625	0.233793	2.531319
34	1	0	4.590801	0.516434	0.419570
35	6	0	2.819015	-0.549120	3.711316
36	1	0	1.135996	-0.944647	2.427488
37	6	0	4.147715	-0.056687	3.717861
38	6	0	2.838343	-1.311749	-1.374244
39	6	0	3.814673	-2.178863	-0.892375
40	6	0	2.442789	-1.407267	-2.703165
41	6	0	4.491955	-3.049927	-1.744500
42	1	0	4.048621	-2.173799	0.163720
43	6	0	3.098536	-2.234575	-3.617029
44	1	0	1.594790	-0.819283	-3.039197
45	6	0	4.200148	-2.966401	-3.126840
46	6	0	-2.590521	0.750927	1.232226
47	6	0	-3.806409	1.180026	0.711861
48	6	0	-2.580218	-0.141937	2.297160
49	6	0	-5.015539	0.800097	1.291495
50	1	0	-3.802088	1.810982	-0.166939
51	6	0	-3.750108	-0.531350	2.956569
52	1	0	-1.628652	-0.532869	2.642705
53	6	0	-4.956802	0.026047	2.478936
54	6	0	-1.354153	2.390240	-0.875691
55	6	0	-1.372117	1.961181	-2.196365
56	6	0	-1.520377	3.743781	-0.584981
57	6	0	-1.605896	2.841298	-3.257664
58	1	0	-1.201972	0.910503	-2.399278
59	6	0	-1.801877	4.663414	-1.590615
60	1	0	-1.420092	4.072982	0.442027
61	6	0	-1.939253	4.166020	-2.912078
62	6	0	-1.429056	2.281494	-4.690421
63	6	0	-2.624284	1.388254	-5.063855
64	1	0	-2.467767	0.940187	-6.051648
65	1	0	-2.742263	0.575920	-4.342621
66	1	0	-3.563912	1.947745	-5.092747
67	6	0	-1.229131	3.357900	-5.772369
68	1	0	-0.439856	4.062828	-5.494892
69	1	0	-0.927198	2.862244	-6.701326
70	1	0	-2.133653	3.927525	-5.984836
71	6	0	-0.151127	1.407476	-4.721919
72	1	0	0.729143	1.988089	-4.426828
73	1	0	-0.215859	0.531018	-4.070912
74	1	0	0.009286	1.037071	-5.739642
75	6	0	-1.862768	6.171986	-1.274552
76	6	0	-0.860663	6.913298	-2.182703
77	1	0	-1.163071	6.871455	-3.230009
78	1	0	-0.789480	7.965249	-1.884737
79	1	0	0.134583	6.465752	-2.102924
80	6	0	-3.273792	6.754202	-1.474667
81	1	0	-4.022233	6.177317	-0.920420
82	1	0	-3.301384	7.783740	-1.100451
83	1	0	-3.559628	6.778891	-2.526438
84	6	0	-1.454780	6.449025	0.182855
85	1	0	-2.167440	6.026384	0.899858
86	1	0	-0.457831	6.056981	0.407784
87	1	0	-1.434655	7.531261	0.345351
88	8	0	-2.383638	5.041971	-3.869090
89	8	0	-6.130810	-0.184862	3.161079
90	8	0	4.756543	0.100459	4.936429
91	8	0	5.019392	-3.648486	-3.992185
92	6	0	5.979809	-2.810377	-4.626196
93	1	0	6.614541	-3.461063	-5.230869
94	1	0	5.505375	-2.067982	-5.277015
95	1	0	6.595681	-2.281666	-3.890032
96	6	0	2.524158	-2.275805	-5.053947
97	6	0	3.042069	-3.423533	-5.937948
98	1	0	2.457623	-3.433002	-6.864659
99	1	0	4.090055	-3.318665	-6.215822
100	1	0	2.914121	-4.393773	-5.450075
101	6	0	2.799553	-0.929356	-5.748752
102	1	0	2.337288	-0.915599	-6.742148
103	1	0	2.384607	-0.093750	-5.175043

104	1	0	3.872435	-0.751540	-5.874194
105	6	0	-3.753106	4.860361	-4.213937
106	1	0	-4.022485	5.687689	-4.873445
107	1	0	-3.917921	3.915618	-4.740593
108	1	0	-4.393443	4.878261	-3.324774
109	6	0	-6.301331	0.697784	4.266823
110	1	0	-7.285631	0.487321	4.688816
111	1	0	-5.538322	0.539190	5.034925
112	1	0	-6.256211	1.745413	3.951936
113	6	0	-6.345154	1.175757	0.598188
114	6	0	-6.091983	1.841443	-0.766856
115	1	0	-5.594134	2.812505	-0.670426
116	1	0	-5.494621	1.208377	-1.431320
117	1	0	-7.054989	2.015986	-1.256635
118	6	0	-7.161098	-0.104833	0.337156
119	1	0	-7.444784	-0.588331	1.272796
120	1	0	-8.077101	0.143123	-0.210934
121	1	0	-6.589648	-0.818120	-0.266277
122	6	0	-7.191943	2.157024	1.429119
123	1	0	-7.627434	1.679957	2.307702
124	1	0	-6.600776	3.020224	1.753139
125	1	0	-8.020205	2.530421	0.816842
126	6	0	-3.570433	-1.514791	4.142551
127	6	0	-2.918568	-0.759042	5.318408
128	1	0	-1.966188	-0.304506	5.028101
129	1	0	-3.568931	0.037448	5.693911
130	1	0	-2.723266	-1.450600	6.145820
131	6	0	-4.849470	-2.204538	4.646920
132	1	0	-4.556225	-2.963275	5.380467
133	1	0	-5.543270	-1.528113	5.144279
134	1	0	-5.380898	-2.711144	3.839081
135	6	0	-2.624231	-2.659766	3.709969
136	1	0	-2.490557	-3.354741	4.545430
137	1	0	-3.043585	-3.220371	2.869002
138	1	0	-1.631164	-2.310438	3.423151
139	6	0	5.468963	-4.095131	-1.166498
140	6	0	0.993935	-2.476469	-4.963819
141	1	0	0.753020	-3.405796	-4.437643
142	1	0	0.483767	-1.655271	-4.455832
143	1	0	0.572431	-2.539376	-5.972679
144	6	0	5.072787	-5.498477	-1.666678
145	1	0	5.742329	-6.248955	-1.231796
146	1	0	4.048415	-5.743077	-1.364235
147	1	0	5.137687	-5.571161	-2.753246
148	6	0	6.929661	-3.796723	-1.551014
149	1	0	7.195843	-2.759929	-1.316462
150	1	0	7.600452	-4.450805	-0.982862
151	1	0	7.115522	-3.975322	-2.611165
152	6	0	5.395142	-4.130011	0.370095
153	1	0	6.037681	-4.936237	0.738033
154	1	0	5.749042	-3.199751	0.825037
155	6	0	4.802676	1.452185	5.381453
156	1	0	5.247605	1.437427	6.378255
157	1	0	5.422390	2.073336	4.726788
158	1	0	3.801459	1.889763	5.435213
159	6	0	6.367299	0.529888	2.398240
160	6	0	6.574727	2.011325	2.035382
161	1	0	7.641228	2.215052	1.887225
162	1	0	6.048982	2.273629	1.111921
163	1	0	6.211926	2.674601	2.826790
164	6	0	7.218361	0.180056	3.632715
165	1	0	8.274538	0.256078	3.352029
166	1	0	7.061173	0.851489	4.476097
167	1	0	7.033476	-0.843136	3.970928
168	6	0	6.934641	-0.345355	1.254837
169	1	0	6.820071	-1.407735	1.492950
170	1	0	6.461105	-0.160978	0.287593
171	1	0	8.003826	-0.141697	1.135663
172	6	0	2.142629	-1.113892	4.977843
173	6	0	0.732994	-1.634536	4.656841
174	1	0	0.280084	-2.038363	5.567524
175	1	0	0.079381	-0.834983	4.289596
176	1	0	0.750809	-2.438158	3.913876
177	6	0	1.993898	-0.067816	6.098230
178	1	0	1.550410	0.858041	5.718468
179	1	0	1.329957	-0.461053	6.876093
180	1	0	2.948638	0.166092	6.569162
181	6	0	2.975282	-2.305819	5.489123
182	1	0	3.033052	-3.092267	4.728519
183	1	0	3.990664	-1.998427	5.748850
184	1	0	2.507224	-2.734762	6.382595
185	8	0	0.211820	-2.685270	-1.070921

186	6	0	0.105757	-3.731402	-0.227585
187	6	0	0.439162	-3.456533	1.239164
188	1	0	-0.178508	-2.636795	1.622145
189	1	0	0.258870	-4.343535	1.855880
190	1	0	1.493995	-3.172889	1.333286
191	6	0	0.836296	-4.954492	-0.771079
192	1	0	1.916603	-4.771320	-0.733381
193	1	0	0.620047	-5.854932	-0.186065
194	1	0	0.558663	-5.135310	-1.814295
195	1	0	-1.025646	-4.028420	-0.171579
196	8	0	-2.174379	-1.047096	-1.271789
197	8	0	-2.739265	-3.022781	-0.455449
198	6	0	-2.746853	-2.174310	-1.385346
199	6	0	-3.492765	-2.374339	-2.713451
200	6	0	-4.504004	-1.221927	-2.847949
201	1	0	-5.009883	-1.279199	-3.817703
202	1	0	-5.266283	-1.283229	-2.063375
203	1	0	-4.009558	-0.251479	-2.767232
204	6	0	-4.238894	-3.704711	-2.794185
205	1	0	-4.978938	-3.800118	-1.995475
206	1	0	-4.771256	-3.762907	-3.749513
207	1	0	-3.558455	-4.559768	-2.754012
208	6	0	-2.445659	-2.299234	-3.837199
209	1	0	-1.725664	-3.120479	-3.754495
210	1	0	-2.934851	-2.371772	-4.814444
211	1	0	-1.892269	-1.358098	-3.792150
212	6	0	-2.303489	-5.128869	-0.318455
213	1	0	-1.706295	-5.520807	0.503022
214	1	0	-1.935887	-5.327736	-1.318847
215	6	0	-3.745724	-5.497127	-0.126132
216	8	0	-3.865197	-6.652767	-0.510454
217	6	0	-4.823622	-4.781023	0.525090
218	6	0	-5.047596	-3.522644	1.029052
219	8	0	-5.950933	-5.540406	0.688265
220	6	0	-6.374625	-3.519334	1.526379
221	1	0	-4.348270	-2.702672	1.023795
222	6	0	-6.865880	-4.769912	1.289014
223	1	0	-6.890178	-2.698453	2.001638
224	1	0	-7.821527	-5.232931	1.487400
225	1	0	4.378563	-4.325446	0.728359

10a

RwB97XD SCF energy -5213.548817 a.u

RwB97XD SCF enthalpy -5211.487545 a.u

RwB97XD SCF free energy -5211.743333 a.u

Three lowest frequencies (cm^{-1}) -21.8, -16.0, 10.6

Standard orientation:

Center Number	Atomic Number	Atomic Type	Coordinates (Angstroms) X	Y	Z
1	6	0	-1.227501	2.865454	-0.694013
2	6	0	-1.148686	4.222599	-0.476600
3	6	0	-1.564029	4.827623	0.709042
4	6	0	-2.053427	4.093695	1.762202
5	6	0	-2.121865	2.705200	1.580484
6	6	0	-1.729704	2.090041	0.392378
7	1	0	-2.386200	4.558763	2.682123
8	1	0	-2.520663	2.112024	2.394163
9	6	0	-0.868526	2.334490	-2.034295
10	6	0	0.161962	1.392622	-2.304002
11	6	0	-1.566621	2.789537	-3.131354
12	6	0	0.402115	0.951426	-3.604053
13	6	0	-1.332181	2.328934	-4.424807
14	6	0	-0.354332	1.402224	-4.695387
15	1	0	1.206234	0.253469	-3.800381
16	1	0	-0.162223	1.047808	-5.700971
17	8	0	-1.410171	6.167245	0.610724
18	8	0	-0.686894	5.168661	-1.330544
19	8	0	-2.584158	3.687487	-3.149182
20	8	0	-2.200441	2.923538	-5.279158
21	6	0	-1.043016	6.422988	-0.750015
22	1	0	-0.185254	7.097040	-0.778322
23	1	0	-1.904135	6.842643	-1.281550
24	6	0	-2.812695	3.977295	-4.529613
25	1	0	-2.334867	4.930353	-4.783901
26	1	0	-3.884589	3.996094	-4.727381
27	15	0	1.109370	0.727004	-0.903323
28	15	0	-1.853239	0.285255	0.236830
29	46	0	0.070482	-0.918751	0.215273
30	6	0	1.625354	2.129480	0.101613
31	6	0	2.312655	3.180363	-0.503351
32	6	0	1.379215	2.145588	1.469659
33	6	0	2.710018	4.295812	0.229491
34	1	0	2.542718	3.110750	-1.559526
35	6	0	1.716631	3.250556	2.252197
36	1	0	0.893380	1.286716	1.919370
37	6	0	2.287864	4.354509	1.581388
38	6	0	2.571678	-0.129667	-1.531890
39	6	0	3.854177	0.338865	-1.255429
40	6	0	2.409706	-1.289017	-2.292276
41	6	0	4.981831	-0.280673	-1.786129
42	1	0	3.968875	1.214497	-0.632718
43	6	0	3.494902	-1.956792	-2.863791
44	1	0	1.408932	-1.675160	-2.446804
45	6	0	4.779764	-1.403654	-2.630553
46	6	0	-2.818495	-0.358726	1.625111
47	6	0	-4.030603	-0.985609	1.367111
48	6	0	-2.331389	-0.323864	2.929846
49	6	0	-4.810775	-1.531921	2.385148
50	1	0	-4.393370	-1.046916	0.350433
51	6	0	-3.080713	-0.806747	4.002728
52	1	0	-1.359065	0.120178	3.115886
53	6	0	-4.354173	-1.353931	3.708708
54	6	0	-2.856659	-0.073930	-1.219664
55	6	0	-2.729209	-1.312093	-1.827906
56	6	0	-3.908042	0.772286	-1.579147
57	6	0	-3.675345	-1.794427	-2.736441
58	1	0	-1.881971	-1.932291	-1.565601
59	6	0	-4.892640	0.346744	-2.466615
60	1	0	-3.966595	1.758904	-1.136363
61	6	0	-4.809819	-0.987038	-2.947139
62	6	0	-3.374783	-3.164227	-3.386755
63	6	0	-3.540913	-4.272533	-2.329816
64	1	0	-3.208132	-5.233585	-2.737759
65	1	0	-2.939669	-4.054187	-1.441212
66	1	0	-4.582425	-4.383338	-2.014786
67	6	0	-4.216233	-3.504261	-4.628838
68	1	0	-4.202260	-2.689462	-5.357291
69	1	0	-3.780492	-4.390284	-5.102511
70	1	0	-5.255266	-3.738063	-4.397255
71	6	0	-1.903624	-3.157186	-3.863606
72	1	0	-1.743377	-2.371669	-4.610613
73	1	0	-1.187483	-3.006752	-3.054814
74	1	0	-1.665101	-4.120007	-4.326742
75	6	0	-5.974623	1.328974	-2.961444
76	6	0	-5.820330	1.462837	-4.489475
77	1	0	-6.012528	0.511870	-4.991840
78	1	0	-6.528712	2.204271	-4.875819
79	1	0	-4.807744	1.786738	-4.752262
80	6	0	-7.409454	0.879872	-2.627333
81	1	0	-7.519518	0.653012	-1.561192
82	1	0	-8.104945	1.691638	-2.867002
83	1	0	-7.712971	0.008504	-3.206152
84	6	0	-5.788113	2.720957	-2.337198
85	1	0	-5.944560	2.706124	-1.252536
86	1	0	-4.797274	3.136100	-2.537091
87	1	0	-6.528354	3.404253	-2.764829
88	8	0	-5.877172	-1.470980	-3.652846
89	8	0	-5.166401	-1.721528	4.742818
90	8	0	2.481354	5.518431	2.281245
91	8	0	5.905511	-1.994138	-3.134299
92	6	0	6.229894	-1.834162	-4.512751
93	1	0	7.309837	-1.681802	-4.581124
94	1	0	5.979671	-2.740175	-5.072133
95	1	0	5.718906	-0.972342	-4.951061
96	6	0	3.202956	-3.215433	-3.715706
97	6	0	4.210856	-4.349082	-3.446936
98	1	0	3.989432	-5.191775	-4.111290
99	1	0	5.251285	-4.064575	-3.591964
100	1	0	4.085509	-4.695469	-2.416125
101	6	0	3.143579	-2.827487	-5.206768
102	1	0	2.884843	-3.709185	-5.804116
103	1	0	2.365176	-2.072959	-5.369610
104	1	0	4.079581	-2.426989	-5.593513
105	6	0	-6.733962	-2.304589	-2.874768
106	1	0	-7.585206	-2.554394	-3.510253
107	1	0	-6.230950	-3.226776	-2.570108
108	1	0	-7.088818	-1.785208	-1.977555
109	6	0	-6.038116	-0.681403	5.165473
110	1	0	-6.624619	-1.079732	5.994949
111	1	0	-5.474711	0.195090	5.508901
112	1	0	-6.713646	-0.369488	4.360154

113	6	0	-6.101873	-2.255361	1.927999
114	6	0	-5.734449	-3.246084	0.796314
115	1	0	-5.320241	-2.753531	-0.087494
116	1	0	-5.008059	-3.988440	1.143546
117	1	0	-6.634738	-3.778801	0.471662
118	6	0	-6.818702	-3.084836	3.006891
119	1	0	-7.283264	-2.473841	3.780700
120	1	0	-7.615963	-3.658113	2.521175
121	1	0	-6.140923	-3.794303	3.489206
122	6	0	-7.088233	-1.212403	1.367584
123	1	0	-7.384816	-0.490086	2.135464
124	1	0	-6.650717	-0.653967	0.533023
125	1	0	-7.993898	-1.710651	1.004245
126	6	0	-2.453684	-0.655167	5.410735
127	6	0	-2.449641	0.840439	5.781706
128	1	0	-1.887023	1.435753	5.055138
129	1	0	-3.469255	1.237778	5.828724
130	1	0	-1.985409	0.982981	6.763854
131	6	0	-3.145703	-1.434077	6.543490
132	1	0	-2.514122	-1.365964	7.436235
133	1	0	-4.122286	-1.027015	6.806646
134	1	0	-3.268833	-2.491248	6.296995
135	6	0	-1.000746	-1.174433	5.368492
136	1	0	-0.539355	-1.058425	6.354728
137	1	0	-0.979489	-2.237513	5.109908
138	1	0	-0.370386	-0.631484	4.659961
139	6	0	6.381486	0.288054	-1.460175
140	6	0	1.823949	-3.813720	-3.372500
141	1	0	1.762513	-4.082386	-2.314824
142	1	0	0.999535	-3.148024	-3.641116
143	1	0	1.688078	-4.730892	-3.954887
144	6	0	7.282673	-0.782564	-0.818244
145	1	0	8.276853	-0.357559	-0.638449
146	1	0	6.872864	-1.097326	0.147311
147	1	0	7.393398	-1.661282	-1.454084
148	6	0	7.038452	0.845540	-2.737635
149	1	0	6.357871	1.519660	-3.269164
150	1	0	7.935142	1.415961	-2.470778
151	1	0	7.349362	0.058646	-3.423022
152	6	0	6.302258	1.454793	-0.458341
153	1	0	7.318308	1.793195	-0.233324
154	1	0	5.756268	2.314729	-0.860809
155	6	0	1.385352	6.428065	2.153972
156	1	0	1.684004	7.347352	2.660600
157	1	0	1.171565	6.641926	1.105682
158	1	0	0.479973	6.031646	2.617169
159	6	0	3.694131	5.314263	-0.390447
160	6	0	4.092154	4.902036	-1.818282
161	1	0	4.842692	5.605161	-2.191615
162	1	0	4.534587	3.901524	-1.856496
163	1	0	3.241675	4.931177	-2.508421
164	6	0	3.148950	6.751001	-0.481478
165	1	0	3.823445	7.354218	-1.098647
166	1	0	2.160225	6.773817	-0.952484
167	1	0	3.087635	7.231656	0.494603
168	6	0	4.972338	5.306480	0.472797
169	1	0	4.757860	5.618764	1.497660
170	1	0	5.419290	4.306558	0.500197
171	1	0	5.711649	5.997408	0.052759
172	6	0	1.599442	3.172956	3.791191
173	6	0	0.978687	1.833841	4.219746
174	1	0	0.910503	1.797933	5.311055
175	1	0	-0.035349	1.720401	3.822292
176	1	0	1.578610	0.977168	3.902071
177	6	0	0.735916	4.284334	4.416166
178	1	0	-0.230255	4.377352	3.910339
179	1	0	0.540554	4.038166	5.465244
180	1	0	1.240077	5.250315	4.402304
181	6	0	3.021263	3.254163	4.380878
182	1	0	3.646061	2.430502	4.019547
183	1	0	3.500230	4.199482	4.112842
184	1	0	2.974461	3.189980	5.473814
185	8	0	-0.761284	-2.572199	1.287489
186	6	0	-0.773987	-3.752219	0.898709
187	6	0	-0.276750	-4.156507	-0.431661
188	1	0	-0.182493	-3.292117	-1.096220
189	1	0	0.739388	-4.592369	-0.294407
190	1	0	-0.901639	-4.935906	-0.878921
191	6	0	-1.272285	-4.805986	1.828991
192	1	0	-2.144754	-5.292485	1.378070
193	1	0	-0.502793	-5.576968	1.944833
194	1	0	-1.538500	-4.383656	2.797898
195	1	0	1.648935	-3.723470	2.758905
196	8	0	2.759369	-3.395131	0.921785
197	8	0	2.522925	-5.263445	-0.307967
198	6	0	3.184414	-4.460274	0.402521
199	6	0	4.679329	-4.795237	0.694958
200	6	0	4.857507	-4.871248	2.217362
201	1	0	4.632806	-3.903935	2.670316
202	1	0	4.190135	-5.618890	2.663636
203	1	0	5.888576	-5.141781	2.476101
204	6	0	5.543529	-3.646491	0.157851
205	1	0	6.599656	-3.796163	0.415024
206	1	0	5.475171	-3.564476	-0.932576
207	1	0	5.209800	-2.699399	0.590980
208	6	0	5.108459	-6.119129	0.061680
209	1	0	6.154200	-6.339105	0.311955
210	1	0	4.488413	-6.946717	0.419346
211	1	0	5.023453	-6.088828	-1.028167
212	6	0	1.499437	-3.275760	3.754458
213	1	0	0.792339	-2.452527	3.627026
214	1	0	1.083525	-4.009612	4.447563
215	6	0	2.825670	-2.799717	4.280966
216	8	0	3.422084	-3.366450	5.184422
217	6	0	3.397133	-1.616648	3.625064
218	6	0	2.934929	-0.814119	2.624716
219	8	0	4.648273	-1.218614	3.992688
220	6	0	3.955596	0.140536	2.362259
221	1	0	1.979352	-0.912520	2.135295
222	6	0	4.969544	-0.163218	3.221201
223	1	0	3.920320	0.951071	1.649339
224	1	0	5.941266	0.275576	3.393124
225	1	0	5.840510	1.152337	0.485460

11a

RwB97XD SCF energy		-5213.607895 au
RwB97XD SCF enthalpy		-5211.544648 au
RwB97XD SCF free energy		-5211.803308 au
Three lowest frequencies (cm^{-1})	12.5, 15.2, 18.6	

Standard orientation:

Center Number	Atomic Number	Atomic Type	Coordinates X	(Angstroms) Y	Z
1	6	0	0.357339	-1.348044	2.660210
2	6	0	0.062305	-1.320975	4.006172
3	6	0	0.574097	-0.360896	4.876169
4	6	0	1.386589	0.654143	4.434620
5	6	0	1.685664	0.664871	3.064269
6	6	0	1.199118	-0.302987	2.187675
7	1	0	1.794005	1.398225	5.108623
8	1	0	2.342937	1.443770	2.699068
9	6	0	-0.086764	-2.512738	1.849866
10	6	0	-0.919798	-2.485734	0.697123
11	6	0	0.367030	-3.756898	2.235403
12	6	0	-1.209021	-3.666564	0.012954
13	6	0	0.071293	-4.927120	1.543643
14	6	0	-0.710561	-4.914393	0.414727
15	1	0	-1.857932	-3.644679	-0.853132
16	1	0	-0.946113	-5.819429	-0.132607
17	8	0	0.152322	-0.622927	6.137947
18	8	0	-0.716095	-2.190123	4.702188
19	8	0	1.185854	-4.048527	3.281893
20	8	0	0.684500	-5.977528	2.147332
21	6	0	-0.446650	-1.920486	6.075546
22	1	0	-1.377508	-1.920557	6.642559
23	1	0	0.262076	-2.663350	6.461686
24	6	0	1.149253	-5.469352	3.400941
25	1	0	0.443120	-5.751358	4.191145
26	1	0	2.152468	-5.846368	3.599880
27	15	0	-1.573371	-0.887119	0.085617
28	15	0	1.618288	-0.221641	0.415164
29	46	0	-0.061218	0.388913	-1.020433
30	6	0	-2.235687	-0.052923	1.546403
31	6	0	-3.103958	-0.707360	2.411126
32	6	0	-1.838819	1.254081	1.818406
33	6	0	-3.584403	-0.091897	3.571386
34	1	0	-3.420710	-1.718372	2.174839
35	6	0	-2.209180	1.892346	2.997849
36	1	0	-1.217566	1.761310	1.091808
37	6	0	-3.033002	1.167207	3.894026
38	6	0	-2.929132	-1.304438	-1.043673

39	6	0	-4.262988	-1.040456	-0.745430
40	6	0	-2.602868	-1.866660	-2.273458
41	6	0	-5.285874	-1.445185	-1.604340
42	1	0	-4.504009	-0.511344	0.167396
43	6	0	-3.576486	-2.317438	-3.166034
44	1	0	-1.555167	-1.968660	-2.537467
45	6	0	-4.918540	-2.196496	-2.745513
46	6	0	2.958351	0.990246	0.245871
47	6	0	4.215475	0.591800	-0.183716
48	6	0	2.706826	2.342845	0.454376
49	6	0	5.260632	1.501408	-0.351800
50	1	0	4.395687	-0.455301	-0.387822
51	6	0	3.713616	3.301053	0.351205
52	1	0	1.700381	2.651487	0.716643
53	6	0	5.009292	2.842029	0.008663
54	6	0	2.385715	-1.796926	-0.027196
55	6	0	2.249087	-2.250547	-1.328325
56	6	0	3.218256	-2.478661	0.860593
57	6	0	2.978828	-3.340255	-1.815091
58	1	0	1.562991	-1.729905	-1.988097
59	6	0	3.999405	-3.547535	0.432787
60	1	0	3.266532	-2.145704	1.890133
61	6	0	3.934123	-3.895048	-0.942263
62	6	0	2.639219	-3.814720	-3.246227
63	6	0	3.171361	-2.796653	-4.271516
64	1	0	2.866693	-3.085863	-5.283624
65	1	0	2.777348	-1.794697	-4.075528
66	1	0	4.263645	-2.735754	-4.254994
67	6	0	3.152005	-5.220859	-3.603267
68	1	0	2.864198	-5.956343	-2.846954
69	1	0	2.698258	-5.520341	-4.554065
70	1	0	4.233321	-5.267775	-3.730958
71	6	0	1.098841	-3.881150	-3.377122
72	1	0	0.673760	-4.561609	-2.631097
73	1	0	0.616186	-2.906221	-3.262220
74	1	0	0.832606	-4.256319	-4.370404
75	6	0	4.838066	-4.356098	1.443672
76	6	0	4.363140	-5.822250	1.406665
77	1	0	4.564023	-6.281106	0.436331
78	1	0	4.882147	-6.404549	2.176318
79	1	0	3.286962	-5.888780	1.595571
80	6	0	6.349258	-4.296770	1.150973
81	1	0	6.694597	-3.262596	1.041056
82	1	0	6.896432	-4.742368	1.989039
83	1	0	6.616813	-4.853160	0.252804
84	6	0	4.638663	-3.831661	2.875529
85	1	0	5.021819	-2.812072	2.995199
86	1	0	3.588007	-3.844393	3.179398
87	1	0	5.196045	-4.471824	3.566394
88	8	0	4.832274	-4.822237	-1.403073
89	8	0	6.051775	3.728306	0.025883
90	8	0	-3.316332	1.742730	5.104983
91	8	0	-5.923806	-2.793916	-3.462096
92	6	0	-6.064714	-4.182781	-3.183099
93	1	0	-6.959535	-4.518203	-3.710550
94	1	0	-5.203208	-4.757808	-3.536817
95	1	0	-6.185896	-4.362875	-2.109327
96	6	0	-3.080977	-2.881734	-4.518992
97	6	0	-4.161874	-2.994268	-5.608650
98	1	0	-3.670445	-3.240399	-6.556046
99	1	0	-4.896146	-3.774530	-5.410799
100	1	0	-4.695230	-2.048722	-5.742265
101	6	0	-2.435711	-4.261137	-4.291499
102	1	0	-2.002430	-4.635426	-5.225829
103	1	0	-1.636810	-4.205004	-3.545401
104	1	0	-3.166670	-4.997249	-3.943194
105	6	0	5.923784	-4.242255	-2.111940
106	1	0	6.598031	-5.059999	-2.372819
107	1	0	5.593589	-3.744020	-3.027984
108	1	0	6.459511	-3.514672	-1.492394
109	6	0	6.715524	3.772301	1.281228
110	1	0	7.506524	4.520005	1.198001
111	1	0	6.028468	4.060916	2.085774
112	1	0	7.160300	2.801430	1.533115
113	6	0	6.581682	0.912695	-0.904164
114	6	0	6.255359	0.108312	-2.184874
115	1	0	5.575990	-0.729599	-2.003189
116	1	0	5.803028	0.754214	-2.945193
117	1	0	7.177941	-0.308739	-2.602380
118	6	0	7.660166	1.933998	-1.302532
119	1	0	8.088803	2.455564	-0.446152
120	1	0	8.474920	1.393192	-1.797207
121	1	0	7.278339	2.680868	-2.003064
122	6	0	7.177901	-0.044663	0.145973
123	1	0	7.434308	0.489774	1.066780
124	1	0	6.478057	-0.846852	0.403596
125	1	0	8.091517	-0.508428	-0.242723
126	6	0	3.306636	4.762549	0.653688
127	6	0	3.027480	4.891767	2.163704
128	1	0	2.243433	4.199606	2.488819
129	1	0	3.927605	4.684685	2.752281
130	1	0	2.695990	5.909528	2.399190
131	6	0	4.325465	5.840783	0.246514
132	1	0	3.849355	6.820317	0.366337
133	1	0	5.223755	5.836940	0.864300
134	1	0	4.628546	5.739411	-0.798719
135	6	0	2.012470	5.091703	-0.121802
136	1	0	1.725533	6.129511	0.074223
137	1	0	2.170419	4.981271	-1.199774
138	1	0	1.163229	4.465833	0.161575
139	6	0	-6.748094	-1.034201	-1.326024
140	6	0	-2.003711	-1.929247	-5.088288
141	1	0	-2.397808	-0.913888	-5.202842
142	1	0	-1.104850	-1.882346	-4.468975
143	1	0	-1.689924	-2.284892	-6.075276
144	6	0	-7.365678	-0.384678	-2.580745
145	1	0	-8.398650	-0.088439	-2.366335
146	1	0	-6.811550	0.515443	-2.865592
147	1	0	-7.375889	-1.070042	-3.428604
148	6	0	-7.605221	-2.236744	-0.889463
149	1	0	-7.133414	-2.777760	-0.061895
150	1	0	-8.583066	-1.883535	-0.542912
151	1	0	-7.780540	-2.935769	-1.708424
152	6	0	-6.819685	0.013355	-0.203265
153	1	0	-7.856061	0.347782	-0.092914
154	1	0	-6.505446	-0.393177	0.761135
155	6	0	-2.553392	1.197491	6.177084
156	1	0	-2.901509	1.684204	7.089967
157	1	0	-2.696112	0.116056	6.266669
158	1	0	-1.486470	1.395256	6.043190
159	6	0	-4.698654	-0.842429	4.343964
160	6	0	-4.128155	-2.118170	4.987056
161	1	0	-4.936651	-2.690973	5.455041
162	1	0	-3.636059	-2.761402	4.251704
163	1	0	-3.398206	-1.874396	5.763475
164	6	0	-5.432081	-0.025281	5.423253
165	1	0	-6.302623	-0.603681	5.751937
166	1	0	-4.826657	0.172435	6.307653
167	1	0	-5.793277	0.929305	5.032203
168	6	0	-5.792017	-1.243028	3.327251
169	1	0	-6.240387	-0.349346	2.882040
170	1	0	-5.420589	-1.880498	2.520756
171	1	0	-6.585808	-1.796080	3.840358
172	6	0	-1.800226	3.362994	3.232074
173	6	0	-0.842983	3.851788	2.131089
174	1	0	-0.589083	4.900191	2.315666
175	1	0	0.091392	3.278046	2.136355
176	1	0	-1.279591	3.788103	1.130918
177	6	0	-1.080087	3.594063	4.574190
178	1	0	-0.264386	2.877434	4.714756
179	1	0	-0.643741	4.598894	4.579139
180	1	0	-1.761201	3.525295	5.422131
181	6	0	-3.074806	4.227529	3.182871
182	1	0	-3.566961	4.140913	2.207799
183	1	0	-3.785477	3.923409	3.956264
184	1	0	-2.822982	5.281513	3.347663
185	8	0	1.327917	1.557284	-2.177840
186	6	0	1.580590	1.406209	-3.377610
187	6	0	0.933162	0.345941	-4.206902
188	1	0	0.674868	-0.527099	-3.602937
189	1	0	-0.000814	0.766580	-4.596559
190	1	0	1.562856	0.062134	-5.052129
191	6	0	2.553976	2.327065	-4.032634
192	1	0	3.408548	1.736941	-4.382602
193	1	0	2.095626	2.775364	-4.920411
194	1	0	2.893551	3.098144	-3.340773
195	1	0	-0.184163	4.340320	-2.553051
196	8	0	-1.790034	2.851348	-1.194395
197	8	0	-1.503663	0.981985	-2.381430
198	6	0	-2.040849	2.135101	-2.173574
199	6	0	-3.060915	2.552808	-3.248505
200	6	0	-2.408496	2.439962	-4.634565
201	1	0	-1.494154	3.040321	-4.698620
202	1	0	-2.154117	1.400493	-4.858908

203	1	0	-3.101509	2.795976	-5.404857
204	6	0	-3.550295	3.981624	-3.007962
205	1	0	-4.305033	4.244007	-3.757513
206	1	0	-4.002639	4.084589	-2.017527
207	1	0	-2.736354	4.707674	-3.081435
208	6	0	-4.250667	1.583191	-3.166451
209	1	0	-4.997535	1.846116	-3.923922
210	1	0	-3.932900	0.551278	-3.337245
211	1	0	-4.732242	1.636416	-2.184076
212	6	0	0.112405	5.105370	-3.274113
213	1	0	1.056295	4.835060	-3.750685
214	1	0	-0.657734	5.154250	-4.052566
215	6	0	0.248907	6.477757	-2.659722
216	8	0	1.012229	7.315864	-3.114178
217	6	0	-0.602188	6.772421	-1.501941
218	6	0	-1.470611	6.010396	-0.767439
219	8	0	-0.527269	8.028248	-0.975922
220	6	0	-1.958916	6.854641	0.270366
221	1	0	-1.713809	4.967392	-0.939538
222	6	0	-1.348458	8.059061	0.088874
223	1	0	-2.663985	6.598884	1.048065
224	1	0	-1.404556	8.998622	0.619157
225	1	0	-6.207579	0.894614	-0.423946

2.15. Catalytic cycle for destructive hydrogenation with 2b
9b(Segphos)

RwB97XD SCF energy -3497.815855 a.u.
RwB97XD SCF enthalpy -3496.845004 a.u.
RwB97XD SCF free energy -3497.000074 a.u.
Three lowest frequencies (cm^{-1}) 18.9, 25.3, 27.0
Cartesian coordinates

Standard orientation:

Center Number	Atomic Number	Atomic Type	Coordinates (Angstroms) X	Y	Z
1	6	0	2.256222	0.332914	1.544066
2	6	0	2.427552	0.209905	2.905242
3	6	0	1.797756	1.042281	3.826737
4	6	0	0.981503	2.072198	3.426914
5	6	0	0.804338	2.233387	2.044726
6	6	0	1.409282	1.392038	1.112597
7	1	0	0.496989	2.730761	4.137982
8	1	0	0.168179	3.043403	1.711111
9	6	0	2.949997	-0.622323	0.640079
10	6	0	2.296141	-1.653802	-0.087753
11	6	0	4.316772	-0.546868	0.491754
12	6	0	3.026357	-2.520129	-0.897290
13	6	0	5.034130	-1.403012	-0.339410
14	6	0	4.418129	-2.409893	-1.042422
15	1	0	2.519729	-3.309068	-1.440512
16	1	0	4.975935	-3.085802	-1.679543
17	8	0	2.137941	0.671931	5.083280
18	8	0	3.182504	-0.707363	3.559150
19	8	0	5.161553	0.338049	1.076809
20	8	0	6.348797	-1.079577	-0.301662
21	6	0	2.977094	-0.480008	4.954553
22	1	0	2.477512	-1.346265	5.398151
23	1	0	3.938809	-0.286487	5.436667
24	6	0	6.459618	0.069706	0.543093
25	1	0	7.151859	-0.146586	1.360647
26	1	0	6.796834	0.926320	-0.048295
27	15	0	0.470214	-1.718963	-0.059453
28	15	0	0.995333	1.553700	-0.661469
29	46	0	-0.504305	-0.016938	-1.303761
30	6	0	0.019630	-1.839068	1.696721
31	6	0	0.747732	-2.662381	2.562676
32	6	0	-1.021835	-1.051091	2.194900
33	6	0	0.433227	-2.695656	3.916739
34	1	0	1.569744	-3.262537	2.184049
35	6	0	-1.317623	-1.071304	3.555253
36	1	0	-1.586460	-0.405424	1.526973
37	6	0	-0.589776	-1.890561	4.415149
38	6	0	0.024083	-3.293109	-0.860993
39	6	0	-0.346038	-4.428562	-0.137612
40	6	0	0.043836	-3.341104	-2.261477
41	6	0	-0.683823	-5.601624	-0.809274
42	1	0	-0.381211	-4.406157	0.946413
43	6	0	-0.285621	-4.515850	-2.926810
44	1	0	0.316844	-2.457558	-2.833583
45	6	0	-0.651106	-5.648327	-2.200024
46	6	0	0.242213	3.199756	-0.841563
47	6	0	0.930745	4.286346	-1.381164
48	6	0	-1.084337	3.359781	-0.423149
49	6	0	0.299963	5.525695	-1.483589
50	1	0	1.951511	4.177189	-1.731389
51	6	0	-1.705932	4.597368	-0.514538
52	1	0	-1.629648	2.507554	-0.031853
53	6	0	-1.012203	5.683820	-1.047674
54	6	0	2.577726	1.536638	-1.549112
55	6	0	2.729874	0.709832	-2.664770
56	6	0	3.657366	2.301306	-1.092198
57	6	0	3.958013	0.640234	-3.315591
58	1	0	1.894489	0.107277	-3.007935
59	6	0	4.877502	2.240179	-1.756277
60	1	0	3.552049	2.926998	-0.211063
61	6	0	5.030930	1.403567	-2.861226
62	8	0	-2.282125	-1.289130	-1.508574
63	6	0	-3.393698	-0.937885	-1.082108
64	6	0	-3.775152	0.524530	-0.940517
65	1	0	-4.786349	0.693861	-1.319588
66	1	0	-3.057811	1.142754	-1.489079
67	8	0	-3.714634	0.802722	0.462686
68	6	0	-4.252296	1.978459	0.855059

69	8	0	-4.724569	2.762465	0.063819
70	6	0	-4.167391	2.162041	2.366387
71	6	0	-2.696744	2.432725	2.734815
72	1	0	-2.333830	3.348934	2.257902
73	1	0	-2.612887	2.558721	3.818967
74	1	0	-2.044040	1.608565	2.437576
75	6	0	-5.029944	3.361115	2.767043
76	1	0	-6.082122	3.195332	2.517512
77	1	0	-4.955523	3.517268	3.847618
78	1	0	-4.700002	4.274278	2.265025
79	6	0	-4.666466	0.889772	3.069377
80	1	0	-4.604108	1.027482	4.153493
81	1	0	-5.711531	0.682798	2.815099
82	1	0	-4.070248	0.015482	2.797456
83	8	0	-1.237186	1.393675	-2.551910
84	6	0	-1.683151	0.915122	-3.793242
85	1	0	-2.439626	0.118829	-3.656914
86	6	0	-2.366356	2.078499	-4.510210
87	1	0	-2.778767	1.762029	-5.474711
88	1	0	-3.182493	2.475029	-3.897866
89	1	0	-1.647060	2.886586	-4.686213
90	6	0	-0.549228	0.332006	-4.638320
91	1	0	0.224424	1.089598	-4.810719
92	1	0	-0.085434	-0.520159	-4.126936
93	1	0	-0.916864	-0.018942	-5.609645
94	6	0	-4.363678	-1.914453	-0.680666
95	6	0	-5.634192	-1.786534	-0.167762
96	8	0	-4.019655	-3.231614	-0.735865
97	6	0	-6.087197	-3.098610	0.101488
98	1	0	-6.166449	-0.860113	-0.003499
99	6	0	-5.062667	-3.926683	-0.265731
100	1	0	-7.039894	-3.397666	0.511967
101	1	0	-4.946990	-5.000519	-0.245181
102	1	0	0.838572	6.367079	-1.907836
103	1	0	-1.499049	6.650548	-1.130976
104	1	0	-2.735387	4.705310	-0.187843
105	1	0	4.077434	-0.016195	-4.171510
106	1	0	5.990333	1.343845	-3.365441
107	1	0	5.712733	2.836131	-1.402410
108	1	0	-0.263043	-4.545292	-4.011683
109	1	0	-0.912790	-6.565059	-2.719373
110	1	0	-0.971708	-6.480368	-0.240662
111	1	0	-2.108299	-0.437813	3.944073
112	1	0	-0.817475	-1.900262	5.476537
113	1	0	0.999256	-3.337158	4.584655

TS4b(Segphos)

RwB97XD SCF energy -3497.738313 a.u.

RwB97XD SCF enthalpy -3496.773588 a.u.

RwB97XD SCF free energy -3496.928626 a.u.

Three lowest frequencies (cm^{-1}) -987.6, 17.2, 19.3

Imaginary frequency (cm^{-1}) -987.6

Cartesian coordinates:

Standard orientation:

Center Number	Atomic Number	Atomic Type	Coordinates (Angstroms) X	Y	Z
1	6	0	2.878753	-0.903934	0.912367
2	6	0	3.325673	-1.933536	1.709791
3	6	0	3.180344	-1.936025	3.094673
4	6	0	2.606882	-0.880644	3.761557
5	6	0	2.154341	0.188973	2.975385
6	6	0	2.270629	0.190256	1.586756
7	1	0	2.502357	-0.871933	4.839881
8	1	0	1.703749	1.032317	3.483183
9	6	0	3.060881	-0.998894	-0.560037
10	6	0	2.007416	-1.198864	-1.495216
11	6	0	4.330504	-0.914324	-1.086374
12	6	0	2.283325	-1.309823	-2.855924
13	6	0	4.590577	-0.999413	-2.451603
14	6	0	3.586974	-1.210643	-3.365247
15	1	0	1.480127	-1.479871	-3.561719
16	1	0	3.786129	-1.298084	-4.426607
17	8	0	3.683411	-3.083879	3.603671
18	8	0	3.923804	-3.082014	1.310027
19	8	0	5.491132	-0.731549	-0.410136
20	8	0	5.919848	-0.868580	-2.670963
21	6	0	4.142540	-3.857350	2.490304
22	1	0	3.569561	-4.786909	2.429668
23	1	0	5.211768	-4.054914	2.603433
24	6	0	6.516962	-0.607471	-1.397089
25	1	0	7.298350	-1.345836	-1.202722
26	1	0	6.918235	0.410688	-1.384268
27	15	0	0.282215	-1.153995	-0.898496
28	15	0	1.534299	1.536320	0.602535
29	46	0	-0.415441	0.982997	-0.443284
30	6	0	0.203148	-2.342328	0.469801
31	6	0	0.825206	-3.590783	0.349949
32	6	0	-0.443347	-1.991997	1.656890
33	6	0	0.801680	-4.479438	1.418388
34	1	0	1.339059	-3.860623	-0.567998
35	6	0	-0.446917	-2.879763	2.730068
36	1	0	-0.936183	-1.028015	1.745689
37	6	0	0.176150	-4.119593	2.611846
38	6	0	-0.756459	-1.795238	-2.248372
39	6	0	-1.403535	-3.028821	-2.153080
40	6	0	-0.942418	-1.000464	-3.387905
41	6	0	-2.208291	-3.473525	-3.200110
42	1	0	-1.290465	-3.646235	-1.268736
43	6	0	-1.732644	-1.456196	-4.435431
44	1	0	-0.474959	-0.021519	-3.453152
45	6	0	-2.366805	-2.694864	-4.342191
46	6	0	1.168584	2.879891	1.772211
47	6	0	1.915256	4.057444	1.796716
48	6	0	0.076605	2.729844	2.637360
49	6	0	1.579148	5.073328	2.690540
50	1	0	2.751177	4.197344	1.119808
51	6	0	-0.245992	3.738778	3.535003
52	1	0	-0.523984	1.824451	2.603800
53	6	0	0.504815	4.915176	3.559687
54	6	0	2.837330	2.088503	-0.531523
55	6	0	2.545365	2.269457	-1.885912
56	6	0	4.143077	2.274283	-0.063370
57	6	0	3.558729	2.622503	-2.772063
58	1	0	1.533016	2.112055	-2.247964
59	6	0	5.147739	2.641944	-0.951191
60	1	0	4.376662	2.117578	0.985529
61	6	0	4.858531	2.805949	-2.305335
62	8	0	-2.365364	0.474872	-1.161757
63	6	0	-3.410560	0.870454	-0.611827
64	6	0	-3.374137	1.674760	0.635091
65	1	0	-4.290248	2.052601	1.075726
66	1	0	-2.443881	1.726505	1.190035
67	8	0	-3.634026	-0.120605	1.471889
68	6	0	-4.852687	-0.344798	1.855531
69	8	0	-5.690526	0.535356	2.067003
70	6	0	-5.214497	-1.841213	1.964384
71	6	0	-6.567403	-2.009494	2.656587
72	1	0	-7.356182	-1.480267	2.115369
73	1	0	-6.833391	-3.071392	2.707745
74	1	0	-6.541244	-1.615556	3.677702
75	6	0	-5.289653	-2.388769	0.526494
76	1	0	-4.332816	-2.266626	0.009542
77	1	0	-5.534841	-3.456847	0.544811
78	1	0	-6.063425	-1.875049	-0.055485
79	6	0	-4.123044	-2.590282	2.739035
80	1	0	-4.379386	-3.652008	2.828044
81	1	0	-3.161906	-2.510408	2.227393
82	1	0	-4.005696	-2.186085	3.751134
83	8	0	-0.908810	2.998225	-0.239021
84	6	0	-2.064966	3.542984	-0.569305
85	1	0	-3.007112	2.842276	-0.073930
86	6	0	-2.329697	4.849219	0.166757
87	1	0	-3.345071	5.212907	-0.011713
88	1	0	-2.172138	4.710809	1.239372
89	1	0	-1.618289	5.601473	-0.191791
90	6	0	-2.429936	3.558106	-2.047397
91	1	0	-1.788065	4.295002	-2.543871
92	1	0	-2.250276	2.584983	-2.511352
93	1	0	-3.473378	3.845364	-2.203321
94	6	0	-4.680568	0.635373	-1.245349
95	6	0	-5.960687	1.040724	-0.954289
96	8	0	-4.681542	-0.128180	-2.372695
97	6	0	-6.788220	0.487310	-1.961892
98	1	0	-6.263339	1.636008	-0.106544
99	6	0	-5.953406	-0.213342	-2.785389
100	1	0	-7.857623	0.591812	-2.067407
101	1	0	-6.125208	-0.794720	-3.679509
102	1	0	3.334509	2.748224	-3.826358
103	1	0	5.648988	3.076830	-2.998187
104	1	0	6.159695	2.787989	-0.587059
105	1	0	2.160207	5.989963	2.702180

106	1	0	0.246239	5.707858	4.254741
107	1	0	-1.088825	3.613554	4.207251
108	1	0	-1.865103	-0.839370	-5.318543
109	1	0	-2.992236	-3.046560	-5.156908
110	1	0	-2.709304	-4.432716	-3.117997
111	1	0	1.281853	-5.447981	1.322972
112	1	0	0.173884	-4.809725	3.449782
113	1	0	-0.939587	-2.601796	3.656205

11b(Segphos)

Standard orientation:

Center Number	Atomic Number	Atomic Type	Coordinates (Angstroms) X	Y	Z
1	6	0	-2.503999	-1.497396	-0.653404
2	6	0	-2.730685	-2.726759	-1.227466
3	6	0	-2.674252	-2.939753	-2.602772
4	6	0	-2.422139	-1.915462	-3.483772
5	6	0	-2.196987	-0.647947	-2.926903
6	6	0	-2.223901	-0.431589	-1.551149
7	1	0	-2.391660	-2.075902	-4.554728
8	1	0	-1.997969	0.173166	-3.604006
9	6	0	-2.585207	-1.345170	0.822156
10	6	0	-1.482075	-1.070286	1.677447
11	6	0	-3.814616	-1.455192	1.432517
12	6	0	-1.675831	-0.931047	3.050603
13	6	0	-3.998834	-1.290152	2.802591
14	6	0	-2.942843	-1.036718	3.644268
15	1	0	-0.831290	-0.731358	3.698351
16	1	0	-3.077497	-0.917817	4.712665
17	8	0	-2.911246	-4.244078	-2.870592
18	8	0	-3.006413	-3.891588	-0.593586
19	8	0	-5.006550	-1.701313	0.836458
20	8	0	-5.310304	-1.429812	3.105907
21	6	0	-3.079315	-4.896204	-1.607134
22	1	0	-2.273955	-5.622355	-1.462641
23	1	0	-4.060011	-5.377134	-1.572725
24	6	0	-5.996371	-1.615713	1.863204
25	1	0	-6.564890	-2.547988	1.898388
26	1	0	-6.646942	-0.756449	1.673109
27	15	0	0.166576	-0.754217	0.951384
28	15	0	-1.793582	1.190556	-0.855591
29	46	0	0.278036	1.360514	0.097691
30	6	0	0.454330	-2.108883	-0.216738
31	6	0	0.167574	-3.426844	0.154752
32	6	0	0.914524	-1.826920	-1.503915
33	6	0	0.352287	-4.458168	-0.760611
34	1	0	-0.215736	-3.648319	1.146438
35	6	0	1.073656	-2.858496	-2.422395
36	1	0	1.153840	-0.806062	-1.779154
37	6	0	0.795395	-4.173141	-2.051150
38	6	0	1.331580	-0.877455	2.341239
39	6	0	2.070311	-2.038899	2.569559
40	6	0	1.452952	0.205501	3.220368
41	6	0	2.919793	-2.117048	3.669834
42	1	0	2.000726	-2.879012	1.889339
43	6	0	2.294101	0.119037	4.323300
44	1	0	0.894936	1.120220	3.043513
45	6	0	3.031285	-1.042240	4.547440
46	6	0	-1.762017	2.375877	-2.231670
47	6	0	-2.693078	3.411412	-2.328782
48	6	0	-0.716797	2.286512	-3.161121
49	6	0	-2.584603	4.344584	-3.357702
50	1	0	-3.497592	3.500855	-1.606631
51	6	0	-0.619346	3.215136	-4.189622
52	1	0	0.025129	1.496705	-3.075956
53	6	0	-1.552768	4.247096	-4.286973
54	6	0	-3.143974	1.641232	0.268191
55	6	0	-2.848132	2.140073	1.539396
56	6	0	-4.475451	1.446538	-0.117452
57	6	0	-3.879113	2.437062	2.425571
58	1	0	-1.812940	2.268912	1.842778
59	6	0	-5.501089	1.754842	0.769130
60	1	0	-4.710158	1.042844	-1.097910
61	6	0	-5.203791	2.242165	2.041631
62	8	0	2.280681	1.623161	0.753021
63	6	0	3.219996	1.703433	-0.068362
64	6	0	3.035011	2.008532	-1.519035
65	1	0	3.674543	1.339046	-2.106914
66	1	0	1.994391	1.859169	-1.812859
67	8	0	3.402219	-0.702092	-0.477010
68	6	0	4.219830	-1.105967	-1.350857
69	8	0	4.542668	-0.499028	-2.401465
70	6	0	4.944014	-2.459978	-1.087322
71	6	0	4.953794	-3.291588	-2.374977
72	1	0	5.401445	-2.723622	-3.194213
73	1	0	5.519252	-4.221864	-2.238296
74	1	0	3.933017	-3.562658	-2.670847
75	6	0	6.387922	-2.117830	-0.682850
76	1	0	6.406783	-1.521587	0.237885
77	1	0	6.973245	-3.028912	-0.505527
78	1	0	6.880584	-1.538161	-1.469736
79	6	0	4.269938	-3.250846	0.033100
80	1	0	4.788464	-4.203911	0.200008
81	1	0	4.274926	-2.684385	0.967050
82	1	0	3.225728	-3.472456	-0.213553
83	8	0	0.337154	3.447685	-0.458544
84	6	0	0.384979	4.418192	0.305410
85	1	0	3.301499	3.057578	-1.694058
86	6	0	0.521477	5.783855	-0.281557
87	1	0	1.548301	6.126559	-0.106851
88	1	0	0.323063	5.765570	-1.353759
89	1	0	-0.144359	6.487094	0.225476
90	6	0	0.324784	4.286105	1.790212
91	1	0	-0.583029	4.785279	2.146441
92	1	0	0.321554	3.242882	2.110130
93	1	0	1.174809	4.812614	2.235865
94	6	0	4.579749	1.698503	0.439089
95	6	0	5.786462	1.799828	-0.197659
96	8	0	4.770796	1.505135	1.770297
97	6	0	6.779758	1.652549	0.809301
98	1	0	5.937500	1.931190	-1.258533
99	6	0	6.099075	1.474603	1.977380
100	1	0	7.852519	1.672011	0.684997
101	1	0	6.416825	1.326744	2.999074
102	1	0	0.136564	-5.481412	-0.470262
103	1	0	0.921272	-4.976145	-2.770912
104	1	0	1.419399	-2.636334	-3.426840
105	1	0	-3.646839	2.810657	3.417626
106	1	0	-6.007113	2.467344	2.736006
107	1	0	-6.533636	1.604162	0.470866
108	1	0	-3.311452	5.147265	-3.429948
109	1	0	-1.471892	4.978134	-5.085331
110	1	0	0.188630	3.138826	-4.910098
111	1	0	2.379826	0.961392	5.002358
112	1	0	3.695663	-1.106622	5.403718
113	1	0	3.493750	-3.022894	3.837598

13

RwB97XD SCF energy -4678.375067 a.u.

RwB97XD SCF enthalpy -4676.473232 a.u.

RwB97XD SCF free energy -4676.705155 a.u.

Three lowest frequencies (cm^{-1}) 18.5, 193, 21.3

Cartesian coordinates

Standard orientation:

Center Number	Atomic Number	Atomic Type	Coordinates X	Y	(Angstroms) Z
1	6	0	-0.195503	-1.118353	2.235454
2	6	0	0.210653	-2.118298	3.093572
3	6	0	-0.273301	-3.420829	3.026899
4	6	0	-1.195785	-3.797813	2.083258
5	6	0	-1.608178	-2.806926	1.181370
6	6	0	-1.128573	-1.498478	1.229847
7	1	0	-1.594503	-4.804061	2.035201
8	1	0	-2.340832	-3.087001	0.436569
9	6	0	0.292619	0.262555	2.495893
10	6	0	1.144922	1.030452	1.657694
11	6	0	-0.074450	0.852084	3.686301
12	6	0	1.547175	2.307541	2.044032
13	6	0	0.322270	2.132581	4.056877
14	6	0	1.134252	2.892991	3.251240
15	1	0	2.214268	2.880583	1.412615
16	1	0	1.454696	3.888968	3.533457
17	8	0	0.293676	-4.169658	4.004488
18	8	0	1.110129	-2.019489	4.106447
19	8	0	-0.871982	0.328386	4.656467
20	8	0	-0.220632	2.440845	5.265558

21	6	0	1.049077	-3.259147	4.809622
22	1	0	2.056705	-3.652595	4.949689
23	1	0	0.538175	-3.112575	5.767941
24	6	0	-0.714963	1.199851	5.774718
25	1	0	0.019847	0.773376	6.469252
26	1	0	-1.680935	1.355856	6.254847
27	15	0	1.640103	0.301883	0.054609
28	15	0	-1.625084	-0.306399	-0.068667
29	46	0	-0.002510	0.202631	-1.574155
30	6	0	2.319229	-1.321000	0.487149
31	6	0	3.344810	-1.438671	1.414495
32	6	0	1.831492	-2.456584	-0.151070
33	6	0	3.921073	-2.674465	1.723020
34	1	0	3.724936	-0.540474	1.889529
35	6	0	2.296241	-3.727639	0.170398
36	1	0	1.056897	-2.326504	-0.895673
37	6	0	3.309650	-3.816201	1.158059
38	6	0	3.027260	1.283562	-0.591266
39	6	0	4.246182	0.693366	-0.918581
40	6	0	2.806128	2.611063	-0.941062
41	6	0	5.294636	1.439421	-1.455908
42	1	0	4.381700	-0.365224	-0.743761
43	6	0	3.819532	3.421619	-1.457938
44	1	0	1.815245	3.030179	-0.801893
45	6	0	5.095572	2.833821	-1.586096
46	6	0	-3.061969	-1.035504	-0.897841
47	6	0	-4.305144	-0.414607	-0.850652
48	6	0	-2.896931	-2.170573	-1.680165
49	6	0	-5.416519	-0.966415	-1.483212
50	1	0	-4.404737	0.518211	-0.314840
51	6	0	-3.969348	-2.789279	-2.326075
52	1	0	-1.902776	-2.589153	-1.788066
53	6	0	-5.244080	-2.215502	-2.127359
54	6	0	-2.246597	1.163459	0.789853
55	6	0	-1.983349	2.425278	0.279282
56	6	0	-3.040845	1.031786	1.930278
57	6	0	-2.545510	3.581528	0.834543
58	1	0	-1.323730	2.509935	-0.577162
59	6	0	-3.671709	2.133433	2.497895
60	1	0	-3.175135	0.047290	2.361215
61	6	0	-3.486877	3.389386	1.864024
62	6	0	-2.057533	4.941791	0.281956
63	6	0	-2.637041	5.181286	-1.123413
64	1	0	-2.284931	6.142377	-1.515169
65	1	0	-2.317922	4.399296	-1.818048
66	1	0	-3.730888	5.199725	-1.117653
67	6	0	-2.369419	6.150953	1.181664
68	1	0	-2.050564	5.976304	2.213130
69	1	0	-1.815694	7.015246	0.799191
70	1	0	-3.424839	6.421672	1.193228
71	6	0	-0.517104	4.894133	0.162689
72	1	0	-0.051280	4.713405	1.137042
73	1	0	-0.171285	4.123076	-0.530513
74	1	0	-0.151742	5.853858	-0.217286
75	6	0	-4.481090	1.984722	3.802958
76	6	0	-3.923098	2.963841	4.854828
77	1	0	-4.094773	4.001609	4.565693
78	1	0	-4.408099	2.791335	5.822165
79	1	0	-2.845332	2.822904	4.982786
80	6	0	-5.982460	2.251393	3.589027
81	1	0	-6.382008	1.635274	2.776011
82	1	0	-6.531831	1.995980	4.502256
83	1	0	-6.186077	3.298967	3.367239
84	6	0	-4.349544	0.563834	4.377195
85	1	0	-4.800279	-0.189116	3.721477
86	1	0	-3.305241	0.288956	4.553900
87	1	0	-4.877409	0.518097	5.334822
88	8	0	-4.250714	4.436531	2.311508
89	8	0	-6.370011	-2.855017	-2.585252
90	8	0	3.719384	-5.070012	1.530372
91	8	0	6.191181	3.614877	-1.852128
92	6	0	6.697219	4.267783	-0.691907
93	1	0	7.616681	4.772872	-0.993158
94	1	0	5.989889	5.007671	-0.301689
95	1	0	6.918594	3.547635	0.103544
96	6	0	3.425031	4.863870	-1.851966
97	6	0	4.468061	5.630831	-2.683734
98	1	0	4.004991	6.557414	-3.040280
99	1	0	5.355803	5.910921	-2.116533
100	1	0	4.787698	5.058412	-3.558768
101	6	0	3.101070	5.666511	-0.577904
102	1	0	2.774691	6.679064	-0.840459
103	1	0	2.299406	5.193145	-0.002084
104	1	0	3.976552	5.752628	0.073746
105	6	0	-5.345555	4.745454	1.454512
106	1	0	-5.974001	5.457125	1.992930
107	1	0	-5.012019	5.201861	0.517698
108	1	0	-5.929148	3.848987	1.216558
109	6	0	-6.816323	-3.889215	-1.714310
110	1	0	-7.746290	-4.276126	-2.135308
111	1	0	-6.088068	-4.703533	-1.644400
112	1	0	-7.004280	-3.505672	-0.705609
113	6	0	-6.741146	-0.174458	-1.533184
114	6	0	-6.591880	1.208615	-0.871995
115	1	0	-6.385726	1.131597	0.201334
116	1	0	-5.800995	1.807841	-1.335578
117	1	0	-7.532008	1.757279	-0.987093
118	6	0	-7.119731	0.058177	-3.009235
119	1	0	-7.259863	-0.888322	-3.535565
120	1	0	-8.054773	0.626694	-3.070297
121	1	0	-6.341089	0.631147	-3.524656
122	6	0	-7.895588	-0.896242	-0.813967
123	1	0	-8.219000	-1.785925	-1.354100
124	1	0	-7.612554	-1.184010	0.204362
125	1	0	-8.757349	-0.223339	-0.742320
126	6	0	-3.631343	-4.020442	-3.203168
127	6	0	-3.252741	-5.204694	-2.293620
128	1	0	-2.402965	-4.954095	-1.649247
129	1	0	-4.085909	-5.501734	-1.648758
130	1	0	-2.969212	-6.072323	-2.900417
131	6	0	-4.741955	-4.457817	-4.174795
132	1	0	-4.327000	-5.206703	-4.858479
133	1	0	-5.597736	-4.915556	-3.678805
134	1	0	-5.103276	-3.619326	-4.776044
135	6	0	-2.411181	-3.679122	-4.090233
136	1	0	-2.185747	-4.530484	-4.741012
137	1	0	-2.619838	-2.811947	-4.725294
138	1	0	-1.505274	-3.469029	-3.515395
139	6	0	6.596305	0.733321	-1.893551
140	6	0	2.156831	4.796615	-2.734169
141	1	0	2.350729	4.234342	-3.654155
142	1	0	1.305582	4.334989	-2.228807
143	1	0	1.854316	5.811028	-3.014559
144	6	0	7.044860	1.220427	-3.285571
145	1	0	7.948410	0.678913	-3.586524
146	1	0	6.273375	1.024368	-4.037615
147	1	0	7.265059	2.287490	-3.291745
148	6	0	7.727204	0.972031	-0.876710
149	1	0	7.408082	0.714278	0.139084
150	1	0	8.589315	0.343779	-1.128208
151	1	0	8.065539	2.010333	-0.882078
152	6	0	6.379447	-0.786549	-2.009416
153	1	0	5.549727	-1.028985	-2.683511
154	1	0	7.285010	-1.244779	-2.418799
155	1	0	6.187730	-1.262682	-1.043531
156	6	0	3.201873	-5.493043	2.787768
157	1	0	3.556379	-6.513286	2.945355
158	1	0	3.562067	-4.862628	3.606491
159	1	0	2.107761	-5.481042	2.792684
160	6	0	5.192821	-2.644577	2.608537
161	6	0	4.815295	-2.306859	4.062258
162	1	0	5.722071	-2.209576	4.669721
163	1	0	4.265300	-1.362339	4.122278
164	1	0	4.195134	-3.087653	4.512317
165	6	0	6.038848	-3.931245	2.581148
166	1	0	6.994275	-3.724301	3.075188
167	1	0	5.581135	-4.768011	3.107497
168	1	0	6.253113	-4.247958	1.556736
169	6	0	6.129608	-1.530938	2.080722
170	1	0	6.412851	-1.723415	1.041293
171	1	0	5.689219	-0.532686	2.136601
172	1	0	7.046189	-1.512250	2.679342
173	6	0	1.782461	-4.950713	-0.619547
174	6	0	0.644169	-4.556257	-1.579442
175	1	0	0.307406	-5.446585	-2.119323
176	1	0	-0.217904	-4.148239	-1.039222
177	1	0	0.963389	-3.824694	-2.328309
178	6	0	1.233954	-6.071292	0.283499
179	1	0	0.522592	-5.679583	1.017373
180	1	0	0.705068	-6.805733	-0.333557
181	1	0	2.030189	-6.596772	0.810014
182	6	0	2.946489	-5.502111	-1.464819
183	1	0	3.310471	-4.746451	-2.170184
184	1	0	3.780176	-5.813765	-0.830543

185	1	0	2.613725	-6.371813	-2.042965
186	8	0	1.410565	0.847019	-3.076097
187	6	0	2.255594	0.151528	-3.646114
188	6	0	2.321264	-1.325859	-3.439280
189	1	0	2.833304	-1.829367	-4.261281
190	1	0	1.317954	-1.735301	-3.299017
191	8	0	-1.407341	0.011335	-3.001150
192	6	0	-1.482374	0.959046	-4.026396
193	1	0	-0.552361	0.977260	-4.624583
194	6	0	-2.624079	0.536347	-4.951298
195	1	0	-2.736904	1.232699	-5.790199
196	1	0	-2.439592	-0.463733	-5.356261
197	1	0	-3.567041	0.506937	-4.392489
198	6	0	-1.717076	2.371643	-3.489341
199	1	0	-2.642210	2.393760	-2.900663
200	1	0	-0.889103	2.678011	-2.838808
201	1	0	-1.800345	3.107622	-4.297953
202	6	0	3.252322	0.796649	-4.547564
203	1	0	2.892430	-1.498487	-2.517666
204	1	0	4.228897	0.321929	-4.423434
205	1	0	3.314684	1.869974	-4.363495
206	1	0	2.939494	0.619444	-5.584184

14•acetone

RwB97XD SCF energy -4679.539687 a.u.

RwB97XD SCF enthalpy -4677.621165 a.u.

RwB97XD SCF free energy -4677.858751 a.u.

Three lowest frequencies (cm^{-1}) 15.2, 17.3, 19.6

Cartesian coordinates

Standard orientation:

Center Number	Atomic Number	Atomic Type	Coordinates (Angstroms) X	Y	Z
1	6	0	-0.089701	-0.830035	2.464943
2	6	0	0.344764	-1.702763	3.440679
3	6	0	-0.027232	-3.043614	3.482238
4	6	0	-0.852208	-3.592805	2.532292
5	6	0	-1.295951	-2.733083	1.517760
6	6	0	-0.935943	-1.387043	1.463964
7	1	0	-1.159830	-4.630993	2.568382
8	1	0	-1.963151	-3.144483	0.772047
9	6	0	0.263926	0.608881	2.601368
10	6	0	1.061201	1.372557	1.706126
11	6	0	-0.197630	1.277419	3.714490
12	6	0	1.317489	2.718721	1.958637
13	6	0	0.057302	2.624343	3.954266
14	6	0	0.810644	3.379338	3.088588
15	1	0	1.938892	3.287718	1.277564
16	1	0	1.016199	4.427533	3.270150
17	8	0	0.529483	-3.637863	4.565397
18	8	0	1.160176	-1.427135	4.491221
19	8	0	-0.977771	0.781534	4.712739
20	8	0	-0.554559	2.999895	5.108637
21	6	0	1.125603	-2.583225	5.326455
22	1	0	2.140768	-2.865274	5.607986
23	1	0	0.507068	-2.379731	6.208196
24	6	0	-0.944703	1.775708	5.735663
25	1	0	-0.196468	1.498161	6.488423
26	1	0	-1.937222	1.885440	6.172445
27	15	0	1.681340	0.564023	0.193712
28	15	0	-1.496809	-0.362357	0.057185
29	46	0	0.134430	0.116241	-1.468372
30	6	0	2.495841	-0.950261	0.752135
31	6	0	3.480220	-0.930784	1.729746
32	6	0	2.114412	-2.159407	0.176955
33	6	0	4.097167	-2.105955	2.170965
34	1	0	3.780705	0.022815	2.152375
35	6	0	2.634252	-3.371281	0.616798
36	1	0	1.371844	-2.138936	-0.609788
37	6	0	3.582535	-3.321293	1.668764
38	6	0	2.925523	1.648450	-0.564729
39	6	0	4.232509	1.223167	-0.785146
40	6	0	2.478282	2.821210	-1.164332
41	6	0	5.126157	1.995485	-1.526630
42	1	0	4.545751	0.266857	-0.388790
43	6	0	3.320057	3.645124	-1.913853
44	1	0	1.431431	3.086231	-1.060615
45	6	0	4.674258	3.253733	-1.993668
46	6	0	-2.855775	-1.250310	-0.746281
47	6	0	-4.124150	-0.683001	-0.803579
48	6	0	-2.608863	-2.436478	-1.424342
49	6	0	-5.182125	-1.329136	-1.435546
50	1	0	-4.286895	0.282709	-0.348784
51	6	0	-3.625357	-3.147862	-2.066195
52	1	0	-1.593522	-2.814348	-1.460204
53	6	0	-4.929553	-2.612803	-1.976412
54	6	0	-2.257466	1.122912	0.756063
55	6	0	-2.114032	2.346478	0.120389
56	6	0	-3.088617	1.014918	1.873528
57	6	0	-2.852374	3.471123	0.512835
58	1	0	-1.415107	2.423052	-0.706930
59	6	0	-3.879811	2.081166	2.284448
60	1	0	-3.128732	0.071056	2.403238
61	6	0	-3.826453	3.266180	1.507183
62	6	0	-2.493714	4.820995	-0.150612
63	6	0	-2.875156	4.798657	-1.641159
64	1	0	-2.602090	5.749632	-2.112021
65	1	0	-2.352904	3.999500	-2.174559
66	1	0	-3.950503	4.649473	-1.781300
67	6	0	-3.110566	6.067560	0.507908
68	1	0	-2.942998	6.081976	1.587851
69	1	0	-2.624837	6.951180	0.079493
70	1	0	-4.180491	6.169459	0.324795
71	6	0	-0.963567	5.001556	-0.037186
72	1	0	-0.649782	5.041762	1.011181
73	1	0	-0.410707	4.194843	-0.523862
74	1	0	-0.668598	5.938233	-0.520942
75	6	0	-4.717591	1.990553	3.575362
76	6	0	-4.287506	3.134389	4.515707
77	1	0	-4.530197	4.110037	4.090149
78	1	0	-4.799496	3.040964	5.479841
79	1	0	-3.208136	3.103287	4.696202
80	6	0	-6.230745	2.083590	3.305102
81	1	0	-6.543685	1.357950	2.546221
82	1	0	-6.779401	1.859587	4.226826
83	1	0	-6.527603	3.081125	2.980816
84	6	0	-4.465638	0.661741	4.306491
85	1	0	-4.826576	-0.198351	3.731677
86	1	0	-3.404940	0.509440	4.527028
87	1	0	-5.009139	0.670324	5.256511
88	8	0	-4.757628	4.237171	1.768237
89	8	0	-6.005322	-3.324754	-2.445785
90	8	0	4.023504	-4.511495	2.186074
91	8	0	5.598745	4.081707	-2.576918
92	6	0	6.056929	5.115053	-1.710766
93	1	0	6.836159	5.655276	-2.251715
94	1	0	5.254958	5.811427	-1.447128
95	1	0	6.476144	4.702126	-0.786873
96	6	0	2.664881	4.860994	-2.612857
97	6	0	3.493972	5.479073	-3.752740
98	1	0	2.865424	6.204158	-4.280678
99	1	0	4.378582	6.010690	-3.403538
100	1	0	3.812752	4.722112	-4.474805
101	6	0	2.346976	5.944554	-1.565625
102	1	0	1.793983	6.768299	-2.030908
103	1	0	1.734852	5.541206	-0.752984
104	1	0	3.258203	6.360533	-1.125187
105	6	0	-5.810890	4.259605	0.808512
106	1	0	-6.499529	5.049027	1.114885
107	1	0	-5.437401	4.474680	-0.197617
108	1	0	-6.343661	3.302092	0.784336
109	6	0	-6.466933	-4.313044	-1.530376
110	1	0	-7.376118	-4.739191	-1.958712
111	1	0	-5.731105	-5.110871	-1.387568
112	1	0	-6.694847	-3.873338	-0.553524
113	6	0	-6.534562	-0.600582	-1.592335
114	6	0	-6.478403	0.816455	-0.990955
115	1	0	-6.311870	0.795928	0.092000
116	1	0	-5.699602	1.436387	-1.448257
117	1	0	-7.438005	1.313289	-1.165343
118	6	0	-6.843462	-0.456086	-3.095177
119	1	0	-6.919256	-1.432106	-3.579626
120	1	0	-7.795324	0.069506	-3.232358
121	1	0	-6.061493	0.122890	-3.598736
122	6	0	-7.694930	-1.336958	-0.897877
123	1	0	-7.953446	-2.263928	-1.409640
124	1	0	-7.454425	-1.564322	0.146356
125	1	0	-8.586247	-0.699439	-0.902450
126	6	0	-3.202424	-4.431588	-2.822088
127	6	0	-2.873569	-5.537447	-1.801069
128	1	0	-2.074673	-5.220822	-1.121038
129	1	0	-3.744554	-5.803839	-1.194122

130	1	0	-2.534723	-6.441601	-2.319415
131	6	0	-4.228281	-4.958956	-3.841714
132	1	0	-3.752954	-5.756322	-4.423381
133	1	0	-5.121175	-5.382667	-3.383463
134	1	0	-4.540246	-4.175643	-4.538118
135	6	0	-1.921343	-4.132208	-3.634117
136	1	0	-1.648909	-5.016158	-4.220610
137	1	0	-2.078477	-3.298018	-4.325048
138	1	0	-1.062587	-3.893439	-3.002834
139	6	0	6.510427	1.426780	-1.907261
140	6	0	1.335577	4.400863	-3.258929
141	1	0	1.499854	3.568959	-3.952718
142	1	0	0.587682	4.094882	-2.524302
143	1	0	0.899119	5.231309	-3.823216
144	6	0	6.616536	1.379840	-3.444899
145	1	0	7.585109	0.959291	-3.737979
146	1	0	5.831634	0.741580	-3.865899
147	1	0	6.528162	2.375366	-3.884333
148	6	0	7.673470	2.253790	-1.328653
149	1	0	7.548970	2.412727	-0.252114
150	1	0	8.615099	1.714236	-1.478912
151	1	0	7.771731	3.222913	-1.817533
152	6	0	6.683227	-0.012079	-1.393038
153	1	0	5.908923	-0.687633	-1.771331
154	1	0	7.649394	-0.396477	-1.734727
155	1	0	6.680634	-0.058847	-0.299669
156	6	0	3.403227	-4.847149	3.424053
157	1	0	3.861299	-5.778515	3.762250
158	1	0	3.564579	-4.070607	4.178548
159	1	0	2.326540	-4.993492	3.300656
160	6	0	5.299892	-1.932833	3.132000
161	6	0	4.803617	-1.390354	4.484365
162	1	0	5.653376	-1.219268	5.154617
163	1	0	4.269966	-0.442131	4.368326
164	1	0	4.129282	-2.100781	4.971490
165	6	0	6.150039	-3.192104	3.380086
166	1	0	7.059380	-2.887803	3.909937
167	1	0	5.652388	-3.936012	4.002120
168	1	0	6.450151	-3.671405	2.445276
169	6	0	6.268056	-0.902889	2.504476
170	1	0	6.663039	-1.278332	1.554895
171	1	0	5.804752	0.069923	2.322192
172	1	0	7.114129	-0.738532	3.179940
173	6	0	2.261520	-4.672700	-0.125431
174	6	0	1.154712	-4.424228	-1.167811
175	1	0	0.929809	-5.364042	-1.681123
176	1	0	0.230718	-4.069174	-0.696119
177	1	0	1.453249	-3.698839	-1.929760
178	6	0	1.751305	-5.791394	0.800894
179	1	0	0.950430	-5.432500	1.455041
180	1	0	1.344224	-6.605644	0.191689
181	1	0	2.549529	-6.206519	1.415305
182	6	0	3.520290	-5.161266	-0.867800
183	1	0	3.874845	-4.402969	-1.574154
184	1	0	4.327448	-5.382021	-0.163817
185	1	0	3.298159	-6.075611	-1.430284
186	8	0	2.819681	-1.497471	-3.336016
187	6	0	2.395588	-2.384844	-4.062992
188	6	0	1.006603	-2.337813	-4.642597
189	1	0	1.065989	-1.819608	-5.608245
190	1	0	0.321437	-1.783946	-3.991859
191	8	0	-1.151602	-0.389269	-2.931191
192	6	0	-1.247212	0.458919	-4.040128
193	1	0	-0.269858	0.545219	-4.558805
194	6	0	-2.238037	-0.171419	-5.016784
195	1	0	-2.370934	0.452740	-5.907607
196	1	0	-1.884526	-1.156450	-5.337907
197	1	0	-3.211581	-0.301082	-4.529863
198	6	0	-1.681681	1.868829	-3.641467
199	1	0	-2.650075	1.830458	-3.128462
200	1	0	-0.948971	2.305519	-2.952083
201	1	0	-1.768025	2.532693	-4.509341
202	6	0	3.246417	-3.569171	-4.432579
203	1	0	0.621100	-3.341800	-4.837403
204	1	0	2.813103	-4.469326	-3.981097
205	1	0	4.271287	-3.439575	-4.081875
206	1	0	3.231521	-3.719294	-5.517317
207	1	0	1.517042	0.075204	-2.780663
208	1	0	1.241928	0.813667	-2.789760

----

3. General Details

All reactions were performed in a glove box under an atmosphere of dry nitrogen, and the workup was carried out in air, unless otherwise noted. Acetone was dried and distilled before use by standard procedures. Commercially available reagents were used without further purification. (±)-DTBM-Segphos was purchased from Strem Chemicals Inc. and used without further purification. GC-MS was performed at the Analysis Center of Shanghai Jiao Tong University (7890A-5975C).

4. General Procedure for Asymmetric Hydrogenation of α-Acyloxy-1-arylethanone

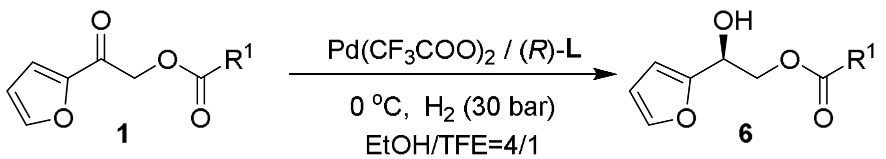

R^1=Me, ^{t}Bu, Ph

(*R*)-DTBM-SegPHOS (2.83 mg, 2.4 mol%) and $Pd(OCOCF_3)_2$ (0.66 mg, 2.0 mol%) were placed in a dried Schlenk tube under nitrogen atmosphere, and degassed anhydrous acetone was added. The mixture was stirred at room temperature for 10 min, then the solvent was removed under vacuum to give the dry catalyst. In a glovebox, substrate **1** (0.1 mmol) was stirred in a solvent (0.5 mL) at room temperature for 10 min. Subsequently, the above catalyst together with a mixed solvent (EtOH/TFE=4/1, 1.0 mL) was added to the reaction mixture. The hydrogenation was performed at 0 °C under H_2 (30 bar) in a stainless steel autoclave for 2 h. After carefully releasing the hydrogen, the conversion of the product **6** was determined by ^{1}H NMR spectroscopic analysis of the crude

reaction mixture. The enantiomeric excess of the products was determined by HPLC with chiral columns.

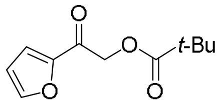

^{1}H NMR (400 MHz, $CDCl_3$): δ 7.60 (1H, dd, J = 3.2, 0.8 Hz), 7.26–7.24 (1H, m), 6.57 (1H, dd, J = 7.2, 3.2 Hz), 5.16 (2H, s), 1.29 (9H, s); ^{13}C NMR (100 MHz, $CDCl_3$): δ 182.2, 178.2, 150.9, 146.9, 117.8, 112.7, 65.4, 39.0, 27.4.

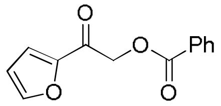

^{1}H NMR (400 MHz, $CDCl_3$): δ 8.11 (2H, d, J = 7.2 Hz), 7.61−7.55 (2H, m), 7.47−7.45 (2H, m), 7.30 (1H, s), 6.57 (1H, s), 5.40 (2H, s); ^{13}C NMR (100 MHz, $CDCl_3$): δ 181.6, 165.9, 150.6, 146.8, 133.4, 130.0, 128.4, 117.8, 112.5, 65.8; HRMS (ESI-MS) Calcd. For $C_{13}H_{10}O_4$ $[M+H]^+$ 231.0657, found: 231.0671.

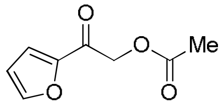

^{1}H NMR (400 MHz, $CDCl_3$): δ 7.55 (1H, s), 7.21 (1H, s), 6.51 (1H, s), 5.11 (2H, s), 2.14 (3H, s); ^{13}C NMR (100 MHz, $CDCl_3$): δ 181.7, 170.4, 150.4, 146.8, 117.8, 112.5, 65.3, 20.5; HRMS (ESI-MS) Calcd. For $C_8H_8O_4$ $[M+Na]^+$ 191.0320, found: 191.0314.

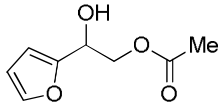

^{1}H NMR (400 MHz, $CDCl_3$): δ 7.38 (1H, s), 6.32 (2H, d, J = 7.6 Hz), 4.95 (1H, s), 4.35 (2H, d, J = 5.6 Hz), 2.52 (1H, s), 2.08 (3H, s); ^{13}C NMR (100 MHz, $CDCl_3$): δ 171.1, 152.6, 142.5, 110.3, 107.2, 66.5, 66.3, 20.8. The ee was determined by

HPLC on a Daicel Chiralcel IE-H column (hexane/2-propanol = 80/20, 210 nm, flow = 0.6 mL/min), t_{R1} = 21.3 min, t_{R2} = 31.5 min, ee = 64%. HRMS (ESI-MS) Calcd. For $C_8H_{10}O_4$ $[M+H]^+$ 171.0657, found: 171.0687.

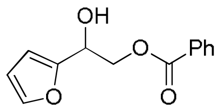

^1H NMR (400 MHz, $CDCl_3$): δ 8.02 (2H, d, J = 7.6 Hz), 7.58−7.54 (1H, m), 7.45−7.41 (3H, m), 6.37–6.35 (2H, m) 5.10 (2H, s), 4.61 (2H, d, J = 4.8 Hz); ^{13}C NMR (100 MHz, $CDCl_3$): δ 166.6, 152.7, 142.6, 133.3, 129.7, 128.4, 110.4, 107.4, 67.0, 66.5. The ee was determined by HPLC on a Daicel Chiralcel IE-H column (hexane/2-propanol = 90/10, 210 nm, flow = 0.8 mL/min), t_{R1} = 17.8 min, t_{R2} = 18.6 min, ee = 79%. HRMS (ESI-MS) Calcd. For $C_{13}H_{12}O_4$ $[M+Na]^+$ 255.0633, found: 255.0642.

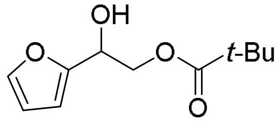

^1H NMR (400 MHz, $CDCl_3$): δ 7.39 (3H, dd, J = 2.0, 0.4 Hz), 6.35–6.31 (2H, m), 4.96 (1H, dd, J = 6.0, 4.8 Hz), 4.38–4.36 (2H, m), 1.10 (9H, s); ^{13}C NMR (100 MHz, $CDCl_3$): δ 178.9, 153.2, 142.7, 110.5, 107.5, 66.7, 66.6, 39.1, 27.3; The ee was determined by HPLC on a Daicel Chiralcel IE-H column (hexane/2-propanol = 95/5, 210 nm, flow = 0.6 mL/min), t_{R1} = 20.1 min, t_{R2} = 21.1 min, ee = 97%.

5. Palladium-Catalyzed Chemo- and Enantioselective C−O Bond Cleavage of 2-(Furan-2-yl)-2-oxoethyl pivalate via Hydrogenolysis

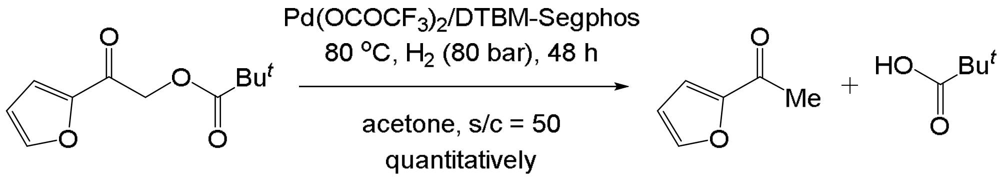

1a **12a**

(±)-DTBM-Segphos (2.2 mol%) and $Pd(OCOCF_3)_2$ (2.0 mol%) were placed in a dried Schlenk tube under a nitrogen atmosphere, and degassed anhydrous acetone (1.0 mL) was added. The mixture was stirred at room temperature for 10 min. In a glove box, substrate **1a** (45.9 mg, 0.3 mmol) was stirred in acetone (1.5 mL) at room temperature for 10 min. Subsequently, the above catalyst together with acetone was added to the reaction mixture. The hydrogenation was carried out at 80 °C under H_2 (80 bar) in a stainless steel autoclave for 48 h. After carefully releasing the hydrogen, the mixture was concentrated in vacuo to afford the corresponding crude product. The compound **1a** was completely transformed to **12a** and t-Butyl alcohol as determined by ^{1}H NMR analysis.

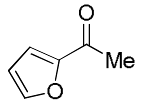

^{1}H NMR (400 MHz, $CDCl_3$): δ 7.56 (1H, s), 7.15 (1H, d, J = 2.8 Hz), 6.51−6.50 (1H, m), 2.45 (3H, s).

6. Mechanism Experiments. Relates to the Scheme 3.

Table S1 The results of mechanism experiments. Relates to Scheme 3.

entry	catalyst	solvent	substrate	isopropanol (GC-MS)
1	$Pd(OCOCF_3)_2$	acetone	no	No Detected
2	$Pd(OCOCF_3)_2$-DTBM-Segphos	acetone	no	Detected
3	$Pd(OCOCF_3)_2$-DTBM-Segphos	d6-acetone	no	Detected the d6-isopropanol
4	$Pd(OCOCF_3)_2$-DTBM-Segphos	acetone	yes	Detected

DTBM-Segphos (2.60 mg), $Pd(OCOCF_3)_2$ (0.66 mg), H_2 (30 bar), RT, acetone (1.0 mL) 12 h. Substrate: 2-oxo-2-phenylethyl pivalate (s/c = 50).

As shown in Table S1, no isopropanol was detected in the absence of DTBM-Segphos and substrate according to GC-MS analysis (entry 1). A trace amount of isopropanol was detected in the reaction mixture when the reaction was carried out in the presence of Pd-DTBM-Segphos catalyst (entry 2). When d6-acetone was used, a trace amount of d6-isopropanol was detected (entry 3). A small amount of isopropanol was also detected if the substrate (2-oxo-2-phenylethyl pivalate) was added. Moreover, isopropanol was also detected when **1a** was used as the substrate (see the above experiment). These results suggest the possibility that acetone takes part in the hydride transfer during the catalytic reaction.

Figure S1. ESI mass-spectra of the reaction mixtures described in the Table S1: Acetone (standard sample and entry 1, peak, m/z = 43). Relates to the Scheme 3.

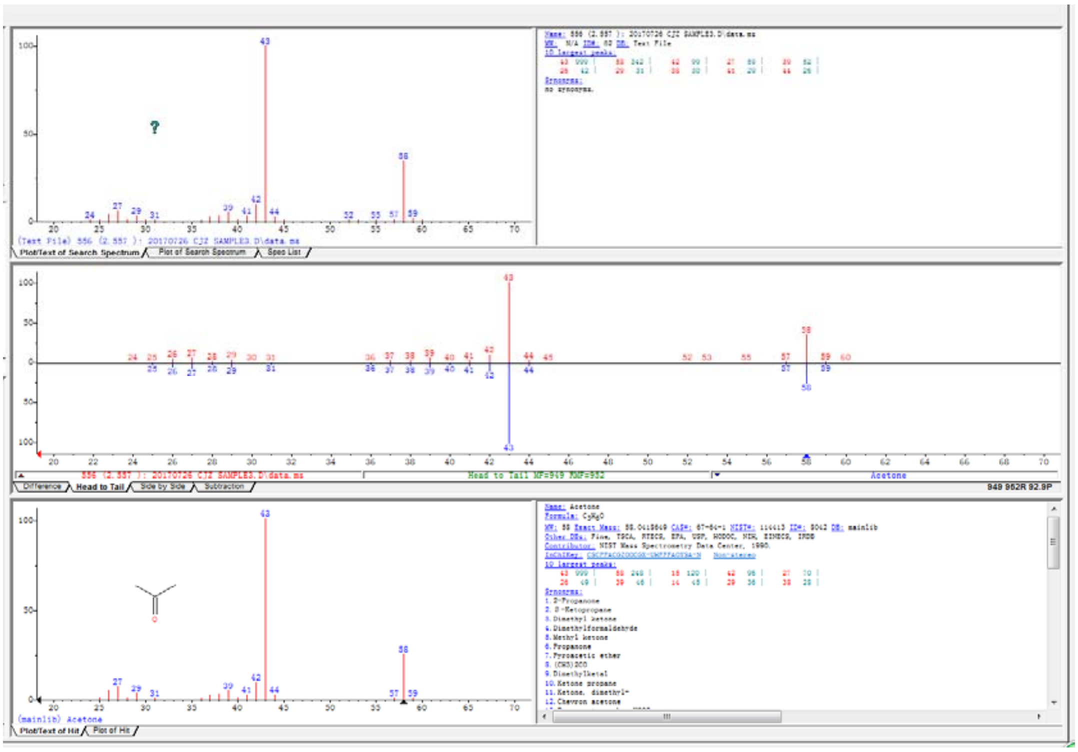

Figure S2. ESI mass-spectra of the reaction mixtures described in the Table S1: Acetone+H_2 (entry 2 and 4, peak, m/z = 45). Relates to the Scheme 3.

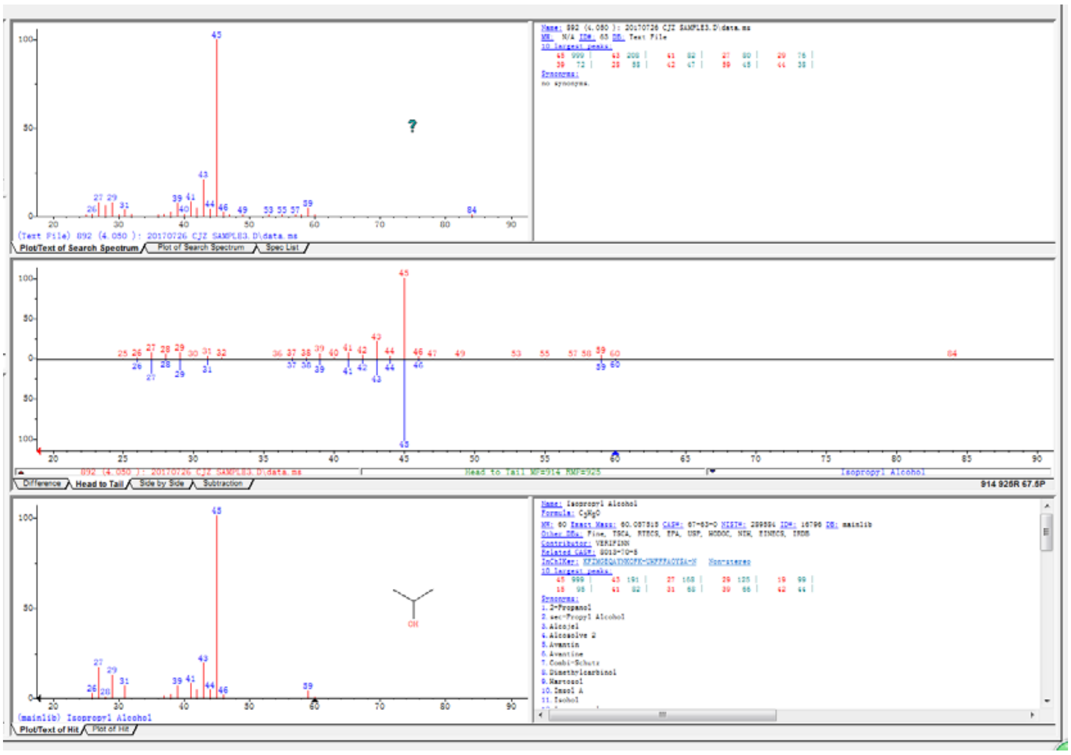

Figure S3. ESI mass-spectra of the reaction mixtures described in the Table S1: d_6-Acetone (standard sample, peak, m/z = 46). Relates to the Scheme 3.

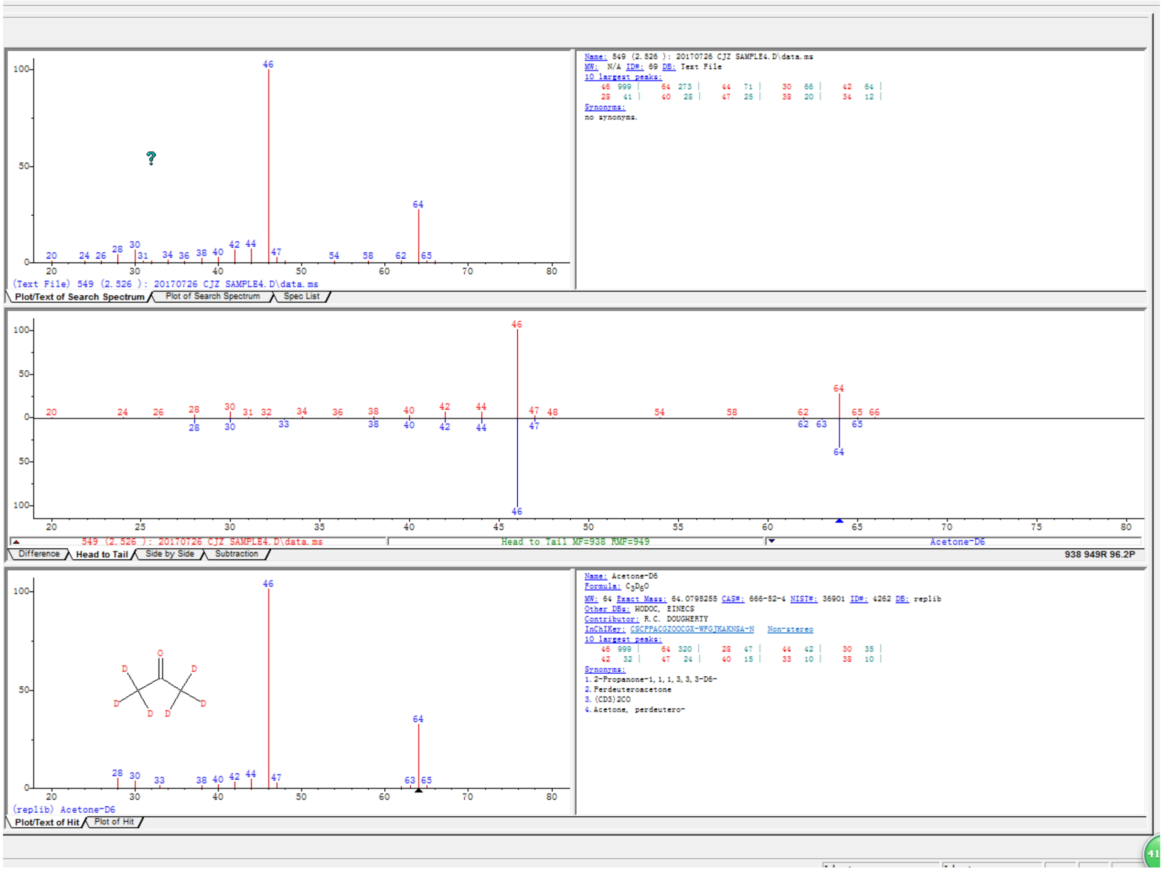

Figure S4. ESI mass-spectra of the reaction mixtures described in the Table S1: d_6-Acetone+H_2 (entry 3, peak, m/z = 48). Relates to the Scheme 3.

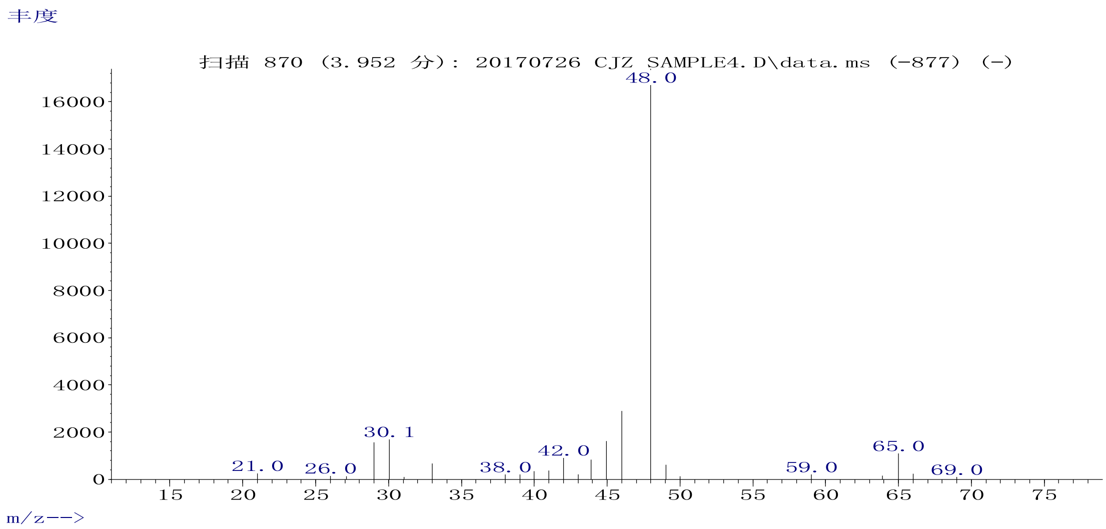

Supplement: Document S1. Transparent Methods, Figures S1–S4, Table S1, and Data S1 [file mmc1.pdf]
